# Hoveyda–Grubbs catalysts with an N→Ru coordinate bond in a six-membered ring. Synthesis of stable, industrially scalable, highly efficient ruthenium metathesis catalysts and 2-vinylbenzylamine ligands as their precursors

**DOI:** 10.3762/bjoc.15.73

**Published:** 2019-03-22

**Authors:** Kirill B Polyanskii, Kseniia A Alekseeva, Pavel V Raspertov, Pavel A Kumandin, Eugeniya V Nikitina, Atash V Gurbanov, Fedor Ivanovich Zubkov

**Affiliations:** 1 Organic Chemistry Department, Faculty of Science, Peoples’ Friendship University of Russia (RUDN University), 6 Miklukho-Maklaya St., Moscow 117198, Russian Federation; 2 Centro de Química Estrutural, Instituto Superior Técnico, Universidade de Lisboa, Av. Rovisco Pais, 1049–001 Lisbon, Portugal; 3 Organic Chemistry Department, Baku State University, Z. Xalilov Str. 23, Az 1148 Baku, Azerbaijan

**Keywords:** CM, cross metathesis, Hoveyda–Grubbs catalyst, olefin metathesis, RCM, ring-closing metathesis, ring-opening cross metathesis, ROCM, ruthenium metathesis catalyst, styrene, 2-vinylbenzylamine

## Abstract

A novel and efficient approach to the synthesis of 2-vinylbenzylamines is reported. This involves obtaining 2-vinylbenzylamine ligands from tetrahydroisoquinoline by alkylation and reduction followed by the Hofmann cleavage. The resultant 2-vinylbenzylamines allowed us to obtain new Hoveyda–Grubbs catalysts, which were thoroughly characterised by NMR, ESIMS, and X-ray crystallography. The utility of this chemistry is further demonstrated by the tests of the novel catalysts (up to 10^−2^ mol %) in different metathesis reactions such as cross metathesis (CM), ring-closing metathesis (RCM) and ring-opening cross metathesis (ROCM).

## Introduction

**Figure 1:**
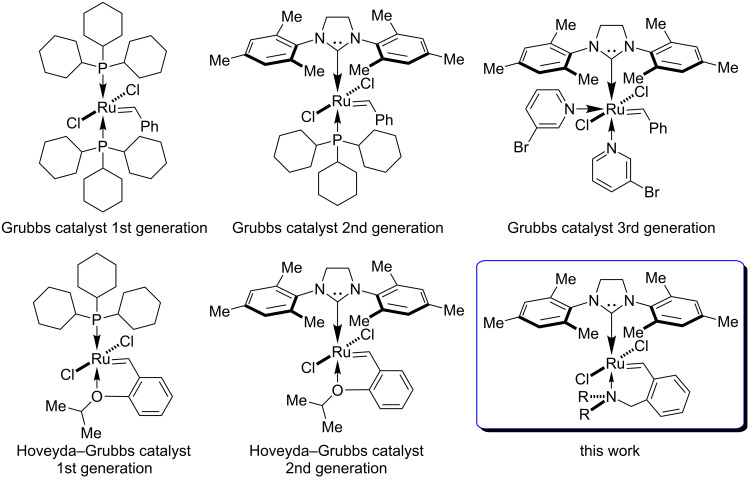
Commercially available ruthenium catalysts for metathesis reactions.

Ruthenium-catalysed olefin metathesis reactions have been playing an important role in various fields of organic synthesis in the past three decades. The significance of this transformation is confirmed by more than 20 reviews devoted to various aspects of metathesis reactions, which were published in last three years (2016–2018). In this paper, we mention only a few of them [1–12], including most popular recent books [13–14]. Obviously, the application of various catalysts is required to achieve the best results in each of the many directions of metathesis reactions such as cross metathesis – CM, ring-opening metathesis – ROM, ring-closing metathesis – RCM, ring-opening metathesis polymerization – ROMP and acyclic diene metathesis – ADMET. This motivates the investigations into the development of new, efficient, stable, and highly selective catalytic systems based on ruthenium complexes. However, in reality, a limited set of commercially available catalysts is used for the whole range of metathesis reactions most probably due to economical reasons. For example, in 2018, Merck offered more than 20 ruthenium metathesis catalysts. The most popular of them are shown in Figure 1.

The framework of these catalysts consists of two main parts that surround the ruthenium centre – “the upper” one is the N-heterocyclic carbene (NHC) ligand and “the lower” one is the 2-alkoxybenzylidene ligand. These determine the principal catalytic properties of the ruthenium complexes. Many ligands were tested as the upper part in various publications, which concluded that NHCs groups, in particular, 1,3-bis(2,4,6-trimethylphenyl)imidazolidine are superior in terms of price/quality ratio. We suppose that further advances should be rather aimed at the lower part of the ruthenium complex [15–17]. This trend is confirmed partly by the Grubbs catalysts with a pyridine group (Figure 1) recently released to the market.

It has been experimentally established that the catalysts with an *O*-isopropyl group in their structure usually show the best combination of stability and activity. However, the oxygen atom having only one site for modification (i.e., the alkyl substituent) reduces the potential diversity of the chemical environment of the catalytic centre [18–21]. In our opinion, replacing the oxygen atom by the nitrogen atom in the lower part of the catalyst would enhance the variability for both steric and electronic effects of the substituents (Figure 1). This would enable a rational selection of the optimal catalysts for specific metathesis reactions.

It should be mentioned here that the idea of replacing the oxygen atom with nitrogen in the Grubbs catalyst is not new but only a limited number of examples are available in the literature [22–38]. Moreover, a small number of patents, which describe the applications of such type of catalysts in ROMP reactions, were published [25–28]. Among all these applications, the use of the catalysts for the polymerization of dicyclopentadiene has the greatest industrial importance [3,9,29–31]. Noteworthy, there are only rare and sporadic publications describing the synthesis and properties of the Grubbs catalysts with an N→Ru coordinate bond in a six-membered ring [32–38].

Thus, the present work opens a series of studies by our group, which will be devoted to the synthesis and reactivity of Hoveyda–Grubbs-type catalysts possessing a six-membered ruthenium-containing ring with the N→Ru, S→Ru, and Se (Te)→Ru coordinate bonds.

## Results and Discussion

### 2-Vinylbenzylamine synthesis

**Figure 2:**
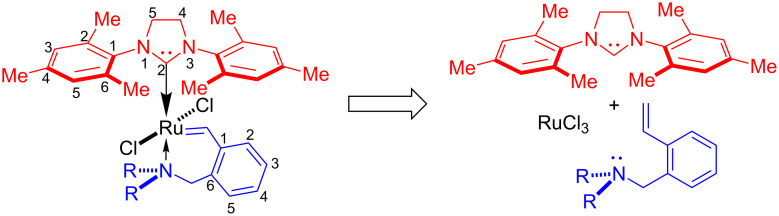
Retrosynthesis of the ruthenium catalysts.

The assembly of the nitrogen-containing ruthenium catalysts required preliminary synthesis of the imidazolium ligand and *o*-vinylbenzylamines (Figure 2). Whereas numerous methods for the preparation of the carbene precursor are known, no satisfactory suitable approach for the synthesis of *ortho*-substituted styrenes was found.

Several methods have been reported for the synthesis of 2-(*N*,*N*-dialkylaminomethyl)styrenes [39–46] relying on different approaches: i) from *o*-vinylbenzyl chloride [43], ii) by the Hofmann cleavage of quaternary tetrahydroisoquinoline salts under the action of silver oxide [39,42] and iii) by a reaction of 2-(2-bromoethyl)benzyl bromide with secondary amines under microwave irradiation followed by the decomposition of the products under the action of potassium *tert*-butoxide [46]. All of the above routes offer some advantages but they all are rather expensive.

**Scheme 1:**
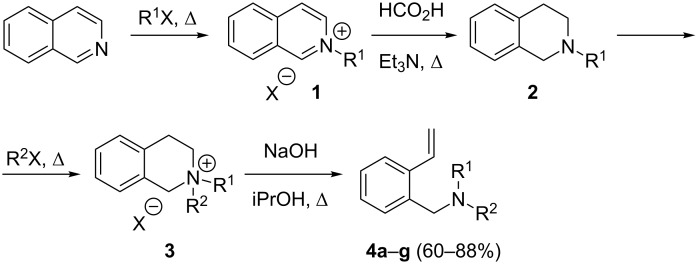
Efficient multigram synthesis of *N*,*N*-dialkyl-2-vinylbenzylamines **4** (R^1^X = Me_2_SO_4_, Et_2_SO_4_ or BnCl, see Experimental part, Supporting Information File 1 and Table 1).

Thus, the initial stage of this work included the development of a preparative scalable method for the synthesis of vinylbenzenes, which provided a wide range of *o*-aminomethylstyrenes from readily accessible reagents in good yields avoiding formation of byproducts. In this process, the synthesis of 2-vinylbenzylamines from isoquinolines (Scheme 1) involved the following steps: alkylation of isoquinolines to afford isoquinolinium salts **1**, its reduction with formic acid giving rise to tetrahydroisoquinolines **2** in a nearly quantitative yield, which upon alkylation gave quaternary salts **3**, finally these salts underwent the Hofmann elimination to form *N*,*N*-dialkylaminomethylstyrenes **4** in yields higher than 60% (Table 1). Our attempts to synthesise highly sterically hindered 2-vinyl-*N*,*N*-diisopropylbenzylamine by an analogous method failed at the stage of the quaternary salt **3**.

**Table 1:** Yields of target 2-vinylbenzylamines **4** after four steps.

entry	compound	R^1^	R^2^	R^2^X	yield, %^а^

1	**4a**	Me	Me	Me_2_SO_4_	87
2	**4b**	Me	Et	Et_2_SO_4_	88
3	**4c**	Me	Bn	BnCl	78
4	**4d**	Me	iPr	iPrI	80
5	**4e**	Et	Et	Et_2_SO_4_	76
6	**4f**	Et	iPr	iPrI	72
7	**4g**	Bn	Bn	BnCl	60

^a^All yields are given after flash column chromatography or vacuum distillation.

**Scheme 2:**
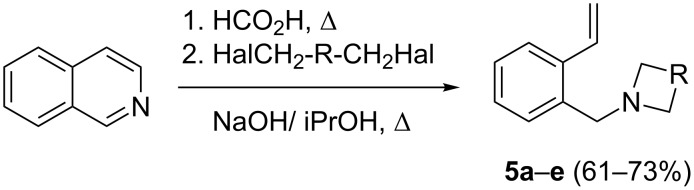
Synthesis of *N*-(2-ethenylbenzyl)heterocycles **5**.

The application of terminal dihalogen derivatives afforded styrenes **5** with a cyclic tertiary amino group from 1,2,3,4-tetrahydroisoquinoline (Scheme 2). In this case, the initial isoquinoline was reduced in the presence of formic acid and then converted into the desired products **5** by a one-pot solvent-free reaction under the action of the corresponding dihalide in alkaline media in a total yield of 61–73% (Table 2).

**Table 2:** Structure and yields of *N*-(2-vinylbenzyl)heterocycles **5**.

entry	compound	structure	initial alkyl halide (HalCH_2_-R-CH_2_Hal)	yield, %^a^

1	**5a**	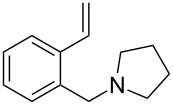	Br(CH_2_)_4_Br	61
2	**5b**	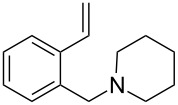	Br(CH_2_)_5_Br	73
3	**5c**	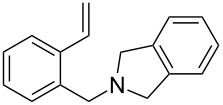	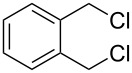	72
4	**5d**	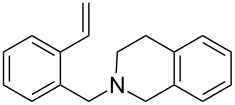	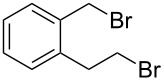	70
5	**5e**	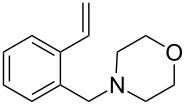	(ClCH_2_CH_2_)_2_O	72

^a^All yields are given after column chromatography or vacuum distillation.

It should be noted that the above-described method was useful for the synthesis of styrenes in quantities of up to 100 g (or even more, if necessary). This scalability was purposefully demonstrated by the multigram synthesis of **5e** (see Experimental part in Supporting Information File 1).

**Scheme 3:**
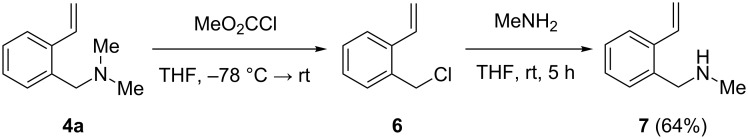
Synthesis of *N*-monoalkyl-2-vinylbenzylamine **7**.

Despite a considerable scope for varying substituents at the nitrogen atom in styrenes **4** and **5**, the developed procedures (Scheme 1 and Scheme 2) do not allow one to obtain benzylamines with a secondary nitrogen atom. The approach outlined in Scheme 3 makes it possible to overcome this problem [44].

**Scheme 4:**
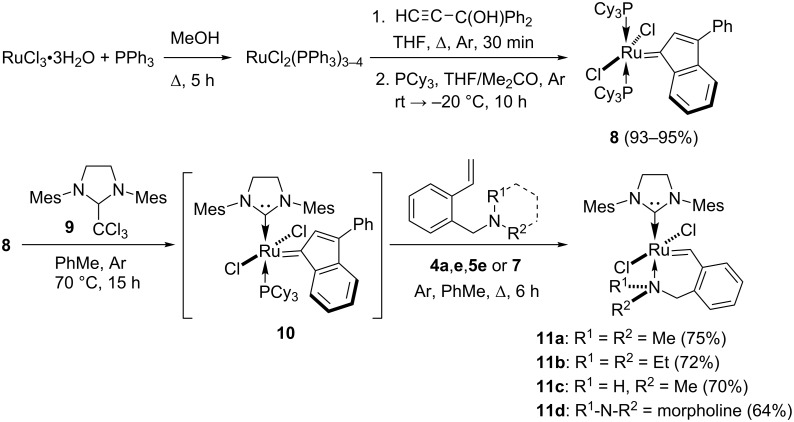
Synthesis of Hoveyda–Grubbs-type catalysts **11**.

Thus, the pathways described in Schemes 1–3 permit one to vary the steric volume of substituents at the nitrogen atom in a wide range enabling synthesis of selective Grubbs catalysts with different catalytic activity. These styrenes were used in the preparation of the target ruthenium complexes shown in Scheme 4. This transformation was carried out using known standard methods including the interaction of the indenylidene derivative **8** with 1,3-dimesityl-2-(trichloromethyl)imidazolidine (**9**) [47–50].

**Scheme 5:**
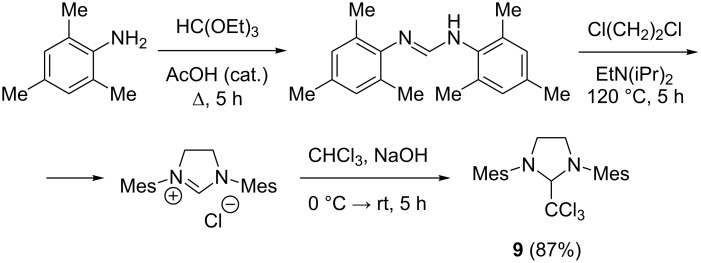
Synthesis of the “chloroform adduct” **9**.

Several approaches have been described earlier for the preparation of the “chloroform adduct” (**9**) [51–55]. Even though these approaches provide good yields they have some drawbacks such as the use of expensive reagents, difficulties in the purification process, and data analysis. Here we propose an alternative reliable procedure for the synthesis of 2-(trichloromethyl)-1,3-bis(2,4,6-trimethylphenyl)imidazolidine (**9**), which was successfully scaled up to 15 g and 30 g (Scheme 5, see Experimental part in Supporting Information File 1). In this process, the key differences are the use of the Hung’s base at the cyclization stage and of granulated alkali in the last step, which provides the target high-purity imidazolidine in 85–87% yield. We stress that there is no need for the isolation and purification of intermediate substances.

The introduction of Ru-indenylidene complex **8** in one-pot reaction with adduct **9** followed by reaction with styrenes **4a**, **4e**, **5e** or **7** gave target Hoveyda–Grubbs-type catalysts **11** in moderate yields (Scheme 4). The products were light-green powders. The synthesised catalysts demonstrated prominent stability in air at room temperature for at least 4 years, which was proved by ^1^H NMR spectra. The simple spectrum recorded in 2014 and at the end of 2018 were identical, they did not show new signals. The catalysts have good solubility in CH_2_Cl_2_, CHCl_3_ and moderate solubility in benzene and toluene. Therefore, they can be used for almost any purpose.

**Figure 3:**
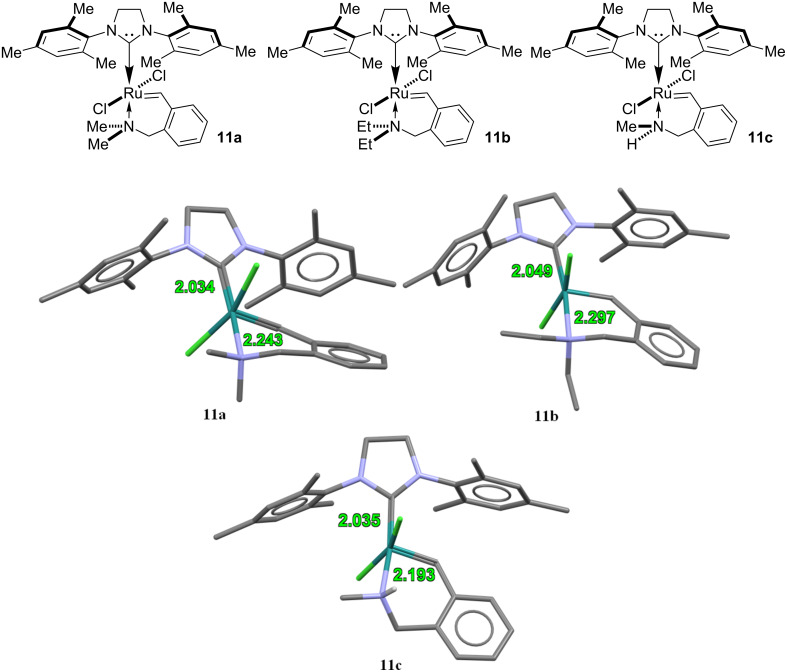
Selected X-ray data for ruthenium complexes **11a**–**c**. All hydrogen atoms were deleted for clarity (except for the hydrogen atom belonging to the NH group in compound **11c**).

Among all of the synthesised catalysts, only three catalysts **11a–c** were obtained as good crystals suitable for X-ray diffraction analysis. Unfortunately, we were not able to obtain single crystals of satisfactory quality for the morpholino-containing catalyst **11d**. Still, the accessible X-ray structural information is sufficient to correlate structure with catalytic activity as presented in the following section (Figure 3). According to the X-ray data, the molecules **11a**–**c** comprise a heterocyclic system with a five-coordinated ruthenium atom having similar general geometrical features. Two chlorine atoms occupy an ordinary *trans*-position relative to the central ruthenium atom. The ruthenium-containing six-membered ring has a slightly distorted envelope conformation with a ca. 51° to 54° deviation of the nitrogen atom from the mean plane of other five atoms. The most important feature of the catalyst structure is the length of the ruthenium–nitrogen bond, which should have the strongest effect on the catalytic activity. With the increase of the steric volume of substituents at the nitrogen atom, the ruthenium–nitrogen coordinate bond is extended, which makes it weaker. That should, obviously, increase the activity of the catalyst towards metathesis reactions. An increase in the N→Ru bond length along the series **11c** (2.193 Å) – **11a** (2.243 Å) – **11b** (2.297 Å) suggests that compound **11b** (with the NEt_2_ substituent) is expected to be the most active as a catalyst.

It should be mentioned that some of catalysts **11** have been synthesized recently in a more complex way in lower yields [32].

**Scheme 6:**
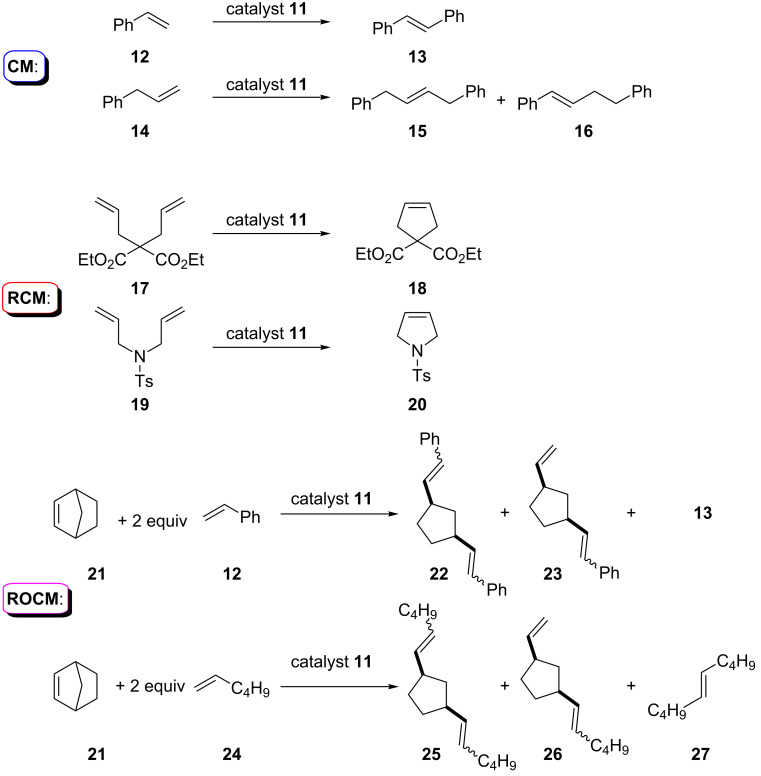
Catalytic activity of compounds **11** in the metathesis reactions.

The third part of this study was devoted to the demonstration of catalytic properties of metallo-complexes **11** in “standard” metathesis reactions (Scheme 6, Table 3). As a model substrate we chose easily available alkenes and dienes, such as i) styrene (**12**) and allylbenzene (**14**) for CM reactions, ii) diethyl diallylmalonate (**17**) and diallyltosylamide (**19**) for RCM reactions, iii) norbornene (**21**) and styrene/hex-1-ene for ROCM metathesis reactions. This selection of model subtests for metathesis is also explained by the possibility to control the course of metathesis and the composition of the reaction mixtures by the GС–MS technique only (GC–MS was carried out using external calibration for CM and RCM reactions). The validity of quantitative GC–MS analysis was confirmed by additional LC–MS and ^1^H NMR analysis of selected reaction mixtures.

**Table 3:** Reaction conditions and yields of the metathesis products.

entry	starting compound	catalyst	catalyst concentration (mol %)	solvent^a^ (conditions)	yield (%), ratio^b^ of the products

1	**12**	**11a**	1	PhMe (Ar)	**13** (traces)^c^
2	**12**	**11b**	1	PhMe (Ar)	**12**/**13** (43%), 51:49
3	**12**	**11a**	1	PhH (Ar)	**13** (traces)^c^
4	**12**	**11b**	1	PhH (Ar)	**12**/**13** (52%), 40:60
5	**12**	**11a**	1	MeCN (Ar)	no product^c^
6	**12**	**11a**	1	THF (Ar)	no product^c^
7	**12**	**11a**	1	CH_2_Cl_2_ (Ar)	**13** (17%)^d^
8	**12**	**11a**	0.1	CH_2_Cl_2_ (Ar)	**13** (traces)^c^
9	**12**	**11b**	1	CH_2_Cl_2_ (Ar)	**13** (49%)^d^
10	**12**	**11b**	0.1	CH_2_Cl_2_ (Ar)	**13** (79%)^d^
11	**12**	**11c**	1	CH_2_Cl_2_ (Ar)	**12/13** (51%), 44:56
12	**12**	**11d**	1	CH_2_Cl_2_ (Ar)	**13** (91%)^d^
13	**12**	**11d**	0.1	CH_2_Cl_2_ (Ar)	**13** (97%)^d^
14	**12**	**11d**	0.01	CH_2_Cl_2_ (Ar)	**12**/**13** (93%), 69:31

15	**12**	**11a**	1	CHCl_3_ (Ar)	**13** (86%)^d^
16	**12**	**11a**	0.1	CHCl_3_ (Ar)	**13** (95%)^d^
17	**12**	**11b**	1	CHCl_3_ (Ar)	**13** (96%)^d^
18	**12**	**11b**	0.1	CHCl_3_ (Ar)	**13** (97%)^d^
19	**12**	**11b**	0.01	CHCl_3_ (Ar)	**13** (81%)^d^
20	**12**	**11b**	0.1	CHCl_3_ (air)	**13** (89%)^d^
21	**12**	**11d**	1	CHCl_3_ (Ar)	**13** (95%)^d^
22	**12**	**11d**	0.1	CHCl_3_ (Ar)	**13** (99%)^d^
23	**12**	**11d**	0.01	CHCl_3_ (Ar)	**12**/**13** (98%), 64:36
24	**14**	**11b**	0.1	CHCl_3_ (Ar)	**15**/**16** (93%), 64:36

25	**17**	**11a**	0.1	CHCl_3_ (air)	**17**/**18** (46%), 44:56
26	**17**	**11b**	0.1	CHCl_3_ (air)	**17**/**18** (58%), 42:58
27	**17**	**11b**	0.01	CHCl_3_ (Ar)	**18** (traces)^e^
28	**17**	**11b**	0.1	CHCl_3_ (Ar)	**17**/**18** (98%), 2:98
29	**17**	**11d**	0.1	CHCl_3_ (air)	**17**/**18** (63%), 42:58
30	**17**	**11d**	0.1	CHCl_3_ (Ar)	**17**/**18** (96%), 4:96
31	**17**	**11d**	0.01	CHCl_3_ (air)	**18** (traces)
32	**19**	**11d**	0.1	CHCl_3_ (Ar)	**19**/**20** (99%), 5:95
33	**19**	**11d**	0.01	CHCl_3_ (Ar)	**19**/**20** (75%), 23/77

34	**21** + 2 equiv **12**	**11a**	0.1	CHCl_3_ (Ar)	**13**/**22**/**23** (71%), ≈77/20/3^f^
35	**21** + 2 equiv **12**	**11d**	0.1	CHCl_3_ (Ar)	**13**/**22**/**23** (78%), ≈81/18/1^f^
36	**21** + 2 equiv **24**	**11a**	0.1	CHCl_3_ (Ar)	**25**/**26**/**27** (40%), ≈3/76/21^f^
37	**21** + 2 equiv **24**	**11b**	0.1	CHCl_3_ (Ar)	**25**/**26**/**27** (52%), ≈2/72/26^f^

^a^All experiments were performed in boiling solvents (10 mL) for 4 h at stirring. ^b^According to GС–MS analysis with external calibration. ^c^Only the starting styrene (**12)** was detected by GС–MS. Term “traces” was used if the product content in the resulting mixture was less than 1%. ^d^Isolated yields are given. ^e^The starting diallyl diethyl malonate **17** was detected by GС–MS. ^f^The compositions of the reaction mixtures were determined by GC–MS without external calibration.

At the beginning of this part, we tested the stability of catalysts **11** in different solvents at different temperatures and conditions (10 mg of **11** in 10 mL of solvent). From this study, we concluded that all catalysts were stable in boiling dry toluene or benzene under argon atmosphere. The catalysts retained their green color for at least a week, which confirms the absence of decomposition. In more polar solvents like dichloromethane and chloroform (bp 40 and 61 °C) boiling under argon also does not cause visible decoloration. On the other hand, the catalysts readily decompose within 0.5–1 h upon boiling in CH_2_Cl_2_ or CHCl_3_ in the presence of air. Boiling in tetrahydrofuran or acetonitrile even under an argon atmosphere leads to a rapid decomposition of the catalysts within 5–10 minutes (the solutions turn black). Therefore, THF and MeCN were excluded from further studies.

In the test reactions, three concentrations of the catalysts (1.0, 0.1 and 0.01 mol %) were applied for the transformation of styrene (**12**) to *trans*-stilbene (**13**) by cross-metathesis reaction (Scheme 6). First experiments (entries 1–4, Table 3) revealed that the nonpolar solvents (PhH and PhMe) are not suitable for the CM reactions. Using of 1 mol % of catalyst **11a** even under an argon atmosphere produced only traces of the target stilbene (**13**). In these conditions, catalyst **11b** was more active than **11a**, but also gave insufficient results. Thereupon, these two solvents were also abandoned in the course of the following investigations.

In this process, temperature also exerts a strong influence on the catalytic activity of metallo complexes **11**. None of catalysts **11** were active at room temperature (19–23 °С) towards the styrene cross metathesis, meaning that all reactions required temperatures higher than 30–35 °С. After all observations, we concluded that dichloromethane is the best solvent for this reaction. At 40 °C, concentrations from 1.0 to 0.1 mol % of the morpholine-based complex **11d** showed the best catalytic activity by providing styrene in 91–97% yields (entries 12 and 13 of Table 3). The somewhat less sterically loaded *N*,*N*-diethyl catalyst **11b** also gave acceptable yields for concentrations from 1.0 to 0.1 mol % (Table 3, entries 9 and 10). Under the same conditions the *N*,*N*-dimethyl catalyst **11a** provided lower yields (Table 3, entries 7 and 8). Similarly, the least sterically loaded *N*-methyl complex **11c** gave a mixture of products **12**/**13** (Table 3, entry 11) in low yields. The metathesis reaction did not proceed completely, even in the presence of 2 mol % of the catalyst. As a result, we did not explore the catalytic activity of **11c** hereinafter. These experimental observations are consistent with the Х-ray data obtained for catalysts **11a**–**c** (Figure 3). In spite of the fact that we do not have the X-ray analysis for catalyst **11d**, it is possible to assume that this complex should exhibit the longest coordination N→Ru bond (at least more than 2.30 Å). Interestingly, lower yields of stilbene from styrene in CH_2_Cl_2_ were obtained in the presence of 1.0 mol % of catalyst **11a**,**c**,**d** as compared with 0.1 mol % of catalyst (cf. Table 3, entries 9/10, 12/13). Obviously, a high concentration of the catalysts accelerates the formation of undesirable products (oligomers and polymers of styrene). At 40 °C in dichloromethane, excellent yields of stilbene were obtained only under the action of metallo complex **11d**. For this reason, we explored elevated temperatures for the metathesis reactions.

The following series of experiments was performed in CHCl_3_ (entries 15–23, Table 3) at 60–61 °C with different concentrations of catalysts. Ruthenium complex **11b** was efficient in the 0.01 mol % concentration under argon atmosphere and in the 0.1 mol % concentration in air. In case of allylbenzene (**14**), the action of catalyst **11b** (0.1 mol %) gave the mixture of isomeric 1,4-diphenylbutenes **15** and **16** in the ratio of 64:36 in 93% yield. Only a small amount of the starting compound **14** underwent polymerisation.

The utility of the catalyst for the RCM reaction was demonstrated by the cyclization of dienes **17** and **19** (Table 3, entries 25–33) in both air and argon atmosphere. The complexes **11a**, **11b** and **11d** in the air atmosphere provided cyclic alkenes **18**, **20** with a strong admixture of initial dienes (Table 3, entries 25, 26, 29). Under argon atmosphere, the same transformations provided good results in the presence of 0.1–0.01 mol % of catalysts **11b**,**d** (Table 3, entries 28, 30–32).

Similarly, in the case of the ROCM reactions (Scheme 6), catalysts **11a**,**b** did not provide high selectivity (Table 3, entries 34–37). Interactions of norbornene (**21**) with a two-fold excess of styrene (**12**) or hex-1-ene (**24**) was accompanied by the CM reaction, which provided products of the ring opening (**22**, **23**, **25**, **26**) and significant amounts of byproducts due to the side cross metathesis (**13** and **27**). Moreover, sparingly soluble high molecular weight products were isolated from all reactions; according to gel permeation chromatography data, these solids are, presumably, norbornene oligomers (see, for example data for entry 35, Supporting Information File 1 and Supporting Information File 2). These four examples demonstrate the principal possibility of application of catalysts **11** in ROCM reactions.

It is known that metathesis reactions carried out in chloroform medium under similar conditions (see Table 3) can give products of the Kharasch radical addition of CHCl_3_ across olefins [56–57]. It is worth to note in the end of this part, that we did not detect formation of chlorine-containing products (molecular peaks with the isotopic distribution characteristic for chlorine were absent in GC–MS spectra).

## Conclusion

The present work reports an efficient method for the synthesis of 2-(*N*,*N*-dialkylaminomethyl)styrenes. The resultant vinyl benzenes are excellent precursors for the synthesis of a new type of Hoveyda–Grubbs catalysts bearing an N→Ru coordinate bond in a six-membered ring. This process does not require the use of complex equipment, extremely expensive or toxic reagents. The structure of the catalysts were elucidated in detail by 2D NMR and X-ray crystallography. The high catalytic activity of the metallo complexes was demonstrated by several examples of cross metathesis (CM), ring-closing (RCM) and ring-opening cross metathesis (ROCM) reactions.

Furthermore, almost all steps of ligands’ and catalysts’ synthesis were accomplished in preparative and multigram scales.

## Supporting Information

File 1 Experimental and analytical data.

File 2 Copies of NMR spectra of synthesised compounds and selected GC–MS data of the metathesis products. Check-cif reports for compounds **11a–c**.
