# Supplementary material for: Hoveyda–Grubbs catalysts with an N→Ru coordinate bond in a six-membered ring. Synthesis of stable, industrially scalable, highly efficient ruthenium metathesis catalysts and 2-vinylbenzylamine ligands as their precursors
Source: Beilstein J Org Chem. 2019 Mar 22;15:769–79. doi: 10.3762/bjoc.15.73 (PMC6444410; doi:10.3762/bjoc.15.73)
Supplement: File 1 — Experimental and analytical data. [file Beilstein_J_Org_Chem-15-769-s001.pdf]

BEILSTEIN JOURNAL OF ORGANIC CHEMISTRY

Supporting Information

for

Hoveyda–Grubbs catalysts with an N→Ru coordinate bond in a six-membered ring. Synthesis of stable, industrially scalable, highly efficient ruthenium metathesis catalysts and 2-vinylbenzylamine ligands as their precursors

Kirill B. Polyanskii, Kseniia A. Alekseeva, Pavel V. Raspertov, Pavel A. Kumandin, Eugeniya V. Nikitina, Atash V. Gurbanov and Fedor I. Zubkov

Beilstein J. Org. Chem. **2019,** *15,* 769–779. doi:10.3762/bjoc.15.73

Experimental and analytical data

License and Terms: This is a supporting information file under the terms of the Creative Commons Attribution License (http://creativecommons.org/licenses/by/4.0). Please note that the reuse, redistribution and reproduction in particular requires that the authors and source are credited. The license is subject to the *Beilstein Journal of Organic Chemistry* terms and conditions: (https://www.beilstein-journals.org/bjoc)

Experimental part

All reagents were purchased from commercial suppliers (Acros Organics and Merck) and used without further purification. All synthesis of ruthenium derivatives require absolute solvents (PhMe, THF, CH_2Cl_2, $ClCH_2CH_2Cl$) and an inert atmosphere (dry Ar). Thin layer chromatography, when necessary, was carried out on aluminum backed silica plates “Sorbfil”. The plates were visualized under UV light (254 nm) or using I_2 vapor. Organic layers were dried over anhydrous $MgSO_4$. Dry (distillated) but not absolute solvents were used in all metathesis reactions.

IR spectra were obtained as KBr pellets or in thin films using an Infralum FT-801 IR-Fourier spectrometer. Mass spectra were taken on a Thermo Focus DSQ II GC-MS spectrometer (electron ionization, 70 eV, ion source temperature 200 °C, gas chromatographic inlet with a Varian FactorFour VF-5ms column) or on an Agilent 1100 series LC/MSD spectrometer with an API-ES/APCI ionization mode. HR-LDI mass spectra of the Grubbs catalysts (**9**) were recorded using a ToF mass spectrometer Bruker autoflex speed, equipped with a solid-state UV-laser of 355 nm and operated in positive reflectron mode. Solutions of the analytes (2 mg/mL) in CH_2Cl_2 were deposited on steel targets (MTP 384 ground steel; Bruker Daltonics Inc., Germany) and air-dried. NMR spectra were run in deuterated solvents (>99.5 atom % D) on Jeol JNM-ECA 600 (600.1 MHz for ^{1}H and 150.9 MHz for ^{13}C) or Bruker Avance 300 or 500 (300 or 500 MHz for ^{1}H and 75.5 or 125.8 MHz for ^{13}C) spectrometers for 3–15 % solutions in $CDCl_3$ or CD_2Cl_2 at 22–30 °C using residual solvent signals (7.26/77.0 ppm for ^{1}H/^{13}C $CDCl_3$ and 5.36/53.4 ppm for CH_2Cl_2) or TMS as an internal standard. The molecular weight of the obtained oligomers was evaluated by gel permeation chromatography (GPC) relative to a polystyrene standard reference material. GPC analysis was performed on a Dionex Ultimate 3000

device with a refractometric detector for 1% solution of the polymer in PhMe. The column Agilent PLgel 5 μm MIXED-C, 7.5 × 300 mm and toluene as a solvent have been used for separation of the components in GPC analysis. Microanalyses were performed for C, H, N on an Eurovector EA 3000 (CHNS) elemental analysis system and were within ±0.4% of theoretical values (the data were obtained at the Center for Collective Use of RUDN University – CCU-PCMR PFUR).

Quantitative analysis of the mixtures of styrene (**12**)/ stilbene (**13**), diallylmalonate (**17**)/ diethyl cyclopent-3-ene-1,1-dicarboxylate (**18**), and diallyltosylamide (**19**)/ 1-tosyl-2,5-dihydro-1*H*-pyrrole (**20**) by GC–MS was carried out using external calibration. Individual response factors for each of compounds were determined using four points calibration curve built by analysis of serially diluted solutions of the analytes in $CHCl_3$ for 0.01–1 mg/mL concentration range. The calibration curves were linear and the established coefficients of determination were not lower than 0.96. It was demonstrated that in the last two cases (**17**–**20**) the obtained GC–MS data can be used without calibration (the error does not exceed +/− 3%).

X-Ray structure determination.

The green crystal of **11a** (CCDC 1851253, $C_{31}H_{39}Cl_2N_3Ru$, M = 625.62) is triclinic, space group *P*-1, at T = 120 K: a = 10.1145(3) Å, b = 10.7226(4) Å, c = 14.6242(5) Å, α = 77.000(1)°, β = 85.492(1)°, γ = 70.337(1)°, V = 1455.28(9) Å^3, Z = 2, μ(Mo Kα) = 0.747 mm^{-1}, d_{calc} = 1.428 g/cm^3, F(000) = 648. 32770 total reflections (9024 unique reflections, R_{int} = 0.0181, R_{sigma} = 0.0160) were measured on a three-circle Bruker APEX-II CCD diffractometer (λ(Mo Kα)-radiation, graphite monochromator, φ and ω scan mode, 2θmax = 61.50°) and corrected for absorption (T_{min} = 0.672; T_{max} =

0.746). The final divergence factors were R_1 = 0.0201 for 8523 independent reflections with I > 2*σ(I)* and wR_2 = 0.0520 (for all data), *S* = 1.041.

The green crystal of **11b** (CCDC 1850381, $C_{33}H_{43}Cl_2N_3Ru$, *M* = 653.67) is monoclinic, space group $P2_1/c$, at *T* = 100 K: *a* = 21.218(3) Å, *b* = 19.218(2) Å, *c* = 15.3386(2) Å, α = 90°, β = 95.830(2)°, γ = 90°, *V* = 6222.4(13) Å^3, *Z* = 8, μ(Mo Kα) = 0.702 mm^{-1}, d_{calc} = 1.396 g/cm^3, F(000) = 2720. 56000 total reflections (12236 unique reflections, R_{int} = 0.0726, R_{sigma} = 0.0569) were measured on a three-circle Bruker APEX-II CCD diffractometer (λ(Mo Kα)-radiation, graphite monochromator, φ and ω scan mode, 2θmax = 52.00°) and corrected for absorption (T_{min} = 0.834; T_{max} = 0.921). The final divergence factors were R_1 0.0612 for 9638 independent reflections with I > 2σ (I) and wR_2 = 0.1547 (for all data), *S* = 1.203. The check-cif file contains an alert B, which is due to a large electron density close to a heavy Ru atom.

The green crystal of **11c** (CCDC 1870071, $C_{30}H_{37}Cl_2N_3Ru$, *M* = 611.59) is monoclinic, space group $P2_1/n$, at *T* = 150 K: *a* = 12.9054(12) Å, *b* = 10.8240(11) Å, *c* = 21.684(2) Å, β = 85.492(1)°, *V* = 2928.1(5) Å^3, *Z* = 4, *μ*(Mo Kα) = 0.741 mm^{-1}, d_{calc} = 1.387 g/cm^3, F(000) = 1264. 23662 total reflections (5778 unique reflections, R_{int} = 0.1183, R_{sigma} = 0.1455) were measured on a three-circle Bruker APEX-II CCD diffractometer (λ(MoK_α)-radiation, graphite monochromator, φ and ω scan mode, 2θmax = 40.54°) and corrected for absorption (T_{min} = 0.885; T_{max} = 0.941). The final divergence factors were R_1 = 0.0581 for 3076 independent reflections with I > 2*σ(I)* and wR_2 = 0.1173 (for all data), *S* = 0.985.

The structures **11a–11c** were determined by direct methods and refined by full-matrix least squares technique on F^2 with anisotropic displacement parameters for non-hydrogen atoms. The hydrogen atoms were placed in calculated positions and refined within riding model with fixed isotropic displacement parameters [U_{iso}(H) = 1.2U_{eq}(C)]. All calculations were carried out using computer programs: for data

collection – APEX2 [1], for data reduction – SAINT V. 8.34A [1], for absorption correction – SADABS [2], for solving structure - SHELXT 2014/6 [3], for refining structure – SHELXL 2014 [4], and for molecular graphics - Olex2 V. 1.2 [5].

Synthesis of 2-(*N*,*N*-dialkyl)aminomethylstyrenes 4. A mixture of isoquinoline 10.0 g (0.078 mol) and the corresponding alkyl halide or dialkyl sulfate (R^1X = BnCl, Me_2SO_4, Et_2SO_4, 0.078 mol) was heated in a two-necked round-bottomed flask at 110 °C under continuous stirring for 1.5 h. After that, formic acid 12.0 mL (14.8 g, 0.326 mol) and triethylamine 12.0 mL (8.6 g, 0.086 mol) were added to the reaction mixture cooled to rt Then stirring and heating at 110 °C were continued for another 4-6 h until gas evolution ceased (a bubble counter was used). After cooling of the reaction mixture to rt, ≈60 mL of 20% NaOH solution (14.0 g, 0.32 mol of NaOH in 56 mL of H_2O) was added. The solution was stirred at rt for 15 min, diluted with Et_2O (50 mL), and stirred for another 15 min. The organic layer was separated and the water phase was extracted with Et_2O (50 mL). The combined organic phases were washed with water (2 × 50 mL), dried over $MgSO_4$ and filtered. The target *N*-alkyl-1,2,3,4-tetrahydroisoquinolines **3** were obtained as viscous, dark-brown oils after evaporation of the solvents under reduced pressure. These crude products were directed to the next alkylation stage without further purification assuming 100% yield.

A mixture of *N*-alkyl-1,2,3,4-tetrahydroisoquinoline (**3**, ≈0.078 mol) and the corresponding alkyl halide or dialkyl sulfate (R^2X, see Table 1, 0.078 mol) was heated at 110 °C and continuous stirring for 1.5 h in a two-necked round-bottomed flask. After cooling of the reaction mixture to rt, iPrOH (40 mL) and NaOH 6.40 g (0.16 mol) were added, then stirring and heating at 85 °C were continued for another hour. The solvent was evaporated under reduced pressure, then a mixture of H_2O (60 mL) and Et_2O (80 mL) was added, and the reaction mixture was stirred for additional 30 min at rt. The organic layer was separated and the water phase was

extracted with Et_2O (80 mL). The combined organic phases were filtered through silica gel (4 × 3 cm), the sorbent was washed with Et_2O (2 × 50 mL). After evaporation of the solvent under reduced pressure, crude 2-(*N,N*-dialkyl)aminomethylstyrenes (**4**) were obtained as viscous brown or dark-yellow oils. The analytical samples for analysis and catalysts synthesis were obtained after purification of the crude products by silica gel column chromatography using hexane as an eluent, which gives target styrenes **4** as slightly yellow or colorless, viscous oils, in 60–88 % yields after four steps, see Table 1.

1-(2-Ethenylphenyl)-*N*,*N*-dimethylmethanamine (4a): colorless oil. Yield 10.92 g (0.068 mol, 87%). ^{1}H NMR (600 MHz, $CDCl_3$) δ, ppm: 7.55 (br.d, *J* 7.6 Hz, 1H, H-3-Ph), 7.27-7.21 (m, 3H, H-Ar), 7.17 (dd, *J* 7.6 and *J* 17.7 Hz, 1H, C*H*=CH_2), 5.68 (dd, *J* 1.5 and *J* 17.7 Hz, 1H, H-2-*trans*), 5.30 (dd, *J* 1.5 and *J* 11.1 Hz, 1H, H-2-*cis*), 3.43 (s, 2H, CH_2N), 2.23 (s, 6H, 2 × Me_2N). ^{13}C NMR (150 MHz, $CDCl_3$) δ, ppm: 137.7, 136.1, 134.6, 130.6, 127.5, 127.4, 125.6, 115.3, 62.1, 45.6 (2C). IR ν_{max}/cm^{-1} (KBr): 1630 (C=C). HR-MS: *m/z* $[M + H]^+$ calcd. for $C_{11}H_{16}N$: 162.1277; found: 162.1273. Anal. calcd. for $C_{11}H_{15}N$: C 81.94, H 9.38, N 8.69; found C 81.90, H 9.33, N 8.61.

***N*-(2-Ethenylbenzyl)-*N*-methylethanamine (4b):** light-yellow oil. Yield 12.01 g (0.069 mol, 88%). ^{1}H NMR (600 MHz, $CDCl_3$) δ, ppm: 7.28-7.17 (m, 5H, H-3-6-Ph, C*H*=CH_2), 5.65 (dd, *J* 1.5 and *J* 17.7 Hz, 1H, H-2-*trans*), 5.28 (dd, *J* 1.5 and *J* 11.1 Hz, 1H, H-2-*cis*), 3.48 (s, 2H, CH_2N), 2.46 (q, *J* 7.1 Hz, 2H, CH_2Me), 2.16 (s, 3H, *Me*N), 1.06 (t, *J* 7.1 Hz, 3H, *Me*CH_2). ^{13}C NMR (150 MHz, $CDCl_3$) δ, ppm: 137.7, 136.4, 134.8, 130.5, 127.4, 127.3, 125.6, 115.1, 59.7, 51.7, 41.6, 12.5. IR ν_{max}/cm^{-1} (KBr): 1640 (C=C). HR-MS: *m/z* $[M + H]^+$ calcd. for $C_{12}H_{18}N$: 176.1434; found: 176.1432. Anal. calcd. for $C_{12}H_{17}N$: C 82.23, H 9.78, N 7.99; found C 81.93, H 10.08, N 8.27.

N-Benzyl-1-(2-ethenylphenyl)-N-methylmethanamine (4c): colorless oil. Yield 14.4 g (0.061 mol, 78%). ^{1}H NMR (600 MHz, $CDCl_3$) δ, ppm: 7.58 (dd, J 1.5 and J 7.1 Hz, 1H, H-3-Ph), 7.38-7.35 (m, 5H, H-Ar), 7.29-7.25 (m, 3H, H-Ar), 7.22 (dd, J 11.1 and J 17.7 Hz, 1H, C*H*=CH_2), 5.69 (dd, J 1.5 and J 17.7 Hz, 1H, H-2-*trans*), 5.31 (dd, J 1.5 and J 11.1 Hz, 1H, H-2-*cis*), 3.59 (s, 2H, CH_2NCH_2), 3.55 (s, 2H, CH_2NCH_2), 2.18 (s, 3H, *Me*N). ^{13}C NMR (150 MHz, $CDCl_3$) δ, ppm: 139.5, 137.9, 136.4, 135.0, 130.6, 129.1 (2C), 128.3 (2C), 127.5 (2C), 127.1, 125.7, 115.0, 62.2, 60.1, 42.2. IR v_{max}/cm^{-1} (KBr): 1625 (C=C). HR-MS: *m/z* $[M + H]^+$ calcd. for $C_{17}H_{20}N$: 238.1596; found: 238.1589. Anal. calcd. for $C_{17}H_{19}N$: C 86.03, H 8.07, N 5.90; found C 86.35, H 8.12, N 5.63.

N-(2-Ethenylbenzyl)-N-methylpropan-2-amine (4d): light-yellow oil. Yield 11.79 g (0.062 mol, 80%). ^{1}H NMR (600 MHz, $CDCl_3$) δ, ppm: 7.54 (dd, J 1.7 and J 7.1 Hz, 1H, H-3-Ph), 7.33-7.31 (m, 1H, H-6-Ph), 7.28-7.22 (m, 3H, H-4,5-Ph, C*H*=CH_2), 5.67 (dd, J 1.5 and J 17.7 Hz, 1H, H-2-*trans*), 5.30 (dd, J 1.5 and J 10.6 Hz, 1H, H-2-*cis*), 3.57 (s, 2H, CH_2N), 2.93 (heptet, J 6.6 Hz, 1H, C*H*Me_2), 2.14 (s, 3H, N*Me*), 1.10 (t, J 6.6 Hz, 6H, Me_2CH). ^{13}C NMR (150 MHz, $CDCl_3$) δ, ppm: 137.7, 137.0, 135.0, 130.2, 127.5, 127.1, 125.7, 115.1, 55.6, 46.8, (2C), 11.7 (2C). IR v_{max}/cm^{-1} (KBr): 1626 (C=C). HR-MS: *m/z* $[M + H]^+$ calcd. for $C_{13}H_{20}N$: 190.1596; found: 190.1587. Anal. calcd. for $C_{13}H_{19}N$: C 82.48, H 10.12, N 7.40; found C 82.32, H 10.20, N 7.29.

N-(2-Ethenylbenzyl)-N-ethylethanamine (4e): light-yellow oil. Yield 11.2 g (0.059 mol, 76%). ^{1}H NMR (600 MHz, $CDCl_3$) δ, ppm: 7.54-7.52 (m, 1H, H-3-Ph), 7.38-7.37 (m, 1H, H-6-Ph), 7.28-7.22 (m, 3H, H-4,5-Ph, C*H*=CH_2), 5.65 (dd, J 1.5 and J 17.7 Hz, 1H, H-2-*trans*), 5.39 (dd, J 1.5 and J 11.1 Hz, 1H, H-2-*cis*), 3.61 (s, 2H, CH_2N), 2.55 (q, J 7.1 Hz, 4H, 2 × CH_2Me), 1.06 (t, J 7.1 Hz, 6H, 2 × *Me*CH_2). ^{13}C NMR (150 MHz, $CDCl_3$) δ, ppm: 137.7, 137.0, 135.0, 130.2, 127.5, 127.1, 125.7, 115.1, 55.6, 46.8 (2C), 11.7 (2C). IR v_{max}/cm^{-1} (KBr): 1626 (C=C). HR-MS: *m/z* $[M + H]^+$ calcd. for

$C_{13}H_{20}N$: 190.1596; found: 190.1589. Anal. calcd. for $C_{13}H_{19}N$: C 82.48, H 10.12, N 7.40; found C 82.63, H 10.40, N 7.69.

***N*-(2-Ethenylbenzyl)-*N*-ethylpropan-2-amine (4f):** light-yellow oil. Yield 11.4 g (0.056 mol, 72%). ^{1}H NMR (600 MHz, $CDCl_3$) δ, ppm: 7.48-7.47 (m, 1H, H-3-Ph), 7.43-7.42 (m, 1H, H-6-Ph), 7.25 (dd, *J* 11.1 and *J* 17.7 Hz, 1H, C*H*=CH_2), 7.28-7.20 (m, 2H, H-4,5-Ph), 5.61 (dd, *J* 1.5 and *J* 17.7 Hz, 1H, H-2-*trans*), 5.25 (dd, *J* 1.5 and *J* 11.1 Hz, 1H, H-2-*cis*), 3.60 (s, 2H, PhCH_2N), 2.94 (heptet, *J* 6.6 Hz, 1H, C*H*Me_2), 2.46 (q, *J* 7.6 Hz, 2H, NC$H_2$$CH_3$), 1.02 (d, *J* 6.6 Hz, 6H, CHMe_2), 1.00 (t, *J* 7.6 Hz, 3H, CH_2*Me*). ^{13}C NMR (150 MHz, $CDCl_3$) δ, ppm: 138.1, 137.5, 135.1, 129.7, 127.4, 126.7, 125.6, 114.9, 51.5, 49.46, 43.24, 18.0 (2C), 14.0. IR v_{max}/cm^{-1} (KBr): 1643 (C=C). HR-MS: *m/z* $[M + H]^+$ calcd. for $C_{14}H_{22}N$: 204.1752; found: 204.1755. Anal. calcd. for $C_{14}H_{21}N$: C 82.70, H 10.41, N 6.89; found C 82.81, H 10.68, N 7.04.

***N*,*N*-Dibenzyl-1-(2-ethenylphenyl)methanamine (4g):** light-yellow oil. Yield 14.65 g (0.047 mol, 60%). ^{1}H NMR (600 MHz, $CDCl_3$) δ, ppm: 7.50-7.49 (m, 1H, H-3-Ph), 7.44-7.43 (m, 1H, H-6-Ph), 7.37 (br.d, *J* 7.1 Hz, 4H, 2 × H-2,6-Bn), 7.32 (br.t, *J* 7.1 Hz, 4H, 2 × H-3,5-Bn), 7.25-7.21 (m, 4H, H-4,5-Ph, 2 × H-4-Bn), 7.00 (dd, *J* 11.1 and *J* 17.2 Hz, 1H, C*H*=CH_2), 5.60 (dd, *J* 1.1 and *J* 17.2 Hz, 1H, H-2-*trans*), 5.20 (dd, *J* 1.1 and *J* 11.1 Hz, 1H, H-2-*cis*), 3.59 (s, 2H, NCH_2), 3.52 (s, 4H, 2 × NCH_2Ph). ^{13}C NMR (150 MHz, $CDCl_3$) δ, ppm: 139.6 (2C), 137.8, 136.5, 135.3, 130.4, 129.2 (4C), 128.3 (4C), 127.5, 127.4, 127.0 (2C), 125.8, 114.7, 58.3 (2C), 56.3. IR v_{max}/cm^{-1} (KBr): 1626 (C=C). HR-MS: *m/z* $[M + H]^+$ calcd. for $C_{23}H_{24}N$: 314.1909; found: 314.1914. Anal. calcd. for $C_{23}H_{23}N$: C 88.13, H 7.40, N 4.47; found C 87.84, H 7.61, N 4.15.

Synthesis of *N*-(2-ethenylbenzyl)heterocycles (5a, 5b, 5d, 5e). A mixture of 1,2,3,4-tetrahydroisoquinoline (10.0 g, 0.075 mol), the corresponding dihalide (0.078 mol, see Table 2), iPrOH (100 mL) and NaOH 6.80 g (0.168 mol) was heated at 90

°C and continuous stirring for 2 h in a two-necked round-bottomed flask. After the solvent evaporation under reduced pressure, Et_2O (60 mL) was added to the reaction mixture and it was stirred for another 30 min at rt Precipitate was filtered of and washed with Et_2O (2 × 30 mL), filtrate was evaporated under reduced pressure, the residue (brown oil) was purified by silica gel column chromatography using an hexane/ethyl acetate (10:1) mixture as an eluent to give target 2-aminomethylstyrenes (**5**) as viscous slight-yellow oils in total yields 57–72 % (see Table 2).

1-(2-Ethenylbenzyl)pyrrolidine (5a): yellow oil. Yield 8.5 g (0.045 mol, 61%). ^{1}H NMR (600 MHz, $CDCl_3$) δ, ppm: 7.51 (dd, *J* 2.0 and *J* 7.1 Hz, 1H, H-3-Ph), 7.34 (dd, *J* 1.8 and *J* 7.5 Hz, 1H, H-6-Ph), 7.26-7.21 (m, 2H, H-4,5-Ph), 7.17 (dd, *J* 10.6 and *J* 17.1 Hz, 1H, C*H*=CH_2), 5.65 (dd, *J* 1.0 and *J* 17.1 Hz, 1H, H-2-*trans*), 5.30 (dd, *J* 1.0 and *J* 10.6 Hz, 1H, H-2-*cis*), 3.69 (s, 2H, CH_2N), 2.56-2.54 (m, 4H, H-2,5-Cyclo), 1.79-1.77 (m, 4H, H-3,4-Cyclo). ^{13}C NMR (150 MHz, $CDCl_3$) δ, ppm: 137.4, 136.5, 134.9, 130.0, 127.6, 127.3, 125.6, 115.4, 58.0, 54.3 (2C), 23.6 (2C). IR v_{max}/cm^{-1} (KBr): 1626 (C=C). HR-MS: *m/z* $[M + H]^+$ calcd. for $C_{13}H_{18}N$: 188.1434; found: 188.1428. Anal. calcd. for $C_{13}H_{17}N$: C 83.37, H 9.15, N 7.48; found C 83.04, H 8.88, N 7.15.

1-(2-Ethenylbenzyl)piperidine (5b): yellow oil. Yield 11.0 g (0.0548 mol, 73% новое). ^{1}H NMR (600 MHz, $CDCl_3$) δ, ppm: 7.51 (dd, *J* 1.5 and *J* 7.6 Hz, 1H, H-3-Ph), 7.28 (dd, *J* 1.5 and *J* 7.2 Hz, 1H, H-6-Ph), 7.24-7.18 (m, 3H, H-4,5-Ph, C*H*=CH_2), 5.63 (dd, *J* 1.5 and *J* 17.7 Hz, 1H, H-2-*trans*), 5.26 (dd, *J* 1.5 and *J* 11.1 Hz, 1H, H-2-*cis*), 3.16 (s, 2H, CH_2NPh), 2.38-2.36 (m, 4H, H-2,6-Pip), 1.56-1.52 (m, 4H, H-3,5-Pip), 1.43-1.41 (m, 2H, H-4-Pip). ^{13}C NMR (150 MHz, $CDCl_3$) δ, ppm: 137.8, 135.9, 135.0, 130.0, 127.2, 127.1, 125.6, 115.0, 61.27, 54.6 (2C), 26.0 (2C), 24.5. IR v_{max}/cm^{-1} (KBr): 1624 (C=C). HR-MS: *m/z* $[M + H]^+$ calcd. for $C_{14}H_{20}N$:

202.1590; found: 202.1593. Anal. calcd. for $C_{14}H_{19}N$: C 83.53, H 9.51, N 6.96; found C 83.33, H 9.28, N 6.77.

2-(2-Ethenylbenzyl)-1,2,3,4-tetrahydroisoquinoline (5d): yellow oil. Yield 13.07 g (0.052 mol, 70%). ^{1}H NMR (600 MHz, $CDCl_3$) δ, ppm: 7.54 (dd, *J* 1.5 and *J* 7.6 Hz, 1H, H-3-Ph(vinyl)), 7.36 (dd, *J* 1.5 and *J* 7.1 Hz, 1H, H-8-Ph), 7.28-7.22 (m, 3H, H-Ar), 7.12-7.08 (m, 3H, 2H-Ar, C*H*=CH_2), 6.99-6.97 (m, 1H, H-6-Ph(vinyl)), 5.65 (dd, *J* 1.5 and *J* 17.6 Hz, 1H, H-2-*trans*), 5.26 (dd, *J* 1.5 and *J* 11.1 Hz, 1H, H-2-*cis*), 3.69 (s, 2H, CH_2N *exo*-cycl), 3.64 (s, 2H, CH_2N-*endo*-cycl (H-1)), 2.87 (br.t, *J* 6.0 Hz, 2H, H-3), 2.73 (t, *J* 6.0 Hz, 2H, H-4). ^{13}C NMR (150 MHz, $CDCl_3$) δ, ppm: 138.0, 135.5, 135.2, 134.9, 134.7, 130.4, 128.8, 127.54, 127.51, 126.7, 126.1, 125.8, 125.6, 115.5, 60.5, 56.3, 50.6, 29.4. IR $ν_{max}/cm^{-1}$ (KBr): 1625 (C=C). HR-MS: *m/z* $[M + H]^+$ calcd. for $C_{18}H_{20}N$: 250.1590; found: 250.1586. Anal. calcd. for $C_{18}H_{19}N$: C 86.70, H 7.68, N 5.62; found C 86.47, H 7.44, N 5.77.

4-(2-Ethenylbenzyl)morpholine (5e): yellow oil. Yield 10.96 g (0.054 mol, 72%). ^{1}H NMR (600 MHz, $CDCl_3$) δ, ppm: 7.53 (dd, *J* 1.5 and *J* 7.6 Hz, 1H, H-3-Ph), 7.26-7.18 (m, 4H, H-4-6-Ph and C*H*=CH_2), 5.65 (dd, *J* 1.5 and *J* 17.7 Hz, 1H, H-2-*trans*), 5.29 (dd, *J* 1.5 and *J* 11.1 Hz, 1H, H-2-*cis*), 3.67 (t, *J* 4.8 Hz, 4H, CH_2OCH_2), 3.50 (s, 2H, ArCH_2N), 2.43 (br.t, 4H, CH_2NCH_2). ^{13}C NMR (150 MHz, $CDCl_3$) δ, ppm: 138.0, 135.0, 134.9, 130.6, 127.7, 127.5, 125.8, 115.4, 67.2 (2C), 61.2, 53.7 (2C). IR $ν_{max}/cm^{-1}$ (KBr): 1626 (C=C). HR-MS: *m/z* $[M + H]^+$ calcd. for $C_{13}H_{18}NO$: 204.1383; found: 204.1387. Anal. calcd. for $C_{13}H_{17}NO$: C 76.81, H 8.43, N 6.89; found C 76.74, H 8.29, N 6.61.

An example of multigram synthesis of 5e. 1,2,3,4-Tetrahydroisoquinoline (100 g, 0.76 mol), bis(2-chloroethyl)ether (98 mL, 120 g, 0.84 mol) and NaOH (68 g, 1.68 mol) were placed in a 1 L round bottom flask equipped with a magnetic stirrer. The reaction mixture was heated at reflux (113–115 °C) for 1 h under continuous stirring.

Then the reaction mixture was cooled to rt and methyl *tert*-butyl ether (MTBE, 600 mL) was added to the solution, which was stirred for another 30 min. The precipitate was filtered off and washed with MTBE (2 × 300 mL). The filtrate was evaporated under reduced pressure, the residual brown oil, was purified by vacuum distillation (b.p. 95–97 °C / 0.01 mbar, 150–155 °C in a bath) to give 110.1 g (71.5% from starting isoquinoline) of 4-(2-ethenylbenzyl)morpholine (**5e**), with 99%+ purity according to GC–MS analysis.

Synthesis of 2-(2-ethenylbenzyl)-2,3-dihydro-1*H*-isoindole (5c). A mixture of 1,2,3,4-tetrahydroisoquinoline (10.0 g, 0.078 mol), 1,2-bis(chloromethyl)benzene (9.7 mL, 0.078 mol), acetonitrile (100 mL), anhydrous NaI (11.7 g, 0.078 mol) and K_2CO_3 (7.72 g, 0.078 mol) was stirred at 85–90 °C for 8 h. The precipitate was filtered off and washed with chloroform (2 × 30 mL), the filtrate was concentrated under reduced pressure. BuOH (80 mL) and KOH (5.0 g, 0.089 mol) were added to the residue. This mixture was heated at 110 °C under continuous stirring for 1.5 h. Butanol was evaporated under reduced pressure, then Et_2O (60 mL) was added to the reaction mixture and it was stirred for another 30 min at rt. The precipitate was filtered off and washed with Et_2O (2 × 30 mL), the filtrate was evaporated under reduced pressure, the residual brown oil was purified by silica gel column chromatography using an hexanes/ethyl acetate (10:1) mixture as eluent to give target 2-aminomethylstyrene **5c** as viscous slight-yellow oil. Yield 13.20 g (0.056 mol, 72%). ^{1}H NMR (600 MHz, $CDCl_3$) δ, ppm: 7.56 (dd, *J* 1.5 and *J* 7.1 Hz, 1H, H-Ar), 7.38 (dd, *J* 1.5 and *J* 7.1 Hz, 1H, H-Ar), 7.30-7.27 (m, 2H, H-Ar), 7.24 (dd, *J* 11.0 and *J* 17.1 Hz, 1H, C*H*=CH_2), 7.18 (br. s, 4H, H-Ar-Isoindole), 5.69 (dd, *J* 1.5 and *J* 17.2 Hz, 1H, H-2-*trans*), 5.30 (dd, *J* 1.5 and *J* 11.1 Hz, 1H, H-2-*cis*), 3.96 (s, 2H, CH_2N *exo*-cycl), 3.94 (s, 4H, H-1,3-*endo*-cycl). ^{13}C NMR (150 MHz, $CDCl_3$) δ, ppm: 140.2, 137.5, 136.0, 134.6, 129.9, 127.9, 127.6, 127.5, 126.6, 125.7 (2C), 122.3 (2C), 115.5, 58.9 (2C), 57.8. IR

v_{max}/cm^{-1} (KBr): 1626 (C=C). MS-HR (ESI+): *m/z* $[M + H]^+$ calcd. for $C_{17}H_{18}N$: 236.1434; found: 236.1431. Anal. calcd. for $C_{17}H_{17}N$: C 86.77, H 7.28, N 5.95; found C 86.37, H 7.43, N 5.78.

Synthesis of 2-(*N*-methylaminomethyl)styrene (7). The method, adapted from a procedure outlined by Molander and Pack [6], was applied. Under vigorous stirring methyl chloroformate (8.3 mL, 0.11 mol) was added dropwise to a mixture of styrene **4a** (16.1 g, 0.10 mol), THF (100 mL), and K_2CO_3 (21.0 g, 0.15 mmol) cooled to −78 °C in a round bottom flask (500 mL) attached to a nitrogen line. The thick, white paste was allowed to warm to rt and stirred at this temperature for ca. 10 min. The slurry was cooled to 0 °C and diluted with a mixture of water (100 mL) and Et_2O (150 mL). The organic layer was separated and the aqueous layer was extracted with Et_2O (2 × 100 mL). The combined organic layers were cooled to 0 °C, washed with ice water (2 × 100 mL), dried over K_2CO_3 and filtered. The solvents were evaporated under reduced pressure to yield 14.5 g (95 mmol, 95%) of compound **6** as a pale yellow oil.

Potash (4.15 g, 0.03 mol) was added to a solution of 2-chloromethylstyrene (**6**, 4.65 g, 0.03 mol) in abs. THF (100 mL). Then, a 2 M solution of methylamine in abs. THF 30 mL (≈0.06 mol) was added dropwise at 0–5 °C. The reaction mixture was stirred at rt for 1 h and then for another 3 h at 60 °C in a tightly closing flask, cooled to rt, treated with brine (100 mL) and extracted with CH_2Cl_2 (3 × 50 mL). The combined organic phases were dried over Na_2SO_4, filtered, evaporated, and the residue was purified by silica gel column chromatography (ethyl acetate/hexane, 1:2 as an eluent) to give 2.82 g (64%) of styrene **7** as colorless oil. ^{1}H NMR (600 MHz, $CDCl_3$) δ, ppm: 7.55-7.52 (m, *J* 7.6 Hz, 1H, H-3-Ph), 7.33-7.22 (m, 3H, H-3,5,6-Ar), 7.09 (dd, *J* 11.0 and *J* 17.4 Hz, 1H, C*H*=CH_2), 5.70 (dd, *J* 1.6 and *J* 17.4 Hz, 1H, H-2-*trans*), 5.30 (dd, *J* 1.6 and *J* 11.0 Hz, 1H, H-2-*cis*), 3.69 (s, 2H, CH_2N), 2.30 (s, 3H, *Me*N). ^{13}C NMR

(150 MHz, $CDCl_3$) δ, ppm: 137.6, 136.8, 134.7, 129.7, 128.0, 127.6, 125.7, 116.1, 53.1, 36.3. IR v_{max}/cm^{-1} (KBr): 1630 (C=C). HR-MS: *m/z* $[M + H]^+$ calcd. for $C_{10}H_{14}N$: 148.2210; found: 148.2216. Anal. calcd. for $C_{10}H_{13}N$: C 81.58, H 8.90, N 9.51; found C 81.63, H 9.16, N 9.69

Synthesis of the Wilkinson complex ($RuCl_2(PPh_3)_{3-4}$). A mixture of ruthenium(III) chloride hydrate (8.05 g ≈0.03 mol) and triphenylphosphane (39.3 g, 0.15 mol) was placed in a Schlenk flask, which was purged with argon. Methanol (300 mL) was added into the flask and the resulting mixture was heated at reflux for 4 h under continuous stirring. After cooling of the reaction mixture to rt, the precipitate was filtered off, washed with Et_2O (3 × 100 mL) and air-dried to give 30.93 g of the Wilkinson complex as black powder.

Dichloro(3-phenyl-1*H*-inden-1-ylidene)bis(tricyclohexylphosphane)ruthenate (8). A solution of **$RuCl_2(PPh_3)_{3-4}$** (40.0 g, ≈41.7 mmol) and 1,1-diphenyl-2-propyn-1-ol (10.4 g, 50.1 mmol) in abs. THF (300 mL) was placed into a Schlenk flask under an argon atmosphere. A 5.4 M solution of HCl in dioxane (6.2 mL, 33.4 mmol) was added and then the mixture was heated at reflux for 30 min under continuous stirring and an argon atmosphere. After cooling to rt, around 50% of mixture volume was evaporated under reduced pressure. Acetone (280 mL) and tricyclohexylphosphane 25.7 g (91.9 mmol) were added to the residue and the resulting suspension was stirred until thickening (≈0.5 h). After holding at –20 °C for 10 h the precipitate was filtered off and washed sequentially by methanol (2 × 80 mL), acetone (2 × 100 mL) and hexane (100 mL), then dried under vacuum at rt to give indenylidene–ruthenium complex **8** in 94% yield (36.3 g, 39.2 mmol) as red-brown powder.

Complex **8** can be obtained by an analogical procedure in 90% yield (34.8 g) using absolute dioxane as a solvent instead absolute THF.

2-(Trichloromethyl)-1,3-bis(2,4,6-trimethylphenyl)imidazolidine (**9**) [7]. A mixture of 2,4,6-trimethylaniline (11.9 g, 0.088 mol), triethyl orthoformate (7.1 g, 0.048 mol) and acetic acid (0.12 mL) was heated at reflux (≈120 °C) for 5 h. The mixture was evaporated until dry under reduced pressure. A mixture of dichloroethane (25 mL) and diisopropylethylamine (8.9 mL, 0.05 mol) was added to the precipitate and after that the suspension was stirred at 120 °C for 5 h in a hermetically sealed Schlenk flask. The formed solution was evaporated to dryness under reduced pressure. The obtained precipitate was washed with acetone (85 mL) and dried under vacuum at rt. The residue was dissolved in chloroform (300 mL) at 0 °C, and then granulated NaOH (68.0 g, 1.70 mol, with a pellet diameter of ≈0.5 mm) was added in one portion to the solution at the same temperature under vigorous stirring. Stirring was continued until the temperature reaches 18 °C (≈40 min), then the reaction was stirred at this temperature for another 4 h (until the end of the heat release process). The precipitate (NaOH) was filtered off, washed with chloroform (2 × 40 mL) and hexane (2 × 40 mL). After the last operation, sodium hydroxide can be used one more time in the same step. The filtrate was evaporated under reduced pressure. The solid residue was treated with hexane (70 mL) under ultrasonic irradiation for 10 min and then held at 4 °C for 24 h. The precipitate was filtered off, suspended with methanol (60 mL), treated by ultrasonic irradiation for 2 min and filtered off. The last operation was repeated three more times, after that the white powder was dried under vacuum at rt to give 14.3 g of the title compound **9** (87% relative to the starting 2,4,6-trimethylaniline) with >95% purity (by ^{1}H NMR). ^{1}H NMR (400 MHz, $CDCl_3$, 24 °C) δ, ppm: 6.90 (s, 2H, H-Ph), 6.86 (s, 2H, H-Ph), 5.60 (s, 1H, H-2), 3.94 (dd, *J* 5.7 and *J* 8.3 Hz, 2H, H-3A and H-4A), 2.28 (s, 6H, Me-Ph), 2.48 (s, 6H, Me-Ph), 2.50 (s, 6H, Me-Ph), 3.34 (dd, *J* 5.7 and *J* 8.3 Hz, 2H, H-3B and H-4B).

The described above technique was successfully scaled for preparation of 30 g of imidazolidine **9** (yield 85%).

Synthesis of the Hoveyda–Grubbs catalysts (11a–d). Absolute toluene (50 mL), complex **8** (4.40 g, 4.75 mmol), and 1,3-bis(2,4,6-trimethylphenyl)-2-trichloromethylimidazolidine (**9**) 2.41 g (5.70 mmol) were placed into a Schlenk flask (100 mL). The mixture was heated under argon at 70 °C for 15 h, then was cooled to r.t and styrene **4a**, **4e**, **5e** or **7** (7.14 mmol) was added in an argon stream. The mixture was heated at reflux under an inert atmosphere for 6 h. Toluene was evaporated under reduced pressure and the residue was suspended in hexane (30 mL). The obtained mixture was kept at −20 °C for 10 h. The precipitate was filtered off and washed with hexane (3 × 12 mL) and then with methanol (2 × 12 mL) to give after drying under vacuum a light green powder of pure catalyst (**11**), which are stable at rt in air at least for 4 years (see the Discussion). System for TLC is hexane : EtOAc, 4:1. R_f 0.3–0.4 (Sorbfil TLC plates were used). The spectroscopic data and physical properties of complexes **11a**,**b** correspond to the published earlier [8].

[1,3-*Bis*(2,4,6-trimethylphenyl)imidazolidin-2-ylidene](dichloro){2-[(dimethylamino)methyl]benzylidene}ruthenium (11a). Light-green powder, (2.22 g, 3.56 mmol, 75%), m.p. 202.4–206 °C (decomp.), dark-green prisms after slow crystallization from an heptane-CH_2Cl_2 mixture. ^{1}H NMR (300.1 MHz, CD_2Cl_2, 30 °C) δ, ppm: 18.71 (s, 1H, CH=Ru), 7.54 (dt, *J* 1.1 and 7.5 Hz, 1H, H-4-C_6H_4), 7.22 (br. t, *J* 7.5 Hz, 1H, H-5-C_6H_4), 7.08 (s, 4H, H-3,5-Mes), 7.03 (br. d, *J* 7.5 Hz, 1H, H-3-C_6H_4), 6.76 (br. d, *J* 7.5 Hz, 1H, H-6-C_6H_4), 4.20 (very br. s, 2H, N-CH_2-C_6H_4), 4.11 (s, 4H, N-CH_2-CH_2-N), 2.50 (very br. s, 12H, Me-2,6-Mes), 2.44 (s, 6H, Me-4-Mes), 1.87 (s, 6H, NMe_2). ^{13}C NMR (75.5 MHz, CD_2Cl_2, 30 °C) δ, ppm: 311.6 (C=Ru), 213.2 (N-C-N), 148.3 (C-2-C_6H_4), 138.8 (very br. s, 4C, C-2,6-Mes), 138.4 (2C, C-1-Mes), 136.2 (br. s, 2C, C-4-Mes), 133.4 (C-1-C_6H_4), 130.7 (C-3-C_6H_4), 129.2 (4C, C-3,5-Mes),

128.6 (C-5-C_6H_4), 128.3 (C-4-C_6H_4), 126.7 (C-6-C_6H_4), 65.5 (N-*C*H_2-C_6H_4), 51.5 (br. s, 2C, NCH_2CH_2N), 47.5 (2C, NMe_2), 20.8 (2C, Me-4-Mes), 19.2 (very br. s, 4C, Me-2,6-Mes). IR ν_{max}/cm^{-1} (KBr): 2907, 1605, 1472, 1261, 1004, 857, 745. HR-MALDI-ToF MS: *m/z* $[M\text{-}Cl]^+$ calcd. for $C_{31}H_{39}ClN_3Ru$: 590.1850; found: 590.1833. Anal. Calcd for $C_{31}H_{39}Cl_2N_3Ru$: C 59.51; H 6.28; N 6.72. Found: C 59.62; H 6.54; N 7.01.

[1,3-*Bis*(2,4,6-trimethylphenyl)imidazolidin-2-ylidene](dichloro){2-[(diethylamino)methyl]benzylidene}ruthenium (11b). Light-green powder, 2.23 g (3.42 mmol, 72%), m.p 203.1–203.5 °C (decomp.), dark-green prisms after slow crystallization from an heptane–CH_2Cl_2 mixture. ^{1}H NMR (500.1 MHz, CD_2Cl_2, 25 °C) δ, ppm: 18.73 (s, 1H, CH=Ru), 7.54 (dt, *J* 1.5 and 7.3 Hz, 1H, H-4-C_6H_4), 7.20 (dt, *J* 1.5 and 7.3 Hz, 1H, H-5-C_6H_4), 7.06 (br. s, 4H, H-3,5-Mes), 7.03 (br. d, *J* 7.3 Hz, 1H, H-3-C_6H_4), 6.63 (dd, *J* 7.3 and 1.5 Hz, 1H, H-6-C_6H_4), 4.24 (very br. s, 2H, N-C*H_2*-C_6H_4), 4.09-4.05 (m, 4H, N-C*H_2*-C*H_2*-N), 2.94-2.87 (m, 2H, C*H_2*Me), 2.65 (br. s, 6H, Me-4-Mes), 2.47 and 2.36 (all br. s, 3H and 6H, Me-2,6-Mes), 2.02-1.95 (m, 2H, C*H_2*Me), 0.47 (t, *J* 7.3 Hz, 6H, CH_2*Me*). ^{1}H NMR (600.1 MHz, $CDCl_3$, 26 °C) δ, ppm: 18.72 (s, 1H, CH=Ru), 7.45 (dt, *J* 1.2 and 7.3 Hz, 1H, H-4-C_6H_4), 7.10 (dt, *J* 1.2 and 7.3 Hz, 1H, H-5-C_6H_4), 7.02 (br. s, 4H, H-3,5-Mes), 6.95 (br. d, *J* 7.3 Hz, 1H, H-3-C_6H_4), 6.57 (br. d, *J* 7.3 Hz, 1H, H-6-C_6H_4), 4.23 (very br. s, 2H, N-C*H_2*-C_6H_4), 4.07-4.03 (m, 4H, N-C*H_2*-C*H_2*-N), 2.94-2.91 (m, 2H, C*H_2*Me), 2.65 (br. s, 6H, Me-4-Mes), 2.43, 2.34 and 2.32 (all br. s, 3H, 3H and 3H, Me-2,6-Mes), 2.02-1.98 (m, 2H, C*H_2*Me), 0.46 (t, *J* 7.3 Hz, 6H, CH_2*Me*). ^{13}C NMR (125.8 MHz, CD_2Cl_2, 25 °C) δ, ppm: 312.7 (C=Ru), 212.0 (N-C-N), 148.5 (C-1-C_6H_4), 139.9 (2C, C-1-Mes), 138.8 (1C), 138.3 (1C) and 137.7 (2C) (C-2,6-Mes), 135.1 (2C, C-4-Mes), 133.1 (C-2-C_6H_4), 130.6 (C-3-C_6H_4), 129.7 and 129.2 (br. s and br. s, 2C and 2C, C-3,5-Mes), 128.6 (C-4-C_6H_4), 128.4 (C-5-C_6H_4), 126.5 (C-6-C_6H_4), 58.5 (N-*C*H_2-C_6H_4), 52.1 and 50.9 (br. s and br. s, NCH_2CH_2N), 46.3 (br. s, 2C, N*C*H_2Me), 20.8 (2C, Me-4-Mes), 20.4 and

18.0 (2C and 2C, Me-2,6-Mes), 8.1 (br. s, 2C, *Me*CH_2). IR v_{max}/cm^{-1} (KBr): 2933, 1932, 1606, 1483, 1262, 754, 598, 580. HR-MALDI-ToF MS: *m/z* $[M-Cl]^+$ calcd. for $C_{33}H_{43}ClN_3Ru$: 618.2382; found: 618.23764. Anal. Calcd for $C_{33}H_{43}Cl_2N_3Ru$: C 60.63; H 6.63; N 6.43. Found: C 60.54; H 5.70; N 6.53.

[1,3-*Bis*(2,4,6-trimethylphenyl)imidazolidin-2-ylidene](dichloro){2-[(methylamino)methyl]benzylidene}ruthenium (11c). Light-green powder, 2.0 g (3.32 mmol, 70%), 196–203 °C (decomp.), green prisms after slow crystallization from an hexane-CH_2Cl_2 mixture. ^{1}H NMR (300.1 MHz, CD_2Cl_2, 25 °C) δ, ppm: 18.93 (s, 1H, CH=Ru), 7.50 (br. t, *J* 7.5 Hz, 1H, H-4-C_6H_4), 7.21 (br. t, *J* 7.5 Hz, 1H, H-5-C_6H_4), 7.12 (br. s, 2H, H-3,3'-Mes), 7.06 (br. s, 2H, H-5,5'-Mes), 7.03 (br. d, *J* 7.5 Hz, 1H, H-3-C_6H_4), 6.71 (br. d, *J* 7.5 Hz, 1H, H-6-C_6H_4), 4.48 (t, 1H, *J* 13.4 Hz, N-C*H_2*-C_6H_4), 4.14 (s, 4H, N-C*H_2*-C*H_2*-N), 3.26 (dd, *J* 2.3 and *J* 13.4 Hz, 1H, N-C*H_2*-C_6H_4), 2.56 (s, 6H, Me-2,6-Mes), 2.46 (br. s, 6H, Me-2,6-Mes), 2.44 (s, 6H, Me-4-Mes), 2.33 (m, 1H, NH), 1.93 (d, *J* 6.0 Hz, 3H, NMe). ^{13}C NMR (75.5 MHz, CD_2Cl_2, 30 °C) δ, ppm: 310.8 (C=Ru), 214.3 (N-C-N), 148.9 (C-C_6H_4), 138.9 (br. s, 2C, C-2,6-Mes), 138.7 (br. s, 2C, C-2,6-Mes), 138.5 (2C, C-1-Mes), 136.2 (2C, C-4-Mes), 132.6 (C-1-C_6H_4), 130.3 (C-3-C_6H_4), 129.21 and 129.17 (2C and 2C, C-3,5-Mes), 129.0 (C-5-C_6H_4), 127.7 (C-4-C_6H_4), 126.0 (C-6-C_6H_4), 54.3 (N-*C*H_2-C_6H_4), 51.5 (br. s, 2C, NCH_2CH_2N), 36.4 (s, NMe), 20.8 (2C, Me-4-Mes), 19.1 (br. s, 4C, Me-2,6-Mes). IR v_{max}/cm^{-1} (KBr): 2930, 1936, 1601, 1473, 1320, 761. HR-MALDI-ToF MS: *m/z* $[M-Cl]^+$ calcd. for $C_{30}H_{37}ClN_3Ru$: 576.1584; found: 576.1563. Anal. Calcd for $C_{30}H_{37}Cl_2N_3Ru$: C 58.91; H 6.10; N 6.87. Found: C 58.97; H 6.15; N 6.91.

[1,3-*Bis*(2,4,6-trimethylphenyl)imidazolidin-2-ylidene](dichloro)[2-(morpholin-4-ylmethyl)benzylidene]ruthenium (11d). Green powder, 2.02 g (3.03 mmol, 64%), 187-192 °C (decomp.), green plates after slow crystallization from an hexane-CH_2Cl_2 mixture. ^{1}H NMR (300.1 MHz, CD_2Cl_2, 30 °C) δ, ppm: 18.95 (s, 1H, CH=Ru), 7.49

(br. t, *J* 7.4 Hz, 1H, H-4-C_6H_4), 7.25 (br. t, *J* 7.4 Hz, 1H, H-5-C_6H_4), 7.10 (s, 4H, H-Mes), 7.07 (br. d, *J* 7.4 Hz, 1H, H-3-C_6H_4), 6.66 (br. d, *J* 7.4 Hz, 1H, H-6-C_6H_4), 4.12 (br. s, 6H, N-C*H*$_2$-C*H*$_2$-N and N-C*H*$_2$-C_6H_4), 3.56 (ddd, 2H, *J* 2.2, 7.0 and 12.3 Hz, H-2,6-Morph), 3.24-3.18 (m, 2H, H-2,6-Morph), 3.10-3.03 (m, 2H, H-3,5-Morph), 2.53 (br. s, 12H, Me-*ortho*-Mes), 2.44 (s, 6H, Me-*para*-Mes), 2.04 (ddd, 2H, *J* 2.0, 5.9 and 12.3 Hz, H-3,5-Morph). ^{13}C NMR (75.5 MHz, CD_2Cl_2, 25 °C) δ, ppm: 316.2 (C=Ru), 210.7 (N-C-N), 151.3 (C-2-C_6H_4), 139.2 (very br. s, 4C, C-2,6-Mes), 138.7 (2C, C-1-Mes), 136.2 (br. s, 2C, C-4-Mes), 131.5 (C-3-C_6H_4), 130.8 (C-1-C_6H_4), 129.3 (4C, C-3,5-Mes), 129.1 (C-5-C_6H_4), 128.1 (C-4-C_6H_4), 122.2 (C-6-C_6H_4), 62.7 (2C, C-2,6-Morph), 60.6 (N-*C*H$_2$-C_6H_4), 53.1 (2C, C-3,5-Morph), 51.6 (2C, N-*C*H$_2$-*C*H$_2$-N), 20.8 (2C, Me-4-Mes), ~ 19.4 (very br. s, 4C, Me-2,6-Mes). ^{1}H NMR (300.1 MHz, $CDCl_3$, 30 °C) δ, ppm: 18.92 (s, 1H, CH=Ru), 7.40 (br. t, *J* 7.5 Hz, 1H, H-4-C_6H_4), 7.15 (br. t, *J* 7.5 Hz, 1H, H-5-C_6H_4), 7.05 (s, 4H, H-Mes), 6.98 (br. d, *J* 7.5 Hz, 1H, H-3-C_6H_4), 6.59 (br. d, *J* 7.5 Hz, 1H, H-6-C_6H_4), 4.12 (s, 2H, N-C*H*$_2$-C_6H_4), 4.09 (s, 4H, N-C*H*$_2$-C*H*$_2$-N), 3.58-3.52 (m, 2H, H-2,6-Morph), 3.25-3.19 (m, 2H, H-2,6-Morph), 3.11-3.05 (m, 2H, H-3,5-Morph), 2.52 (very br. s, 12H, Me-*ortho*-Mes), 2.38 (s, 6H, Me-*para*-Mes), 2.07-2.02 (m, 2H, H-3,5-Morph). ^{13}C NMR (75.5 MHz, $CDCl_3$, 30 °C) δ, ppm: 317.5 (C=Ru), 211.3 (N-C-N), 151.1 (C-2-C_6H_4), 138.7 (very br. s, 4C, C-2,6-Mes), 140.1 (very br. signal, 2C, C-1-Mes), 135.2 (2C, C-4-Mes), 131.4 (C-3-C_6H_4), 130.9 (C-1-C_6H_4), 129.4 (4C, C-3,5-Mes), 129.1 (C-5-C_6H_4), 128.2 (C-4-C_6H_4), 122.8 (C-6-C_6H_4), 62.8 (2C, C-2,6-Morph), 60.9 (N-*C*H$_2$-C_6H_4), 53.2 (2C, C-3,5-Morph), 51.5 (very br. signal, 2C, N-*C*H$_2$-*C*H$_2$-N), 21.0 (2C, Me-4-Mes), 20.4-18.6 (very br. signal, 4C, Me-2,6-Mes). IR ν_{max}/cm^{-1} (KBr): 2953, 2914, 1934, 1607, 1483, 1449, 1397, 1258, 1112, 996, 871, 852, 785, 749, 731, 580. HR-MALDI-ToF MS: *m/z* [M-Cl]$^+$ calcd. for $C_{33}H_{41}ClN_3ORu$: 632.2217; found: 632.2223. Anal. Calcd for $C_{33}H_{41}Cl_2N_3ORu$: C 59.36; H 6.19; N 6.29. Found: C 59.40; H 5.83; N 6.03.

General procedure for metathesis reactions. Styrene (**12**, 0.51 g, 4.80 mmol), or allylbenzene (**14**, 0.50 g, 4.23 mmol), or diethyl diallylmalonate (**17**, 0.48 g, 2.0 mmol), or diallyltosylamide (**19**, 0.50 g, 2.0 mmol), or the mixture of norbornene (**21**, 0.5 g, 5.3 mmol) and styrene (**12**, 1.11 g, 10.6 mmol), or the mixture of **21** (0.5 g, 5.3 mmol) and hex-1-ene (**24,** 0.89 g, 10.6 mmol) was added to a solution of catalyst (**11a**, **b**, **d**) 1.00 or 0.10 or 0.01 mol % (see Table 3) in dry solvent (10 mL) in a Schlenk flask purged with argon. The resulting mixture was heated at reflux for 4 h under continuous stirring and an argon atmosphere (as rule, after that time the green color of the reaction mixtures changes to yellow). The solvent was evaporated under reduced pressure, polymeric and inorganic products were separated by filtration of the residue through basic Al_2O_3 (1.5 × 2 cm), Et_2O or CH_2Cl_2 (2 × 15 mL) were used as an eluent. Solvent was evaporated under reduced pressure to give a mixture of metathesis products. Compositions of these mixtures were established using GC–MS analysis (see the Supporting Information File 2) or ^{1}H NMR analysis. Most of the products were isolated and fully characterized by NMR. Well-crystallizing stilbene (**13**) was isolated from the most part of styrene metathesis reactions by silica gel column chromatography using hexane as eluent, see data of Table 3 (entry 8–10, 12, 13, 15–22).

Typical procedure for RCM reactions. Diethyl diallylmalonate **17** (0.48 g, 2.0 mmol, Entry 28 of Table 3) or diallyltosylamide **19** (0.50 g, 2.0 mmol, Entry 32 of Table 3) were added to a solution of catalyst (**11b** or **11d**) 0.1 mol % (1.3 mg, 0.002 mmol) in dry chloroform (10 mL) in a Schlenk flask purged with argon. The resulting mixture was heated at reflux for 4 h under continuous stirring in an argon atmosphere (after this time the green color of the reaction mixtures changes to yellow). The solvent was evaporated under reduced pressure, polymeric and inorganic products were separated by filtration of the residue through basic Al_2O_3 (1.5 × 2 cm), Et_2O or CH_2Cl_2

(3 × 15 mL) were used as an eluent. The solvent was evaporated under reduced pressure yield a mixture of metathesis products. The composition of the mixtures was established by GC/MS.

Diethyl cyclopent-3-ene-1,1-dicarboxylate (18). Viscous colorless oil, 0.39 g (98 %). ^{1}H NMR (600 MHz, $CDCl_3$) δ, ppm: 5.57 (br. s, 2H, H-3,4), 4.17 (q, *J* 7.2 Hz, 4H, CH_2CH_3), 2.98 (br. s, 4H, H-2,5), 1.22 (t, *J* 7.2 Hz, 6H, CH_2CH_3). ^{13}C NMR (150.9 MHz, $CDCl_3$) δ, ppm: 172.3 (2C, C=O), 127.8 (2C, C-3,4), 61.6 (2C, CH_2CH_3), 58.9 (C-1), 40.9 (2C, C-2,5), 14.1 (2C, CH_2CH_3).

1-Tosyl-2,5-dihydro-1*H*-pyrrole (20). White solid, 0.41 g (98 %), m.p 123.5-124.5 °C. ^{1}H NMR (600 MHz, $CDCl_3$) δ, ppm: 7.73 (d, *J* 8.1 Hz, 2H, H-C_6H_4), 7.32 (d, *J* 8.1 Hz, 2H, H-C_6H_4), 5.65 (t, *J* 4.5 Hz, 2H, CH=CH), 4.12 (t, *J* 4.5 Hz, 4H, (CH_2)×2), 2.44 (s, 3H, Me). ^{13}C NMR (150.9 MHz, $CDCl_3$) δ, ppm: 143.4, 134.3, 129.8 (2C), 127.4 (2C), 125.5 (2C), 54.8 (2C), 21.5. Spectral data of compounds **18** and **20** are in good agreement with those reported in the literature [9].

Stilbene (13). White solid, m.p 123-125 °C. ^{1}H NMR (600 MHz, $CDCl_3$) δ, ppm: 7.52 (dd, *J* 1.6 and *J* 7.4 Hz, 4H, H-2,6-Ph), 7.36 (br.t, 4H, *J* 7.4 Hz, H-3,5-Ph), 7.26 (br.t, 2H, *J* 7.4 Hz, H-4-Ph), 7.12 (s, 2H, CH=CH). ^{13}C NMR (150.9 MHz, $CDCl_3$) δ, ppm: 137.4 (2C, Ph-1), 128.80 (2C, *C*H=*C*H), 128.78 (4C, C-3,5-Ph), 127.7 (2C, C-4-Ph), 126.6 (4C, 2,6-Ph).

1,1'-(2*E*)-But-2-ene-1,4-diyldibenzene (15). Viscous colorless oil. ^{1}H NMR (600 MHz, $CDCl_3$) δ, ppm: 7.30-7.27 (m, 4H, H-Ph), 7.21 (m, 6H, H-Ph), 5.68-5.66 (m, 2H, CH=CH), 3.37-3.36 (m, 4H, CH_2 × 2). ^{13}C NMR (150.9 MHz, $CDCl_3$) δ, ppm: 140.8 (2C, Ph-1), 130.5 (2C, *C*H=*C*H), 128.6 (4C, C-Ph), 128.5 (4C, C-Ph), 126.1 (2C, 4-Ph), 39.0 (2C, CH_2 × 2).

References

[1] Bruker (2013). *APEX2* and *SAINT*. Bruker AXS Inc., Madison, Wisconsin, USA.

[2] Bruker (2014). *SADABS*. Bruker AXS Inc., Madison, Wisconsin, USA.

[3] Sheldrick, G. M. *Acta Cryst.* **2015**, *A71*, 3–8. doi: 10.1107/S2053273314026370

[4] Sheldrick, G. M. *Acta Cryst.* **2015**, *C71*, 3–8. doi: 10.1107/S2053229614024218

[5] Dolomanov, O. V.; Bourhis, L. J.; Gildea, R. J.; Howard, J. A. K.; Puschmann, H. *J. Appl. Cryst*. **2009**, *42*, 339-341. doi: 10.1107/S0021889808042726

[6] Molander, G. A.; Pack, S. K. *Tetrahedron* **2003**, *59*, 10581-10591. doi: 10.1016/j.tet.2003.08.071

[7] “Method of obtaining *N*,*N*-diaryl-substituted 2-trichloromethyl-imidazolidines”. Bespalova, N. B.; Cheredilin, D.; Afanas'ev, V. V.; Zemtsov, D. B. Patent RU2497810 C1, priority date: 28-06-2012.

[8] Shcheglova, N. M.; Kolesnik, V. D.; Ashirov, R. V.; Krasnokutskaya, E. A. *Russ. Chem. Bull.* **2016**, *65*, 490-497. doi: 10.1007/s11172-016-1327-x

[9] Hongfa, C.; Tian, J.; Bazzi, H. S.; Bergbreiter, D. E. *Org. Lett*. **2007**, *9*, 3259–3261. DOI: 10.1021/ol071210k
