# Supplementary material for: Hoveyda–Grubbs catalysts with an N→Ru coordinate bond in a six-membered ring. Synthesis of stable, industrially scalable, highly efficient ruthenium metathesis catalysts and 2-vinylbenzylamine ligands as their precursors
Source: Beilstein J Org Chem. 2019 Mar 22;15:769–79. doi: 10.3762/bjoc.15.73 (PMC6444410; doi:10.3762/bjoc.15.73)

BEILSTEIN JOURNAL OF ORGANIC CHEMISTRY

# Supporting Information

for

# Hoveyda–Grubbs catalysts with an N→Ru coordinate bond in a six-membered ring. Synthesis of stable, industrially scalable, highly efficient ruthenium metathesis catalysts and 2-vinylbenzylamine ligands as their precursors

Kirill B. Polyanskii, Kseniia A. Alekseeva, Pavel V. Raspertov, Pavel A. Kumandin, Eugeniya V. Nikitina, Atash V. Gurbanov and Fedor I. Zubkov

*Beilstein J. Org. Chem.* **2019,** *15,* 769–779. doi:10.3762/bjoc.15.73

## Copies of NMR spectra of synthesised compounds and selected GC–MS data of the metathesis products. Check-cif reports for compounds 11a–c

License and Terms: This is a supporting information file under the terms of the Creative Commons Attribution License (http://creativecommons.org/licenses/by/4.0). Please note that the reuse, redistribution and reproduction in particular requires that the authors and source are credited.
The license is subject to the *Beilstein Journal of Organic Chemistry* terms and conditions: (https://www.beilstein-journals.org/bjoc)

**Table of contents**


| | | |
|---|---|---|
| 1. | Copies of $^{1}H$ and $^{13}C$ NMR spectra for compounds **1**, **3-5**, **7**, **11**, **13**, **15**, **18** | 3-204 |
| 2. | Selected GC-MS data for metathesis products (**13-27**) | 205-246 |
| 3. | Comparison of $^{1}H$ NMR and GC/MS methods for quantitative analysis of mixtures **17/18**, and **19/20** obtained in the course of metathesis reactions. | 247-256 |
| 4. | Experimental details and GPC analysis of polymer materials obtained in the metathesis reaction between norbornene **21** and styrene **12** (see Entry 35, Table 3) | 257-264 |
| 5. | Check-cif reports for compounds **11a**, **11b**, **11c** | 265-275 |

1. Copies of $^{1}$H and $^{13}$C NMR spectra for compounds **1**, **3-5**, **7**, **11**, **13**, **15**, **18**

| | | | | | | | |
|---|---|---|---|---|---|---|---|
| ***Acquisition Time (sec)*** | 1.9818 | ***Comment*** | single_pulse | ***Date*** | 22 Jul 1990 10:41:08 | | |
| ***Date Stamp*** | 13 Dec 2017 13:16:16 | | | ***File Name*** | C:\Users\Fedor\Desktop\12.12.17\FZ6277-1.jdf | ***Frequency (MHz)*** | 600.17 |
| ***Nucleus*** | 1H | ***Number of Transients*** | 8 | ***Origin*** | ECA 600 | ***Original Points Count*** | 32768 |
| ***Owner*** | delta | | | | | | |
| ***Points Count*** | 32768 | ***Pulse Sequence*** | single_pulse.ex2 | ***Receiver Gain*** | 26.00 | ***Solvent*** | CHLOROFORM-d |
| ***Spectrum Offset (Hz)*** | 5397.0029 | ***Sweep Width (Hz)*** | 16534.39 | ***Temperature (degree C)*** | 19.700 | | |

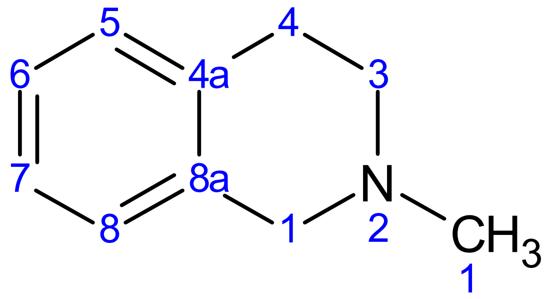


1a

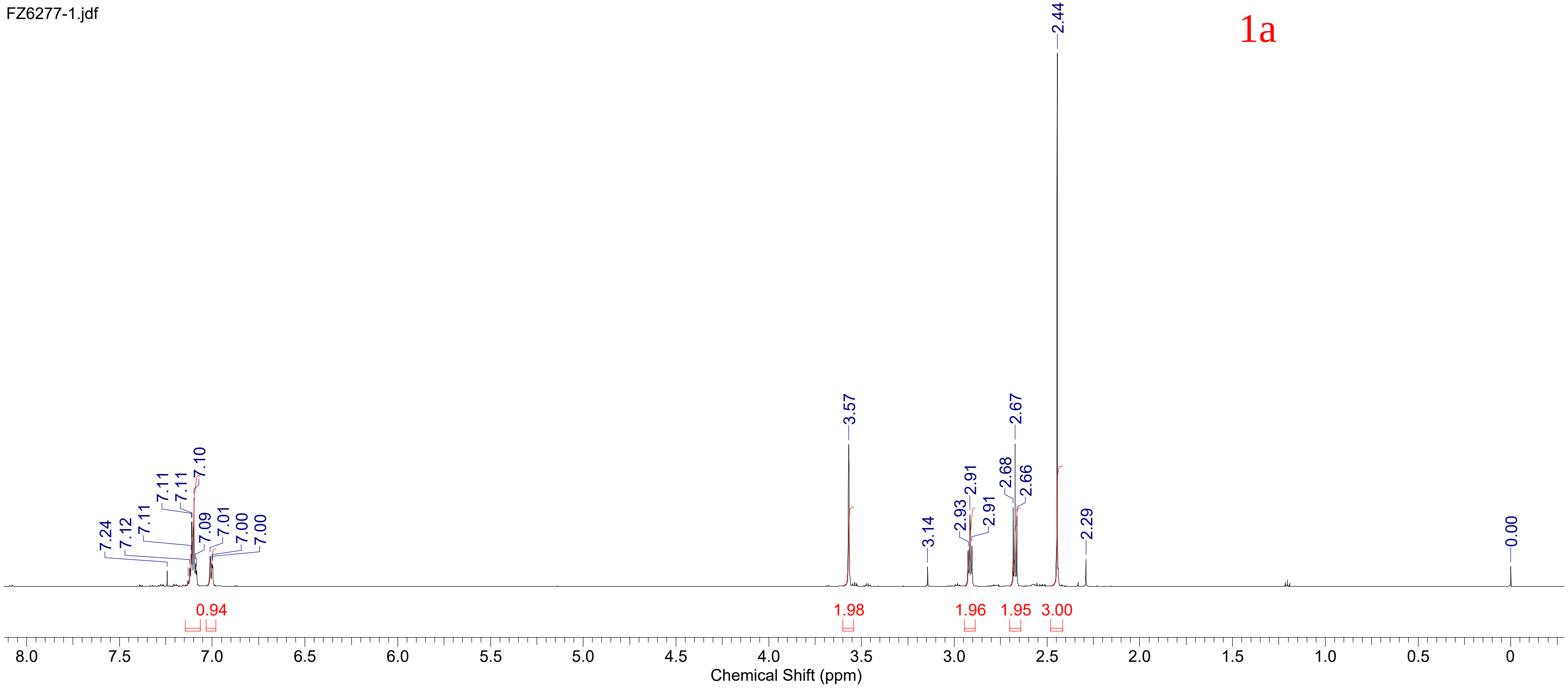

| | | | | | | | |
|---|---|---|---|---|---|---|---|
| ***Acquisition Time (sec)*** | 1.9818 | ***Comment*** | single_pulse | ***Date*** | 22 Jul 1990 10:41:08 | | |
| ***Date Stamp*** | 13 Dec 2017 13:16:16 | | | ***File Name*** | C:\Users\Fedor\Desktop\12.12.17\FZ6277-1.jdf | ***Frequency (MHz)*** | 600.17 |
| ***Nucleus*** | 1H | ***Number of Transients*** | 8 | ***Origin*** | ECA 600 | ***Original Points Count*** 32768 | ***Owner*** delta |
| ***Points Count*** | 32768 | ***Pulse Sequence*** | single_pulse.ex2 | | | ***Receiver Gain*** 26.00 | ***Solvent*** CHLOROFORM-d |
| ***Spectrum Offset (Hz)*** | 5397.0029 | ***Sweep Width (Hz)*** | 16534.39 | ***Temperature (degree C)*** | 19.700 | | |

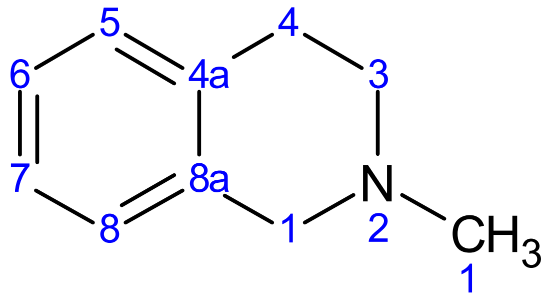


1a

FZ6277-1.jdf

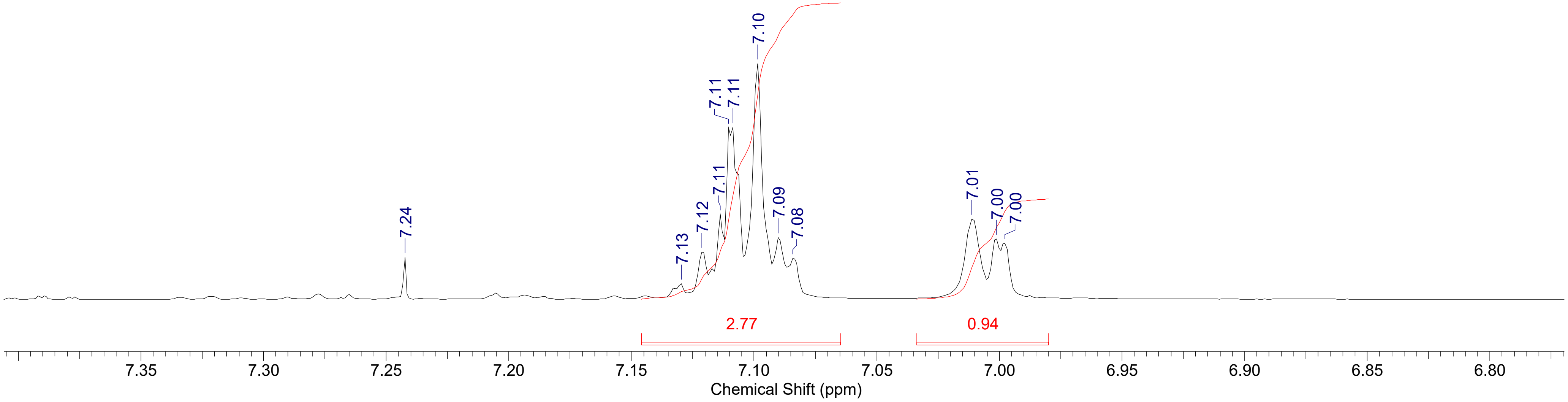

| | | | | | | | |
|---|---|---|---|---|---|---|---|
| ***Acquisition Time (sec)*** | 1.9818 | ***Comment*** | single_pulse | ***Date*** | 22 Jul 1990 10:41:08 | | |
| ***Date Stamp*** | 13 Dec 2017 13:16:16 | | | ***File Name*** | C:\Users\Fedor\Desktop\12.12.17\FZ6277-1.jdf | ***Frequency (MHz)*** | 600.17 |
| ***Nucleus*** | 1H | ***Number of Transients*** | 8 | ***Origin*** | ECA 600 | ***Original Points Count*** | 32768 |
| ***Owner*** | delta | | | | | | |
| ***Points Count*** | 32768 | ***Pulse Sequence*** | single_pulse.ex2 | ***Receiver Gain*** | 26.00 | ***Solvent*** | CHLOROFORM-d |
| ***Spectrum Offset (Hz)*** | 5397.0029 | ***Sweep Width (Hz)*** | 16534.39 | ***Temperature (degree C)*** | 19.700 | | |

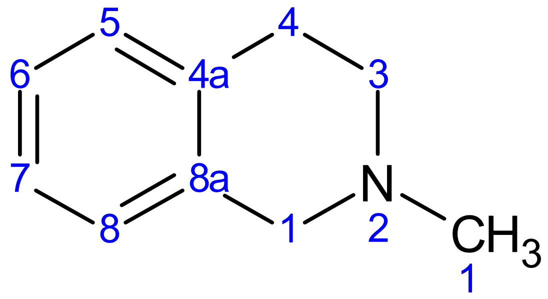


FZ6277-1.jdf

1a

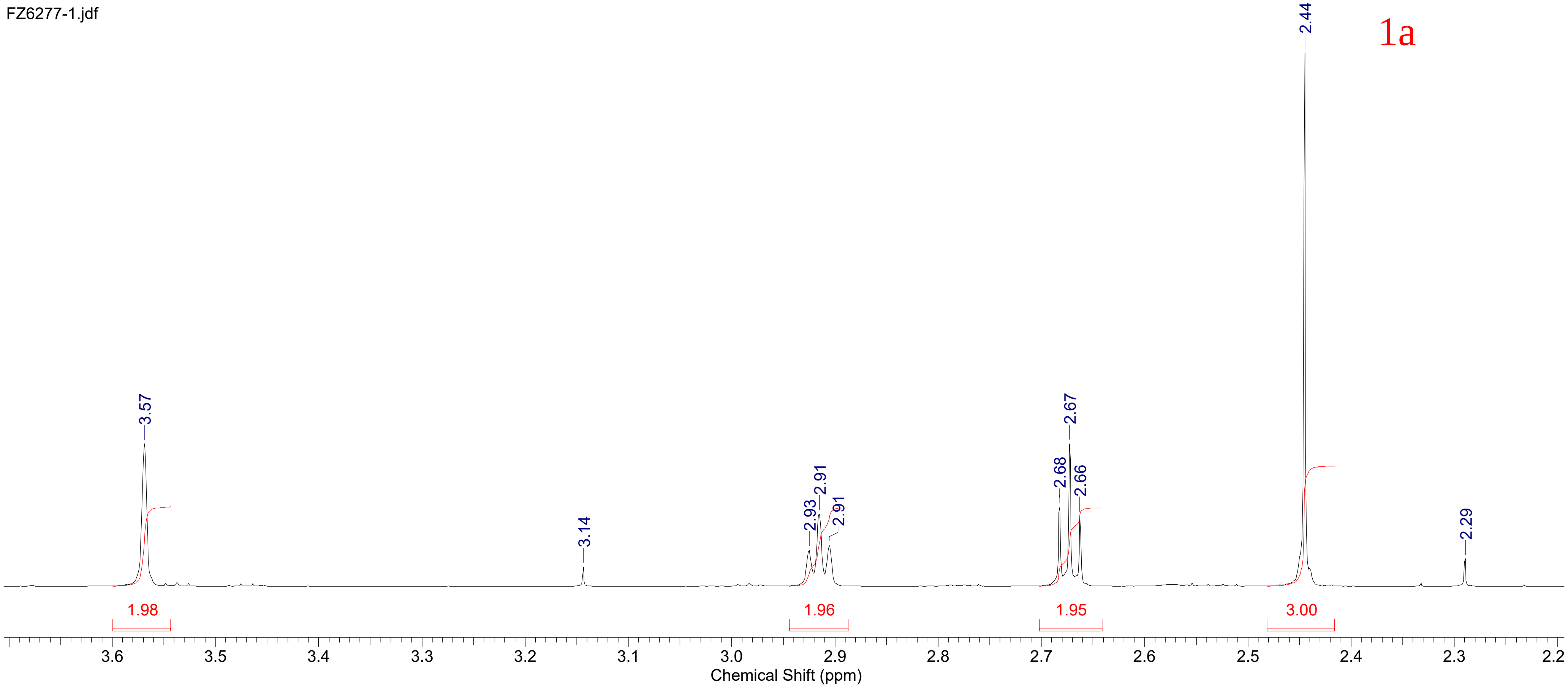

| | | | | | | | |
|---|---|---|---|---|---|---|---|
| **Acquisition Time (sec)** | 1.9818 | **Comment** | single_pulse | **Date** | 22 Apr 1990 07:55:10 | | |
| **Date Stamp** | 18 Apr 2018 12:42:49 | | | **File Name** | C:\Users\Fedor\Desktop\17.04.18\FZ6623-1.jdf | **Frequency (MHz)** | 600.17 |
| **Nucleus** | 1H | **Number of Transients** | 8 | **Origin** | ECA 600 | **Original Points Count** 32768 | **Owner** delta |
| **Points Count** | 32768 | **Pulse Sequence** | single_pulse.ex2 | | | **Receiver Gain** 30.00 | **Solvent** CHLOROFORM-d |
| **Spectrum Offset (Hz)** | 5401.5503 | **Sweep Width (Hz)** | 16534.39 | **Temperature (degree C)** | 21.800 | | |

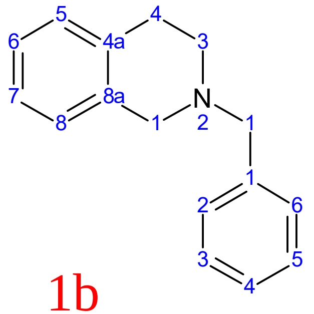


1b

FZ6623-1.jdf

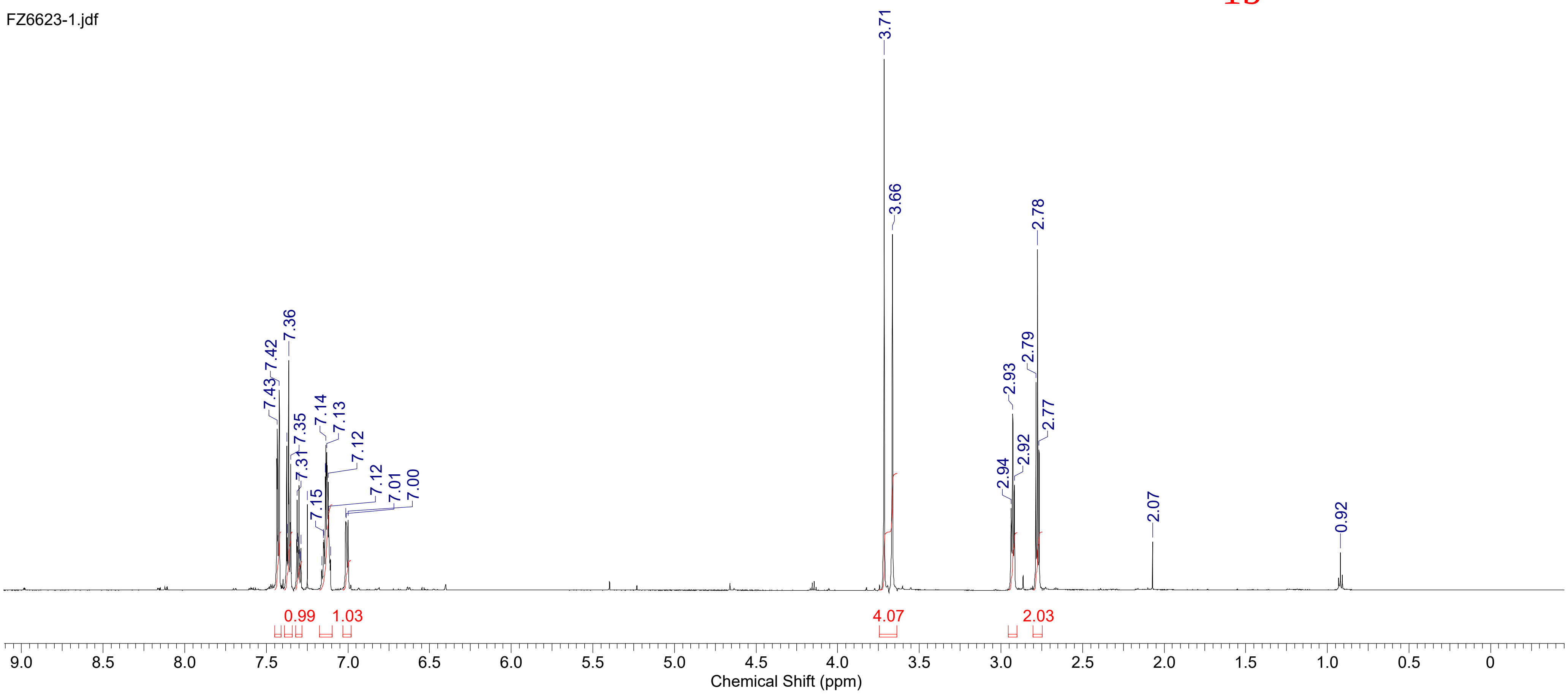

| | | | | | | | | | |
|---|---|---|---|---|---|---|---|---|---|
| ***Acquisition Time (sec)*** | 1.9818 | ***Comment*** | single_pulse | ***Date*** | 22 Apr 1990 07:55:10 | | | | |
| ***Date Stamp*** | 18 Apr 2018 12:42:49 | | | ***File Name*** | C:\Users\Fedor\Desktop\17.04.18\FZ6623-1.jdf | | | ***Frequency (MHz)*** | 600.17 |
| ***Nucleus*** | 1H | ***Number of Transients*** | 8 | ***Origin*** | ECA 600 | ***Original Points Count*** | 32768 | ***Owner*** | delta |
| ***Points Count*** | 32768 | ***Pulse Sequence*** | single_pulse.ex2 | | | ***Receiver Gain*** | 30.00 | ***Solvent*** | CHLOROFORM-d |
| ***Spectrum Offset (Hz)*** | 5401.5503 | ***Sweep Width (Hz)*** | 16534.39 | ***Temperature (degree C)*** | 21.800 | | | | |

1b

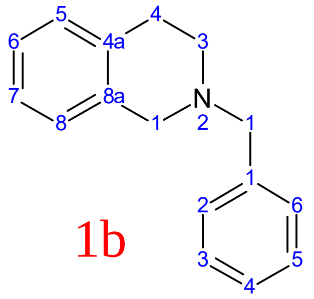


FZ6623-1.jdf

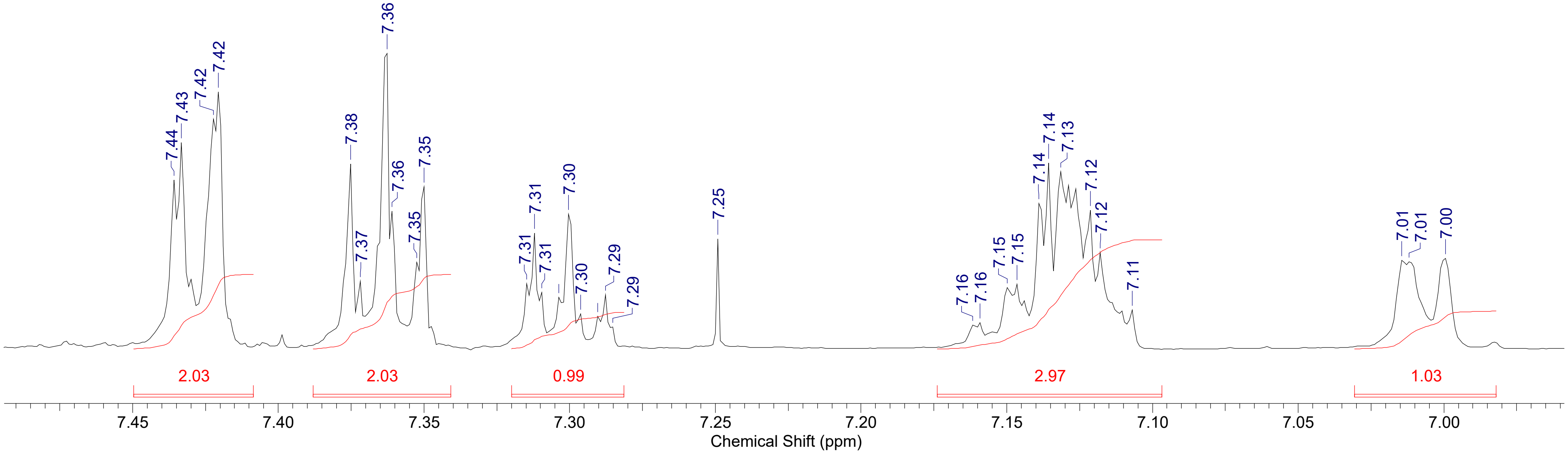

| | | | | | | | |
|---|---|---|---|---|---|---|---|
| ***Acquisition Time (sec)*** | 1.9818 | ***Comment*** | single_pulse | ***Date*** | 22 Apr 1990 07:55:10 | | |
| ***Date Stamp*** | 18 Apr 2018 12:42:49 | | | ***File Name*** | C:\Users\Fedor\Desktop\17.04.18\FZ6623-1.jdf | ***Frequency (MHz)*** | 600.17 |
| ***Nucleus*** | 1H | ***Number of Transients*** | 8 | ***Origin*** | ECA 600 | ***Original Points Count*** | 32768 |
| ***Owner*** | delta | | | | | | |
| ***Points Count*** | 32768 | ***Pulse Sequence*** | single_pulse.ex2 | | | ***Receiver Gain*** | 30.00 |
| ***Solvent*** | CHLOROFORM-d | | | | | | |
| ***Spectrum Offset (Hz)*** | 5401.5503 | ***Sweep Width (Hz)*** | 16534.39 | ***Temperature (degree C)*** | 21.800 | | |

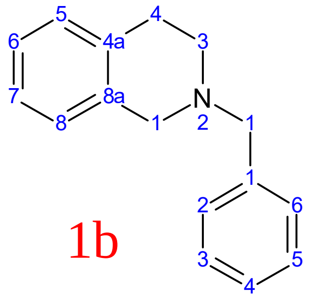


1b

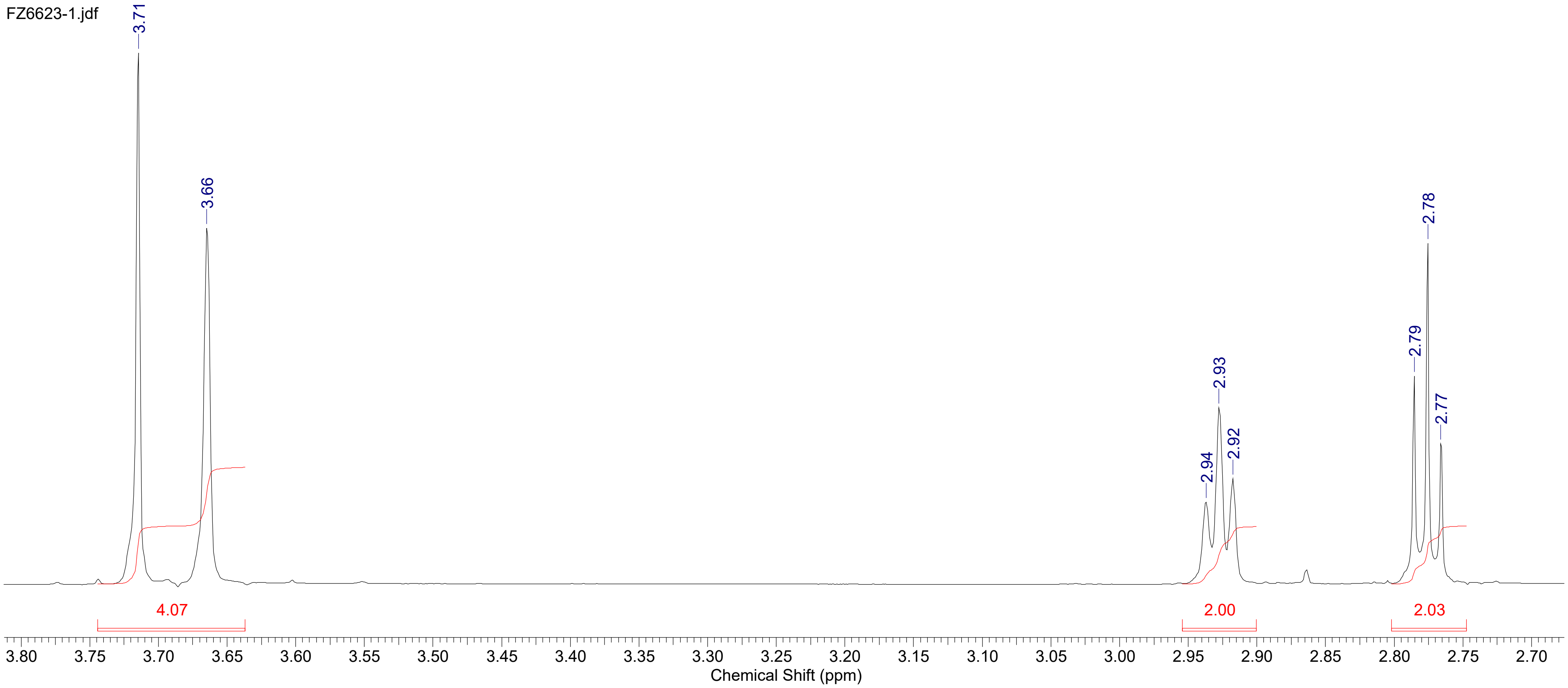

| | | | | | |
|---|---|---|---|---|---|
| ***Acquisition Time (sec)*** | 1.5729 | ***Date*** | 11 Jul 2018 14:51:44 | | |
| ***File Name*** | C:\Users\Fedor\Desktop\11.07.18\fz6800\fz6800_001000fid | | | | |
| ***Number of Transients*** | 1 | ***Origin*** | spect | ***Original Points Count*** | 16384 |
| ***Pulse Sequence*** | zg | ***Receiver Gain*** | 64.00 | ***SW(cyclical) (Hz)*** | 10416.67 |
| ***Spectrum Offset (Hz)*** | 2823.8298 | ***Sweep Width (Hz)*** | 10416.03 | ***Temperature (degree C)*** | 21.500 |

| | | | |
|---|---|---|---|
| ***Date Stamp*** | 11 Jul 2018 14:51:44 | | |
| ***Frequency (MHz)*** | 400.13 | ***Nucleus*** | 1H |
| ***Owner*** | pr0vider | ***Points Count*** | 16384 |
| ***Solvent*** | CHLOROFORM-d | | |

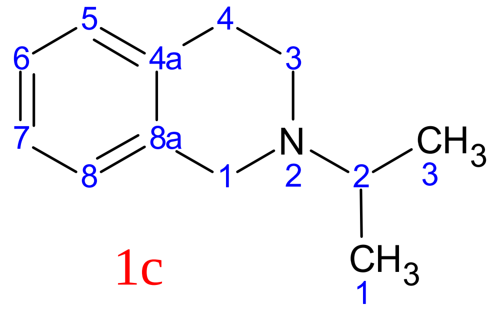


1c

fz6800_001000fid

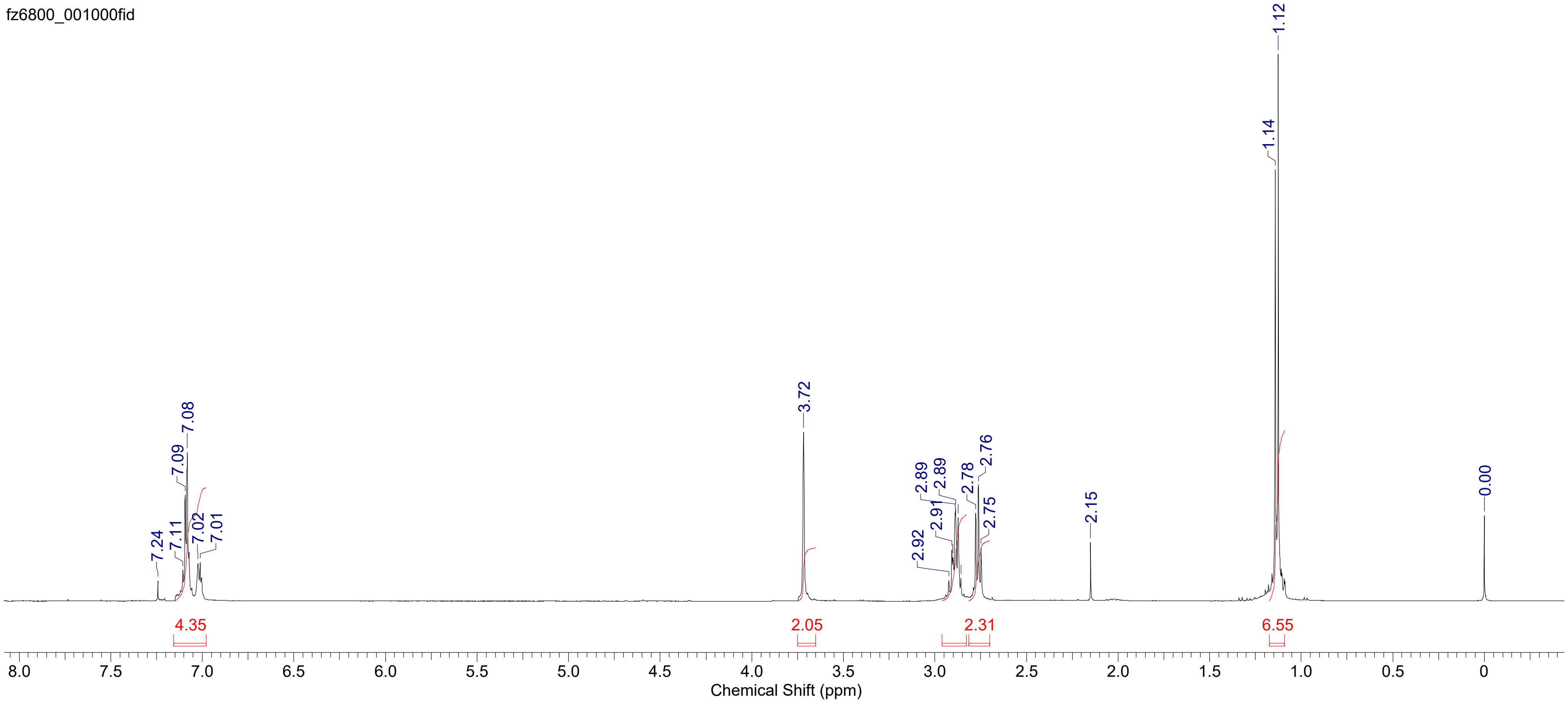

| Acquisition Time (sec) | 1.5729 | Date | 11 Jul 2018 14:51:44 | | | Date Stamp | 11 Jul 2018 14:51:44 | | |
|---|---|---|---|---|---|---|---|---|---|
| File Name | C:\Users\Fedor\Desktop\11.07.18\fz6800\fz6800_001000fid | | | | | Frequency (MHz) | 400.13 | Nucleus | 1H |
| Number of Transients | 1 | Origin | spect | Original Points Count | 16384 | Owner | pr0vider | Points Count | 16384 |
| Pulse Sequence | zg | Receiver Gain | 64.00 | SW(cyclical) (Hz) | 10416.67 | Solvent | CHLOROFORM-d | | |
| Spectrum Offset (Hz) | 2823.8298 | Sweep Width (Hz) | 10416.03 | Temperature (degree C) | 21.500 | | | | |

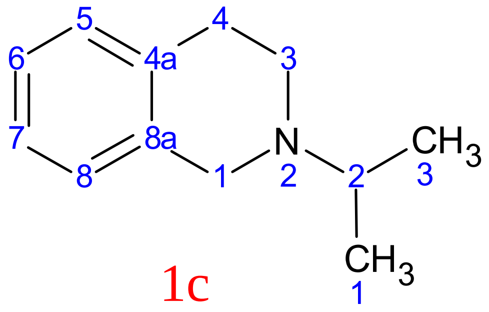


1c

fz6800_001000fid

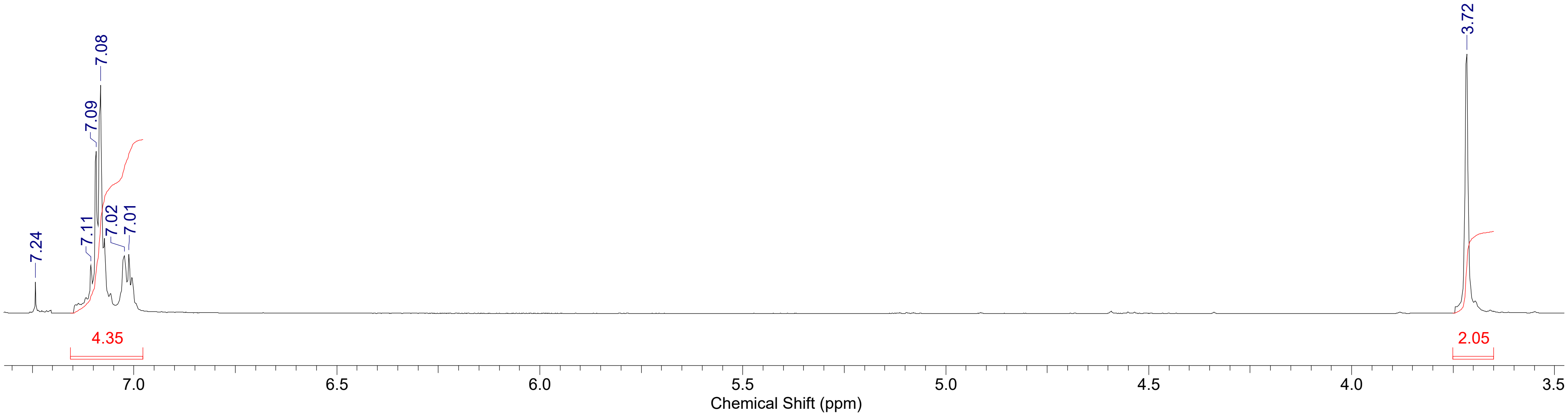

| Acquisition Time (sec) | 1.5729 | Date | 11 Jul 2018 14:51:44 | | | Date Stamp | 11 Jul 2018 14:51:44 | | |
|---|---|---|---|---|---|---|---|---|---|
| File Name | C:\Users\Fedor\Desktop\11.07.18\fz6800\fz6800_001000fid | | | | | Frequency (MHz) | 400.13 | Nucleus | 1H |
| Number of Transients | 1 | Origin | spect | Original Points Count | 16384 | Owner | pr0vider | Points Count | 16384 |
| Pulse Sequence | zg | Receiver Gain | 64.00 | SW(cyclical) (Hz) | 10416.67 | Solvent | CHLOROFORM-d | | |
| Spectrum Offset (Hz) | 2823.8298 | Sweep Width (Hz) | 10416.03 | Temperature (degree C) | 21.500 | | | | |

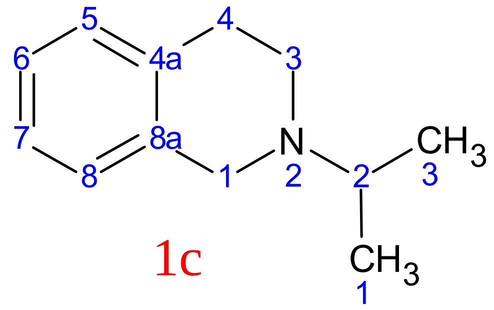


1c

fz6800_001000fid

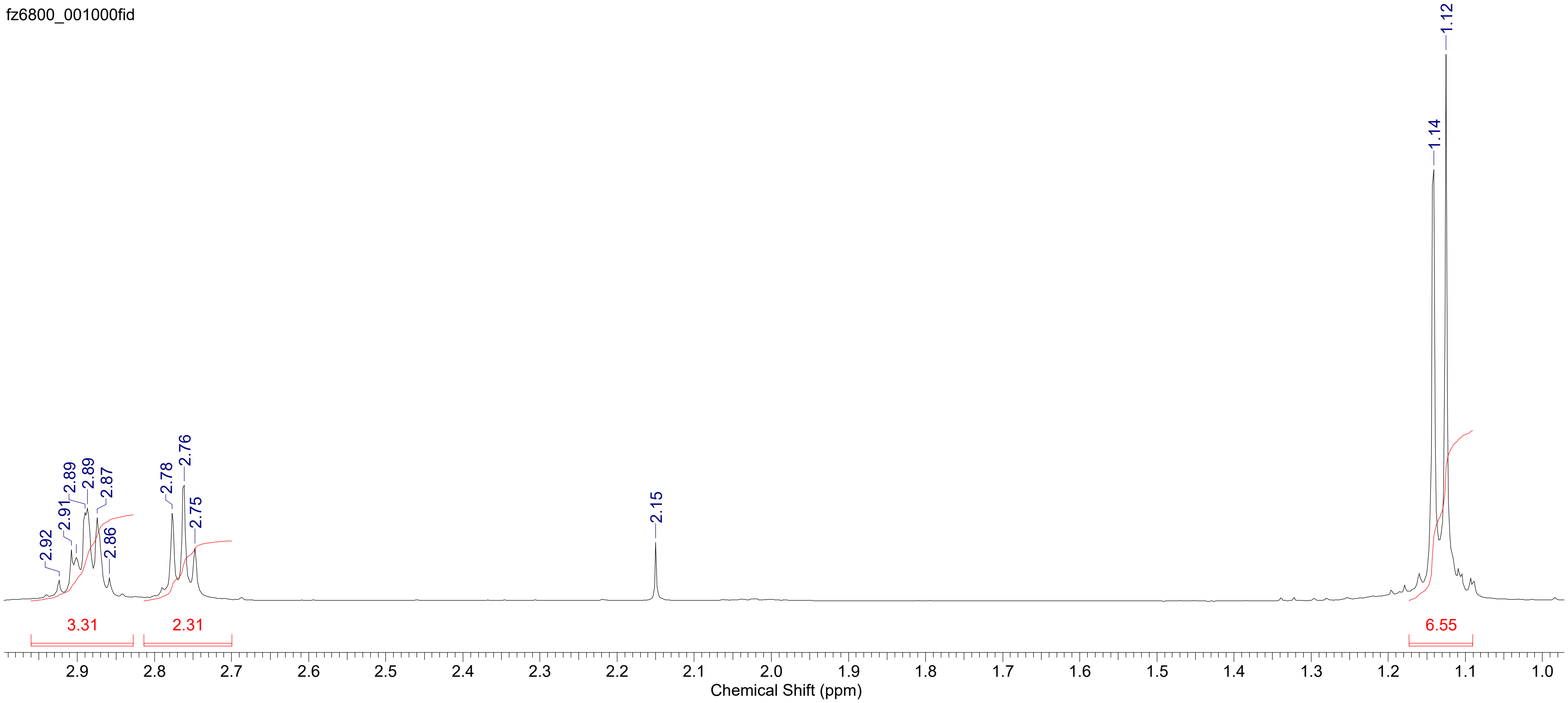

| Acquisition Time (sec) | 0.6816 | Date | 16 Jul 2018 15:28:00 | | | Date Stamp | 16 Jul 2018 15:28:00 |
|---|---|---|---|---|---|---|---|
| File Name | C:\Users\Fedor\Desktop\11.07.18\fz6800-13\fz6800-13_013000fid | | | | | Frequency (MHz) | 100.62 |
| Nucleus | 13C | Number of Transients | 80 | Origin | spect | Original Points Count | 16384 |
| Owner | pr0vider | Points Count | 16384 | Pulse Sequence | zgpg30 | Receiver Gain | 32768.00 |
| SW(cyclical) (Hz) | 24038.46 | Solvent | CHLOROFORM-d | | | Spectrum Offset (Hz) | 10055.3965 |
| Sweep Width (Hz) | 24037.00 | Temperature (degree C) | 21.900 | | | | |

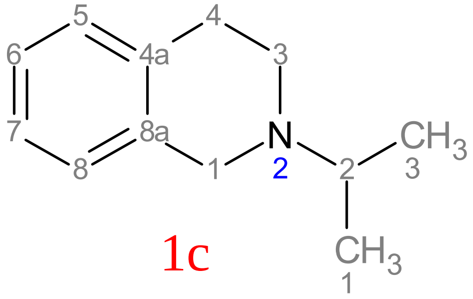


1c

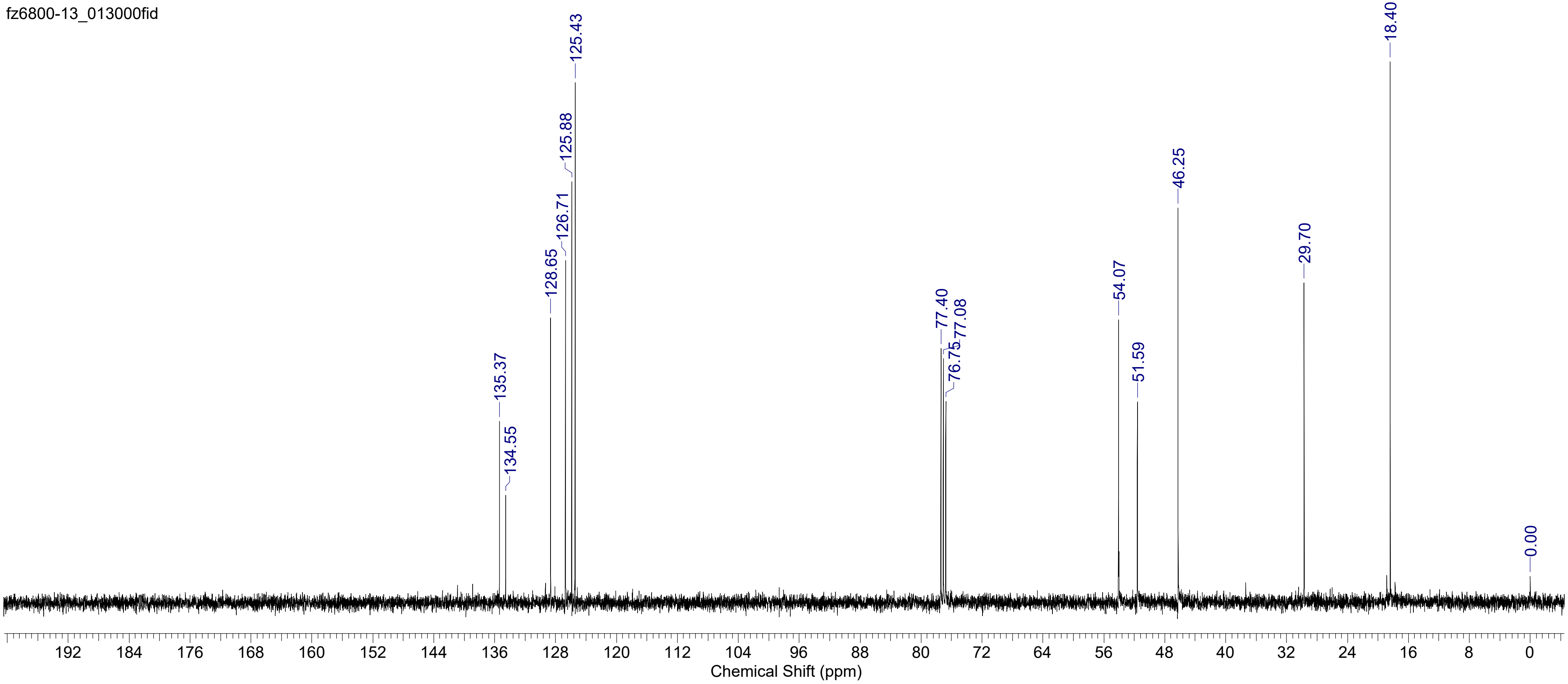

| | | | | | | | |
|---|---|---|---|---|---|---|---|
| ***Acquisition Time (sec)*** | 0.6816 | ***Date*** | 16 Jul 2018 15:28:00 | | | ***Date Stamp*** | 16 Jul 2018 15:28:00 |
| ***File Name*** | C:\Users\Fedor\Desktop\11.07.18\fz6800-13\fz6800-13_013000fid | | | | | ***Frequency (MHz)*** | 100.62 |
| ***Nucleus*** | 13C | ***Number of Transients*** | 80 | ***Origin*** | spect | ***Original Points Count*** | 16384 |
| ***Owner*** | pr0vider | ***Points Count*** | 16384 | ***Pulse Sequence*** | zgpg30 | ***Receiver Gain*** | 32768.00 |
| ***SW(cyclical) (Hz)*** | 24038.46 | ***Solvent*** | CHLOROFORM-d | | | ***Spectrum Offset (Hz)*** | 10055.3965 |
| ***Sweep Width (Hz)*** | 24037.00 | ***Temperature (degree C)*** | 21.900 | | | | |

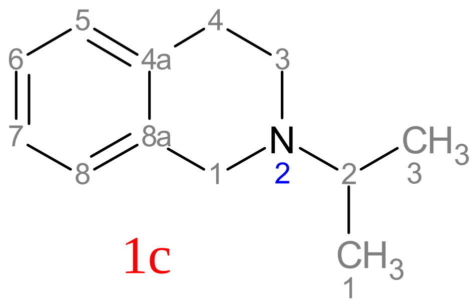


1c

fz6800-13_013000fid

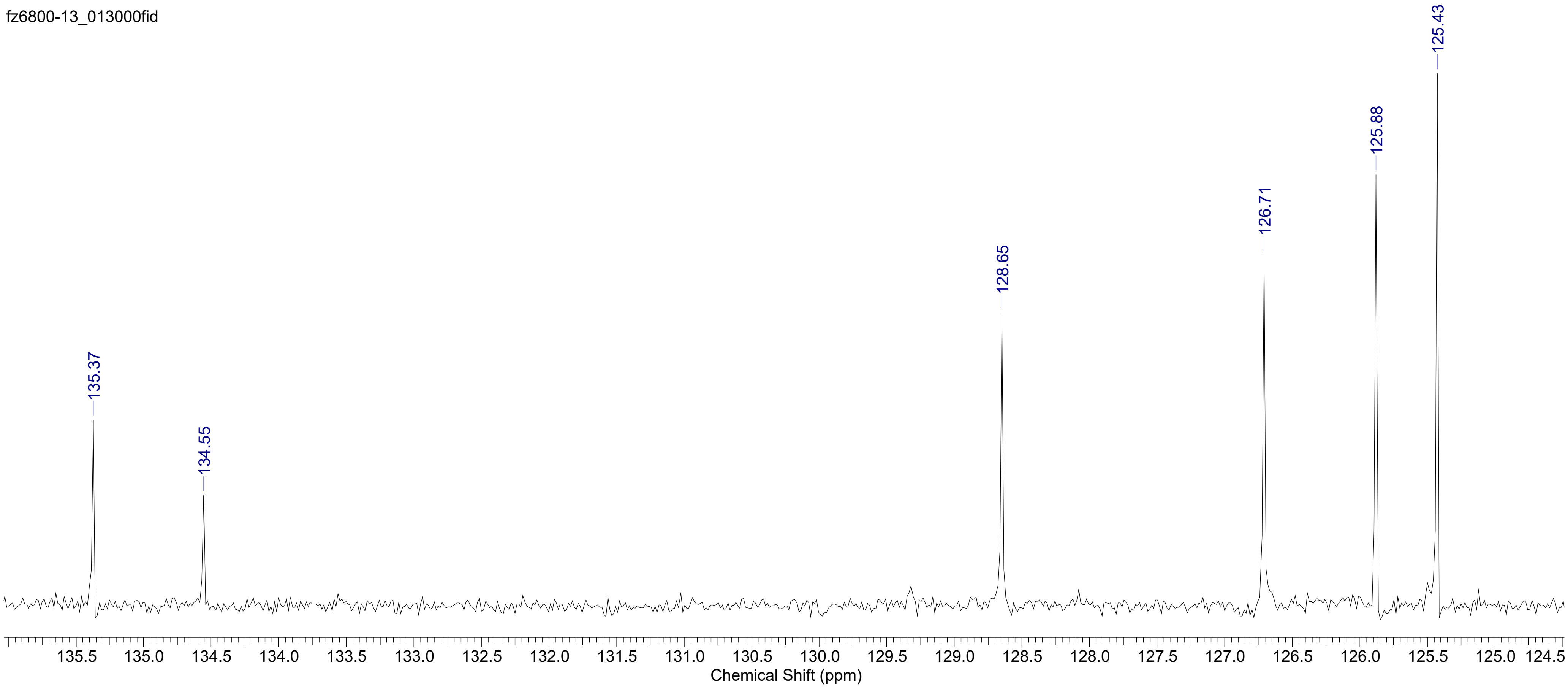

| | | | | | | | |
|---|---|---|---|---|---|---|---|
| **Acquisition Time (sec)** | 0.6816 | **Date** | 16 Jul 2018 15:28:00 | | | **Date Stamp** | 16 Jul 2018 15:28:00 |
| **File Name** | C:\Users\Fedor\Desktop\11.07.18\fz6800-13\fz6800-13_013000fid | | | | | **Frequency (MHz)** | 100.62 |
| **Nucleus** | 13C | **Number of Transients** | 80 | **Origin** | spect | **Original Points Count** | 16384 |
| **Owner** | pr0vider | **Points Count** | 16384 | **Pulse Sequence** | zgpg30 | **Receiver Gain** | 32768.00 |
| **SW(cyclical) (Hz)** | 24038.46 | **Solvent** | CHLOROFORM-d | | | **Spectrum Offset (Hz)** | 10055.3965 |
| **Sweep Width (Hz)** | 24037.00 | **Temperature (degree C)** | 21.900 | | | | |

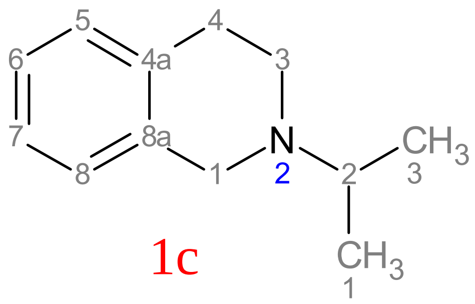


1c

fz6800-13_013000fid

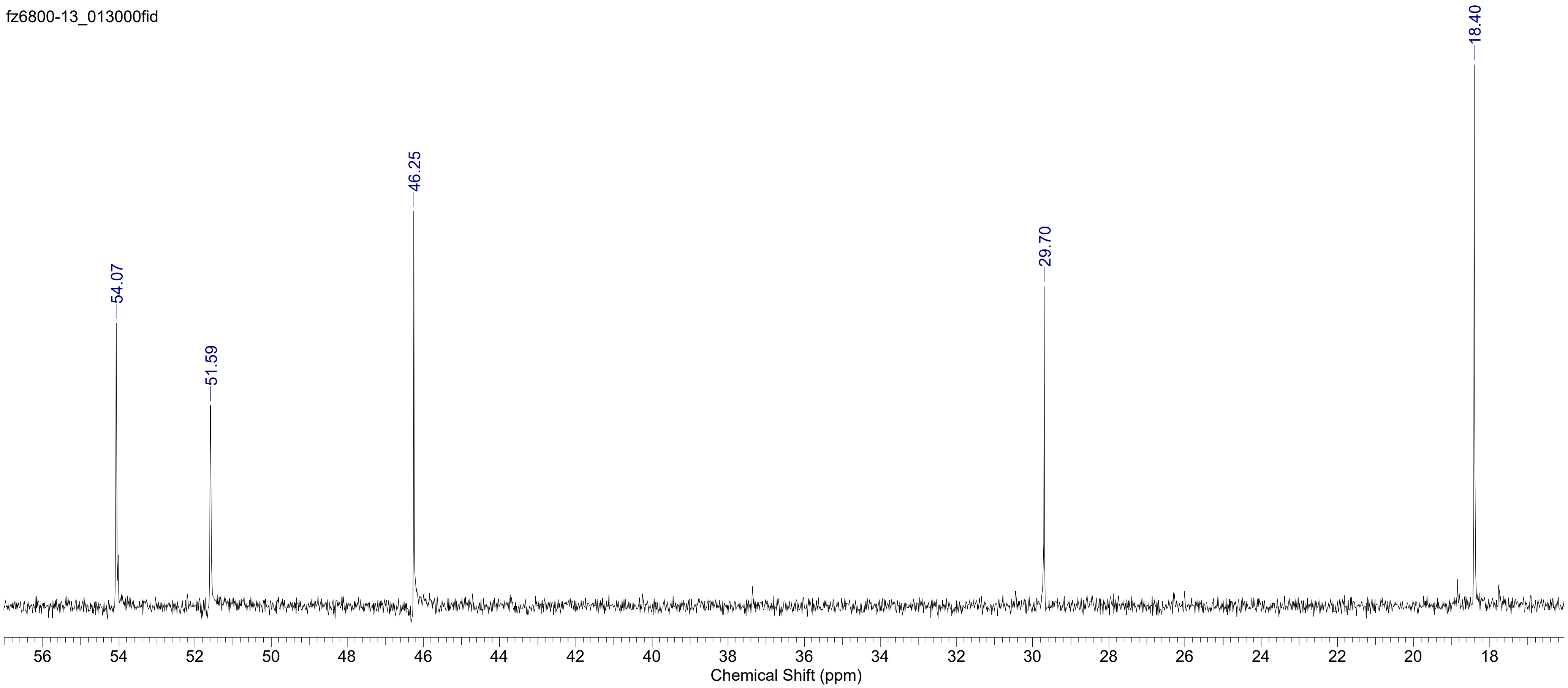

| | | | | | | | | | |
|---|---|---|---|---|---|---|---|---|---|
| ***Acquisition Time (sec)*** | 1.9818 | ***Comment*** | single_pulse | ***Date*** | 09 Apr 1990 03:59:35 | | | ***Date Stamp*** | 05 Apr 2018 08:47:02 |
| ***File Name*** | C:\Users\Fedor\Desktop\03.04.18\FZ6590-1.jdf | | | ***Frequency (MHz)*** | 600.17 | ***Nucleus*** | 1H | ***Number of Transients*** | 8 |
| ***Origin*** | ECA 600 | ***Original Points Count*** | 32768 | ***Owner*** | delta | ***Points Count*** | 32768 | ***Pulse Sequence*** | single_pulse.ex2 |
| ***Receiver Gain*** | 28.00 | ***Solvent*** | DMSO-d6 | ***Spectrum Offset (Hz)*** | 5401.5503 | ***Sweep Width (Hz)*** | 16534.39 | ***Temperature (degree C)*** | 21.700 |

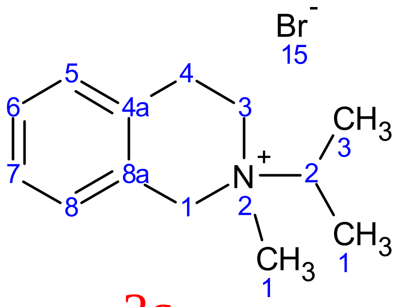


3c

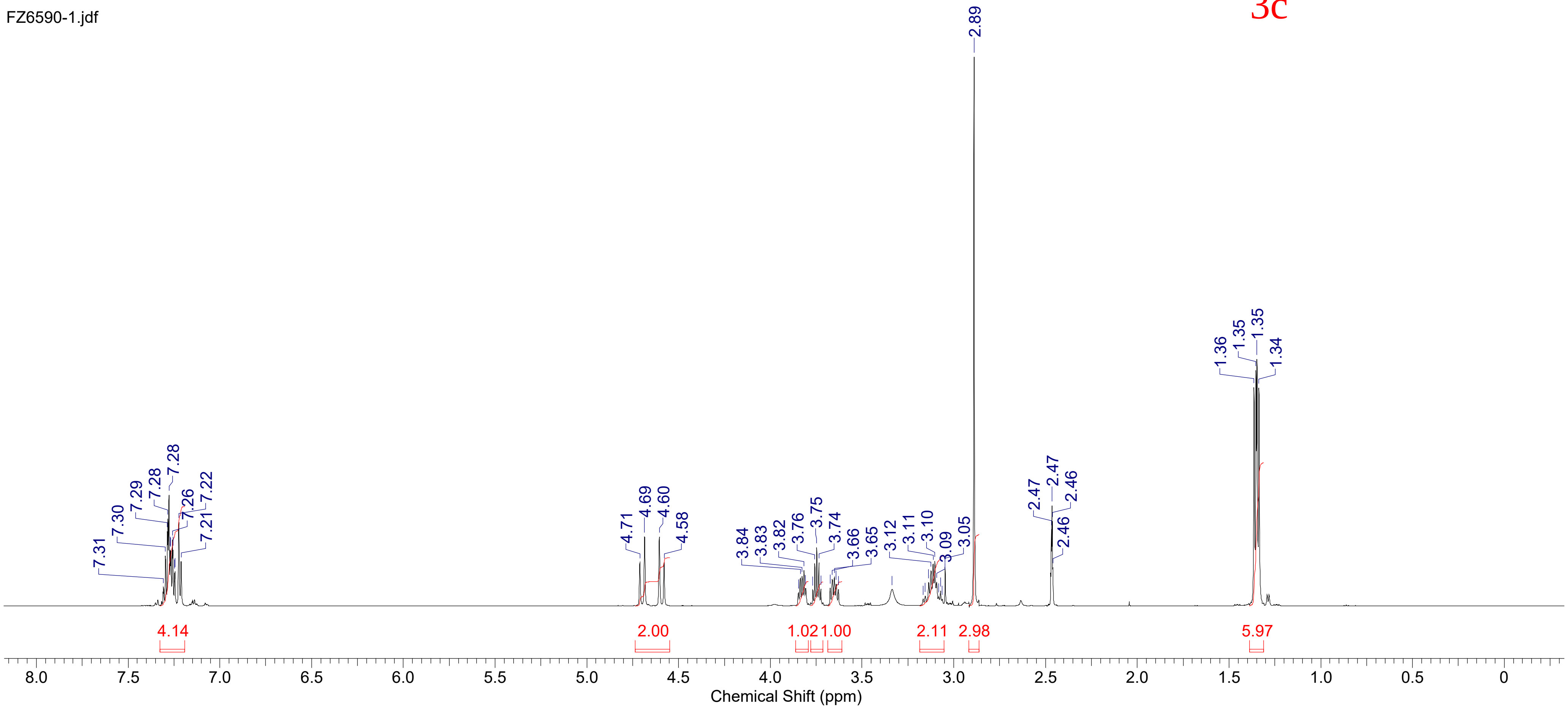

| Acquisition Time (sec) | 1.9818 | Comment | single_pulse | Date | 09 Apr 1990 03:59:35 | | | Date Stamp | 05 Apr 2018 08:47:02 |
|---|---|---|---|---|---|---|---|---|---|
| File Name | C:\Users\Fedor\Desktop\03.04.18\FZ6590-1.jdf | | | Frequency (MHz) | 600.17 | Nucleus | 1H | Number of Transients | 8 |
| Origin | ECA 600 | Original Points Count | 32768 | Owner | delta | Points Count | 32768 | Pulse Sequence | single_pulse.ex2 |
| Receiver Gain | 28.00 | Solvent | DMSO-d6 | Spectrum Offset (Hz) | 5401.5503 | Sweep Width (Hz) | 16534.39 | Temperature (degree C) | 21.700 |

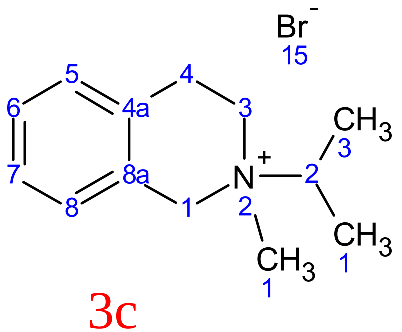


3c

FZ6590-1.jdf

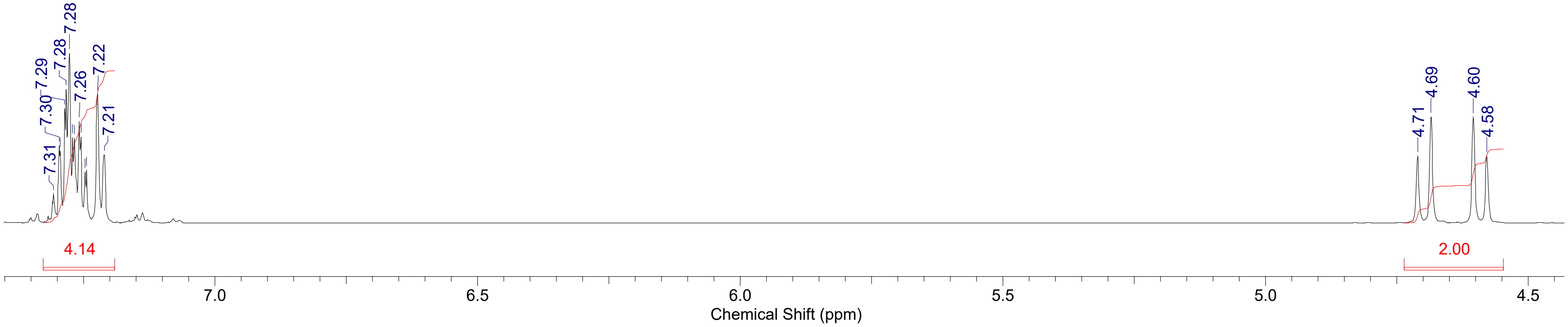

| | | | | | | | | | |
|---|---|---|---|---|---|---|---|---|---|
| ***Acquisition Time (sec)*** | 1.9818 | ***Comment*** | single_pulse | ***Date*** | 09 Apr 1990 03:59:35 | | | ***Date Stamp*** | 05 Apr 2018 08:47:02 |
| ***File Name*** | C:\Users\Fedor\Desktop\03.04.18\FZ6590-1.jdf | | | ***Frequency (MHz)*** | 600.17 | ***Nucleus*** | 1H | ***Number of Transients*** | 8 |
| ***Origin*** | ECA 600 | ***Original Points Count*** | 32768 | ***Owner*** | delta | ***Points Count*** | 32768 | ***Pulse Sequence*** | single_pulse.ex2 |
| ***Receiver Gain*** | 28.00 | ***Solvent*** | DMSO-d6 | ***Spectrum Offset (Hz)*** | 5401.5503 | ***Sweep Width (Hz)*** | 16534.39 | ***Temperature (degree C)*** | 21.700 |

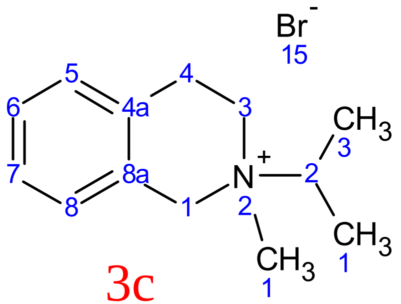


3c

FZ6590-1.jdf

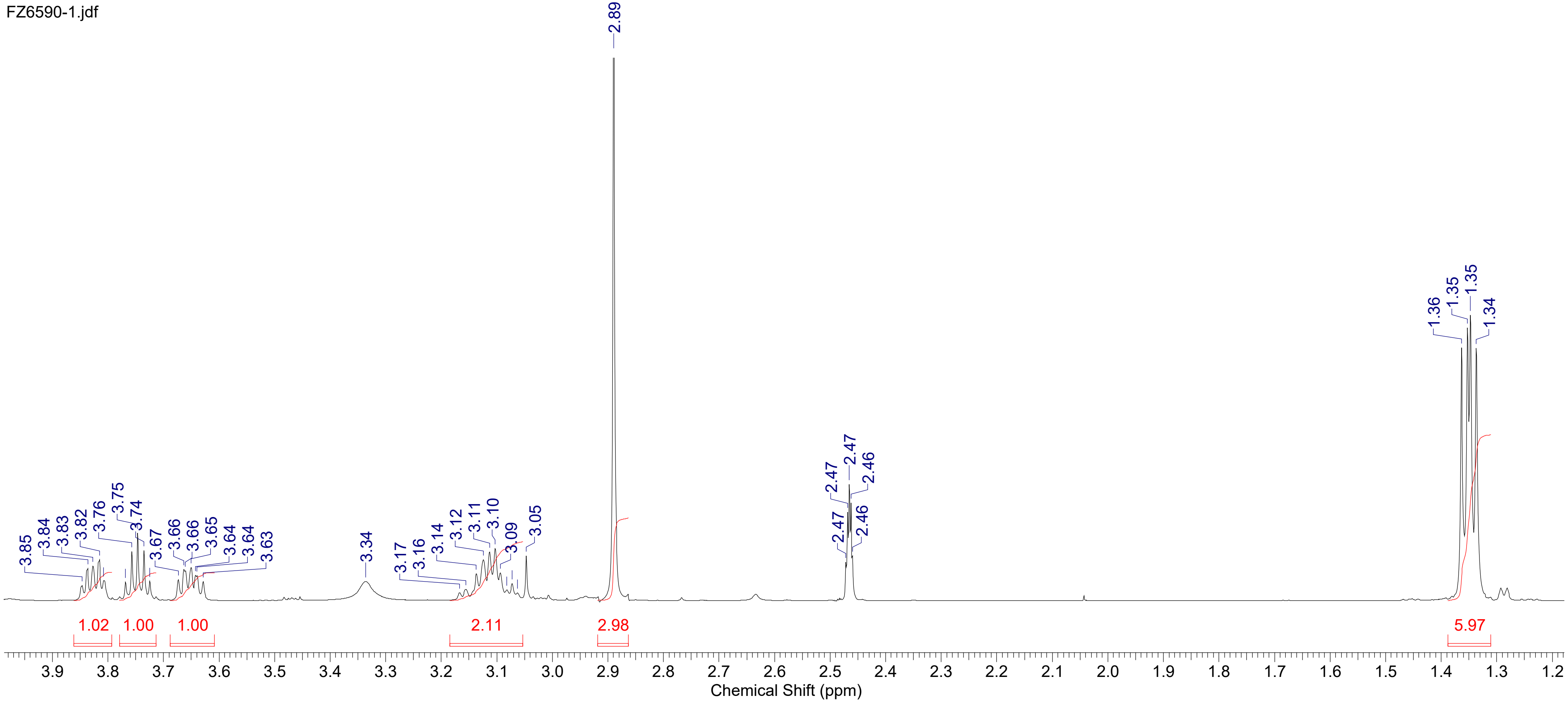

| | | | | | | | |
|---|---|---|---|---|---|---|---|
| ***Acquisition Time (sec)*** | 0.6921 | ***Comment*** | single pulse decoupled gated NOE | | | ***Date*** | 13 Apr 1990 17:28:35 |
| ***Date Stamp*** | 10 Apr 2018 04:03:52 | | | ***File Name*** | C:\Users\Fedor\Desktop\03.04.18\FZ6590-2.jdf | | |
| ***Frequency (MHz)*** | 150.91 | ***Nucleus*** | 13C | ***Number of Transients*** | 2000 | ***Origin*** | ECA 600 |
| ***Original Points Count*** | 32768 | ***Owner*** | delta | ***Points Count*** | 32768 | ***Pulse Sequence*** | single_pulse_dec |
| ***Receiver Gain*** | 54.00 | ***Solvent*** | DMSO-d6 | ***Spectrum Offset (Hz)*** | 15091.3428 | ***Sweep Width (Hz)*** | 47348.49 |
| ***Temperature (degree C)*** | 22.500 | | | | | | |

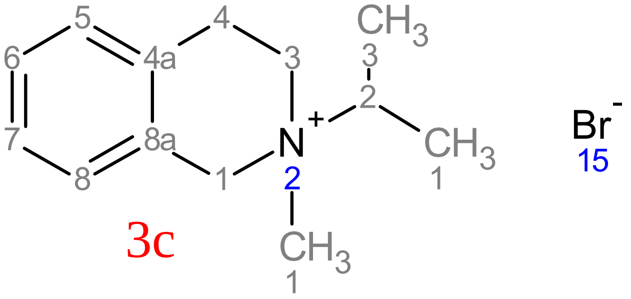


3c

FZ6590-2.jdf

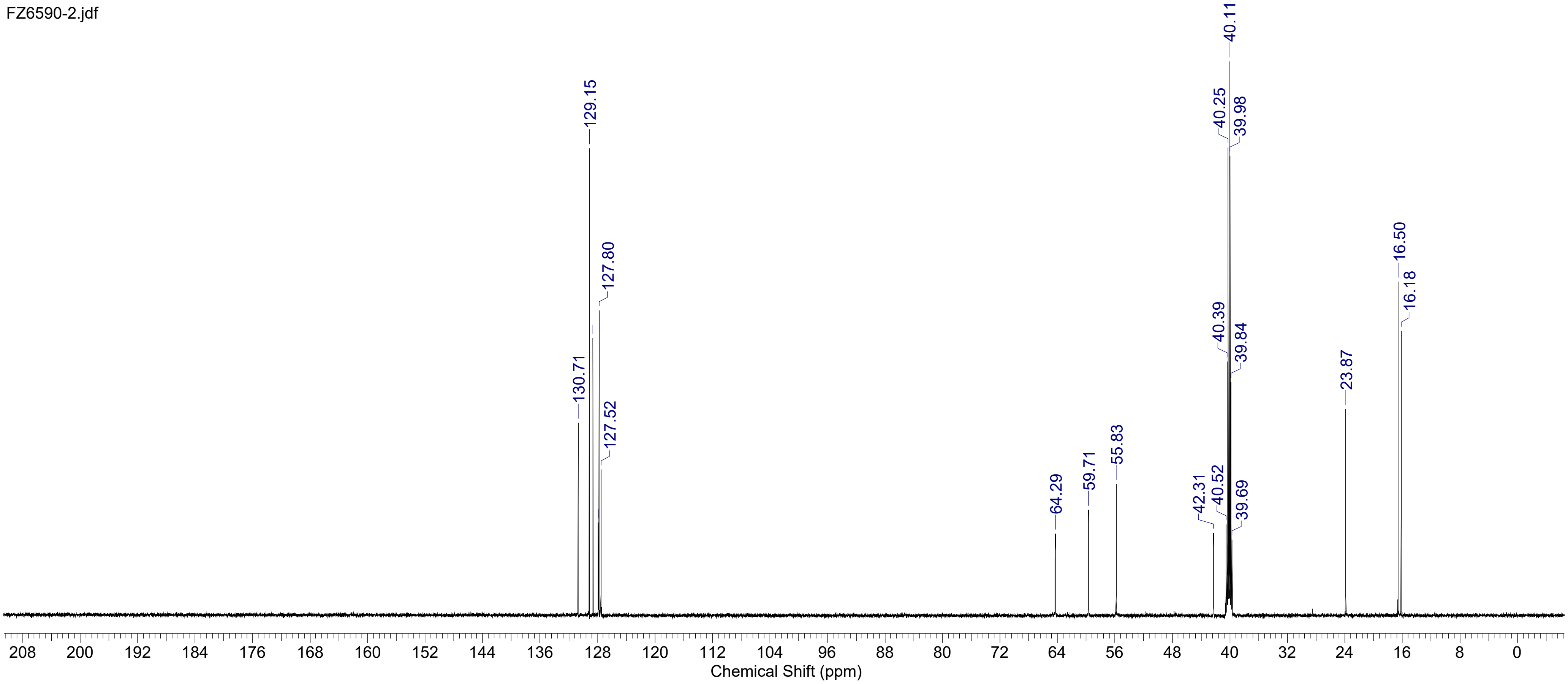

| | | | | | | | |
|---|---|---|---|---|---|---|---|
| ***Acquisition Time (sec)*** | 0.6921 | ***Comment*** | single pulse decoupled gated NOE | | | ***Date*** | 13 Apr 1990 17:28:35 |
| ***Date Stamp*** | 10 Apr 2018 04:03:52 | | | ***File Name*** | C:\Users\Fedor\Desktop\03.04.18\FZ6590-2.jdf | | |
| ***Frequency (MHz)*** | 150.91 | ***Nucleus*** | 13C | ***Number of Transients*** | 2000 | ***Origin*** | ECA 600 |
| ***Original Points Count*** | 32768 | ***Owner*** | delta | ***Points Count*** | 32768 | ***Pulse Sequence*** | single_pulse_dec |
| ***Receiver Gain*** | 54.00 | ***Solvent*** | DMSO-d6 | ***Spectrum Offset (Hz)*** | 15091.3428 | ***Sweep Width (Hz)*** | 47348.49 |
| ***Temperature (degree C)*** | 22.500 | | | | | | |

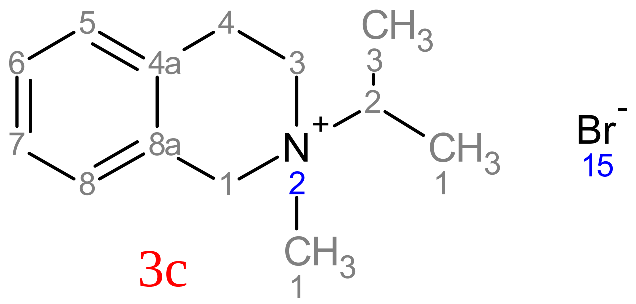


3c

FZ6590-2.jdf

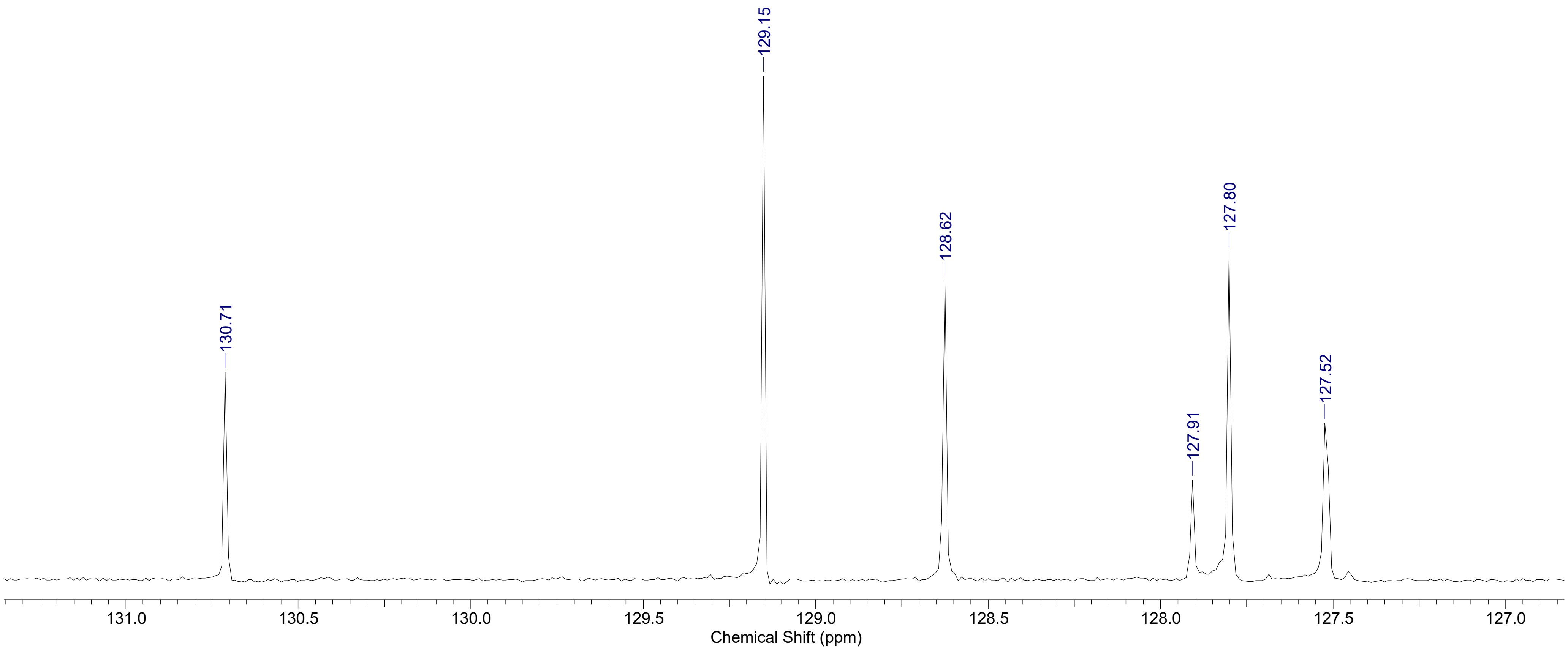

| | | | | | | | |
|---|---|---|---|---|---|---|---|
| **Acquisition Time (sec)** | 0.6921 | **Comment** | single pulse decoupled gated NOE | | | **Date** | 13 Apr 1990 17:28:35 |
| **Date Stamp** | 10 Apr 2018 04:03:52 | | | **File Name** | C:\Users\Fedor\Desktop\03.04.18\FZ6590-2.jdf | | |
| **Frequency (MHz)** | 150.91 | **Nucleus** | 13C | **Number of Transients** | 2000 | **Origin** | ECA 600 |
| **Original Points Count** | 32768 | **Owner** | delta | **Points Count** | 32768 | **Pulse Sequence** | single_pulse_dec |
| **Receiver Gain** | 54.00 | **Solvent** | DMSO-d6 | **Spectrum Offset (Hz)** | 15091.3428 | **Sweep Width (Hz)** | 47348.49 |
| **Temperature (degree C)** | 22.500 | | | | | | |

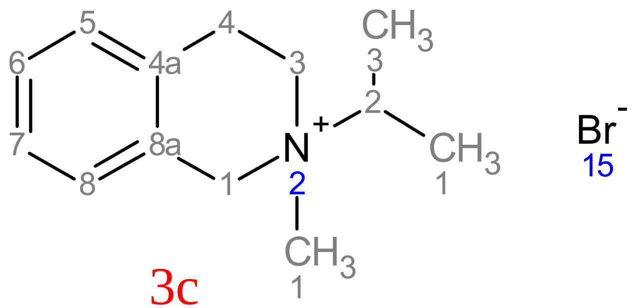


3c

FZ6590-2.jdf

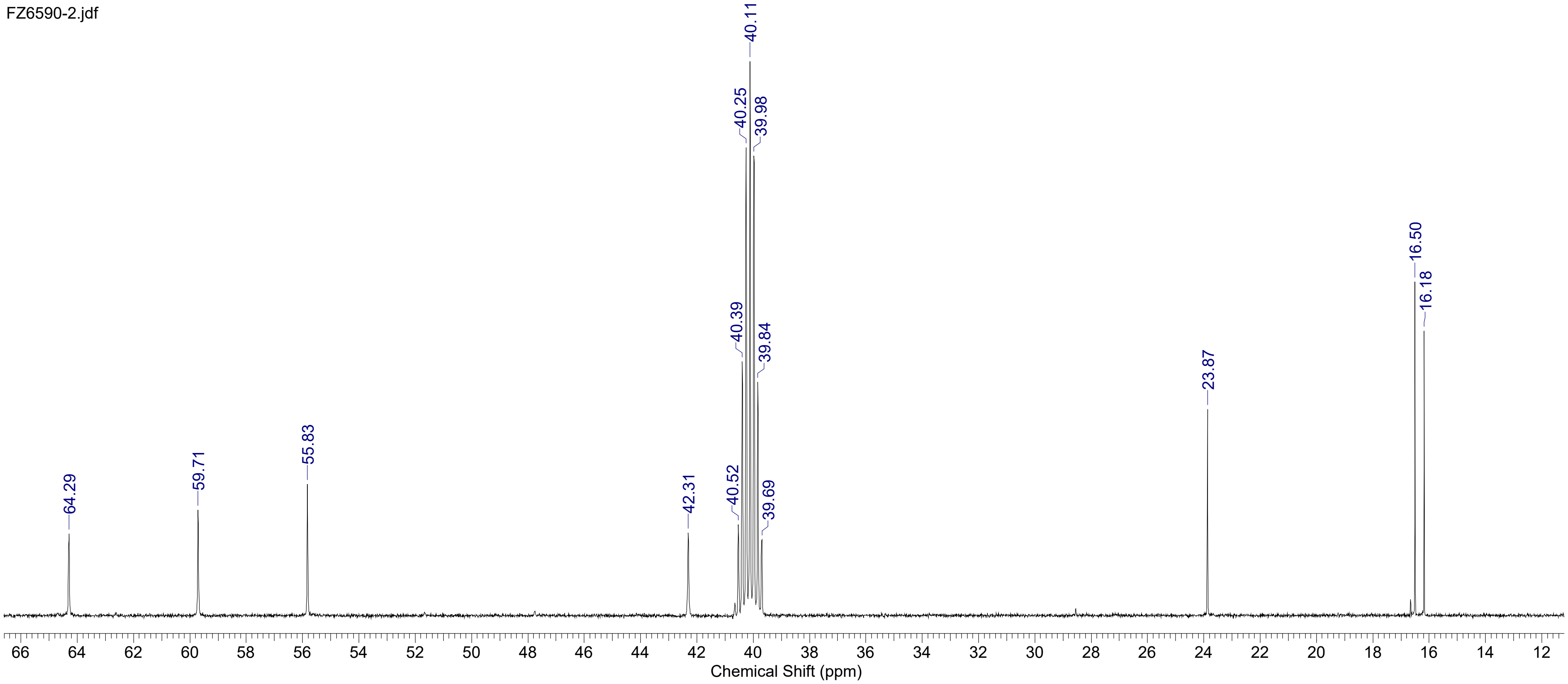

| | | | | | | | |
|---|---|---|---|---|---|---|---|
| *Acquisition Time (sec)* | 1.9818 | *Comment* | single_pulse | *Date* | 22 Jul 1990 10:49:20 | | |
| *Date Stamp* | 13 Dec 2017 13:24:28 | | | *File Name* | C:\Users\Fedor\Desktop\12.12.17\FZ6278-1.jdf | *Frequency (MHz)* | 600.17 |
| *Nucleus* | 1H | *Number of Transients* | 8 | *Origin* | ECA 600 | *Original Points Count* | 32768 |
| *Owner* | delta | | | | | | |
| *Points Count* | 32768 | *Pulse Sequence* | single_pulse.ex2 | *Receiver Gain* | 34.00 | *Solvent* | CHLOROFORM-d |
| *Spectrum Offset (Hz)* | 5401.5503 | *Sweep Width (Hz)* | 16534.39 | *Temperature (degree C)* | 19.400 | | |

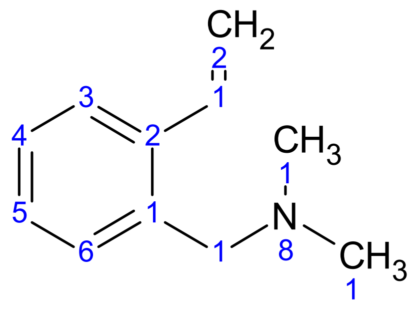

4a

FZ6278-1.jdf

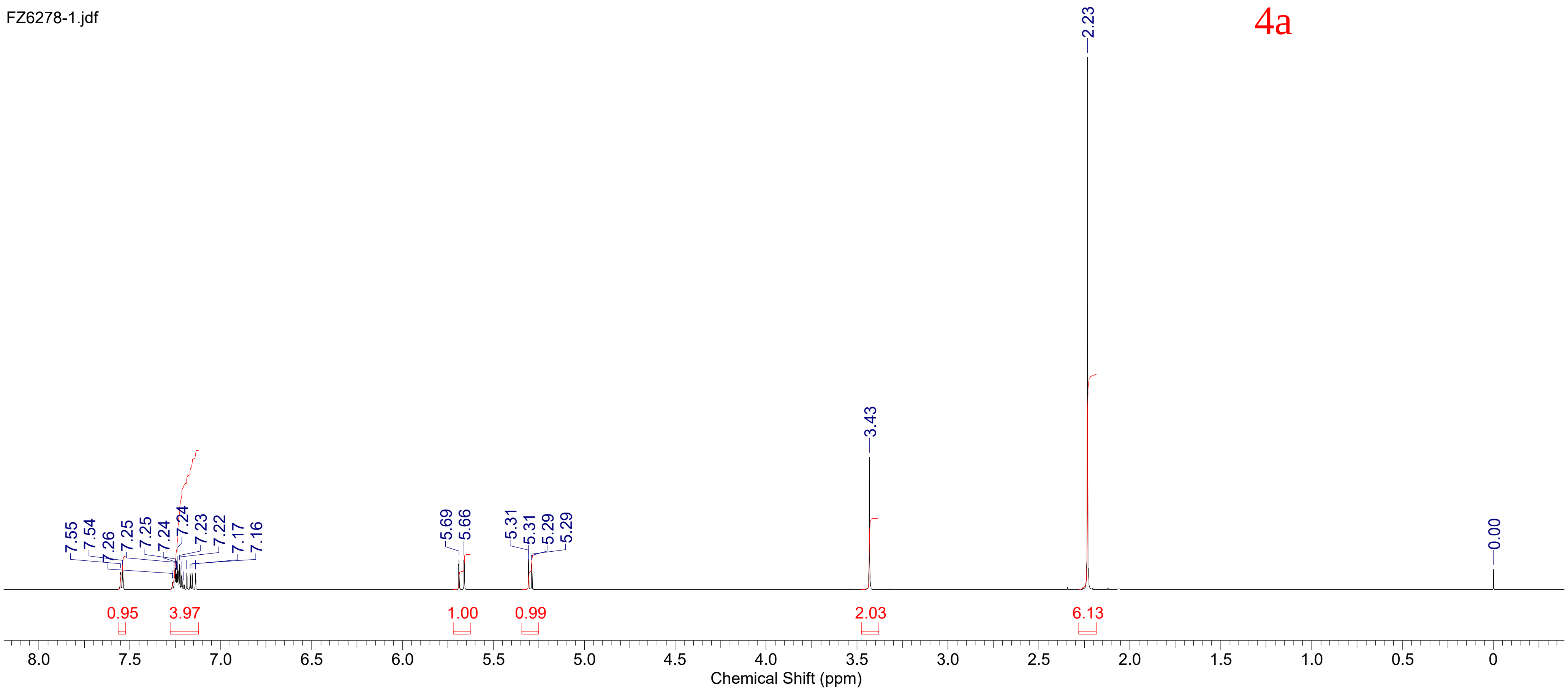

| | | | | | | | |
|---|---|---|---|---|---|---|---|
| ***Acquisition Time (sec)*** | 1.9818 | ***Comment*** | single_pulse | ***Date*** | 22 Jul 1990 10:49:20 | | |
| ***Date Stamp*** | 13 Dec 2017 13:24:28 | | | ***File Name*** | C:\Users\Fedor\Desktop\12.12.17\FZ6278-1.jdf | ***Frequency (MHz)*** | 600.17 |
| ***Nucleus*** | 1H | ***Number of Transients*** | 8 | ***Origin*** | ECA 600 | ***Original Points Count*** | 32768 |
| ***Owner*** | delta | | | | | | |
| ***Points Count*** | 32768 | ***Pulse Sequence*** | single_pulse.ex2 | ***Receiver Gain*** | 34.00 | ***Solvent*** | CHLOROFORM-d |
| ***Spectrum Offset (Hz)*** | 5401.5503 | ***Sweep Width (Hz)*** | 16534.39 | ***Temperature (degree C)*** | 19.400 | | |

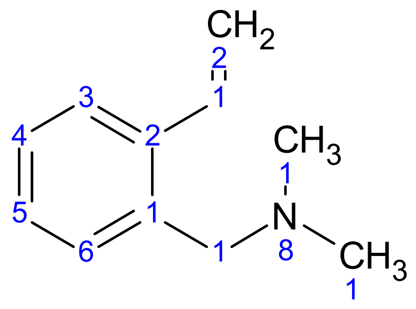


4a

FZ6278-1.jdf

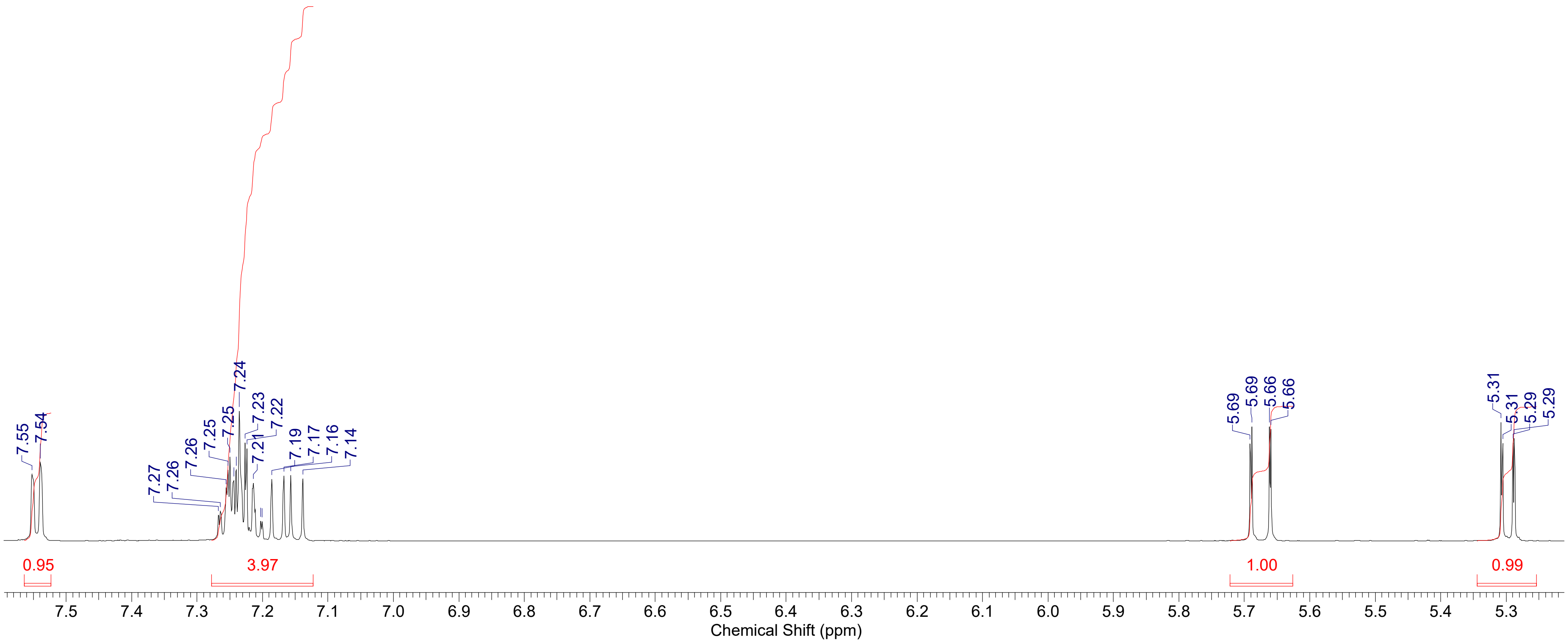

| | | | | | | | |
|---|---|---|---|---|---|---|---|
| ***Acquisition Time (sec)*** | 1.9818 | ***Comment*** | single_pulse | ***Date*** | 22 Jul 1990 10:49:20 | | |
| ***Date Stamp*** | 13 Dec 2017 13:24:28 | | | ***File Name*** | C:\Users\Fedor\Desktop\12.12.17\FZ6278-1.jdf | ***Frequency (MHz)*** | 600.17 |
| ***Nucleus*** | 1H | ***Number of Transients*** | 8 | ***Origin*** | ECA 600 | ***Original Points Count*** 32768 | ***Owner*** delta |
| ***Points Count*** | 32768 | ***Pulse Sequence*** | single_pulse.ex2 | | | ***Receiver Gain*** 34.00 | ***Solvent*** CHLOROFORM-d |
| ***Spectrum Offset (Hz)*** | 5401.5503 | ***Sweep Width (Hz)*** | 16534.39 | ***Temperature (degree C)*** | 19.400 | | |

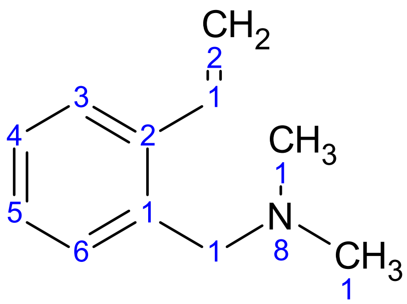

4a

FZ6278-1.jdf

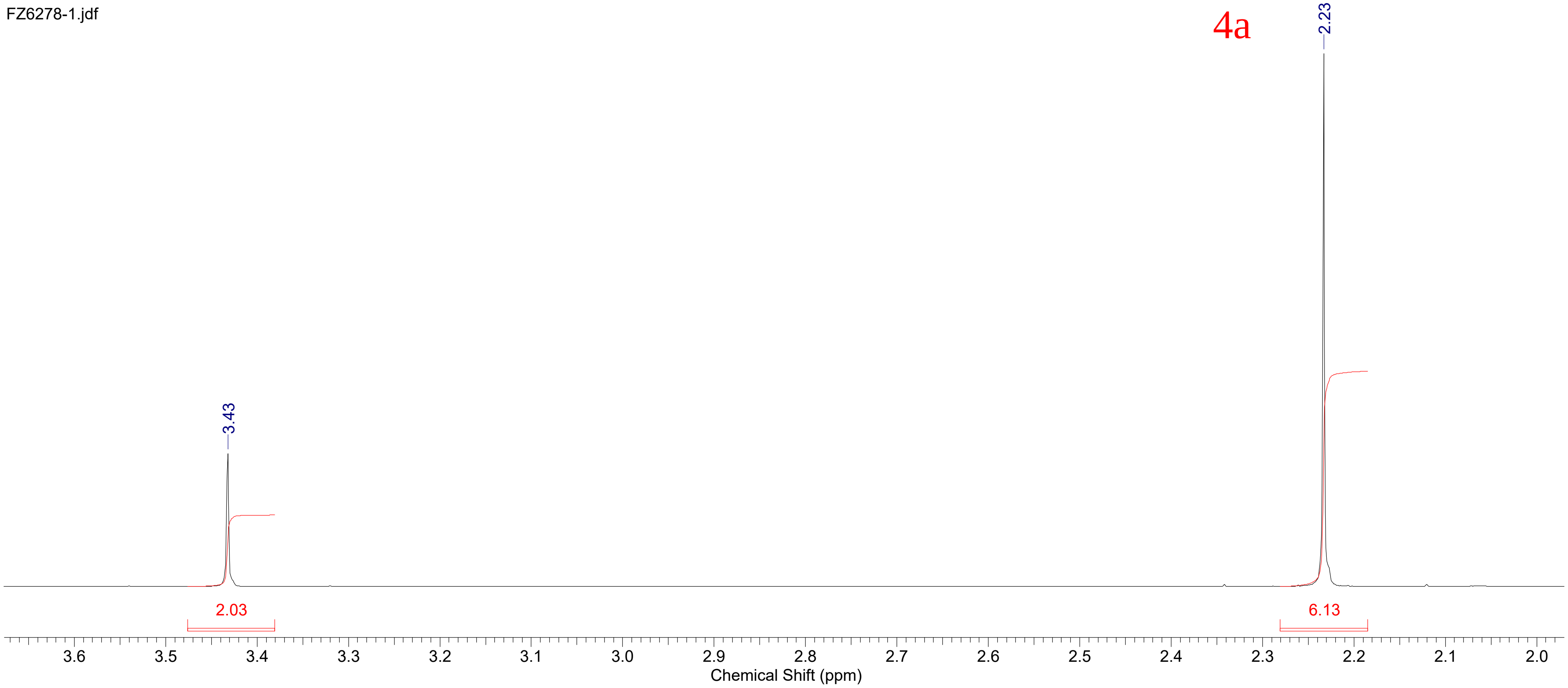

| | | | | | | | |
|---|---|---|---|---|---|---|---|
| ***Acquisition Time (sec)*** | 0.6921 | ***Comment*** | single pulse decoupled gated NOE | | | ***Date*** | 22 Apr 1990 08:17:52 |
| ***Date Stamp*** | 18 Apr 2018 13:05:31 | | | ***File Name*** | C:\Users\Fedor\Desktop\17.04.18\FZ6620-1.jdf | | |
| ***Frequency (MHz)*** | 150.91 | ***Nucleus*** | 13C | ***Number of Transients*** | 235 | ***Origin*** | ECA 600 |
| ***Original Points Count*** | 32768 | ***Owner*** | delta | ***Points Count*** | 32768 | ***Pulse Sequence*** | single_pulse_dec |
| ***Receiver Gain*** | 52.00 | ***Solvent*** | CHLOROFORM-d | | | ***Spectrum Offset (Hz)*** | 15091.3428 |
| ***Sweep Width (Hz)*** | 47348.49 | ***Temperature (degree C)*** | 23.200 | | | | |

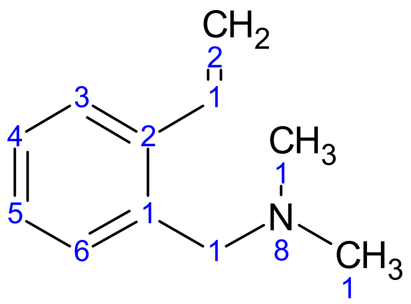

4a

FZ6620-1.jdf

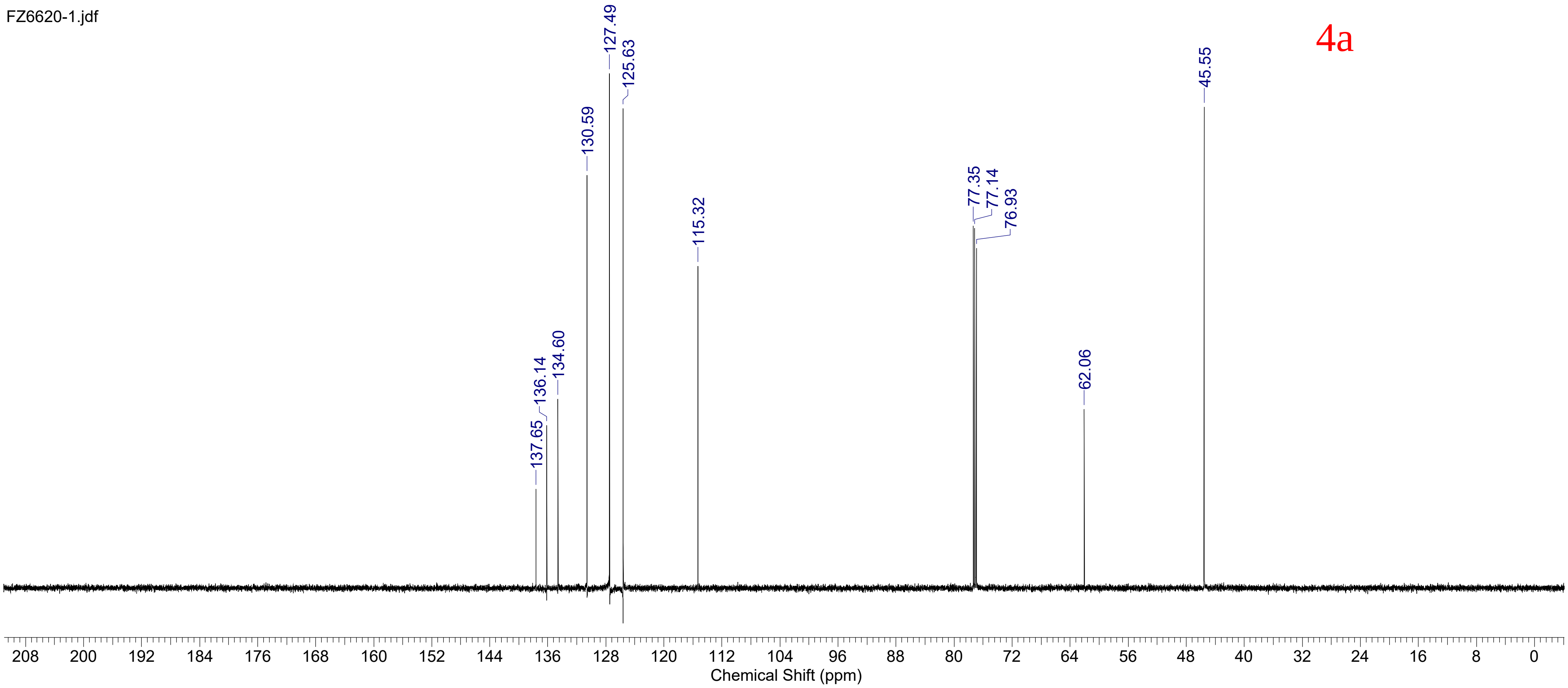

| Acquisition Time (sec) | 0.6921 | Comment | single pulse decoupled gated NOE | | | Date | 22 Apr 1990 08:17:52 |
|---|---|---|---|---|---|---|---|
| Date Stamp | 18 Apr 2018 13:05:31 | | | File Name | C:\Users\Fedor\Desktop\17.04.18\FZ6620-1.jdf | | |
| Frequency (MHz) | 150.91 | Nucleus | 13C | Number of Transients | 235 | Origin | ECA 600 |
| Original Points Count | 32768 | Owner | delta | Points Count | 32768 | Pulse Sequence | single_pulse_dec |
| Receiver Gain | 52.00 | Solvent | CHLOROFORM-d | | | Spectrum Offset (Hz) | 15091.3428 |
| Sweep Width (Hz) | 47348.49 | Temperature (degree C) | 23.200 | | | | |

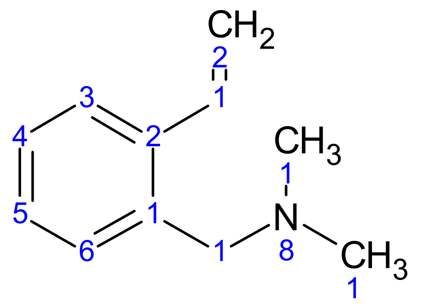


4a

FZ6620-1.jdf

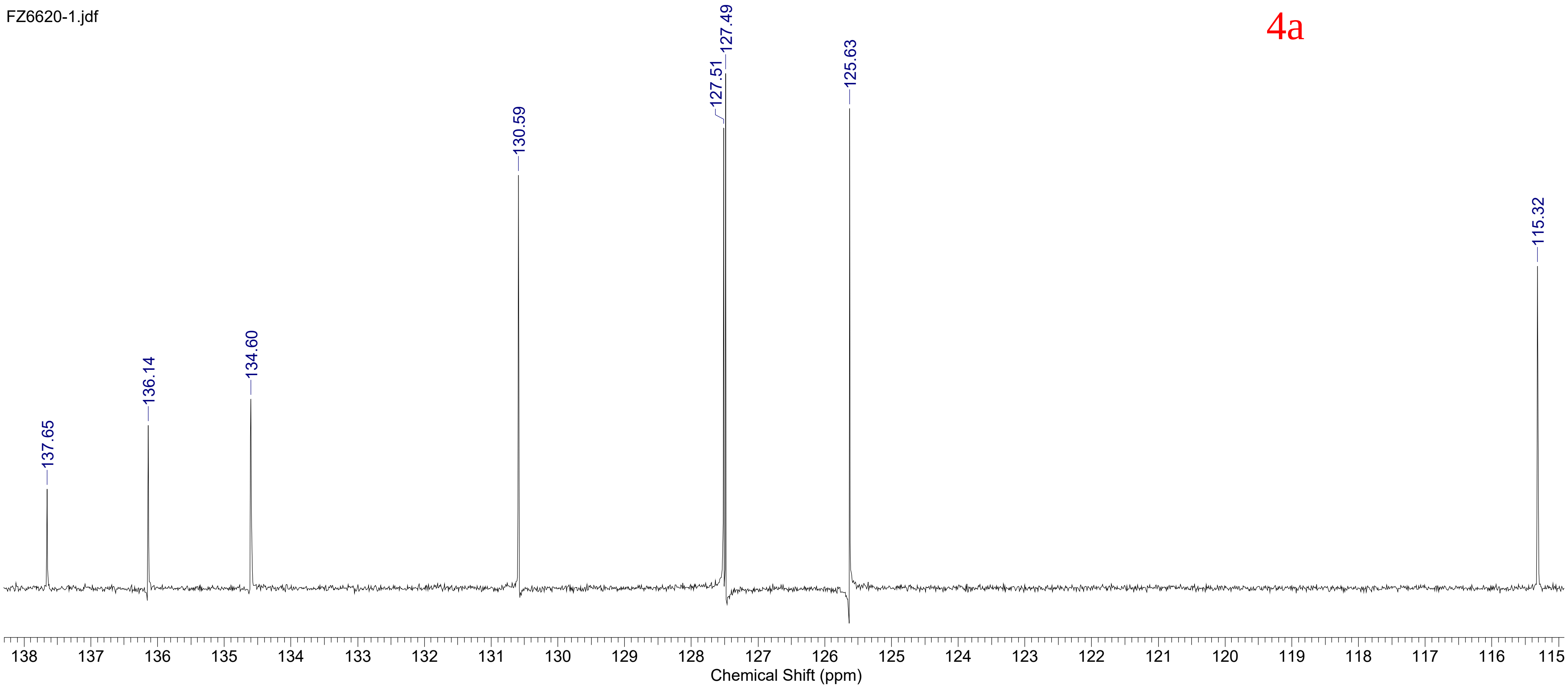

| | | | | | | | |
|---|---|---|---|---|---|---|---|
| ***Acquisition Time (sec)*** | 0.6921 | ***Comment*** | single pulse decoupled gated NOE | | | ***Date*** | 22 Apr 1990 08:17:52 |
| ***Date Stamp*** | 18 Apr 2018 13:05:31 | | | ***File Name*** | C:\Users\Fedor\Desktop\17.04.18\FZ6620-1.jdf | | |
| ***Frequency (MHz)*** | 150.91 | ***Nucleus*** | 13C | ***Number of Transients*** | 235 | ***Origin*** | ECA 600 |
| ***Original Points Count*** | 32768 | ***Owner*** | delta | ***Points Count*** | 32768 | ***Pulse Sequence*** | single_pulse_dec |
| ***Receiver Gain*** | 52.00 | ***Solvent*** | CHLOROFORM-d | | | ***Spectrum Offset (Hz)*** | 15091.3428 |
| ***Sweep Width (Hz)*** | 47348.49 | ***Temperature (degree C)*** | 23.200 | | | | |

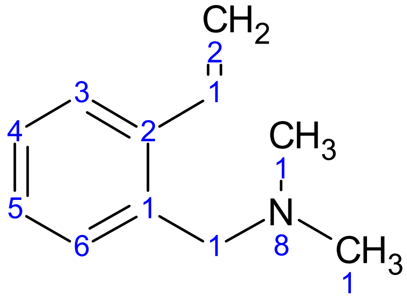


4a

FZ6620-1.jdf

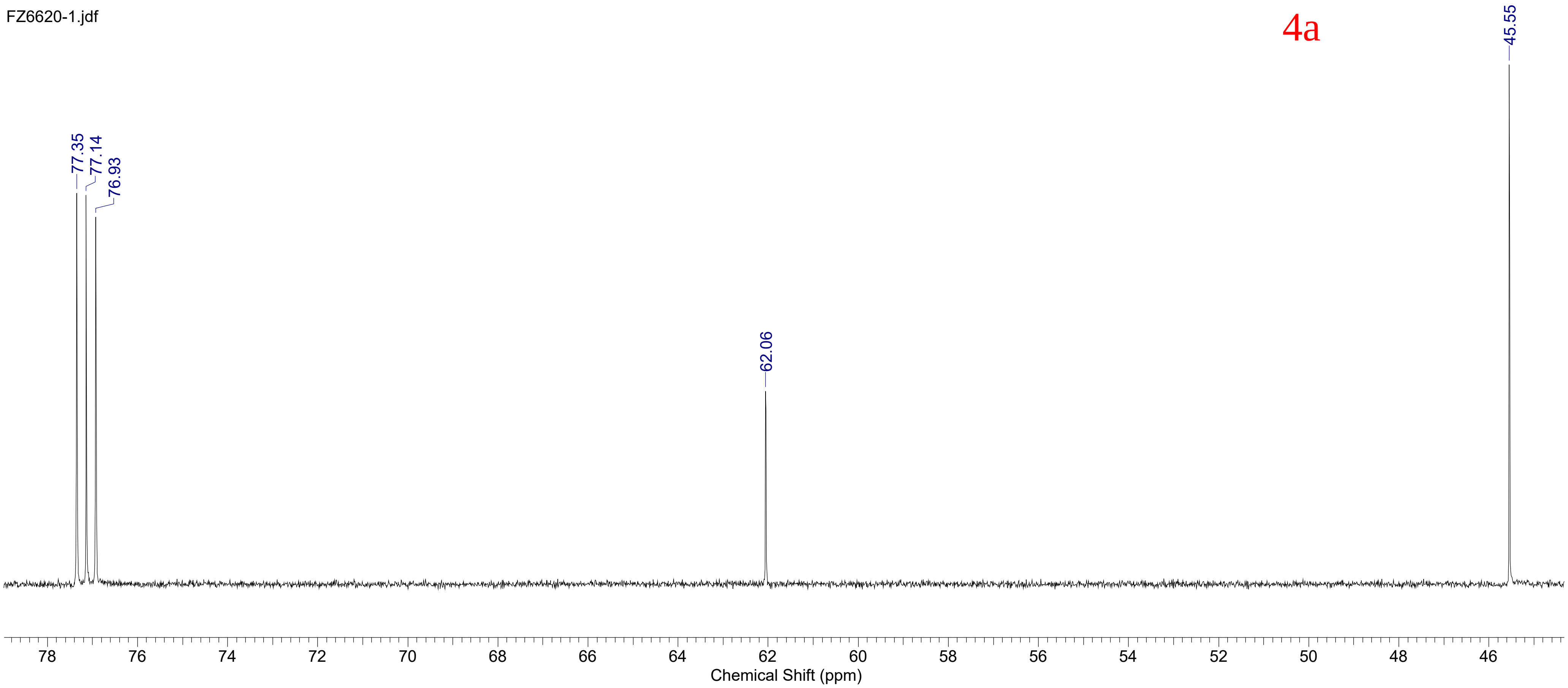

| | | | | | | | |
|---|---|---|---|---|---|---|---|
| ***Acquisition Time (sec)*** | 1.9818 | ***Comment*** | single_pulse | ***Date*** | 11 Mar 1990 03:06:05 | | |
| ***Date Stamp*** | 07 Mar 2018 07:53:44 | | | ***File Name*** | C:\Users\Fedor\Desktop\06.03.18\FZ6486-1.jdf | ***Frequency (MHz)*** | 600.17 |
| ***Nucleus*** | 1H | ***Number of Transients*** | 8 | ***Origin*** | ECA 600 | ***Original Points Count*** | 32768 |
| ***Owner*** | delta | | | | | | |
| ***Points Count*** | 32768 | ***Pulse Sequence*** | single_pulse.ex2 | ***Receiver Gain*** | 28.00 | ***Solvent*** | CHLOROFORM-d |
| ***Spectrum Offset (Hz)*** | 5393.9751 | ***Sweep Width (Hz)*** | 16534.39 | ***Temperature (degree C)*** | 22.200 | | |

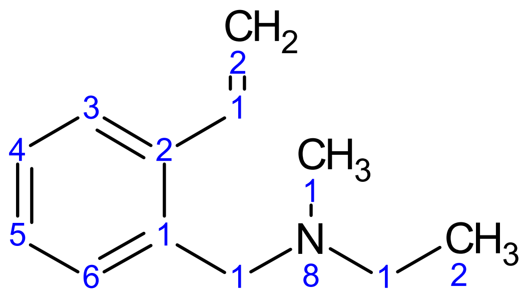


4b

FZ6486-1.jdf

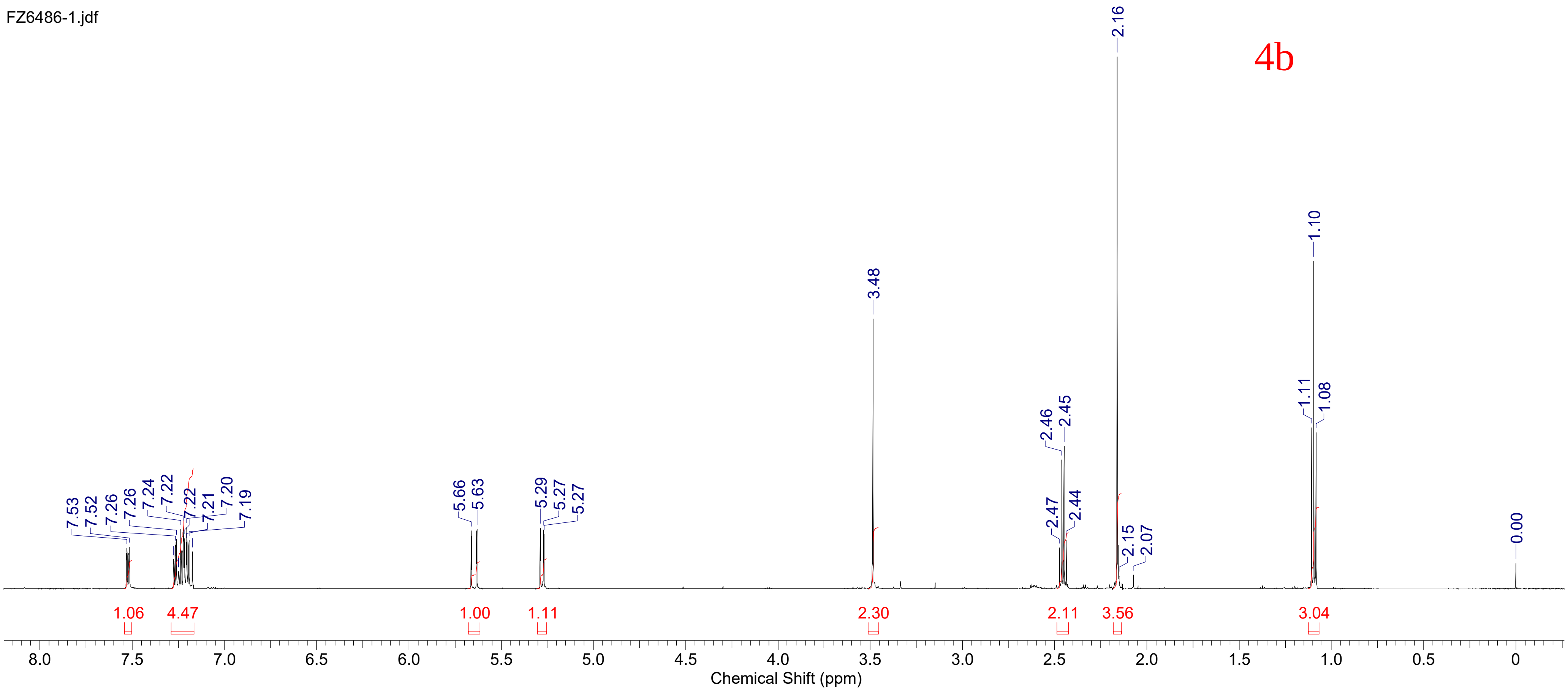

| | | | | | | | |
|---|---|---|---|---|---|---|---|
| ***Acquisition Time (sec)*** | 1.9818 | ***Comment*** | single_pulse | ***Date*** | 11 Mar 1990 03:06:05 | | |
| ***Date Stamp*** | 07 Mar 2018 07:53:44 | | | ***File Name*** | C:\Users\Fedor\Desktop\06.03.18\FZ6486-1.jdf | ***Frequency (MHz)*** | 600.17 |
| ***Nucleus*** | 1H | ***Number of Transients*** | 8 | ***Origin*** | ECA 600 | ***Original Points Count*** 32768 | ***Owner*** delta |
| ***Points Count*** | 32768 | ***Pulse Sequence*** | single_pulse.ex2 | | | ***Receiver Gain*** 28.00 | ***Solvent*** CHLOROFORM-d |
| ***Spectrum Offset (Hz)*** | 5393.9751 | ***Sweep Width (Hz)*** | 16534.39 | ***Temperature (degree C)*** | 22.200 | | |

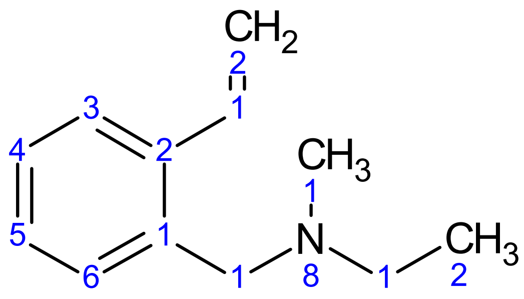

4b

FZ6486-1.jdf

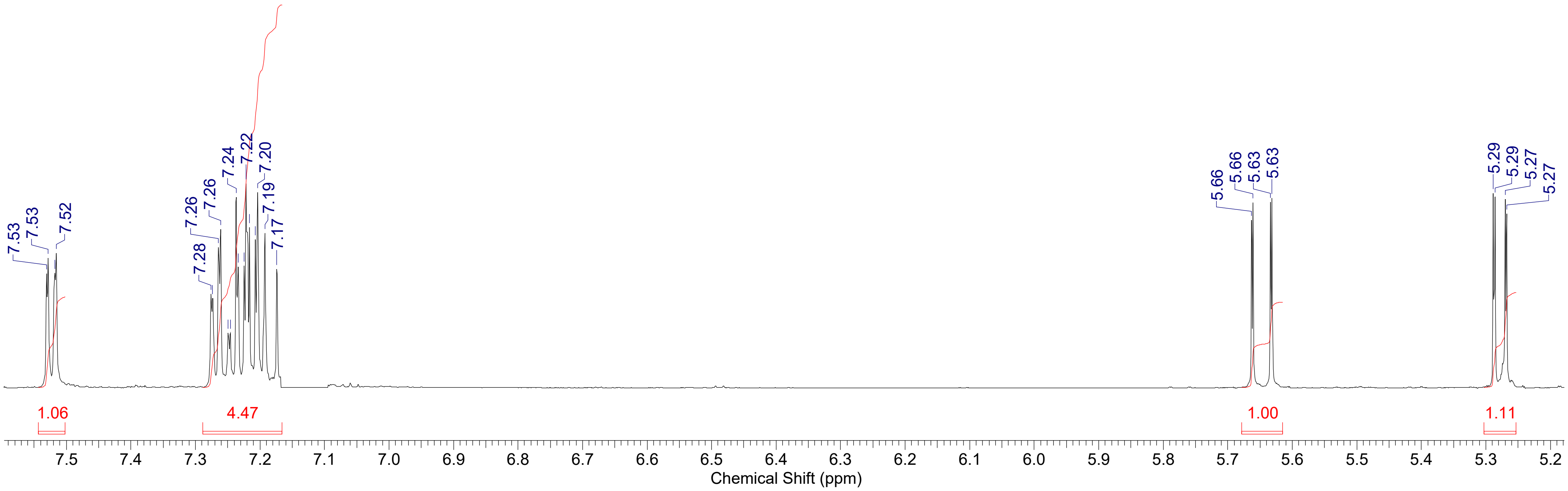

| | | | | | | | |
|---|---|---|---|---|---|---|---|
| ***Acquisition Time (sec)*** | 1.9818 | ***Comment*** | single_pulse | ***Date*** | 11 Mar 1990 03:06:05 | | |
| ***Date Stamp*** | 07 Mar 2018 07:53:44 | | | ***File Name*** | C:\Users\Fedor\Desktop\06.03.18\FZ6486-1.jdf | ***Frequency (MHz)*** | 600.17 |
| ***Nucleus*** | 1H | ***Number of Transients*** | 8 | ***Origin*** | ECA 600 | ***Original Points Count*** 32768 | ***Owner*** delta |
| ***Points Count*** | 32768 | ***Pulse Sequence*** | single_pulse.ex2 | | | ***Receiver Gain*** 28.00 | ***Solvent*** CHLOROFORM-d |
| ***Spectrum Offset (Hz)*** | 5393.9751 | ***Sweep Width (Hz)*** | 16534.39 | ***Temperature (degree C)*** | 22.200 | | |

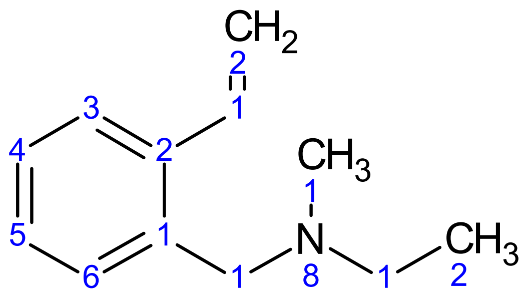


4b

FZ6486-1.jdf

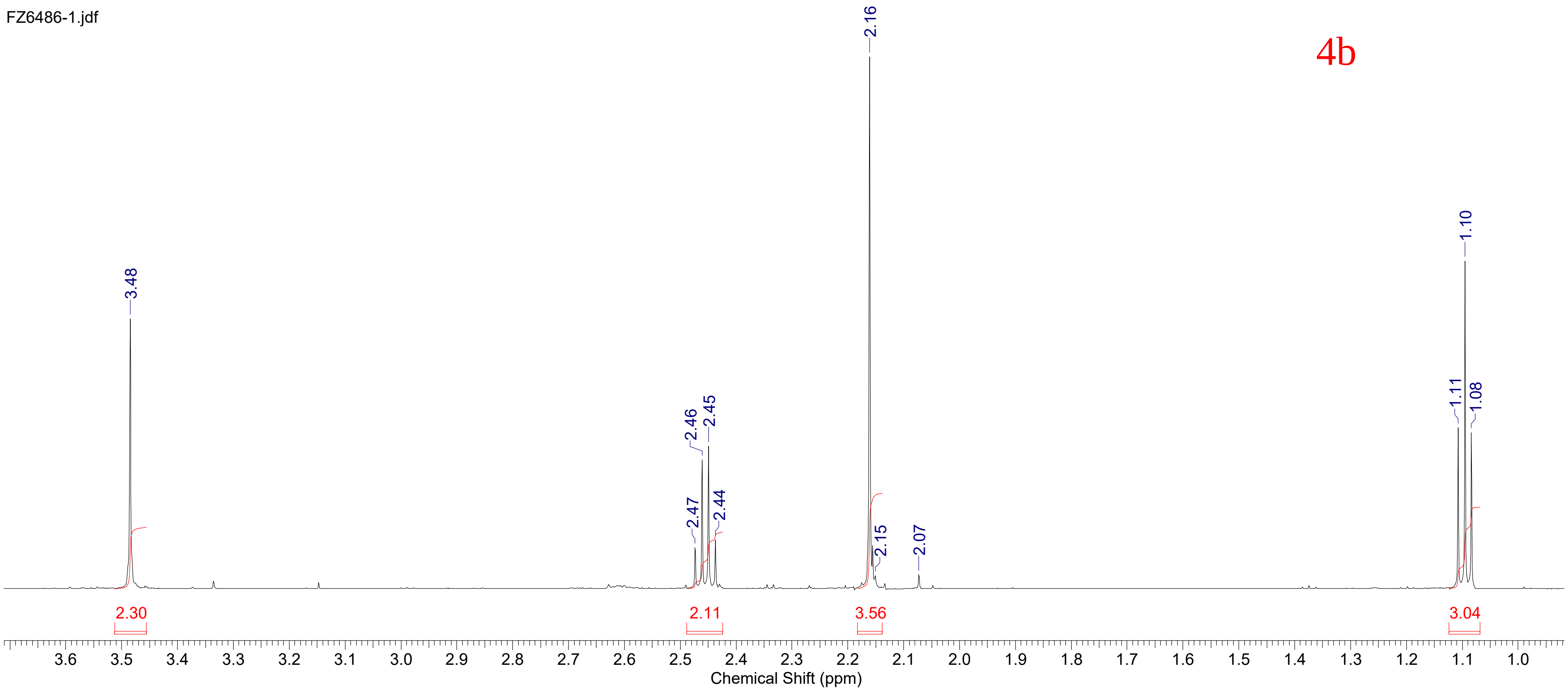

| | | | | | | | |
|---|---|---|---|---|---|---|---|
| ***Acquisition Time (sec)*** | 0.6921 | ***Comment*** | single pulse decoupled gated NOE | | | ***Date*** | 18 May 1990 05:49:40 |
| ***Date Stamp*** | 14 May 2018 10:36:25 | | | ***File Name*** | C:\Users\Fedor\Desktop\FZ6702-2.jdf | | |
| ***Frequency (MHz)*** | 150.91 | ***Nucleus*** | 13C | ***Number of Transients*** | 2000 | ***Origin*** | ECA 600 |
| ***Original Points Count*** | 32768 | ***Owner*** | delta | ***Points Count*** | 32768 | ***Pulse Sequence*** | single_pulse_dec |
| ***Receiver Gain*** | 52.00 | ***Solvent*** | CHLOROFORM-d | | | ***Spectrum Offset (Hz)*** | 15091.3428 |
| ***Sweep Width (Hz)*** | 47348.49 | ***Temperature (degree C)*** | 22.100 | | | | |

4b

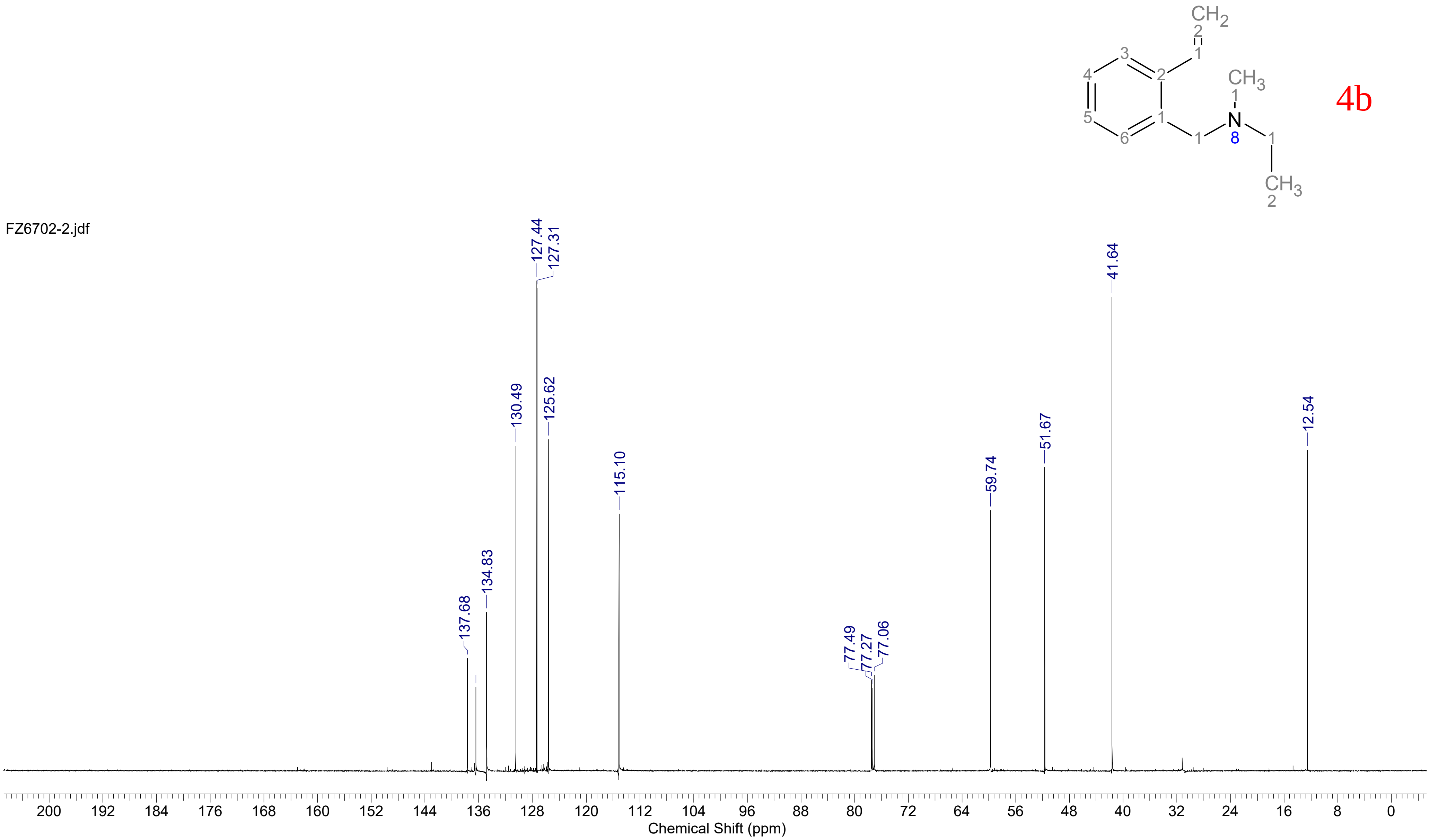

| | | | | | | | |
|---|---|---|---|---|---|---|---|
| ***Acquisition Time (sec)*** | 0.6921 | ***Comment*** | single pulse decoupled gated NOE | | | ***Date*** | 18 May 1990 05:49:40 |
| ***Date Stamp*** | 14 May 2018 10:36:25 | | | ***File Name*** | C:\Users\Fedor\Desktop\FZ6702-2.jdf | | |
| ***Frequency (MHz)*** | 150.91 | ***Nucleus*** | 13C | ***Number of Transients*** | 2000 | ***Origin*** | ECA 600 |
| ***Original Points Count*** | 32768 | ***Owner*** | delta | ***Points Count*** | 32768 | ***Pulse Sequence*** | single_pulse_dec |
| ***Receiver Gain*** | 52.00 | ***Solvent*** | CHLOROFORM-d | | | ***Spectrum Offset (Hz)*** | 15091.3428 |
| ***Sweep Width (Hz)*** | 47348.49 | ***Temperature (degree C)*** | 22.100 | | | | |

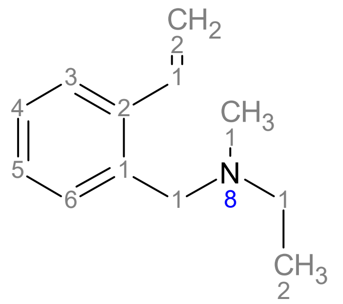


4b

FZ6702-2.jdf

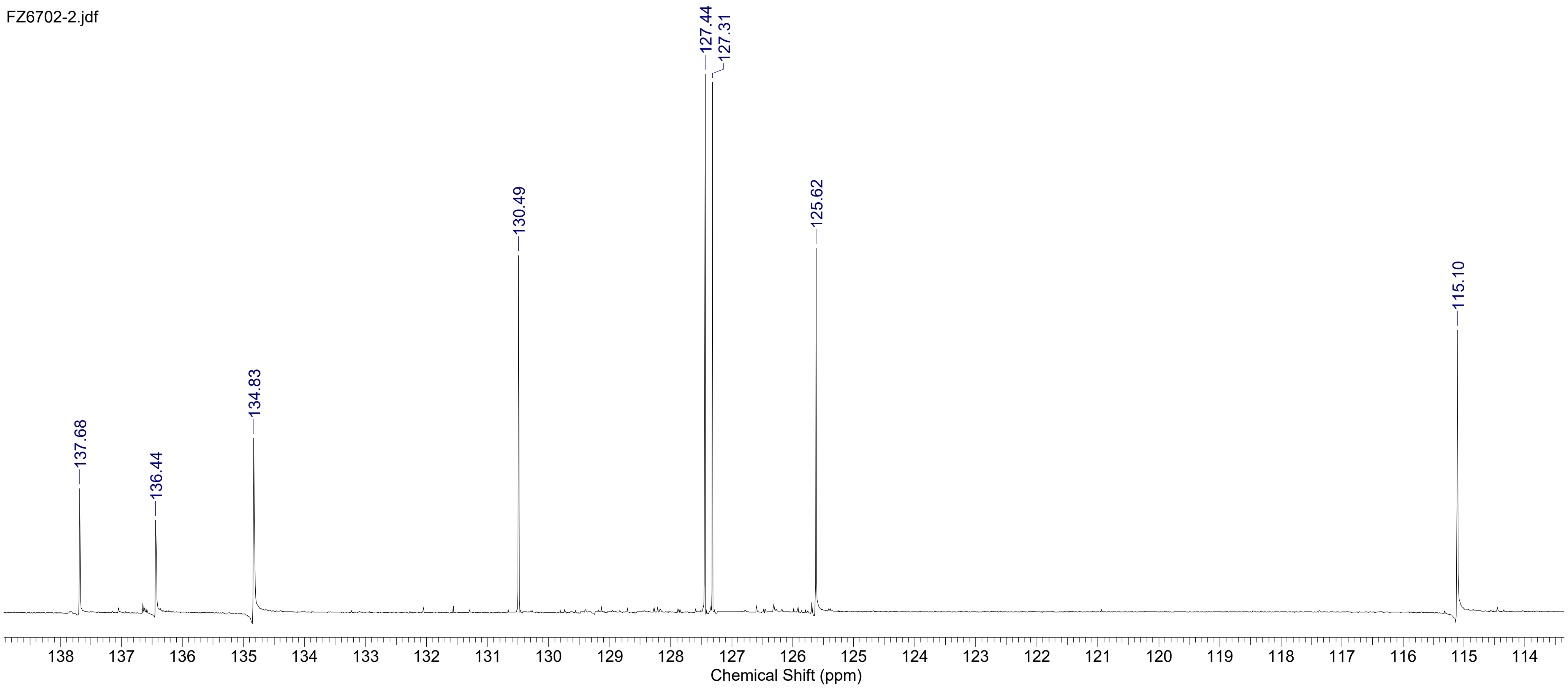

| | | | | | | | |
|---|---|---|---|---|---|---|---|
| ***Acquisition Time (sec)*** | 0.6921 | ***Comment*** | single pulse decoupled gated NOE | | | ***Date*** | 18 May 1990 05:49:40 |
| ***Date Stamp*** | 14 May 2018 10:36:25 | | | ***File Name*** | C:\Users\Fedor\Desktop\FZ6702-2.jdf | | |
| ***Frequency (MHz)*** | 150.91 | ***Nucleus*** | 13C | ***Number of Transients*** | 2000 | ***Origin*** | ECA 600 |
| ***Original Points Count*** | 32768 | ***Owner*** | delta | ***Points Count*** | 32768 | ***Pulse Sequence*** | single_pulse_dec |
| ***Receiver Gain*** | 52.00 | ***Solvent*** | CHLOROFORM-d | | | ***Spectrum Offset (Hz)*** | 15091.3428 |
| ***Sweep Width (Hz)*** | 47348.49 | ***Temperature (degree C)*** | 22.100 | | | | |

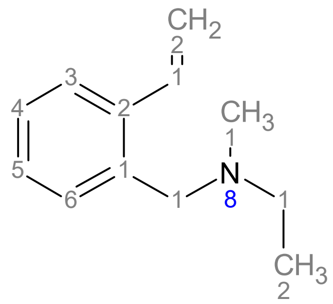


4b

FZ6702-2.jdf

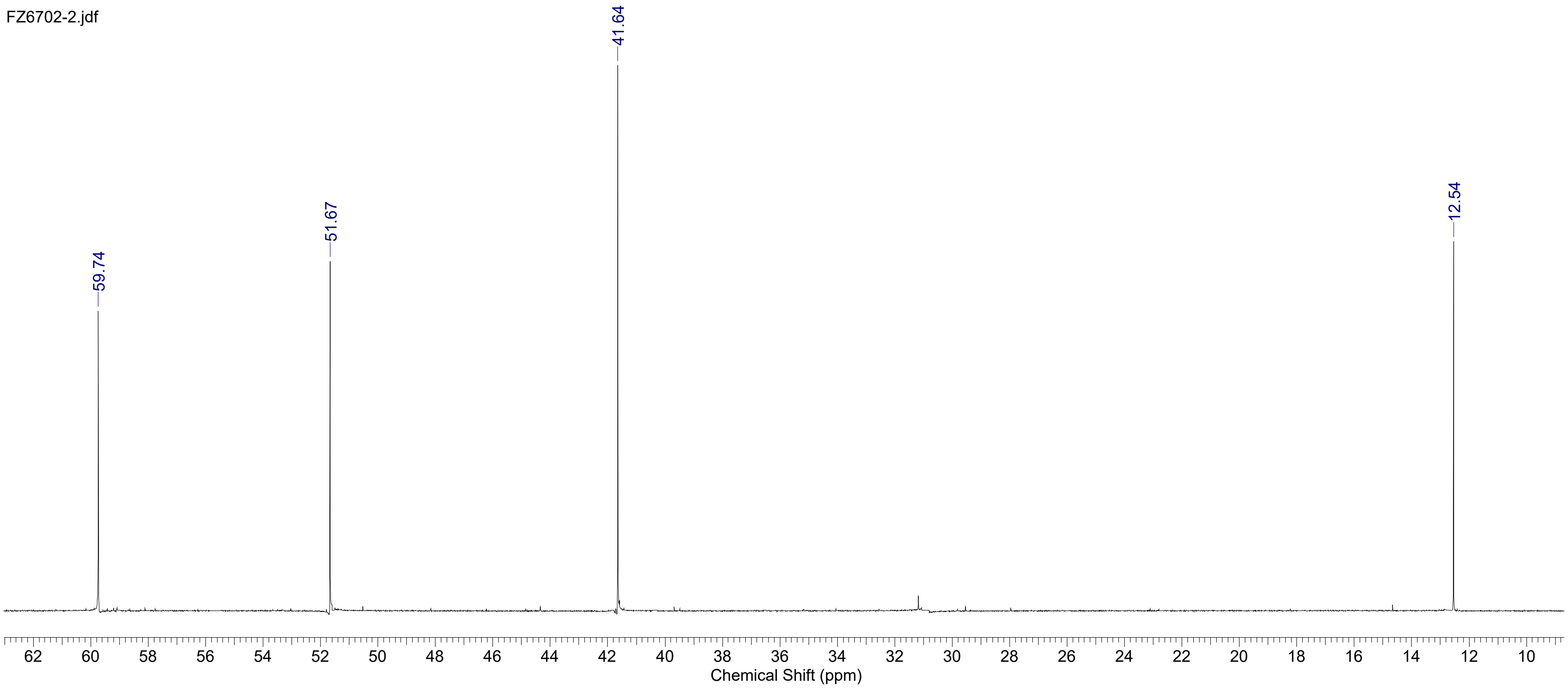

| | | | | | | | |
|---|---|---|---|---|---|---|---|
| ***Acquisition Time (sec)*** | 1.9818 | ***Comment*** | single_pulse | ***Date*** | 15 Apr 1990 04:18:56 | | |
| ***Date Stamp*** | 11 Apr 2018 09:06:28 | | | ***File Name*** | C:\Users\Fedor\Desktop\10.04.18\FZ6611-1.jdf | ***Frequency (MHz)*** | 600.17 |
| ***Nucleus*** | 1H | ***Number of Transients*** | 8 | ***Origin*** | ECA 600 | ***Original Points Count*** | 32768 |
| | | | | | | ***Owner*** | delta |
| ***Points Count*** | 32768 | ***Pulse Sequence*** | single_pulse.ex2 | ***Receiver Gain*** | 30.00 | ***Solvent*** | CHLOROFORM-d |
| ***Spectrum Offset (Hz)*** | 5401.5503 | ***Sweep Width (Hz)*** | 16534.39 | ***Temperature (degree C)*** | 21.900 | | |

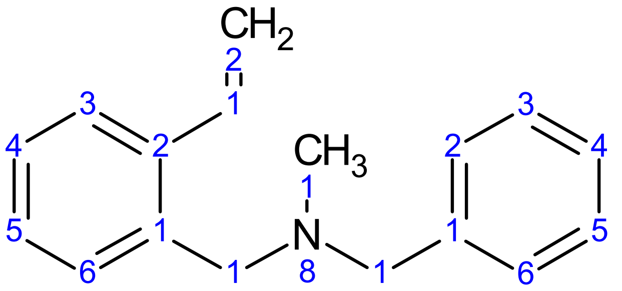


4c

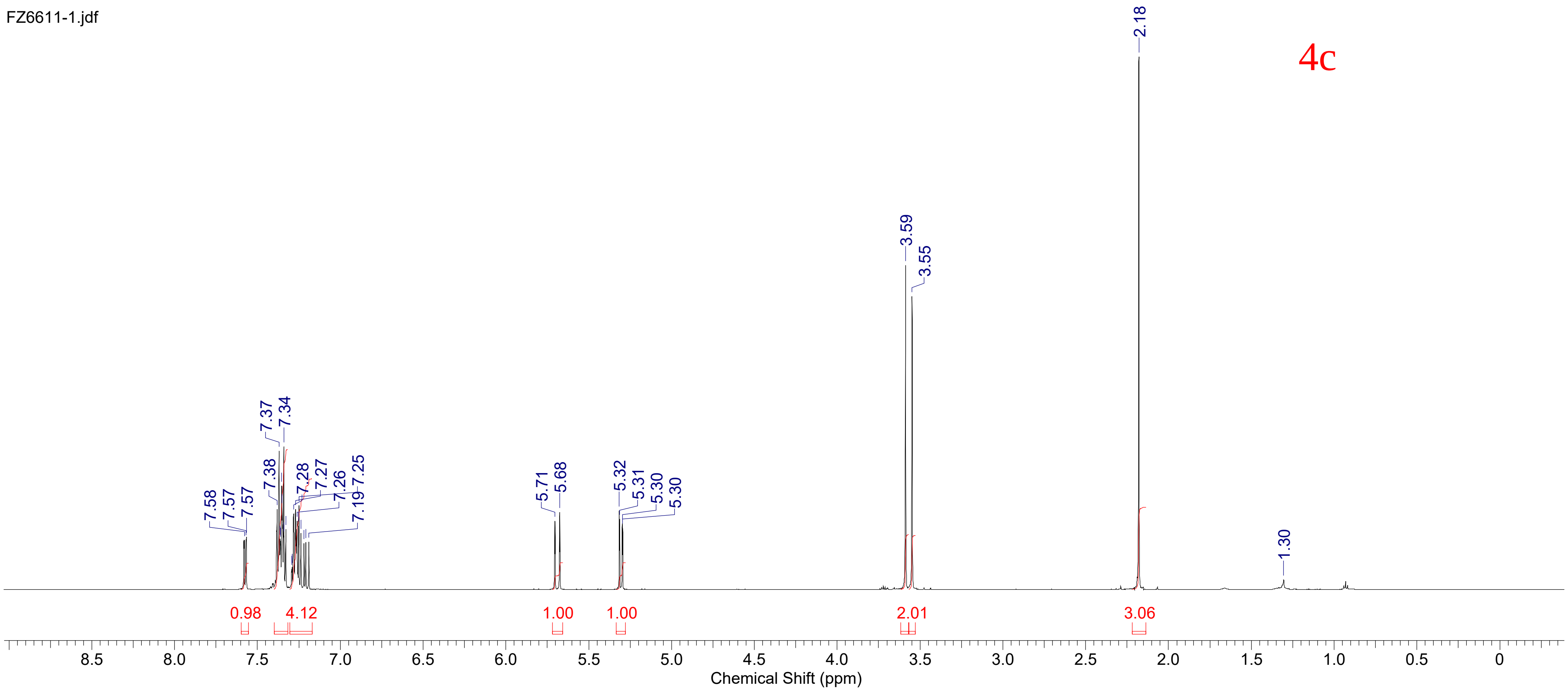

| | | | | | | | |
|---|---|---|---|---|---|---|---|
| ***Acquisition Time (sec)*** | 1.9818 | ***Comment*** | single_pulse | ***Date*** | 15 Apr 1990 04:18:56 | | |
| ***Date Stamp*** | 11 Apr 2018 09:06:28 | | | ***File Name*** | C:\Users\Fedor\Desktop\10.04.18\FZ6611-1.jdf | ***Frequency (MHz)*** | 600.17 |
| ***Nucleus*** | 1H | ***Number of Transients*** | 8 | ***Origin*** | ECA 600 | ***Original Points Count*** 32768 | ***Owner*** delta |
| ***Points Count*** | 32768 | ***Pulse Sequence*** | single_pulse.ex2 | | | ***Receiver Gain*** 30.00 | ***Solvent*** CHLOROFORM-d |
| ***Spectrum Offset (Hz)*** | 5401.5503 | ***Sweep Width (Hz)*** | 16534.39 | ***Temperature (degree C)*** | 21.900 | | |

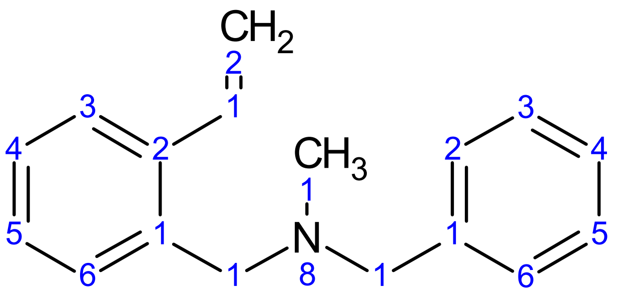


4c

FZ6611-1.jdf

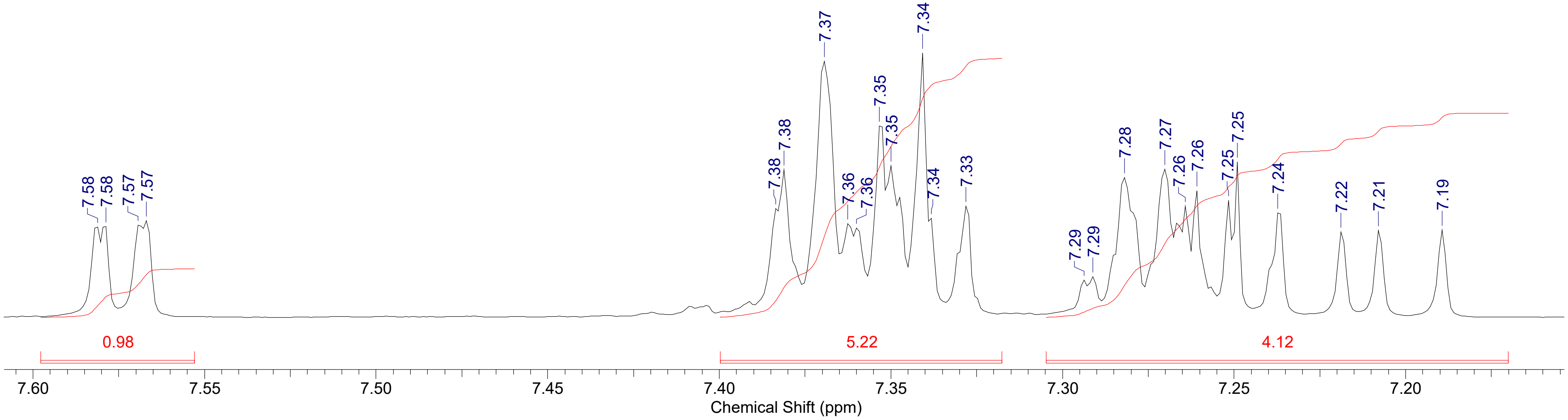

| | | | | | | | |
|---|---|---|---|---|---|---|---|
| ***Acquisition Time (sec)*** | 1.9818 | ***Comment*** | single_pulse | ***Date*** | 15 Apr 1990 04:18:56 | | |
| ***Date Stamp*** | 11 Apr 2018 09:06:28 | | | ***File Name*** | C:\Users\Fedor\Desktop\10.04.18\FZ6611-1.jdf | ***Frequency (MHz)*** | 600.17 |
| ***Nucleus*** | 1H | ***Number of Transients*** | 8 | ***Origin*** | ECA 600 | ***Original Points Count*** | 32768 |
| ***Owner*** | delta | | | | | | |
| ***Points Count*** | 32768 | ***Pulse Sequence*** | single_pulse.ex2 | ***Receiver Gain*** | 30.00 | ***Solvent*** | CHLOROFORM-d |
| ***Spectrum Offset (Hz)*** | 5401.5503 | ***Sweep Width (Hz)*** | 16534.39 | ***Temperature (degree C)*** | 21.900 | | |

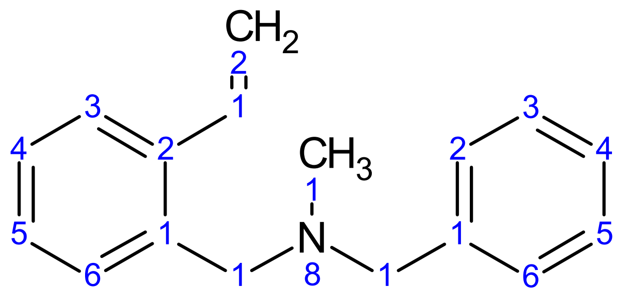


4c

FZ6611-1.jdf

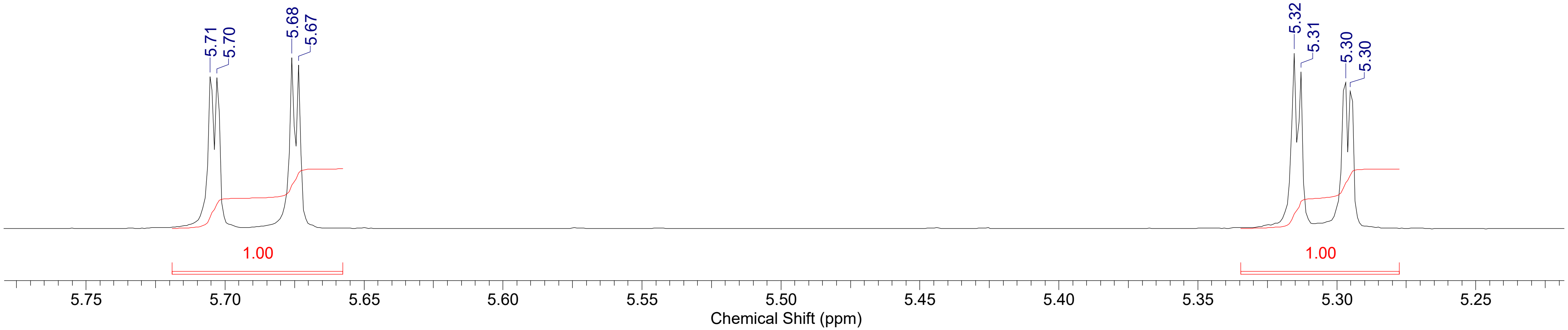

| | | | | | | | |
|---|---|---|---|---|---|---|---|
| ***Acquisition Time (sec)*** | 1.9818 | ***Comment*** | single_pulse | ***Date*** | 15 Apr 1990 04:18:56 | | |
| ***Date Stamp*** | 11 Apr 2018 09:06:28 | | | ***File Name*** | C:\Users\Fedor\Desktop\10.04.18\FZ6611-1.jdf | ***Frequency (MHz)*** | 600.17 |
| ***Nucleus*** | 1H | ***Number of Transients*** | 8 | ***Origin*** | ECA 600 | ***Original Points Count*** 32768 | ***Owner*** delta |
| ***Points Count*** | 32768 | ***Pulse Sequence*** | single_pulse.ex2 | | | ***Receiver Gain*** 30.00 | ***Solvent*** CHLOROFORM-d |
| ***Spectrum Offset (Hz)*** | 5401.5503 | ***Sweep Width (Hz)*** | 16534.39 | ***Temperature (degree C)*** | 21.900 | | |

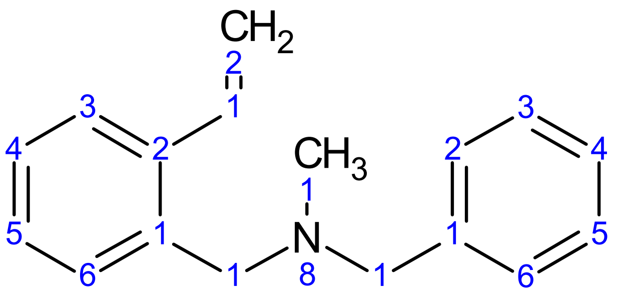


4c

FZ6611-1.jdf

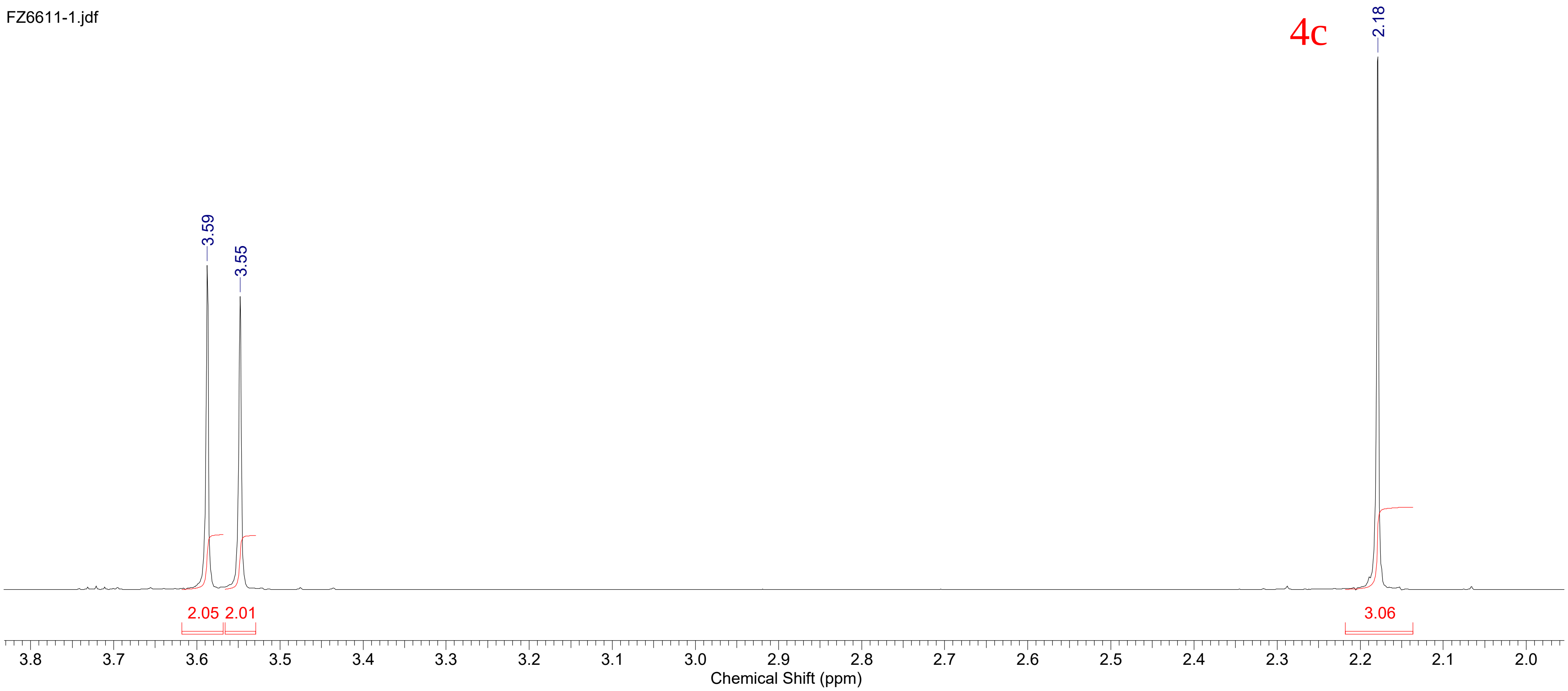

| Acquisition Time (sec) | 0.6921 | Comment | single pulse decoupled gated NOE | | | Date | 27 Apr 1990 04:56:23 |
|---|---|---|---|---|---|---|---|
| Date Stamp | 23 Apr 2018 09:43:28 | | | File Name | C:\Users\Fedor\Desktop\FZ6622-2.jdf | | |
| Frequency (MHz) | 150.91 | Nucleus | 13C | Number of Transients | 800 | Origin | ECA 600 |
| Original Points Count | 32768 | Owner | delta | Points Count | 32768 | Pulse Sequence | single_pulse_dec |
| Receiver Gain | 54.00 | Solvent | CHLOROFORM-d | | | Spectrum Offset (Hz) | 15091.3428 |
| Sweep Width (Hz) | 47348.49 | Temperature (degree C) | 23.400 | | | | |

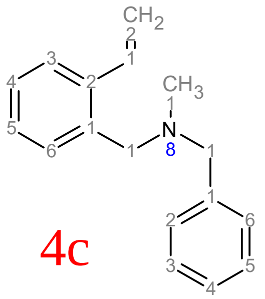


4c

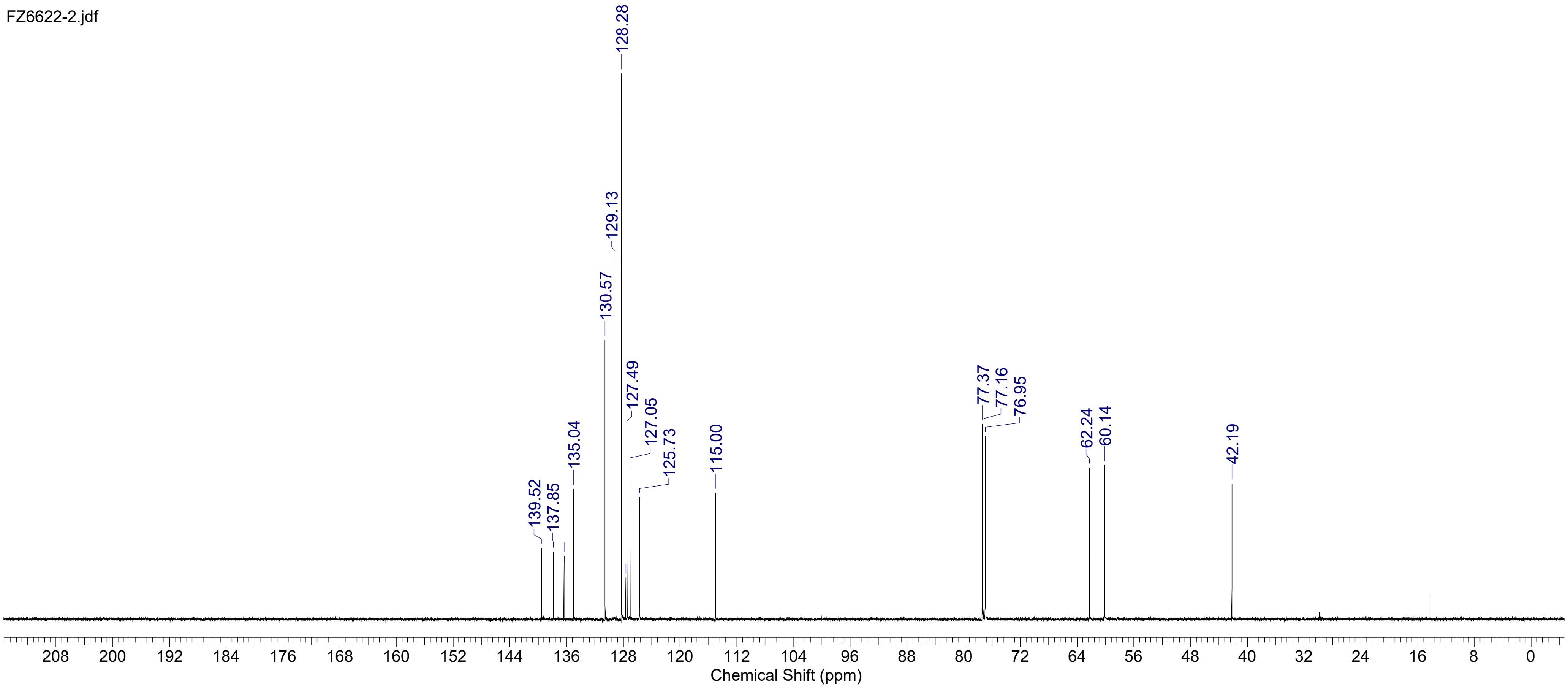

| | | | | | | | |
|---|---|---|---|---|---|---|---|
| ***Acquisition Time (sec)*** | 0.6921 | ***Comment*** | single pulse decoupled gated NOE | | | ***Date*** | 27 Apr 1990 04:56:23 |
| ***Date Stamp*** | 23 Apr 2018 09:43:28 | | | ***File Name*** | C:\Users\Fedor\Desktop\FZ6622-2.jdf | | |
| ***Frequency (MHz)*** | 150.91 | ***Nucleus*** | 13C | ***Number of Transients*** | 800 | ***Origin*** | ECA 600 |
| ***Original Points Count*** | 32768 | ***Owner*** | delta | ***Points Count*** | 32768 | ***Pulse Sequence*** | single_pulse_dec |
| ***Receiver Gain*** | 54.00 | ***Solvent*** | CHLOROFORM-d | | | ***Spectrum Offset (Hz)*** | 15091.3428 |
| ***Sweep Width (Hz)*** | 47348.49 | ***Temperature (degree C)*** | 23.400 | | | | |

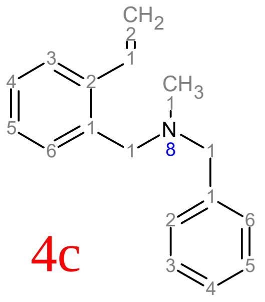


4c

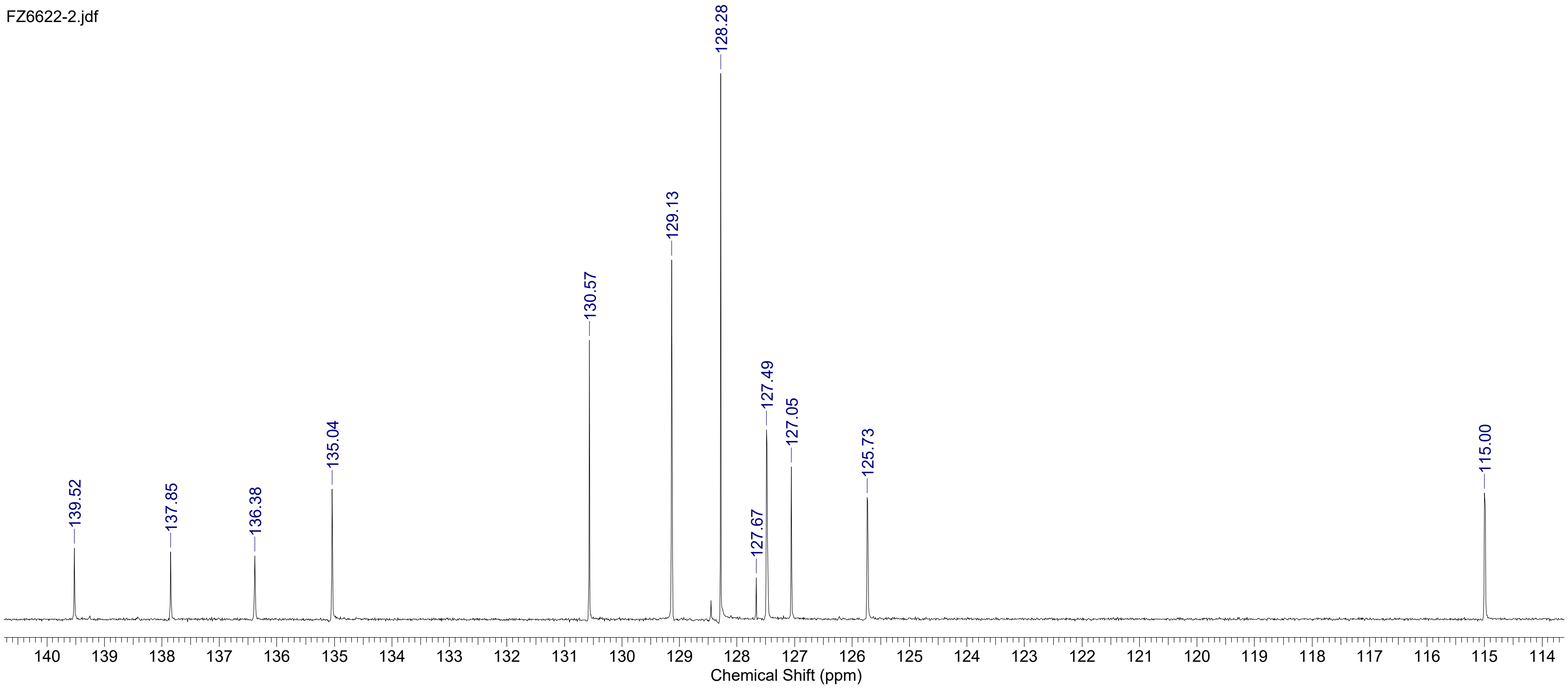

| | | | | | | | |
|---|---|---|---|---|---|---|---|
| ***Acquisition Time (sec)*** | 0.6921 | ***Comment*** | single pulse decoupled gated NOE | | | ***Date*** | 27 Apr 1990 04:56:23 |
| ***Date Stamp*** | 23 Apr 2018 09:43:28 | | | ***File Name*** | C:\Users\Fedor\Desktop\FZ6622-2.jdf | | |
| ***Frequency (MHz)*** | 150.91 | ***Nucleus*** | 13C | ***Number of Transients*** | 800 | ***Origin*** | ECA 600 |
| ***Original Points Count*** | 32768 | ***Owner*** | delta | ***Points Count*** | 32768 | ***Pulse Sequence*** | single_pulse_dec |
| ***Receiver Gain*** | 54.00 | ***Solvent*** | CHLOROFORM-d | | | ***Spectrum Offset (Hz)*** | 15091.3428 |
| ***Sweep Width (Hz)*** | 47348.49 | ***Temperature (degree C)*** | 23.400 | | | | |

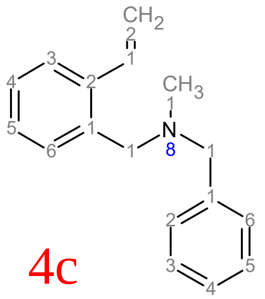


4c

FZ6622-2.jdf

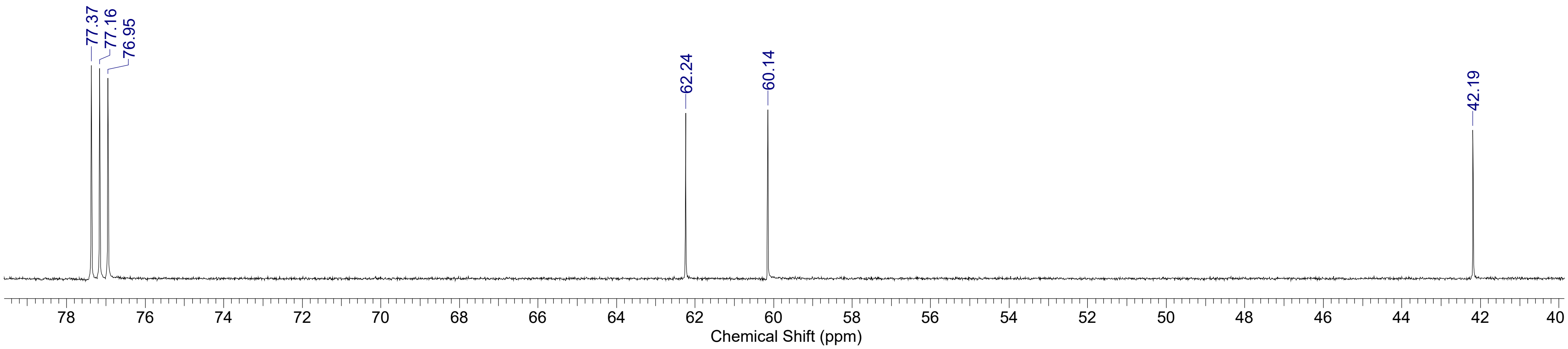

| | | | | | | | | | |
|---|---|---|---|---|---|---|---|---|---|
| ***Acquisition Time (sec)*** | 1.9818 | ***Comment*** | single_pulse | ***Date*** | 15 Apr 1990 04:13:18 | | | | |
| ***Date Stamp*** | 11 Apr 2018 09:00:50 | | | ***File Name*** | C:\Users\Fedor\Desktop\10.04.18\FZ6610-1.jdf | | | ***Frequency (MHz)*** | 600.17 |
| ***Nucleus*** | 1H | ***Number of Transients*** | 8 | ***Origin*** | ECA 600 | ***Original Points Count*** | 32768 | ***Owner*** | delta |
| ***Points Count*** | 32768 | ***Pulse Sequence*** | single_pulse.ex2 | | | ***Receiver Gain*** | 26.00 | ***Solvent*** | CHLOROFORM-d |
| ***Spectrum Offset (Hz)*** | 5401.5503 | ***Sweep Width (Hz)*** | 16534.39 | ***Temperature (degree C)*** | 22.000 | | | | |

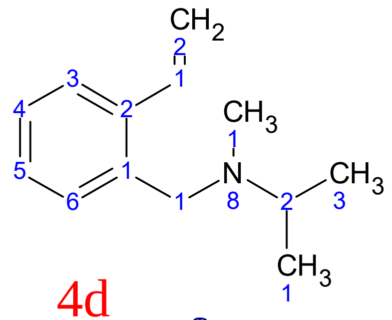

4d

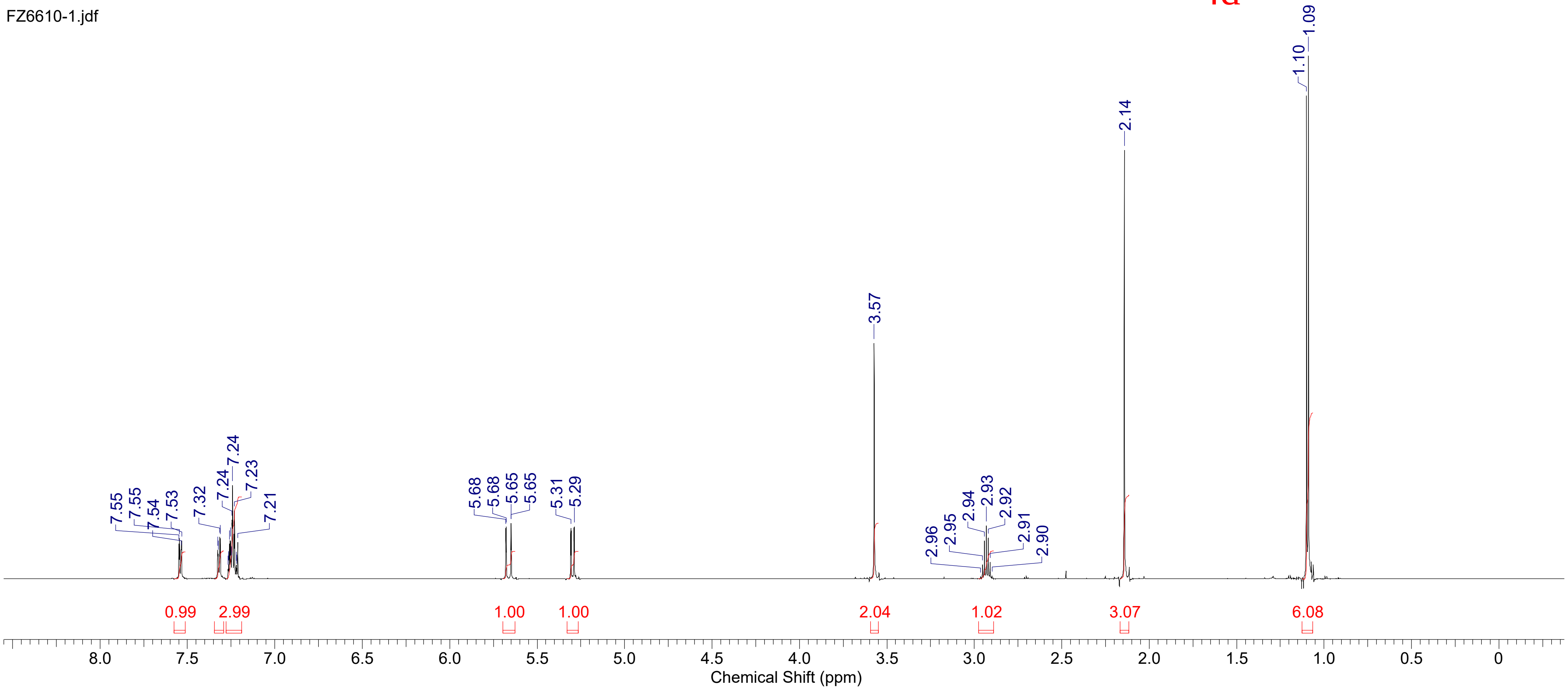

| | | | | | | | |
|---|---|---|---|---|---|---|---|
| **Acquisition Time (sec)** | 1.9818 | **Comment** | single_pulse | **Date** | 15 Apr 1990 04:13:18 | | |
| **Date Stamp** | 11 Apr 2018 09:00:50 | | | **File Name** | C:\Users\Fedor\Desktop\10.04.18\FZ6610-1.jdf | **Frequency (MHz)** | 600.17 |
| **Nucleus** | 1H | **Number of Transients** | 8 | **Origin** | ECA 600 | **Original Points Count** | 32768 |
| | | | | | | **Owner** | delta |
| **Points Count** | 32768 | **Pulse Sequence** | single_pulse.ex2 | | | **Receiver Gain** | 26.00 |
| | | | | | | **Solvent** | CHLOROFORM-d |
| **Spectrum Offset (Hz)** | 5401.5503 | **Sweep Width (Hz)** | 16534.39 | **Temperature (degree C)** | 22.000 | | |

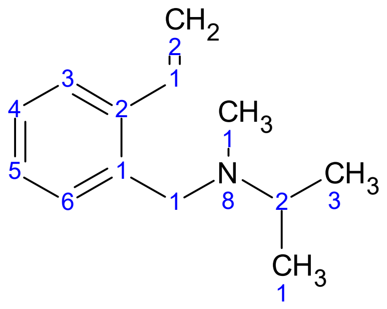

FZ6610-1.jdf

4d

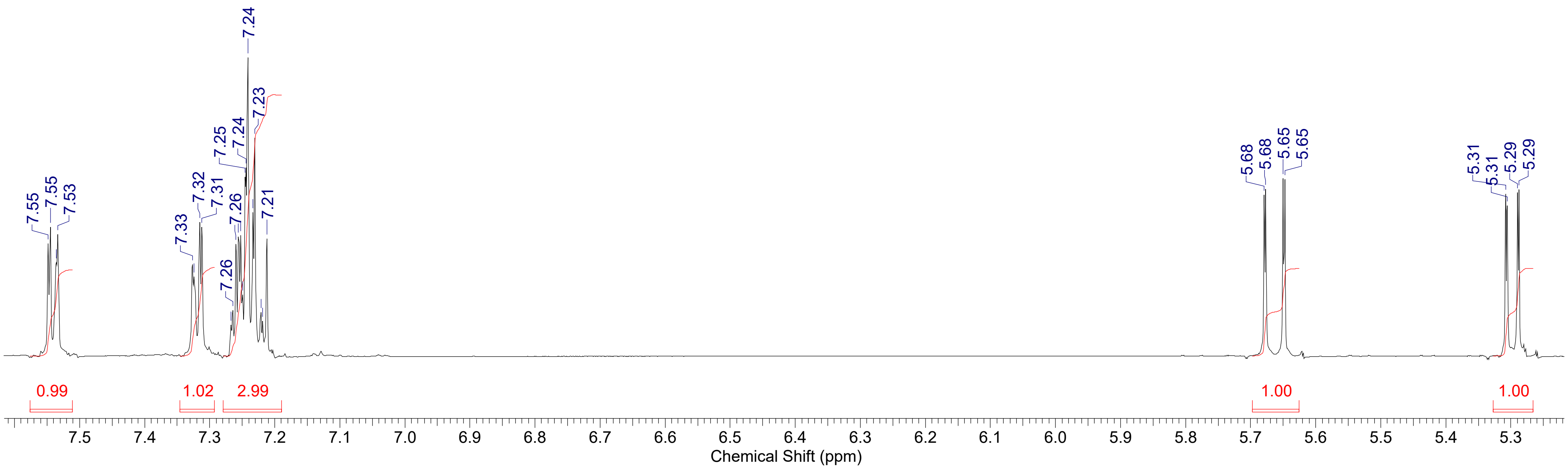

| | | | | | | | | | |
|---|---|---|---|---|---|---|---|---|---|
| ***Acquisition Time (sec)*** | 1.9818 | ***Comment*** | single_pulse | ***Date*** | 15 Apr 1990 04:13:18 | | | | |
| ***Date Stamp*** | 11 Apr 2018 09:00:50 | | | ***File Name*** | C:\Users\Fedor\Desktop\10.04.18\FZ6610-1.jdf | | | ***Frequency (MHz)*** | 600.17 |
| ***Nucleus*** | 1H | ***Number of Transients*** | 8 | ***Origin*** | ECA 600 | ***Original Points Count*** | 32768 | ***Owner*** | delta |
| ***Points Count*** | 32768 | ***Pulse Sequence*** | single_pulse.ex2 | | | ***Receiver Gain*** | 26.00 | ***Solvent*** | CHLOROFORM-d |
| ***Spectrum Offset (Hz)*** | 5401.5503 | ***Sweep Width (Hz)*** | 16534.39 | ***Temperature (degree C)*** | 22.000 | | | | |

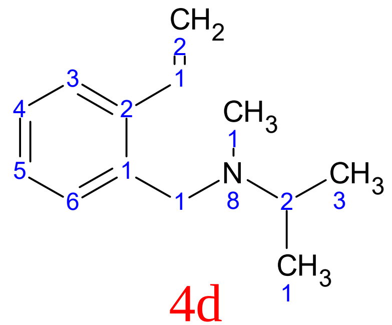

4d

FZ6610-1.jdf

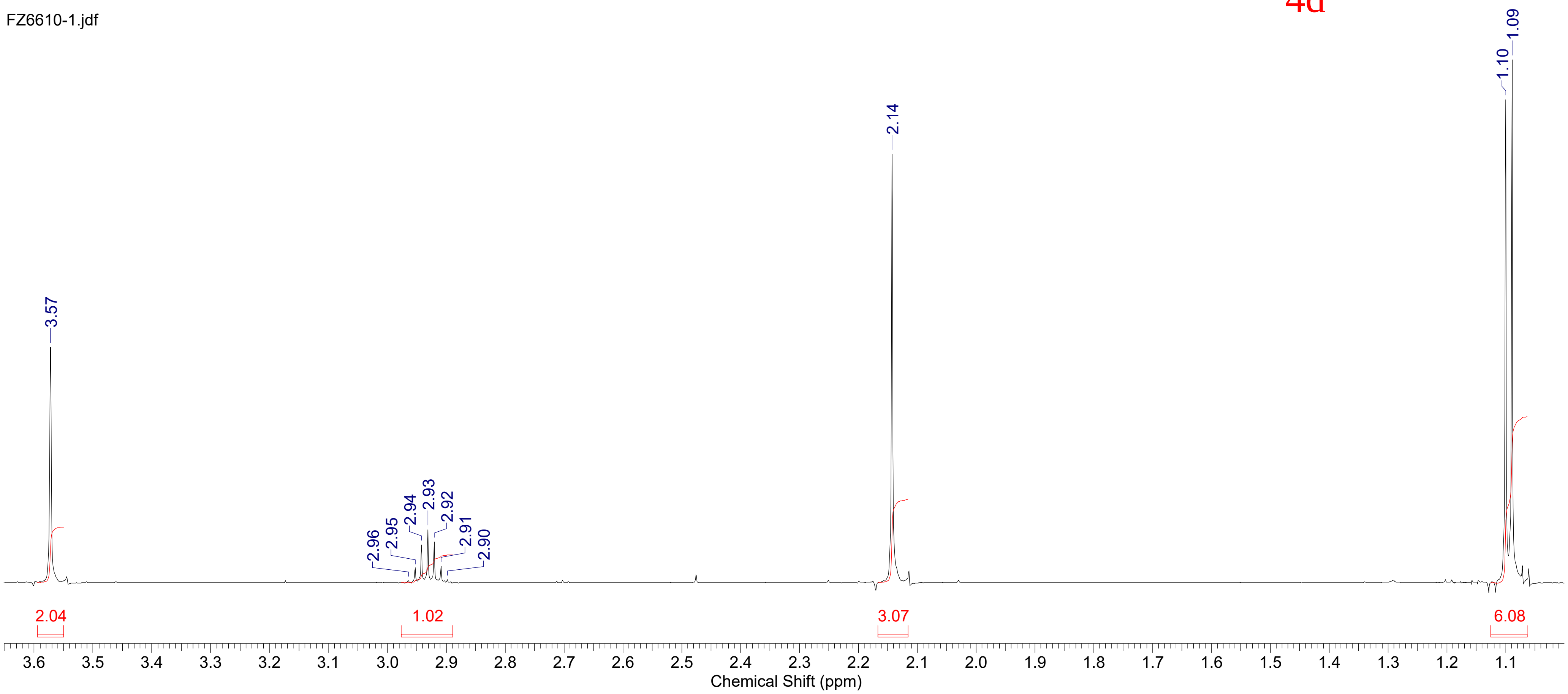

| | | | | | | | |
|---|---|---|---|---|---|---|---|
| ***Acquisition Time (sec)*** | 0.6921 | ***Comment*** | single pulse decoupled gated NOE | | | ***Date*** | 22 Apr 1990 08:30:36 |
| ***Date Stamp*** | 18 Apr 2018 13:18:15 | | | ***File Name*** | C:\Users\Fedor\Desktop\17.04.18\FZ6621-1.jdf | | |
| ***Frequency (MHz)*** | 150.91 | ***Nucleus*** | 13C | ***Number of Transients*** | 246 | ***Origin*** | ECA 600 |
| ***Original Points Count*** | 32768 | ***Owner*** | delta | ***Points Count*** | 32768 | ***Pulse Sequence*** | single_pulse_dec |
| ***Receiver Gain*** | 52.00 | ***Solvent*** | CHLOROFORM-d | | | ***Spectrum Offset (Hz)*** | 15091.3428 |
| ***Sweep Width (Hz)*** | 47348.49 | ***Temperature (degree C)*** | 22.700 | | | | |

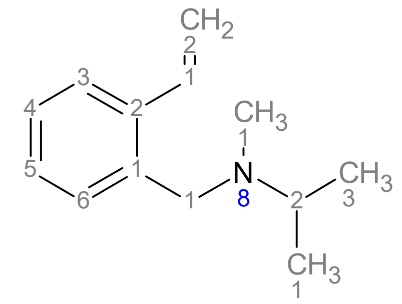


4d

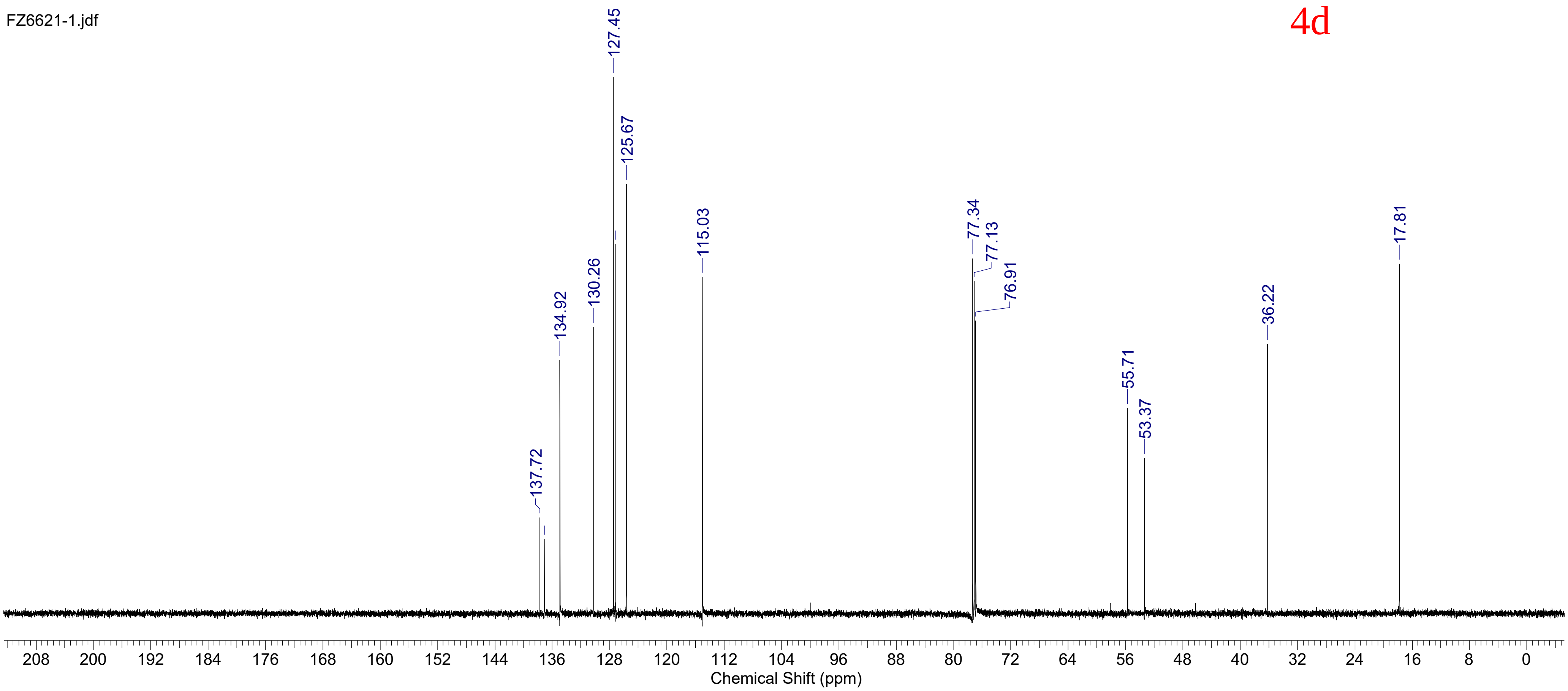

| | | | | | | | |
|---|---|---|---|---|---|---|---|
| ***Acquisition Time (sec)*** | 0.6921 | ***Comment*** | single pulse decoupled gated NOE | | | ***Date*** | 22 Apr 1990 08:30:36 |
| ***Date Stamp*** | 18 Apr 2018 13:18:15 | | | ***File Name*** | C:\Users\Fedor\Desktop\17.04.18\FZ6621-1.jdf | | |
| ***Frequency (MHz)*** | 150.91 | ***Nucleus*** | 13C | ***Number of Transients*** | 246 | ***Origin*** | ECA 600 |
| ***Original Points Count*** | 32768 | ***Owner*** | delta | ***Points Count*** | 32768 | ***Pulse Sequence*** | single_pulse_dec |
| ***Receiver Gain*** | 52.00 | ***Solvent*** | CHLOROFORM-d | | | ***Spectrum Offset (Hz)*** | 15091.3428 |
| ***Sweep Width (Hz)*** | 47348.49 | ***Temperature (degree C)*** | 22.700 | | | | |

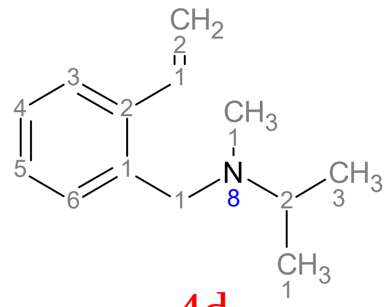


4d

FZ6621-1.jdf

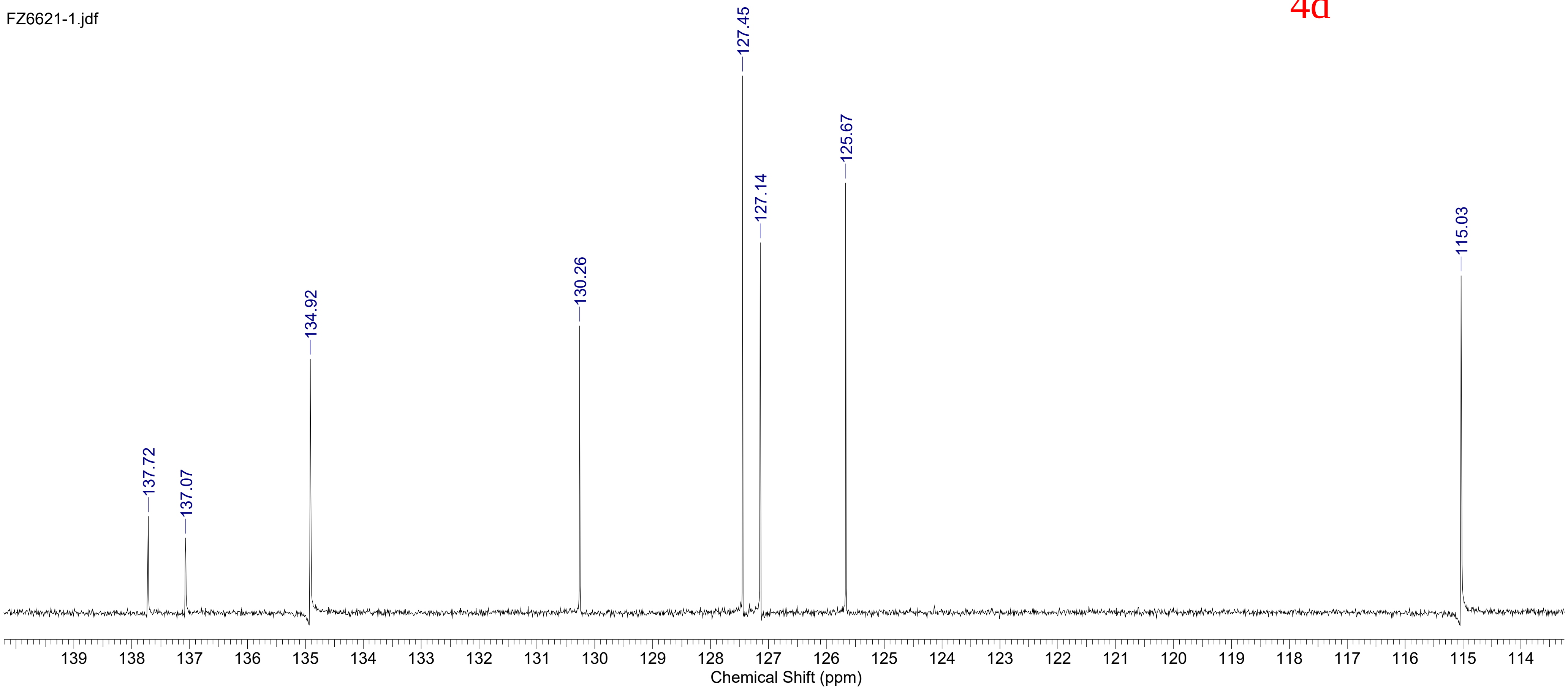

| | | | | | | | |
|---|---|---|---|---|---|---|---|
| ***Acquisition Time (sec)*** | 0.6921 | ***Comment*** | single pulse decoupled gated NOE | | | ***Date*** | 22 Apr 1990 08:30:36 |
| ***Date Stamp*** | 18 Apr 2018 13:18:15 | | | ***File Name*** | C:\Users\Fedor\Desktop\17.04.18\FZ6621-1.jdf | | |
| ***Frequency (MHz)*** | 150.91 | ***Nucleus*** | 13C | ***Number of Transients*** | 246 | ***Origin*** | ECA 600 |
| ***Original Points Count*** | 32768 | ***Owner*** | delta | ***Points Count*** | 32768 | ***Pulse Sequence*** | single_pulse_dec |
| ***Receiver Gain*** | 52.00 | ***Solvent*** | CHLOROFORM-d | | | ***Spectrum Offset (Hz)*** | 15091.3428 |
| ***Sweep Width (Hz)*** | 47348.49 | ***Temperature (degree C)*** | 22.700 | | | | |

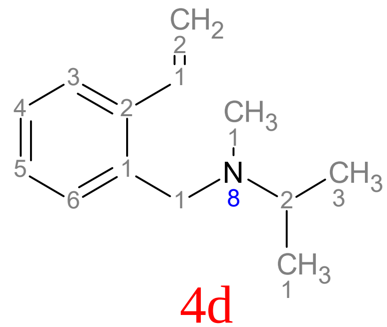


4d

FZ6621-1.jdf

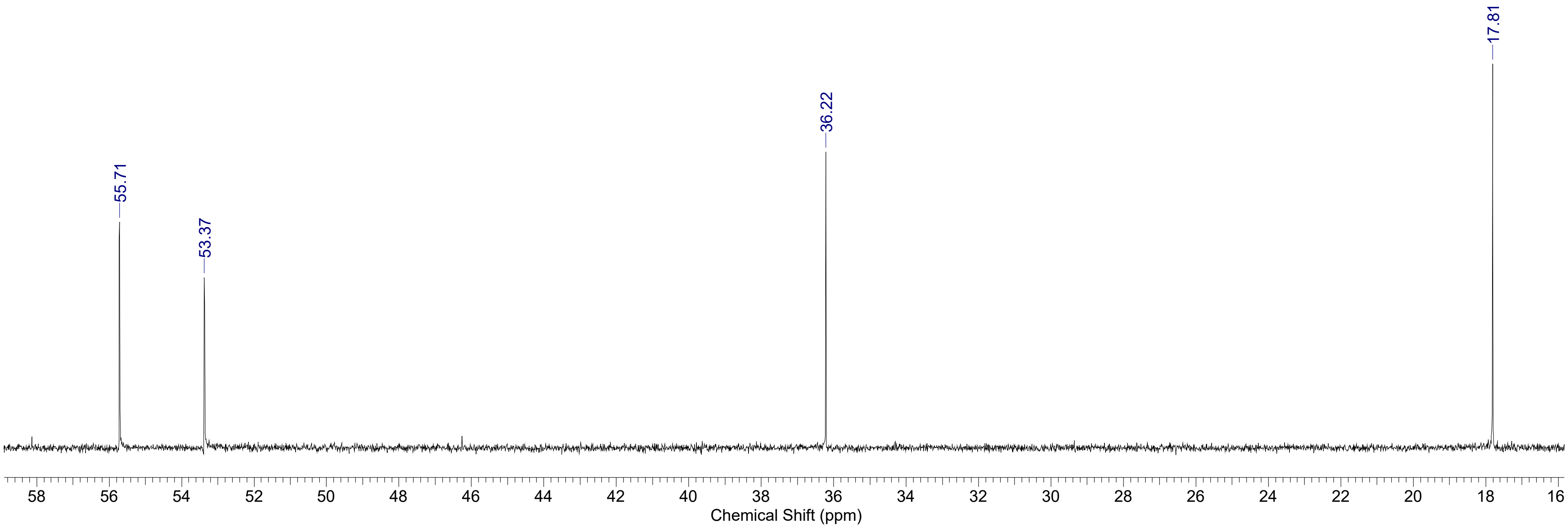

| | | | | | | | |
|---|---|---|---|---|---|---|---|
| ***Acquisition Time (sec)*** | 1.9818 | ***Comment*** | single_pulse | ***Date*** | 21 May 1990 06:49:43 | | |
| ***Date Stamp*** | 17 May 2018 11:36:26 | | | ***File Name*** | C:\Users\Fedor\Desktop\15.05.18\FZ6709-1.jdf | ***Frequency (MHz)*** | 600.17 |
| ***Nucleus*** | 1H | ***Number of Transients*** | 8 | ***Origin*** | ECA 600 ***Original Points Count*** 32768 | ***Owner*** | delta |
| ***Points Count*** | 32768 | ***Pulse Sequence*** | single_pulse.ex2 | | ***Receiver Gain*** 26.00 | ***Solvent*** | CHLOROFORM-d |
| ***Spectrum Offset (Hz)*** | 5401.5503 | ***Sweep Width (Hz)*** | 16534.39 | ***Temperature (degree C)*** | 19.600 | | |

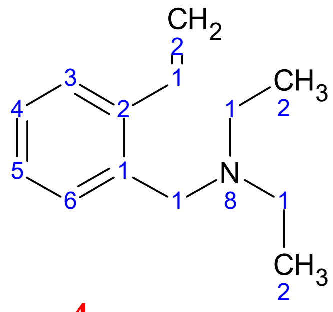

4e

FZ6709-1.jdf

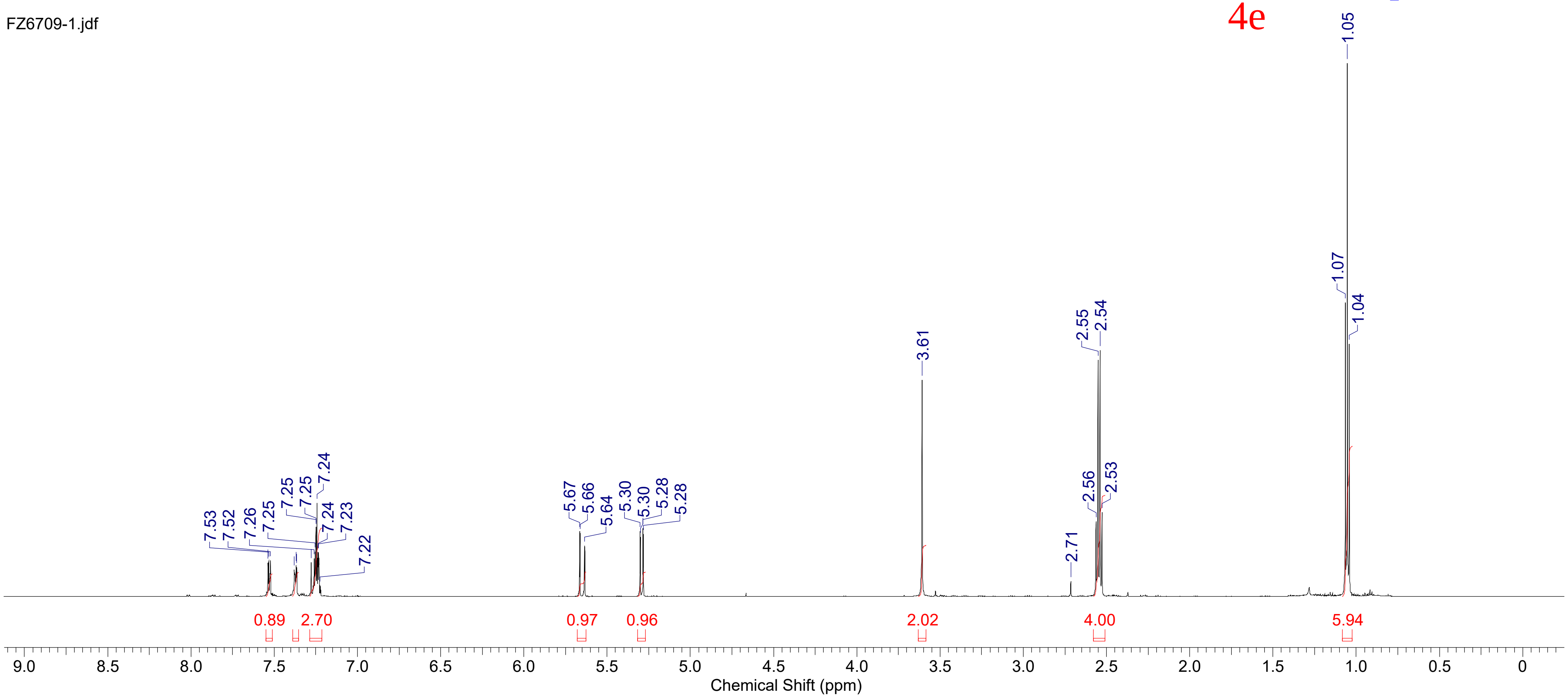

| | | | | | | | |
|---|---|---|---|---|---|---|---|
| **Acquisition Time (sec)** | 1.9818 | **Comment** | single_pulse | **Date** | 21 May 1990 06:49:43 | | |
| **Date Stamp** | 17 May 2018 11:36:26 | | | **File Name** | C:\Users\Fedor\Desktop\15.05.18\FZ6709-1.jdf | **Frequency (MHz)** | 600.17 |
| **Nucleus** | 1H | **Number of Transients** | 8 | **Origin** | ECA 600 | **Original Points Count** | 32768 |
| **Owner** | delta | | | | | | |
| **Points Count** | 32768 | **Pulse Sequence** | single_pulse.ex2 | **Receiver Gain** | 26.00 | **Solvent** | CHLOROFORM-d |
| **Spectrum Offset (Hz)** | 5401.5503 | **Sweep Width (Hz)** | 16534.39 | **Temperature (degree C)** | 19.600 | | |

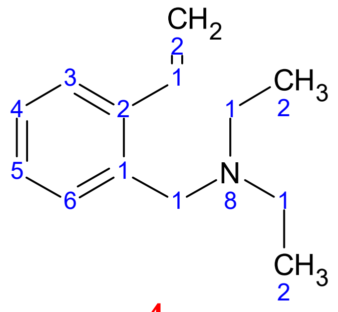

4e

FZ6709-1.jdf

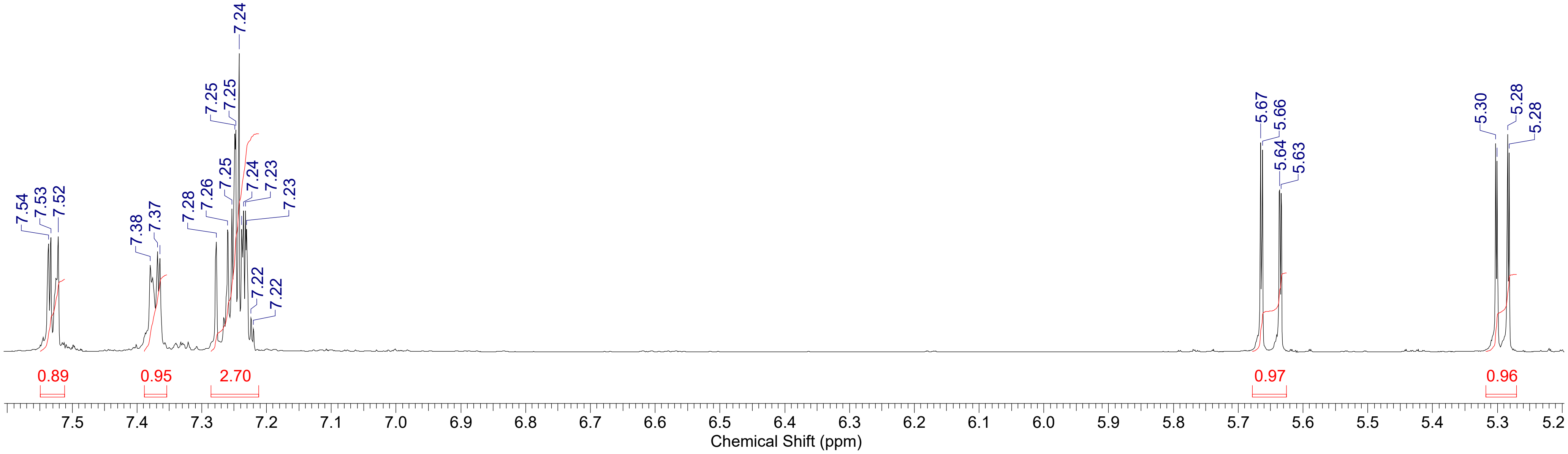

| | | | | | | | |
|---|---|---|---|---|---|---|---|
| ***Acquisition Time (sec)*** | 1.9818 | ***Comment*** | single_pulse | ***Date*** | 21 May 1990 06:49:43 | | |
| ***Date Stamp*** | 17 May 2018 11:36:26 | | | ***File Name*** | C:\Users\Fedor\Desktop\15.05.18\FZ6709-1.jdf | ***Frequency (MHz)*** | 600.17 |
| ***Nucleus*** | 1H | ***Number of Transients*** | 8 | ***Origin*** | ECA 600 | ***Original Points Count*** | 32768 |
| ***Owner*** | delta | | | | | | |
| ***Points Count*** | 32768 | ***Pulse Sequence*** | single_pulse.ex2 | ***Receiver Gain*** | 26.00 | ***Solvent*** | CHLOROFORM-d |
| ***Spectrum Offset (Hz)*** | 5401.5503 | ***Sweep Width (Hz)*** | 16534.39 | ***Temperature (degree C)*** | 19.600 | | |

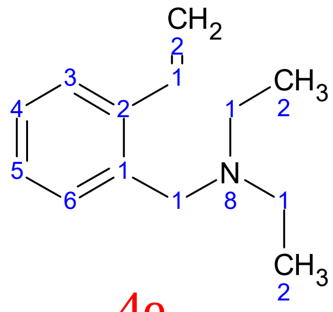

4e

FZ6709-1.jdf

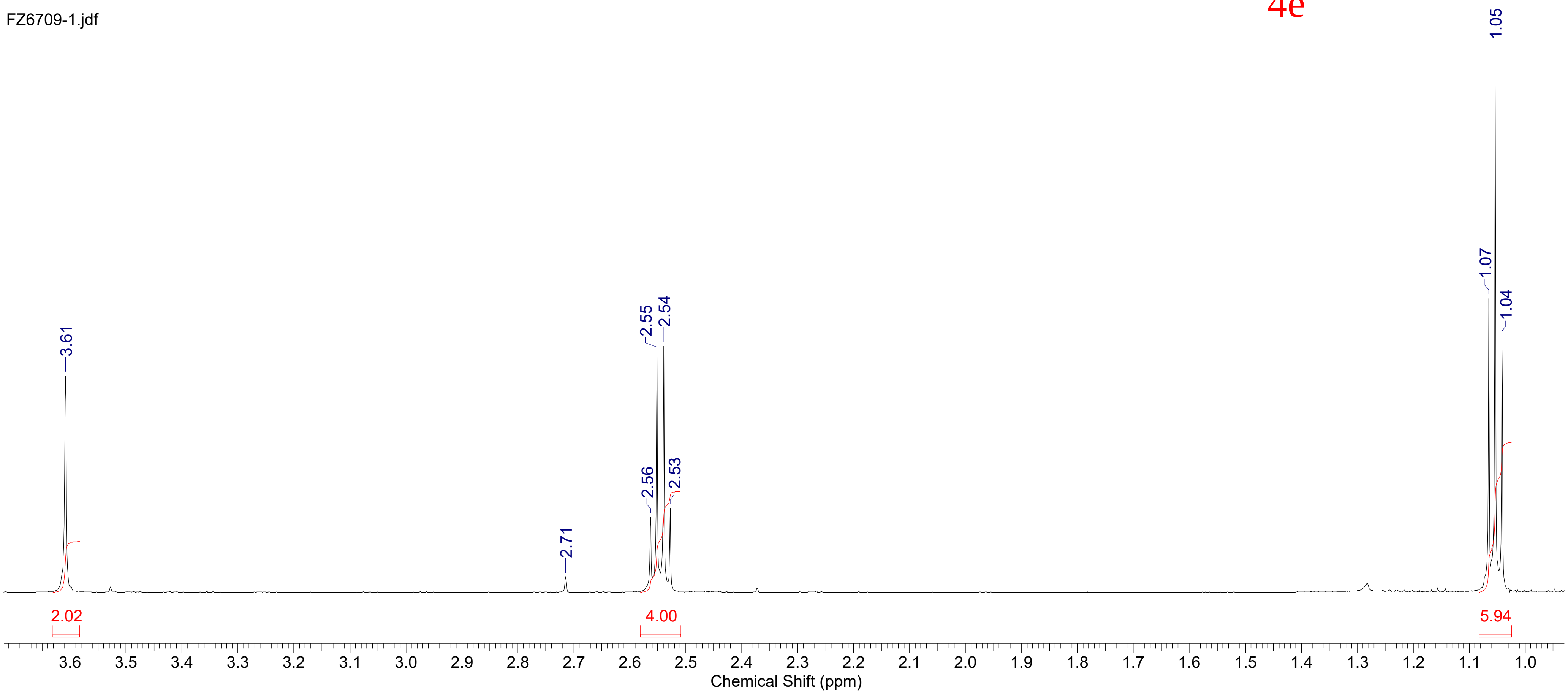

| | | | | | | | |
|---|---|---|---|---|---|---|---|
| ***Acquisition Time (sec)*** | 0.6921 | ***Comment*** | single pulse decoupled gated NOE | | | ***Date*** | 28 May 1990 00:10:35 |
| ***Date Stamp*** | 24 May 2018 04:57:18 | | | ***File Name*** | C:\Users\Fedor\Desktop\22.05.18\FZ6732-1.jdf | | |
| ***Frequency (MHz)*** | 150.91 | ***Nucleus*** | 13C | ***Number of Transients*** | 1000 | ***Origin*** | ECA 600 |
| ***Original Points Count*** | 32768 | ***Owner*** | delta | ***Points Count*** | 32768 | ***Pulse Sequence*** | single_pulse_dec |
| ***Receiver Gain*** | 52.00 | ***Solvent*** | CHLOROFORM-d | | | ***Spectrum Offset (Hz)*** | 15091.3428 |
| ***Sweep Width (Hz)*** | 47348.49 | ***Temperature (degree C)*** | 22.200 | | | | |

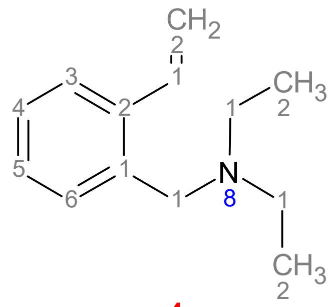


4e

FZ6732-1.jdf

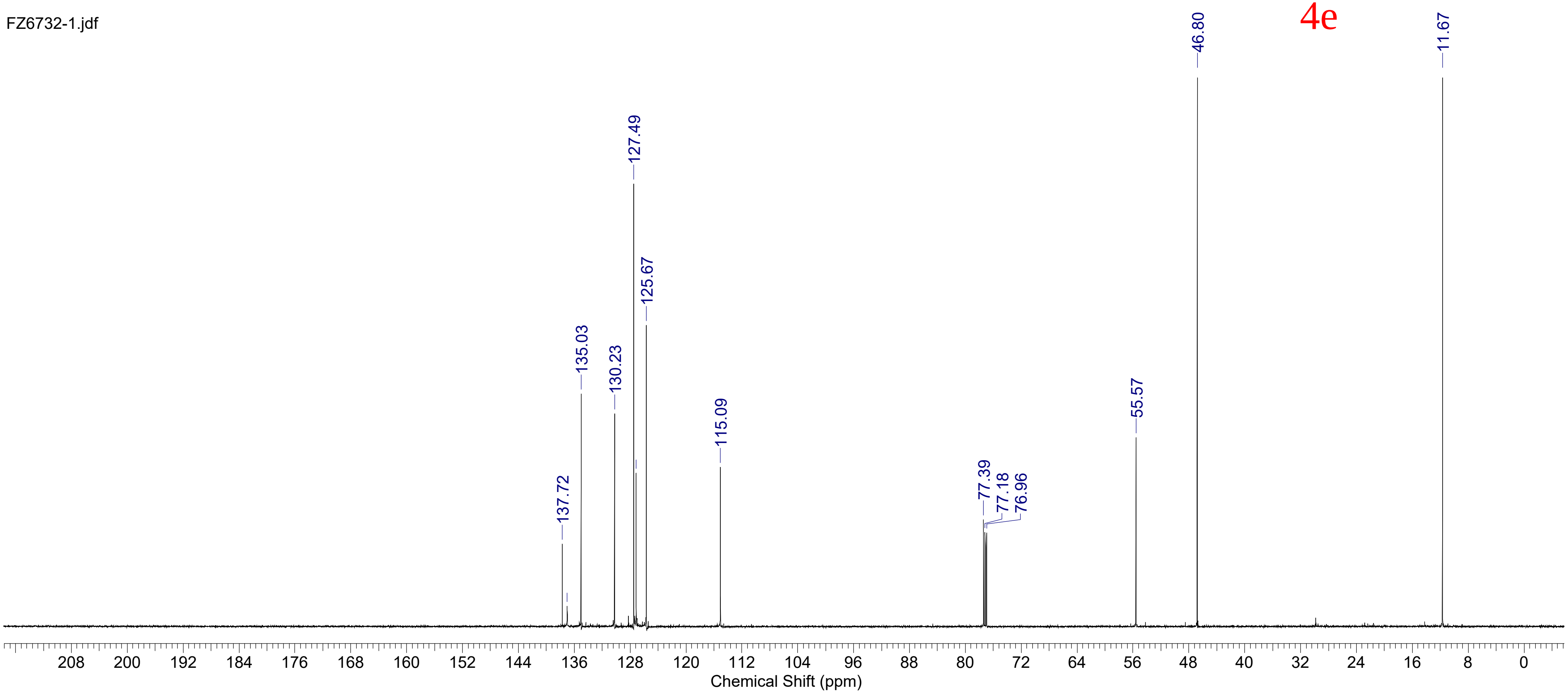

| | | | | | | | |
|---|---|---|---|---|---|---|---|
| ***Acquisition Time (sec)*** | 0.6921 | ***Comment*** | single pulse decoupled gated NOE | | | ***Date*** | 28 May 1990 00:10:35 |
| ***Date Stamp*** | 24 May 2018 04:57:18 | | | ***File Name*** | C:\Users\Fedor\Desktop\22.05.18\FZ6732-1.jdf | | |
| ***Frequency (MHz)*** | 150.91 | ***Nucleus*** | 13C | ***Number of Transients*** | 1000 | ***Origin*** | ECA 600 |
| ***Original Points Count*** | 32768 | ***Owner*** | delta | ***Points Count*** | 32768 | ***Pulse Sequence*** | single_pulse_dec |
| ***Receiver Gain*** | 52.00 | ***Solvent*** | CHLOROFORM-d | | | ***Spectrum Offset (Hz)*** | 15091.3428 |
| ***Sweep Width (Hz)*** | 47348.49 | ***Temperature (degree C)*** | 22.200 | | | | |

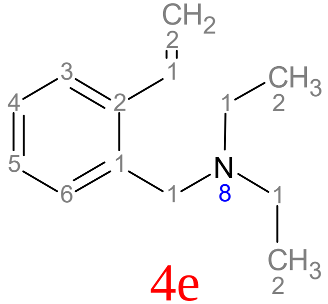

4e

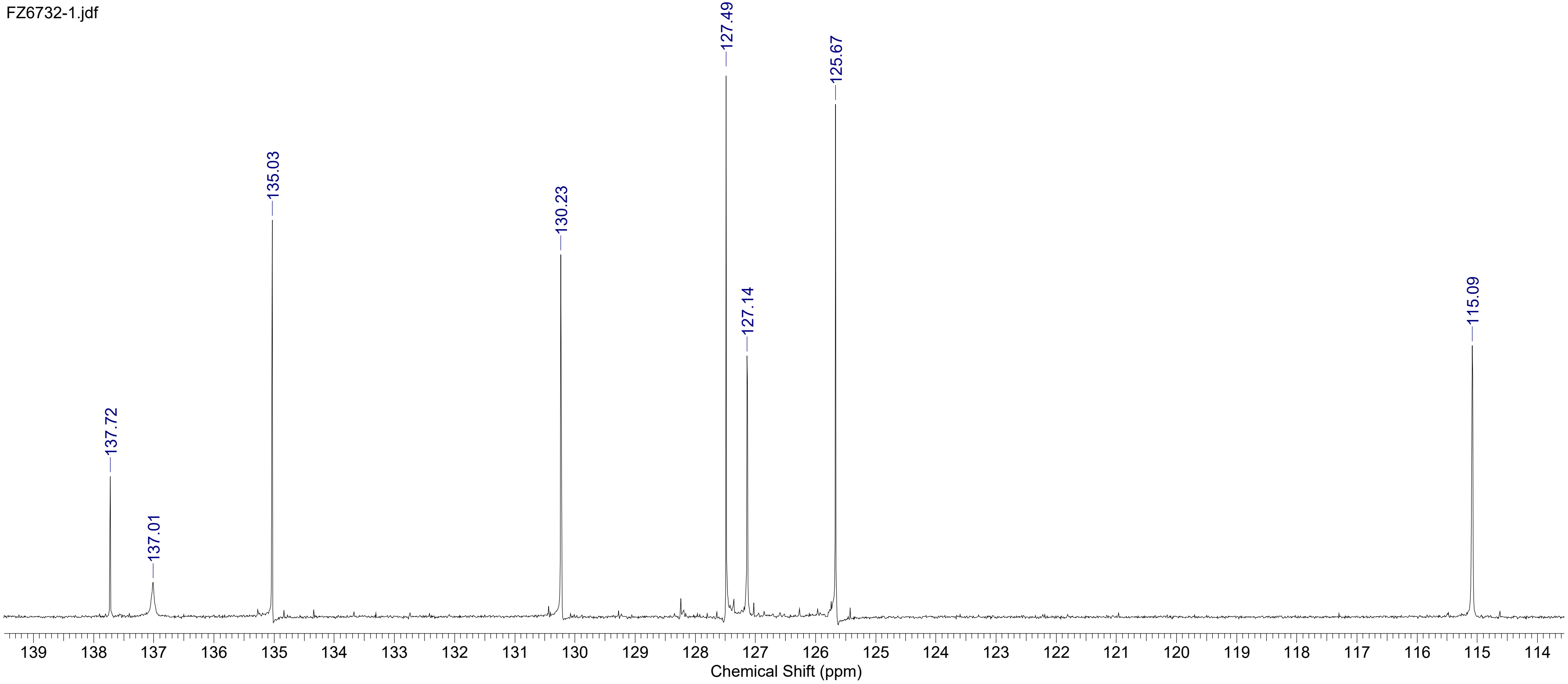

| | | | | | | | |
|---|---|---|---|---|---|---|---|
| ***Acquisition Time (sec)*** | 0.6921 | ***Comment*** | single pulse decoupled gated NOE | | | ***Date*** | 28 May 1990 00:10:35 |
| ***Date Stamp*** | 24 May 2018 04:57:18 | | | ***File Name*** | C:\Users\Fedor\Desktop\22.05.18\FZ6732-1.jdf | | |
| ***Frequency (MHz)*** | 150.91 | ***Nucleus*** | 13C | ***Number of Transients*** | 1000 | ***Origin*** | ECA 600 |
| ***Original Points Count*** | 32768 | ***Owner*** | delta | ***Points Count*** | 32768 | ***Pulse Sequence*** | single_pulse_dec |
| ***Receiver Gain*** | 52.00 | ***Solvent*** | CHLOROFORM-d | | | ***Spectrum Offset (Hz)*** | 15091.3428 |
| ***Sweep Width (Hz)*** | 47348.49 | ***Temperature (degree C)*** | 22.200 | | | | |

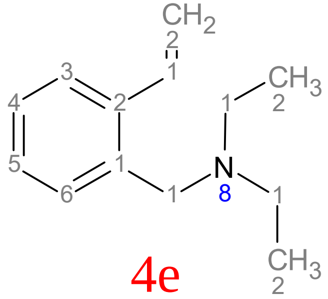


4e

FZ6732-1.jdf

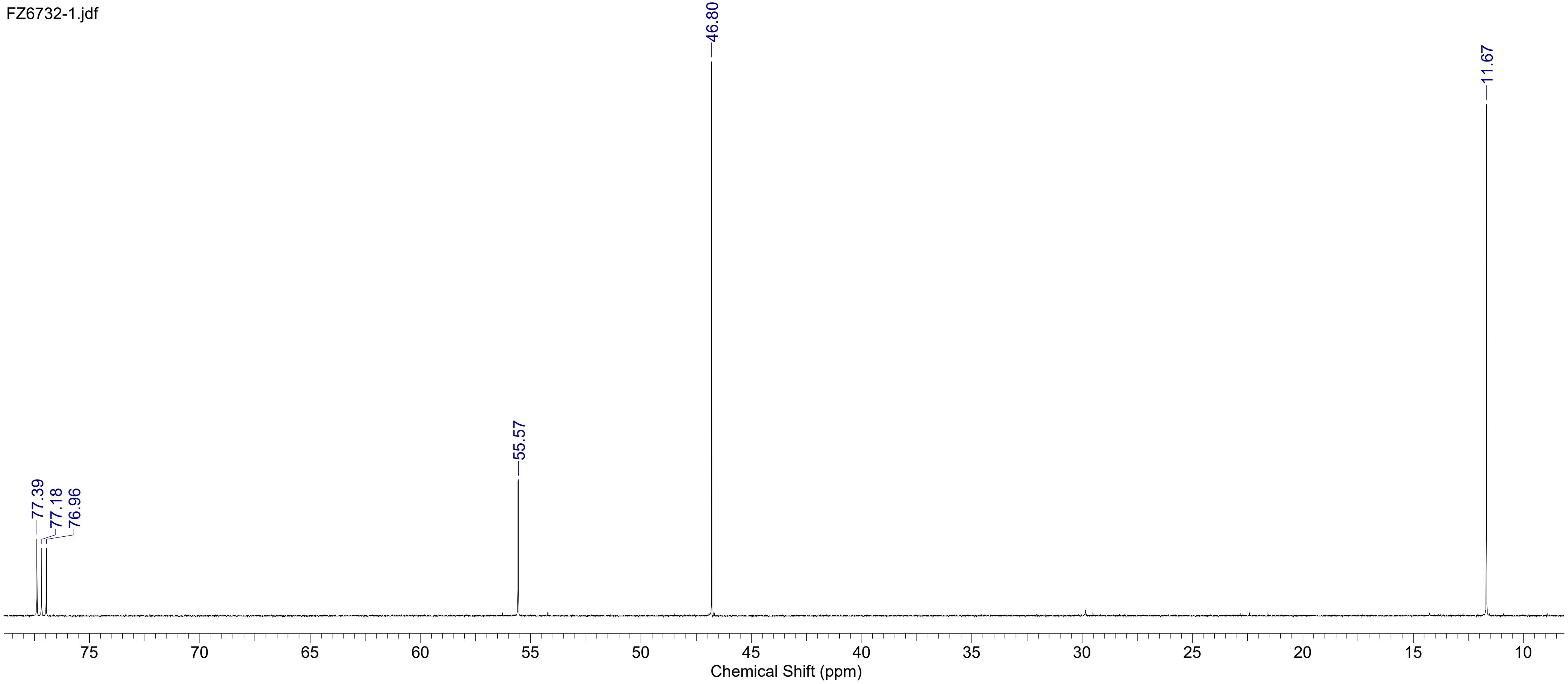

| Acquisition Time (sec) | 1.9818 | Comment | single_pulse | Date | 01 Mar 1990 00:08:24 | | | Date Stamp | 29 Aug 2018 09:38:17 |
|---|---|---|---|---|---|---|---|---|---|
| File Name | C:\Users\Fedor\Desktop\28.08.18\FZ6866-1.jdf | | | Frequency (MHz) | 600.17 | Nucleus | 1H | Number of Transients | 8 |
| Origin | ECA 600 | Original Points Count | 32768 | Owner | delta | Points Count | 32768 | Pulse Sequence | single_pulse.ex2 |
| Receiver Gain | 30.00 | Solvent | CHLOROFORM-d | | | Spectrum Offset (Hz) | 5401.5503 | Sweep Width (Hz) | 16534.39 |

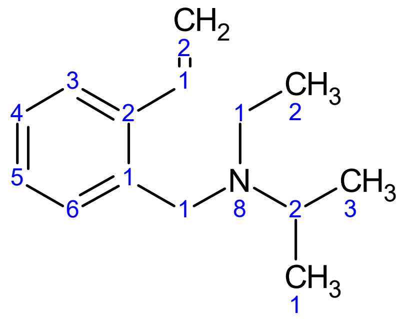


4f

FZ6866-1.jdf

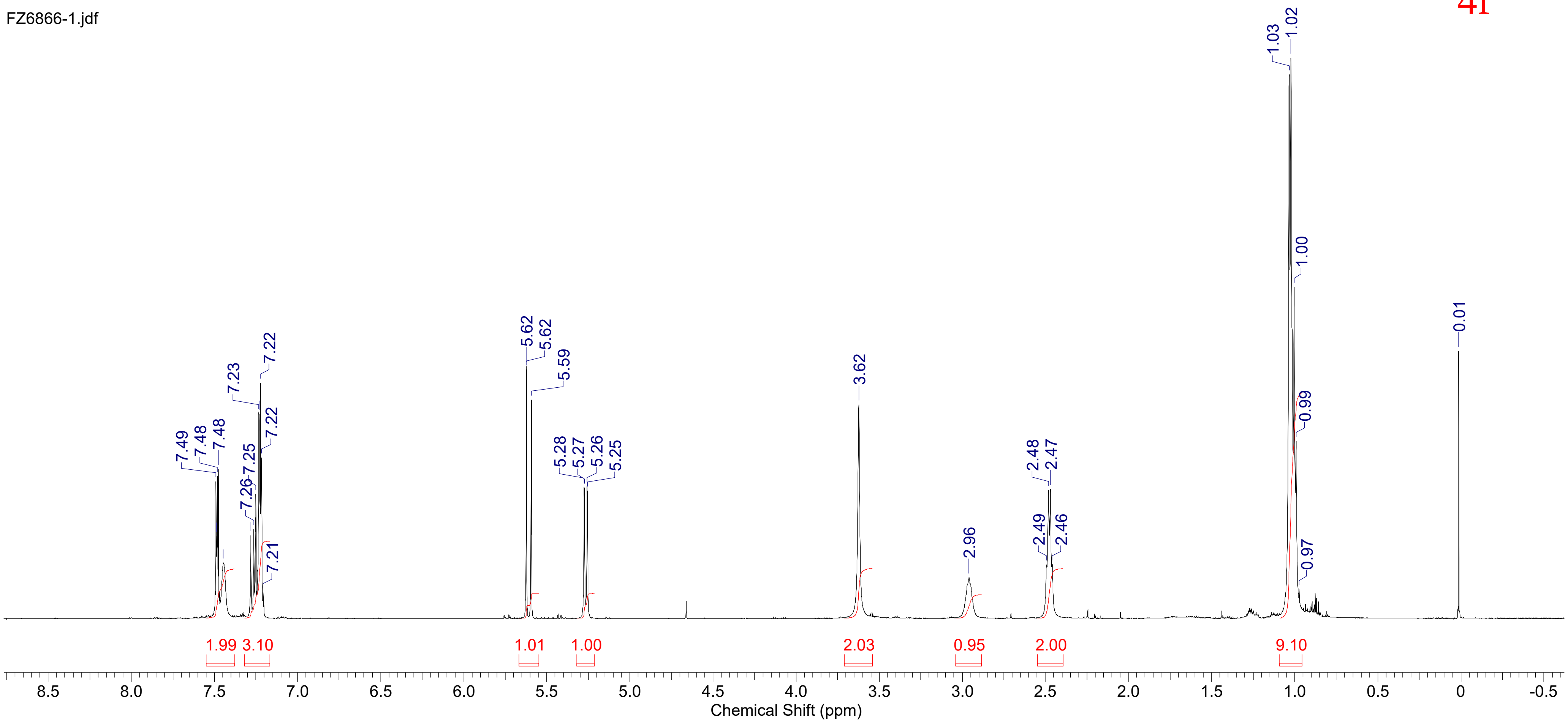

| Acquisition Time (sec) | 1.9818 | Comment | single_pulse | Date | 01 Mar 1990 00:08:24 | | | Date Stamp | 29 Aug 2018 09:38:17 |
|---|---|---|---|---|---|---|---|---|---|
| File Name | C:\Users\Fedor\Desktop\28.08.18\FZ6866-1.jdf | | | Frequency (MHz) | 600.17 | Nucleus | 1H | Number of Transients | 8 |
| Origin | ECA 600 | Original Points Count | 32768 | Owner | delta | Points Count | 32768 | Pulse Sequence | single_pulse.ex2 |
| Receiver Gain | 30.00 | Solvent | CHLOROFORM-d | | | Spectrum Offset (Hz) | 5401.5503 | Sweep Width (Hz) | 16534.39 |

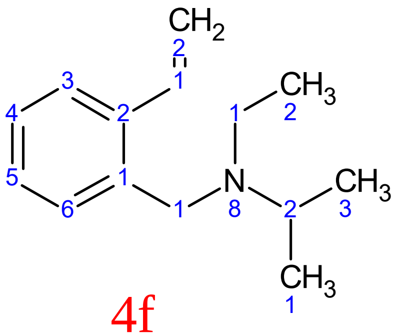

4f

FZ6866-1.jdf

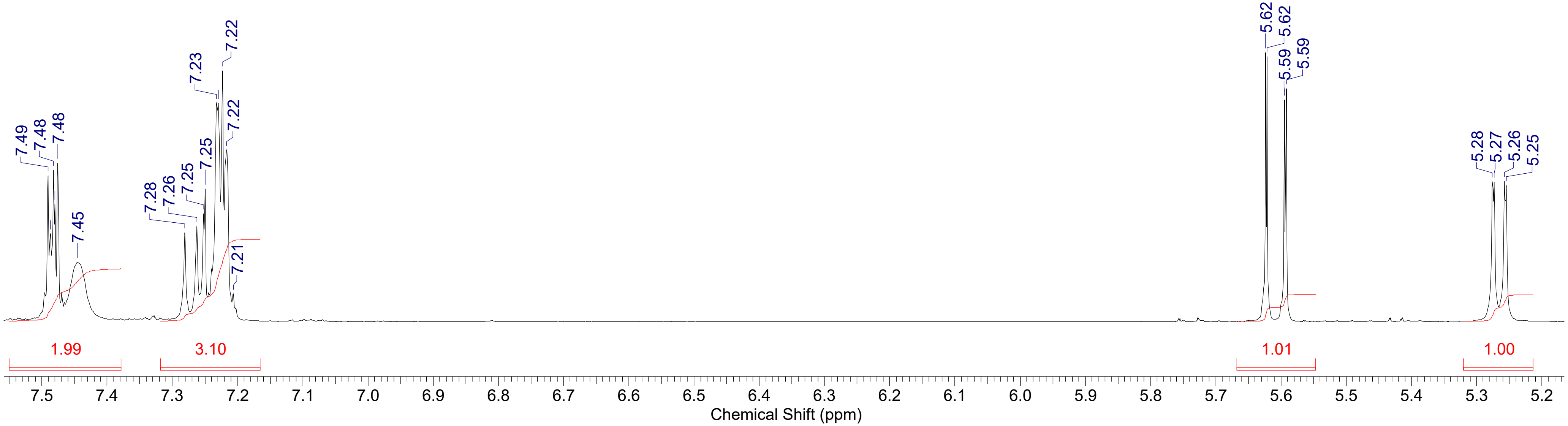

| | | | | | | | |
|---|---|---|---|---|---|---|---|
| ***Acquisition Time (sec)*** | 1.9818 | ***Comment*** | single_pulse | ***Date*** | 01 Mar 1990 00:08:24 | ***Date Stamp*** | 29 Aug 2018 09:38:17 |
| ***File Name*** | C:\Users\Fedor\Desktop\28.08.18\FZ6866-1.jdf | ***Frequency (MHz)*** | 600.17 | ***Nucleus*** | 1H | ***Number of Transients*** | 8 |
| ***Origin*** | ECA 600 | ***Original Points Count*** | 32768 | ***Owner*** | delta | ***Points Count*** | 32768 |
| ***Pulse Sequence*** | single_pulse.ex2 | ***Receiver Gain*** | 30.00 | ***Solvent*** | CHLOROFORM-d | ***Spectrum Offset (Hz)*** | 5401.5503 |
| ***Sweep Width (Hz)*** | 16534.39 | | | | | | |

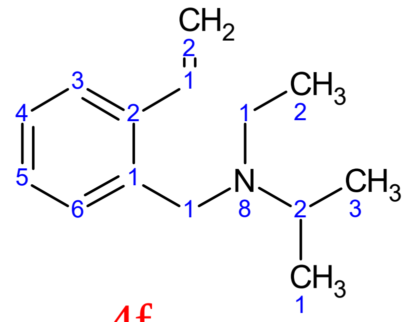


4f

FZ6866-1.jdf

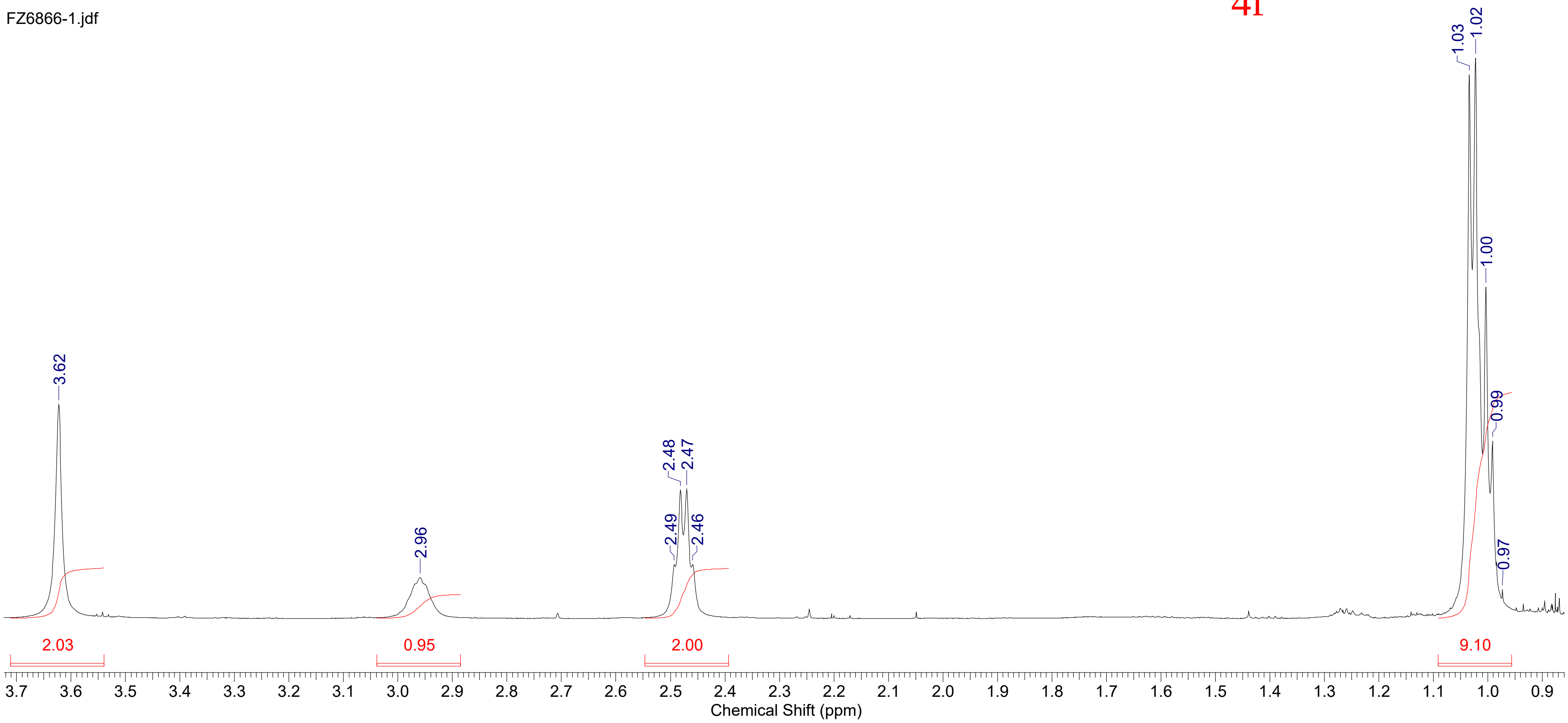

| Acquisition Time (sec) | 0.6921 | Comment | single pulse decoupled gated NOE | | Date | 16 Feb 1990 04:58:32 | | |
|---|---|---|---|---|---|---|---|---|
| Date Stamp | 15 Aug 2018 14:28:51 | | File Name | C:\Users\Fedor\Desktop\16.08.18\FZ6860-1.jdf | | | Frequency (MHz) | 150.91 |
| Nucleus | 13C | Number of Transients | 2000 | Origin | ECA 600 | Original Points Count | 32768 | Owner | delta |
| Points Count | 32768 | Pulse Sequence | single_pulse_dec | | Receiver Gain | 56.00 | Solvent | CHLOROFORM-d |
| Spectrum Offset (Hz) | 15091.3428 | Sweep Width (Hz) | 47348.49 | | | | | |

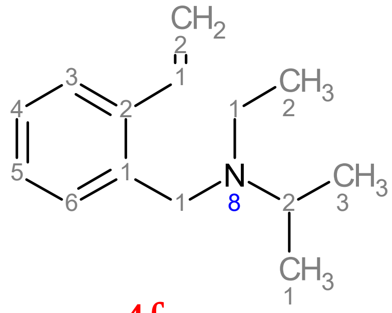

4f

FZ6860-1.jdf

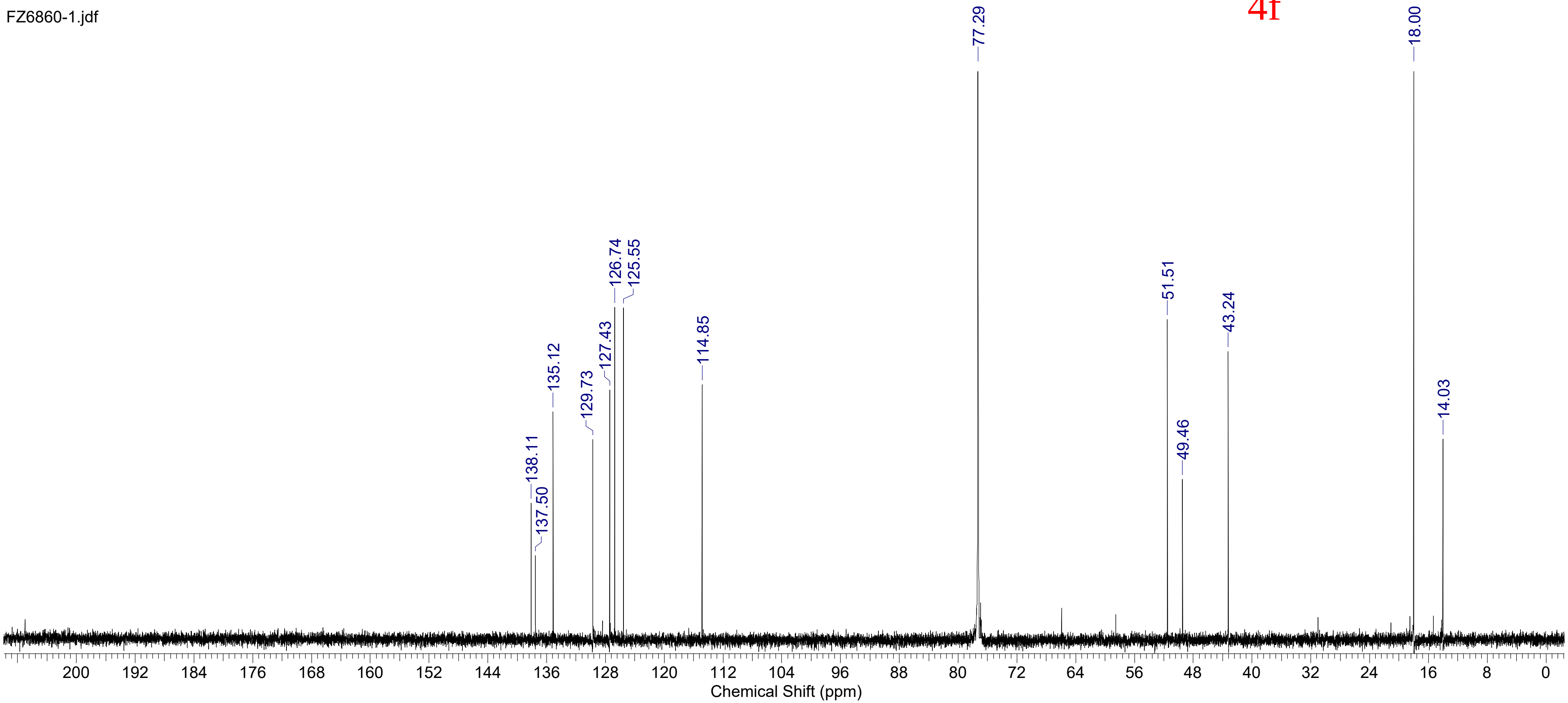

| Acquisition Time (sec) | 0.6921 | Comment | single pulse decoupled gated NOE | | | Date | 16 Feb 1990 04:58:32 | | |
|---|---|---|---|---|---|---|---|---|---|
| Date Stamp | 15 Aug 2018 14:28:51 | | | File Name | C:\Users\Fedor\Desktop\16.08.18\FZ6860-1.jdf | | | Frequency (MHz) | 150.91 |
| Nucleus | 13C | Number of Transients | 2000 | Origin | ECA 600 | Original Points Count | 32768 | Owner | delta |
| Points Count | 32768 | Pulse Sequence | single_pulse_dec | | | Receiver Gain | 56.00 | Solvent | CHLOROFORM-d |
| Spectrum Offset (Hz) | 15091.3428 | Sweep Width (Hz) | 47348.49 | | | | | | |

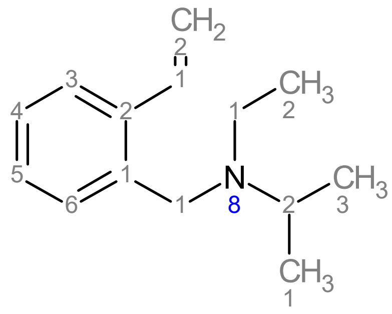


4f

FZ6860-1.jdf

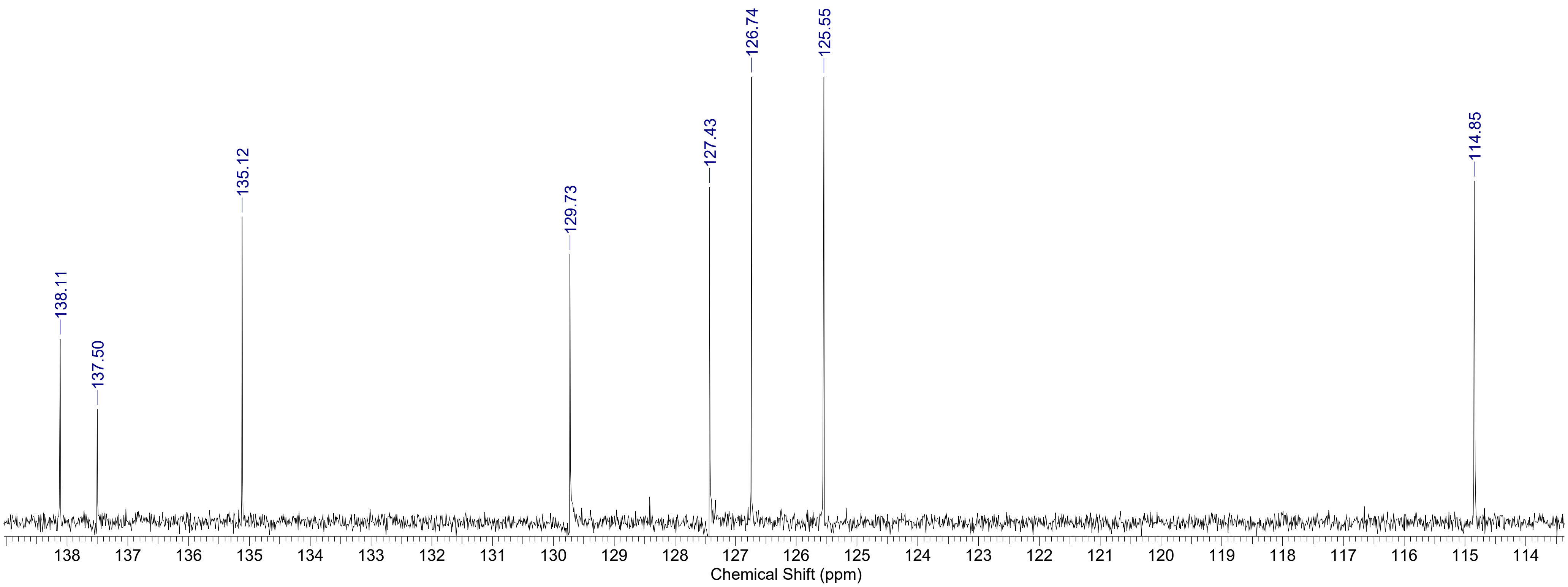

| | | | | | | | |
|---|---|---|---|---|---|---|---|
| **Acquisition Time (sec)** | 0.6921 | **Comment** | single pulse decoupled gated NOE | | **Date** | 16 Feb 1990 04:58:32 | |
| **Date Stamp** | 15 Aug 2018 14:28:51 | **File Name** | C:\Users\Fedor\Desktop\16.08.18\FZ6860-1.jdf | | **Frequency (MHz)** | 150.91 | |
| **Nucleus** | 13C | **Number of Transients** | 2000 | **Origin** | ECA 600 | **Original Points Count** | 32768 |
| **Owner** | delta | **Points Count** | 32768 | **Pulse Sequence** | single_pulse_dec | **Receiver Gain** | 56.00 |
| **Solvent** | CHLOROFORM-d | **Spectrum Offset (Hz)** | 15091.3428 | **Sweep Width (Hz)** | 47348.49 | | |

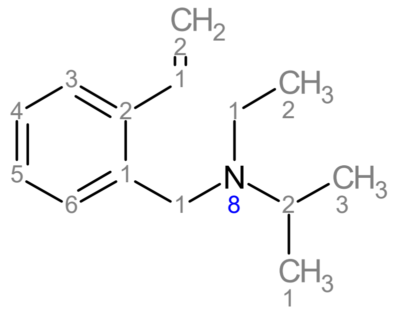

4f

FZ6860-1.jdf

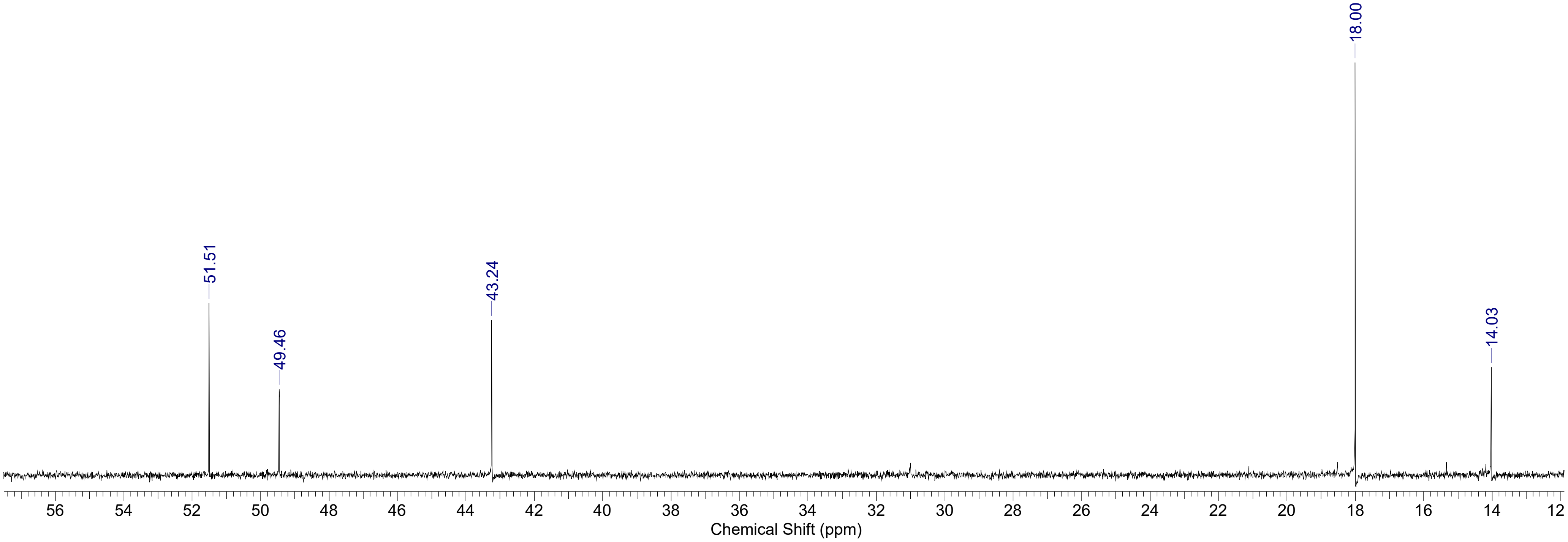

| | | | | | | | |
|---|---|---|---|---|---|---|---|
| ***Acquisition Time (sec)*** | 1.9818 | ***Comment*** | single_pulse | ***Date*** | 21 May 1990 06:44:21 | | |
| ***Date Stamp*** | 17 May 2018 11:31:05 | | | ***File Name*** | C:\Users\Fedor\Desktop\15.05.18\FZ6726-1.jdf | ***Frequency (MHz)*** | 600.17 |
| ***Nucleus*** | 1H | ***Number of Transients*** | 8 | ***Origin*** | ECA 600 | ***Original Points Count*** | 32768 |
| ***Owner*** | delta | | | | | | |
| ***Points Count*** | 32768 | ***Pulse Sequence*** | single_pulse.ex2 | ***Receiver Gain*** | 36.00 | ***Solvent*** | CHLOROFORM-d |
| ***Spectrum Offset (Hz)*** | 5401.5503 | ***Sweep Width (Hz)*** | 16534.39 | ***Temperature (degree C)*** | 19.700 | | |

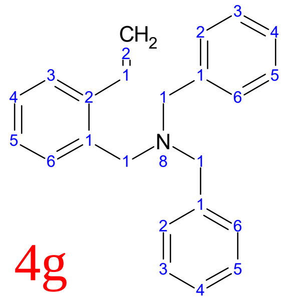


4g

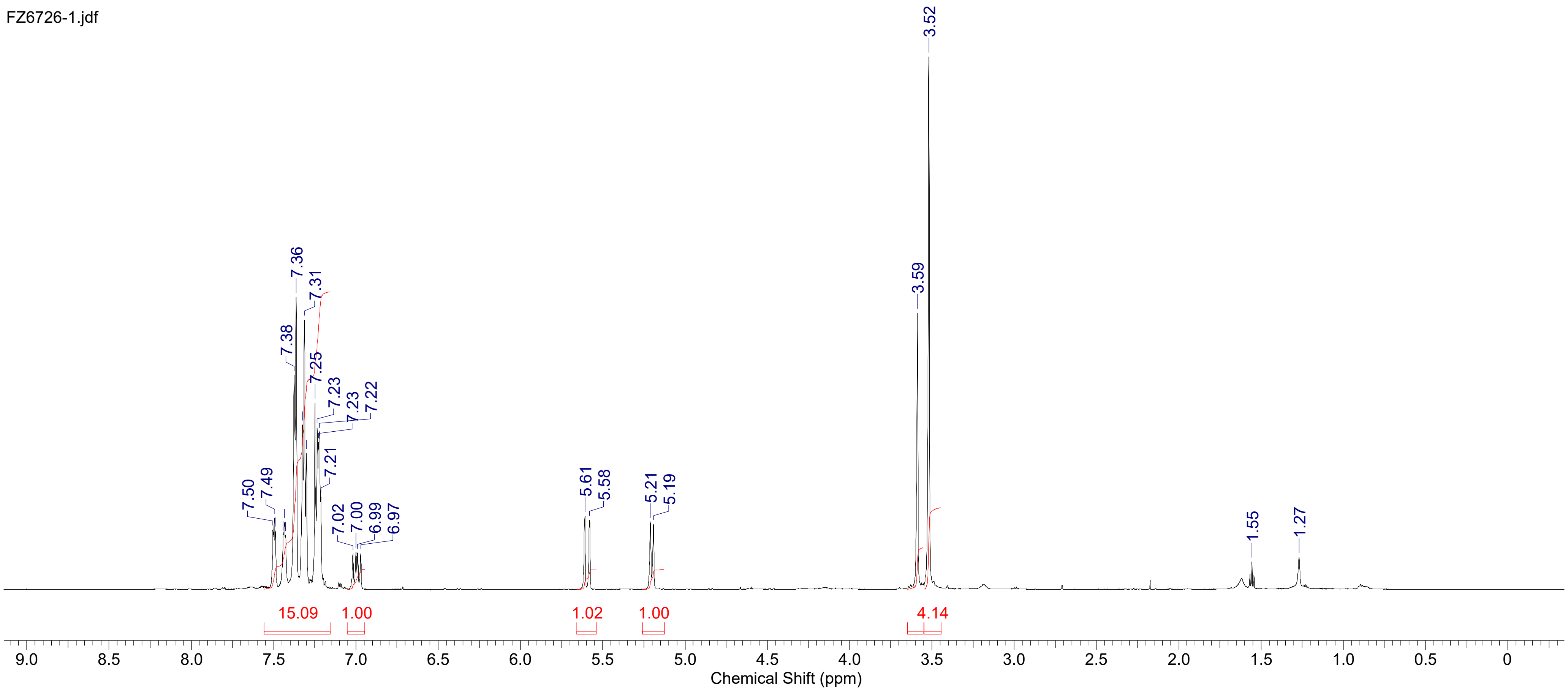

| | | | | | | | |
|---|---|---|---|---|---|---|---|
| ***Acquisition Time (sec)*** | 1.9818 | ***Comment*** | single_pulse | ***Date*** | 21 May 1990 06:44:21 | | |
| ***Date Stamp*** | 17 May 2018 11:31:05 | | | ***File Name*** | C:\Users\Fedor\Desktop\15.05.18\FZ6726-1.jdf | ***Frequency (MHz)*** | 600.17 |
| ***Nucleus*** | 1H | ***Number of Transients*** | 8 | ***Origin*** | ECA 600 | ***Original Points Count*** 32768 | ***Owner*** delta |
| ***Points Count*** | 32768 | ***Pulse Sequence*** | single_pulse.ex2 | | | ***Receiver Gain*** 36.00 | ***Solvent*** CHLOROFORM-d |
| ***Spectrum Offset (Hz)*** | 5401.5503 | ***Sweep Width (Hz)*** | 16534.39 | ***Temperature (degree C)*** | 19.700 | | |

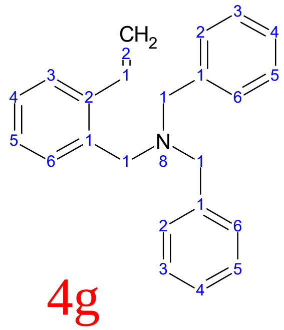

4g

FZ6726-1.jdf

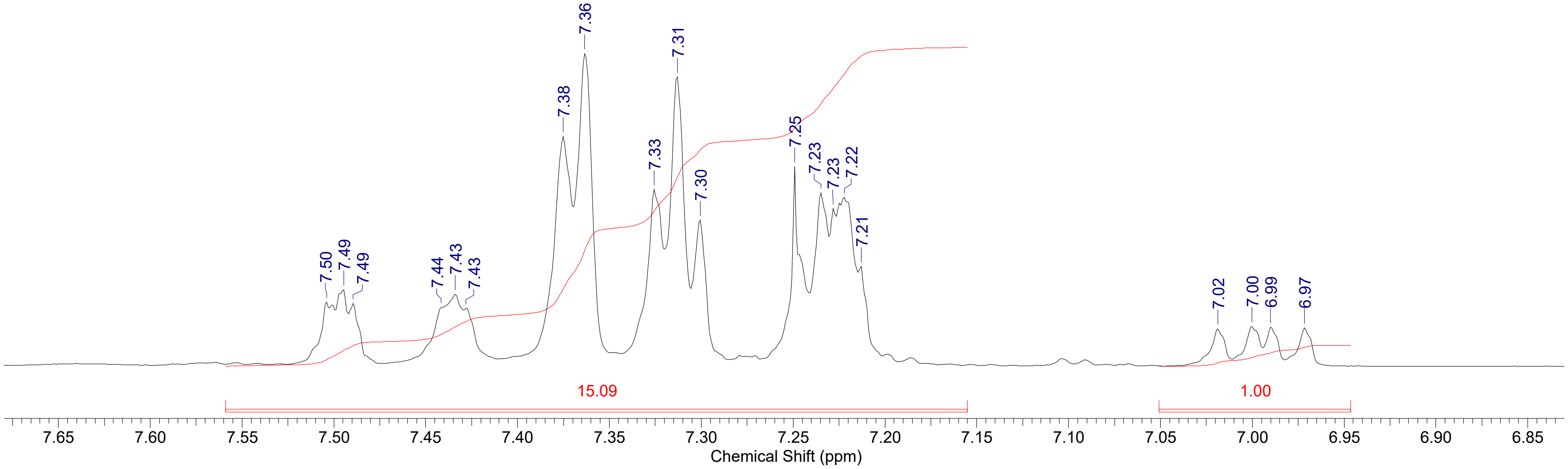

| | | | | | | | | | |
|---|---|---|---|---|---|---|---|---|---|
| ***Acquisition Time (sec)*** | 1.9818 | ***Comment*** | single_pulse | ***Date*** | 21 May 1990 06:44:21 | | | | |
| ***Date Stamp*** | 17 May 2018 11:31:05 | | | ***File Name*** | C:\Users\Fedor\Desktop\15.05.18\FZ6726-1.jdf | | | ***Frequency (MHz)*** | 600.17 |
| ***Nucleus*** | 1H | ***Number of Transients*** | 8 | ***Origin*** | ECA 600 | ***Original Points Count*** | 32768 | ***Owner*** | delta |
| ***Points Count*** | 32768 | ***Pulse Sequence*** | single_pulse.ex2 | | | ***Receiver Gain*** | 36.00 | ***Solvent*** | CHLOROFORM-d |
| ***Spectrum Offset (Hz)*** | 5401.5503 | ***Sweep Width (Hz)*** | 16534.39 | ***Temperature (degree C)*** | 19.700 | | | | |

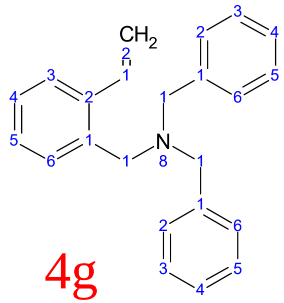


4g

FZ6726-1.jdf

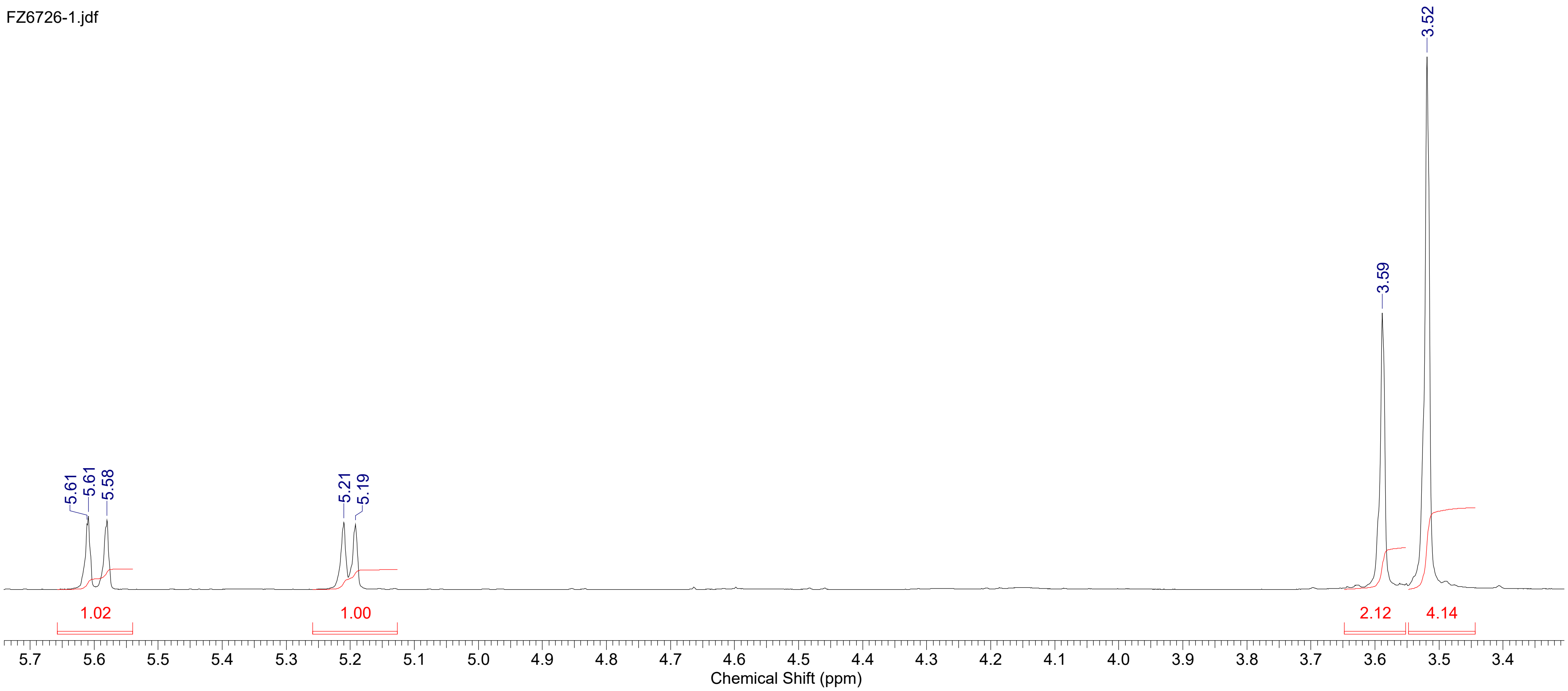

| | | | | | | | |
|---|---|---|---|---|---|---|---|
| ***Acquisition Time (sec)*** | 0.6921 | ***Comment*** | single pulse decoupled gated NOE | | | ***Date*** | 28 May 1990 05:20:52 |
| ***Date Stamp*** | 24 May 2018 10:07:34 | | | ***File Name*** | C:\Users\Fedor\Desktop\22.05.18\FZ6743-1.jdf | | |
| ***Frequency (MHz)*** | 150.91 | ***Nucleus*** | 13C | ***Number of Transients*** | 800 | ***Origin*** | ECA 600 |
| ***Original Points Count*** | 32768 | ***Owner*** | delta | ***Points Count*** | 32768 | ***Pulse Sequence*** | single_pulse_dec |
| ***Receiver Gain*** | 54.00 | ***Solvent*** | CHLOROFORM-d | | | ***Spectrum Offset (Hz)*** | 15091.3428 |
| ***Sweep Width (Hz)*** | 47348.49 | ***Temperature (degree C)*** | 22.000 | | | | |

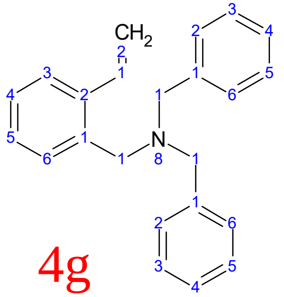


4g

FZ6743-1.jdf

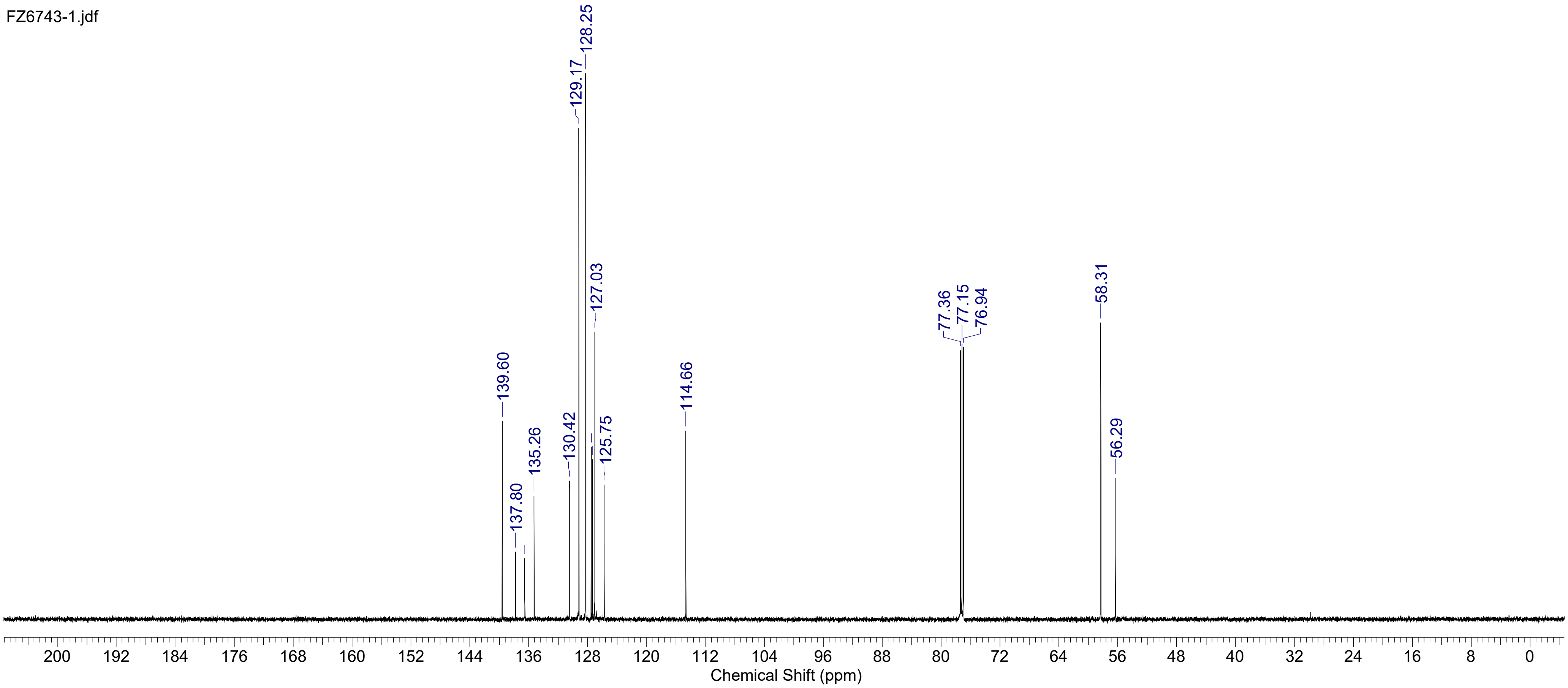

| | | | | | | | |
|---|---|---|---|---|---|---|---|
| ***Acquisition Time (sec)*** | 0.6921 | ***Comment*** | single pulse decoupled gated NOE | | | ***Date*** | 28 May 1990 05:20:52 |
| ***Date Stamp*** | 24 May 2018 10:07:34 | | | ***File Name*** | C:\Users\Fedor\Desktop\22.05.18\FZ6743-1.jdf | | |
| ***Frequency (MHz)*** | 150.91 | ***Nucleus*** | 13C | ***Number of Transients*** | 800 | ***Origin*** | ECA 600 |
| ***Original Points Count*** | 32768 | ***Owner*** | delta | ***Points Count*** | 32768 | ***Pulse Sequence*** | single_pulse_dec |
| ***Receiver Gain*** | 54.00 | ***Solvent*** | CHLOROFORM-d | | | ***Spectrum Offset (Hz)*** | 15091.3428 |
| ***Sweep Width (Hz)*** | 47348.49 | ***Temperature (degree C)*** | 22.000 | | | | |

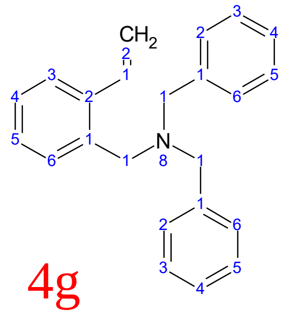


4g

FZ6743-1.jdf

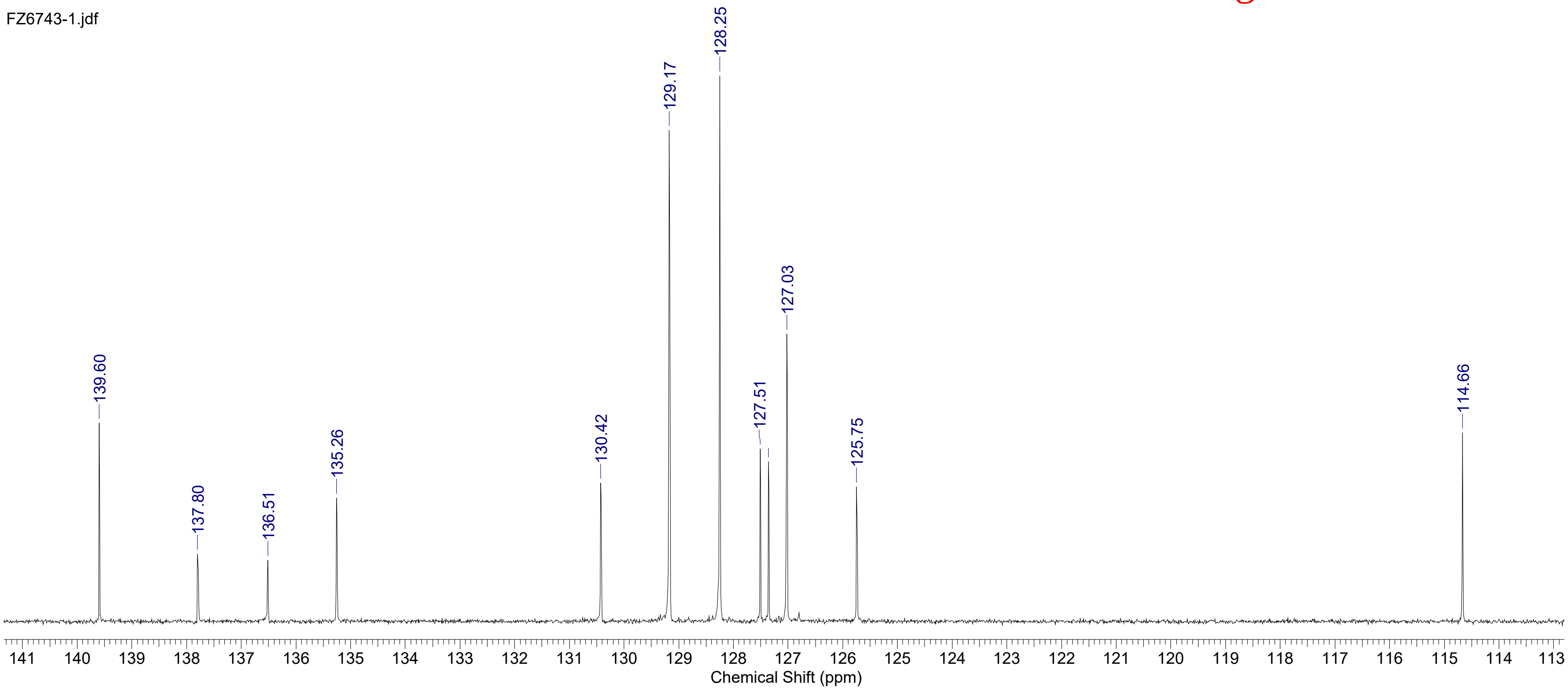

| | | | | | |
|---|---|---|---|---|---|
| ***Acquisition Time (sec)*** | 0.6921 | ***Comment*** | single pulse decoupled gated NOE | ***Date*** | 28 May 1990 05:20:52 |
| ***Date Stamp*** | 24 May 2018 10:07:34 | ***File Name*** | C:\Users\Fedor\Desktop\22.05.18\FZ6743-1.jdf | | |
| ***Frequency (MHz)*** | 150.91 | ***Nucleus*** | 13C | ***Number of Transients*** | 800 |
| ***Origin*** | ECA 600 | | | | |
| ***Original Points Count*** | 32768 | ***Owner*** | delta | ***Points Count*** | 32768 |
| ***Pulse Sequence*** | single_pulse_dec | | | | |
| ***Receiver Gain*** | 54.00 | ***Solvent*** | CHLOROFORM-d | ***Spectrum Offset (Hz)*** | 15091.3428 |
| ***Sweep Width (Hz)*** | 47348.49 | ***Temperature (degree C)*** | 22.000 | | |

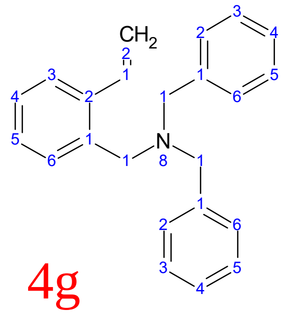


4g

FZ6743-1.jdf

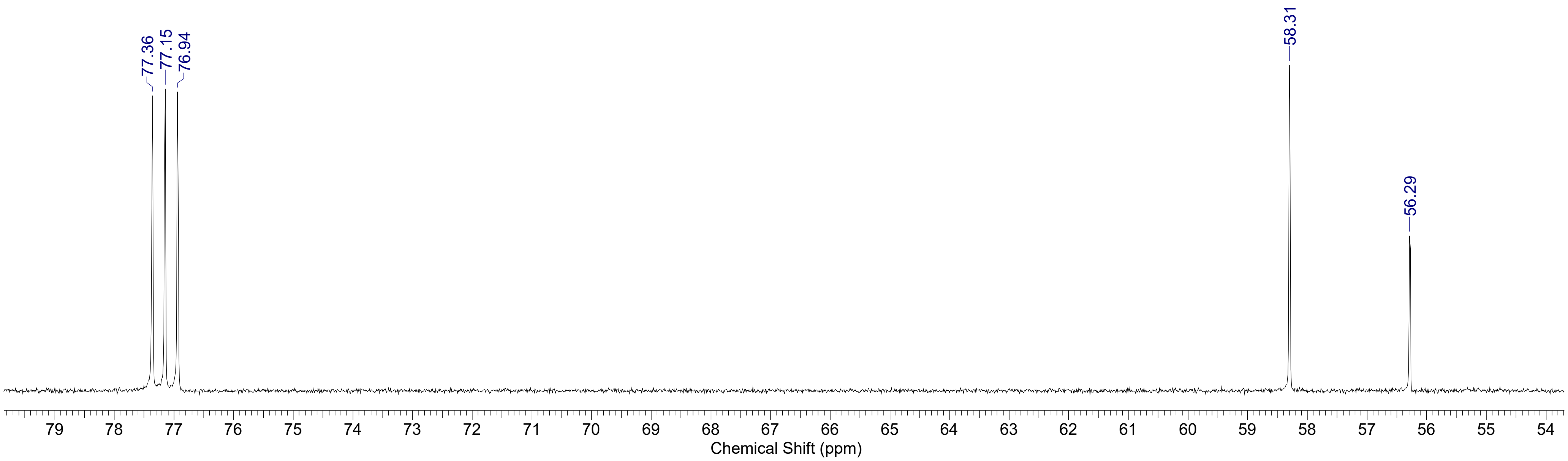

| Acquisition Time (sec) | 1.9818 | Comment | single_pulse | Date | 29 Apr 1990 07:51:18 | | |
|---|---|---|---|---|---|---|---|
| Date Stamp | 25 Apr 2018 12:38:26 | | | File Name | C:\Users\Fedor\Desktop\24.04.18\FZ6660-1.jdf | Frequency (MHz) | 600.17 |
| Nucleus | 1H | Number of Transients | 8 | Origin | ECA 600 | Original Points Count | 32768 |
| Owner | delta | | | | | | |
| Points Count | 32768 | Pulse Sequence | single_pulse.ex2 | | | Receiver Gain | 24.00 |
| Solvent | CHLOROFORM-d | | | | | | |
| Spectrum Offset (Hz) | 5401.5503 | Sweep Width (Hz) | 16534.39 | Temperature (degree C) | 21.600 | | |

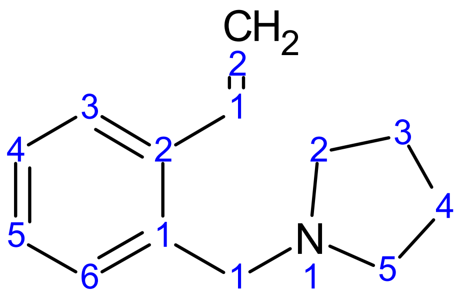

5a

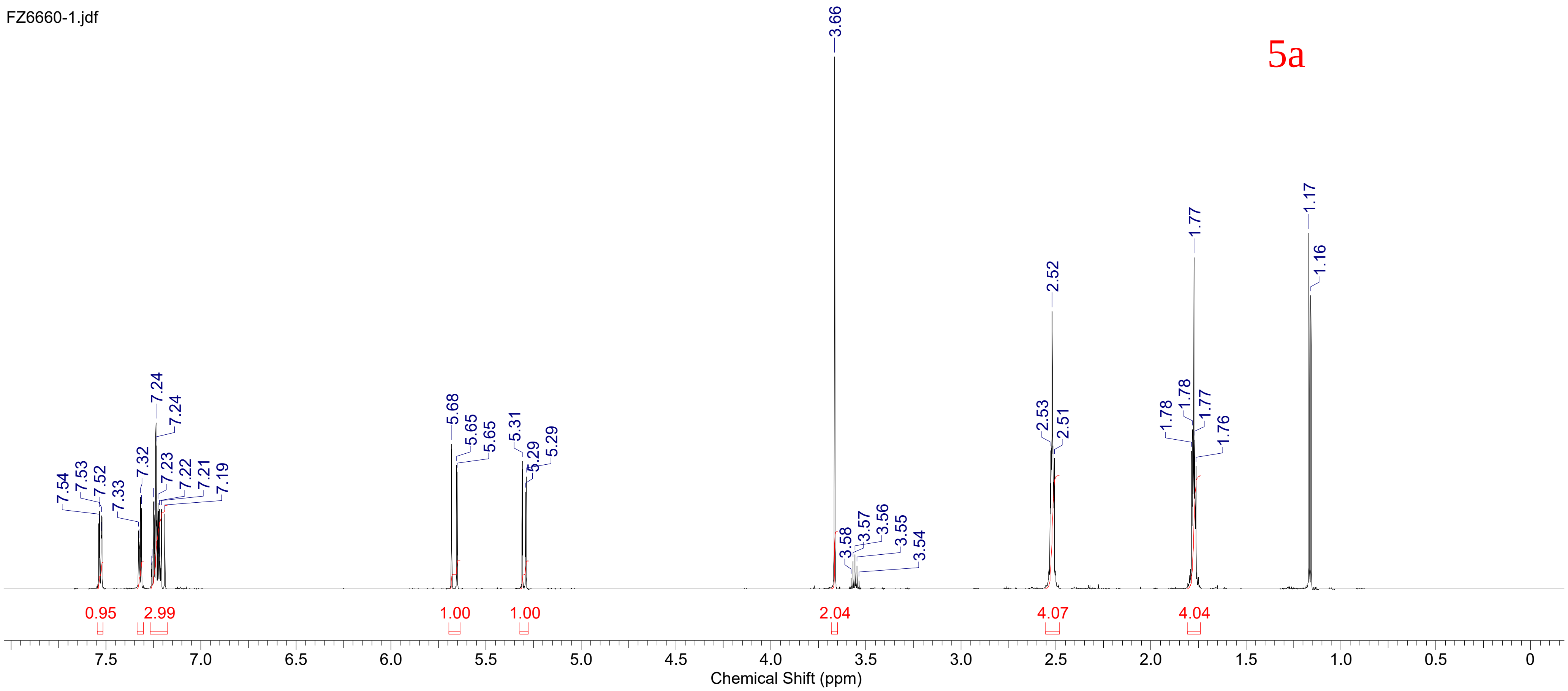

| | | | | | | | | | |
|---|---|---|---|---|---|---|---|---|---|
| ***Acquisition Time (sec)*** | 1.9818 | ***Comment*** | single_pulse | ***Date*** | 29 Apr 1990 07:51:18 | | | | |
| ***Date Stamp*** | 25 Apr 2018 12:38:26 | | | ***File Name*** | C:\Users\Fedor\Desktop\24.04.18\FZ6660-1.jdf | | | ***Frequency (MHz)*** | 600.17 |
| ***Nucleus*** | 1H | ***Number of Transients*** | 8 | ***Origin*** | ECA 600 | ***Original Points Count*** | 32768 | ***Owner*** | delta |
| ***Points Count*** | 32768 | ***Pulse Sequence*** | single_pulse.ex2 | | | ***Receiver Gain*** | 24.00 | ***Solvent*** | CHLOROFORM-d |
| ***Spectrum Offset (Hz)*** | 5401.5503 | ***Sweep Width (Hz)*** | 16534.39 | ***Temperature (degree C)*** | 21.600 | | | | |

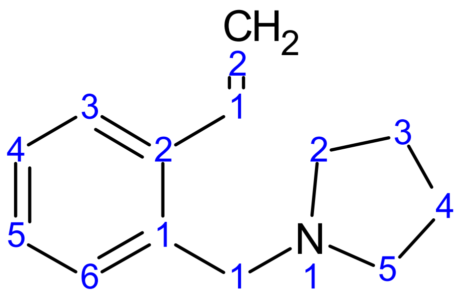


5a

FZ6660-1.jdf

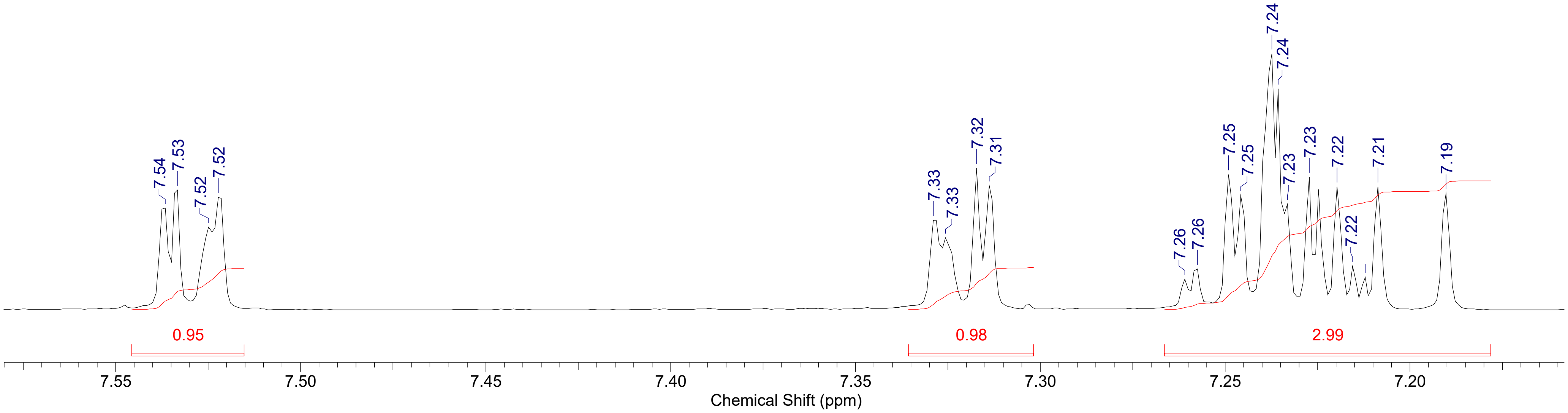

| | | | | | | | | | |
|---|---|---|---|---|---|---|---|---|---|
| ***Acquisition Time (sec)*** | 1.9818 | ***Comment*** | single_pulse | ***Date*** | 29 Apr 1990 07:51:18 | | | | |
| ***Date Stamp*** | 25 Apr 2018 12:38:26 | | | ***File Name*** | C:\Users\Fedor\Desktop\24.04.18\FZ6660-1.jdf | | | ***Frequency (MHz)*** | 600.17 |
| ***Nucleus*** | 1H | ***Number of Transients*** | 8 | ***Origin*** | ECA 600 | ***Original Points Count*** | 32768 | ***Owner*** | delta |
| ***Points Count*** | 32768 | ***Pulse Sequence*** | single_pulse.ex2 | | | ***Receiver Gain*** | 24.00 | ***Solvent*** | CHLOROFORM-d |
| ***Spectrum Offset (Hz)*** | 5401.5503 | ***Sweep Width (Hz)*** | 16534.39 | ***Temperature (degree C)*** | 21.600 | | | | |

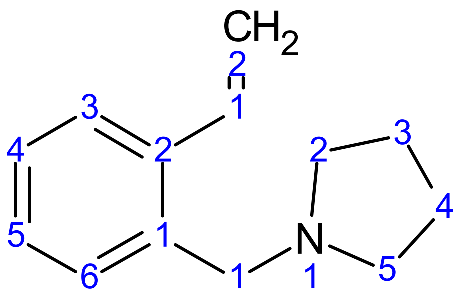


5a

FZ6660-1.jdf

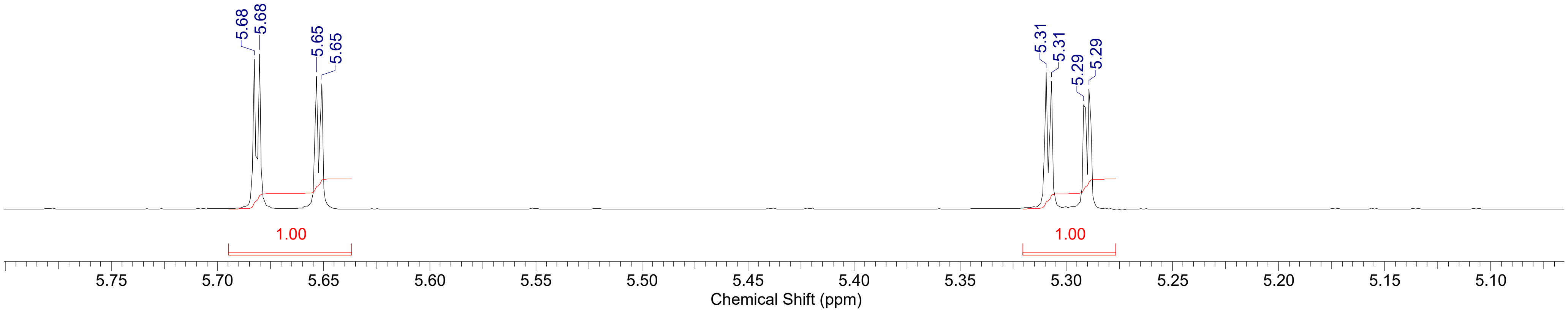

| | | | | | | | |
|---|---|---|---|---|---|---|---|
| ***Acquisition Time (sec)*** | 1.9818 | ***Comment*** | single_pulse | ***Date*** | 29 Apr 1990 07:51:18 | | |
| ***Date Stamp*** | 25 Apr 2018 12:38:26 | | | ***File Name*** | C:\Users\Fedor\Desktop\24.04.18\FZ6660-1.jdf | ***Frequency (MHz)*** | 600.17 |
| ***Nucleus*** | 1H | ***Number of Transients*** | 8 | ***Origin*** | ECA 600 | ***Original Points Count*** | 32768 |
| ***Owner*** | delta | | | | | | |
| ***Points Count*** | 32768 | ***Pulse Sequence*** | single_pulse.ex2 | ***Receiver Gain*** | 24.00 | ***Solvent*** | CHLOROFORM-d |
| ***Spectrum Offset (Hz)*** | 5401.5503 | ***Sweep Width (Hz)*** | 16534.39 | ***Temperature (degree C)*** | 21.600 | | |

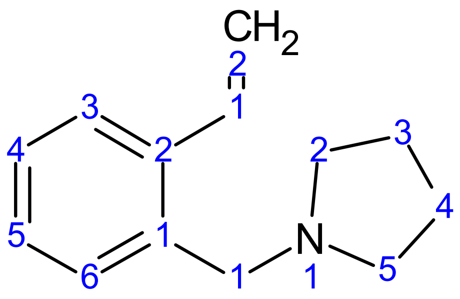


5a

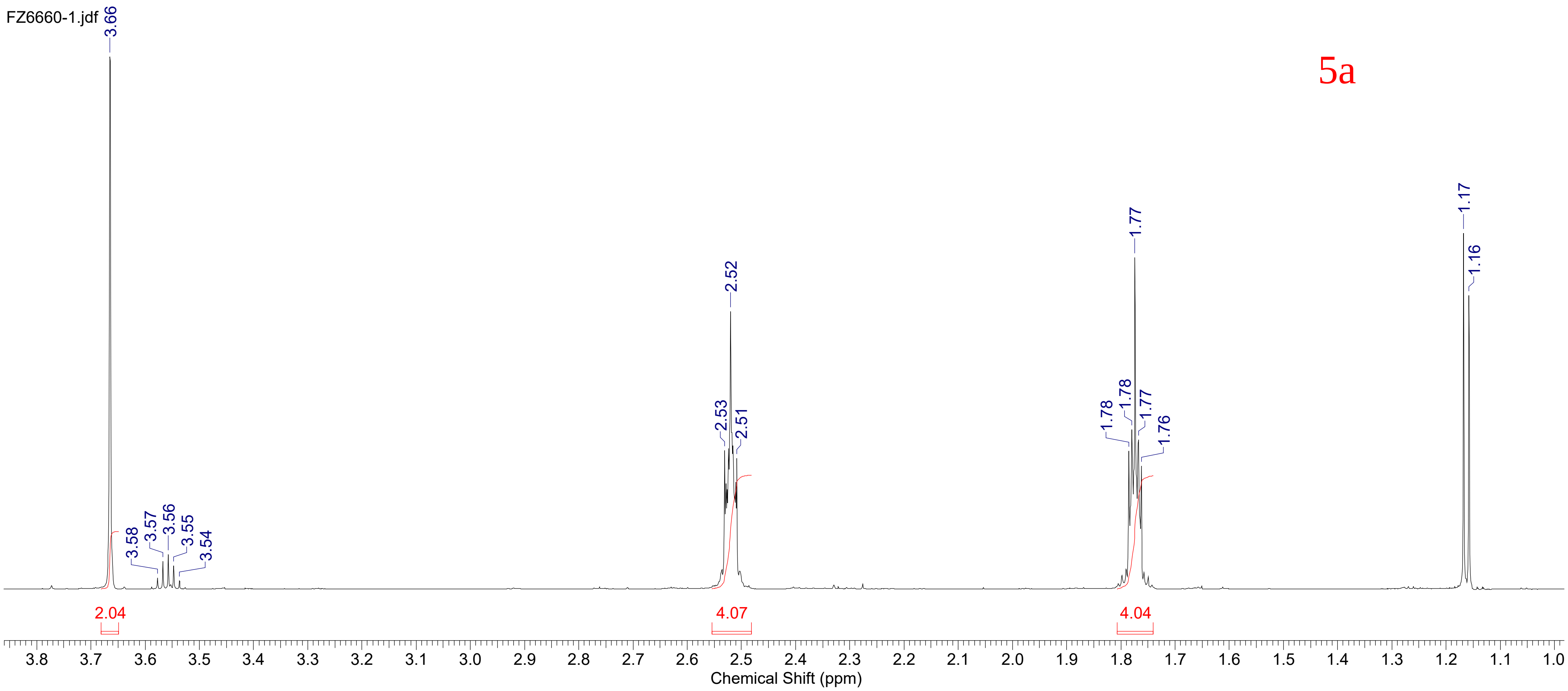

| | | | | | | | |
|---|---|---|---|---|---|---|---|
| ***Acquisition Time (sec)*** | 0.6921 | ***Comment*** | single pulse decoupled gated NOE | | | ***Date*** | 27 May 1990 17:44:26 |
| ***Date Stamp*** | 24 May 2018 04:18:54 | | | ***File Name*** | C:\Users\Fedor\Desktop\22.05.18\FZ6731-1.jdf | | |
| ***Frequency (MHz)*** | 150.91 | ***Nucleus*** | 13C | ***Number of Transients*** | 1000 | ***Origin*** | ECA 600 |
| ***Original Points Count*** | 32768 | ***Owner*** | delta | ***Points Count*** | 32768 | ***Pulse Sequence*** | single_pulse_dec |
| ***Receiver Gain*** | 54.00 | ***Solvent*** | CHLOROFORM-d | | | ***Spectrum Offset (Hz)*** | 15091.3428 |
| ***Sweep Width (Hz)*** | 47348.49 | ***Temperature (degree C)*** | 22.200 | | | | |

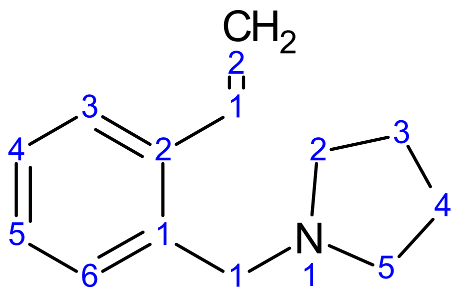


5a

FZ6731-1.jdf

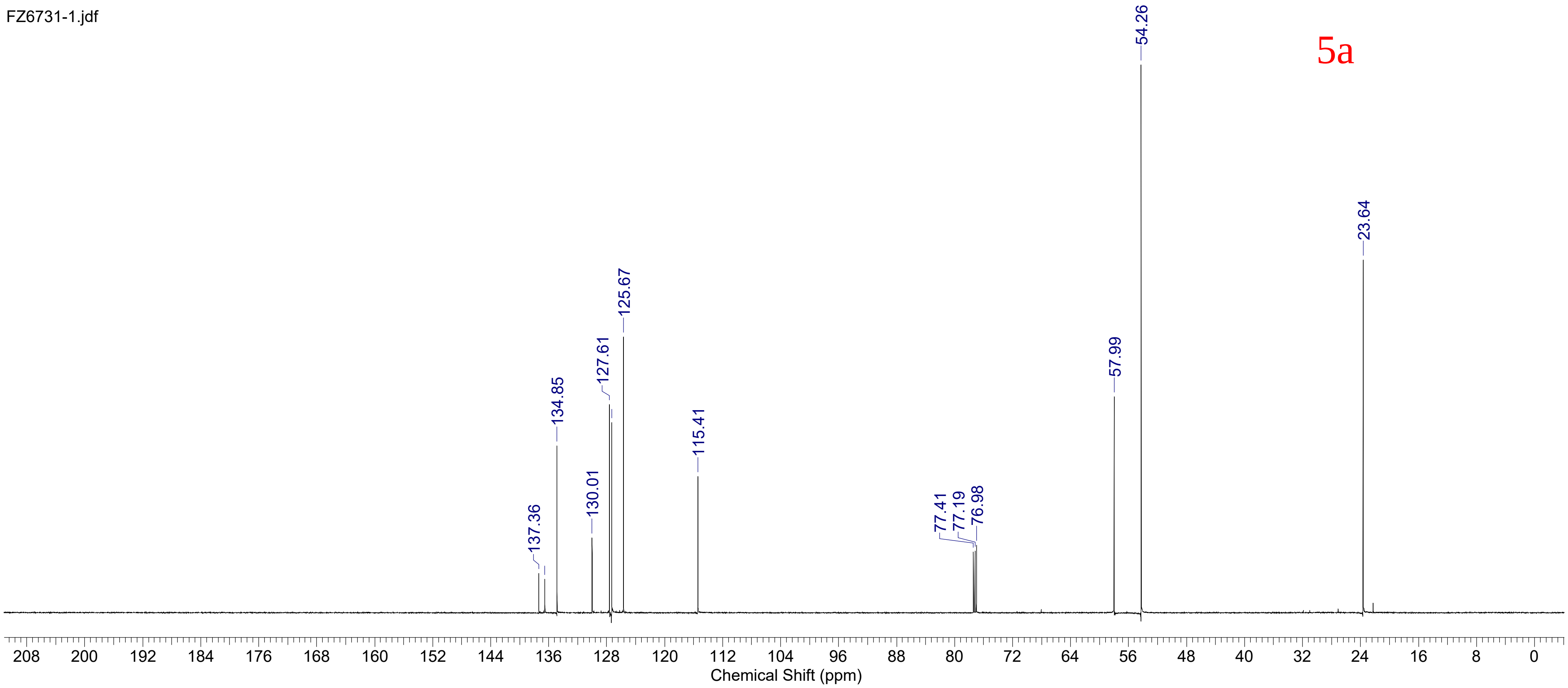

| | | | | | | | |
|---|---|---|---|---|---|---|---|
| ***Acquisition Time (sec)*** | 0.6921 | ***Comment*** | single pulse decoupled gated NOE | | | ***Date*** | 27 May 1990 17:44:26 |
| ***Date Stamp*** | 24 May 2018 04:18:54 | | | ***File Name*** | C:\Users\Fedor\Desktop\22.05.18\FZ6731-1.jdf | | |
| ***Frequency (MHz)*** | 150.91 | ***Nucleus*** | 13C | ***Number of Transients*** | 1000 | ***Origin*** | ECA 600 |
| ***Original Points Count*** | 32768 | ***Owner*** | delta | ***Points Count*** | 32768 | ***Pulse Sequence*** | single_pulse_dec |
| ***Receiver Gain*** | 54.00 | ***Solvent*** | CHLOROFORM-d | | | ***Spectrum Offset (Hz)*** | 15091.3428 |
| ***Sweep Width (Hz)*** | 47348.49 | ***Temperature (degree C)*** | 22.200 | | | | |

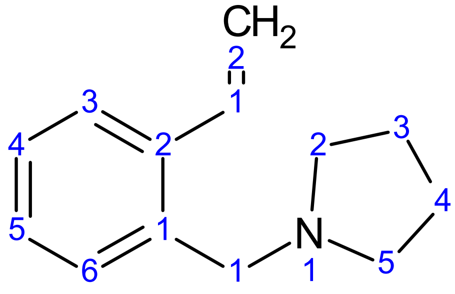


5a

FZ6731-1.jdf

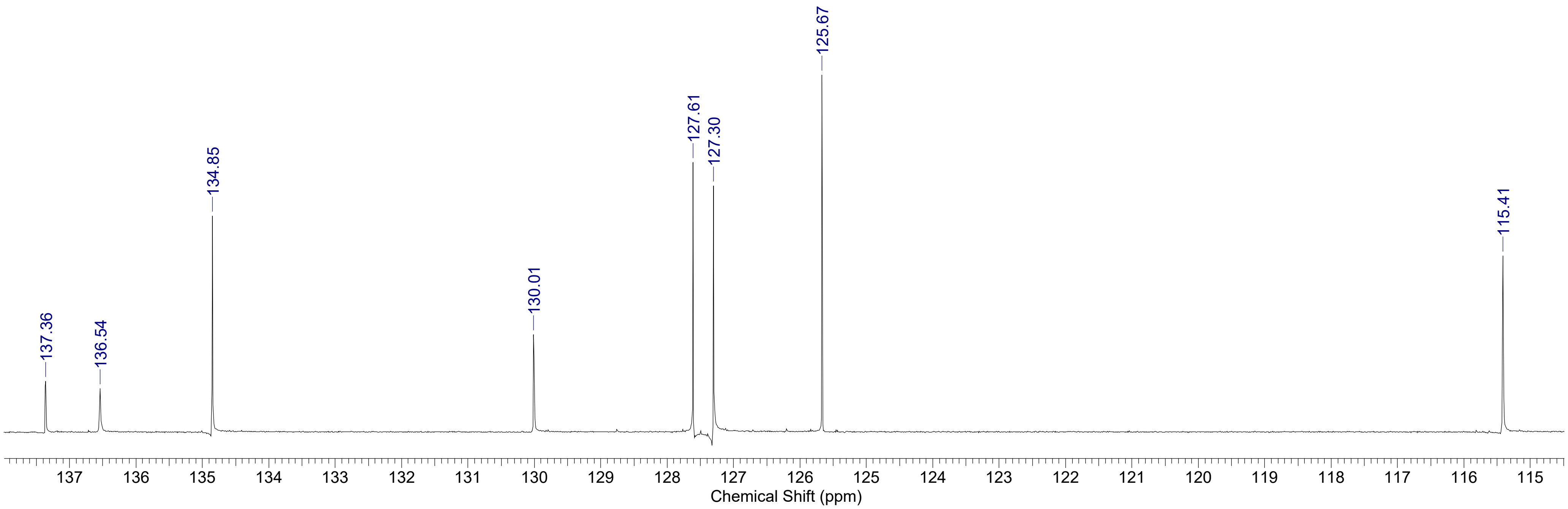

| | | | | | |
|---|---|---|---|---|---|
| ***Acquisition Time (sec)*** | 0.6921 | ***Comment*** | single pulse decoupled gated NOE | ***Date*** | 27 May 1990 17:44:26 |
| ***Date Stamp*** | 24 May 2018 04:18:54 | ***File Name*** | C:\Users\Fedor\Desktop\22.05.18\FZ6731-1.jdf | | |
| ***Frequency (MHz)*** | 150.91 | ***Nucleus*** | 13C | ***Number of Transients*** | 1000 |
| ***Origin*** | ECA 600 | | | | |
| ***Original Points Count*** | 32768 | ***Owner*** | delta | ***Points Count*** | 32768 |
| ***Pulse Sequence*** | single_pulse_dec | | | | |
| ***Receiver Gain*** | 54.00 | ***Solvent*** | CHLOROFORM-d | ***Spectrum Offset (Hz)*** | 15091.3428 |
| ***Sweep Width (Hz)*** | 47348.49 | ***Temperature (degree C)*** | 22.200 | | |

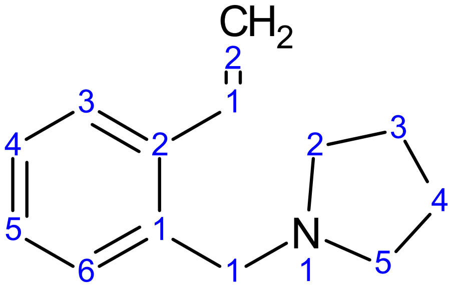


5a

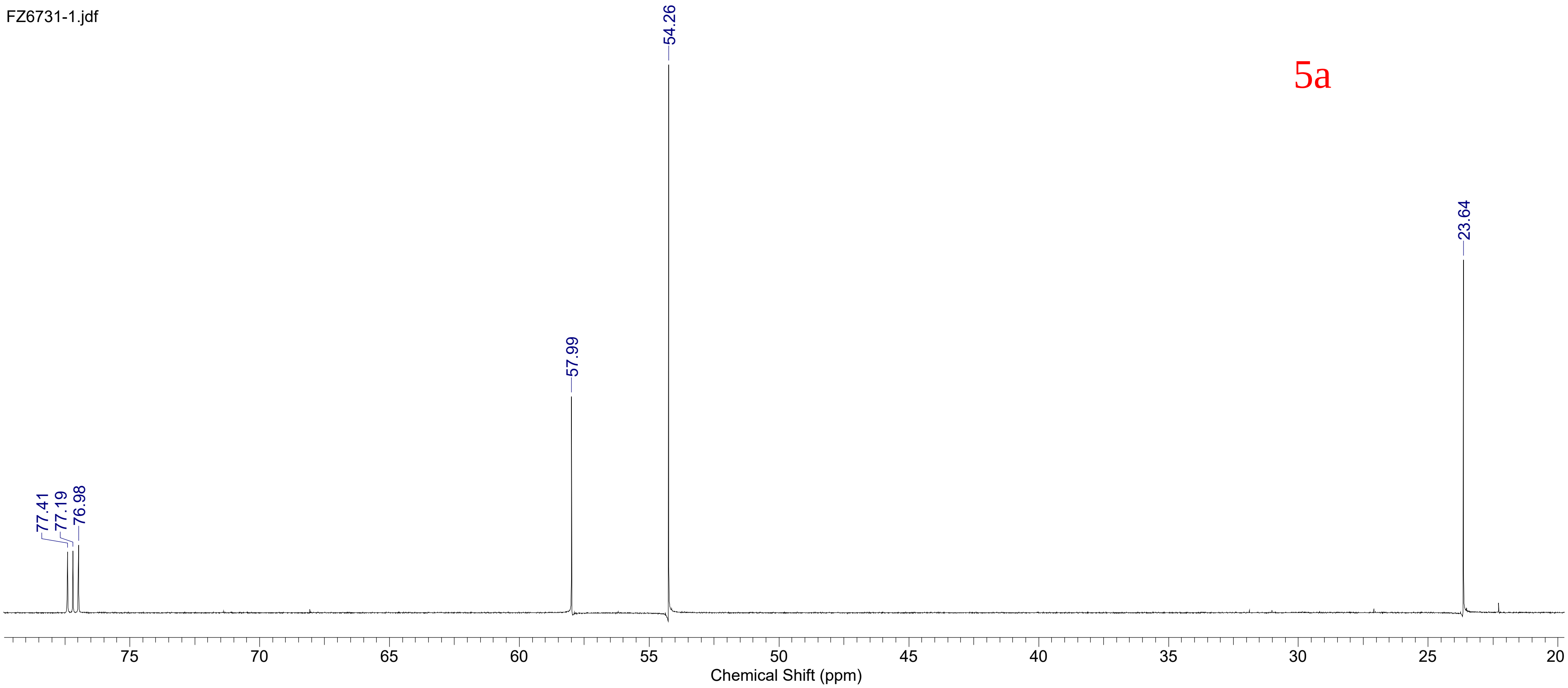

| | | | | | | | |
|---|---|---|---|---|---|---|---|
| ***Acquisition Time (sec)*** | 1.9818 | ***Comment*** | single_pulse | ***Date*** | 09 Feb 1990 03:52:47 | ***Date Stamp*** | 08 Aug 2018 13:22:59 |
| ***File Name*** | C:\USERS\FEDOR\DESKTOP\08.08.18\FZ6851-1.JDF | ***Frequency (MHz)*** | 600.17 | ***Nucleus*** | 1H | ***Number of Transients*** | 8 |
| ***Origin*** | ECA 600 | ***Original Points Count*** | 32768 | ***Owner*** | delta | ***Points Count*** | 32768 | 
| ***Pulse Sequence*** | single_pulse.ex2 | ***Receiver Gain*** | 24.00 | ***Solvent*** | CHLOROFORM-d | ***Spectrum Offset (Hz)*** | 5385.9019 |
| ***Sweep Width (Hz)*** | 16534.39 | | | | | | |

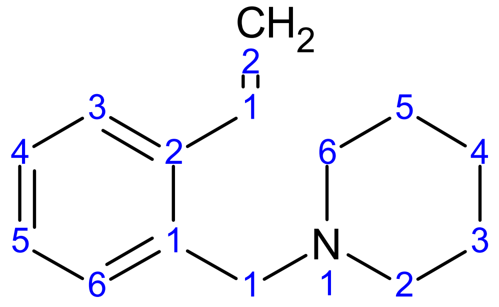

5b

FZ6851-1.JDF

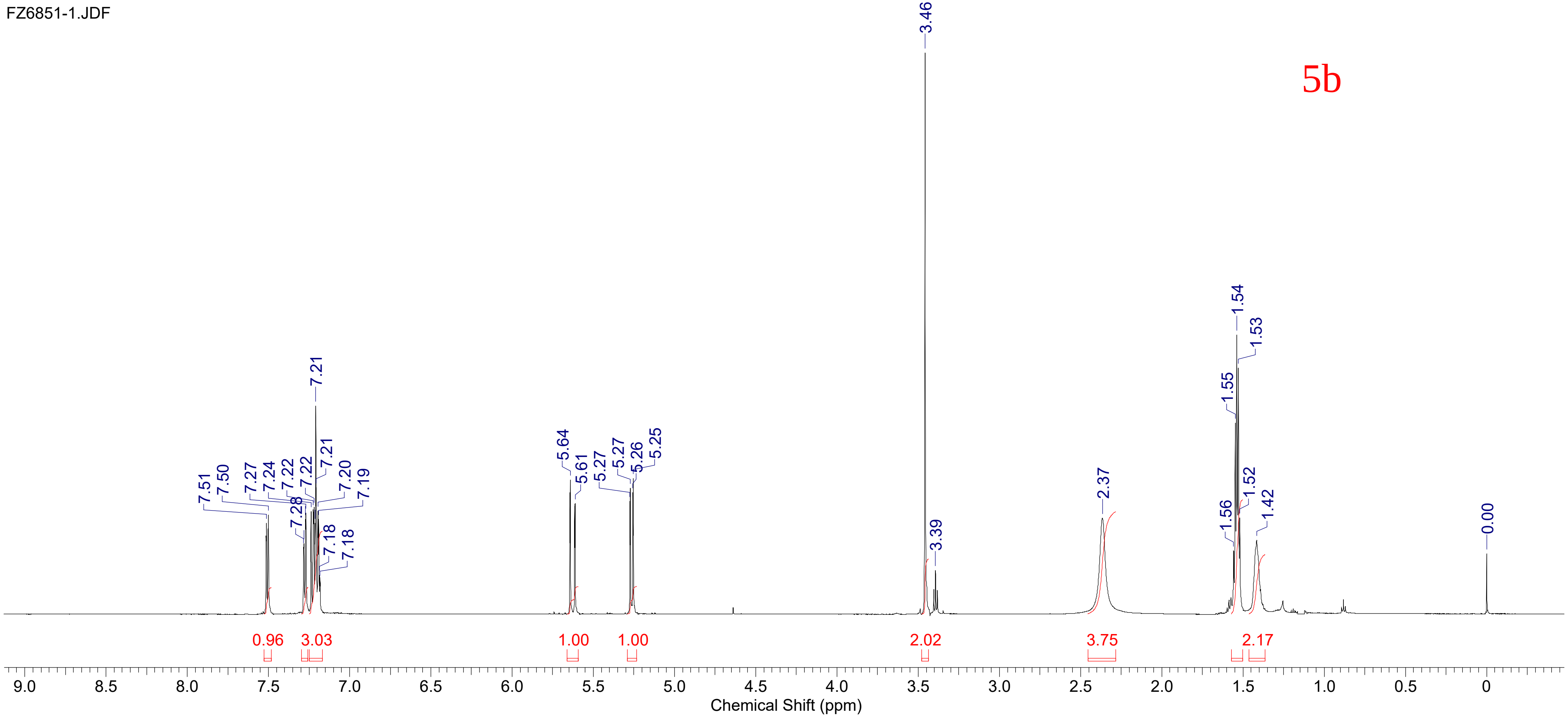

| Acquisition Time (sec) | 1.9818 | Comment | single_pulse | Date | 09 Feb 1990 03:52:47 | | | Date Stamp | 08 Aug 2018 13:22:59 |
|---|---|---|---|---|---|---|---|---|---|
| File Name | C:\USERS\FEDOR\DESKTOP\08.08.18\FZ6851-1.JDF | | | Frequency (MHz) | 600.17 | Nucleus | 1H | Number of Transients | 8 |
| Origin | ECA 600 | Original Points Count | 32768 | Owner | delta | Points Count | 32768 | Pulse Sequence | single_pulse.ex2 |
| Receiver Gain | 24.00 | Solvent | CHLOROFORM-d | | | Spectrum Offset (Hz) | 5385.9019 | Sweep Width (Hz) | 16534.39 |

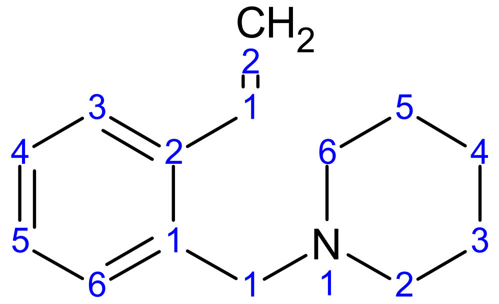

FZ6851-1.JDF

5b

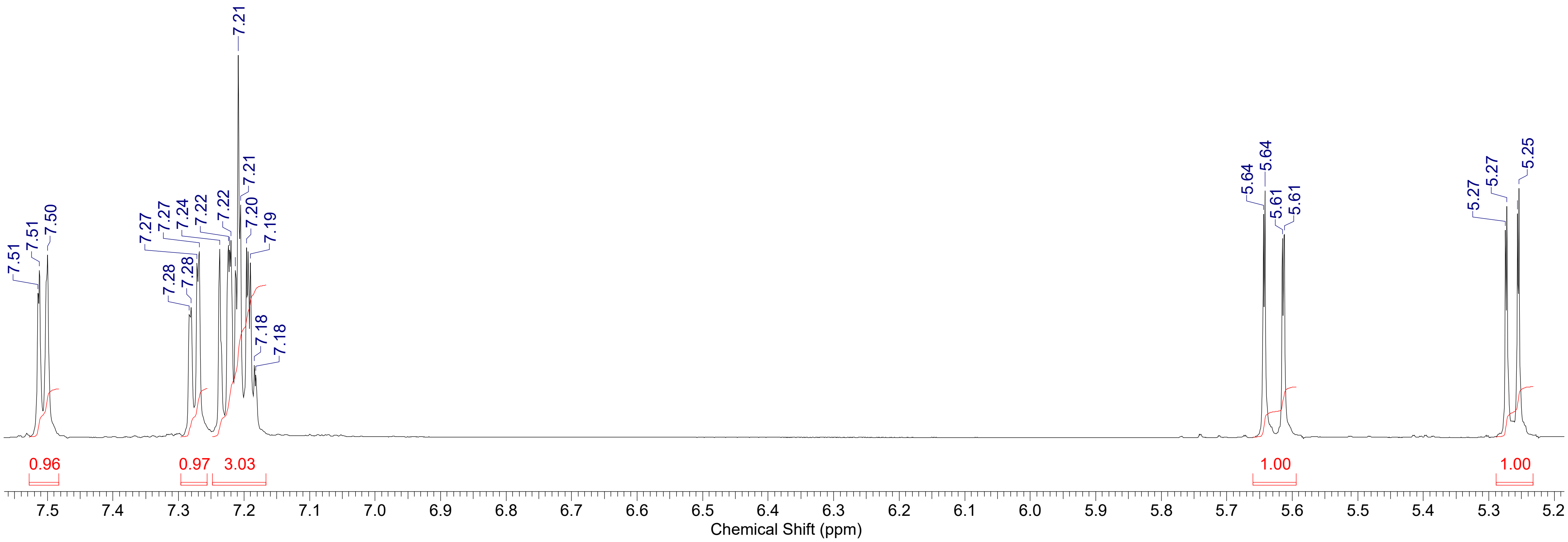

| | | | | | | | | | |
|---|---|---|---|---|---|---|---|---|---|
| ***Acquisition Time (sec)*** | 1.9818 | ***Comment*** | single_pulse | ***Date*** | 09 Feb 1990 03:52:47 | | | ***Date Stamp*** | 08 Aug 2018 13:22:59 |
| ***File Name*** | C:\USERS\FEDOR\DESKTOP\08.08.18\FZ6851-1.JDF | | | ***Frequency (MHz)*** | 600.17 | ***Nucleus*** | 1H | ***Number of Transients*** | 8 |
| ***Origin*** | ECA 600 | ***Original Points Count*** | 32768 | ***Owner*** | delta | ***Points Count*** | 32768 | ***Pulse Sequence*** | single_pulse.ex2 |
| ***Receiver Gain*** | 24.00 | ***Solvent*** | CHLOROFORM-d | | | ***Spectrum Offset (Hz)*** | 5385.9019 | ***Sweep Width (Hz)*** | 16534.39 |

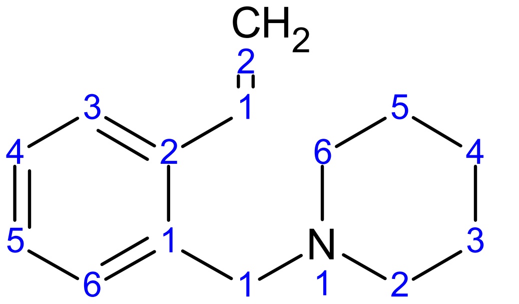

5b

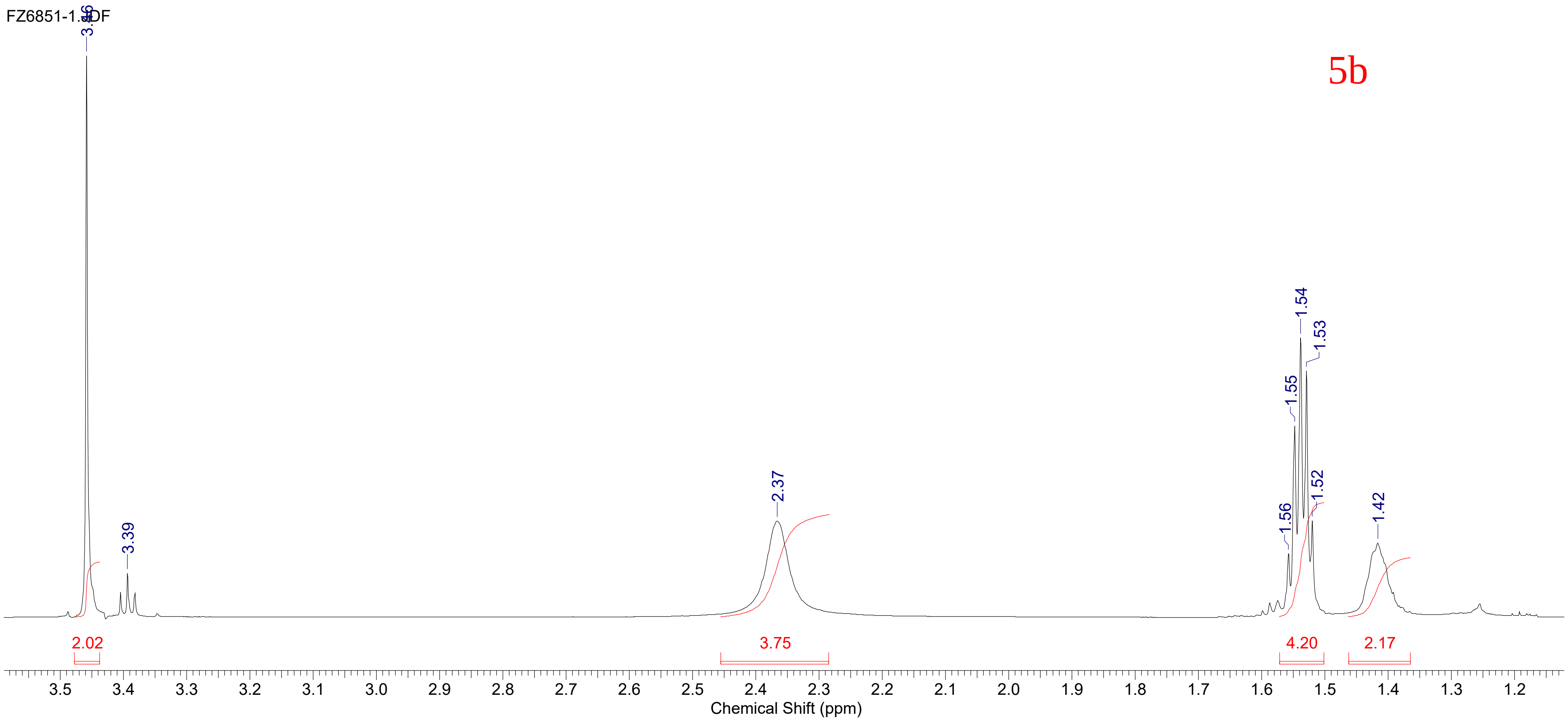

| | | | | | | | | | |
|---|---|---|---|---|---|---|---|---|---|
| ***Acquisition Time (sec)*** | 0.6921 | ***Comment*** | single pulse decoupled gated NOE | | | ***Date*** | 14 Feb 1990 01:52:31 | | |
| ***Date Stamp*** | 13 Aug 2018 11:22:48 | | | ***File Name*** | C:\Users\Fedor\Desktop\08.08.18\FZ6851-2.jdf | | | ***Frequency (MHz)*** | 150.91 |
| ***Nucleus*** | 13C | ***Number of Transients*** | 969 | ***Origin*** | ECA 600 | ***Original Points Count*** | 32768 | ***Owner*** | delta |
| ***Points Count*** | 32768 | ***Pulse Sequence*** | single_pulse_dec | | | ***Receiver Gain*** | 54.00 | ***Solvent*** | CHLOROFORM-d |
| ***Spectrum Offset (Hz)*** | 15072.1279 | ***Sweep Width (Hz)*** | 47348.49 | | | | | | |

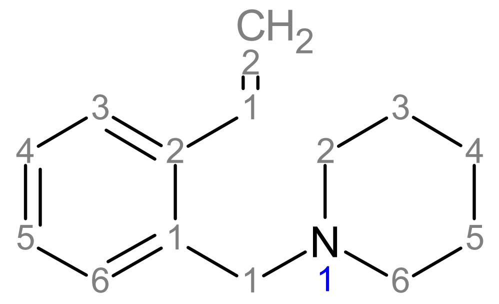


5b

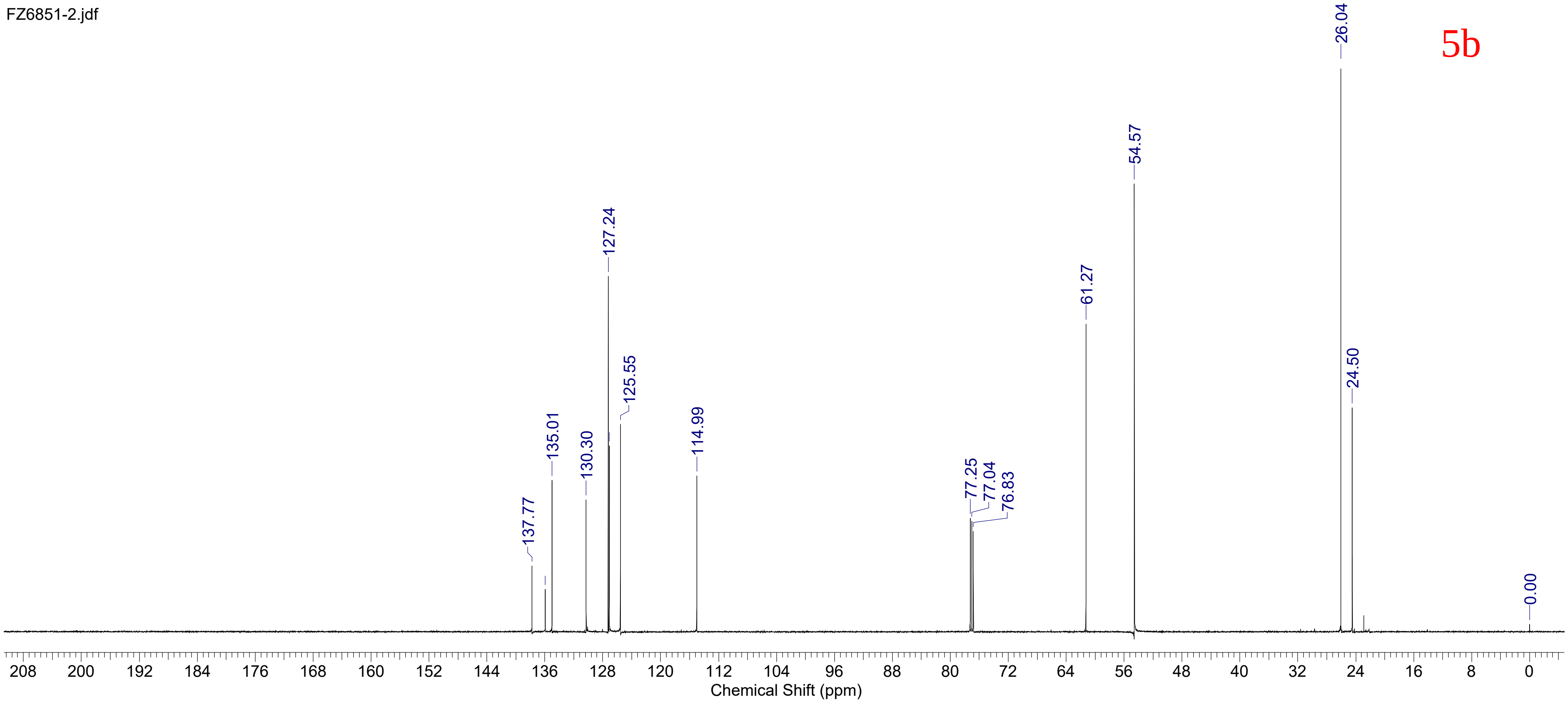

| | | | | | | | |
|---|---|---|---|---|---|---|---|
| ***Acquisition Time (sec)*** | 0.6921 | ***Comment*** | single pulse decoupled gated NOE | ***Date*** | 14 Feb 1990 01:52:31 | | |
| ***Date Stamp*** | 13 Aug 2018 11:22:48 | ***File Name*** | C:\Users\Fedor\Desktop\08.08.18\FZ6851-2.jdf | ***Frequency (MHz)*** | 150.91 | | |
| ***Nucleus*** | 13C | ***Number of Transients*** | 969 | ***Origin*** | ECA 600 | ***Original Points Count*** | 32768 |
| ***Owner*** | delta | ***Points Count*** | 32768 | ***Pulse Sequence*** | single_pulse_dec | ***Receiver Gain*** | 54.00 |
| ***Solvent*** | CHLOROFORM-d | ***Spectrum Offset (Hz)*** | 15072.1279 | ***Sweep Width (Hz)*** | 47348.49 | | |

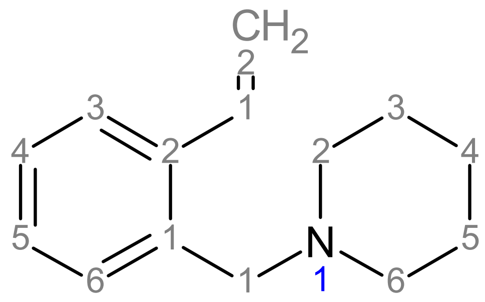

5b

FZ6851-2.jdf

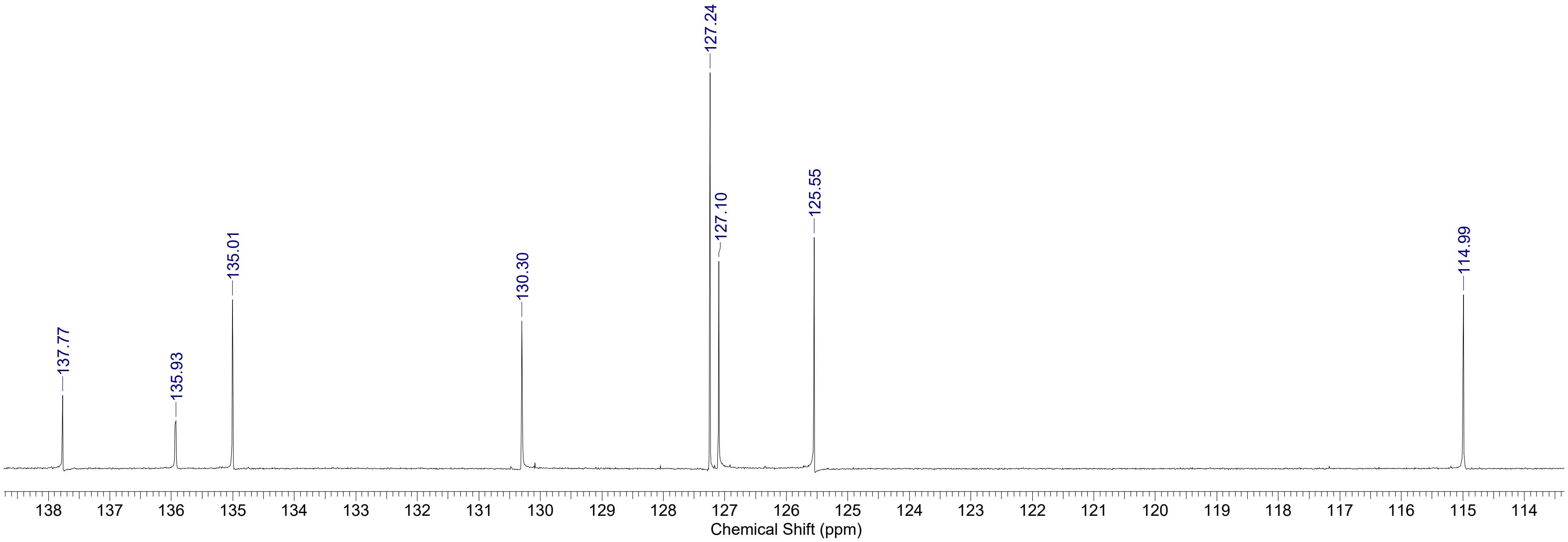

| | | | | | | | | | |
|---|---|---|---|---|---|---|---|---|---|
| ***Acquisition Time (sec)*** | 0.6921 | ***Comment*** | single pulse decoupled gated NOE | | | ***Date*** | 14 Feb 1990 01:52:31 | | |
| ***Date Stamp*** | 13 Aug 2018 11:22:48 | | | ***File Name*** | C:\Users\Fedor\Desktop\08.08.18\FZ6851-2.jdf | | | ***Frequency (MHz)*** | 150.91 |
| ***Nucleus*** | 13C | ***Number of Transients*** | 969 | ***Origin*** | ECA 600 | ***Original Points Count*** | 32768 | ***Owner*** | delta |
| ***Points Count*** | 32768 | ***Pulse Sequence*** | single_pulse_dec | | | ***Receiver Gain*** | 54.00 | ***Solvent*** | CHLOROFORM-d |
| ***Spectrum Offset (Hz)*** | 15072.1279 | ***Sweep Width (Hz)*** | 47348.49 | | | | | | |

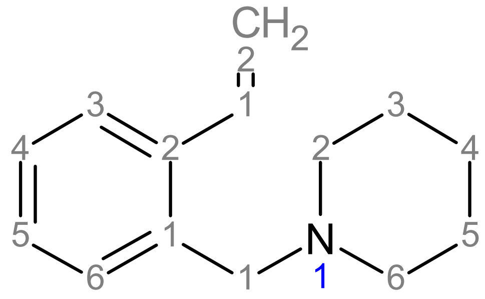

5b

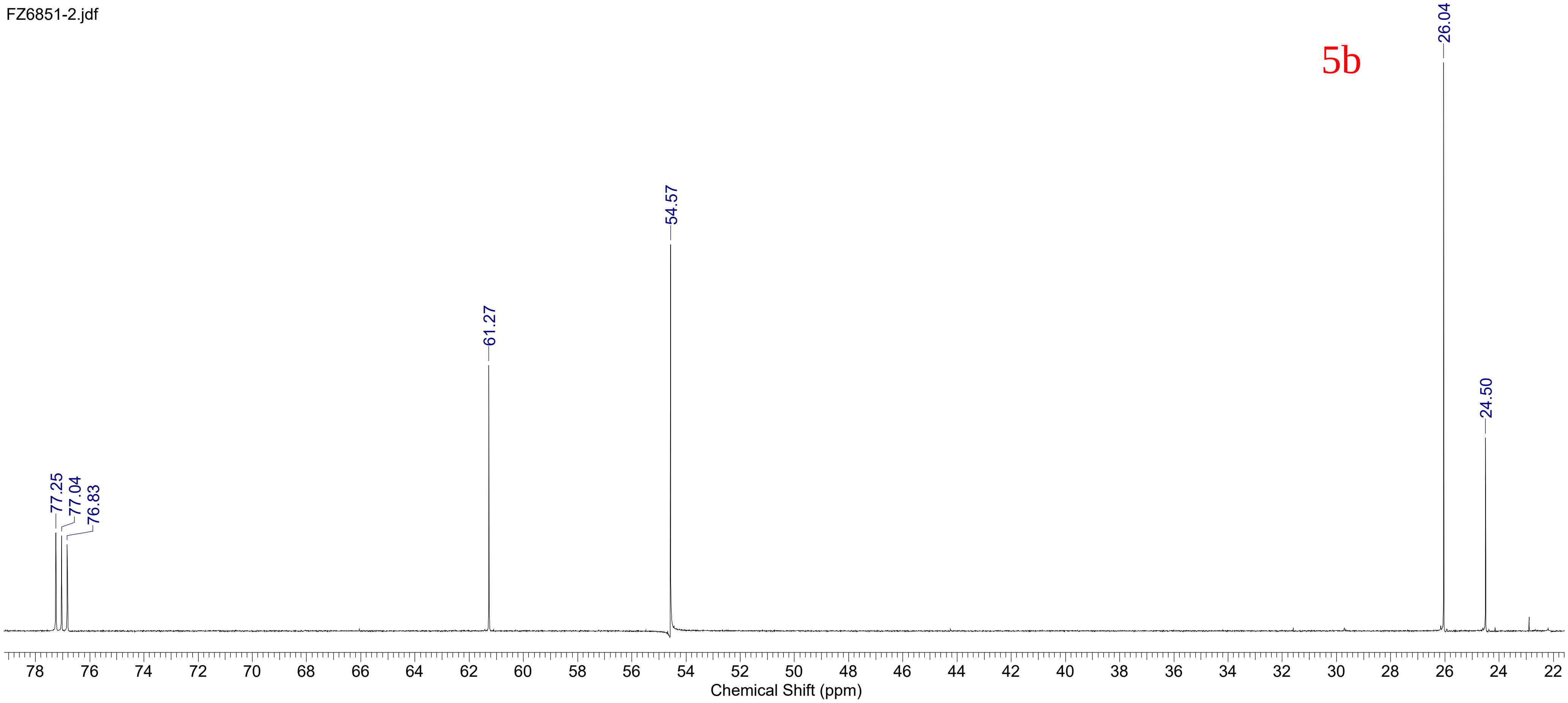

| | | | | | | | | | |
|---|---|---|---|---|---|---|---|---|---|
| ***Acquisition Time (sec)*** | 1.9818 | ***Comment*** | single_pulse | ***Date*** | 29 Mar 1990 02:03:03 | | | ***Date Stamp*** | 26 Sep 2018 11:32:31 |
| ***File Name*** | C:\Users\Fedor\Desktop\26.09.18\FZ6888-1.jdf | | | ***Frequency (MHz)*** | 600.17 | ***Nucleus*** | 1H | ***Number of Transients*** | 8 |
| ***Origin*** | ECA 600 | ***Original Points Count*** | 32768 | ***Owner*** | delta | ***Points Count*** | 32768 | ***Pulse Sequence*** | single_pulse.ex2 |
| ***Receiver Gain*** | 36.00 | ***Solvent*** | CHLOROFORM-d | | | ***Spectrum Offset (Hz)*** | 5401.5503 | ***Sweep Width (Hz)*** | 16534.39 |

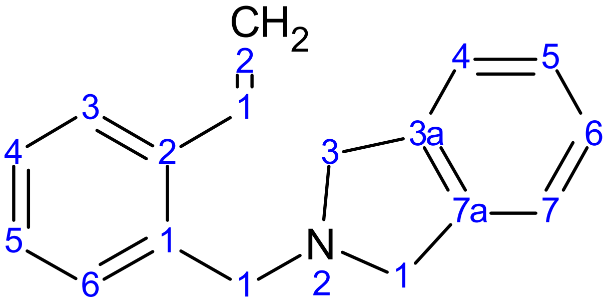


5c

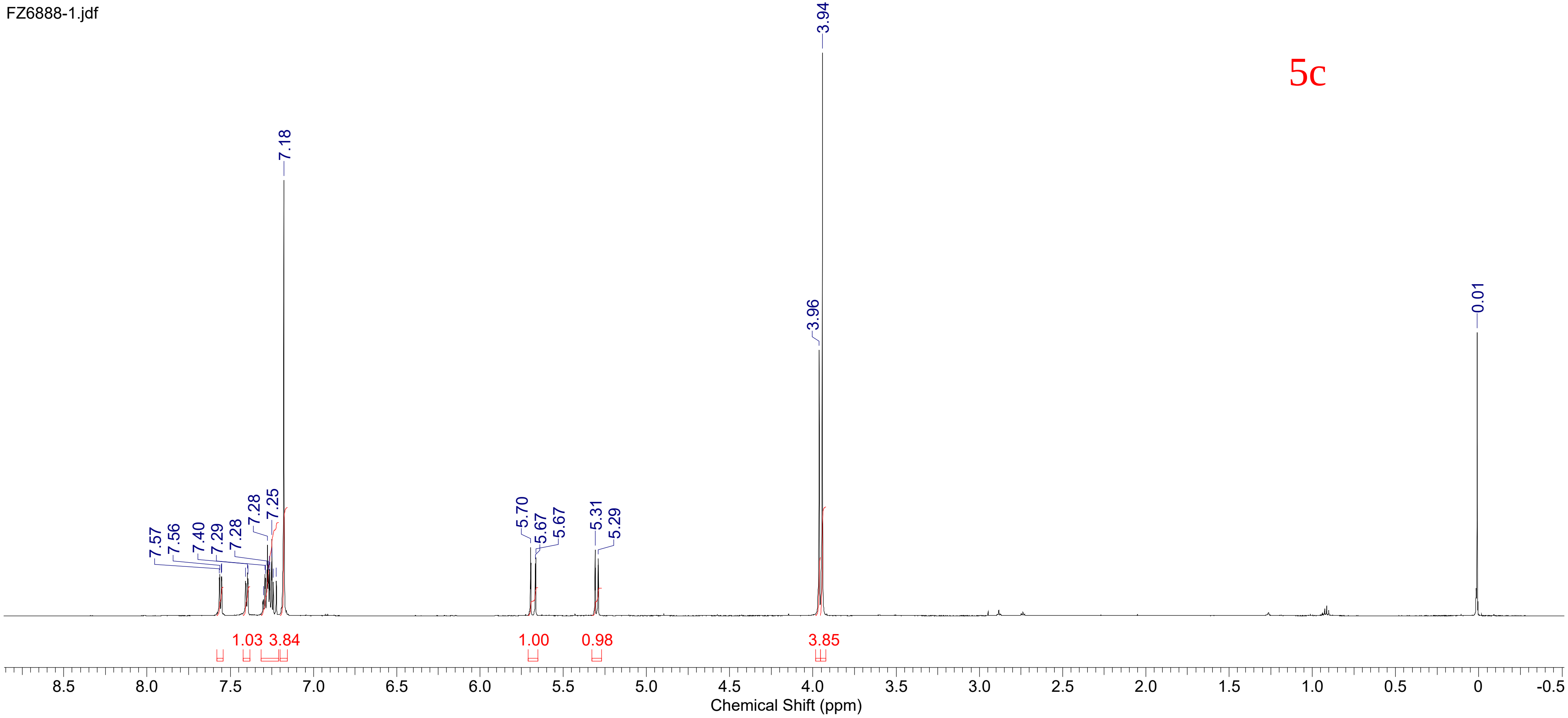

| Acquisition Time (sec) | 1.9818 | Comment | single_pulse | Date | 29 Mar 1990 02:03:03 | | | Date Stamp | 26 Sep 2018 11:32:31 |
|---|---|---|---|---|---|---|---|---|---|
| File Name | C:\Users\Fedor\Desktop\26.09.18\FZ6888-1.jdf | | | Frequency (MHz) | 600.17 | Nucleus | 1H | Number of Transients | 8 |
| Origin | ECA 600 | Original Points Count | 32768 | Owner | delta | Points Count | 32768 | Pulse Sequence | single_pulse.ex2 |
| Receiver Gain | 36.00 | Solvent | CHLOROFORM-d | | | Spectrum Offset (Hz) | 5401.5503 | Sweep Width (Hz) | 16534.39 |

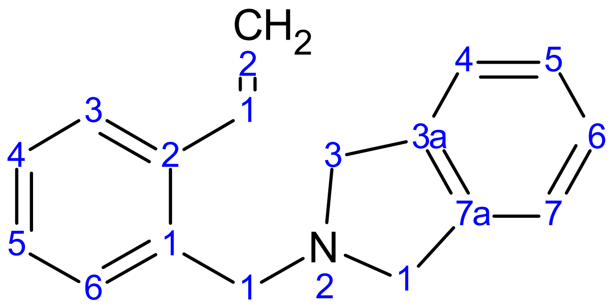


FZ6888-1.jdf

5c

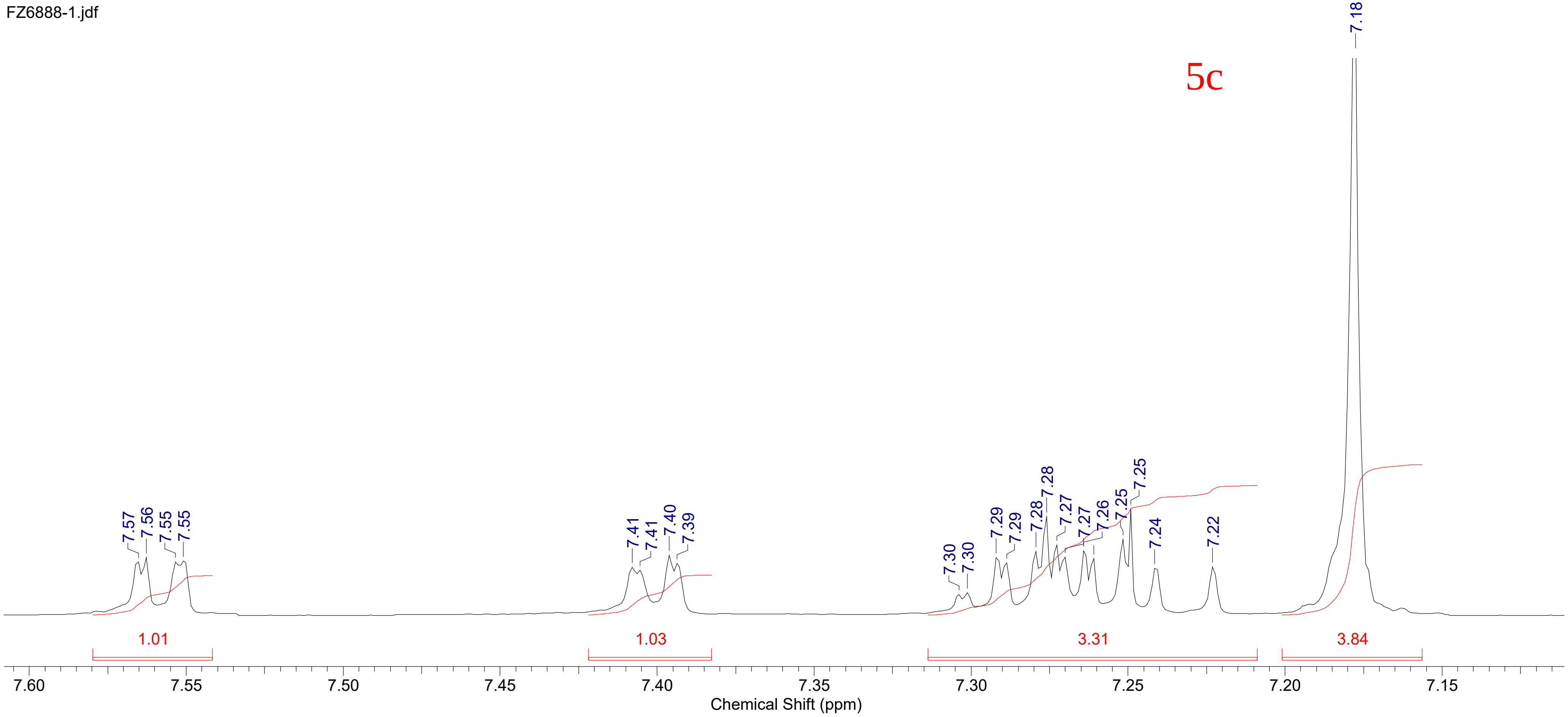

| Acquisition Time (sec) | 1.9818 | Comment | single_pulse | Date | 29 Mar 1990 02:03:03 | | | Date Stamp | 26 Sep 2018 11:32:31 |
|---|---|---|---|---|---|---|---|---|---|
| File Name | C:\Users\Fedor\Desktop\26.09.18\FZ6888-1.jdf | | | Frequency (MHz) | 600.17 | Nucleus | 1H | Number of Transients | 8 |
| Origin | ECA 600 | Original Points Count | 32768 | Owner | delta | Points Count | 32768 | Pulse Sequence | single_pulse.ex2 |
| Receiver Gain | 36.00 | Solvent | CHLOROFORM-d | | | Spectrum Offset (Hz) | 5401.5503 | Sweep Width (Hz) | 16534.39 |

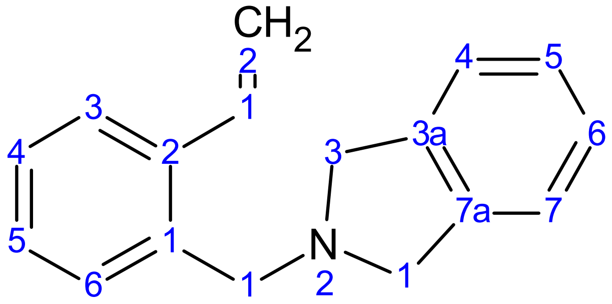


5c

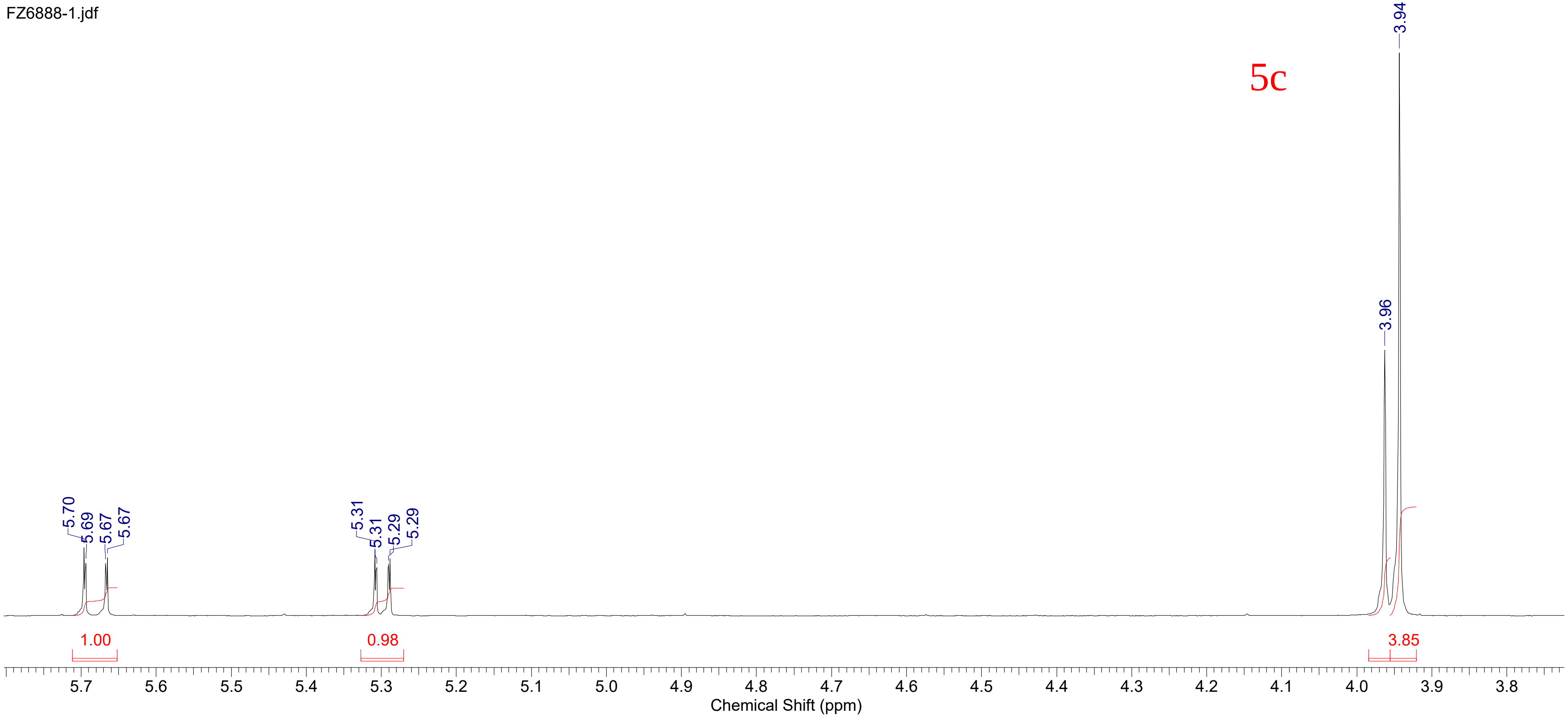

| | | | | | | | |
|---|---|---|---|---|---|---|---|
| ***Acquisition Time (sec)*** | 0.6921 | ***Comment*** | single pulse decoupled gated NOE | ***Date*** | 30 Mar 1990 17:27:25 | | |
| ***Date Stamp*** | 28 Sep 2018 07:59:09 | ***File Name*** | C:\Users\Fedor\Desktop\26.09.18\FZ6888-2.jdf | ***Frequency (MHz)*** | 150.91 | | |
| ***Nucleus*** | 13C | ***Number of Transients*** | 1000 | ***Origin*** | ECA 600 | ***Original Points Count*** | 32768 |
| ***Owner*** | delta | ***Points Count*** | 32768 | ***Pulse Sequence*** | single_pulse_dec | ***Receiver Gain*** | 52.00 |
| ***Solvent*** | CHLOROFORM-d | ***Spectrum Offset (Hz)*** | 15073.5723 | ***Sweep Width (Hz)*** | 47348.49 | | |

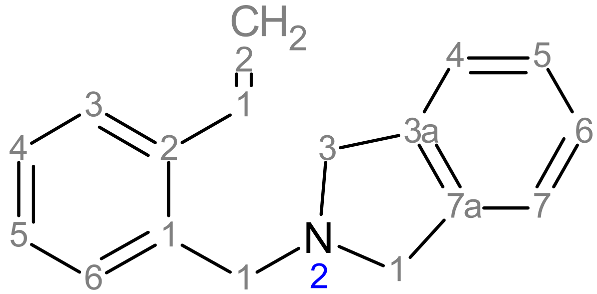


FZ6888-2.jdf

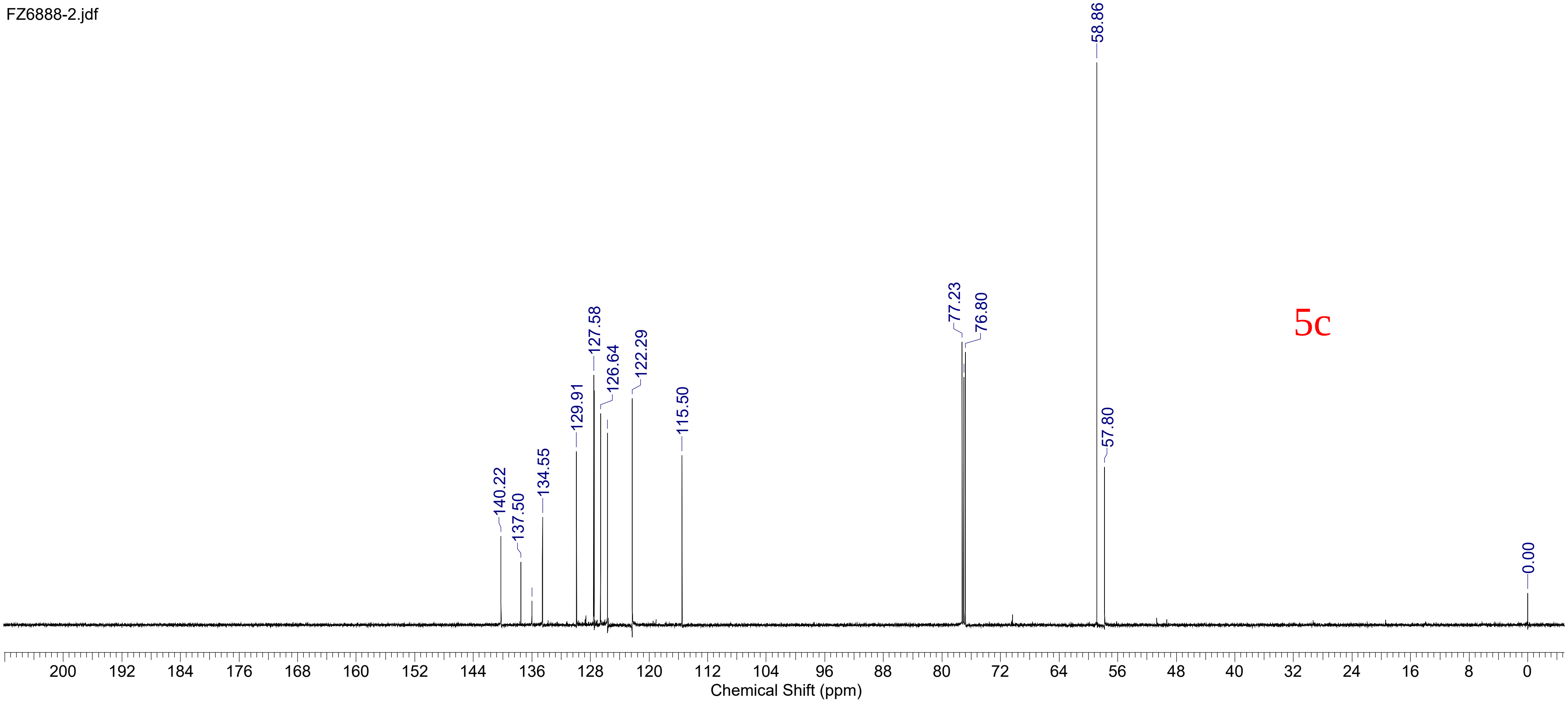


5c

| | | | | | | | | | |
|---|---|---|---|---|---|---|---|---|---|
| ***Acquisition Time (sec)*** | 0.6921 | ***Comment*** | single pulse decoupled gated NOE | | | ***Date*** | 30 Mar 1990 17:27:25 | | |
| ***Date Stamp*** | 28 Sep 2018 07:59:09 | | | ***File Name*** | C:\Users\Fedor\Desktop\26.09.18\FZ6888-2.jdf | | | ***Frequency (MHz)*** | 150.91 |
| ***Nucleus*** | 13C | ***Number of Transients*** | 1000 | ***Origin*** | ECA 600 | ***Original Points Count*** | 32768 | ***Owner*** | delta |
| ***Points Count*** | 32768 | ***Pulse Sequence*** | single_pulse_dec | | | ***Receiver Gain*** | 52.00 | ***Solvent*** | CHLOROFORM-d |
| ***Spectrum Offset (Hz)*** | 15073.5723 | ***Sweep Width (Hz)*** | 47348.49 | | | | | | |

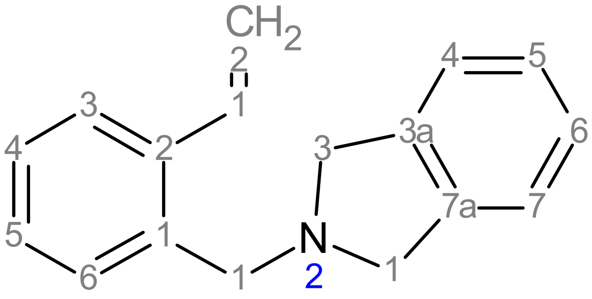


FZ6888-2.jdf

5c

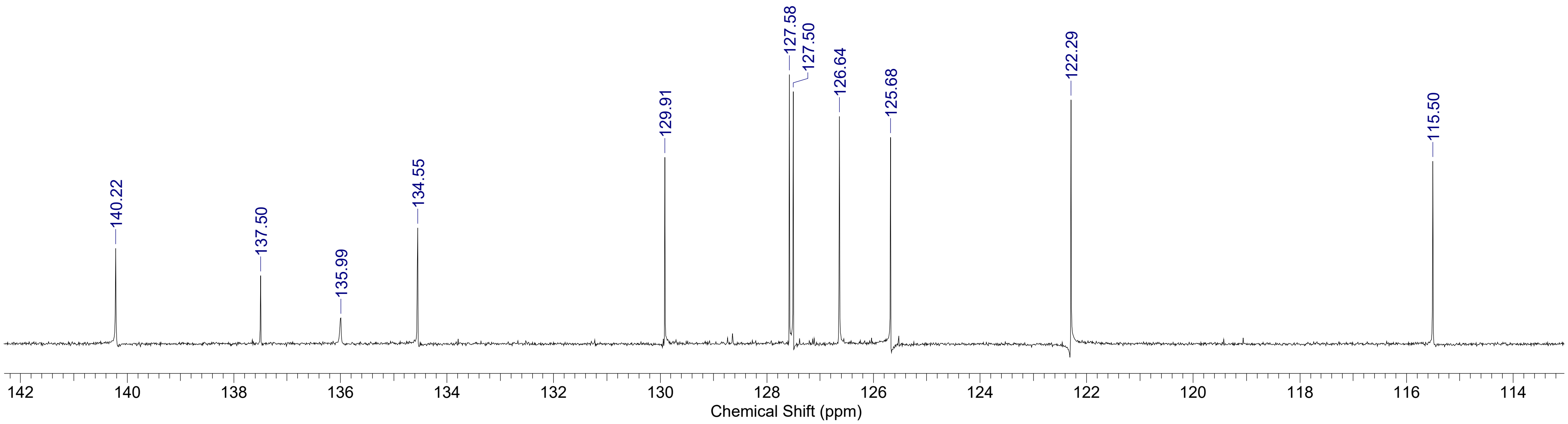

| | | | | | | | |
|---|---|---|---|---|---|---|---|
| ***Acquisition Time (sec)*** | 0.6921 | ***Comment*** | single pulse decoupled gated NOE | ***Date*** | 30 Mar 1990 17:27:25 | | |
| ***Date Stamp*** | 28 Sep 2018 07:59:09 | ***File Name*** | C:\Users\Fedor\Desktop\26.09.18\FZ6888-2.jdf | ***Frequency (MHz)*** | 150.91 | | |
| ***Nucleus*** | 13C | ***Number of Transients*** | 1000 | ***Origin*** | ECA 600 | ***Original Points Count*** | 32768 |
| ***Owner*** | delta | ***Points Count*** | 32768 | ***Pulse Sequence*** | single_pulse_dec | ***Receiver Gain*** | 52.00 |
| ***Solvent*** | CHLOROFORM-d | ***Spectrum Offset (Hz)*** | 15073.5723 | ***Sweep Width (Hz)*** | 47348.49 | | |

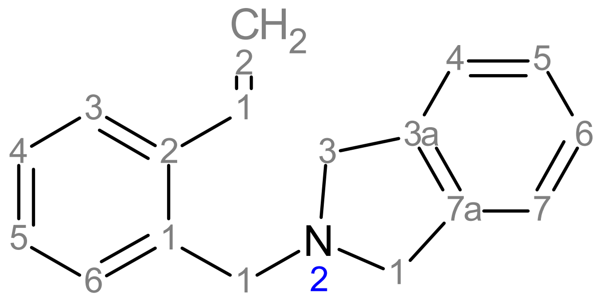


5c

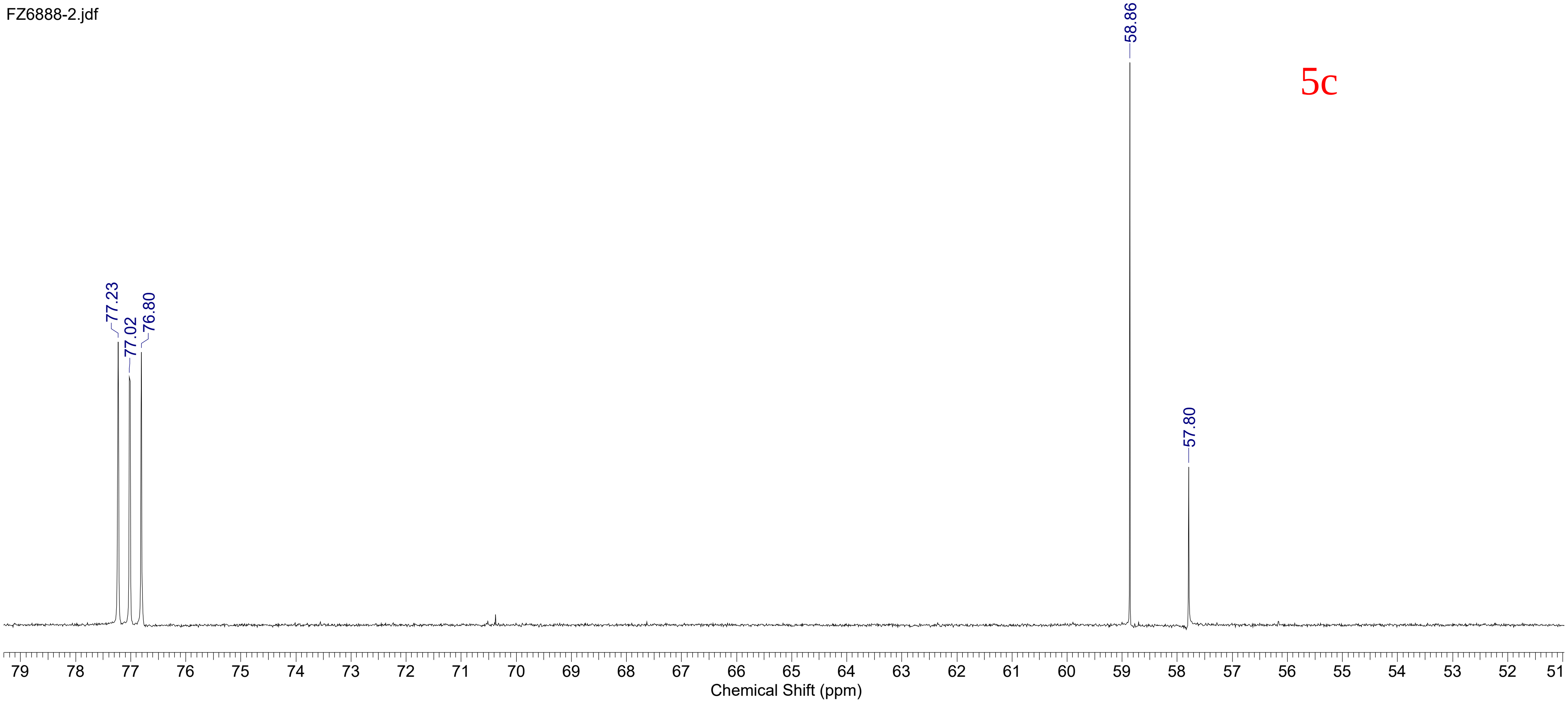

| | | | | | | | | | |
|---|---|---|---|---|---|---|---|---|---|
| ***Acquisition Time (sec)*** | 1.9818 | ***Comment*** | single_pulse | ***Date*** | 19 Feb 1990 07:27:10 | | | | |
| ***Date Stamp*** | 14 Feb 2018 12:15:19 | | | ***File Name*** | C:\Users\Fedor\Desktop\13.02.18\FZ6413-1.jdf | | | ***Frequency (MHz)*** | 600.17 |
| ***Nucleus*** | 1H | ***Number of Transients*** | 8 | ***Origin*** | ECA 600 | ***Original Points Count*** | 32768 | ***Owner*** | delta |
| ***Points Count*** | 32768 | ***Pulse Sequence*** | single_pulse.ex2 | | | ***Receiver Gain*** | 36.00 | ***Solvent*** | CHLOROFORM-d |
| ***Spectrum Offset (Hz)*** | 5391.4521 | ***Sweep Width (Hz)*** | 16534.39 | ***Temperature (degree C)*** | 25.000 | | | | |

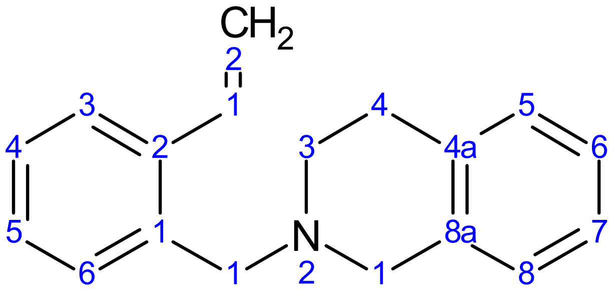


FZ6413-1.jdf

5d

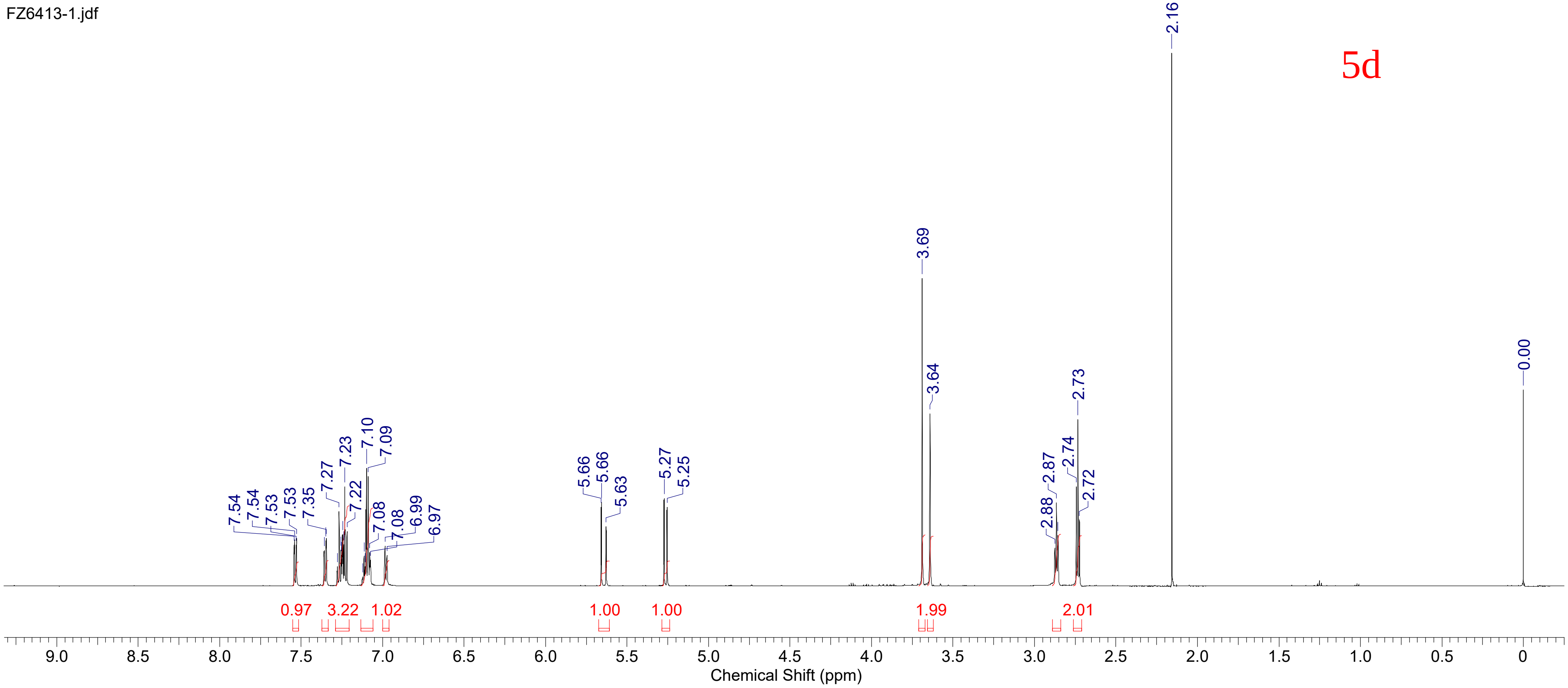

| | | | | | | | |
|---|---|---|---|---|---|---|---|
| ***Acquisition Time (sec)*** | 1.9818 | ***Comment*** | single_pulse | ***Date*** | 19 Feb 1990 07:27:10 | | |
| ***Date Stamp*** | 14 Feb 2018 12:15:19 | | | ***File Name*** | C:\Users\Fedor\Desktop\13.02.18\FZ6413-1.jdf | ***Frequency (MHz)*** | 600.17 |
| ***Nucleus*** | 1H | ***Number of Transients*** | 8 | ***Origin*** | ECA 600 | ***Original Points Count*** 32768 | ***Owner*** delta |
| ***Points Count*** | 32768 | ***Pulse Sequence*** | single_pulse.ex2 | | | ***Receiver Gain*** 36.00 | ***Solvent*** CHLOROFORM-d |
| ***Spectrum Offset (Hz)*** | 5391.4521 | ***Sweep Width (Hz)*** | 16534.39 | ***Temperature (degree C)*** | 25.000 | | |

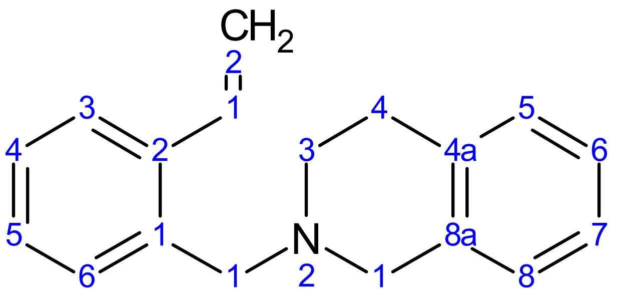


FZ6413-1.jdf

5d

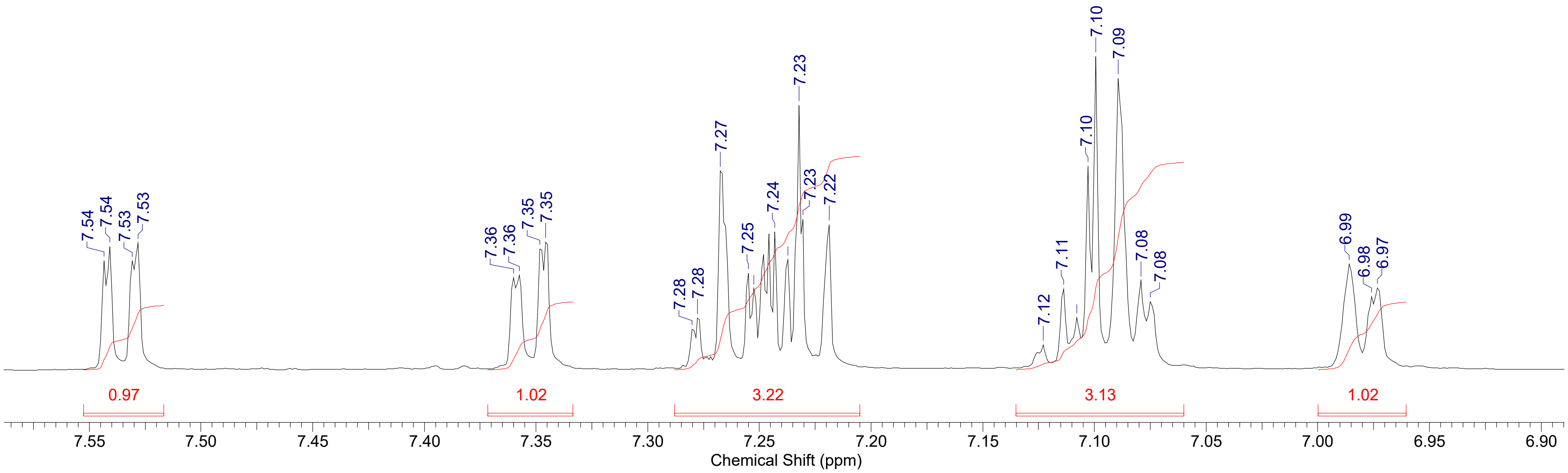

| | | | | | | | |
|---|---|---|---|---|---|---|---|
| ***Acquisition Time (sec)*** | 1.9818 | ***Comment*** | single_pulse | ***Date*** | 19 Feb 1990 07:27:10 | | |
| ***Date Stamp*** | 14 Feb 2018 12:15:19 | | | ***File Name*** | C:\Users\Fedor\Desktop\13.02.18\FZ6413-1.jdf | ***Frequency (MHz)*** | 600.17 |
| ***Nucleus*** | 1H | ***Number of Transients*** | 8 | ***Origin*** | ECA 600 | ***Original Points Count*** | 32768 |
| | | | | | | ***Owner*** | delta |
| ***Points Count*** | 32768 | ***Pulse Sequence*** | single_pulse.ex2 | | | ***Receiver Gain*** | 36.00 |
| | | | | | | ***Solvent*** | CHLOROFORM-d |
| ***Spectrum Offset (Hz)*** | 5391.4521 | ***Sweep Width (Hz)*** | 16534.39 | ***Temperature (degree C)*** | 25.000 | | |

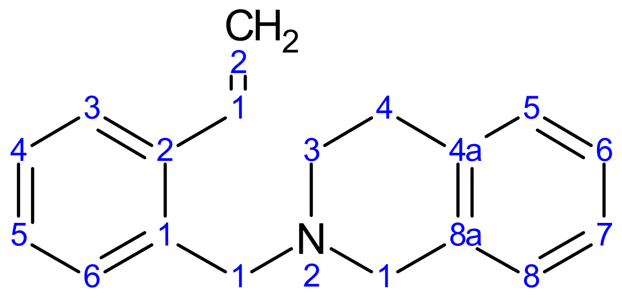


5d

FZ6413-1.jdf

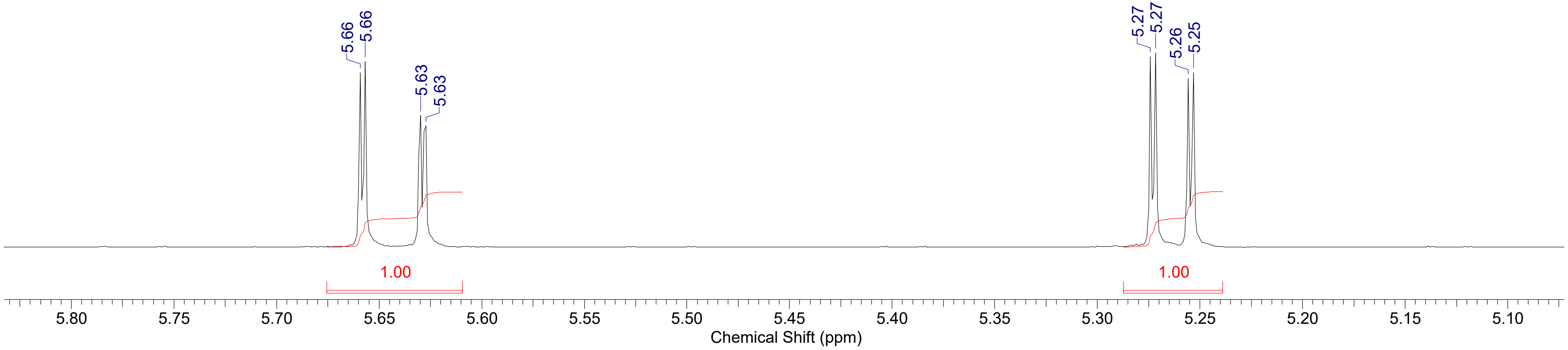

| Acquisition Time (sec) | 1.9818 | Comment | single_pulse | Date | 19 Feb 1990 07:27:10 | | |
|---|---|---|---|---|---|---|---|
| Date Stamp | 14 Feb 2018 12:15:19 | | | File Name | C:\Users\Fedor\Desktop\13.02.18\FZ6413-1.jdf | Frequency (MHz) | 600.17 |
| Nucleus | 1H | Number of Transients | 8 | Origin | ECA 600 | Original Points Count | 32768 | Owner | delta |
| Points Count | 32768 | Pulse Sequence | single_pulse.ex2 | | | Receiver Gain | 36.00 | Solvent | CHLOROFORM-d |
| Spectrum Offset (Hz) | 5391.4521 | Sweep Width (Hz) | 16534.39 | Temperature (degree C) | 25.000 | | |

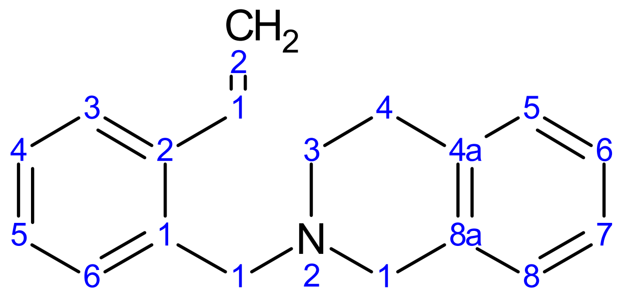

5d

FZ6413-1.jdf

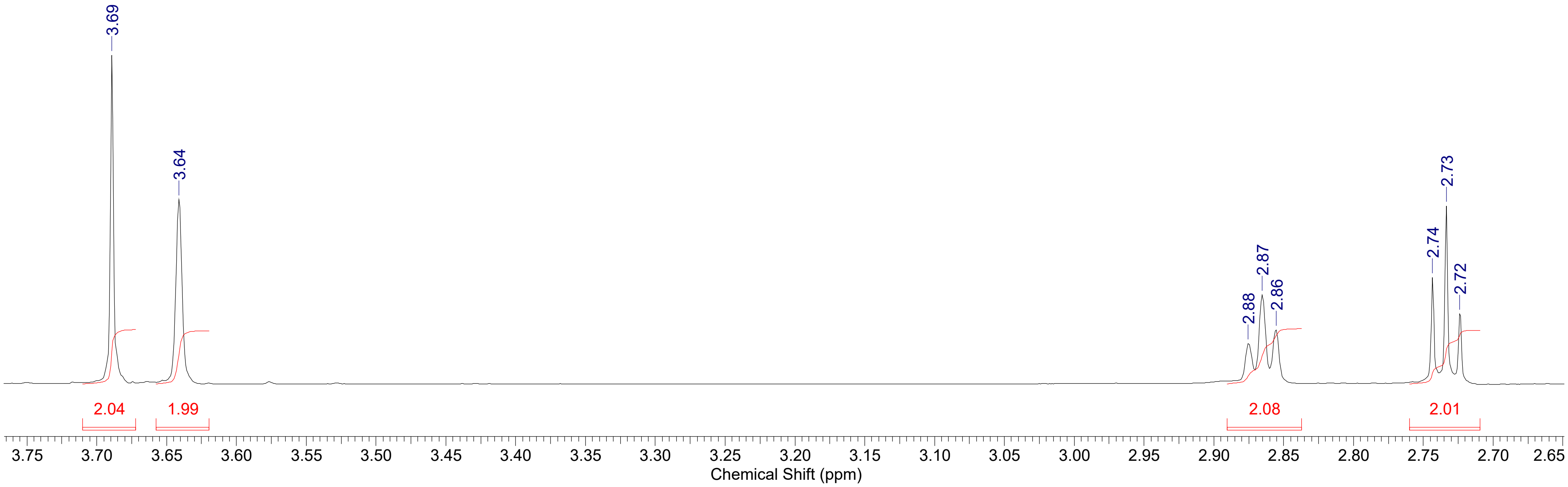

| | | | | | | | |
|---|---|---|---|---|---|---|---|
| ***Acquisition Time (sec)*** | 0.6921 | ***Comment*** | single pulse decoupled gated NOE | | | ***Date*** | 10 Mar 1990 09:44:54 |
| ***Date Stamp*** | 06 Mar 2018 14:32:33 | | | ***File Name*** | C:\Users\Fedor\Desktop\06.03.18\FZ6488-1.jdf | | |
| ***Frequency (MHz)*** | 150.91 | ***Nucleus*** | 13C | ***Number of Transients*** | 161 | ***Origin*** | ECA 600 |
| ***Original Points Count*** | 32768 | ***Owner*** | delta | ***Points Count*** | 32768 | ***Pulse Sequence*** | single_pulse_dec |
| ***Receiver Gain*** | 52.00 | ***Solvent*** | CHLOROFORM-d | | | ***Spectrum Offset (Hz)*** | 15091.3428 |
| ***Sweep Width (Hz)*** | 47348.49 | ***Temperature (degree C)*** | 23.200 | | | | |

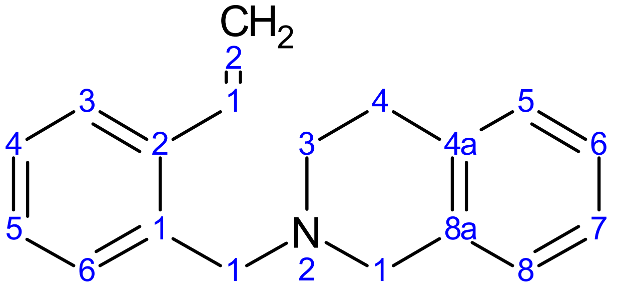


5d

FZ6488-1.jdf

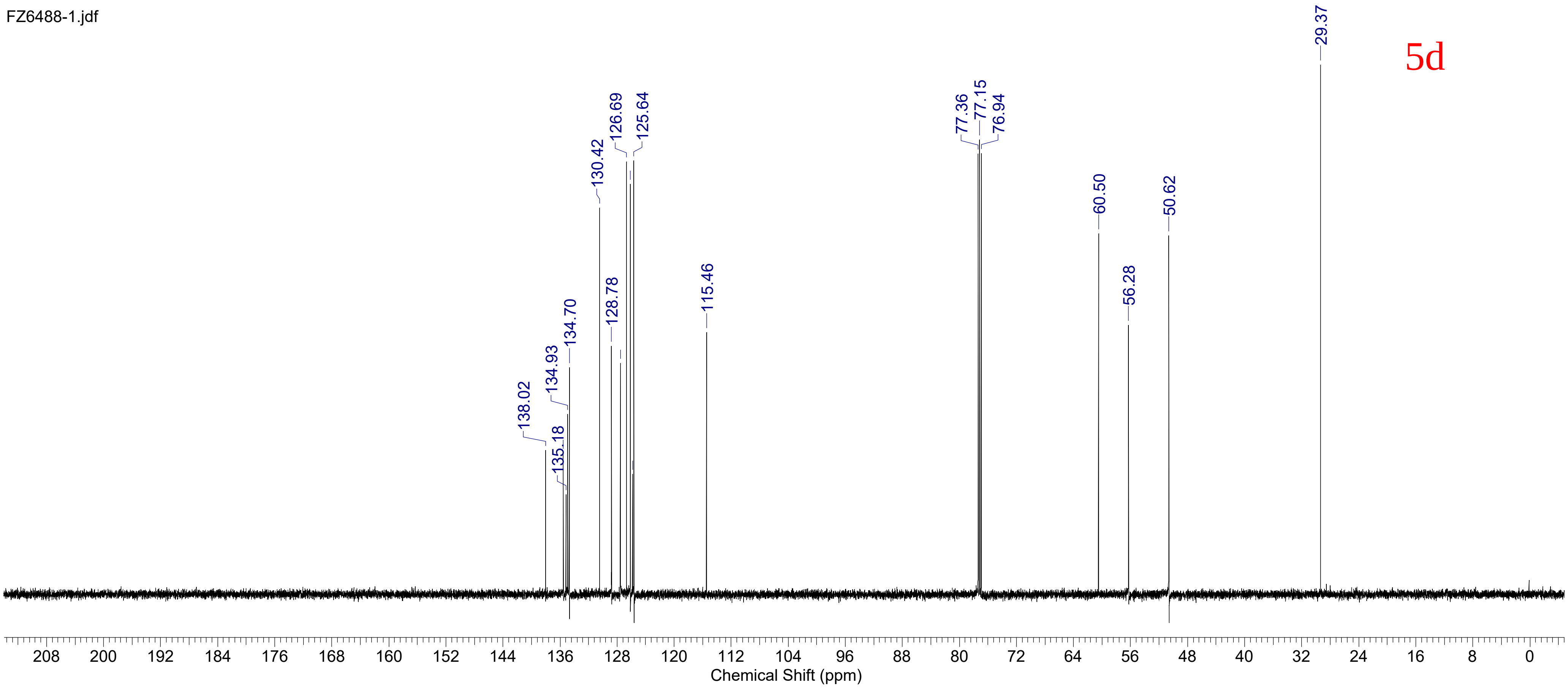

| | | | | | | | |
|---|---|---|---|---|---|---|---|
| ***Acquisition Time (sec)*** | 0.6921 | ***Comment*** | single pulse decoupled gated NOE | | | ***Date*** | 10 Mar 1990 09:44:54 |
| ***Date Stamp*** | 06 Mar 2018 14:32:33 | | | ***File Name*** | C:\Users\Fedor\Desktop\06.03.18\FZ6488-1.jdf | | |
| ***Frequency (MHz)*** | 150.91 | ***Nucleus*** | 13C | ***Number of Transients*** | 161 | ***Origin*** | ECA 600 |
| ***Original Points Count*** | 32768 | ***Owner*** | delta | ***Points Count*** | 32768 | ***Pulse Sequence*** | single_pulse_dec |
| ***Receiver Gain*** | 52.00 | ***Solvent*** | CHLOROFORM-d | | | ***Spectrum Offset (Hz)*** | 15091.3428 |
| ***Sweep Width (Hz)*** | 47348.49 | ***Temperature (degree C)*** | 23.200 | | | | |

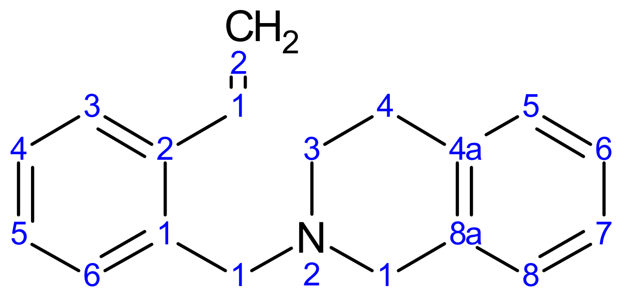

5d

FZ6488-1.jdf

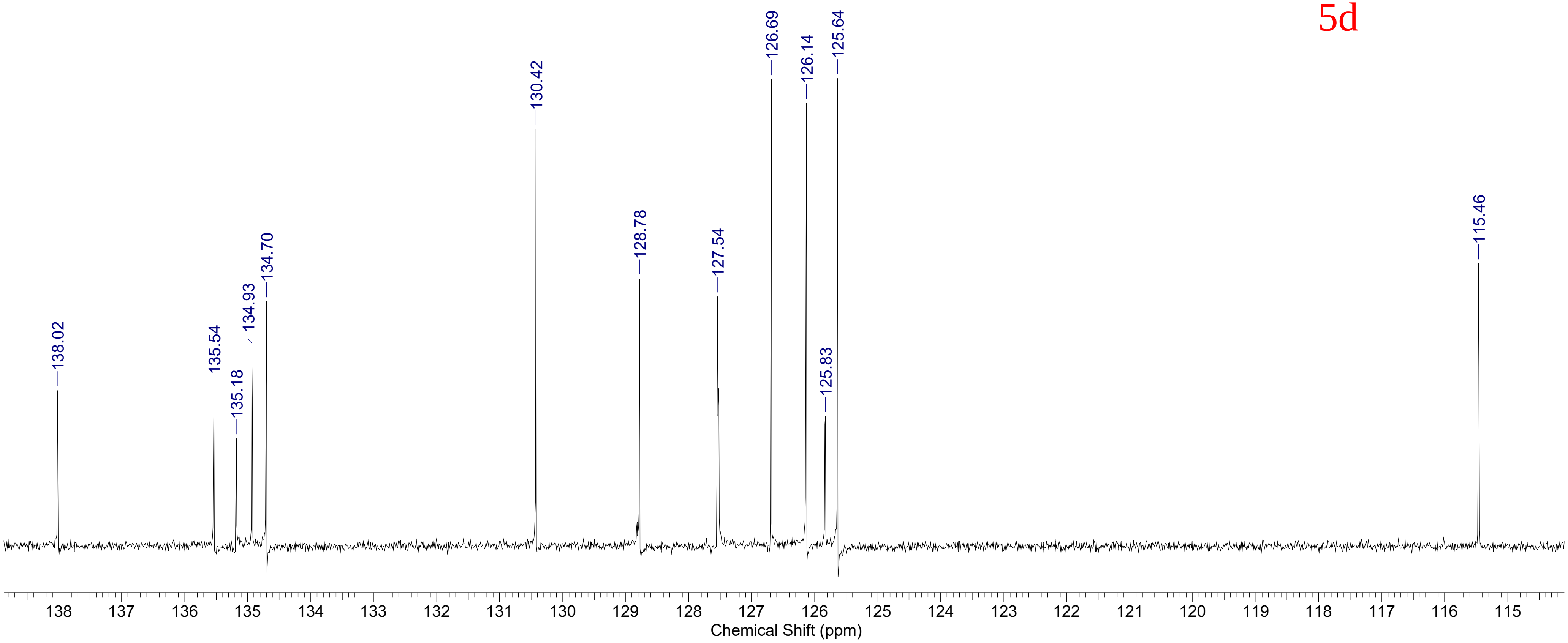

| | | | | | | | |
|---|---|---|---|---|---|---|---|
| ***Acquisition Time (sec)*** | 0.6921 | ***Comment*** | single pulse decoupled gated NOE | | | ***Date*** | 10 Mar 1990 09:44:54 |
| ***Date Stamp*** | 06 Mar 2018 14:32:33 | | | ***File Name*** | C:\Users\Fedor\Desktop\06.03.18\FZ6488-1.jdf | | |
| ***Frequency (MHz)*** | 150.91 | ***Nucleus*** | 13C | ***Number of Transients*** | 161 | ***Origin*** | ECA 600 |
| ***Original Points Count*** | 32768 | ***Owner*** | delta | ***Points Count*** | 32768 | ***Pulse Sequence*** | single_pulse_dec |
| ***Receiver Gain*** | 52.00 | ***Solvent*** | CHLOROFORM-d | | | ***Spectrum Offset (Hz)*** | 15091.3428 |
| ***Sweep Width (Hz)*** | 47348.49 | ***Temperature (degree C)*** | 23.200 | | | | |

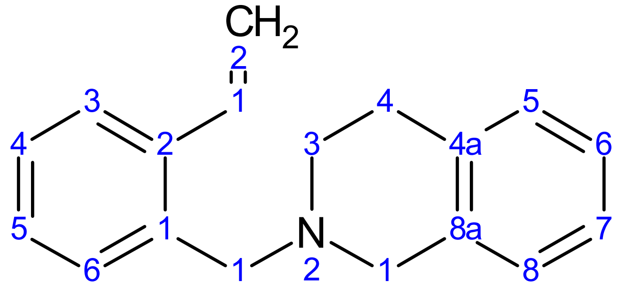


5d

FZ6488-1.jdf

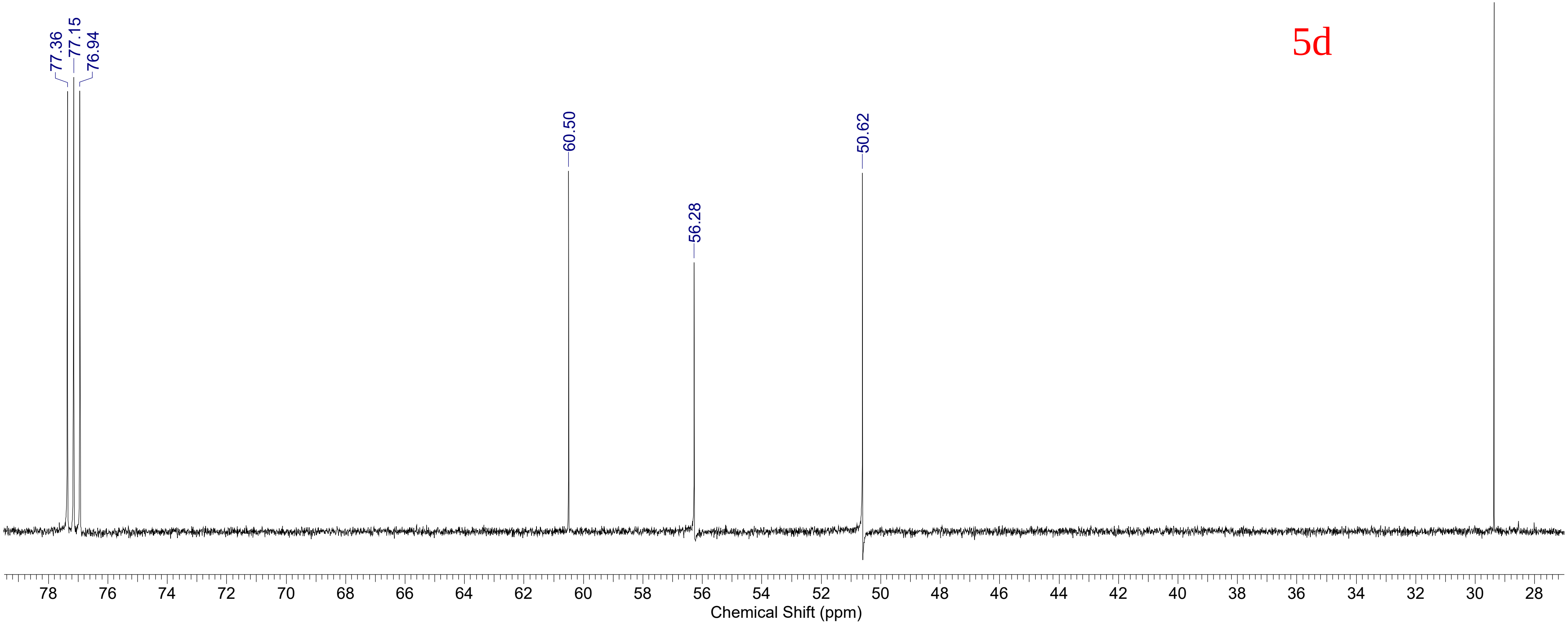

| | | | | | | | |
|---|---|---|---|---|---|---|---|
| ***Acquisition Time (sec)*** | 1.9818 | ***Comment*** | single_pulse | ***Date*** | 18 Mar 1990 08:34:49 | | |
| ***Date Stamp*** | 14 Mar 2018 13:22:36 | | | ***File Name*** | C:\Users\Fedor\Desktop\13.03.18\FZ6493-1.jdf | ***Frequency (MHz)*** | 600.17 |
| ***Nucleus*** | 1H | ***Number of Transients*** | 8 | ***Origin*** | ECA 600 | ***Original Points Count*** 32768 | ***Owner*** delta |
| ***Points Count*** | 32768 | ***Pulse Sequence*** | single_pulse.ex2 | | | ***Receiver Gain*** 28.00 | ***Solvent*** CHLOROFORM-d |
| ***Spectrum Offset (Hz)*** | 5394.4800 | ***Sweep Width (Hz)*** | 16534.39 | ***Temperature (degree C)*** | 22.200 | | |

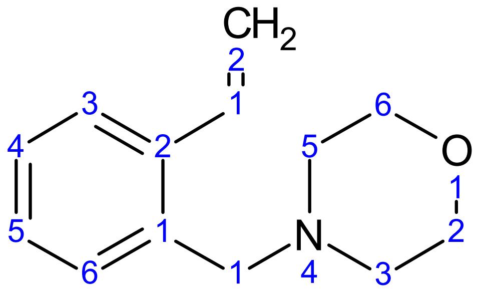


5e

FZ6493-1.jdf

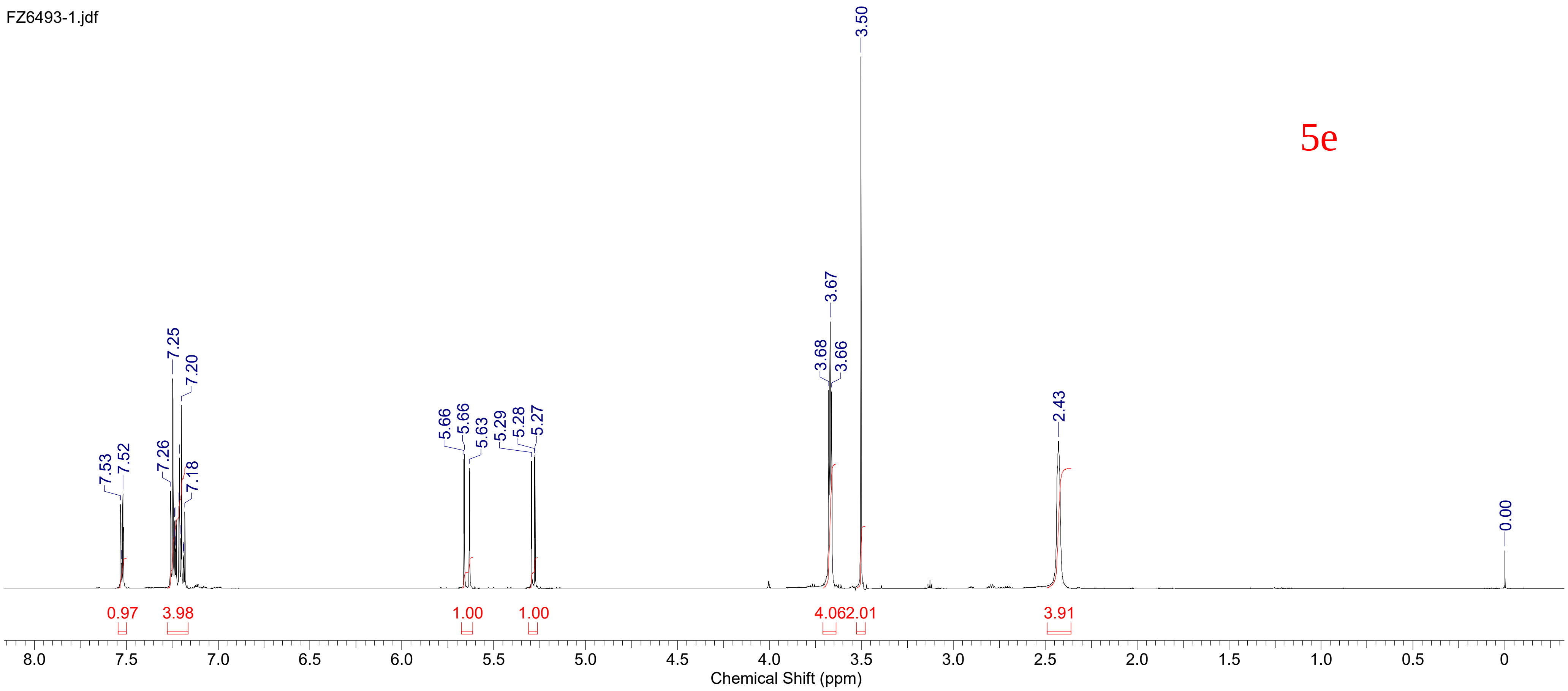

| | | | | | | | |
|---|---|---|---|---|---|---|---|
| ***Acquisition Time (sec)*** | 1.9818 | ***Comment*** | single_pulse | ***Date*** | 18 Mar 1990 08:34:49 | | |
| ***Date Stamp*** | 14 Mar 2018 13:22:36 | | | ***File Name*** | C:\Users\Fedor\Desktop\13.03.18\FZ6493-1.jdf | ***Frequency (MHz)*** | 600.17 |
| ***Nucleus*** | 1H | ***Number of Transients*** | 8 | ***Origin*** | ECA 600 | ***Original Points Count*** | 32768 |
| ***Owner*** | delta | | | | | | |
| ***Points Count*** | 32768 | ***Pulse Sequence*** | single_pulse.ex2 | | | ***Receiver Gain*** | 28.00 |
| ***Solvent*** | CHLOROFORM-d | | | | | | |
| ***Spectrum Offset (Hz)*** | 5394.4800 | ***Sweep Width (Hz)*** | 16534.39 | ***Temperature (degree C)*** | 22.200 | | |

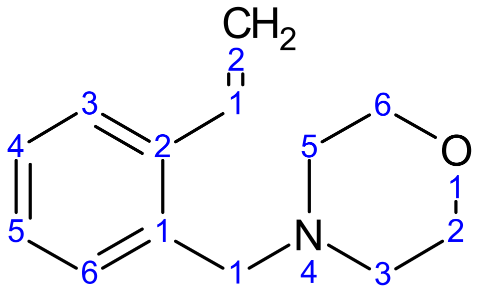


5e

FZ6493-1.jdf

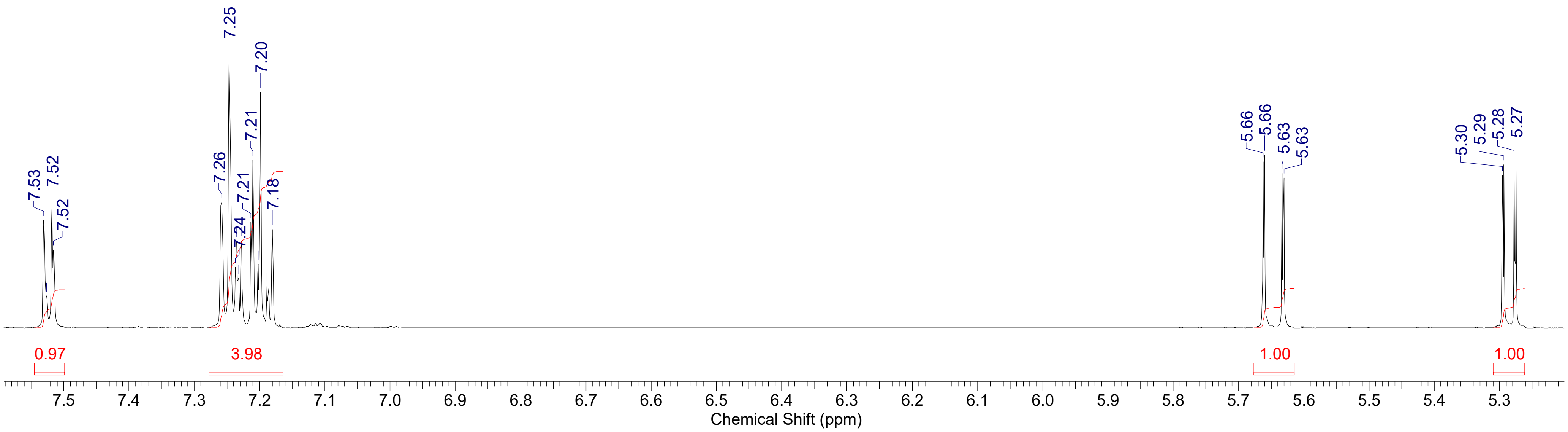

| | | | | | | | |
|---|---|---|---|---|---|---|---|
| ***Acquisition Time (sec)*** | 1.9818 | ***Comment*** | single_pulse | ***Date*** | 18 Mar 1990 08:34:49 | | |
| ***Date Stamp*** | 14 Mar 2018 13:22:36 | | | ***File Name*** | C:\Users\Fedor\Desktop\13.03.18\FZ6493-1.jdf | ***Frequency (MHz)*** | 600.17 |
| ***Nucleus*** | 1H | ***Number of Transients*** | 8 | ***Origin*** | ECA 600 | ***Original Points Count*** | 32768 |
| ***Owner*** | delta | | | | | | |
| ***Points Count*** | 32768 | ***Pulse Sequence*** | single_pulse.ex2 | ***Receiver Gain*** | 28.00 | ***Solvent*** | CHLOROFORM-d |
| ***Spectrum Offset (Hz)*** | 5394.4800 | ***Sweep Width (Hz)*** | 16534.39 | ***Temperature (degree C)*** | 22.200 | | |

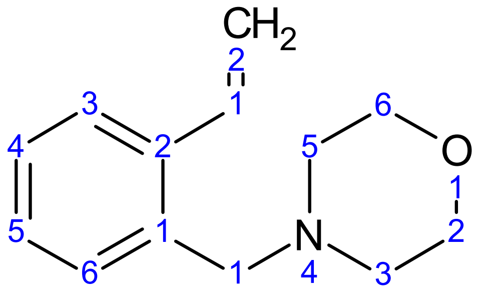


5e

FZ6493-1.jdf

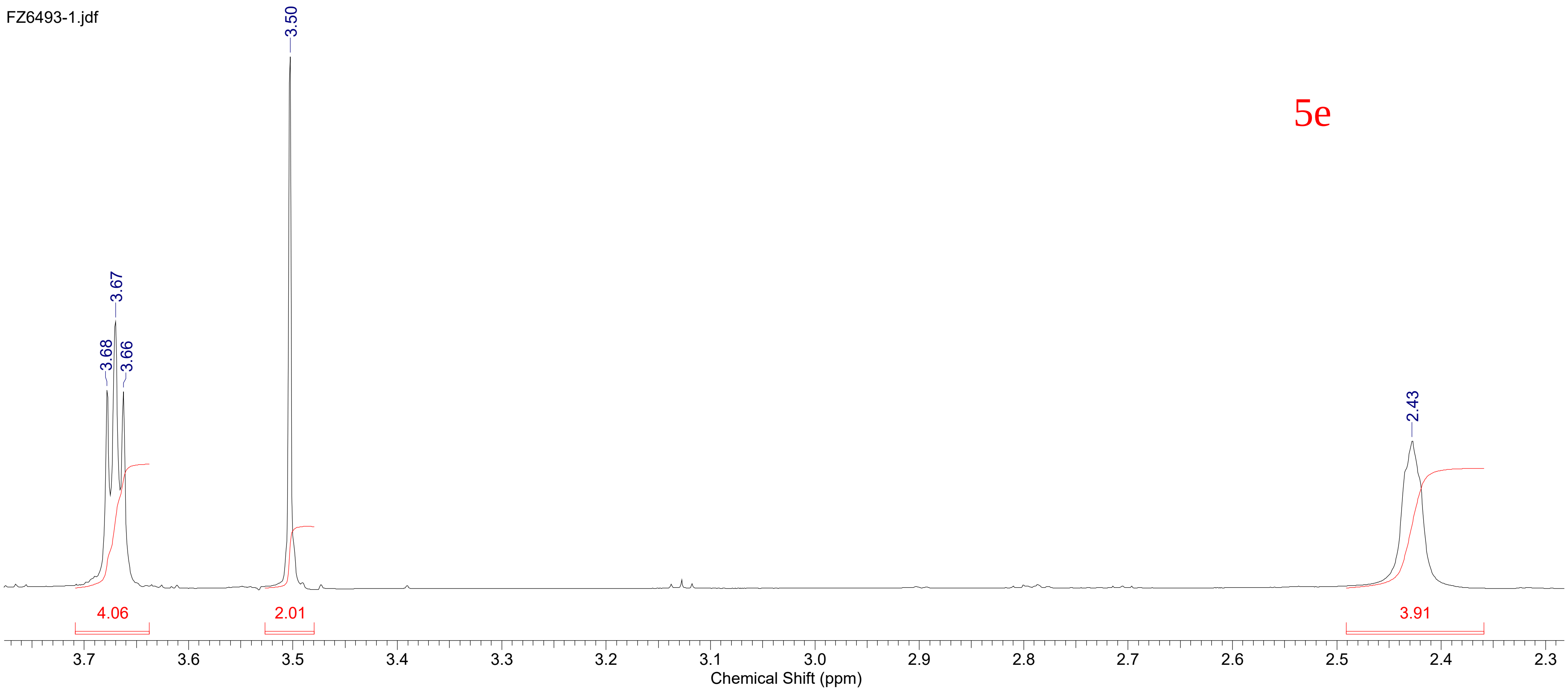

| | | | | | | | |
|---|---|---|---|---|---|---|---|
| ***Acquisition Time (sec)*** | 0.6921 | ***Comment*** | single pulse decoupled gated NOE | | | ***Date*** | 14 May 1990 08:53:09 |
| ***Date Stamp*** | 10 May 2018 13:40:28 | | | ***File Name*** | C:\Users\Fedor\Desktop\08.05.18\FZ6701-1.jdf | | |
| ***Frequency (MHz)*** | 150.91 | ***Nucleus*** | 13C | ***Number of Transients*** | 1000 | ***Origin*** | ECA 600 |
| ***Original Points Count*** | 32768 | ***Owner*** | delta | ***Points Count*** | 32768 | ***Pulse Sequence*** | single_pulse_dec |
| ***Receiver Gain*** | 52.00 | ***Solvent*** | CHLOROFORM-d | | | ***Spectrum Offset (Hz)*** | 15091.3428 |
| ***Sweep Width (Hz)*** | 47348.49 | ***Temperature (degree C)*** | 22.300 | | | | |

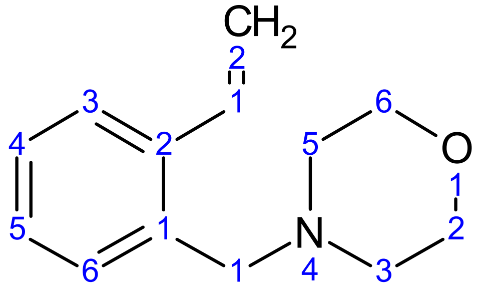


5e

FZ6701-1.jdf

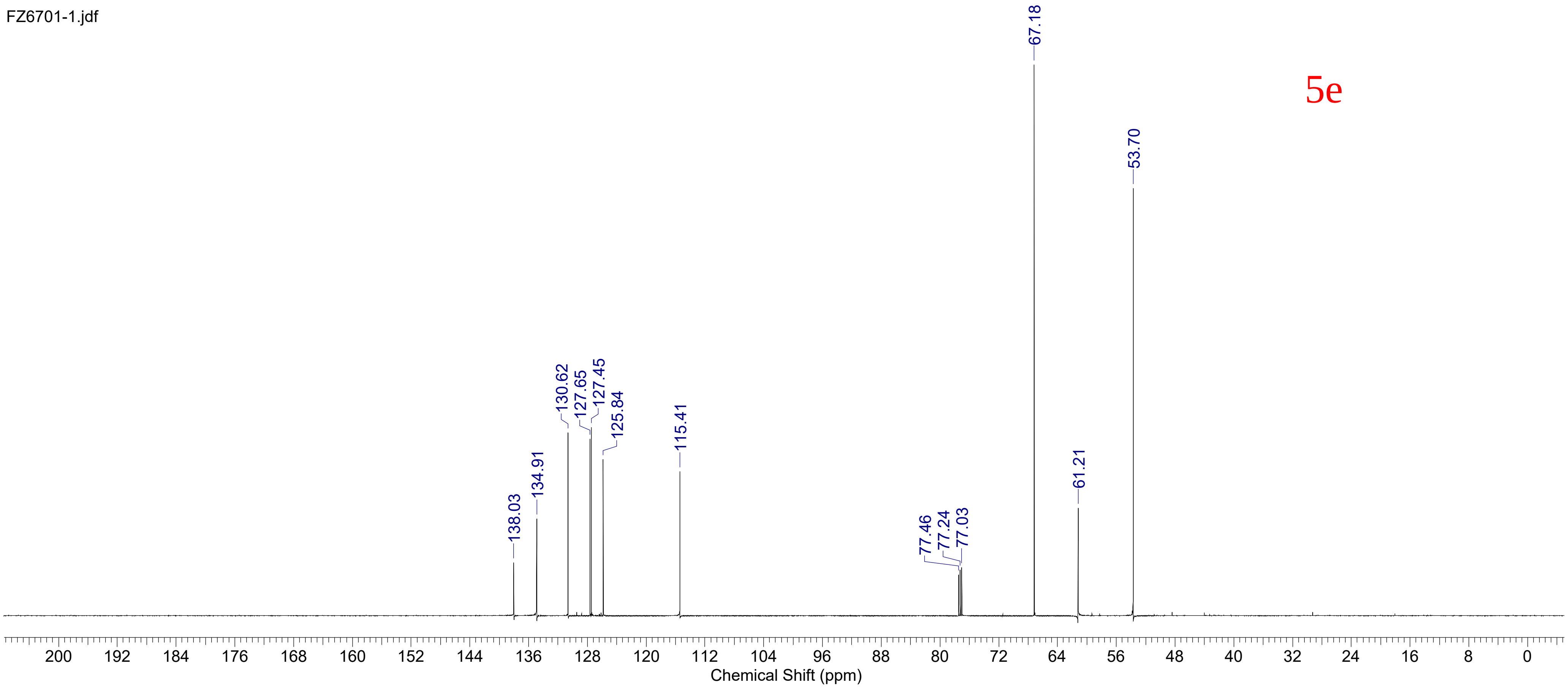

| Acquisition Time (sec) | 0.6921 | Comment | single pulse decoupled gated NOE | | | Date | 14 May 1990 08:53:09 |
|---|---|---|---|---|---|---|---|
| Date Stamp | 10 May 2018 13:40:28 | | | File Name | C:\Users\Fedor\Desktop\08.05.18\FZ6701-1.jdf | | |
| Frequency (MHz) | 150.91 | Nucleus | 13C | Number of Transients | 1000 | Origin | ECA 600 |
| Original Points Count | 32768 | Owner | delta | Points Count | 32768 | Pulse Sequence | single_pulse_dec |
| Receiver Gain | 52.00 | Solvent | CHLOROFORM-d | | | Spectrum Offset (Hz) | 15091.3428 |
| Sweep Width (Hz) | 47348.49 | Temperature (degree C) | 22.300 | | | | |

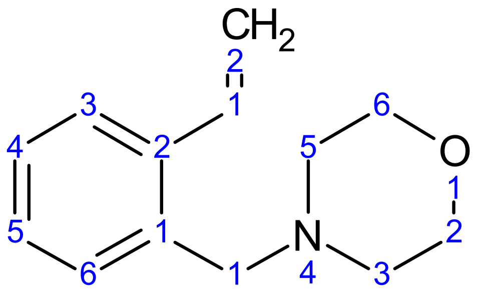


FZ6701-1.jdf

5e

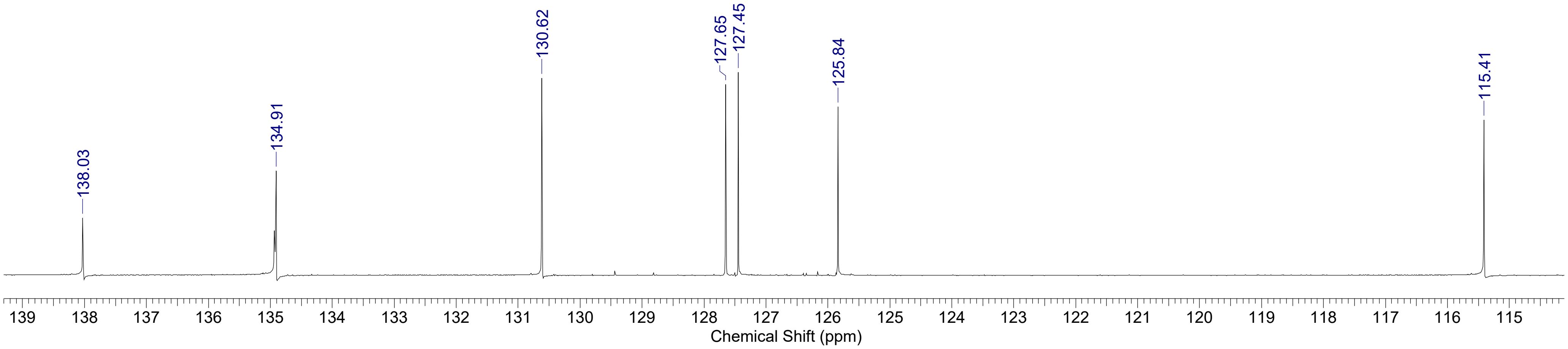

| | | | | | | | |
|---|---|---|---|---|---|---|---|
| ***Acquisition Time (sec)*** | 0.6921 | ***Comment*** | single pulse decoupled gated NOE | | | ***Date*** | 14 May 1990 08:53:09 |
| ***Date Stamp*** | 10 May 2018 13:40:28 | | | ***File Name*** | C:\Users\Fedor\Desktop\08.05.18\FZ6701-1.jdf | | |
| ***Frequency (MHz)*** | 150.91 | ***Nucleus*** | 13C | ***Number of Transients*** | 1000 | ***Origin*** | ECA 600 |
| ***Original Points Count*** | 32768 | ***Owner*** | delta | ***Points Count*** | 32768 | ***Pulse Sequence*** | single_pulse_dec |
| ***Receiver Gain*** | 52.00 | ***Solvent*** | CHLOROFORM-d | | | ***Spectrum Offset (Hz)*** | 15091.3428 |
| ***Sweep Width (Hz)*** | 47348.49 | ***Temperature (degree C)*** | 22.300 | | | | |

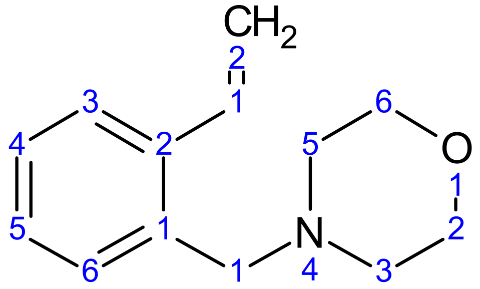


5e

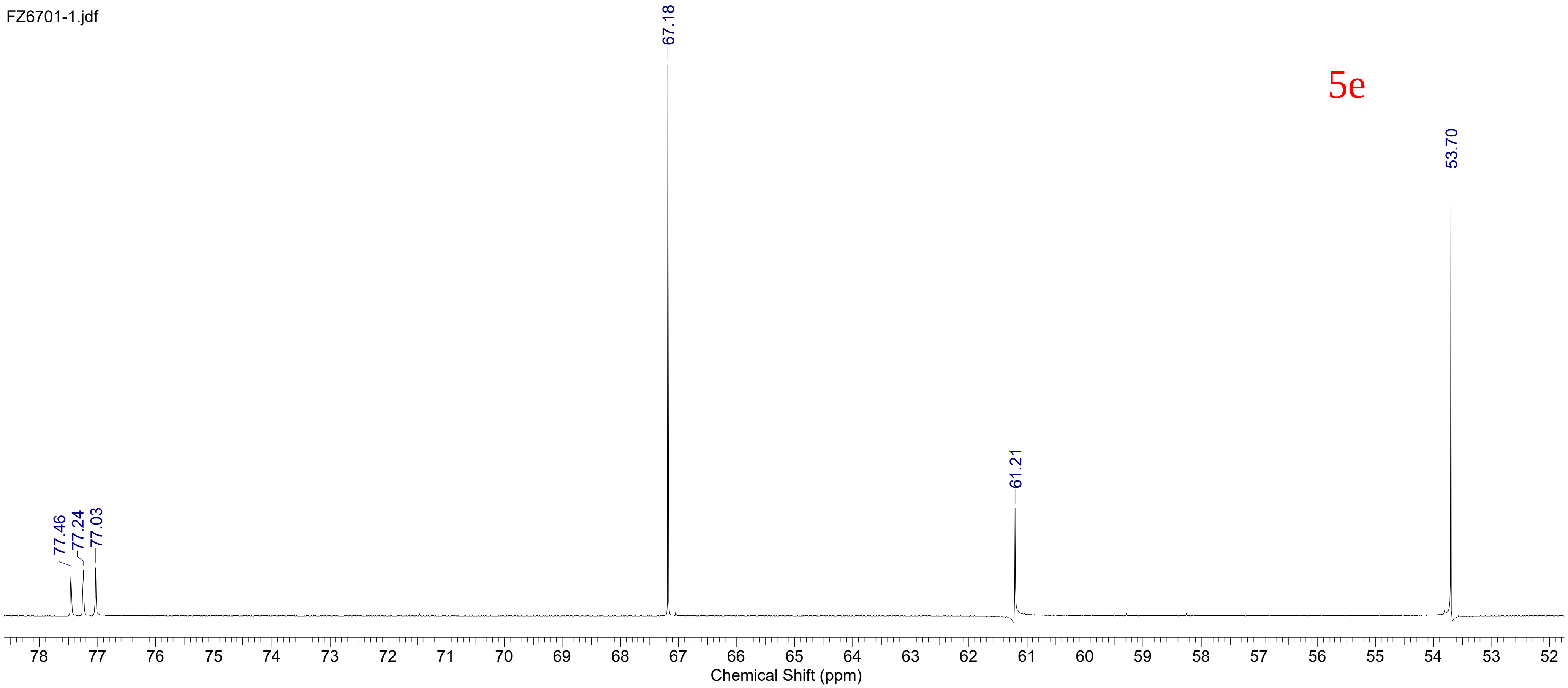

| | | | | | | | |
|---|---|---|---|---|---|---|---|
| ***Acquisition Time (sec)*** | 0.6921 | ***Comment*** | single pulse decoupled gated NOE | | | ***Date*** | 14 May 1990 08:53:09 |
| ***Date Stamp*** | 10 May 2018 13:40:28 | | | ***File Name*** | C:\Users\Fedor\Desktop\08.05.18\FZ6701-1.jdf | | |
| ***Frequency (MHz)*** | 150.91 | ***Nucleus*** | 13C | ***Number of Transients*** | 1000 | ***Origin*** | ECA 600 |
| ***Original Points Count*** | 32768 | ***Owner*** | delta | ***Points Count*** | 32768 | ***Pulse Sequence*** | single_pulse_dec |
| ***Receiver Gain*** | 52.00 | ***Solvent*** | CHLOROFORM-d | | | ***Spectrum Offset (Hz)*** | 15091.3428 |
| ***Sweep Width (Hz)*** | 47348.49 | ***Temperature (degree C)*** | 22.300 | | | | |

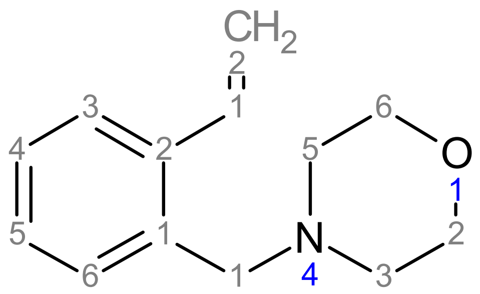

5e

FZ6701-1.jdf

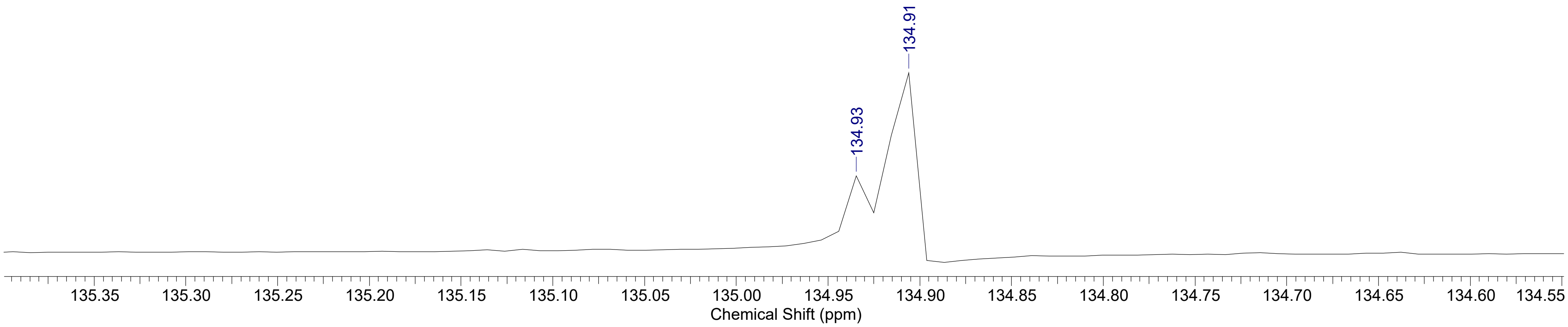

| Acquisition Time (sec) | 2.7263 | Comment | FZ6892 | Date | 01 Oct 2018 17:46:40 | | | Date Stamp | 01 Oct 2018 17:46:40 |
|---|---|---|---|---|---|---|---|---|---|
| File Name | C:\Users\Fedor\Desktop\01.10.18\FZ6892\FZ6892_001000fid | | | | | Frequency (MHz) | 300.13 | Nucleus | 1H |
| Number of Transients | 64 | Origin | spect | Original Points Count | 32768 | Owner | nmr | Points Count | 32768 |
| Pulse Sequence | zg | Receiver Gain | 202.48 | SW(cyclical) (Hz) | 12019.23 | Solvent | DMSO-d6 | Spectrum Offset (Hz) | 1348.1652 |
| Sweep Width (Hz) | 12018.86 | Temperature (degree C) | 29.999 | | | | | | |

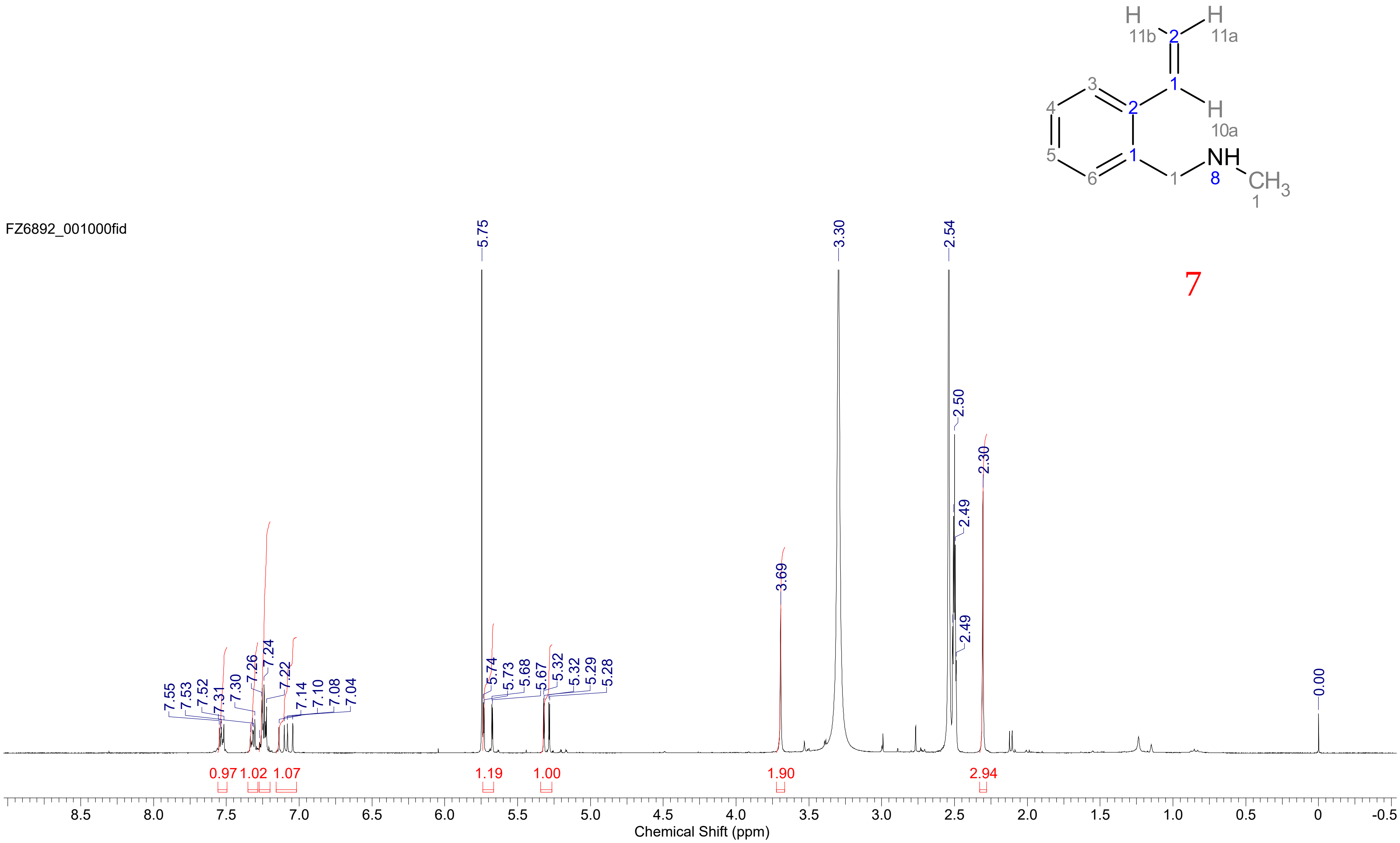

| | | | | | | | |
|---|---|---|---|---|---|---|---|
| ***Acquisition Time (sec)*** | 2.7263 | ***Comment*** | FZ6892 | ***Date*** | 01 Oct 2018 17:46:40 | ***Date Stamp*** | 01 Oct 2018 17:46:40 |
| ***File Name*** | C:\Users\Fedor\Desktop\01.10.18\FZ6892\FZ6892_001000fid | | | ***Frequency (MHz)*** | 300.13 | ***Nucleus*** | 1H |
| ***Number of Transients*** | 64 | ***Origin*** | spect | ***Original Points Count*** | 32768 | ***Owner*** nmr | ***Points Count*** 32768 |
| ***Pulse Sequence*** | zg | ***Receiver Gain*** | 202.48 | ***SW(cyclical) (Hz)*** | 12019.23 | ***Solvent*** DMSO-d6 | ***Spectrum Offset (Hz)*** 1348.1652 |
| ***Sweep Width (Hz)*** | 12018.86 | ***Temperature (degree C)*** | 29.999 | | | | |

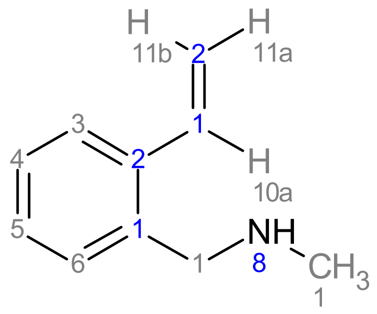

7

FZ6892_001000fid

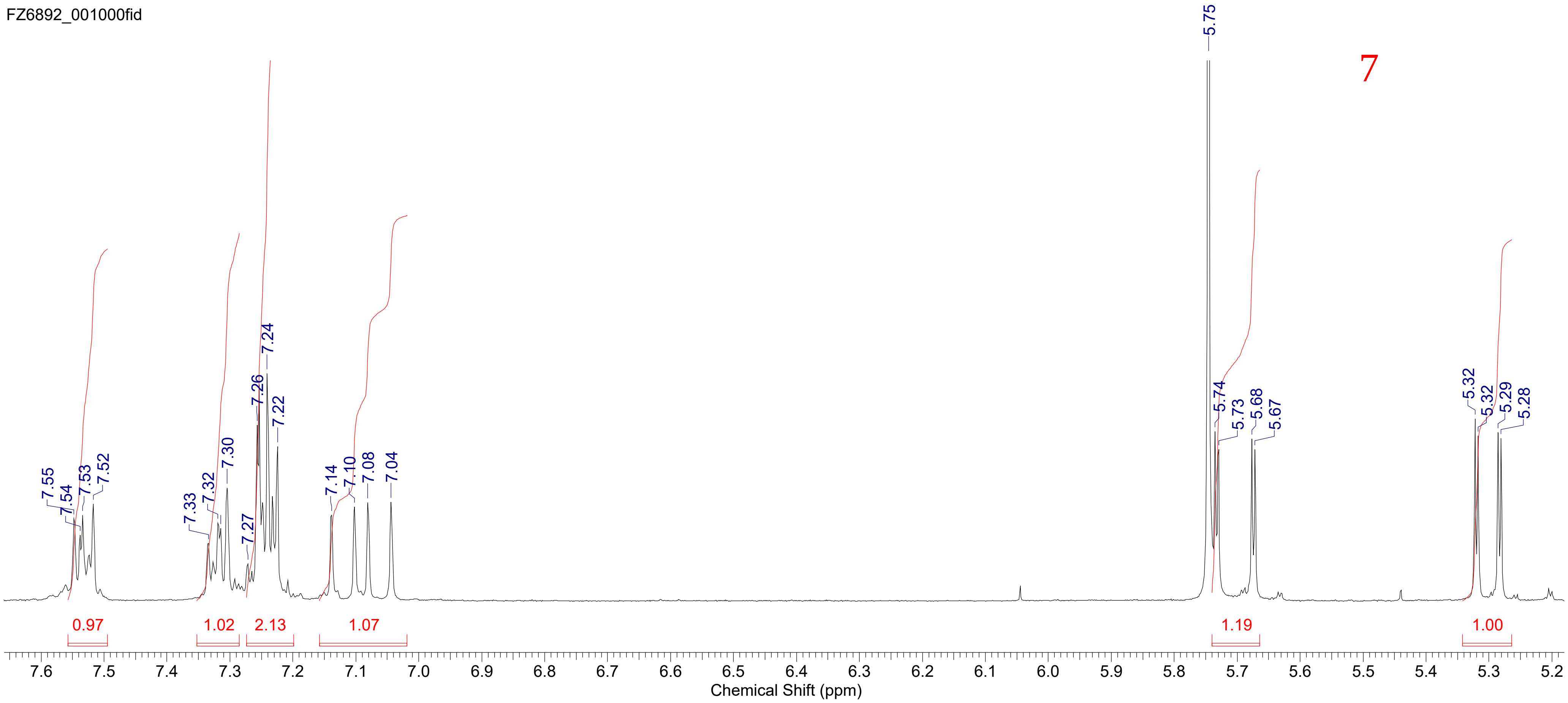

| | | | | | | | |
|---|---|---|---|---|---|---|---|
| ***Acquisition Time (sec)*** | 2.7263 | ***Comment*** | FZ6892 | ***Date*** | 01 Oct 2018 17:46:40 | ***Date Stamp*** | 01 Oct 2018 17:46:40 |
| ***File Name*** | C:\Users\Fedor\Desktop\01.10.18\FZ6892\FZ6892_001000fid | ***Frequency (MHz)*** | 300.13 | | | ***Nucleus*** | 1H |
| ***Number of Transients*** | 64 | ***Origin*** | spect | ***Original Points Count*** | 32768 | ***Owner*** nmr | ***Points Count*** 32768 |
| ***Pulse Sequence*** | zg | ***Receiver Gain*** | 202.48 | ***SW(cyclical) (Hz)*** | 12019.23 | ***Solvent*** DMSO-d6 | ***Spectrum Offset (Hz)*** 1348.1652 |
| ***Sweep Width (Hz)*** | 12018.86 | ***Temperature (degree C)*** | 29.999 | | | | |

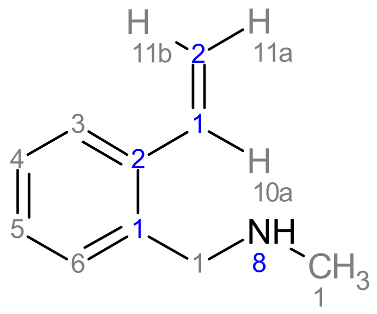

7

FZ6892_001000fid

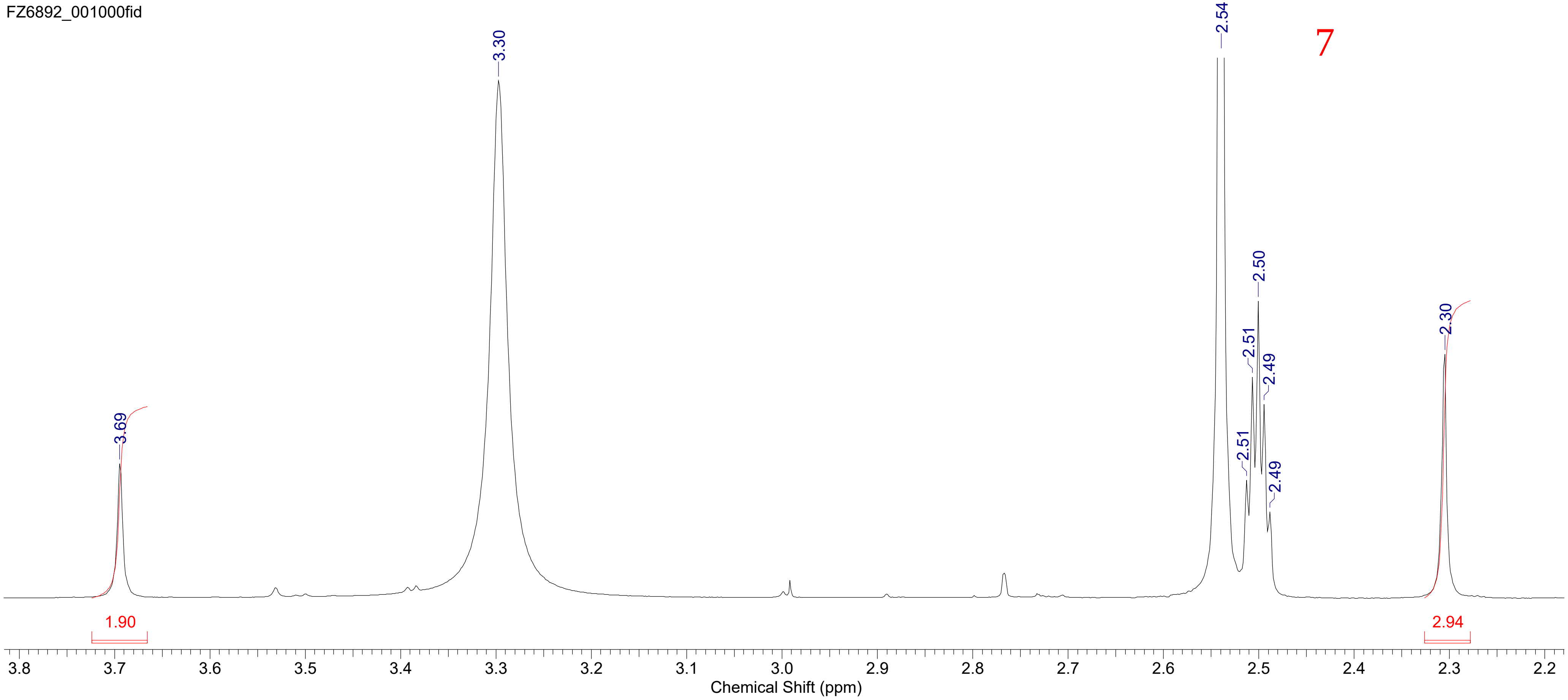

| | | | | | |
|---|---|---|---|---|---|
| ***Acquisition Time (sec)*** | 0.8561 | ***Comment*** | FZ6892-c13dec-night | ***Date*** | 01 Oct 2018 19:03:28 |
| ***Date Stamp*** | 01 Oct 2018 19:03:28 | ***File Name*** | C:\Users\Fedor\Desktop\01.10.18\FZ6892-c13dec-night\FZ6892-c13dec-night_013000fid | | |
| ***Frequency (MHz)*** | 75.48 | ***Nucleus*** | 13C | ***Number of Transients*** | 20480 |
| ***Origin*** | spect | ***Original Points Count*** | 22528 | ***Owner*** | nmr |
| ***Points Count*** | 32768 | ***Pulse Sequence*** | zgpg | ***Receiver Gain*** | 202.48 |
| ***SW(cyclical) (Hz)*** | 26315.79 | ***Solvent*** | DMSO-d6 | ***Spectrum Offset (Hz)*** | 7548.8423 |
| ***Sweep Width (Hz)*** | 26314.99 | ***Temperature (degree C)*** | 29.991 | | |

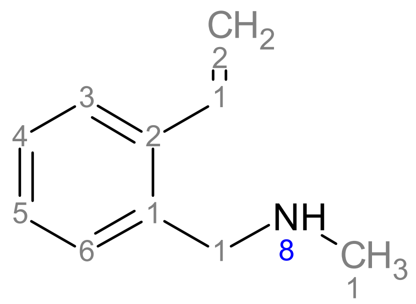

FZ6892-c13dec-night_013000fid

7

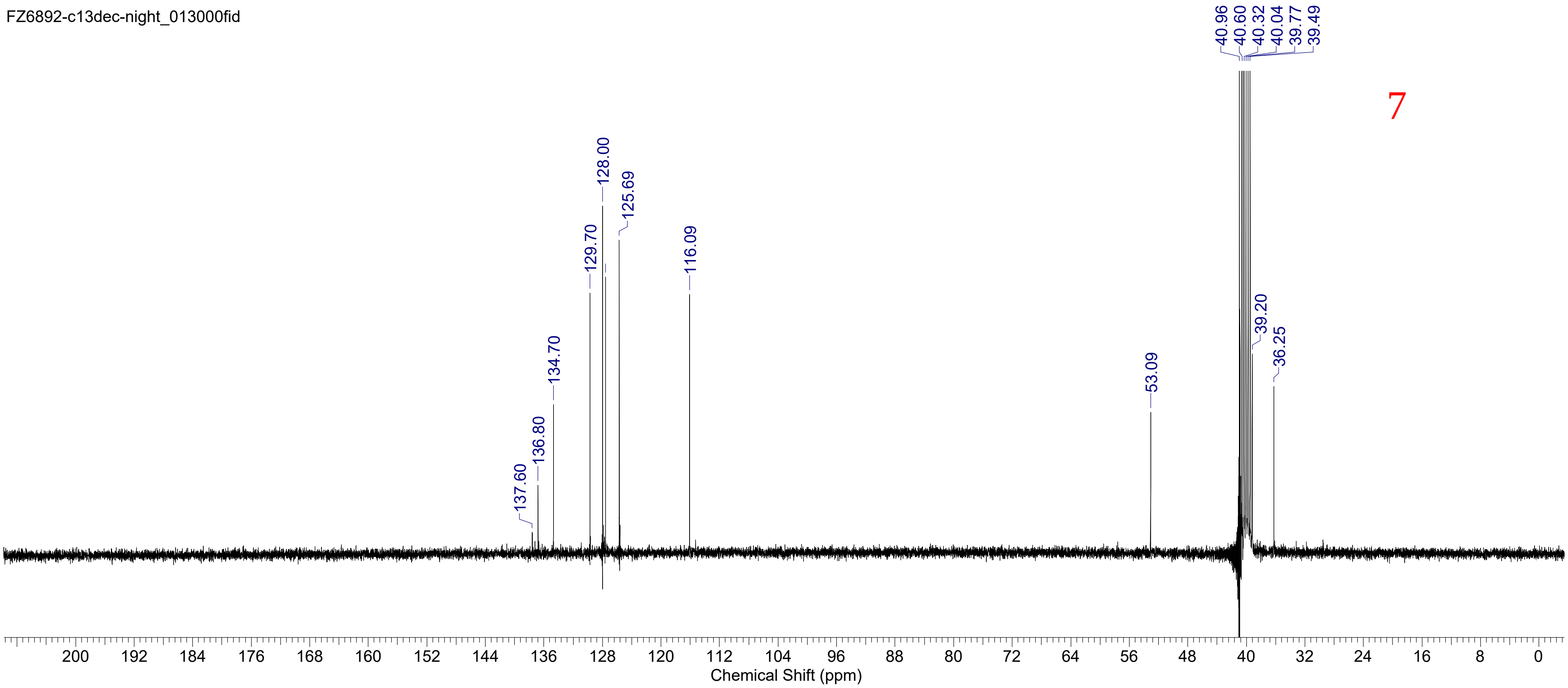

| | | | | | | | |
|---|---|---|---|---|---|---|---|
| ***Acquisition Time (sec)*** | 0.8561 | ***Comment*** | FZ6892-c13dec-night | | | ***Date*** | 01 Oct 2018 19:03:28 |
| ***Date Stamp*** | 01 Oct 2018 19:03:28 | | | ***File Name*** | C:\Users\Fedor\Desktop\01.10.18\FZ6892-c13dec-night\FZ6892-c13dec-night_013000fid | | |
| ***Frequency (MHz)*** | 75.48 | ***Nucleus*** | 13C | ***Number of Transients*** | 20480 | ***Origin*** | spect |
| ***Original Points Count*** | 22528 | ***Owner*** | nmr | ***Points Count*** | 32768 | ***Pulse Sequence*** | zgpg |
| ***Receiver Gain*** | 202.48 | ***SW(cyclical) (Hz)*** | 26315.79 | ***Solvent*** | DMSO-d6 | ***Spectrum Offset (Hz)*** | 7548.8423 |
| ***Sweep Width (Hz)*** | 26314.99 | ***Temperature (degree C)*** | 29.991 | | | | |

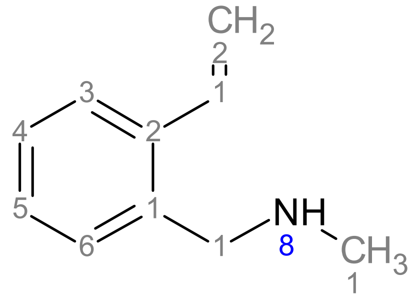

7

FZ6892-c13dec-night_013000fid

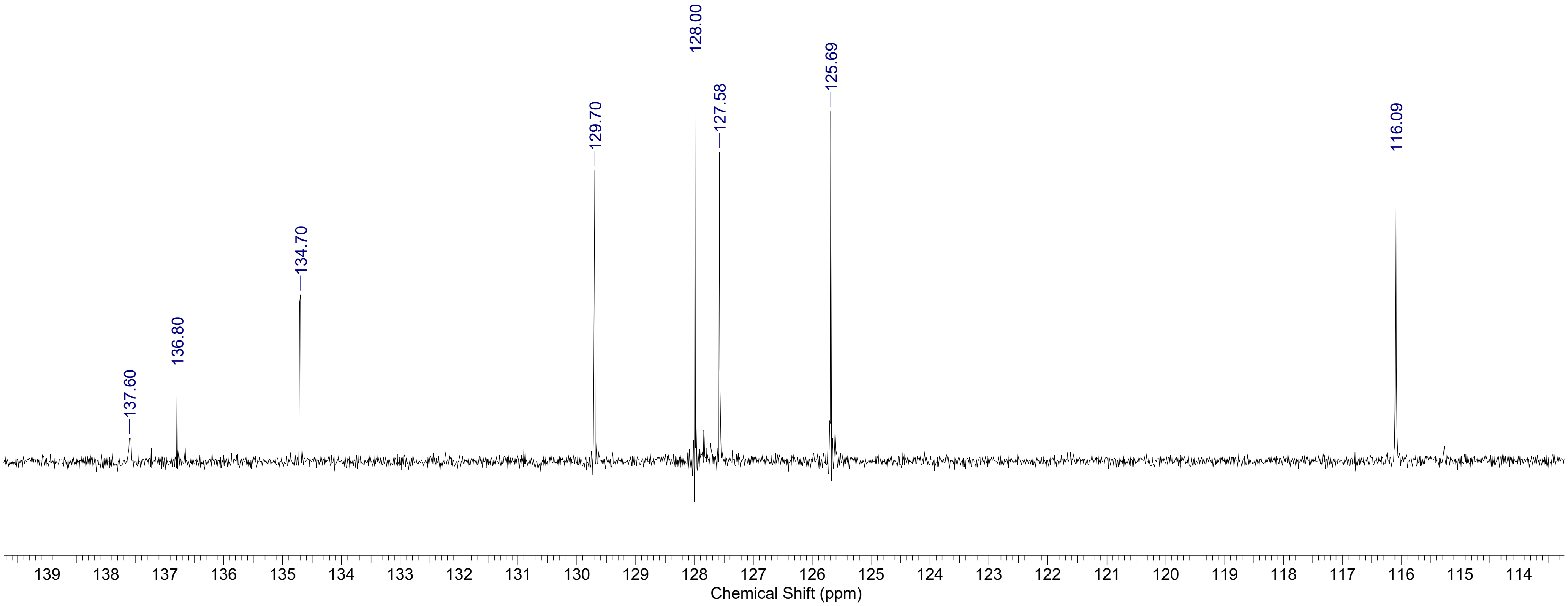

| | | | | | |
|---|---|---|---|---|---|
| ***Acquisition Time (sec)*** | 0.8561 | ***Comment*** | FZ6892-c13dec-night | ***Date*** | 01 Oct 2018 19:03:28 |
| ***Date Stamp*** | 01 Oct 2018 19:03:28 | ***File Name*** | C:\Users\Fedor\Desktop\01.10.18\FZ6892-c13dec-night\FZ6892-c13dec-night_013000fid | | |
| ***Frequency (MHz)*** | 75.48 | ***Nucleus*** | 13C | ***Number of Transients*** | 20480 |
| ***Origin*** | spect | ***Original Points Count*** | 22528 | ***Owner*** | nmr |
| ***Points Count*** | 32768 | ***Pulse Sequence*** | zgpg | ***Receiver Gain*** | 202.48 |
| ***SW(cyclical) (Hz)*** | 26315.79 | ***Solvent*** | DMSO-d6 | ***Spectrum Offset (Hz)*** | 7548.8423 |
| ***Sweep Width (Hz)*** | 26314.99 | ***Temperature (degree C)*** | 29.991 | | |

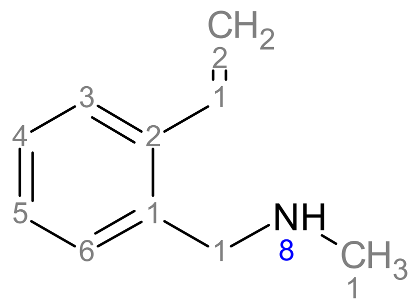


7

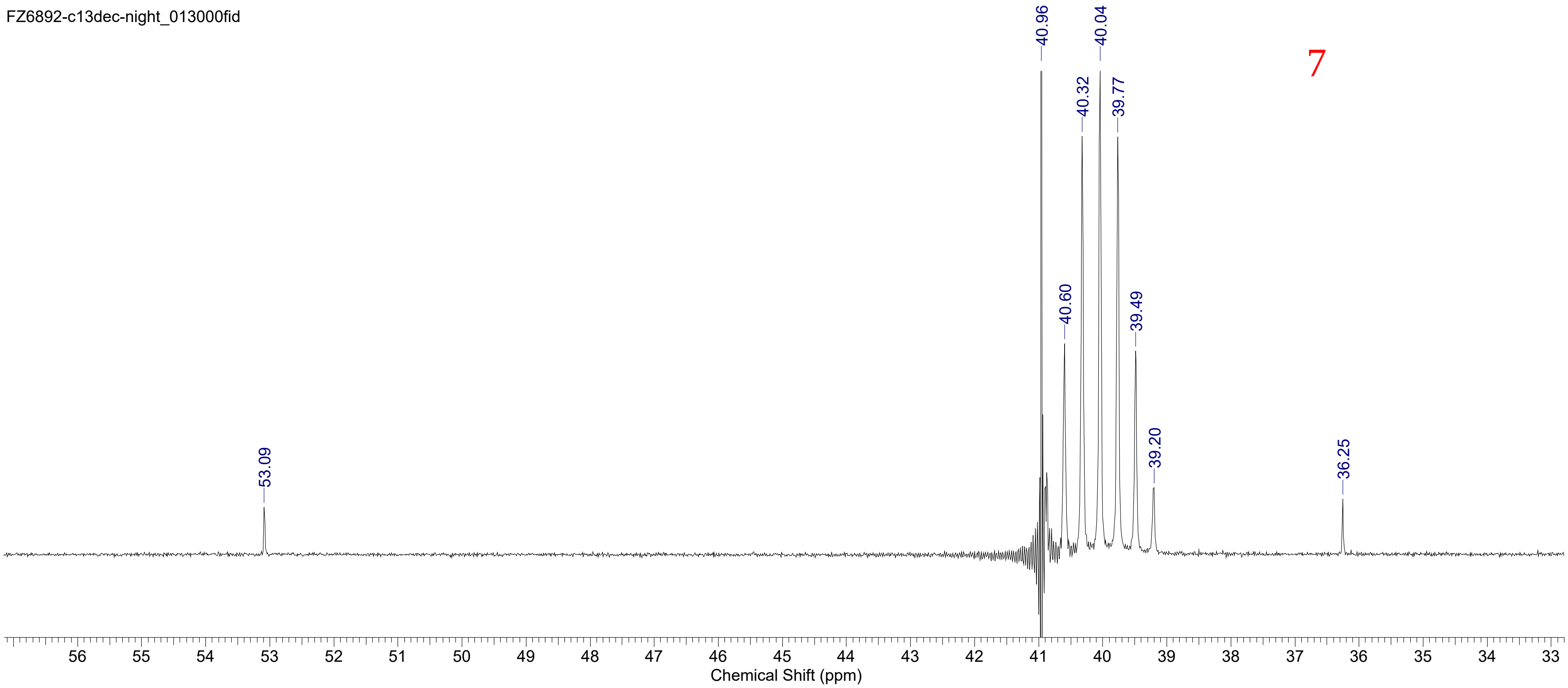

| | | | | | | | |
|---|---|---|---|---|---|---|---|
| ***Acquisition Time (sec)*** | 1.9818 | ***Comment*** | single_pulse | ***Date*** | 10 Jun 1990 05:09:58 | | |
| ***Date Stamp*** | 06 Jun 2018 09:56:41 | | | ***File Name*** | C:\Users\Fedor\Desktop\05.06.18\FZ6761-1.jdf | ***Frequency (MHz)*** | 600.17 |
| ***Nucleus*** | 1H | ***Number of Transients*** | 8 | ***Origin*** | ECA 600 | ***Original Points Count*** | 32768 |
| ***Owner*** | delta | ***Points Count*** | 32768 | ***Pulse Sequence*** | single_pulse.ex2 | ***Receiver Gain*** | 26.00 |
| ***Solvent*** | DMSO-d6 | ***Spectrum Offset (Hz)*** | 5401.5503 | ***Sweep Width (Hz)*** | 16534.39 | ***Temperature (degree C)*** | 21.100 |

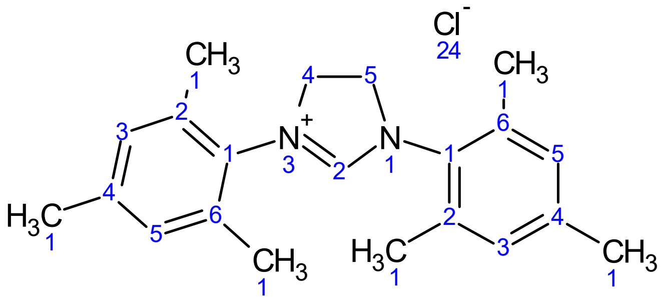

FZ6761-1.jdf

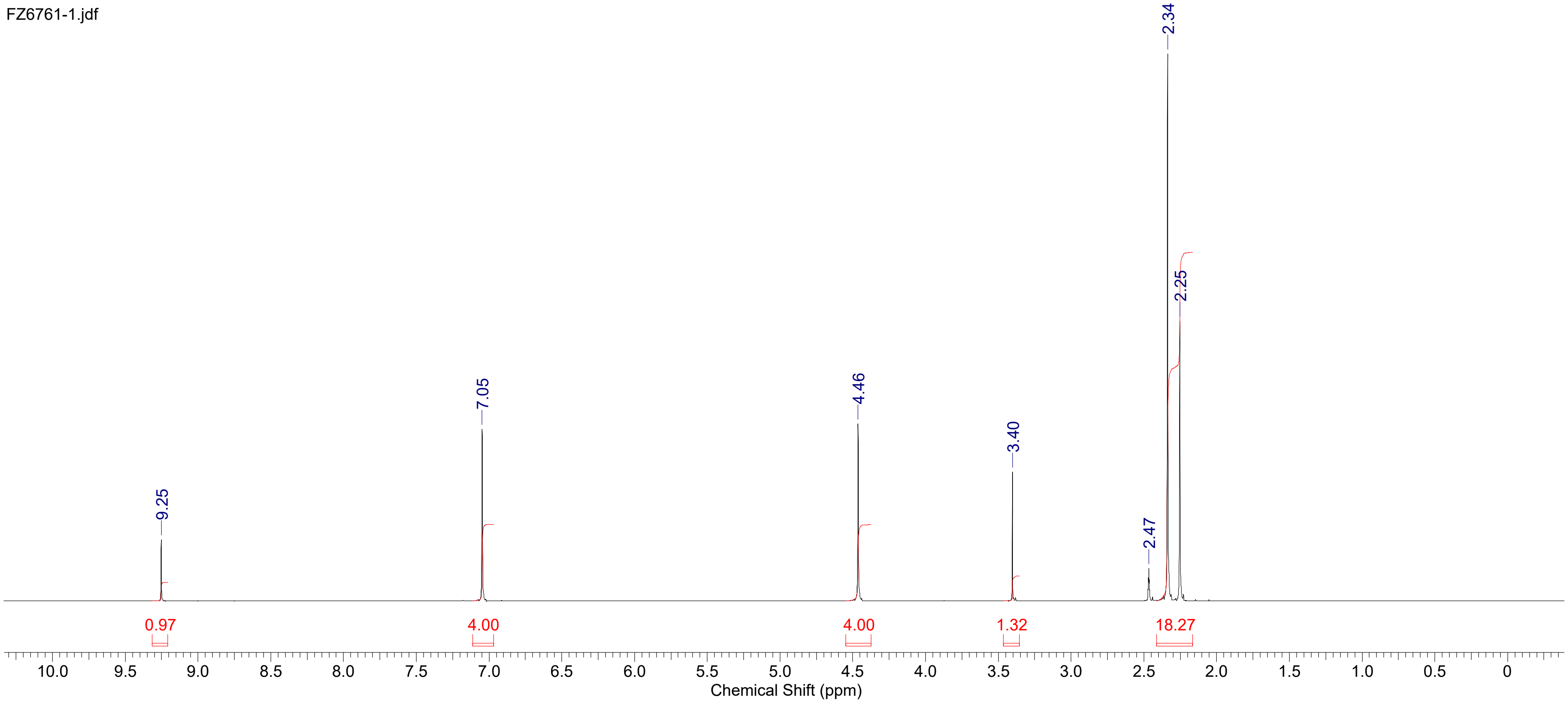

| | | | | | | | |
|---|---|---|---|---|---|---|---|
| ***Acquisition Time (sec)*** | 0.6921 | ***Comment*** | single pulse decoupled gated NOE | | | ***Date*** | 17 Jun 1990 13:43:07 |
| ***Date Stamp*** | 14 Jun 2018 00:17:21 | | | ***File Name*** | C:\Users\Fedor\Desktop\12.06.18\FZ6770-1.jdf | | |
| ***Frequency (MHz)*** | 150.91 | ***Nucleus*** | 13C | ***Number of Transients*** | 1000 | ***Origin*** | ECA 600 |
| ***Original Points Count*** | 32768 | ***Owner*** | delta | ***Points Count*** | 32768 | ***Pulse Sequence*** | single_pulse_dec |
| ***Receiver Gain*** | 54.00 | ***Solvent*** | DMSO-d6 | ***Spectrum Offset (Hz)*** | 15091.3428 | ***Sweep Width (Hz)*** | 47348.49 |
| ***Temperature (degree C)*** | 21.300 | | | | | | |

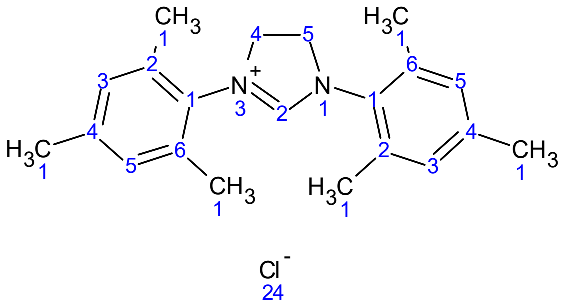


FZ6770-1.jdf

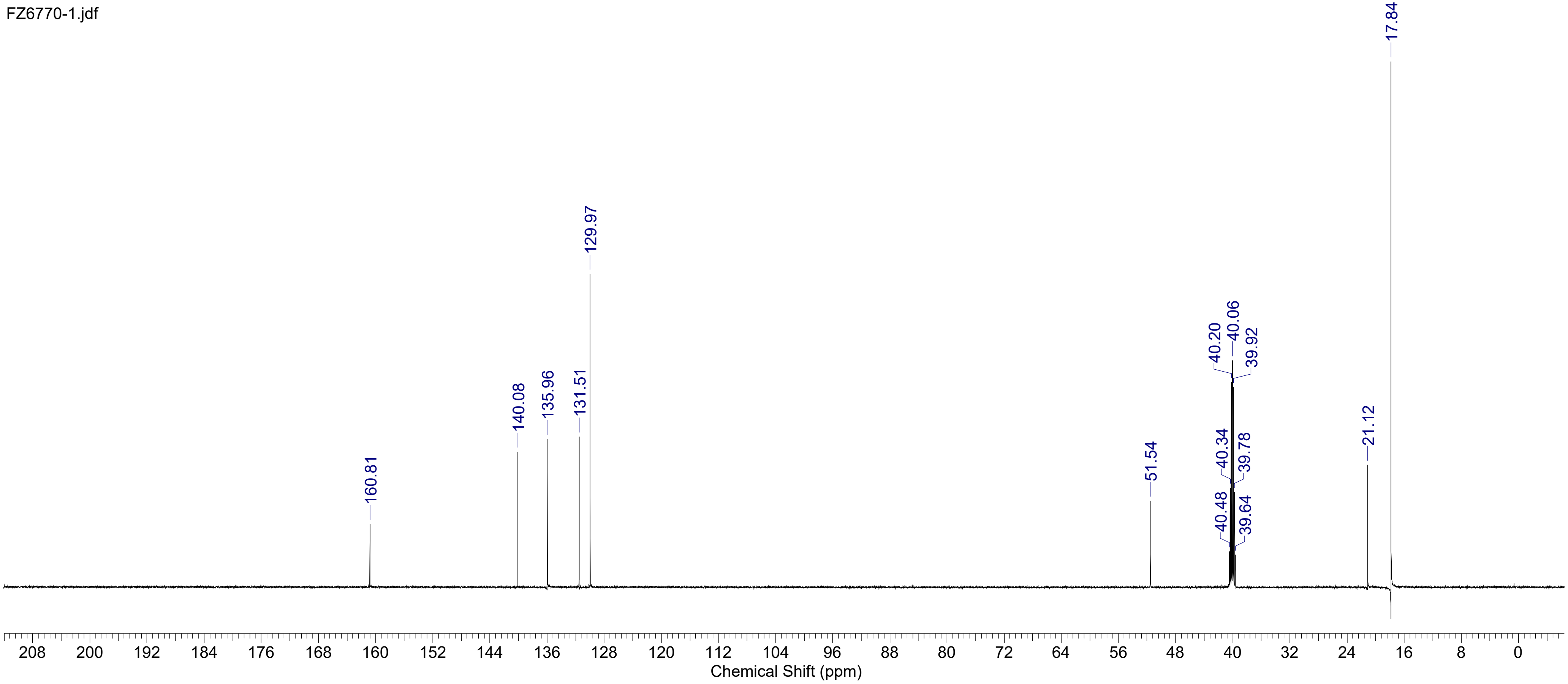

| | | | | | | | |
|---|---|---|---|---|---|---|---|
| ***Acquisition Time (sec)*** | 0.6921 | ***Comment*** | single pulse decoupled gated NOE | | | ***Date*** | 17 Jun 1990 13:43:07 |
| ***Date Stamp*** | 14 Jun 2018 00:17:21 | | | ***File Name*** | C:\Users\Fedor\Desktop\12.06.18\FZ6770-1.jdf | | |
| ***Frequency (MHz)*** | 150.91 | ***Nucleus*** | 13C | ***Number of Transients*** | 1000 | ***Origin*** | ECA 600 |
| ***Original Points Count*** | 32768 | ***Owner*** | delta | ***Points Count*** | 32768 | ***Pulse Sequence*** | single_pulse_dec |
| ***Receiver Gain*** | 54.00 | ***Solvent*** | DMSO-d6 | ***Spectrum Offset (Hz)*** | 15091.3428 | ***Sweep Width (Hz)*** | 47348.49 |
| ***Temperature (degree C)*** | 21.300 | | | | | | |

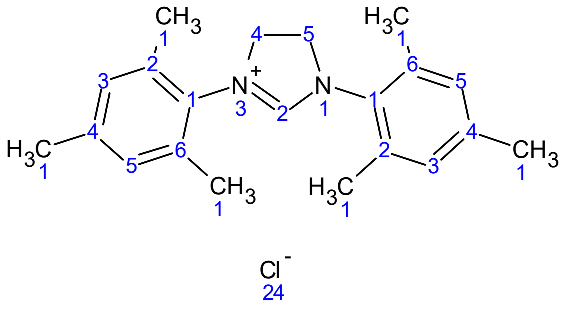

FZ6770-1.jdf

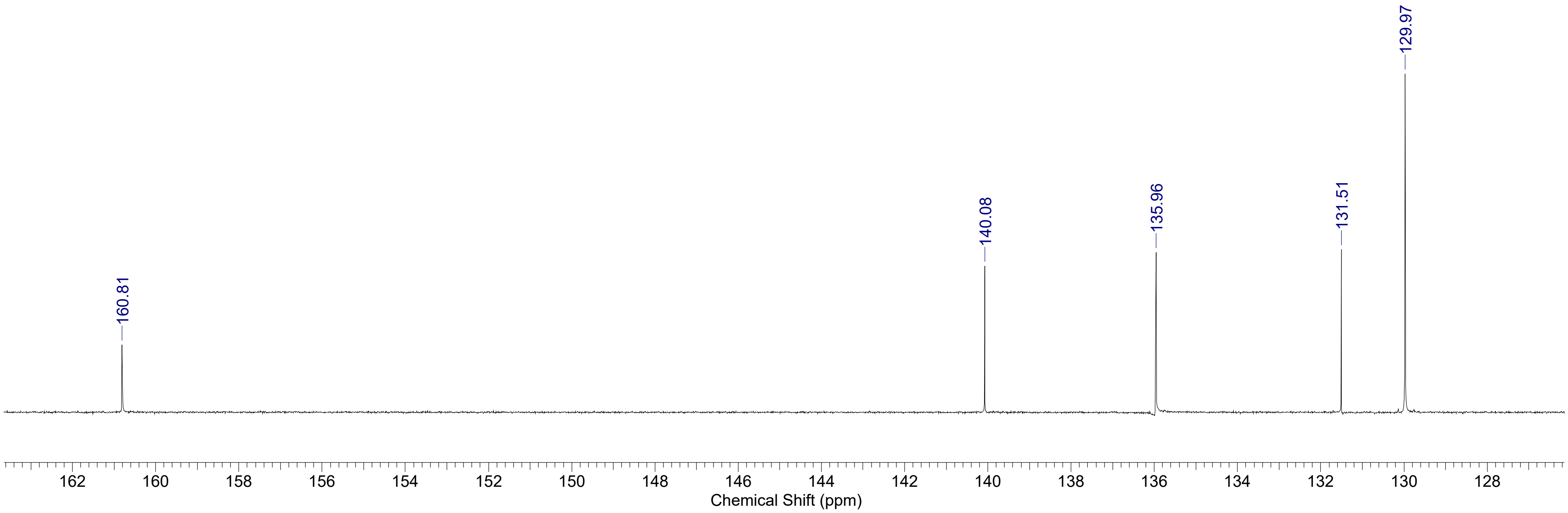

| | | | | | | | |
|---|---|---|---|---|---|---|---|
| ***Acquisition Time (sec)*** | 0.6921 | ***Comment*** | single pulse decoupled gated NOE | | | ***Date*** | 17 Jun 1990 13:43:07 |
| ***Date Stamp*** | 14 Jun 2018 00:17:21 | | | ***File Name*** | C:\Users\Fedor\Desktop\12.06.18\FZ6770-1.jdf | | |
| ***Frequency (MHz)*** | 150.91 | ***Nucleus*** | 13C | ***Number of Transients*** | 1000 | ***Origin*** | ECA 600 |
| ***Original Points Count*** | 32768 | ***Owner*** | delta | ***Points Count*** | 32768 | ***Pulse Sequence*** | single_pulse_dec |
| ***Receiver Gain*** | 54.00 | ***Solvent*** | DMSO-d6 | ***Spectrum Offset (Hz)*** | 15091.3428 | ***Sweep Width (Hz)*** | 47348.49 |
| ***Temperature (degree C)*** | 21.300 | | | | | | |

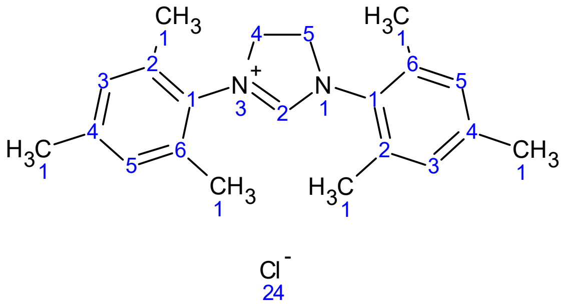


FZ6770-1.jdf

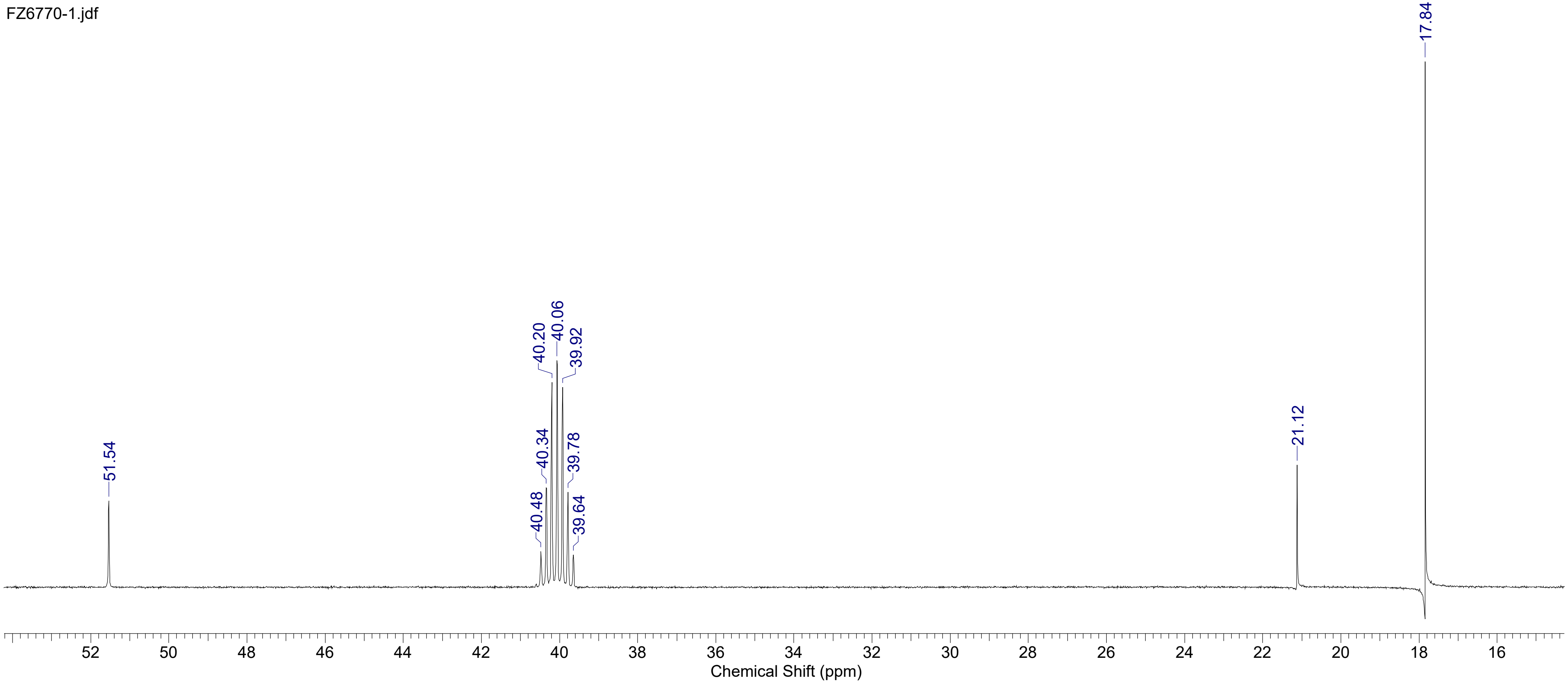

| | | | | | |
|---|---|---|---|---|---|
| ***Acquisition Time (sec)*** | 2.5690 | | | | |
| ***Comment*** | FZ_Grubbs_3_1 Mes2NHC-(cyclo)-RuCl2=CH-C6H4-CH2-NMe2- 26 mg / CD2Cl2 / Jan 12 Spectra - Jan 17, storage at -18C in CD2Cl2 solution | | | | |
| ***Date*** | 17 Jan 2018 16:25:36 | ***Date Stamp*** | 17 Jan 2018 16:25:36 | | |
| ***File Name*** | C:\Users\Fedor\Desktop\Наброски Статей\Кирилл Статья по Катализаторам Граббса\ЯМР Граббс\ЯМР Граббс от Ромы\Grubbs_3 NMe2_001001r | | | | |
| ***Frequency (MHz)*** | 300.13 | ***Nucleus*** | 1H | ***Number of Transients*** | 32 |
| ***Origin*** | spect | ***Original Points Count*** | 65536 | ***Owner*** | nmr |
| ***Points Count*** | 262144 | ***Pulse Sequence*** | zg | ***Receiver Gain*** | 89.51 |
| ***SW(cyclical) (Hz)*** | 25510.20 | ***Solvent*** | DICHLOROMETHANE-d2 | ***Spectrum Offset (Hz)*** | 1350.5919 |
| ***Sweep Width (Hz)*** | 25510.11 | ***Temperature (degree C)*** | 29.998 | | |

11a

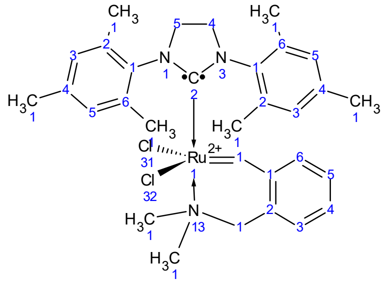

Grubbs_3 NMe2_001001r

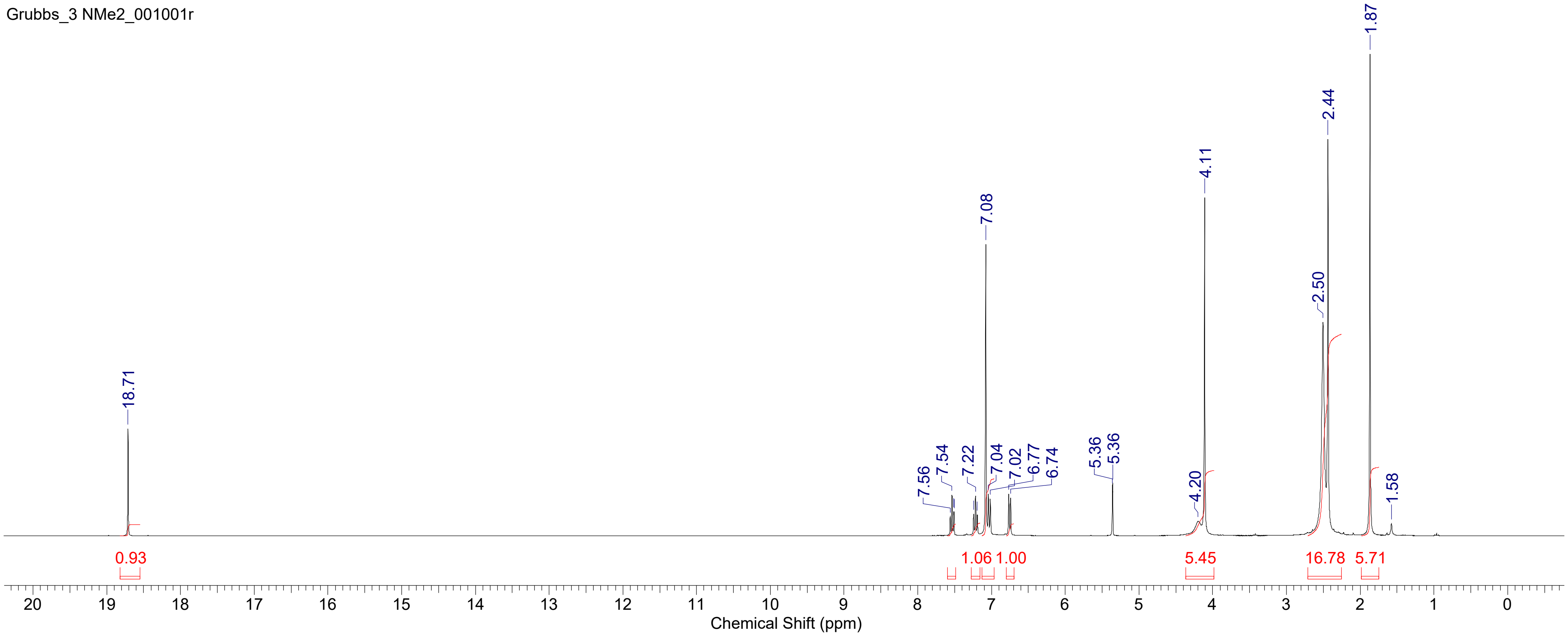

| | | | | | |
|---|---|---|---|---|---|
| ***Acquisition Time (sec)*** | 2.5690 | | | | |
| ***Comment*** | FZ_Grubbs_3_1 Mes2NHC-(cyclo)-RuCl2=CH-C6H4-CH2-NMe2- 26 mg / CD2Cl2 / Jan 12 Spectra - Jan 17, storage at -18C in CD2Cl2 solution | | | | |
| ***Date*** | 17 Jan 2018 16:25:36 | ***Date Stamp*** | 17 Jan 2018 16:25:36 | | |
| ***File Name*** | C:\Users\Fedor\Desktop\Наброски Статей\Кирилл Статья по Катализаторам Граббса\ЯМР Граббс\ЯМР Граббс от Ромы\Grubbs_3 NMe2_001001r | | | | |
| ***Frequency (MHz)*** | 300.13 | ***Nucleus*** | 1H | ***Number of Transients*** | 32 |
| ***Origin*** | spect | ***Original Points Count*** | 65536 | ***Owner*** | nmr |
| ***Points Count*** | 262144 | ***Pulse Sequence*** | zg | ***Receiver Gain*** | 89.51 |
| ***SW(cyclical) (Hz)*** | 25510.20 | ***Solvent*** | DICHLOROMETHANE-d2 | ***Spectrum Offset (Hz)*** | 1350.5919 |
| ***Sweep Width (Hz)*** | 25510.11 | ***Temperature (degree C)*** | 29.998 | | |

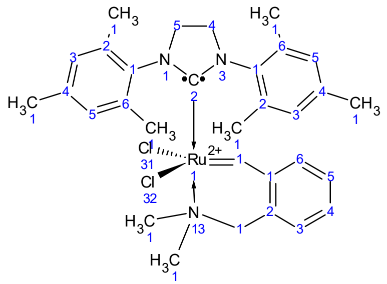

11a

Grubbs_3 NMe2_001001r

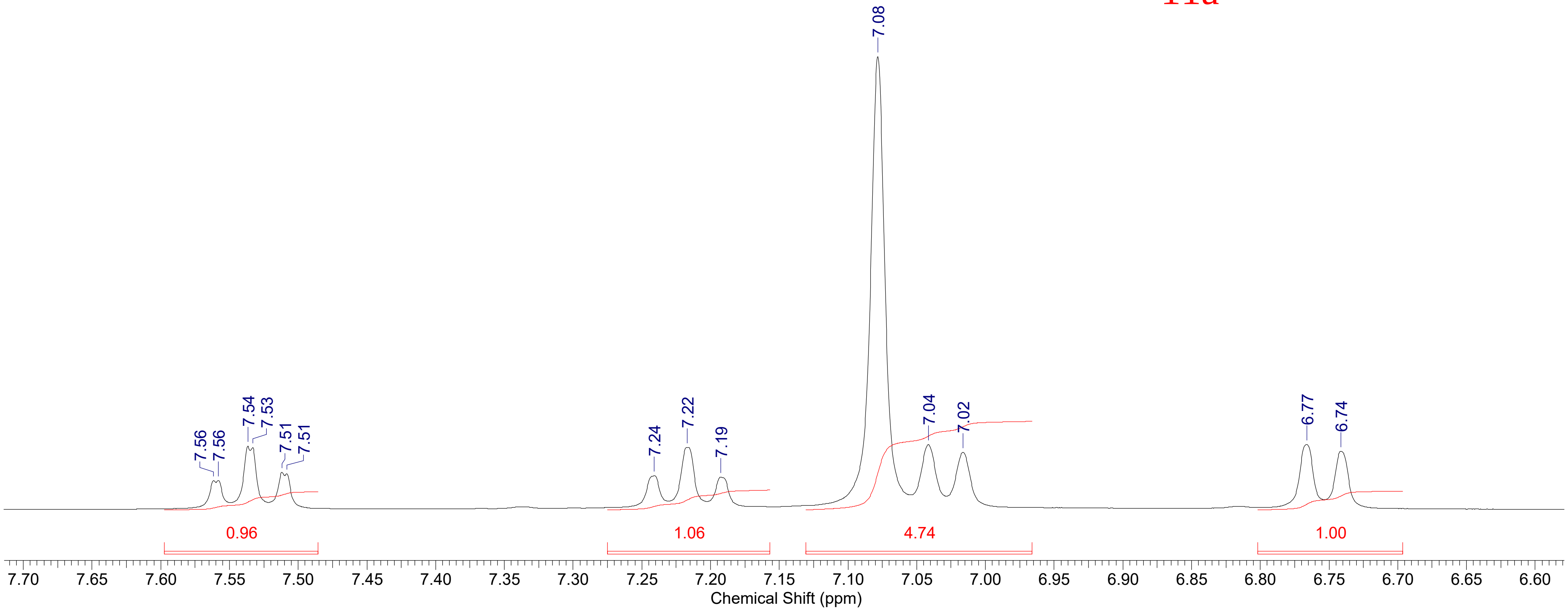

| | | | | | |
|---|---|---|---|---|---|
| ***Acquisition Time (sec)*** | 2.5690 | | | | |
| ***Comment*** | FZ_Grubbs_3_1 Mes2NHC-(cyclo)-RuCl2=CH-C6H4-CH2-NMe2- 26 mg / CD2Cl2 / Jan 12 Spectra - Jan 17, storage at -18C in CD2Cl2 solution | | | | |
| ***Date*** | 17 Jan 2018 16:25:36 | ***Date Stamp*** | 17 Jan 2018 16:25:36 | | |
| ***File Name*** | C:\Users\Fedor\Desktop\Наброски Статей\Кирилл Статья по Катализаторам Граббса\ЯМР Граббс\ЯМР Граббс от Ромы\Grubbs_3 NMe2_001001r | | | | |
| ***Frequency (MHz)*** | 300.13 | ***Nucleus*** | 1H | ***Number of Transients*** | 32 |
| ***Origin*** | spect | ***Original Points Count*** | 65536 | ***Owner*** | nmr |
| ***Points Count*** | 262144 | ***Pulse Sequence*** | zg | ***Receiver Gain*** | 89.51 |
| ***SW(cyclical) (Hz)*** | 25510.20 | ***Solvent*** | DICHLOROMETHANE-d2 | ***Spectrum Offset (Hz)*** | 1350.5919 |
| ***Sweep Width (Hz)*** | 25510.11 | ***Temperature (degree C)*** | 29.998 | | |

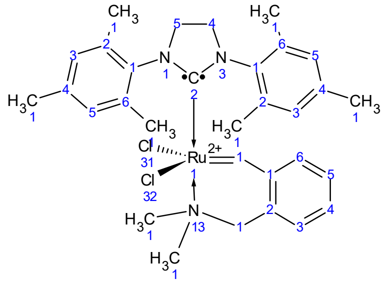

Grubbs_3 NMe2_001001r

11a

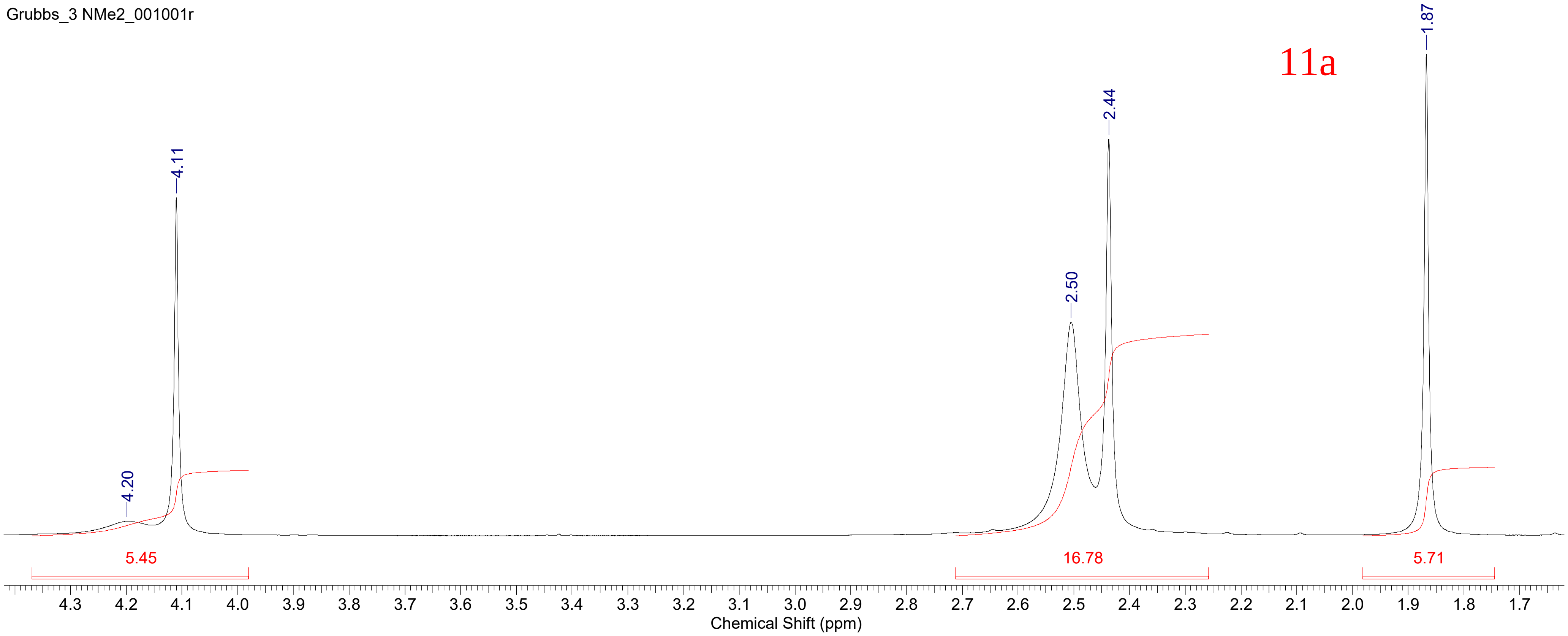

| Acquisition Time (sec) | 1.1010 | Comment | FZ_Grubbs_3-C13dc | Date | 12 Jan 2018 17:14:40 |
|---|---|---|---|---|---|
| Date Stamp | 12 Jan 2018 17:14:40 | | | | |
| File Name | C:\Users\Fedor\Desktop\Наброски Статей\Кирилл Статья по Катализаторам Граббса\ЯМР Граббс\ЯМР Граббс от Ромы\Grubbs_3 NMe2_013001r | | | | |
| Frequency (MHz) | 75.47 | Nucleus | 13C | Number of Transients | 1208 | Origin | spect |
| Original Points Count | 65536 | Owner | nmr | Points Count | 262144 | Pulse Sequence | zgpg |
| Receiver Gain | 202.48 | SW(cyclical) (Hz) | 59523.81 | Solvent | DICHLOROMETHANE-d2 | | |
| Spectrum Offset (Hz) | 7546.7783 | Sweep Width (Hz) | 59523.58 | Temperature (degree C) | 30.126 | | |

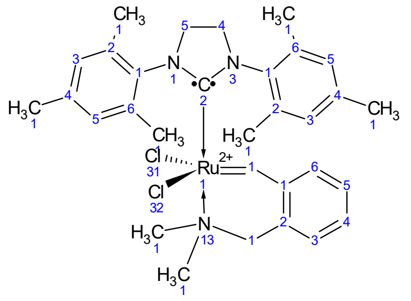


Grubbs_3 NMe2_013001r

11a

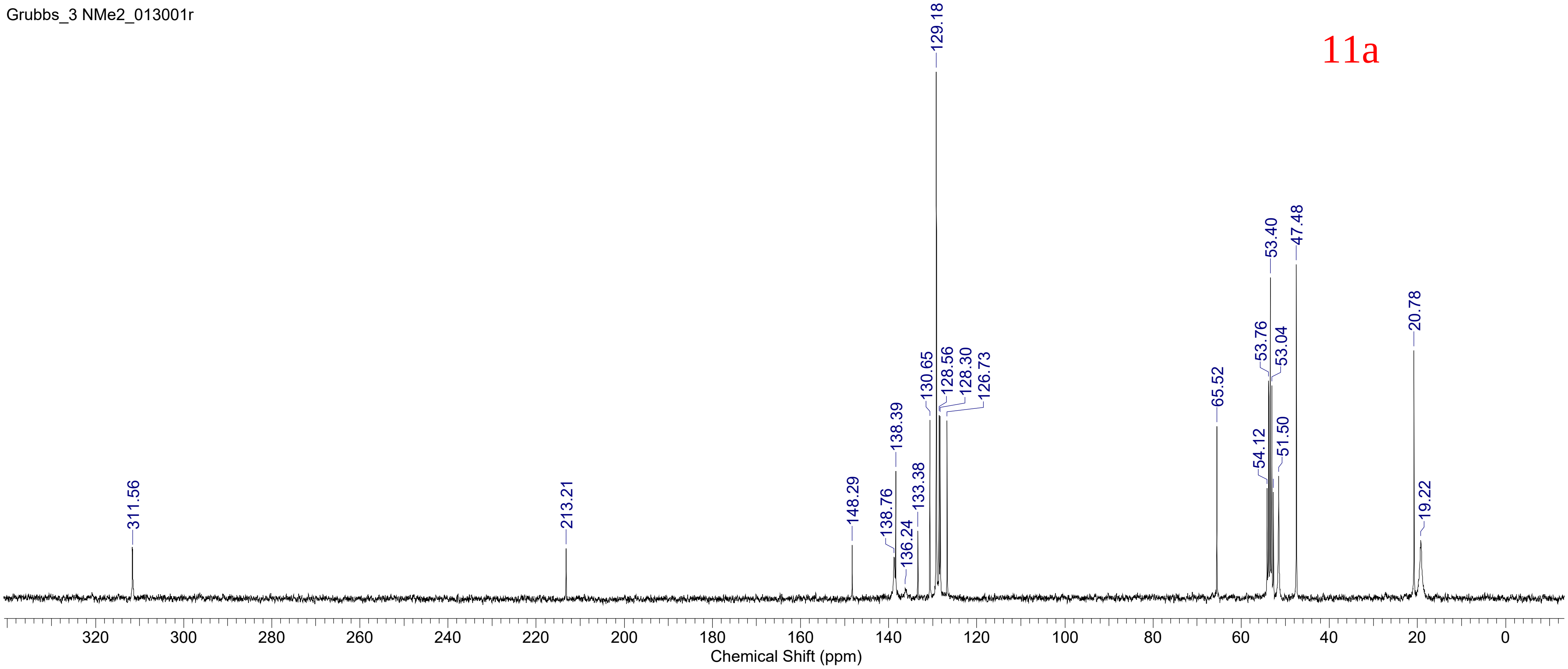

| | | | | | | | |
|---|---|---|---|---|---|---|---|
| ***Acquisition Time (sec)*** | 1.1010 | ***Comment*** | FZ_Grubbs_3-C13dc | | | ***Date*** | 12 Jan 2018 17:14:40 |
| ***Date Stamp*** | 12 Jan 2018 17:14:40 | | | | | | |
| ***File Name*** | C:\Users\Fedor\Desktop\Наброски Статей\Кирилл Статья по Катализаторам Граббса\ЯМР Граббс\ЯМР Граббс от Ромы\Grubbs_3 NMe2_013001r | | | | | | |
| ***Frequency (MHz)*** | 75.47 | ***Nucleus*** | 13C | ***Number of Transients*** | 1208 | ***Origin*** | spect |
| ***Original Points Count*** | 65536 | ***Owner*** | nmr | ***Points Count*** | 262144 | ***Pulse Sequence*** | zgpg |
| ***Receiver Gain*** | 202.48 | ***SW(cyclical) (Hz)*** | 59523.81 | ***Solvent*** | DICHLOROMETHANE-d2 | | |
| ***Spectrum Offset (Hz)*** | 7546.7783 | ***Sweep Width (Hz)*** | 59523.58 | ***Temperature (degree C)*** | 30.126 | | |

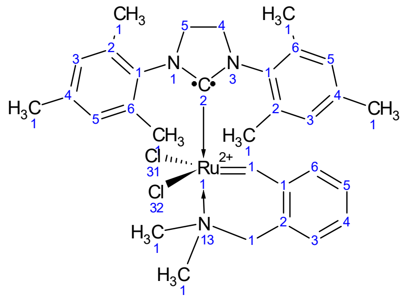

Grubbs_3 NMe2_013001r

11a

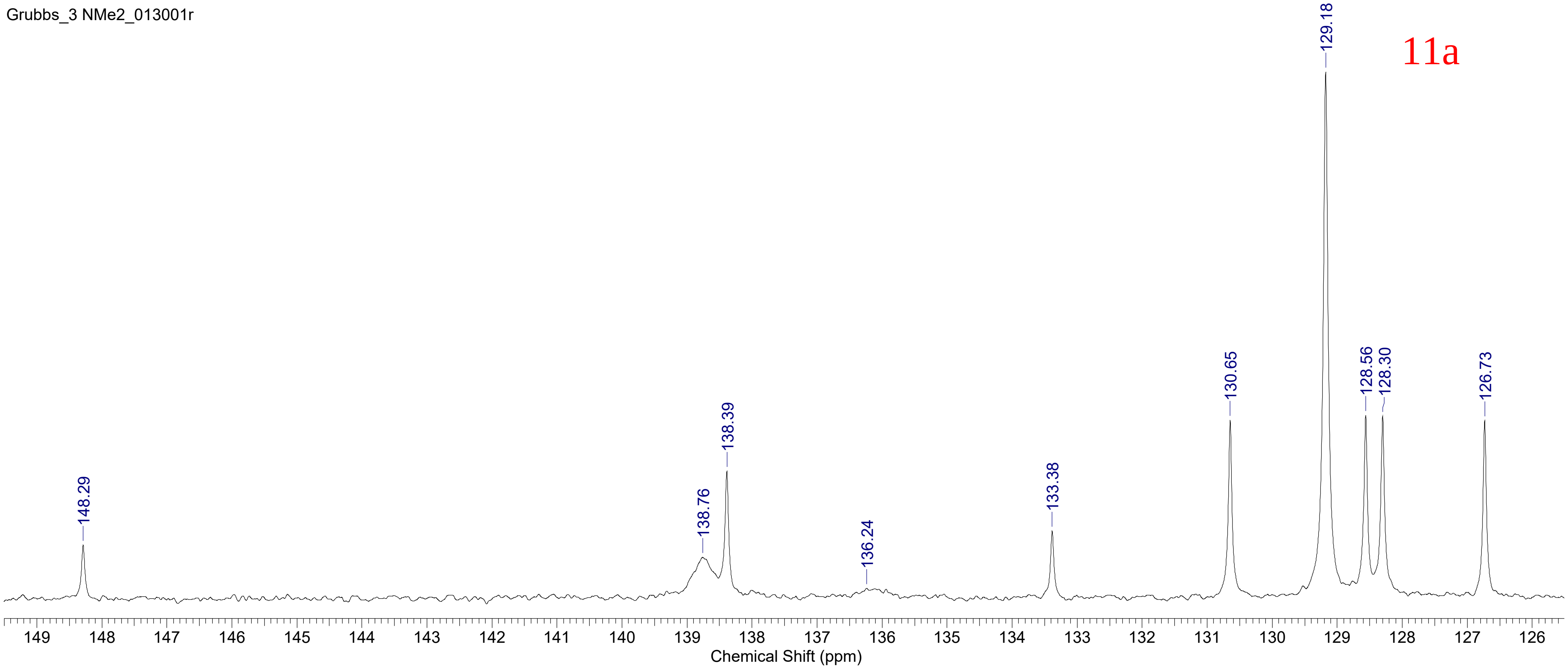

| | | | | | | | |
|---|---|---|---|---|---|---|---|
| ***Acquisition Time (sec)*** | 1.1010 | ***Comment*** | FZ_Grubbs_3-C13dc | | | ***Date*** | 12 Jan 2018 17:14:40 |
| ***Date Stamp*** | 12 Jan 2018 17:14:40 | | | | | | |
| ***File Name*** | C:\Users\Fedor\Desktop\Наброски Статей\Кирилл Статья по Катализаторам Граббса\ЯМР Граббс\ЯМР Граббс от Ромы\Grubbs_3 NMe2_013001r | | | | | | |
| ***Frequency (MHz)*** | 75.47 | ***Nucleus*** | 13C | ***Number of Transients*** | 1208 | ***Origin*** | spect |
| ***Original Points Count*** | 65536 | ***Owner*** | nmr | ***Points Count*** | 262144 | ***Pulse Sequence*** | zgpg |
| ***Receiver Gain*** | 202.48 | ***SW(cyclical) (Hz)*** | 59523.81 | ***Solvent*** | DICHLOROMETHANE-d2 | | |
| ***Spectrum Offset (Hz)*** | 7546.7783 | ***Sweep Width (Hz)*** | 59523.58 | ***Temperature (degree C)*** | 30.126 | | |

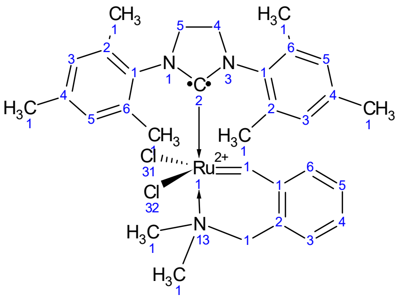

11a

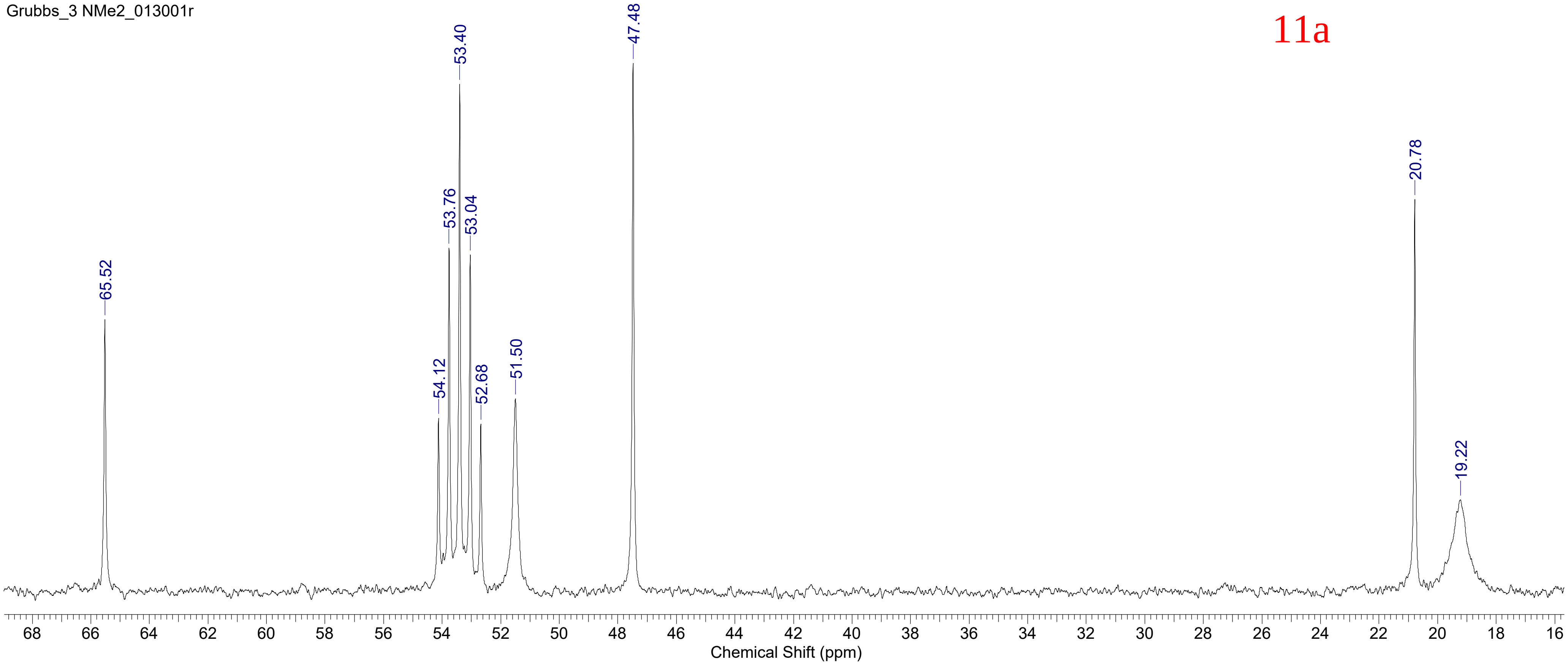

| | | | | | | | |
|---|---|---|---|---|---|---|---|
| ***Acquisition Time (sec)*** | 1.1010 | ***Comment*** | FZ_Grubbs_3-dept135 | | | ***Date*** | 12 Jan 2018 18:03:44 |
| ***Date Stamp*** | 12 Jan 2018 18:03:44 | | | | | | |
| ***File Name*** | C:\Users\Fedor\Desktop\Наброски Статей\Кирилл Статья по Катализаторам Граббса\ЯМР Граббс\ЯМР Граббс от Ромы\Grubbs_3 NMe2_015001r | | | | | | |
| ***Frequency (MHz)*** | 75.47 | ***Nucleus*** | 13C | ***Number of Transients*** | 800 | ***Origin*** | spect |
| ***Original Points Count*** | 65536 | ***Owner*** | nmr | ***Points Count*** | 262144 | ***Pulse Sequence*** | dept135 |
| ***Receiver Gain*** | 202.48 | ***SW(cyclical) (Hz)*** | 59523.81 | ***Solvent*** | DICHLOROMETHANE-d2 | | |
| ***Spectrum Offset (Hz)*** | 7546.7783 | ***Sweep Width (Hz)*** | 59523.58 | ***Temperature (degree C)*** | 29.964 | | |

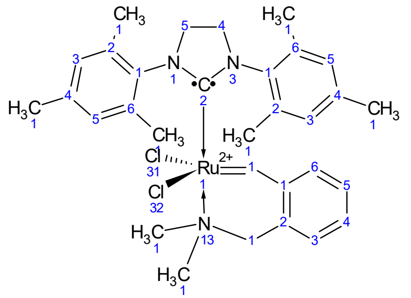

11a

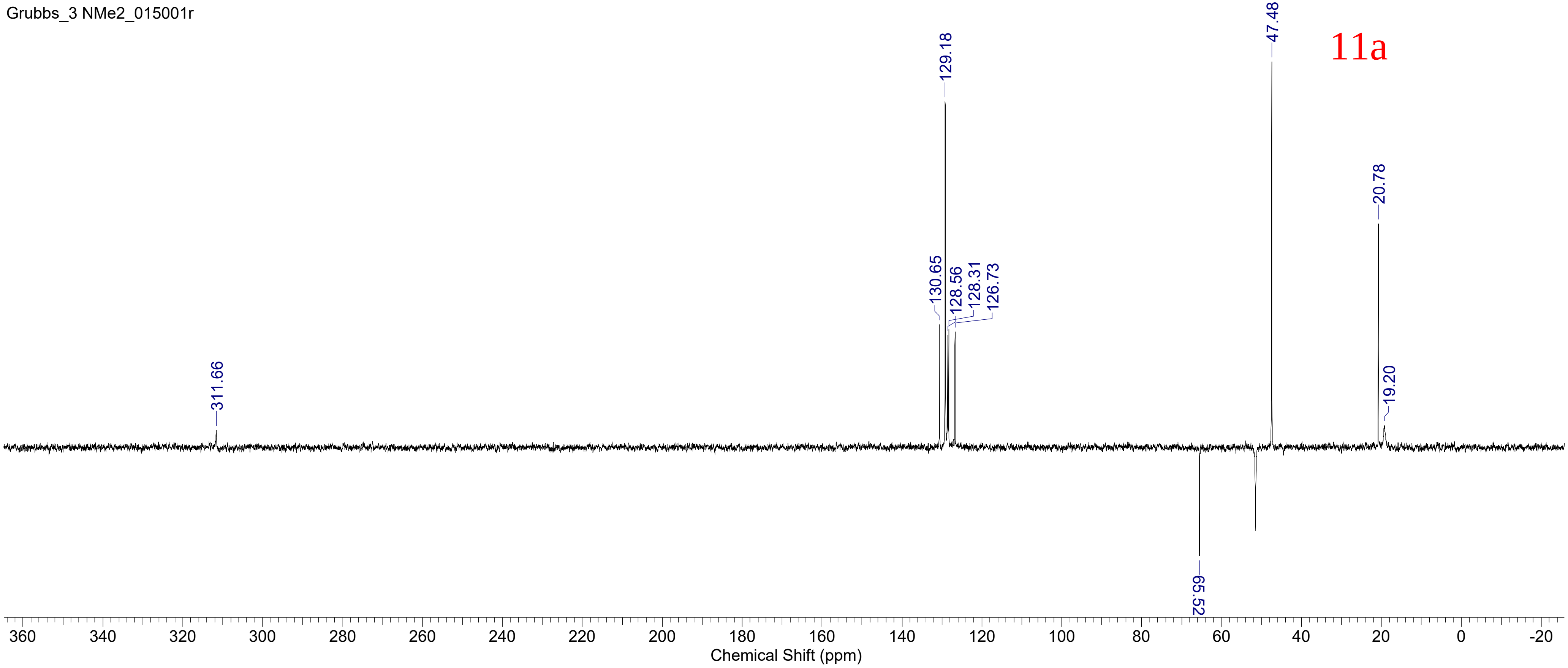

| | | | | | | | |
|---|---|---|---|---|---|---|---|
| **Acquisition Time (sec)** | 1.1010 | **Comment** | FZ_Grubbs_3-dept135 | | | **Date** | 12 Jan 2018 18:03:44 |
| **Date Stamp** | 12 Jan 2018 18:03:44 | | | | | | |
| **File Name** | C:\Users\Fedor\Desktop\Наброски Статей\Кирилл Статья по Катализаторам Граббса\ЯМР Граббс\ЯМР Граббс от Ромы\Grubbs_3 NMe2_015001r | | | | | | |
| **Frequency (MHz)** | 75.47 | **Nucleus** | 13C | **Number of Transients** | 800 | **Origin** | spect |
| **Original Points Count** | 65536 | **Owner** | nmr | **Points Count** | 262144 | **Pulse Sequence** | dept135 |
| **Receiver Gain** | 202.48 | **SW(cyclical) (Hz)** | 59523.81 | **Solvent** | DICHLOROMETHANE-d2 | | |
| **Spectrum Offset (Hz)** | 7546.7783 | **Sweep Width (Hz)** | 59523.58 | **Temperature (degree C)** | 29.964 | | |

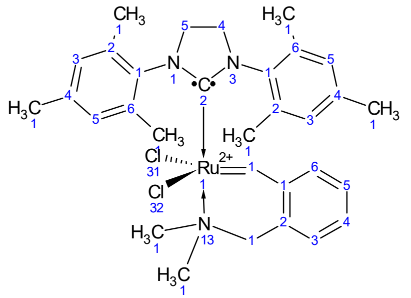


11a

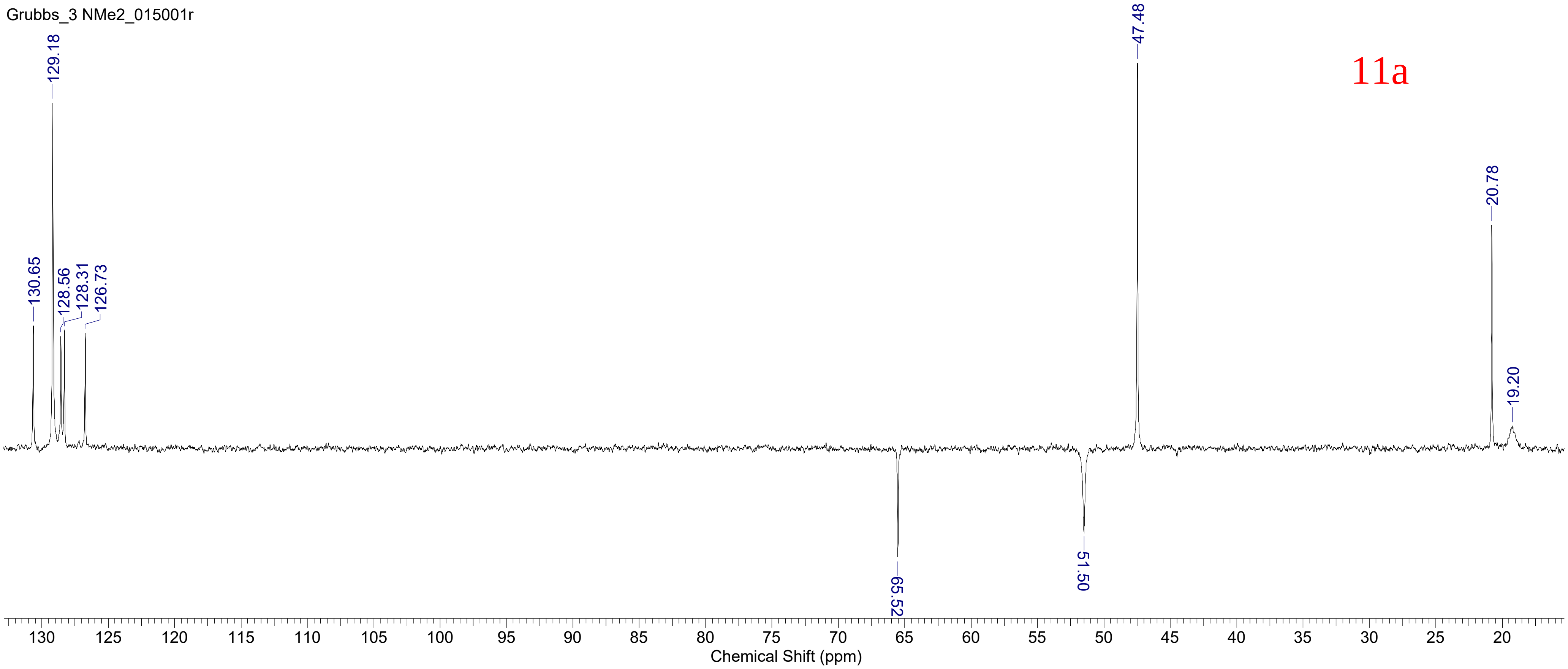

| | | | | | |
|---|---|---|---|---|---|
| **Acquisition Time (sec)** | (0.3408, 0.0333) | **Comment** | 5 mm PABBO BB-1H/D Z-GRD Z104275/0345 | | |
| **Date** | 12 Jan 2018 22:02:36 | | | | |
| **File Name** | C:\Users\Fedor\Desktop\Наброски Статей\Кирилл Статья по Катализаторам Граббса\ЯМР Граббс\ЯМР Граббс от Ромы\Grubbs_3 NMe2\11\pdata\1\2rr | | | | |
| **Frequency (MHz)** | (300.13, 300.13) | **Nucleus** | (1H, 1H) | **Number of Transients** | 1 |
| **Origin** | spect | **Original Points Count** | (3072, 300) | **Owner** | nmr |
| **Points Count** | (8192, 2048) | **Pulse Sequence** | cosygpqf | **Solvent** | CD2Cl2 |
| **Sweep Width (Hz)** | (9014.42, 9009.01) | **Temperature (degree C)** | 29.918 | **Title** | FZ_Grubbs_3-COSY |

| | | | |
|---|---|---|---|
| **Formula** | $C_{31}H_{39}Cl_2N_3Ru$ | **FW** | 625.6364 |

Grubbs_3 NMe2.011.001.2rr.esp

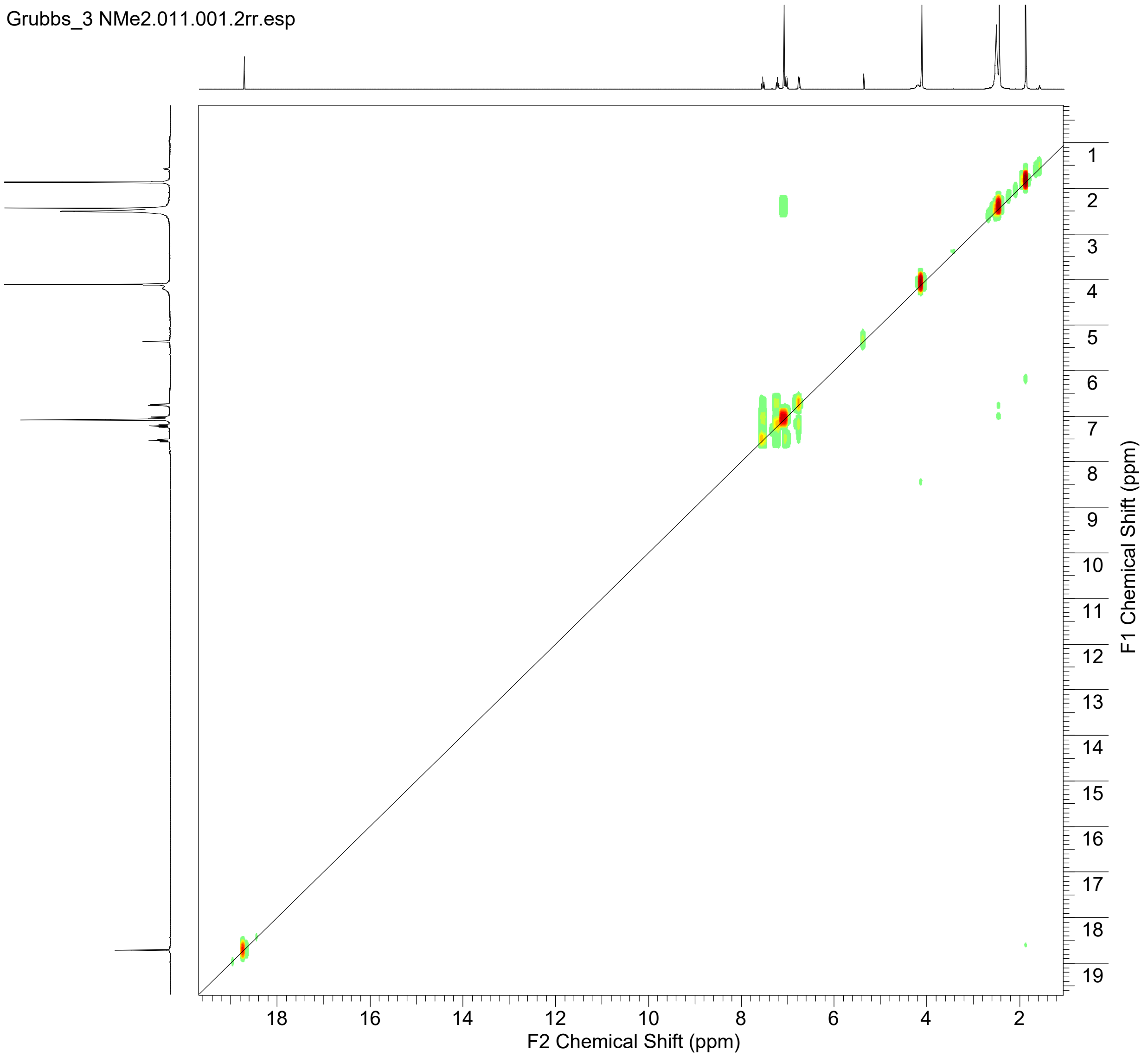


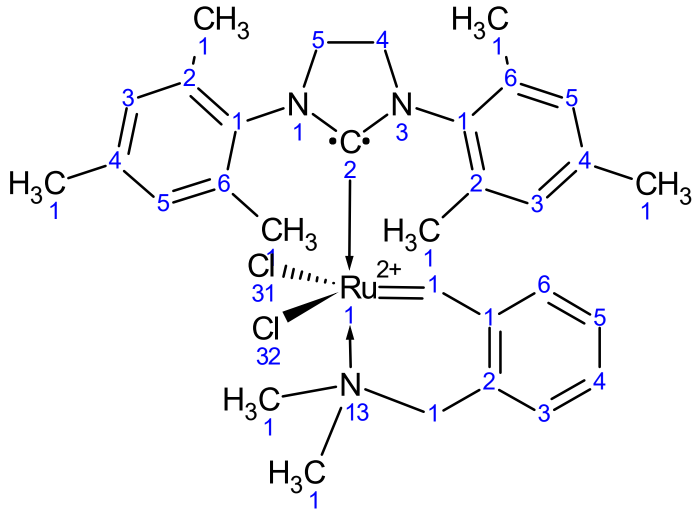

11a

| | | | | | |
|---|---|---|---|---|---|
| ***Acquisition Time (sec)*** | (0.3408, 0.0333) | ***Comment*** | 5 mm PABBO BB-1H/D Z-GRD Z104275/0345 | | |
| ***Date*** | 12 Jan 2018 22:02:36 | | | | |
| ***File Name*** | C:\Users\Fedor\Desktop\Наброски Статей\Кирилл Статья по Катализаторам Граббса\ЯМР Граббс\ЯМР Граббс от Ромы\Grubbs_3 NMe2\11\pdata\1\2rr | | | | |
| ***Frequency (MHz)*** | (300.13, 300.13) | ***Nucleus*** | (1H, 1H) | ***Number of Transients*** | 1 |
| ***Origin*** | spect | ***Original Points Count*** | (3072, 300) | ***Owner*** | nmr |
| ***Points Count*** | (8192, 2048) | ***Pulse Sequence*** | cosygpqf | ***Solvent*** | CD2Cl2 |
| ***Sweep Width (Hz)*** | (9014.42, 9009.01) | ***Temperature (degree C)*** | 29.918 | ***Title*** | FZ_Grubbs_3-COSY |

| | |
|---|---|
| ***Formula*** $C_{31}H_{39}Cl_2N_3Ru$ | ***FW*** 625.6364 |

Grubbs_3 NMe2.011.001.2rr.esp

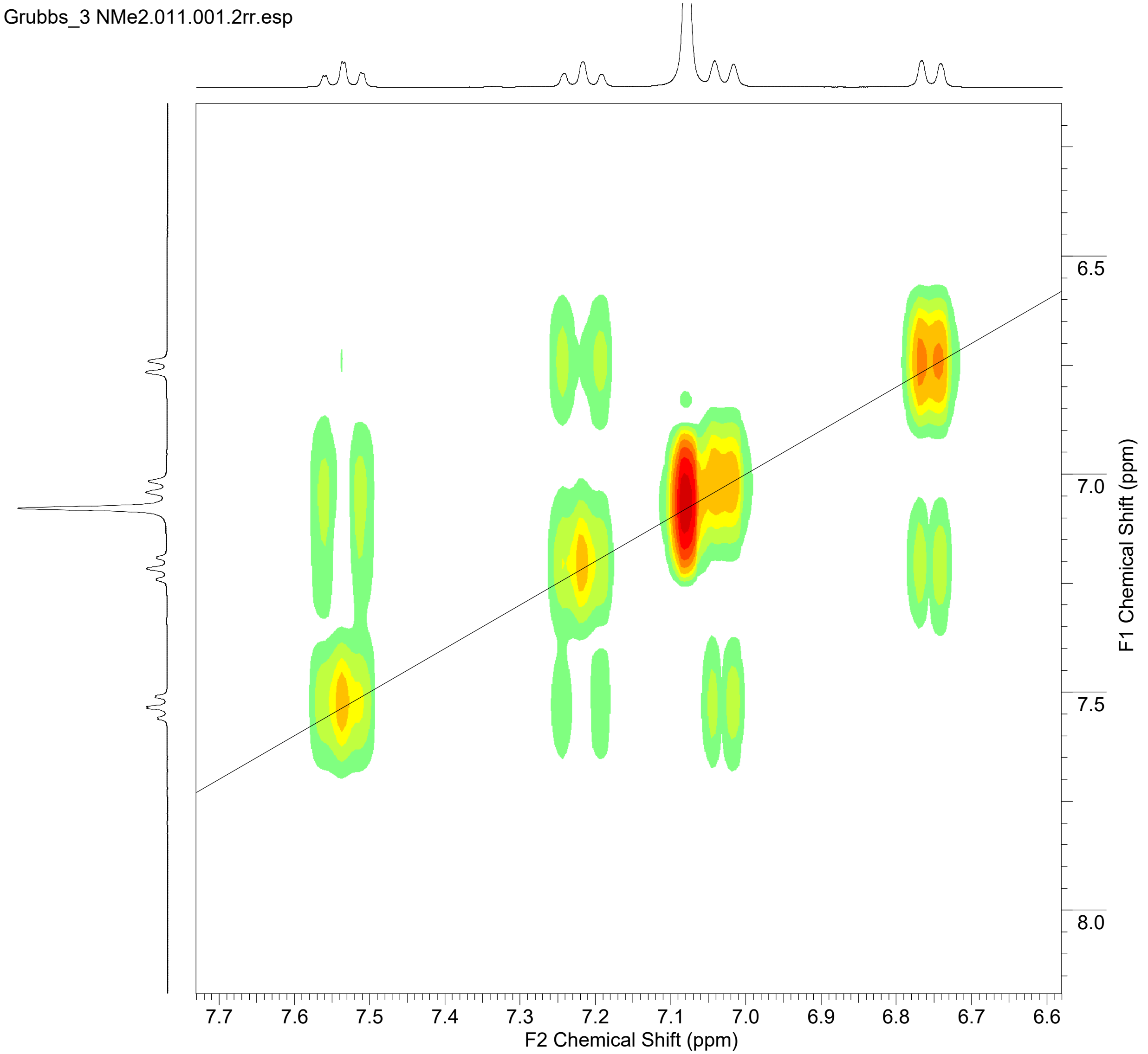


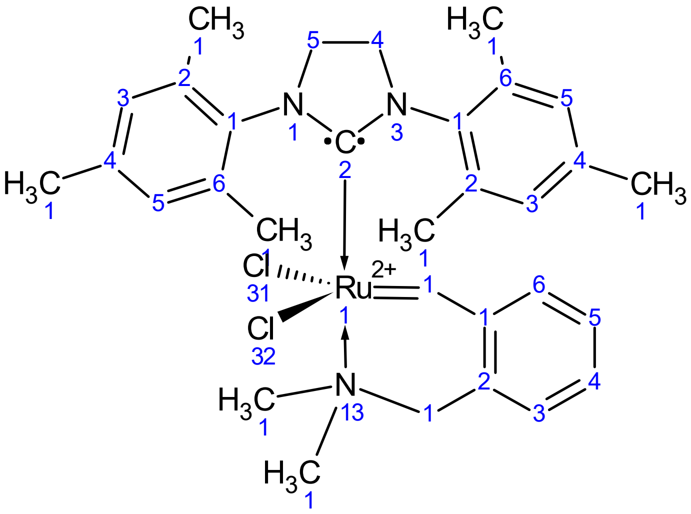


11a

| | | | | | |
|---|---|---|---|---|---|
| ***Acquisition Time (sec)*** | (0.3408, 0.0333) | ***Comment*** | 5 mm PABBO BB-1H/D Z-GRD Z104275/0345 | | |
| ***Date*** | 12 Jan 2018 22:02:36 | | | | |
| ***File Name*** | C:\Users\Fedor\Desktop\Наброски Статей\Кирилл Статья по Катализаторам Граббса\ЯМР Граббс\ЯМР Граббс от Ромы\Grubbs_3 NMe2\11\pdata\1\2rr | | | | |
| ***Frequency (MHz)*** | (300.13, 300.13) | ***Nucleus*** | (1H, 1H) | ***Number of Transients*** | 1 |
| ***Origin*** | spect | ***Original Points Count*** | (3072, 300) | ***Owner*** | nmr |
| ***Points Count*** | (8192, 2048) | ***Pulse Sequence*** | cosygpqf | ***Solvent*** | CD2Cl2 |
| ***Sweep Width (Hz)*** | (9014.42, 9009.01) | ***Temperature (degree C)*** | 29.918 | ***Title*** | FZ_Grubbs_3-COSY |

| | |
|---|---|
| ***Formula*** $C_{31}H_{39}Cl_2N_3Ru$ | ***FW*** 625.6364 |

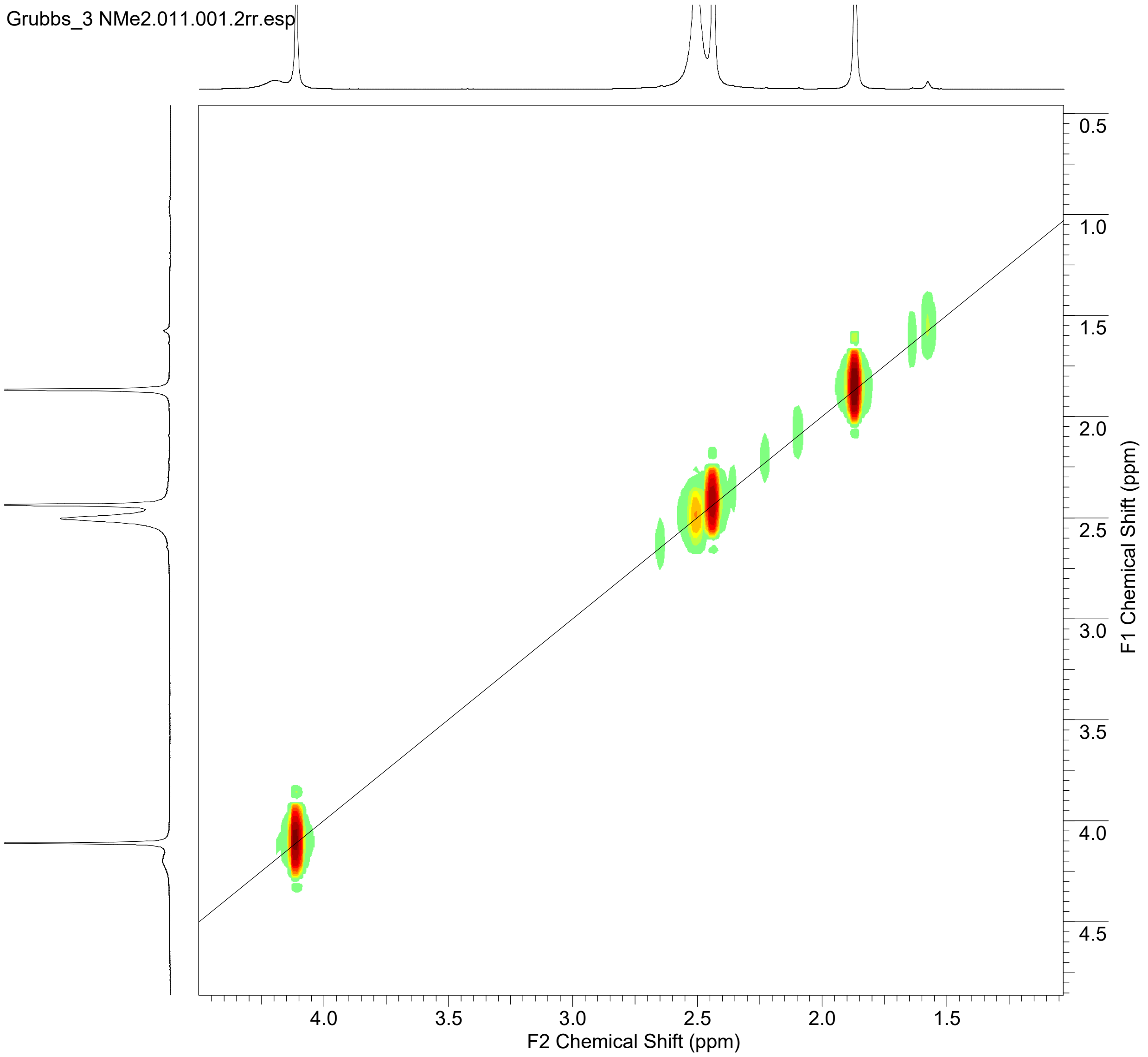


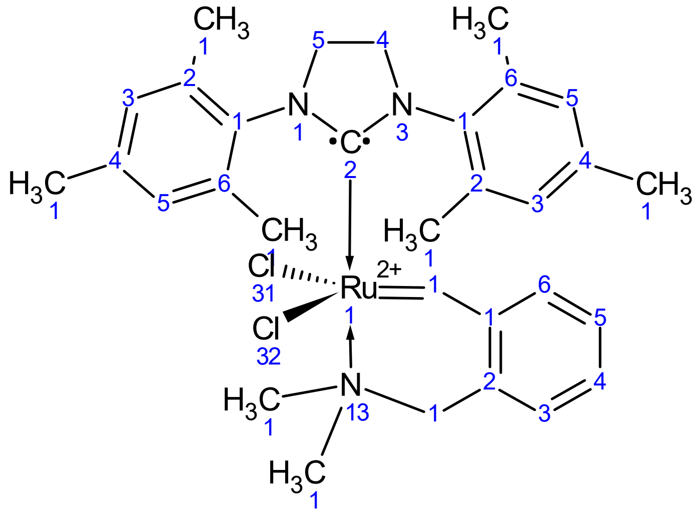

11a

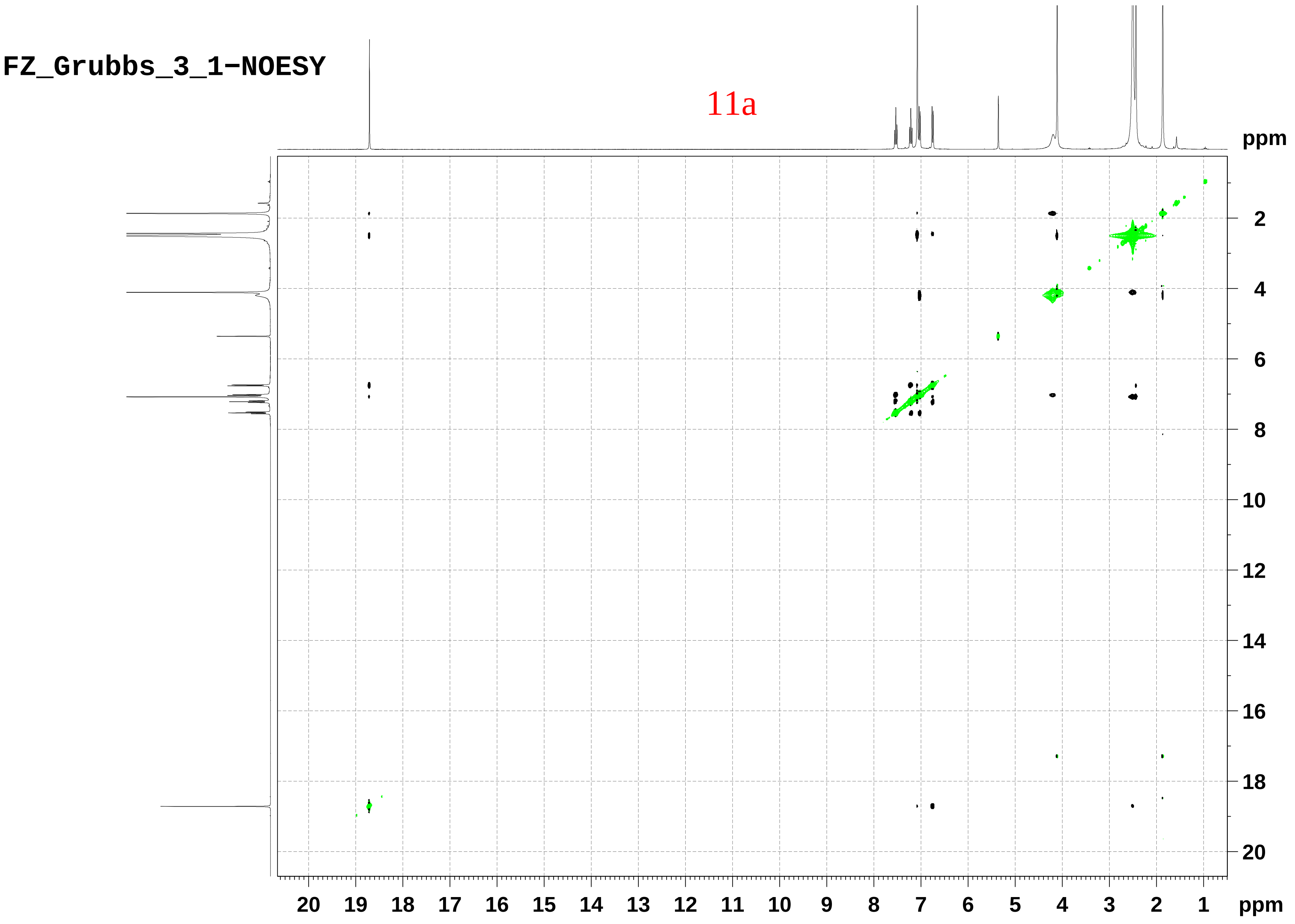

FZ_Grubbs_3_1-NOESY
11a
ppm
2
4
6
8
10
12
14
16
18
20
20 19 18 17 16 15 14 13 12 11 10 9 8 7 6 5 4 3 2 1
ppm

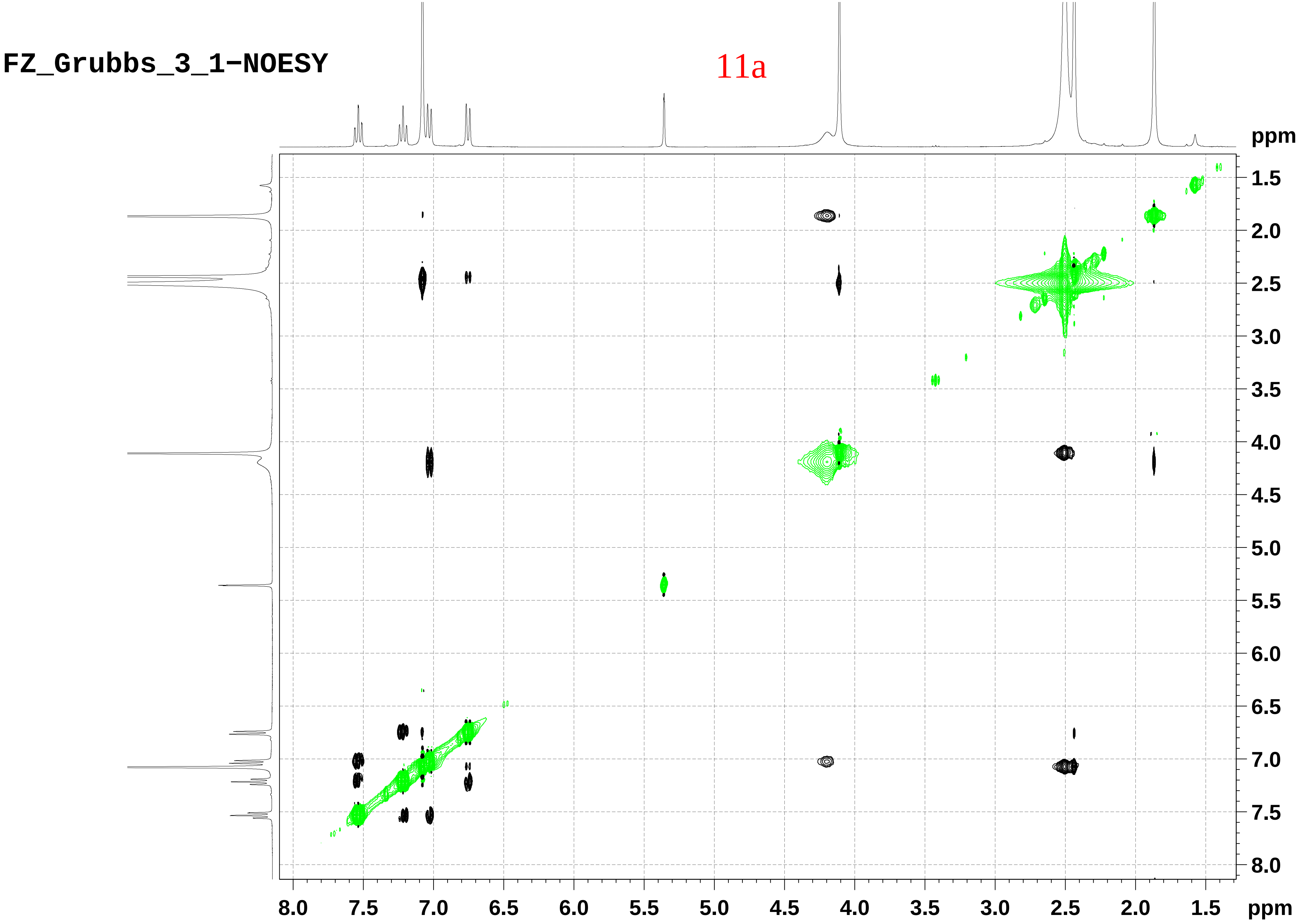

FZ_Grubbs_3_1-NOESY
11a
ppm
1.5
2.0
2.5
3.0
3.5
4.0
4.5
5.0
5.5
6.0
6.5
7.0
7.5
8.0
8.0
7.5
7.0
6.5
6.0
5.5
5.0
4.5
4.0
3.5
3.0
2.5
2.0
1.5
ppm

FZ_Grubbs_3_1-NOESY

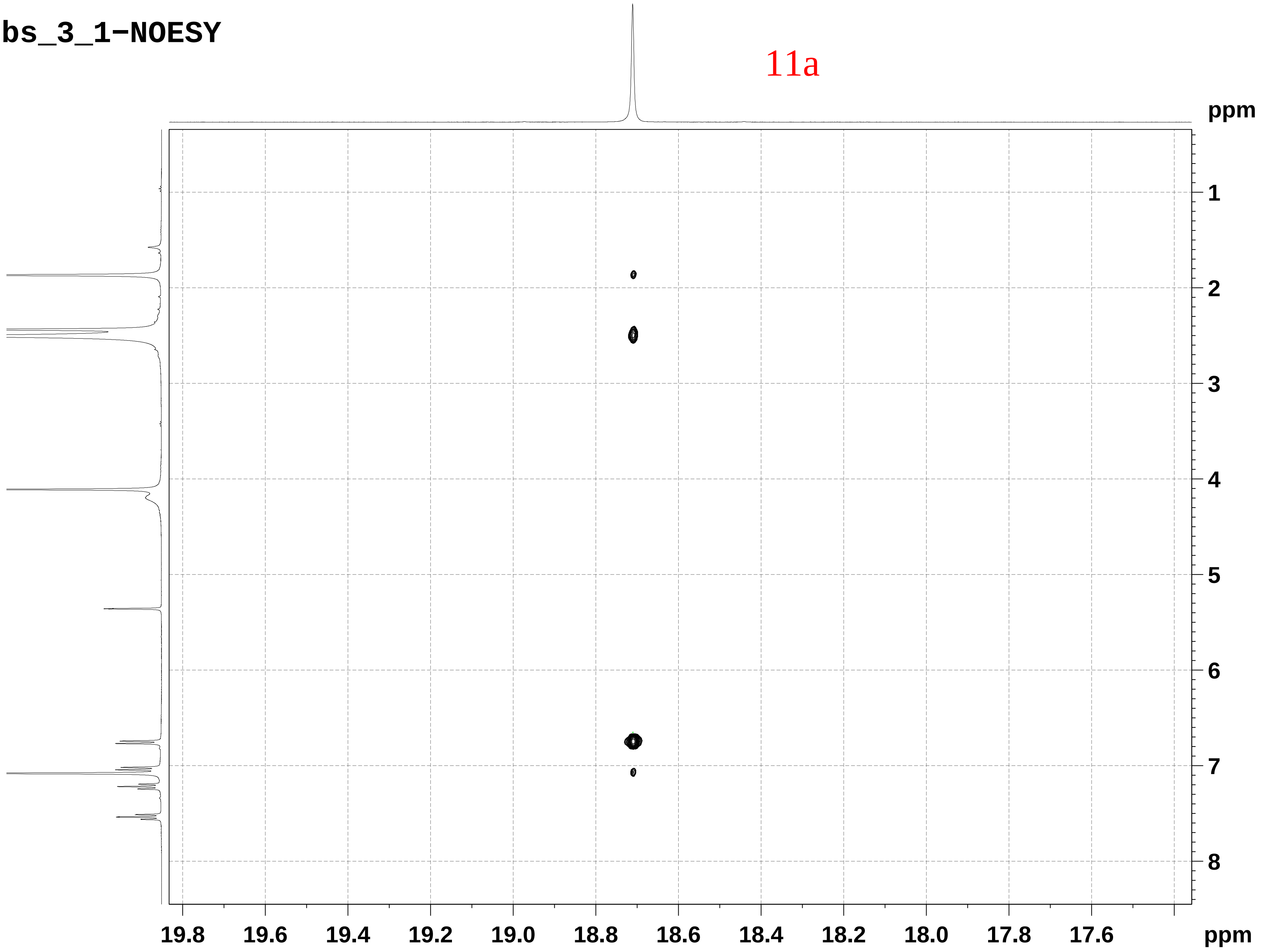

| Acquisition Time (sec) | (0.3408, 0.0037) | Comment | 5 mm PABBO BB-1H/D Z-GRD Z104275/0345 | | |
|---|---|---|---|---|---|
| Date | 12 Jan 2018 21:44:54 | | | | |
| File Name | C:\Users\Fedor\Desktop\Наброски статей\Кирлл Статья по Катализаторам Граббса\ЯМР Граббс\ЯМР Граббс от Ромы\FZ_Grubbs_3\114\pdata\1\2rr | | | | |
| Frequency (MHz) | (300.13, 75.48) | Nucleus | (1H, 13C) | Number of Transients | 2 |
| Origin | spect | Original Points Count | (3072, 127) | Owner | nmr |
| Points Count | (8192, 2048) | Pulse Sequence | hsqcedetgp | Solvent | CD2Cl2 |
| Sweep Width (Hz) | (9014.42, 34013.61) | Temperature (degree C) | 30.056 | Title | FZ_Grubbs_3-edited-HSQC [proc = EM] |

| Formula | $C_{31}H_{39}Cl_2N_3Ru$ | FW | 625.6364 |
|---|---|---|---|

FZ_Grubbs_3.114.001.2rr.esp

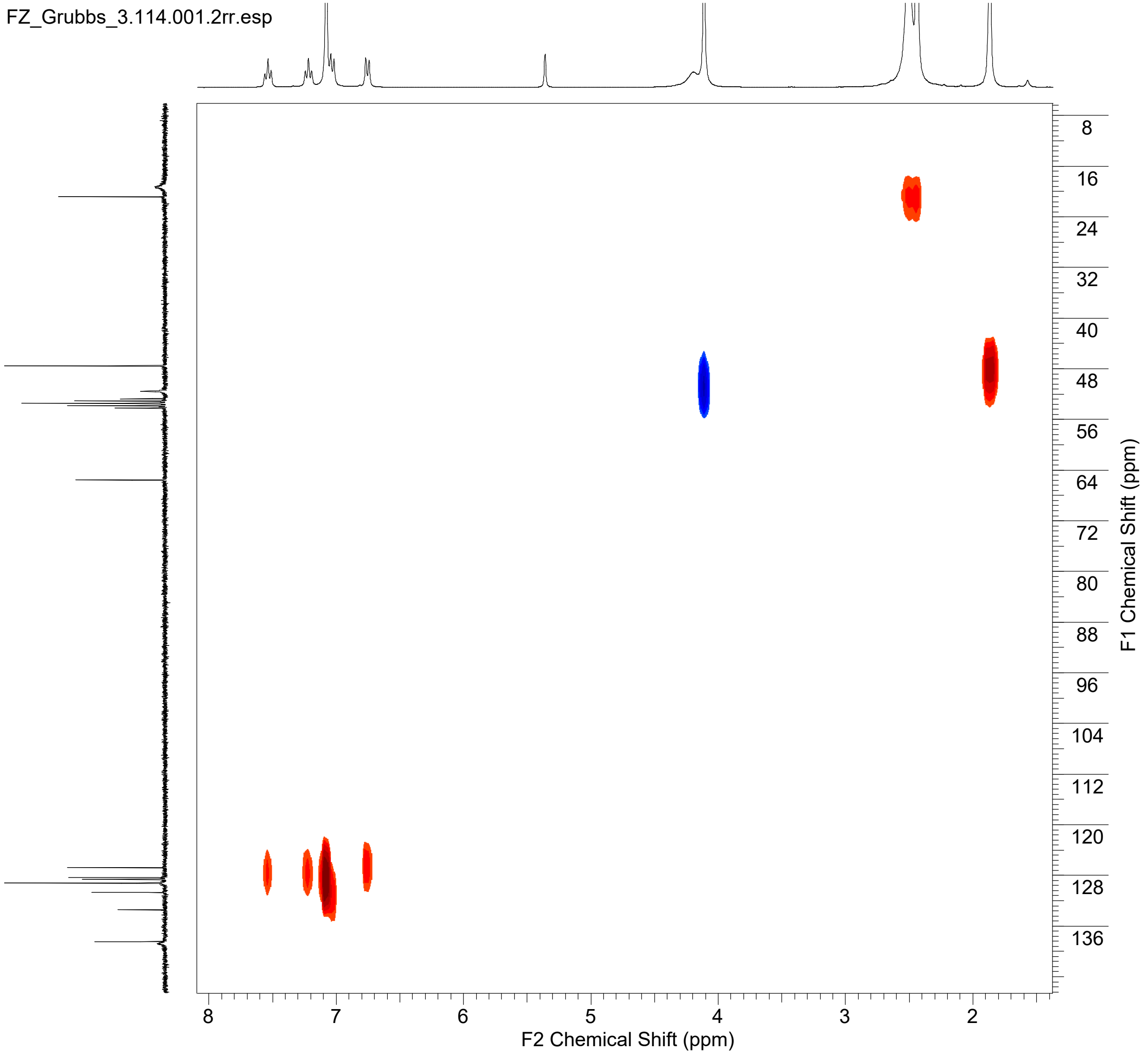


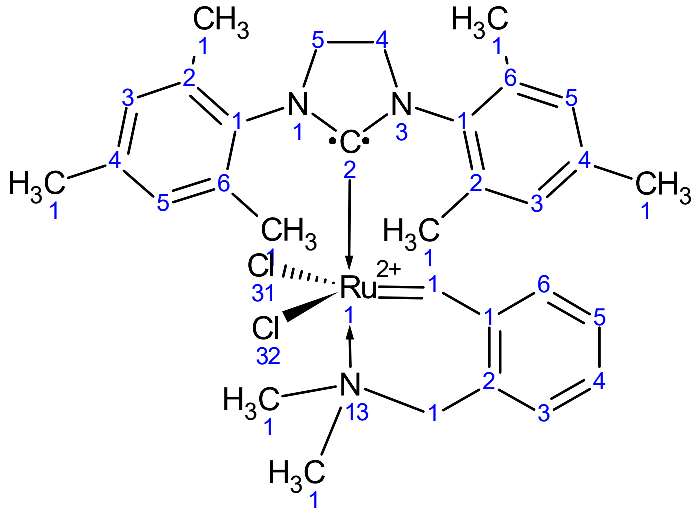

11a

| | | | | | |
|---|---|---|---|---|---|
| ***Acquisition Time (sec)*** | (0.3408, 0.0037) | ***Comment*** | 5 mm PABBO BB-1H/D Z-GRD Z104275/0345 | | |
| ***Date*** | 12 Jan 2018 21:44:54 | | | | |
| ***File Name*** | C:\Users\Fedor\Desktop\Наброски статей\Кирлл Статья по Катализаторам Граббса\ЯМР Граббс\ЯМР Граббс от Ромы\FZ_Grubbs_3\114\pdata\1\2rr | | | | |
| ***Frequency (MHz)*** | (300.13, 75.48) | ***Nucleus*** | (1H, 13C) | ***Number of Transients*** | 2 |
| ***Origin*** | spect | ***Original Points Count*** | (3072, 127) | ***Owner*** | nmr |
| ***Points Count*** | (8192, 2048) | ***Pulse Sequence*** | hsqcedetgp | ***Solvent*** | CD2Cl2 |
| ***Sweep Width (Hz)*** | (9014.42, 34013.61) | ***Temperature (degree C)*** | 30.056 | ***Title*** | FZ_Grubbs_3-edited-HSQC [proc = EM] |

| | | | |
|---|---|---|---|
| ***Formula*** | $C_{31}H_{39}Cl_2N_3Ru$ | ***FW*** | 625.6364 |

FZ_Grubbs_3.114.001.2rr.esp

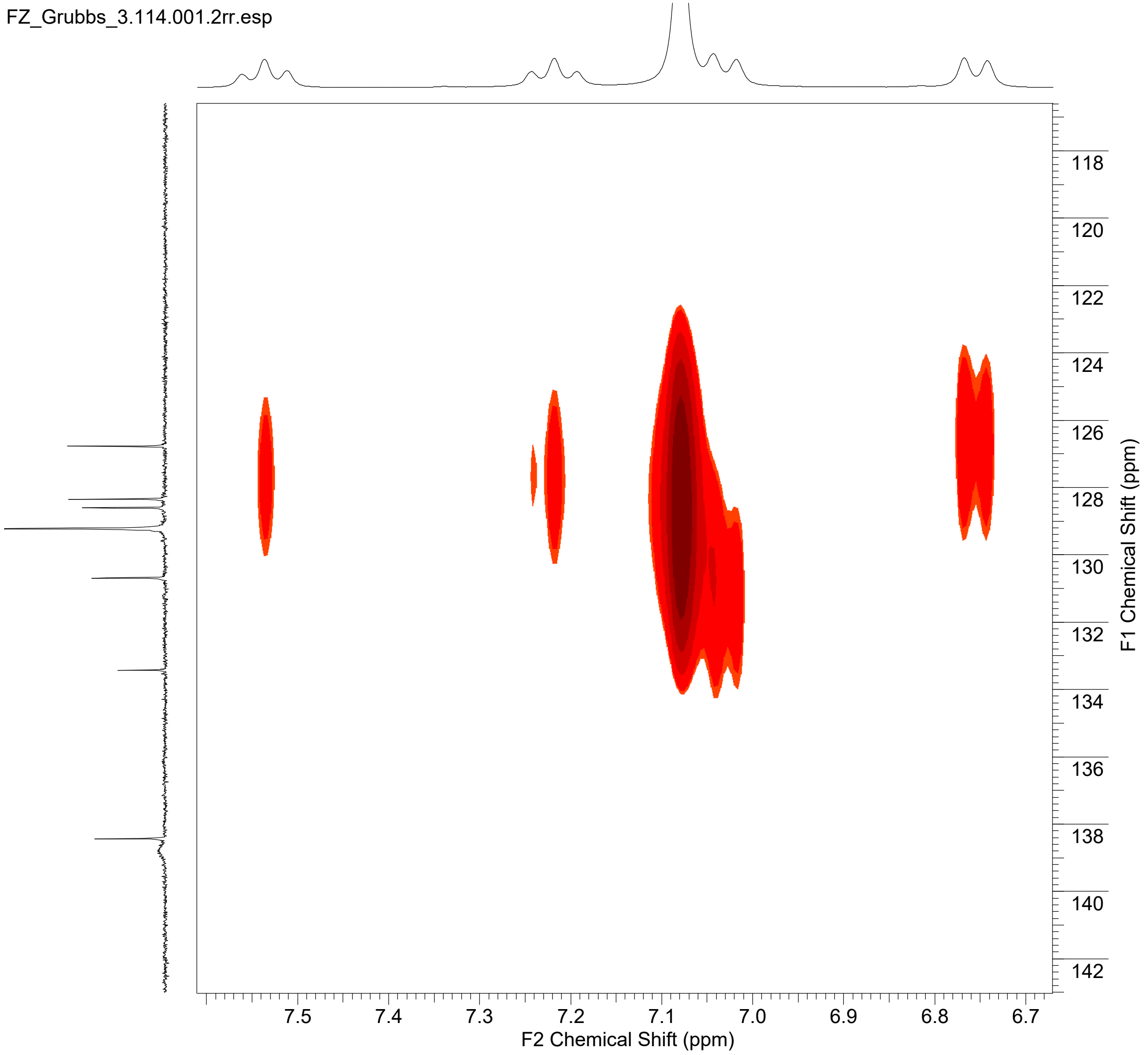


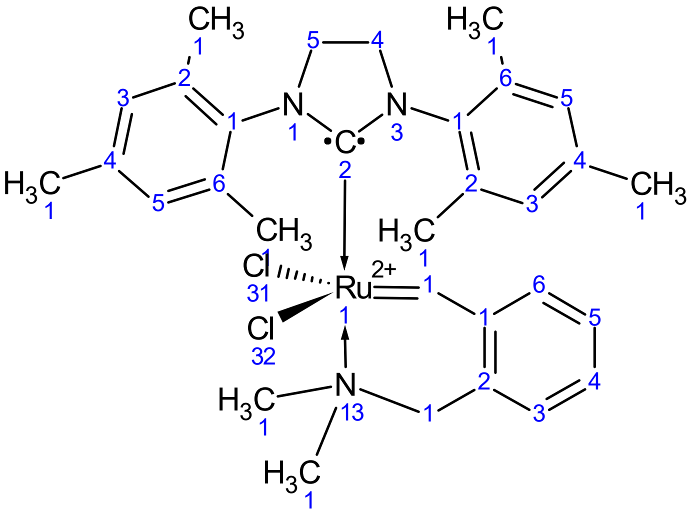

11a

| | | | | | |
|---|---|---|---|---|---|
| ***Acquisition Time (sec)*** | (0.3408, 0.0037) | ***Comment*** | 5 mm PABBO BB-1H/D Z-GRD Z104275/0345 | | |
| ***Date*** | 12 Jan 2018 21:44:54 | | | | |
| ***File Name*** | C:\Users\Fedor\Desktop\Наброски статей\Кирлл Статья по Катализаторам Граббса\ЯМР Граббс\ЯМР Граббс от Ромы\FZ_Grubbs_3\114\pdata\1\2rr | | | | |
| ***Frequency (MHz)*** | (300.13, 75.48) | ***Nucleus*** | (1H, 13C) | ***Number of Transients*** | 2 |
| ***Origin*** | spect | ***Original Points Count*** | (3072, 127) | ***Owner*** | nmr |
| ***Points Count*** | (8192, 2048) | ***Pulse Sequence*** | hsqcedetgp | ***Solvent*** | CD2Cl2 |
| ***Sweep Width (Hz)*** | (9014.42, 34013.61) | ***Temperature (degree C)*** | 30.056 | ***Title*** | FZ_Grubbs_3-edited-HSQC [proc = EM] |

| | | | |
|---|---|---|---|
| ***Formula*** | $C_{31}H_{39}Cl_2N_3Ru$ | ***FW*** | 625.6364 |

FZ_Grubbs_3.114.001.2rr.esp

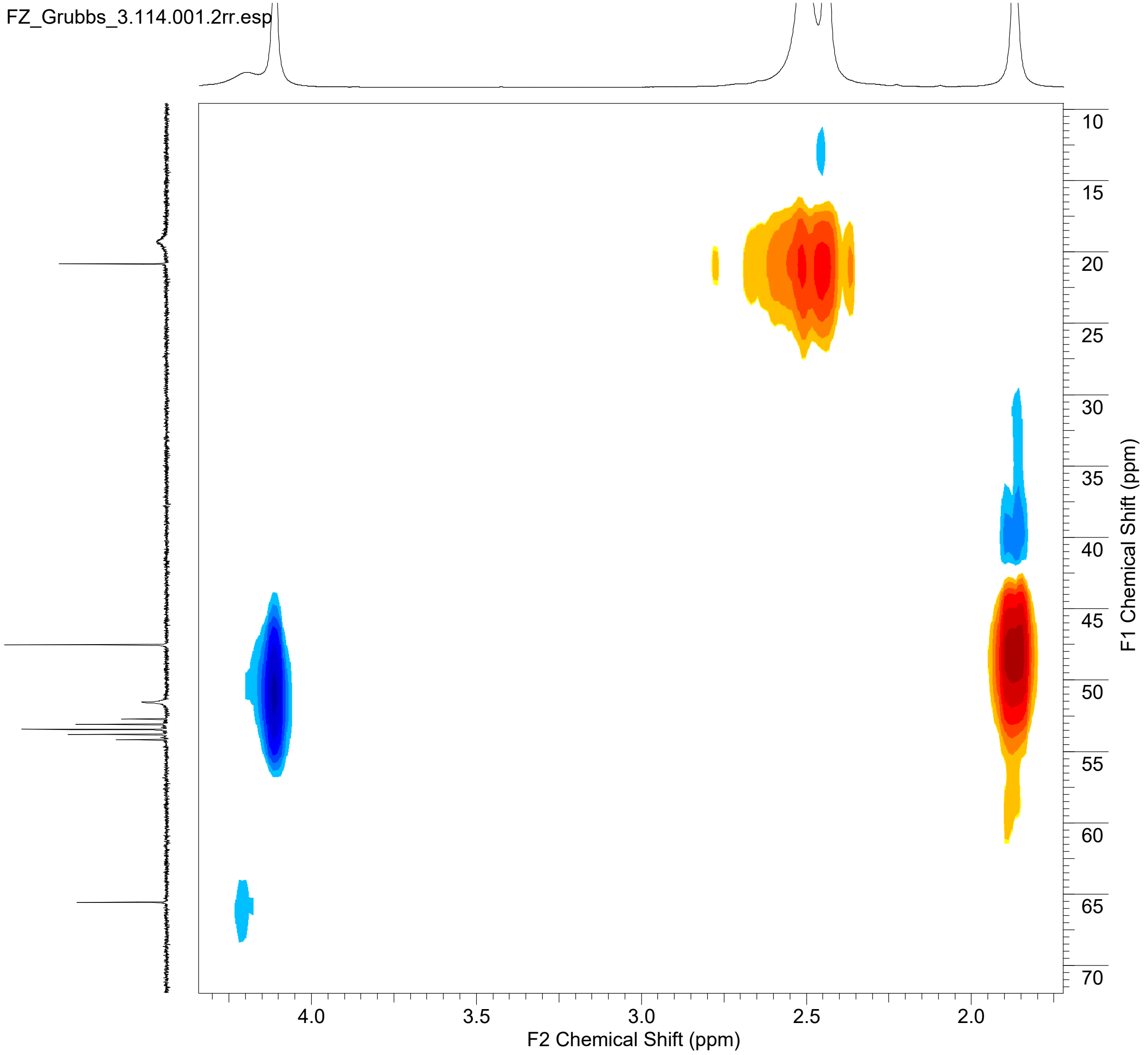


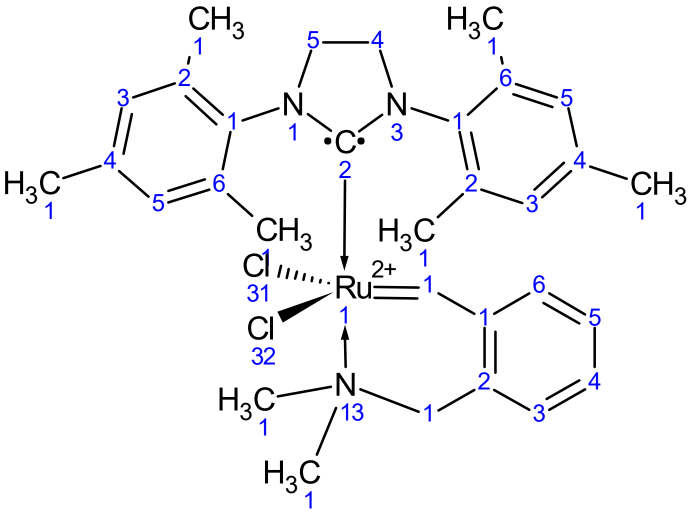

11a

| | | | | | |
|---|---|---|---|---|---|
| ***Acquisition Time (sec)*** | (0.3408, 0.0173) | ***Comment*** | 5 mm PABBO BB-1H/D Z-GRD Z104275/0345 | | |
| ***Date*** | 17 Jan 2018 21:32:24 | | | | |
| ***File Name*** | C:\Users\Fedor\Desktop\Наброски Статей\Кирилл Статья по Катализаторам Граббса\ЯМР Граббс\ЯМР Граббс от Ромы\Grubbs_3 NMe2\213\pdata\1\2rr | | | | |
| ***Frequency (MHz)*** | (300.13, 75.48) | ***Nucleus*** | (1H, 13C) | ***Number of Transients*** | 6 |
| ***Origin*** | spect | ***Original Points Count*** | (3072, 590) | ***Owner*** | nmr |
| ***Points Count*** | (8192, 2048) | ***Pulse Sequence*** | hmbcgplpndqf | ***Solvent*** | CD2Cl2 |
| ***Sweep Width (Hz)*** | (9014.42, 34013.61) | ***Temperature (degree C)*** | 30.007 | ***Title*** | FZ_Grubbs_3_1-HMBC |

| | | | |
|---|---|---|---|
| ***Formula*** | $C_{31}H_{39}Cl_2N_3Ru$ | ***FW*** | 625.6364 |

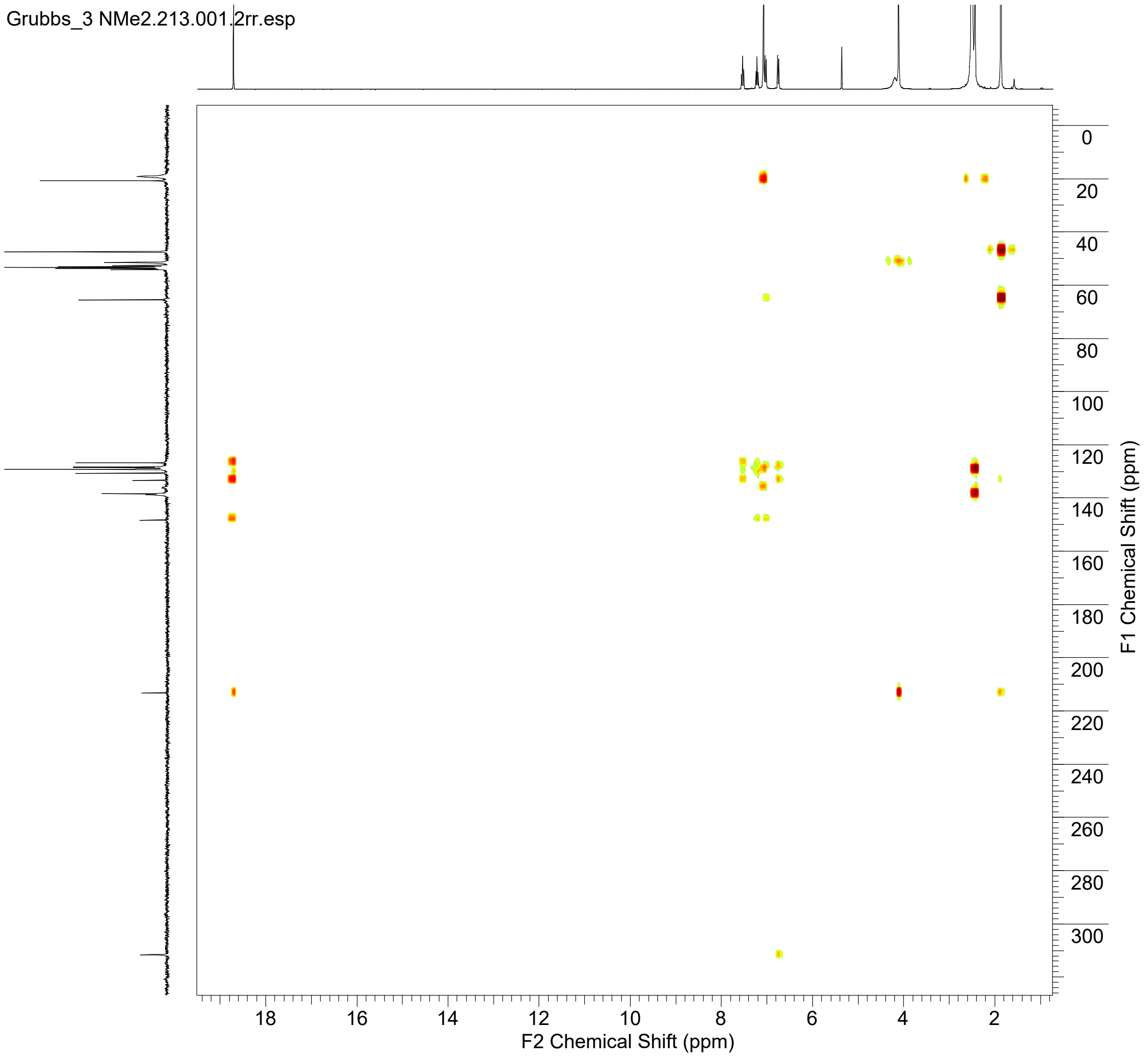


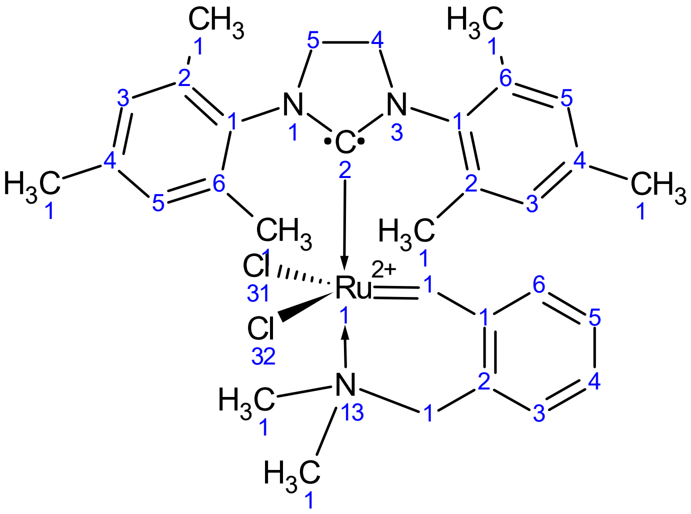

11a

| | | | | | |
|---|---|---|---|---|---|
| ***Acquisition Time (sec)*** | (0.3408, 0.0173) | ***Comment*** | 5 mm PABBO BB-1H/D Z-GRD Z104275/0345 | | |
| ***Date*** | 17 Jan 2018 21:32:24 | | | | |
| ***File Name*** | C:\Users\Fedor\Desktop\Наброски Статей\Кирилл Статья по Катализаторам Граббса\ЯМР Граббс\ЯМР Граббс от Ромы\Grubbs_3 NMe2\213\pdata\1\2rr | | | | |
| ***Frequency (MHz)*** | (300.13, 75.48) | ***Nucleus*** | (1H, 13C) | ***Number of Transients*** | 6 |
| ***Origin*** | spect | ***Original Points Count*** | (3072, 590) | ***Owner*** | nmr |
| ***Points Count*** | (8192, 2048) | ***Pulse Sequence*** | hmbcgplpndqf | ***Solvent*** | CD2Cl2 |
| ***Sweep Width (Hz)*** | (9014.42, 34013.61) | ***Temperature (degree C)*** | 30.007 | ***Title*** | FZ_Grubbs_3_1-HMBC |

| | | | |
|---|---|---|---|
| ***Formula*** | $C_{31}H_{39}Cl_2N_3Ru$ | ***FW*** | 625.6364 |

Grubbs_3 NMe2.213.001.2rr.esp

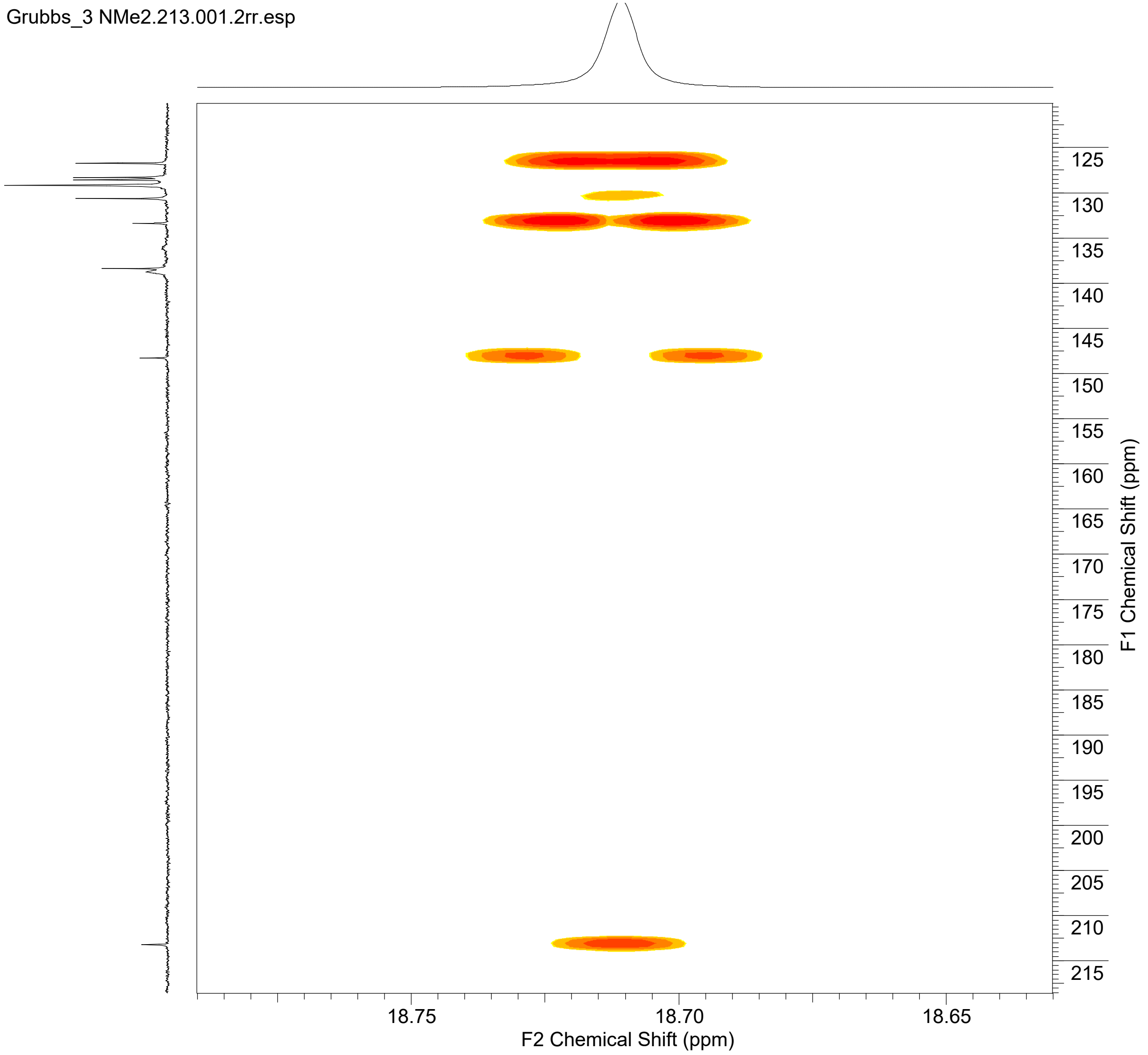


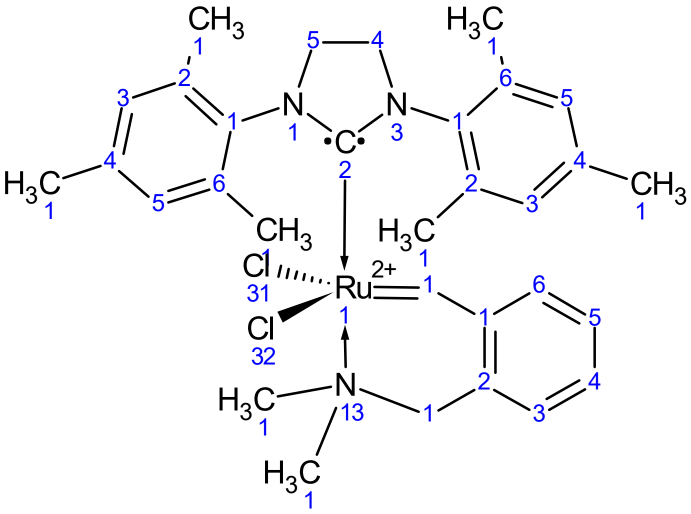

11a

| Acquisition Time (sec) | (0.3408, 0.0173) | Comment | 5 mm PABBO BB-1H/D Z-GRD Z104275/0345 | | |
|---|---|---|---|---|---|
| Date | 17 Jan 2018 21:32:24 | | | | |
| File Name | C:\Users\Fedor\Desktop\Наброски Статей\Кирилл Статья по Катализаторам Граббса\ЯМР Граббс\ЯМР Граббс от Ромы\Grubbs_3 NMe2\213\pdata\1\2rr | | | | |
| Frequency (MHz) | (300.13, 75.48) | Nucleus | (1H, 13C) | Number of Transients | 6 |
| Origin | spect | Original Points Count | (3072, 590) | Owner | nmr |
| Points Count | (8192, 2048) | Pulse Sequence | hmbcgplpndqf | Solvent | CD2Cl2 |
| Sweep Width (Hz) | (9014.42, 34013.61) | Temperature (degree C) | 30.007 | Title | FZ_Grubbs_3_1-HMBC |

| Formula | $C_{31}H_{39}Cl_2N_3Ru$ | FW | 625.6364 |
|---|---|---|---|

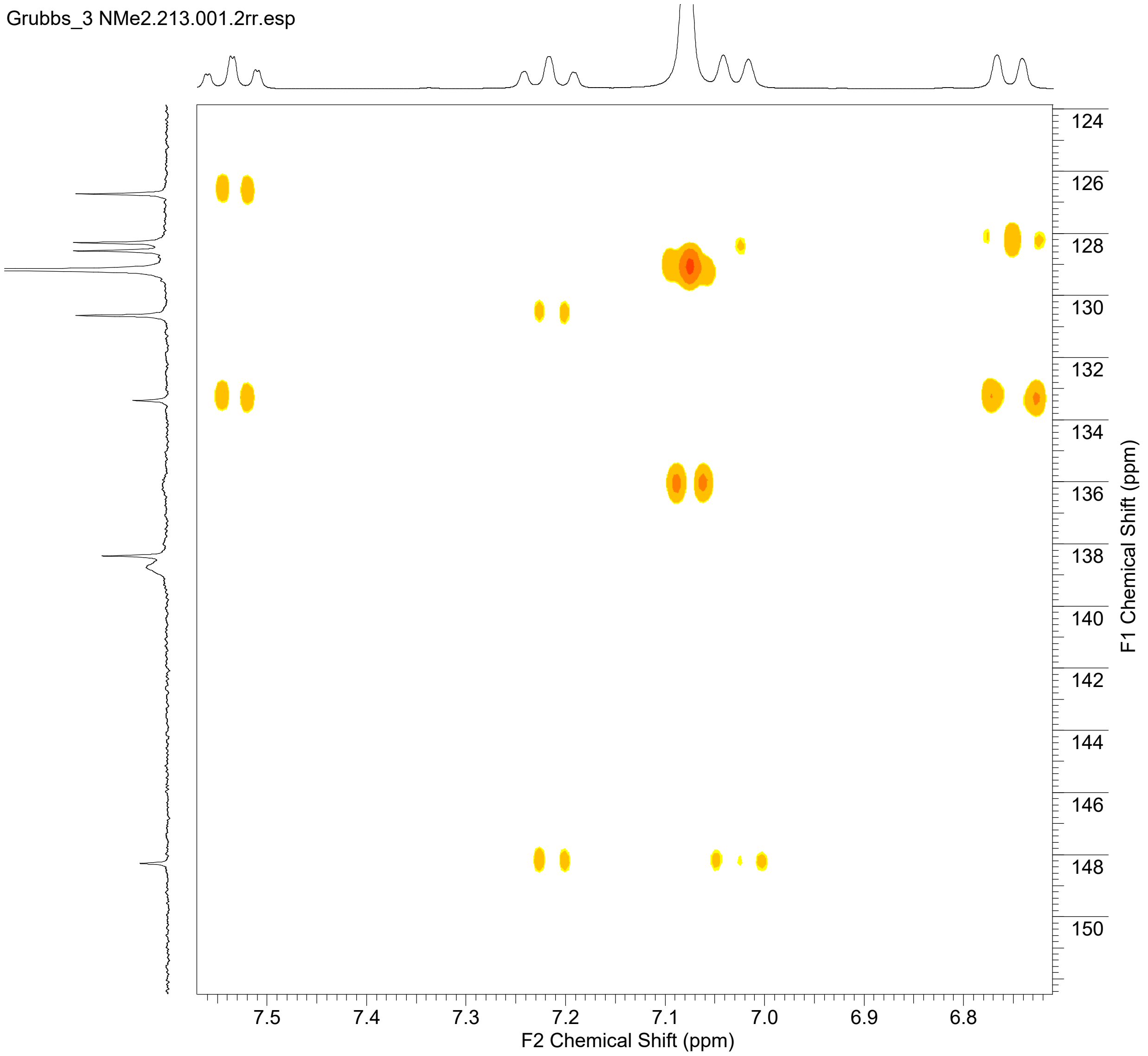


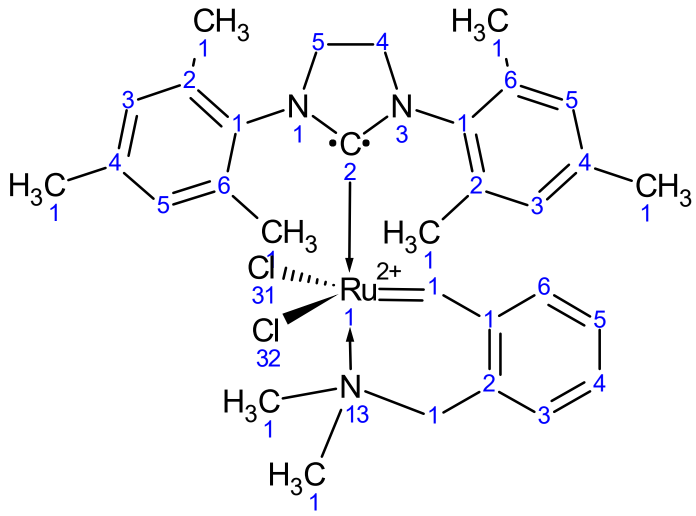

11a

| | | | | | |
|---|---|---|---|---|---|
| ***Acquisition Time (sec)*** | (0.3408, 0.0173) | ***Comment*** | 5 mm PABBO BB-1H/D Z-GRD Z104275/0345 | | |
| ***Date*** | 17 Jan 2018 21:32:24 | | | | |
| ***File Name*** | C:\Users\Fedor\Desktop\Наброски Статей\Кирилл Статья по Катализаторам Граббса\ЯМР Граббс\ЯМР Граббс от Ромы\Grubbs_3 NMe2\213\pdata\1\2rr | | | | |
| ***Frequency (MHz)*** | (300.13, 75.48) | ***Nucleus*** | (1H, 13C) | ***Number of Transients*** | 6 |
| ***Origin*** | spect | ***Original Points Count*** | (3072, 590) | ***Owner*** | nmr |
| ***Points Count*** | (8192, 2048) | ***Pulse Sequence*** | hmbcgplpndqf | ***Solvent*** | CD2Cl2 |
| ***Sweep Width (Hz)*** | (9014.42, 34013.61) | ***Temperature (degree C)*** | 30.007 | ***Title*** | FZ_Grubbs_3_1-HMBC |

| | |
|---|---|
| ***Formula*** $C_{31}H_{39}Cl_2N_3Ru$ | ***FW*** 625.6364 |

Grubbs_3 NMe2.213.001.2rr.esp

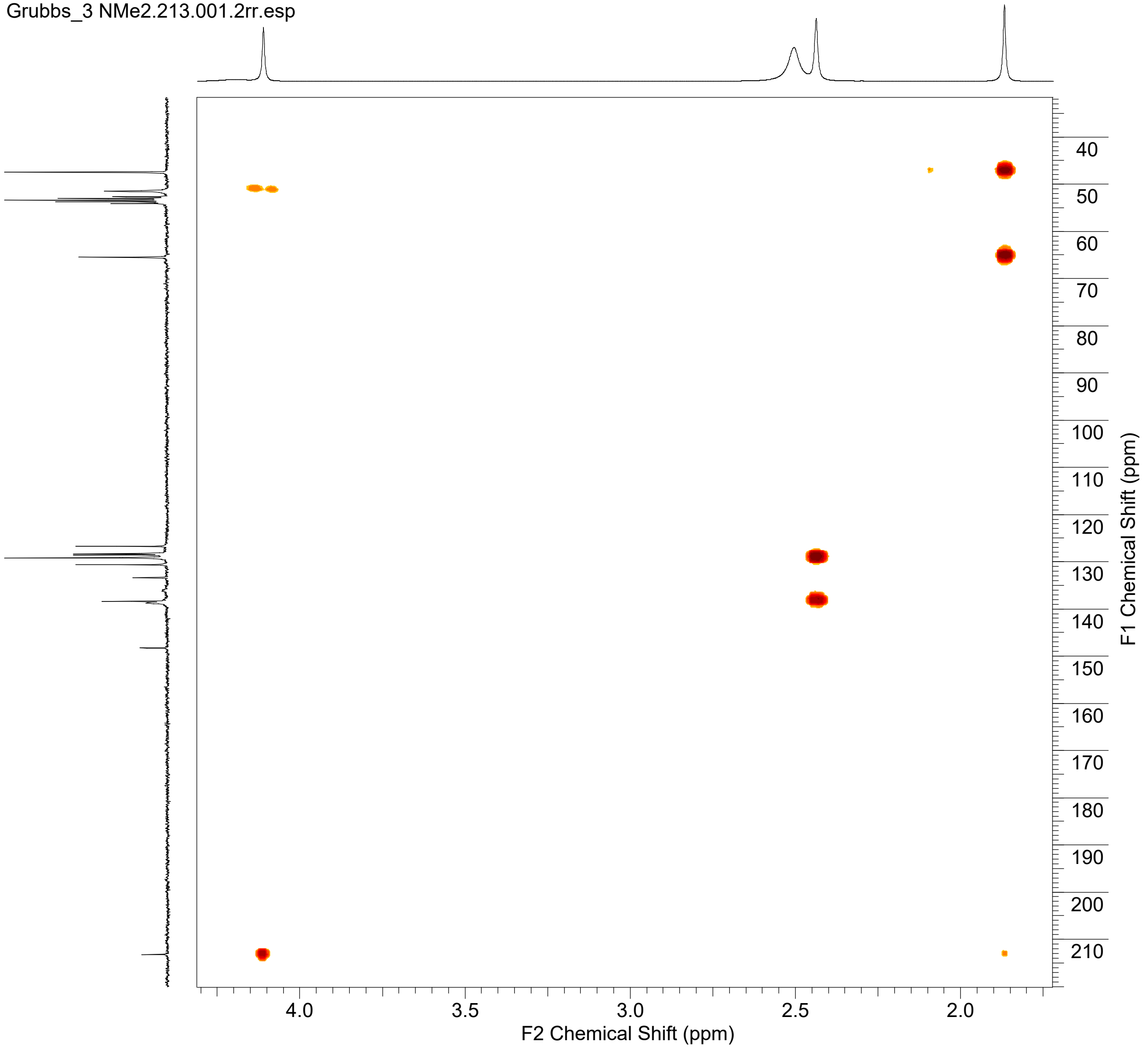


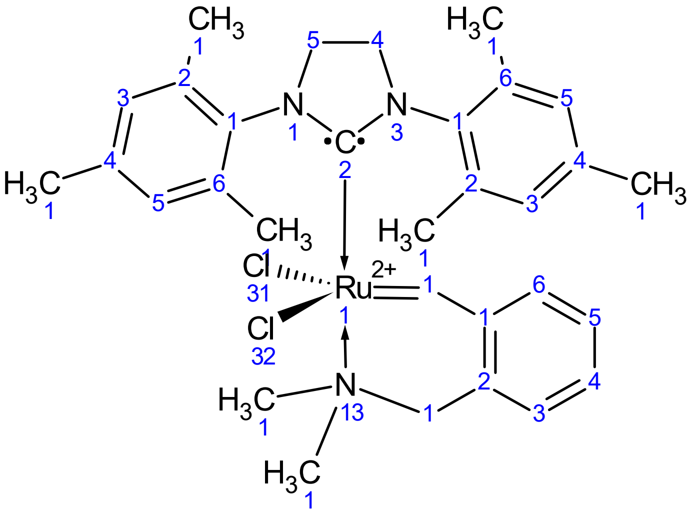


11a

FZ_Grubbs_3_1-N15-HMBC

11a

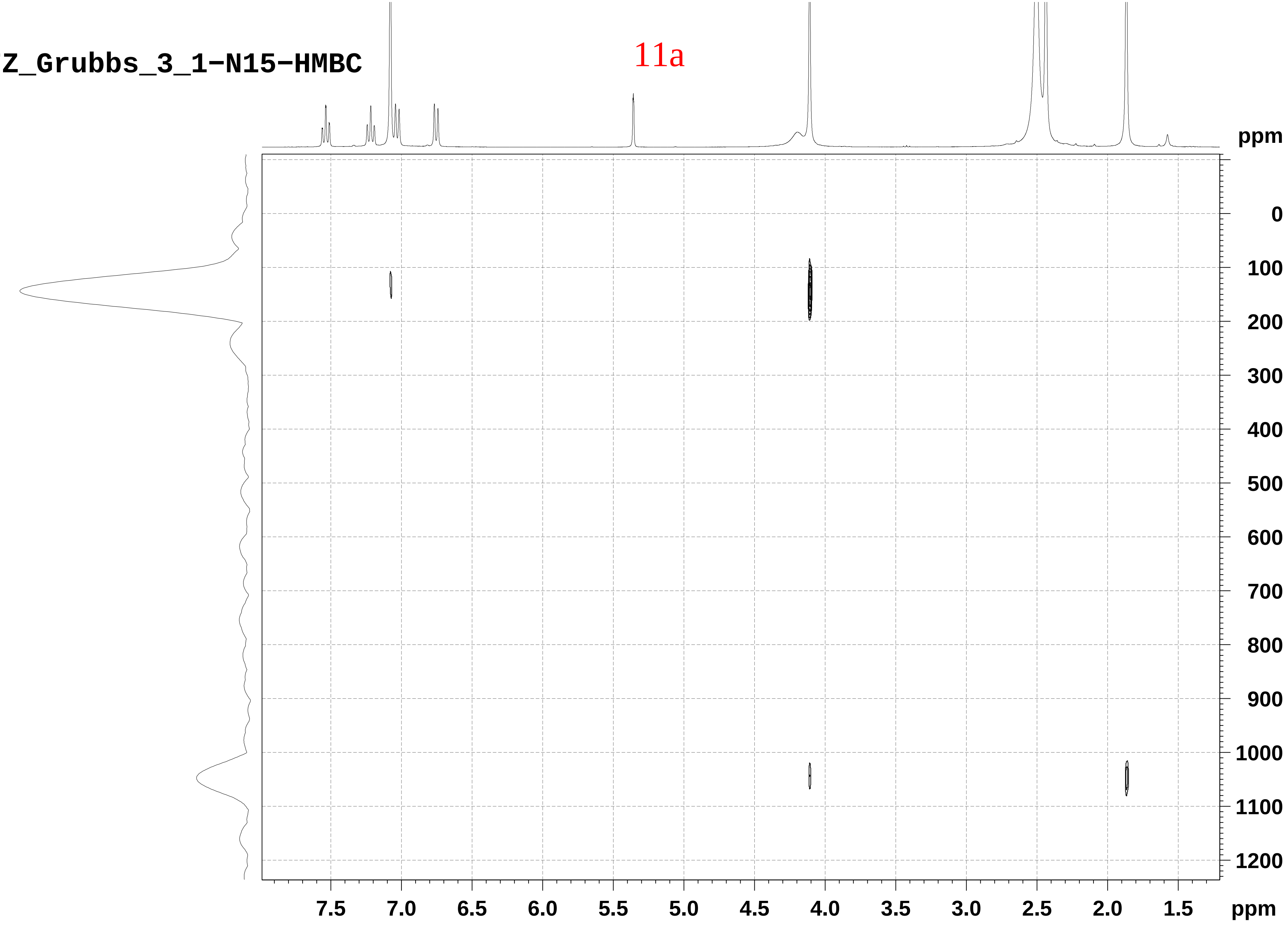

| | | | | | | | |
|---|---|---|---|---|---|---|---|
| ***Acquisition Time (sec)*** | 2.3243 | ***Comment*** | 5 mm CPPBBO BB-1H/19F/D Z-GRD Z125869/0025 | | | ***Date*** | 21 Feb 2017 05:26:24 |
| ***Date Stamp*** | 21 Feb 2017 05:26:24 | | | | | | |
| ***File Name*** | C:\Users\Fedor\Desktop\Наброски Статей\Кирилл Статья по Катализаторам Граббса\ЯМР Граббс\Kirill-NEW1 Видно слабое поле\kp2102-2017-K1-NEt2_001001r | | | | | | |
| ***Frequency (MHz)*** | 500.13 | ***Nucleus*** | 1H | ***Number of Transients*** | 8 | ***Origin*** | spect |
| ***Original Points Count*** | 32768 | ***Owner*** | nmrsu | ***Points Count*** | 262144 | ***Pulse Sequence*** | zg30 |
| ***Receiver Gain*** | 122.15 | ***SW(cyclical) (Hz)*** | 14097.74 | ***Solvent*** | DICHLOROMETHANE-d2 | | |
| ***Spectrum Offset (Hz)*** | 4088.6934 | ***Sweep Width (Hz)*** | 14097.69 | ***Temperature (degree C)*** | 25.017 | | |

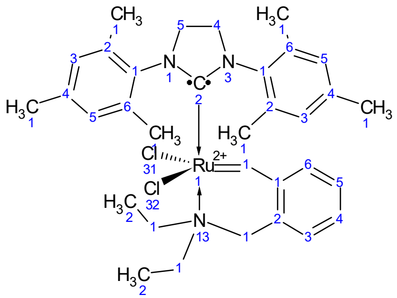


kp2102-2017-K1-NEt2_001001r

11b

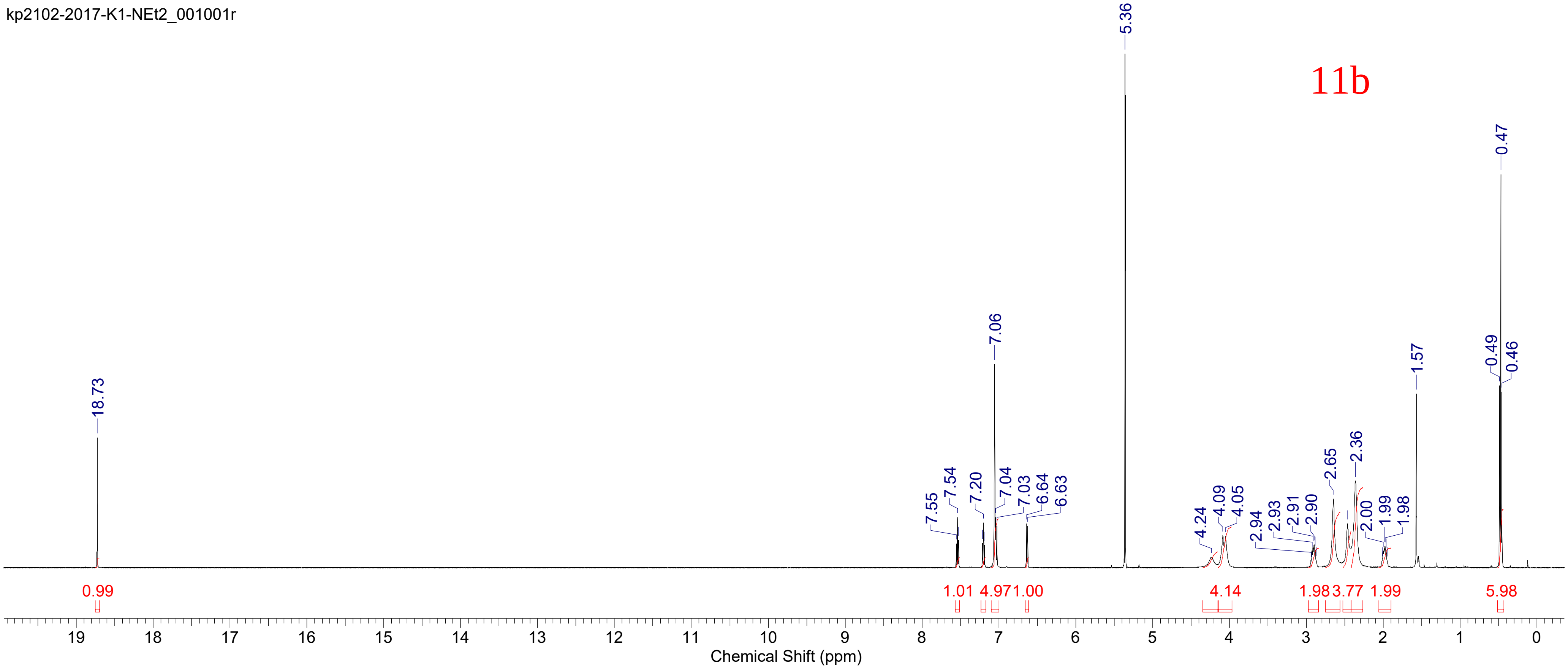

| | | | | | | | |
|---|---|---|---|---|---|---|---|
| **Acquisition Time (sec)** | 2.3243 | **Comment** | 5 mm CPPBBO BB-1H/19F/D Z-GRD Z125869/0025 | | | **Date** | 21 Feb 2017 05:26:24 |
| **Date Stamp** | 21 Feb 2017 05:26:24 | | | | | | |
| **File Name** | C:\Users\Fedor\Desktop\Наброски Статей\Кирилл Статья по Катализаторам Граббса\ЯМР Граббс\Kirill-NEW1 Видно слабое поле\kp2102-2017-K1-NEt2_001001r | | | | | | |
| **Frequency (MHz)** | 500.13 | **Nucleus** | 1H | **Number of Transients** | 8 | **Origin** | spect |
| **Original Points Count** | 32768 | **Owner** | nmrsu | **Points Count** | 262144 | **Pulse Sequence** | zg30 |
| **Receiver Gain** | 122.15 | **SW(cyclical) (Hz)** | 14097.74 | **Solvent** | DICHLOROMETHANE-d2 | | |
| **Spectrum Offset (Hz)** | 4088.6934 | **Sweep Width (Hz)** | 14097.69 | **Temperature (degree C)** | 25.017 | | |

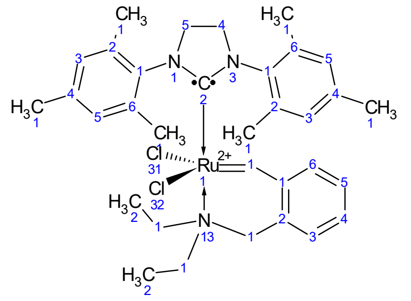

kp2102-2017-K1-NEt2_001001r

11b

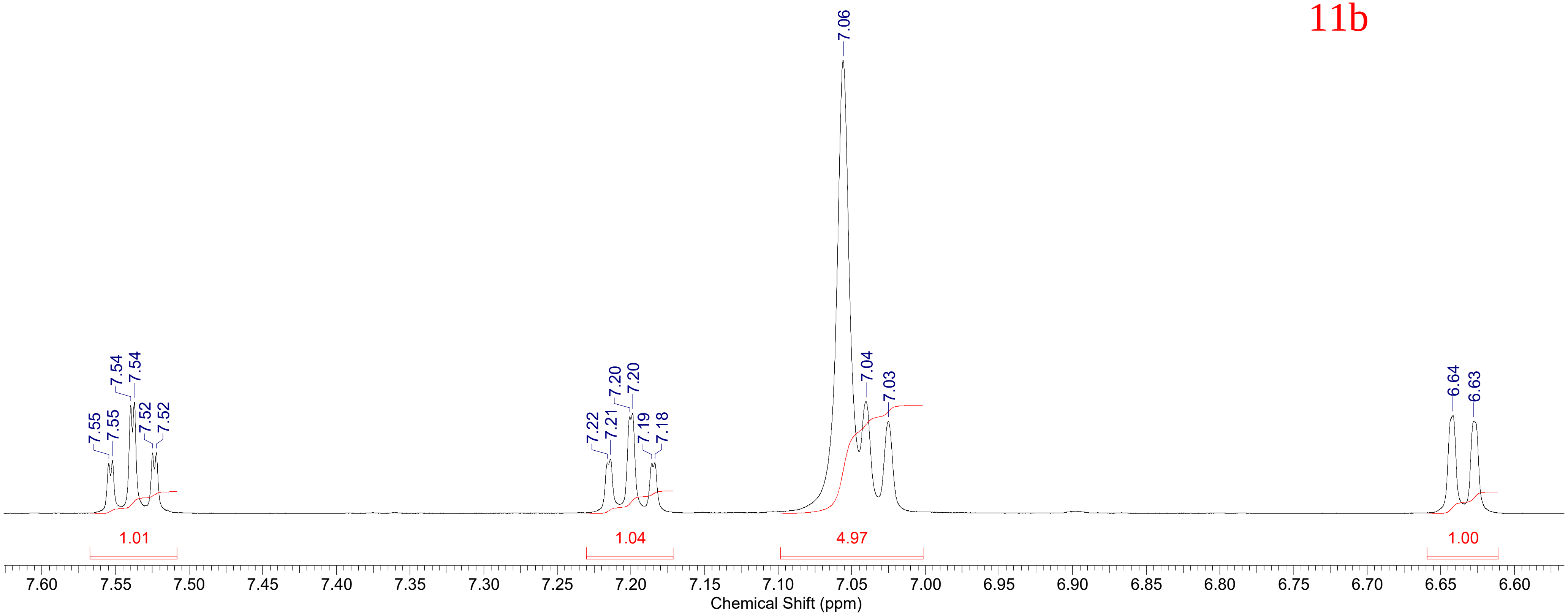

| | | | | | | | |
|---|---|---|---|---|---|---|---|
| ***Acquisition Time (sec)*** | 2.3243 | ***Comment*** | 5 mm CPPBBO BB-1H/19F/D Z-GRD Z125869/0025 | | | ***Date*** | 21 Feb 2017 05:26:24 |
| ***Date Stamp*** | 21 Feb 2017 05:26:24 | | | | | | |
| ***File Name*** | C:\Users\Fedor\Desktop\Наброски Статей\Кирилл Статья по Катализаторам Граббса\ЯМР Граббс\Kirill-NEW1 Видно слабое поле\kp2102-2017-K1-NEt2_001001r | | | | | | |
| ***Frequency (MHz)*** | 500.13 | ***Nucleus*** | 1H | ***Number of Transients*** | 8 | ***Origin*** | spect |
| ***Original Points Count*** | 32768 | ***Owner*** | nmrsu | ***Points Count*** | 262144 | ***Pulse Sequence*** | zg30 |
| ***Receiver Gain*** | 122.15 | ***SW(cyclical) (Hz)*** | 14097.74 | ***Solvent*** | DICHLOROMETHANE-d2 | | |
| ***Spectrum Offset (Hz)*** | 4088.6934 | ***Sweep Width (Hz)*** | 14097.69 | ***Temperature (degree C)*** | 25.017 | | |

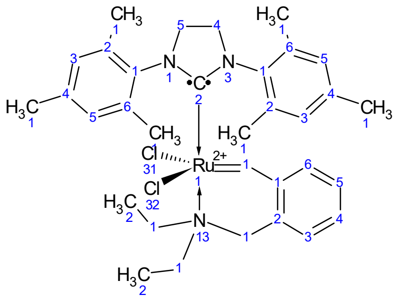

kp2102-2017-K1-NEt2_001001r

11b

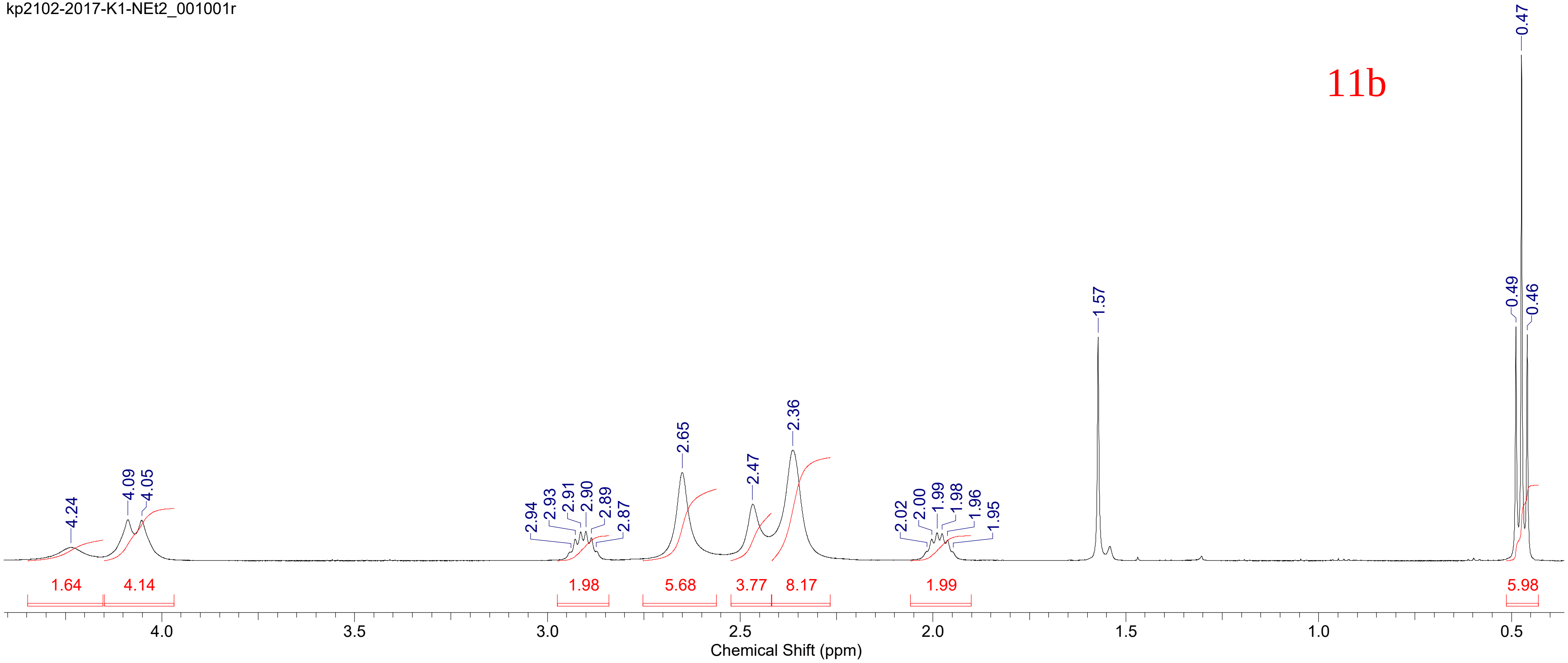

| | | | | | | | |
|---|---|---|---|---|---|---|---|
| ***Acquisition Time (sec)*** | 0.6554 | ***Comment*** | 5 mm CPPBBO BB-1H/19F/D Z-GRD Z125869/0025 | | | ***Date*** | 21 Feb 2017 06:09:04 |
| ***Date Stamp*** | 21 Feb 2017 06:09:04 | | | | | | |
| ***File Name*** | C:\Users\Fedor\Desktop\Наброски Статей\Кирилл Статья по Катализаторам Граббса\ЯМР Граббс\Kirill-NEW1 Видно слабое поле\kp2102-2017-K1-NEt2_013001r | | | | | | |
| ***Frequency (MHz)*** | 125.76 | ***Nucleus*** | 13C | ***Number of Transients*** | 193 | ***Origin*** | spect |
| ***Original Points Count*** | 32768 | ***Owner*** | nmrsu | ***Points Count*** | 32768 | ***Pulse Sequence*** | jmod |
| ***Receiver Gain*** | 189.77 | ***SW(cyclical) (Hz)*** | 50000.00 | ***Solvent*** | DICHLOROMETHANE-d2 | | |
| ***Spectrum Offset (Hz)*** | 25151.5527 | ***Sweep Width (Hz)*** | 49998.47 | ***Temperature (degree C)*** | 25.037 | | |

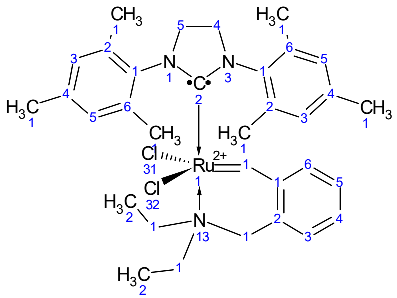

11b

kp2102-2017-K1-NEt2_013001r

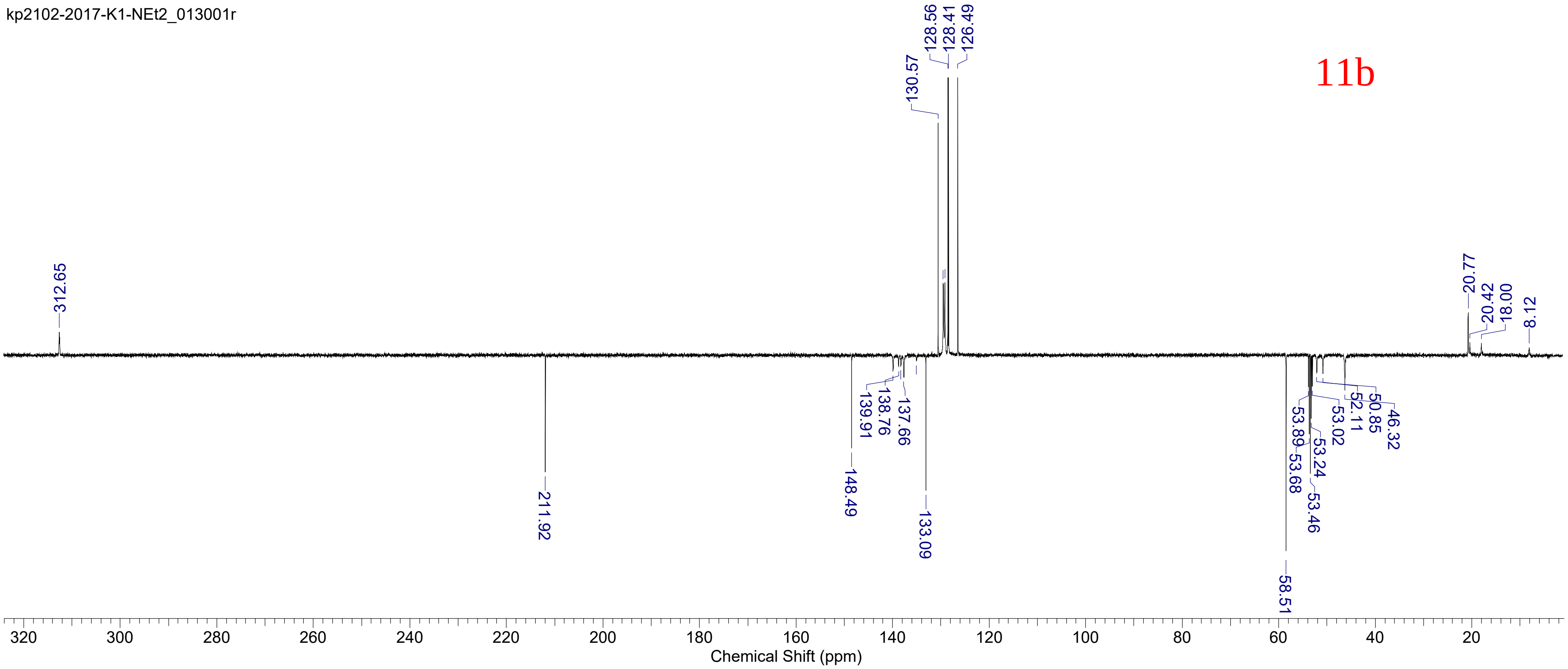

| | | | | | | | |
|---|---|---|---|---|---|---|---|
| ***Acquisition Time (sec)*** | 0.6554 | ***Comment*** | 5 mm CPPBBO BB-1H/19F/D Z-GRD Z125869/0025 | | | ***Date*** | 21 Feb 2017 06:09:04 |
| ***Date Stamp*** | 21 Feb 2017 06:09:04 | | | | | | |
| ***File Name*** | C:\Users\Fedor\Desktop\Наброски Статей\Кирилл Статья по Катализаторам Граббса\ЯМР Граббс\Kirill-NEW1 Видно слабое поле\kp2102-2017-K1-NEt2_013001r | | | | | | |
| ***Frequency (MHz)*** | 125.76 | ***Nucleus*** | 13C | ***Number of Transients*** | 193 | ***Origin*** | spect |
| ***Original Points Count*** | 32768 | ***Owner*** | nmrsu | ***Points Count*** | 32768 | ***Pulse Sequence*** | jmod |
| ***Receiver Gain*** | 189.77 | ***SW(cyclical) (Hz)*** | 50000.00 | ***Solvent*** | DICHLOROMETHANE-d2 | | |
| ***Spectrum Offset (Hz)*** | 25151.5527 | ***Sweep Width (Hz)*** | 49998.47 | ***Temperature (degree C)*** | 25.037 | | |

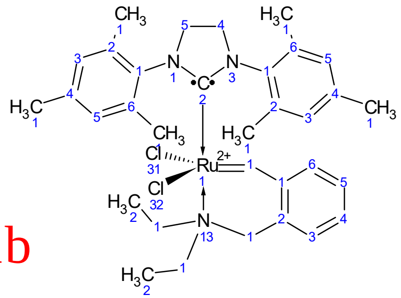


11b

kp2102-2017-K1-NEt2_013001r

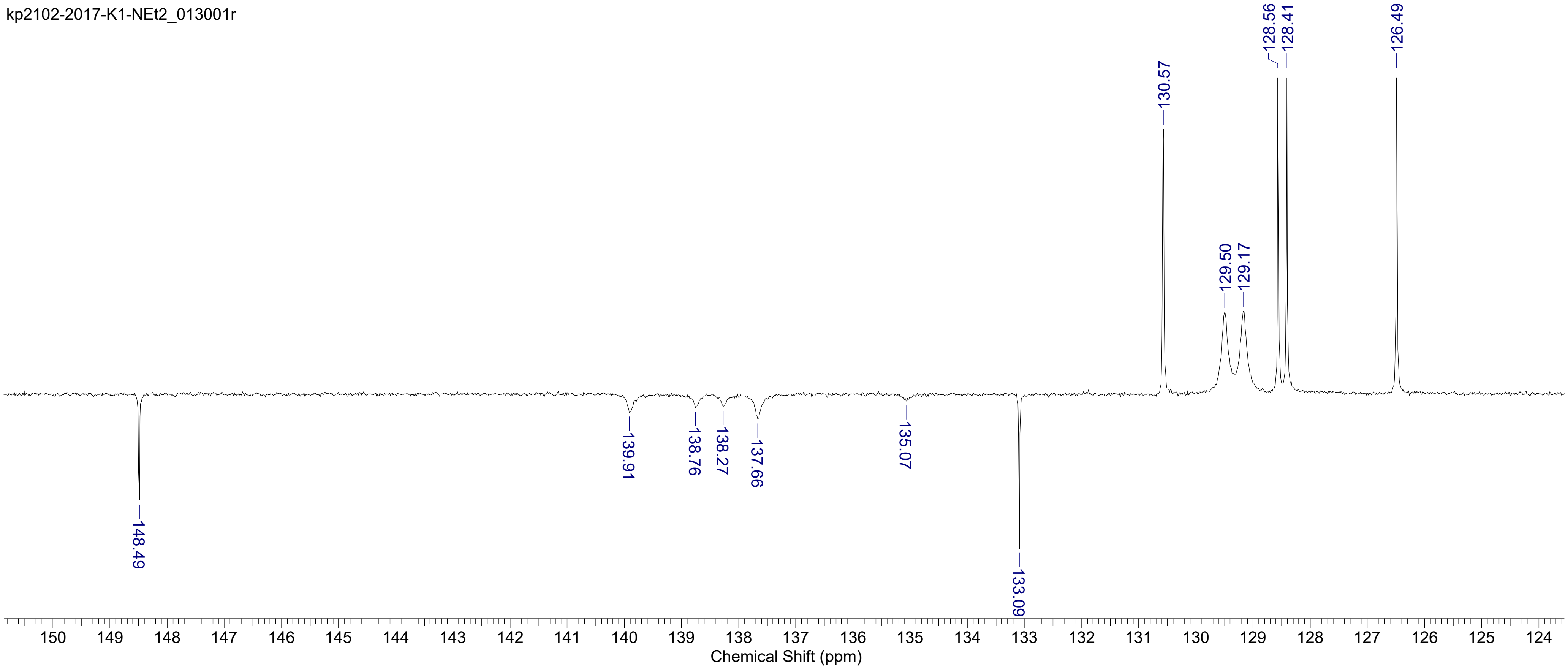

| | | | | | | | |
|---|---|---|---|---|---|---|---|
| *Acquisition Time (sec)* | 0.6554 | *Comment* | 5 mm CPPBBO BB-1H/19F/D Z-GRD Z125869/0025 | | | *Date* | 21 Feb 2017 06:09:04 |
| *Date Stamp* | 21 Feb 2017 06:09:04 | | | | | | |
| *File Name* | C:\Users\Fedor\Desktop\Наброски Статей\Кирилл Статья по Катализаторам Граббса\ЯМР Граббс\Kirill-NEW1 Видно слабое поле\kp2102-2017-K1-NEt2_013001r | | | | | | |
| *Frequency (MHz)* | 125.76 | *Nucleus* | 13C | *Number of Transients* | 193 | *Origin* | spect |
| *Original Points Count* | 32768 | *Owner* | nmrsu | *Points Count* | 32768 | *Pulse Sequence* | jmod |
| *Receiver Gain* | 189.77 | *SW(cyclical) (Hz)* | 50000.00 | *Solvent* | DICHLOROMETHANE-d2 | | |
| *Spectrum Offset (Hz)* | 25151.5527 | *Sweep Width (Hz)* | 49998.47 | *Temperature (degree C)* | 25.037 | | |

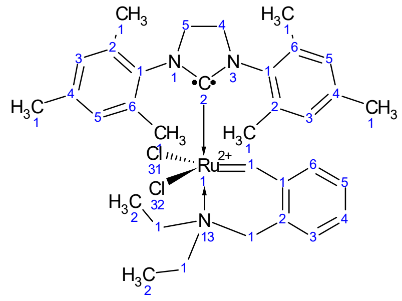

kp2102-2017-K1-NEt2_013001r

11b

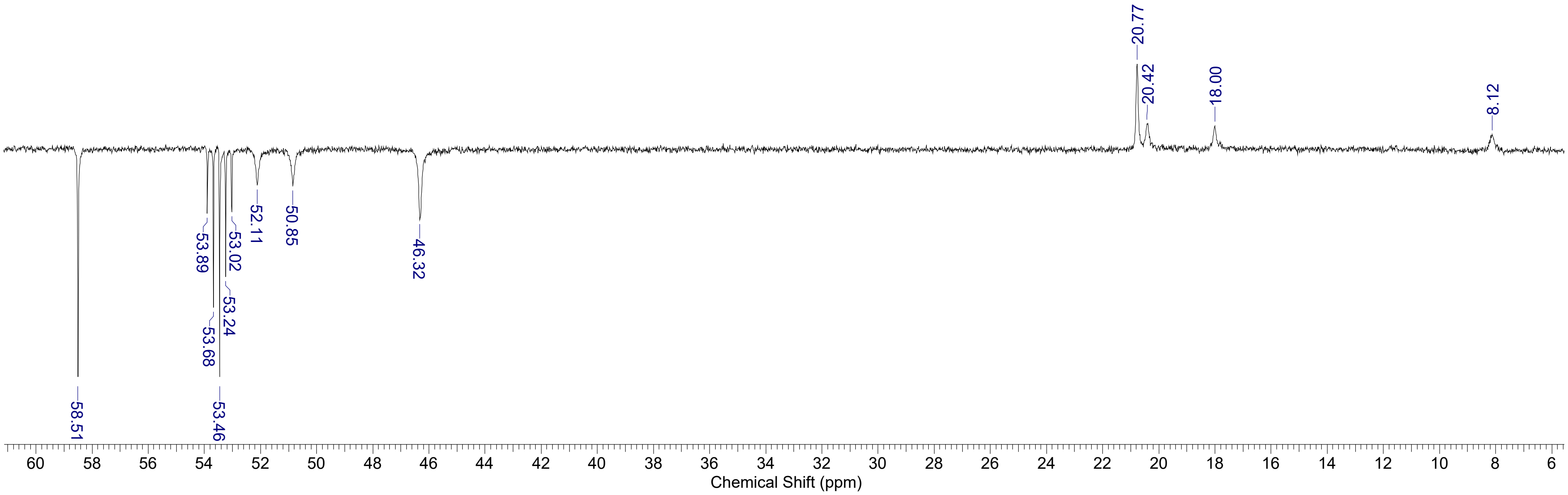

| | | | | | |
|---|---|---|---|---|---|
| ***Acquisition Time (sec)*** | (0.0512, 0.0128) | ***Comment*** | 5 mm CPPBBO BB-1H/19F/D Z-GRD Z125869/0025 | | |
| ***Date*** | 21 Feb 2017 09:34:24 | | | | |
| ***File Name*** | C:\Users\Fedor\Desktop\Наброски Статей\Кирилл Статья по Катализаторам Граббса\ЯМР Граббс\Kirill-NEW1 Видно слабое поле\kp2102-2017-K1-NEt2\10\pdata\1\2rr | | | | |
| ***Frequency (MHz)*** | (500.14, 500.14) | ***Nucleus*** | (1H, 1H) | ***Number of Transients*** | 1 |
| ***Origin*** | spect | ***Original Points Count*** | (512, 128) | ***Owner*** | nmrsu |
| ***Points Count*** | (1024, 1024) | ***Pulse Sequence*** | cosygpqf | ***Solvent*** | CD2Cl2 |
| ***Sweep Width (Hz)*** | (10000.00, 10000.00) | ***Temperature (degree C)*** | 25.096 | | |

| ***Formula*** $C_{33}H_{43}Cl_2N_3Ru$ | ***FW*** 653.6895 |
|---|---|

kp2102-2017-K1-NEt2.010.001.2rr.esp

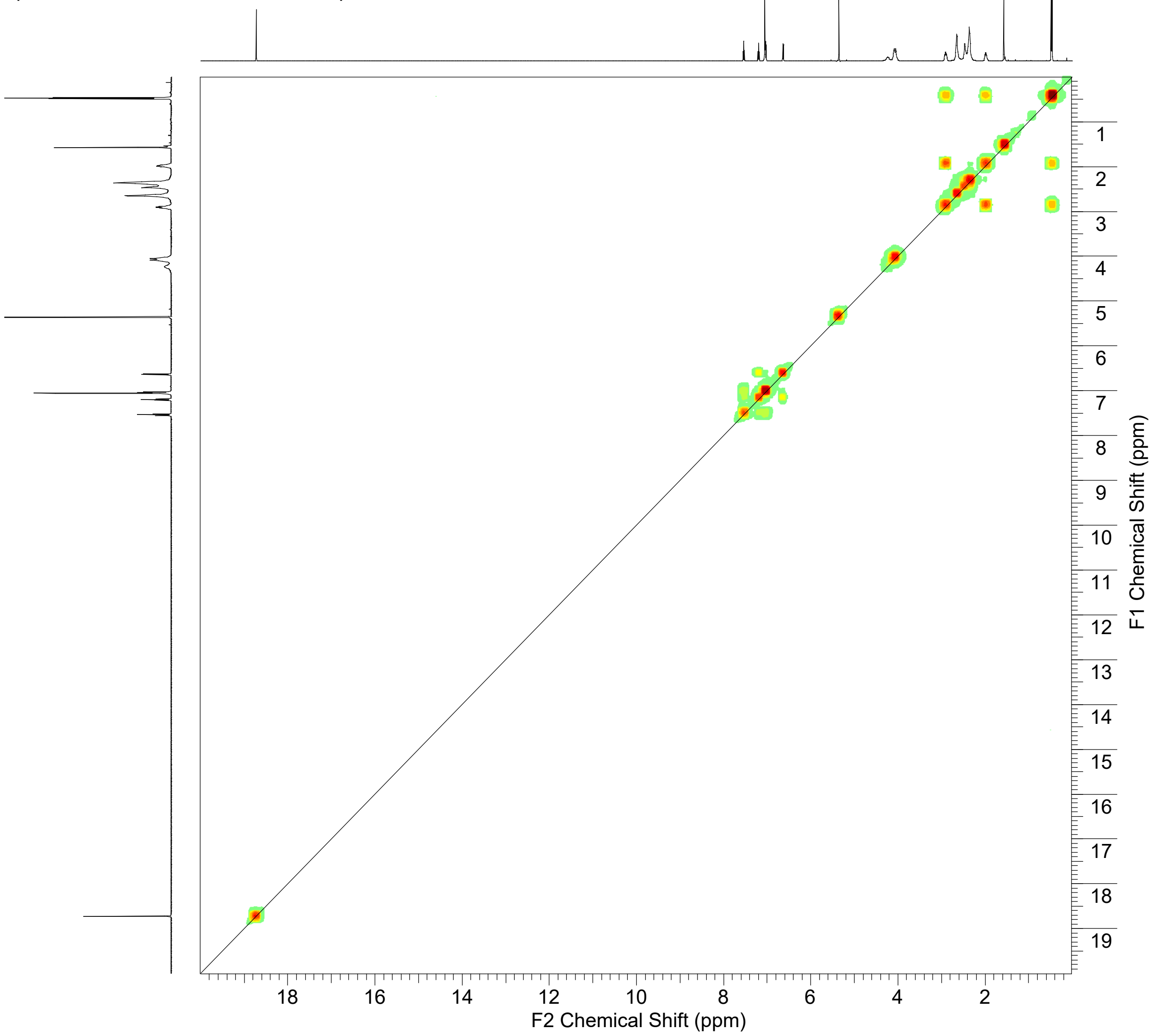


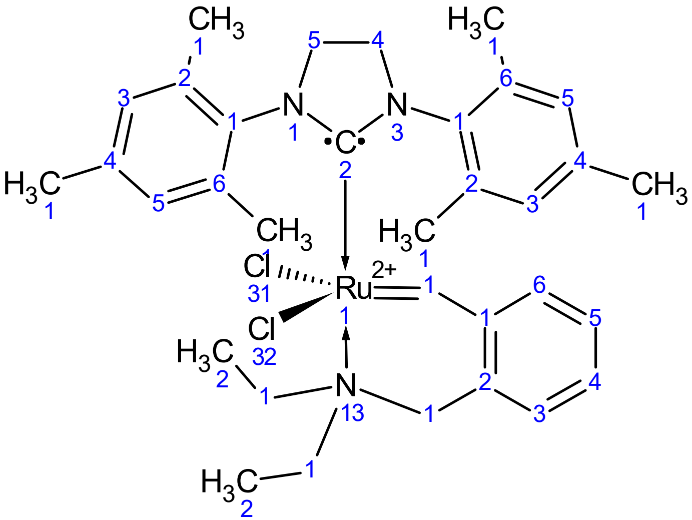


11b

| | | | | | |
|---|---|---|---|---|---|
| ***Acquisition Time (sec)*** | (0.0512, 0.0128) | ***Comment*** | 5 mm CPPBBO BB-1H/19F/D Z-GRD Z125869/0025 | | |
| ***Date*** | 21 Feb 2017 09:34:24 | | | | |
| ***File Name*** | C:\Users\Fedor\Desktop\Наброски Статей\Кирилл Статья по Катализаторам Граббса\ЯМР Граббс\Kirill-NEW1 Видно слабое поле\kp2102-2017-K1-NEt2\10\pdata\1\2rr | | | | |
| ***Frequency (MHz)*** | (500.14, 500.14) | ***Nucleus*** | (1H, 1H) | ***Number of Transients*** | 1 |
| ***Origin*** | spect | ***Original Points Count*** | (512, 128) | ***Owner*** | nmrsu |
| ***Points Count*** | (1024, 1024) | ***Pulse Sequence*** | cosygpqf | ***Solvent*** | CD2Cl2 |
| ***Sweep Width (Hz)*** | (10000.00, 10000.00) | ***Temperature (degree C)*** | 25.096 | | |

| | |
|---|---|
| ***Formula*** $C_{33}H_{43}Cl_2N_3Ru$ | ***FW*** 653.6895 |

kp2102-2017-K1-NEt2.010.001.2rr.esp

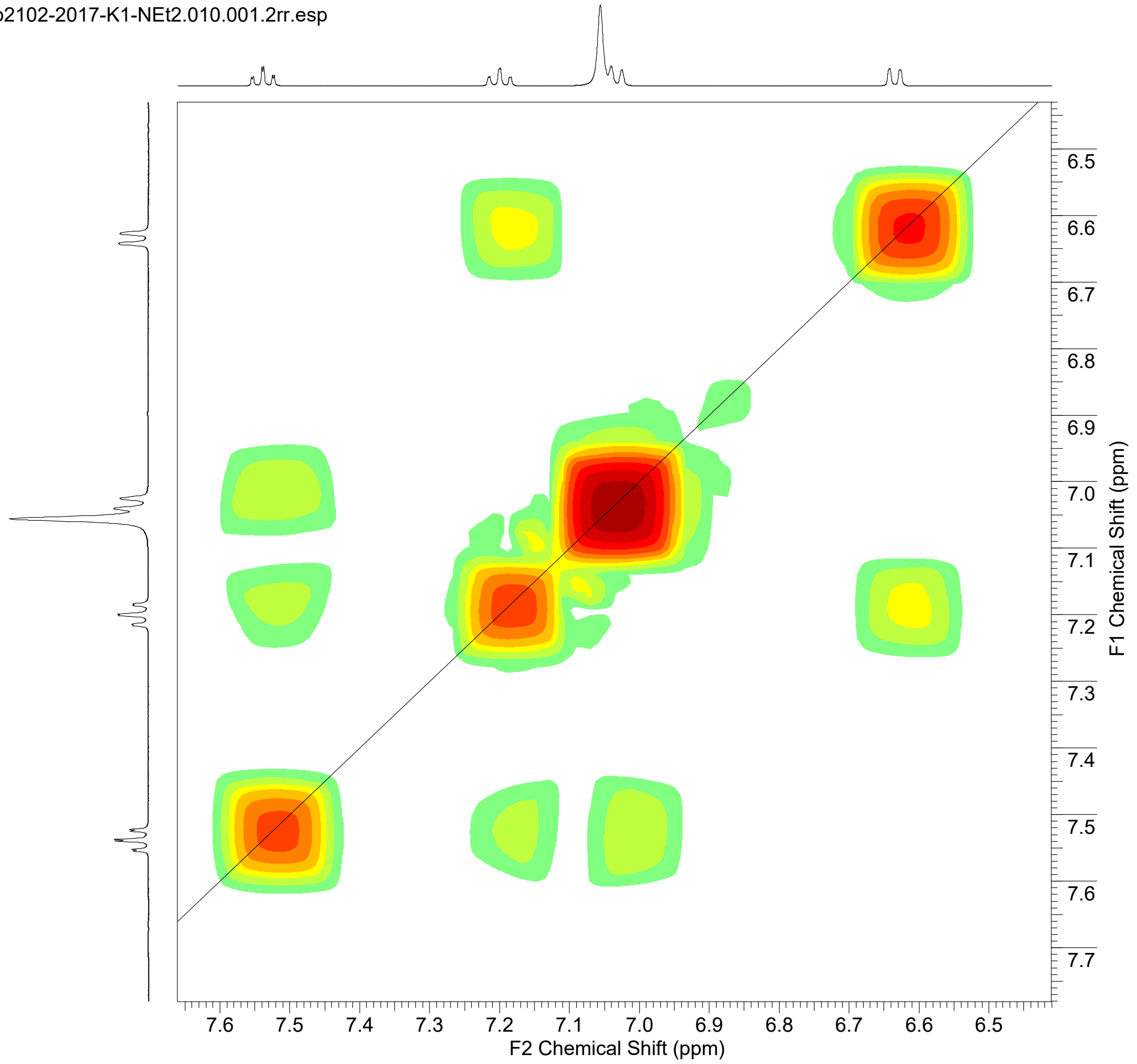


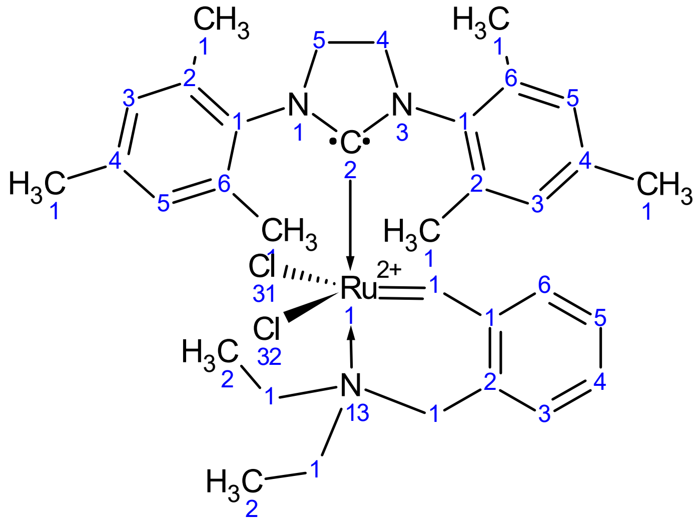

11b

| | | | | | |
|---|---|---|---|---|---|
| **Acquisition Time (sec)** | (0.0512, 0.0128) | **Comment** | 5 mm CPPBBO BB-1H/19F/D Z-GRD Z125869/0025 | | |
| **Date** | 21 Feb 2017 09:34:24 | | | | |
| **File Name** | C:\Users\Fedor\Desktop\Наброски Статей\Кирилл Статья по Катализаторам Граббса\ЯМР Граббс\Kirill-NEW1 Видно слабое поле\kp2102-2017-K1-NEt2\10\pdata\1\2rr | | | | |
| **Frequency (MHz)** | (500.14, 500.14) | **Nucleus** | (1H, 1H) | **Number of Transients** | 1 |
| **Origin** | spect | **Original Points Count** | (512, 128) | **Owner** | nmrsu |
| **Points Count** | (1024, 1024) | **Pulse Sequence** | cosygpqf | **Solvent** | CD2Cl2 |
| **Sweep Width (Hz)** | (10000.00, 10000.00) | **Temperature (degree C)** | 25.096 | | |

| | | | |
|---|---|---|---|
| **Formula** | $C_{33}H_{43}Cl_2N_3Ru$ | **FW** | 653.6895 |

kp2102-2017-K1-NEt2.010.001.2rr.esp

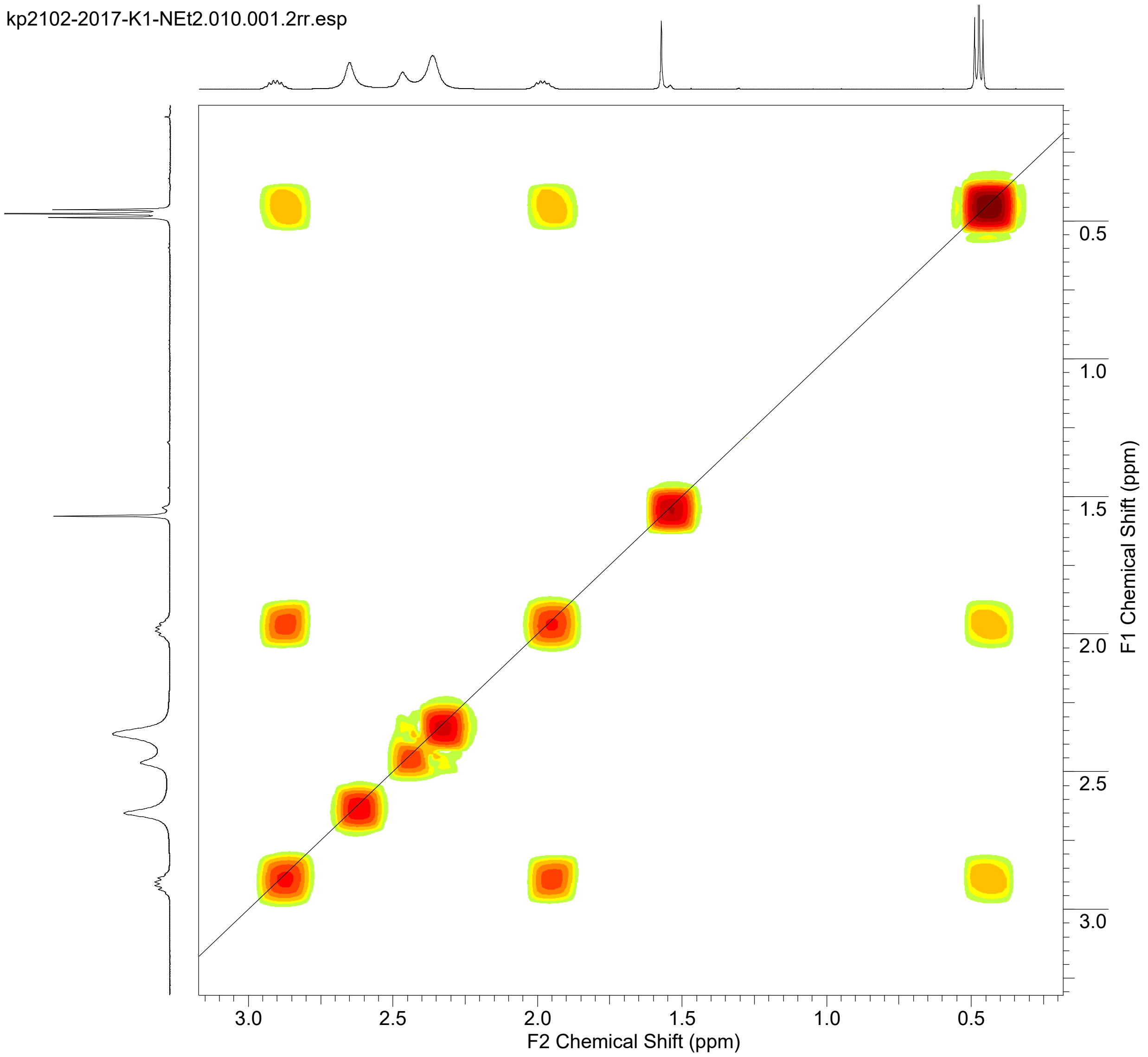


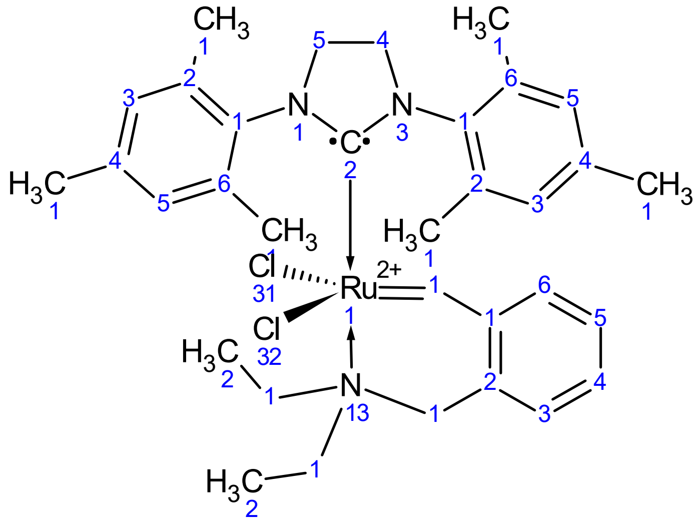

11b

| | | | | | |
|---|---|---|---|---|---|
| **Acquisition Time (sec)** | (0.0512, 0.0063) | **Comment** | 5 mm CPPBBO BB-1H/19F/D Z-GRD Z125869/0025 | | |
| **Date** | 21 Feb 2017 10:43:58 | | | | |
| **File Name** | C:\Users\Fedor\Desktop\Наброски Статей\Кирилл Статья по Катализаторам Граббса\ЯМР Граббс\Kirill-NEW1 Видно слабое поле\kp2102-2017-K1-NEt2\11\pdata\1\2rr | | | | |
| **Frequency (MHz)** | (500.14, 125.78) | **Nucleus** | (1H, 13C) | **Number of Transients** | 2 |
| **Origin** | spect | **Original Points Count** | (512, 256) | **Owner** | nmrsu |
| **Points Count** | (1024, 1024) | **Pulse Sequence** | hmqcgpqf | **Solvent** | CD2Cl2 |
| **Sweep Width (Hz)** | (10000.00, 40322.58) | **Temperature (degree C)** | 25.078 | | |

| | | | |
|---|---|---|---|
| **Formula** | $C_{33}H_{43}Cl_2N_3Ru$ | **FW** | 653.6895 |

kp2102-2017-K1-NEt2.011.001.2rr.esp

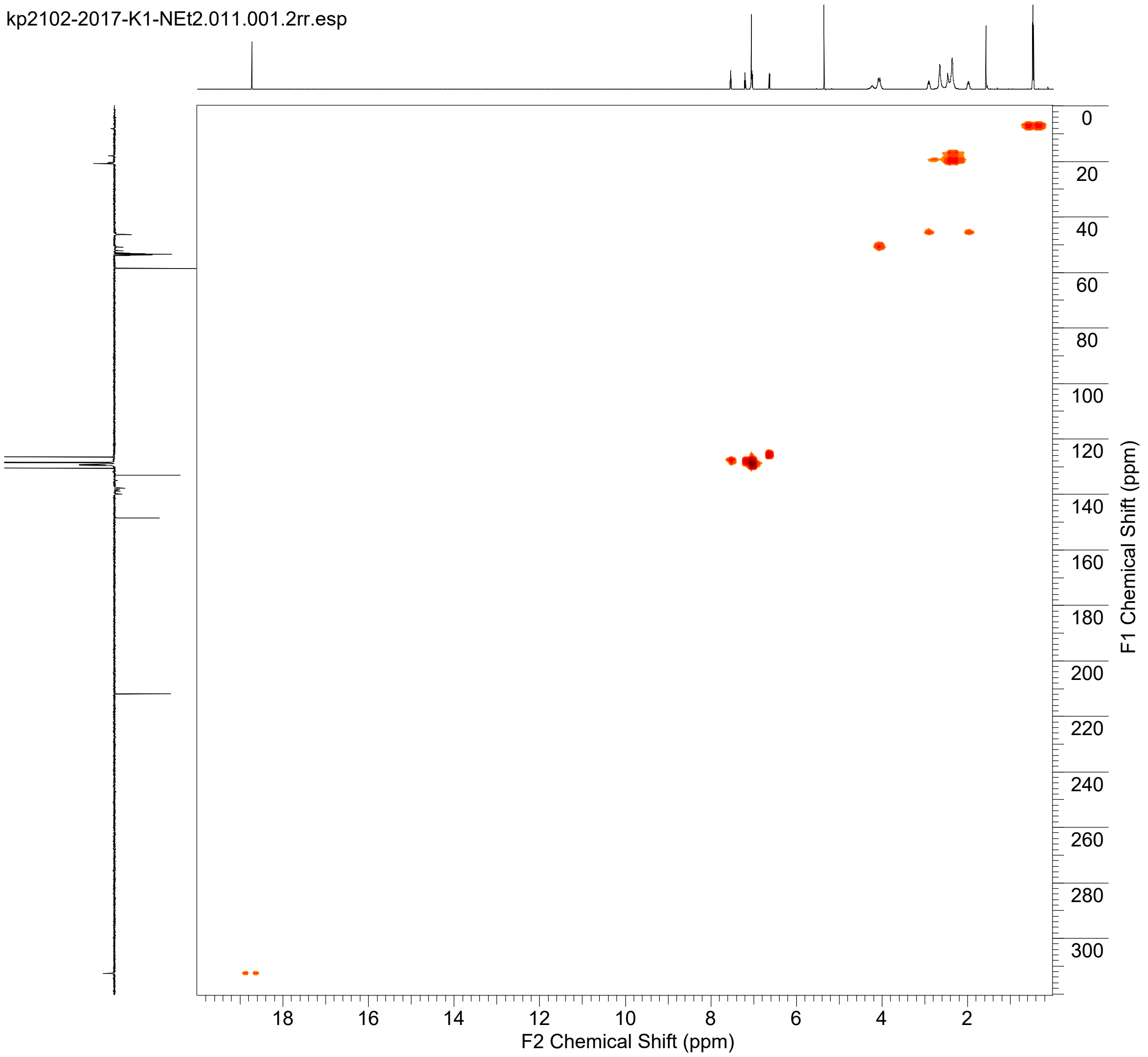


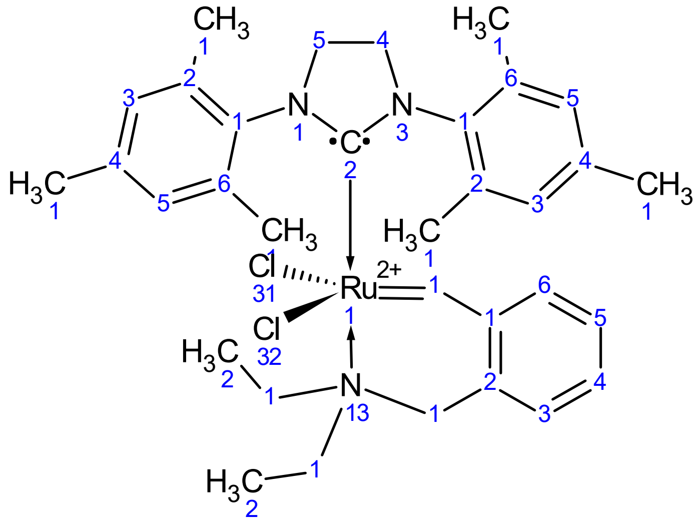

11b

| | | | | | |
|---|---|---|---|---|---|
| ***Acquisition Time (sec)*** | (0.0512, 0.0063) | ***Comment*** | 5 mm CPPBBO BB-1H/19F/D Z-GRD Z125869/0025 | | |
| ***Date*** | 21 Feb 2017 10:43:58 | | | | |
| ***File Name*** | C:\Users\Fedor\Desktop\Наброски Статей\Кирилл Статья по Катализаторам Граббса\ЯМР Граббс\Kirill-NEW1 Видно слабое поле\kp2102-2017-K1-NEt2\11\pdata\1\2rr | | | | |
| ***Frequency (MHz)*** | (500.14, 125.78) | ***Nucleus*** | (1H, 13C) | ***Number of Transients*** | 2 |
| ***Origin*** | spect | ***Original Points Count*** | (512, 256) | ***Owner*** | nmrsu |
| ***Points Count*** | (1024, 1024) | ***Pulse Sequence*** | hmqcgpqf | ***Solvent*** | CD2Cl2 |
| ***Sweep Width (Hz)*** | (10000.00, 40322.58) | ***Temperature (degree C)*** | 25.078 | | |

| | | | |
|---|---|---|---|
| ***Formula*** | $C_{33}H_{43}Cl_2N_3Ru$ | ***FW*** | 653.6895 |

kp2102-2017-K1-NEt2.011.001.2rr.esp

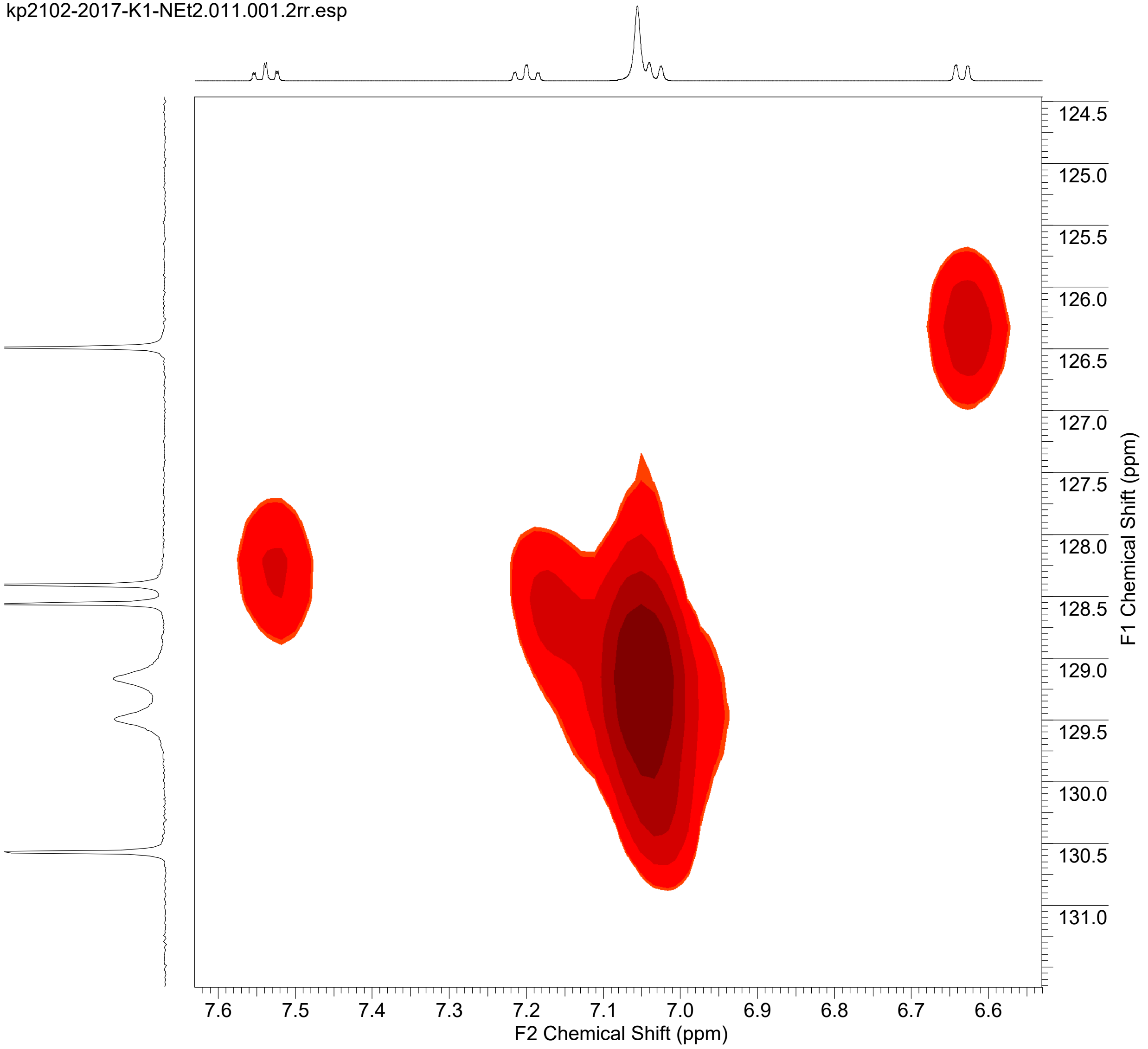


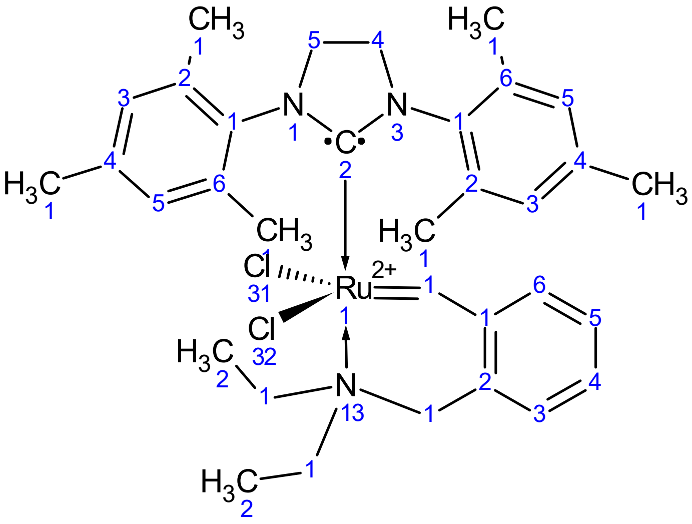

11b

| | | | | | |
|---|---|---|---|---|---|
| ***Acquisition Time (sec)*** | (0.0512, 0.0063) | ***Comment*** | 5 mm CPPBBO BB-1H/19F/D Z-GRD Z125869/0025 | | |
| ***Date*** | 21 Feb 2017 10:43:58 | | | | |
| ***File Name*** | C:\Users\Fedor\Desktop\Наброски Статей\Кирилл Статья по Катализаторам Граббса\ЯМР Граббс\Kirill-NEW1 Видно слабое поле\kp2102-2017-K1-NEt2\11\pdata\1\2rr | | | | |
| ***Frequency (MHz)*** | (500.14, 125.78) | ***Nucleus*** | (1H, 13C) | ***Number of Transients*** | 2 |
| ***Origin*** | spect | ***Original Points Count*** | (512, 256) | ***Owner*** | nmrsu |
| ***Points Count*** | (1024, 1024) | ***Pulse Sequence*** | hmqcgpqf | ***Solvent*** | CD2Cl2 |
| ***Sweep Width (Hz)*** | (10000.00, 40322.58) | ***Temperature (degree C)*** | 25.078 | | |

| | |
|---|---|
| ***Formula*** $C_{33}H_{43}Cl_2N_3Ru$ | ***FW*** 653.6895 |

kp2102-2017-K1-NEt2.011.001.2rr.esp

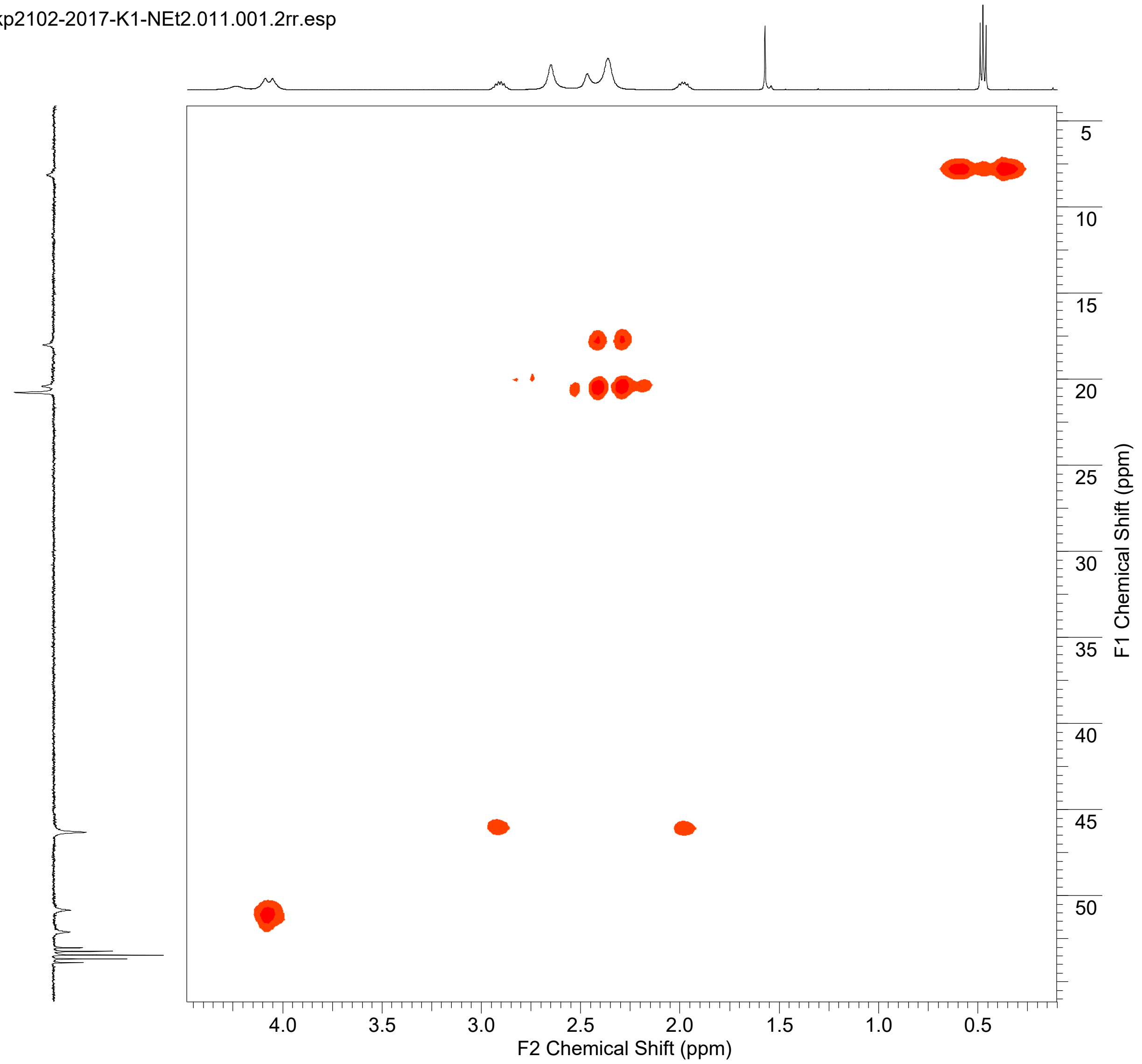


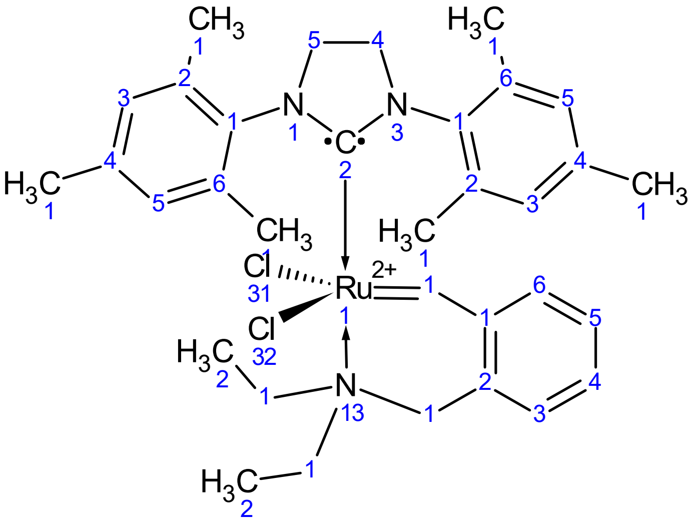

11b

| | | | | | |
|---|---|---|---|---|---|
| ***Acquisition Time (sec)*** | (0.0512, 0.0063) | ***Comment*** | 5 mm CPPBBO BB-1H/19F/D Z-GRD Z125869/0025 | | |
| ***Date*** | 21 Feb 2017 11:02:14 | | | | |
| ***File Name*** | C:\Users\Fedor\Desktop\Наброски Статей\Кирилл Статья по Катализаторам Граббса\ЯМР Граббс\Kirill-NEW1 Видно слабое поле\kp2102-2017-K1-NEt2\12\pdata\1\2rr | | | | |
| ***Frequency (MHz)*** | (500.14, 125.78) | ***Nucleus*** | (1H, 13C) | ***Number of Transients*** | 4 |
| ***Origin*** | spect | ***Original Points Count*** | (512, 256) | ***Owner*** | nmrsu |
| ***Points Count*** | (1024, 1024) | ***Pulse Sequence*** | hmbcgplpndqf | ***Solvent*** | CD2Cl2 |
| ***Sweep Width (Hz)*** | (10000.00, 40322.58) | ***Temperature (degree C)*** | 24.970 | | |

| | | | |
|---|---|---|---|
| ***Formula*** | $C_{33}H_{43}Cl_2N_3Ru$ | ***FW*** | 653.6895 |

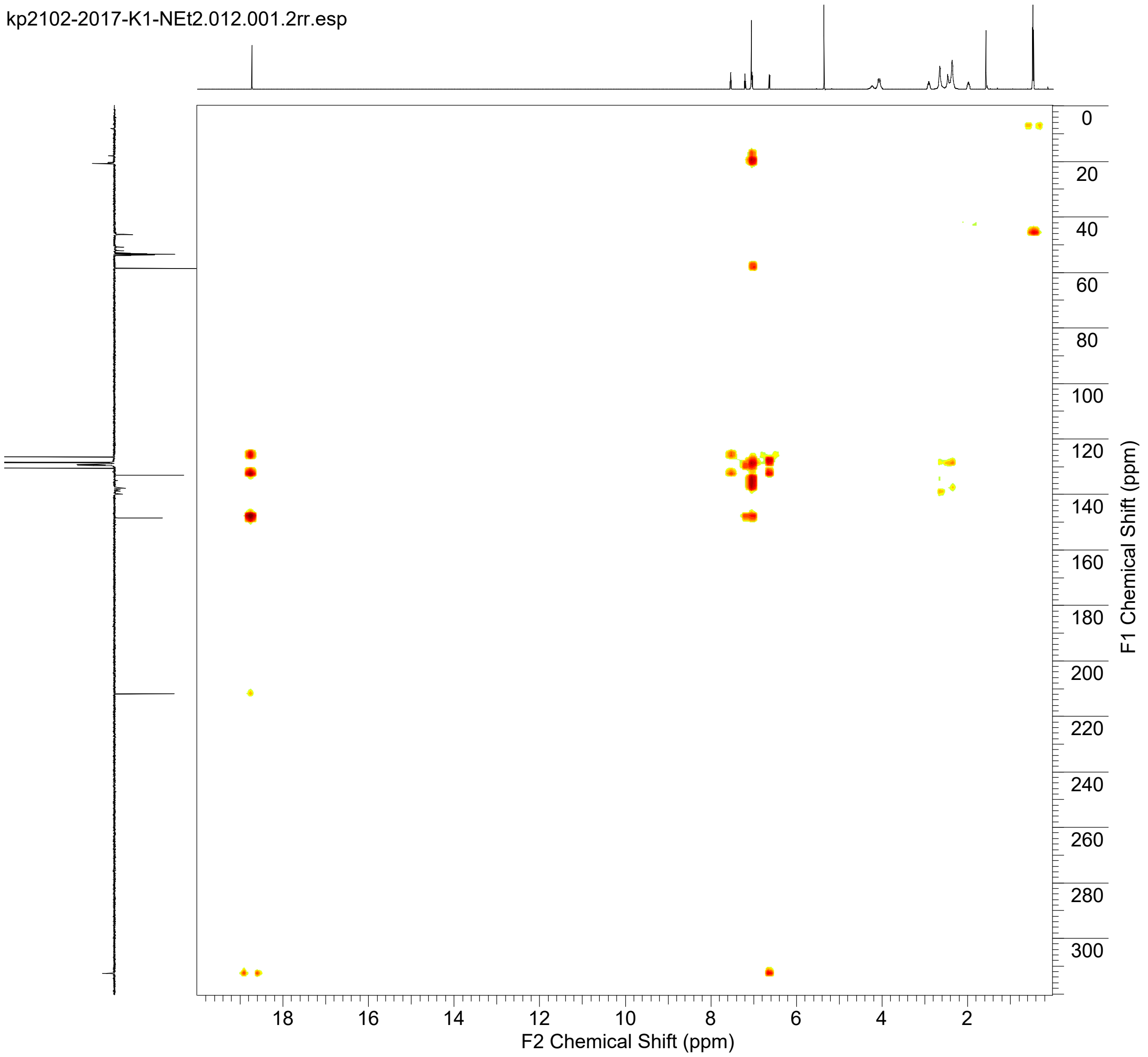


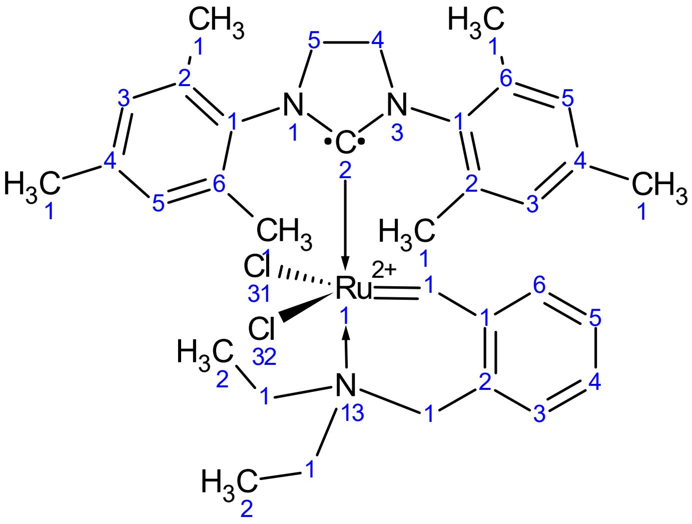

11b

| | | | | | |
|---|---|---|---|---|---|
| ***Acquisition Time (sec)*** | (0.0512, 0.0063) | ***Comment*** | 5 mm CPPBBO BB-1H/19F/D Z-GRD Z125869/0025 | | |
| ***Date*** | 21 Feb 2017 11:02:14 | | | | |
| ***File Name*** | C:\Users\Fedor\Desktop\Наброски Статей\Кирилл Статья по Катализаторам Граббса\ЯМР Граббс\Kirill-NEW1 Видно слабое поле\kp2102-2017-K1-NEt2\12\pdata\1\2rr | | | | |
| ***Frequency (MHz)*** | (500.14, 125.78) | ***Nucleus*** | (1H, 13C) | ***Number of Transients*** | 4 |
| ***Origin*** | spect | ***Original Points Count*** | (512, 256) | ***Owner*** | nmrsu |
| ***Points Count*** | (1024, 1024) | ***Pulse Sequence*** | hmbcgplpndqf | ***Solvent*** | CD2Cl2 |
| ***Sweep Width (Hz)*** | (10000.00, 40322.58) | ***Temperature (degree C)*** | 24.970 | | |

| | | | |
|---|---|---|---|
| ***Formula*** | $C_{33}H_{43}Cl_2N_3Ru$ | ***FW*** | 653.6895 |

kp2102-2017-K1-NEt2.012.001.2rr.esp

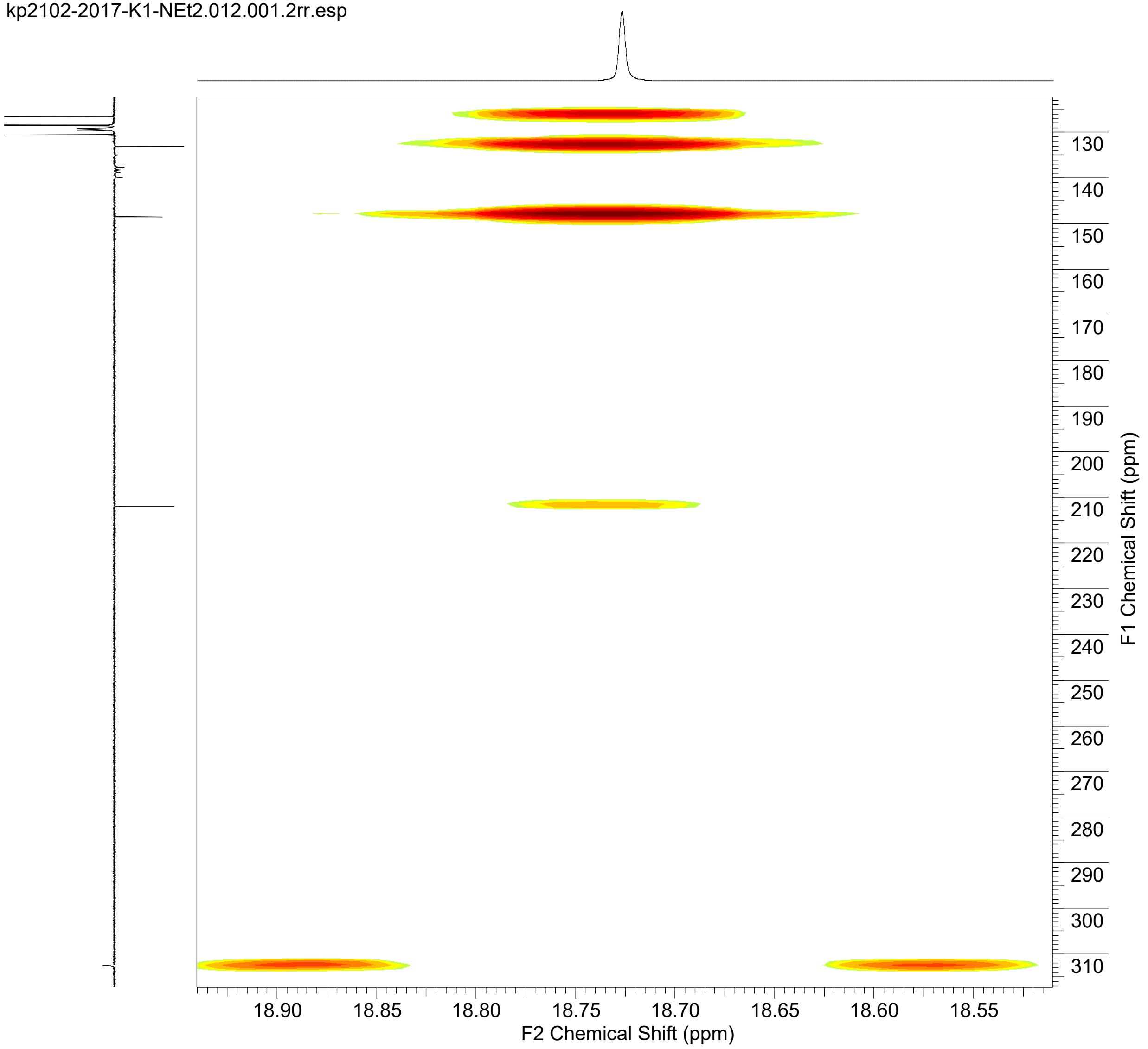


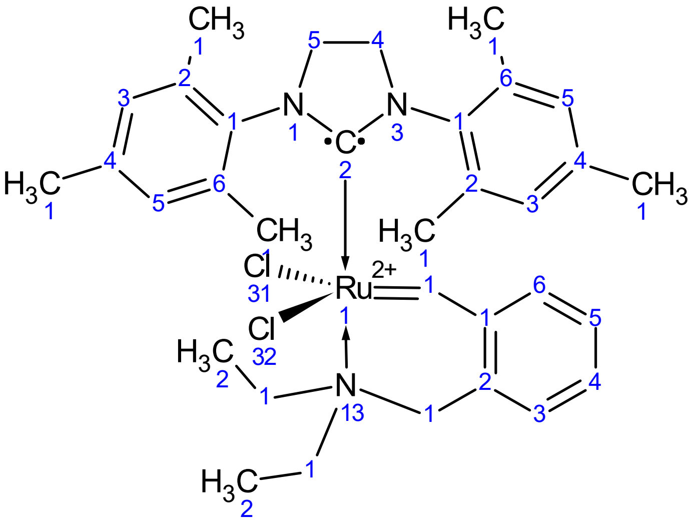


11b

| | | | | | |
|---|---|---|---|---|---|
| ***Acquisition Time (sec)*** | (0.0512, 0.0063) | ***Comment*** | 5 mm CPPBBO BB-1H/19F/D Z-GRD Z125869/0025 | | |
| ***Date*** | 21 Feb 2017 11:02:14 | | | | |
| ***File Name*** | C:\Users\Fedor\Desktop\Наброски Статей\Кирилл Статья по Катализаторам Граббса\ЯМР Граббс\Kirill-NEW1 Видно слабое поле\kp2102-2017-K1-NEt2\12\pdata\1\2rr | | | | |
| ***Frequency (MHz)*** | (500.14, 125.78) | ***Nucleus*** | (1H, 13C) | ***Number of Transients*** | 4 |
| ***Origin*** | spect | ***Original Points Count*** | (512, 256) | ***Owner*** | nmrsu |
| ***Points Count*** | (1024, 1024) | ***Pulse Sequence*** | hmbcgplpndqf | ***Solvent*** | CD2Cl2 |
| ***Sweep Width (Hz)*** | (10000.00, 40322.58) | ***Temperature (degree C)*** | 24.970 | | |

| | | | |
|---|---|---|---|
| ***Formula*** | $C_{33}H_{43}Cl_2N_3Ru$ | ***FW*** | 653.6895 |

kp2102-2017-K1-NEt2.012.001.2rr.esp

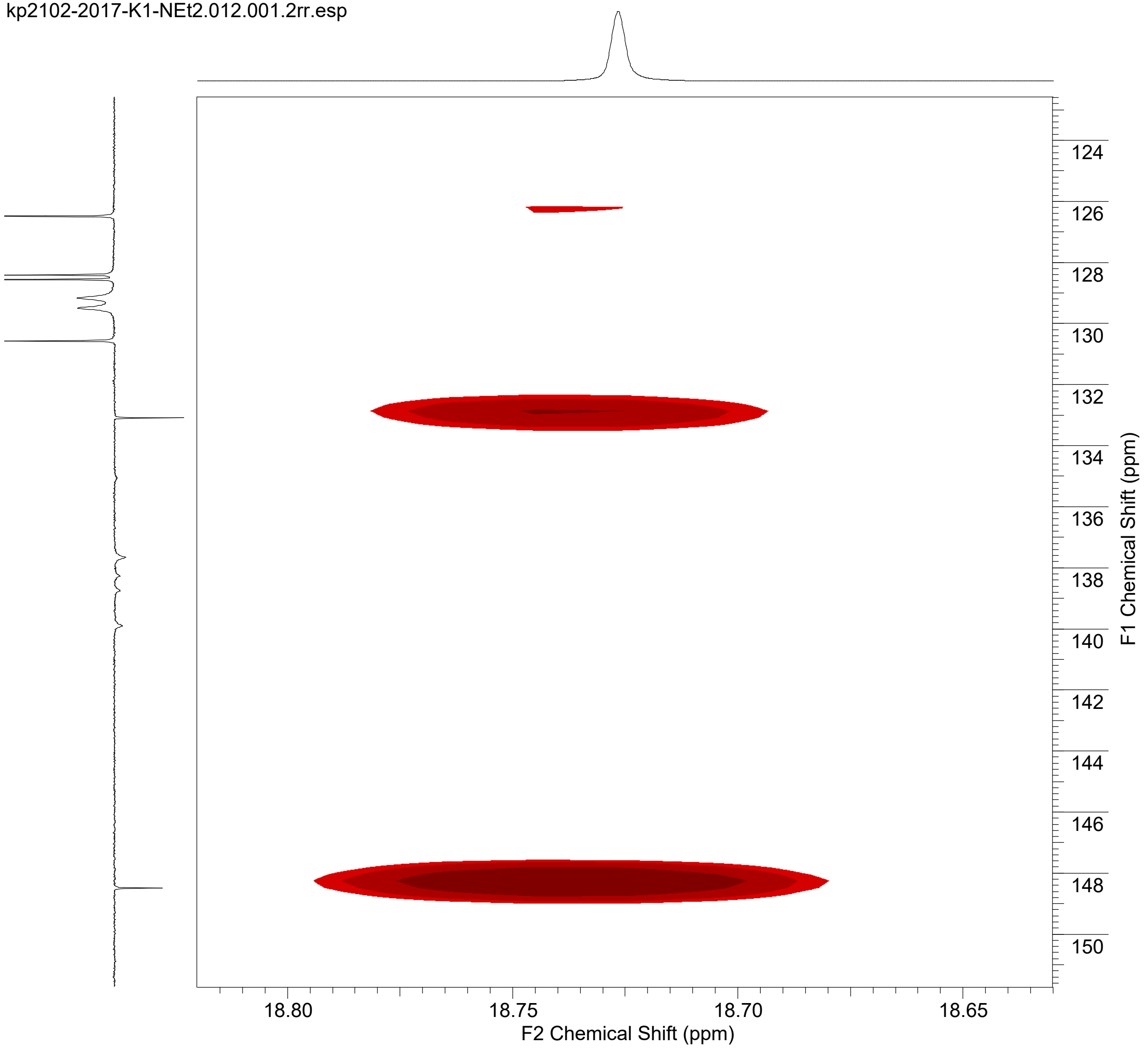


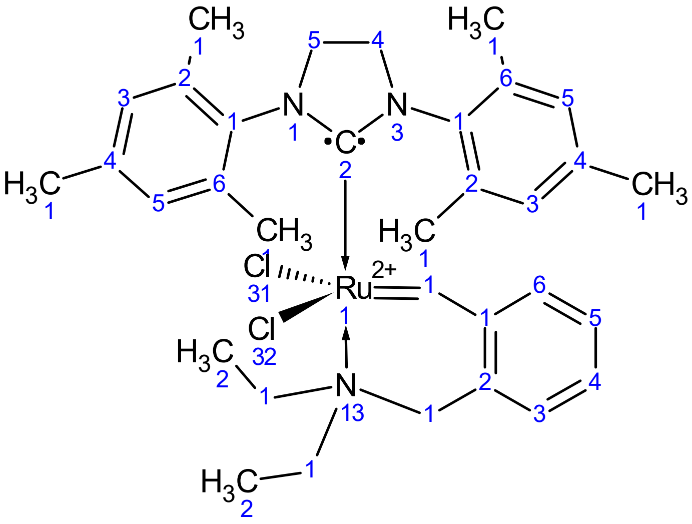


11b

| | | | | | |
|---|---|---|---|---|---|
| ***Acquisition Time (sec)*** | (0.0512, 0.0063) | ***Comment*** | 5 mm CPPBBO BB-1H/19F/D Z-GRD Z125869/0025 | | |
| ***Date*** | 21 Feb 2017 11:02:14 | | | | |
| ***File Name*** | C:\Users\Fedor\Desktop\Наброски Статей\Кирилл Статья по Катализаторам Граббса\ЯМР Граббс\Kirill-NEW1 Видно слабое поле\kp2102-2017-K1-NEt2\12\pdata\1\2rr | | | | |
| ***Frequency (MHz)*** | (500.14, 125.78) | ***Nucleus*** | (1H, 13C) | ***Number of Transients*** | 4 |
| ***Origin*** | spect | ***Original Points Count*** | (512, 256) | ***Owner*** | nmrsu |
| ***Points Count*** | (1024, 1024) | ***Pulse Sequence*** | hmbcgplpndqf | ***Solvent*** | CD2Cl2 |
| ***Sweep Width (Hz)*** | (10000.00, 40322.58) | ***Temperature (degree C)*** | 24.970 | | |

| | |
|---|---|
| ***Formula*** $C_{33}H_{43}Cl_2N_3Ru$ | ***FW*** 653.6895 |

kp2102-2017-K1-NEt2.012.001.2rr.esp

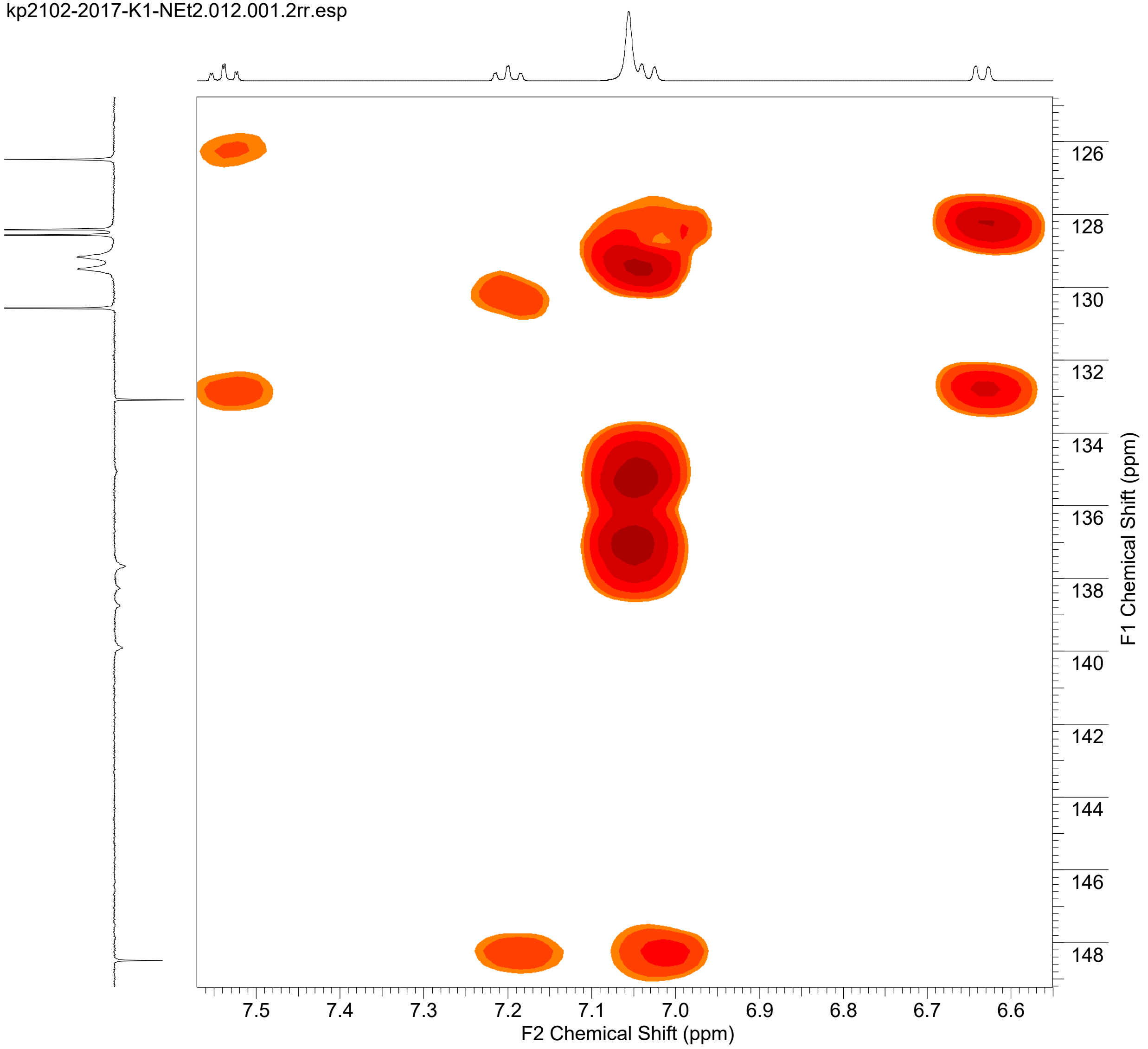


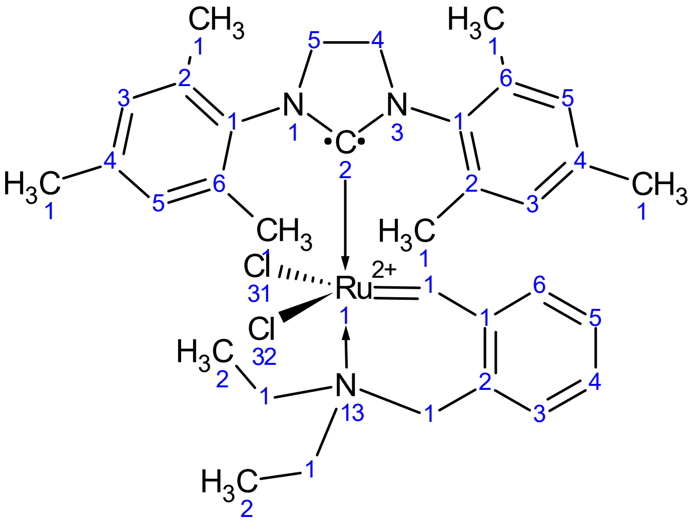


11b

| | | | | | |
|---|---|---|---|---|---|
| ***Acquisition Time (sec)*** | (0.0512, 0.0063) | ***Comment*** | 5 mm CPPBBO BB-1H/19F/D Z-GRD Z125869/0025 | | |
| ***Date*** | 21 Feb 2017 11:02:14 | | | | |
| ***File Name*** | C:\Users\Fedor\Desktop\Наброски Статей\Кирилл Статья по Катализаторам Граббса\ЯМР Граббс\Kirill-NEW1 Видно слабое поле\kp2102-2017-K1-NEt2\12\pdata\1\2rr | | | | |
| ***Frequency (MHz)*** | (500.14, 125.78) | ***Nucleus*** | (1H, 13C) | ***Number of Transients*** | 4 |
| ***Origin*** | spect | ***Original Points Count*** | (512, 256) | ***Owner*** | nmrsu |
| ***Points Count*** | (1024, 1024) | ***Pulse Sequence*** | hmbcgplpndqf | ***Solvent*** | CD2Cl2 |
| ***Sweep Width (Hz)*** | (10000.00, 40322.58) | ***Temperature (degree C)*** | 24.970 | | |

| | | | |
|---|---|---|---|
| ***Formula*** | $C_{33}H_{43}Cl_2N_3Ru$ | ***FW*** | 653.6895 |

kp2102-2017-K1-NEt2.012.001.2rr.esp

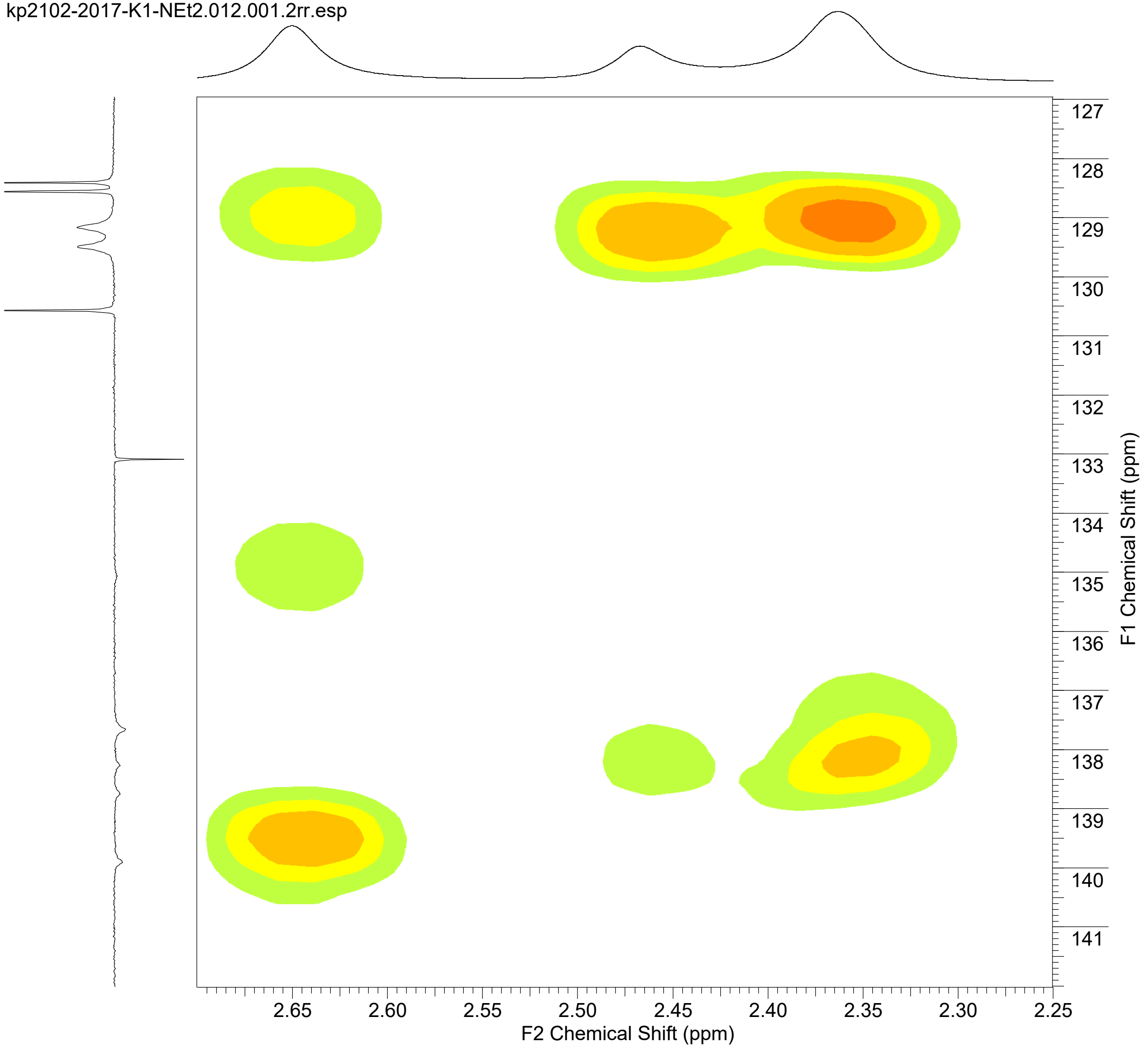


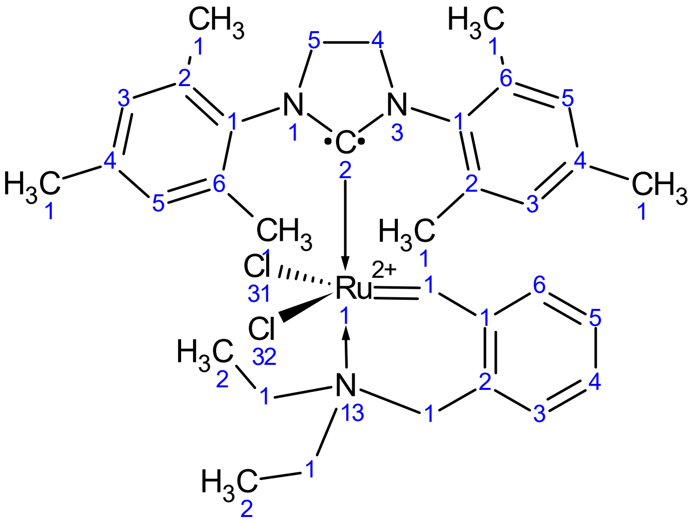


11b

| | | | | | |
|---|---|---|---|---|---|
| ***Acquisition Time (sec)*** | (0.0512, 0.0063) | ***Comment*** | 5 mm CPPBBO BB-1H/19F/D Z-GRD Z125869/0025 | | |
| ***Date*** | 21 Feb 2017 11:02:14 | | | | |
| ***File Name*** | C:\Users\Fedor\Desktop\Наброски Статей\Кирилл Статья по Катализаторам Граббса\ЯМР Граббс\Kirill-NEW1 Видно слабое поле\kp2102-2017-K1-NEt2\12\pdata\1\2rr | | | | |
| ***Frequency (MHz)*** | (500.14, 125.78) | ***Nucleus*** | (1H, 13C) | ***Number of Transients*** | 4 |
| ***Origin*** | spect | ***Original Points Count*** | (512, 256) | ***Owner*** | nmrsu |
| ***Points Count*** | (1024, 1024) | ***Pulse Sequence*** | hmbcgplpndqf | ***Solvent*** | CD2Cl2 |
| ***Sweep Width (Hz)*** | (10000.00, 40322.58) | ***Temperature (degree C)*** | 24.970 | | |

| | | | |
|---|---|---|---|
| ***Formula*** | $C_{33}H_{43}Cl_2N_3Ru$ | ***FW*** | 653.6895 |

kp2102-2017-K1-NEt2.012.001.2rr.esp

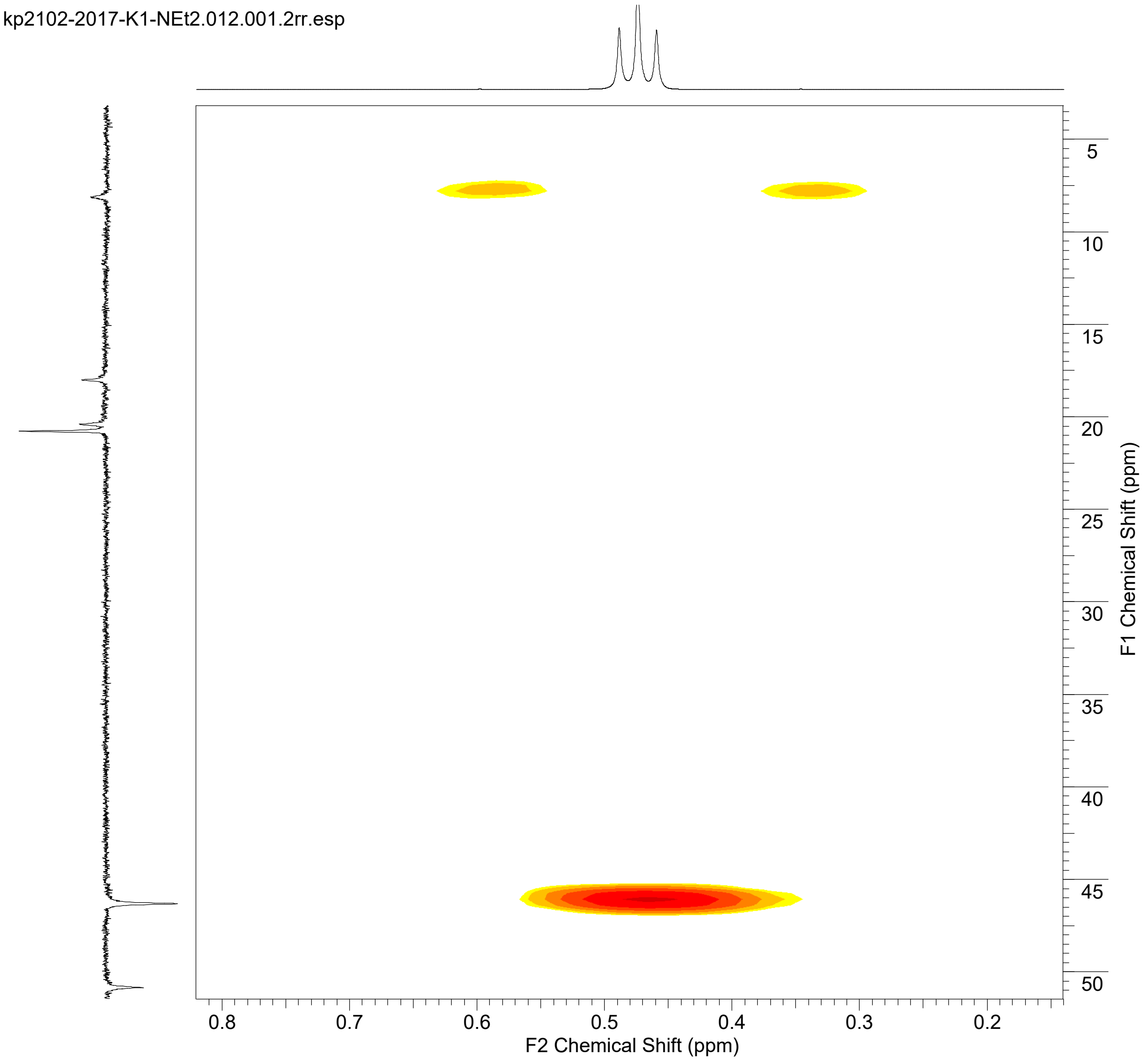


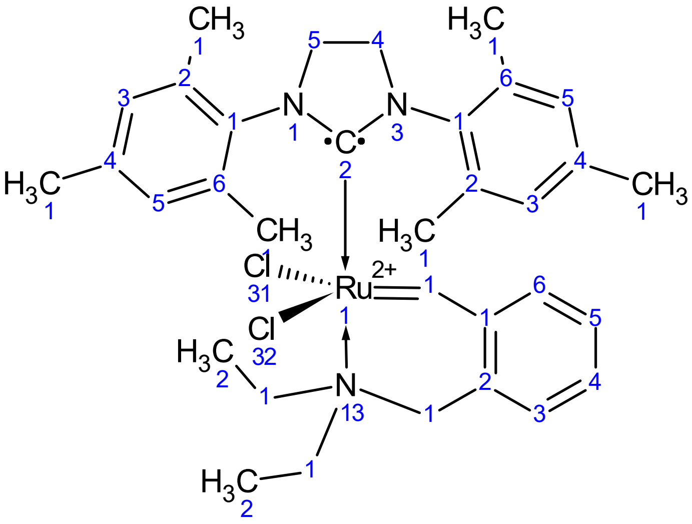

11b

| | | | | | | | | | |
|---|---|---|---|---|---|---|---|---|---|
| ***Acquisition Time (sec)*** | 1.9818 | ***Comment*** | single_pulse | ***Date*** | 19 Jan 1990 15:19:31 | | | ***Date Stamp*** | 07 Nov 2018 11:08:25 |
| ***File Name*** | C:\Users\Fedor\Desktop\07.11.18\FZ6955-1.jdf | | | ***Frequency (MHz)*** | 600.17 | ***Nucleus*** | 1H | ***Number of Transients*** | 8 |
| ***Origin*** | ECA 600 | ***Original Points Count*** | 32768 | ***Owner*** | delta | ***Points Count*** | 32768 | ***Pulse Sequence*** | single_pulse.ex2 |
| ***Receiver Gain*** | 38.00 | ***Solvent*** | CHLOROFORM-d | | | ***Spectrum Offset (Hz)*** | 5405.5811 | ***Sweep Width (Hz)*** | 16534.39 |

11b in CDCl3

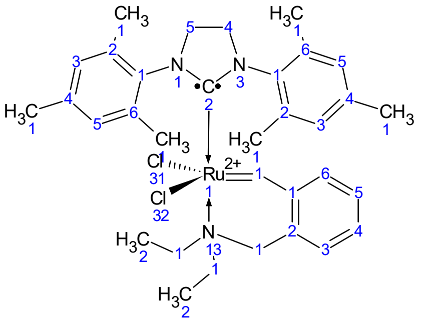

FZ6955-1.esp

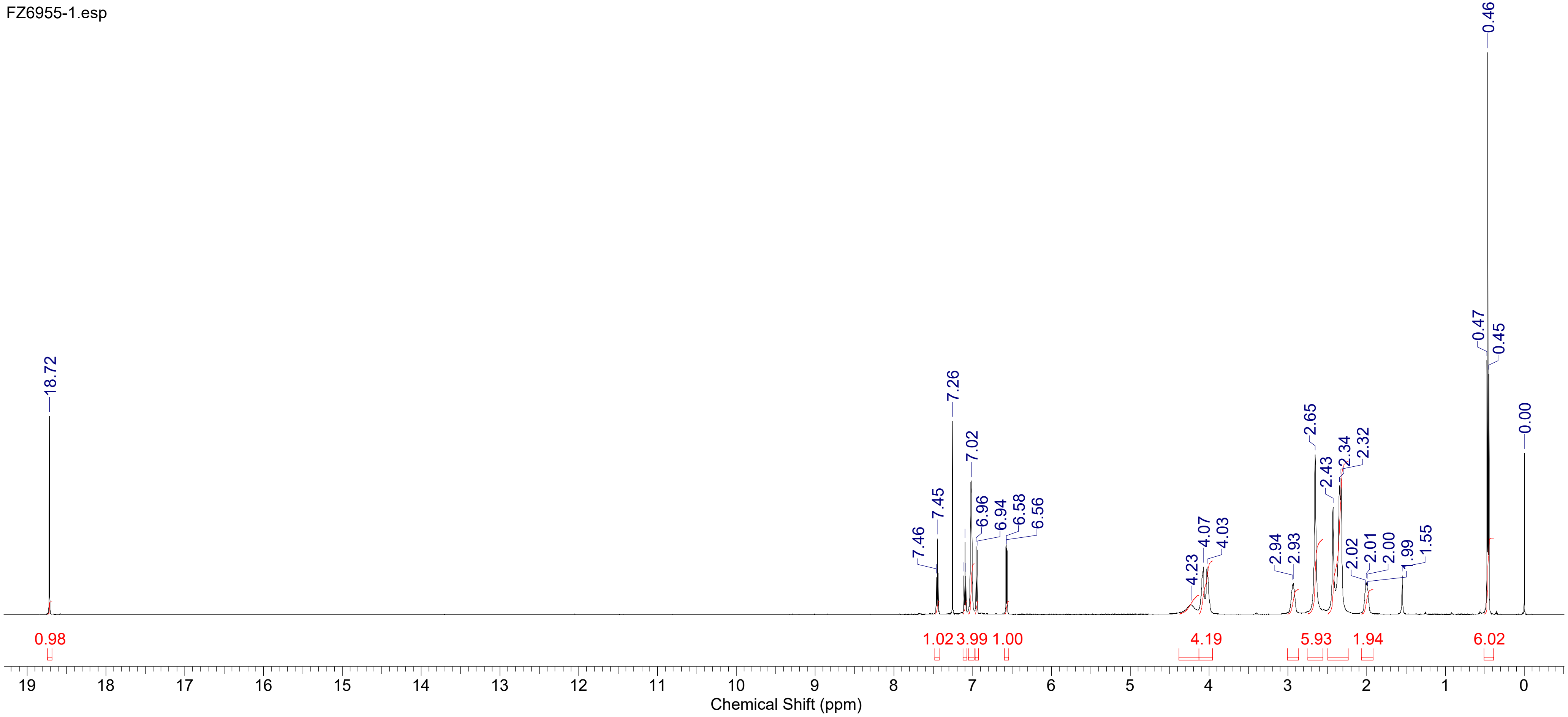

| Acquisition Time (sec) | 1.9818 | Comment | single_pulse | Date | 19 Jan 1990 15:19:31 | | | Date Stamp | 07 Nov 2018 11:08:25 |
|---|---|---|---|---|---|---|---|---|---|
| File Name | C:\Users\Fedor\Desktop\07.11.18\FZ6955-1.jdf | | | Frequency (MHz) | 600.17 | Nucleus | 1H | Number of Transients | 8 |
| Origin | ECA 600 | Original Points Count | 32768 | Owner | delta | Points Count | 32768 | Pulse Sequence | single_pulse.ex2 |
| Receiver Gain | 38.00 | Solvent | CHLOROFORM-d | | | Spectrum Offset (Hz) | 5405.5811 | Sweep Width (Hz) | 16534.39 |

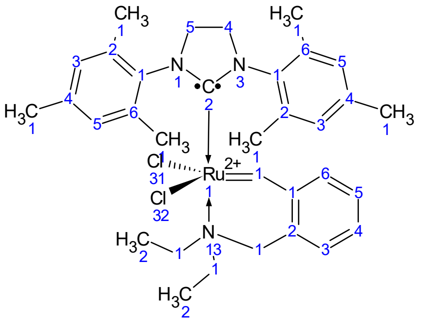

FZ6955-1.esp

11b in CDCl3

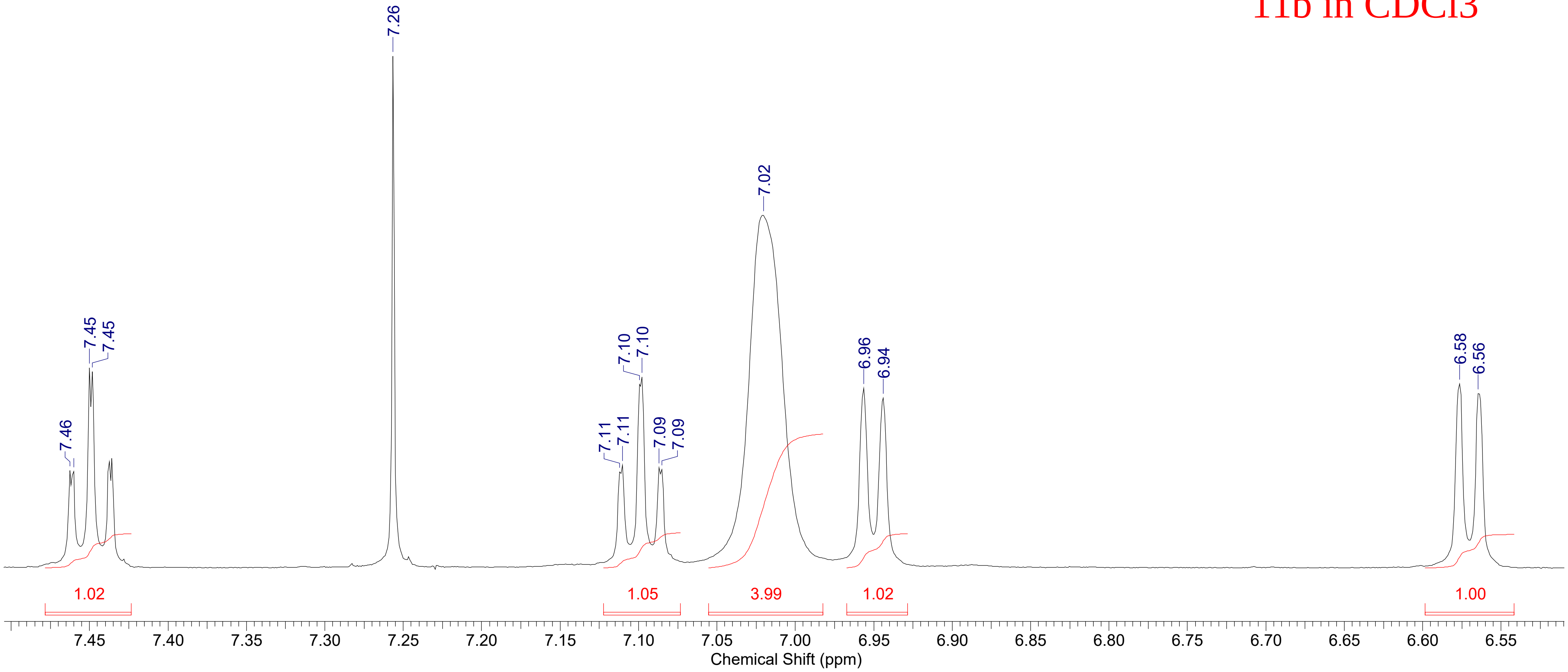

| | | | | | | | |
|---|---|---|---|---|---|---|---|
| ***Acquisition Time (sec)*** | 1.9818 | ***Comment*** | single_pulse | ***Date*** | 19 Jan 1990 15:19:31 | ***Date Stamp*** | 07 Nov 2018 11:08:25 |
| ***File Name*** | C:\Users\Fedor\Desktop\07.11.18\FZ6955-1.jdf | | | ***Frequency (MHz)*** | 600.17 | ***Nucleus*** | 1H |
| ***Number of Transients*** | 8 | ***Origin*** | ECA 600 | ***Original Points Count*** | 32768 | ***Owner*** | delta |
| ***Points Count*** | 32768 | ***Pulse Sequence*** | single_pulse.ex2 | ***Receiver Gain*** | 38.00 | ***Solvent*** | CHLOROFORM-d |
| ***Spectrum Offset (Hz)*** | 5405.5811 | ***Sweep Width (Hz)*** | 16534.39 | | | | |

11b in CDCl3

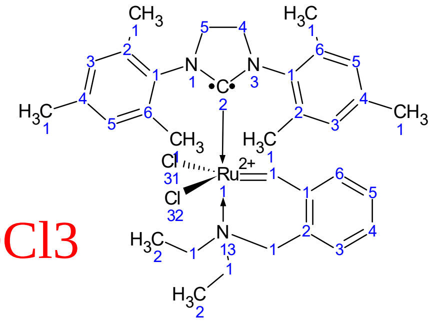

FZ6955-1.esp

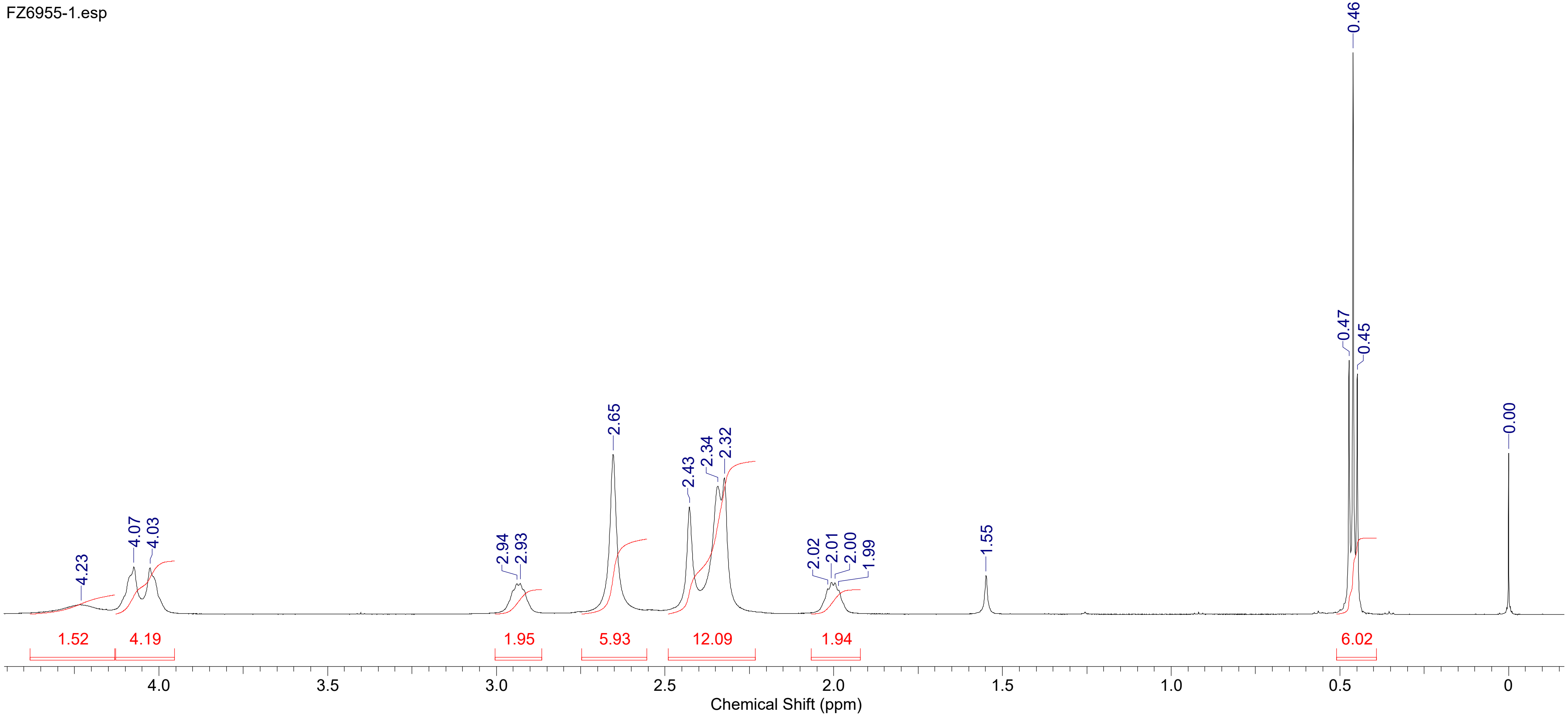

| | | | | | | | |
|---|---|---|---|---|---|---|---|
| ***Acquisition Time (sec)*** | 2.5690 | ***Comment*** | H1 | ***Date*** | 02 Oct 2018 18:46:24 | | |
| ***Date Stamp*** | 02 Oct 2018 18:46:24 | | | | | | |
| ***File Name*** | C:\Users\Fedor\Desktop\Наброски Статей\Кирилл Статья по Катализаторам Граббса\ЯМР Граббс\ЯМР Граббс от Ромы\FZ_Grubbs_1 NHMe_002001r | | | | | | |
| ***Frequency (MHz)*** | 300.13 | ***Nucleus*** | 1H | ***Number of Transients*** | 32 | ***Origin*** | spect |
| ***Original Points Count*** | 65536 | ***Owner*** | nmr | ***Points Count*** | 262144 | ***Pulse Sequence*** | zg |
| ***Receiver Gain*** | 72.88 | ***SW(cyclical) (Hz)*** | 25510.20 | ***Solvent*** | DICHLOROMETHANE-d2 | | |
| ***Spectrum Offset (Hz)*** | 1350.5919 | ***Sweep Width (Hz)*** | 25510.11 | ***Temperature (degree C)*** | 30.010 | | |

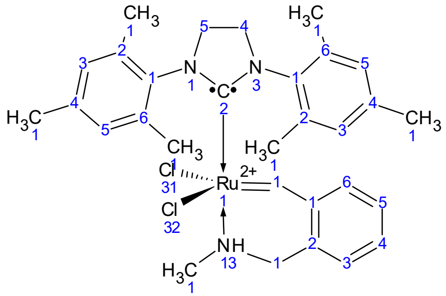

FZ_Grubbs_1 NHMe_002001r

11c

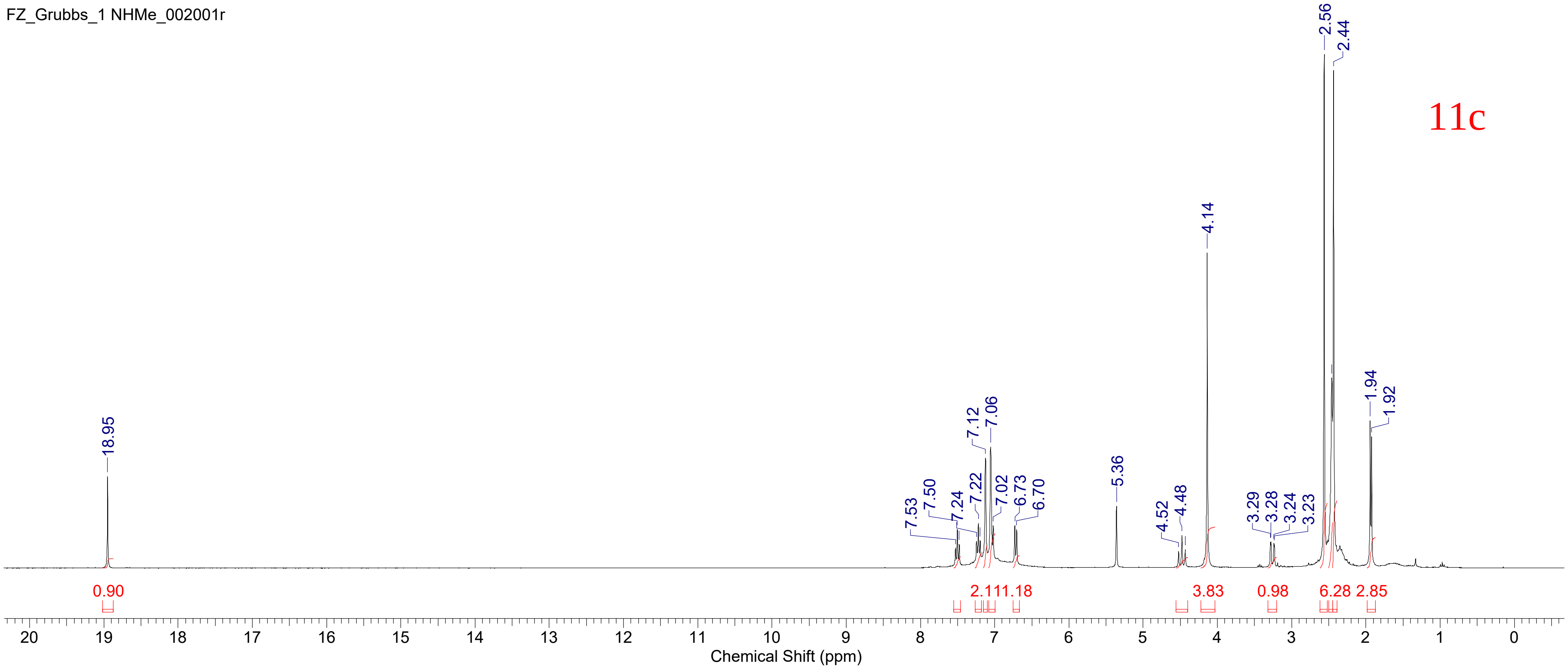

| | | | | | | | |
|---|---|---|---|---|---|---|---|
| ***Acquisition Time (sec)*** | 2.5690 | ***Comment*** | H1 | ***Date*** | 02 Oct 2018 18:46:24 | | |
| ***Date Stamp*** | 02 Oct 2018 18:46:24 | | | | | | |
| ***File Name*** | C:\Users\Fedor\Desktop\Наброски Статей\Кирилл Статья по Катализаторам Граббса\ЯМР Граббс\ЯМР Граббс от Ромы\FZ_Grubbs_1 NHMe_002001r | | | | | | |
| ***Frequency (MHz)*** | 300.13 | ***Nucleus*** | 1H | ***Number of Transients*** | 32 | ***Origin*** | spect |
| ***Original Points Count*** | 65536 | ***Owner*** | nmr | ***Points Count*** | 262144 | ***Pulse Sequence*** | zg |
| ***Receiver Gain*** | 72.88 | ***SW(cyclical) (Hz)*** | 25510.20 | ***Solvent*** | DICHLOROMETHANE-d2 | | |
| ***Spectrum Offset (Hz)*** | 1350.5919 | ***Sweep Width (Hz)*** | 25510.11 | ***Temperature (degree C)*** | 30.010 | | |

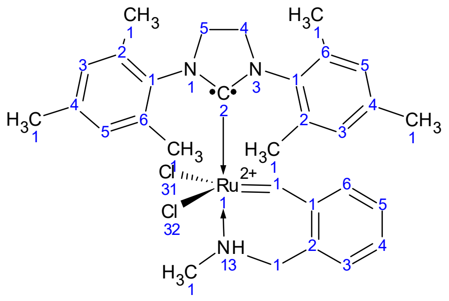

FZ_Grubbs_1 NHMe_002001r

11c

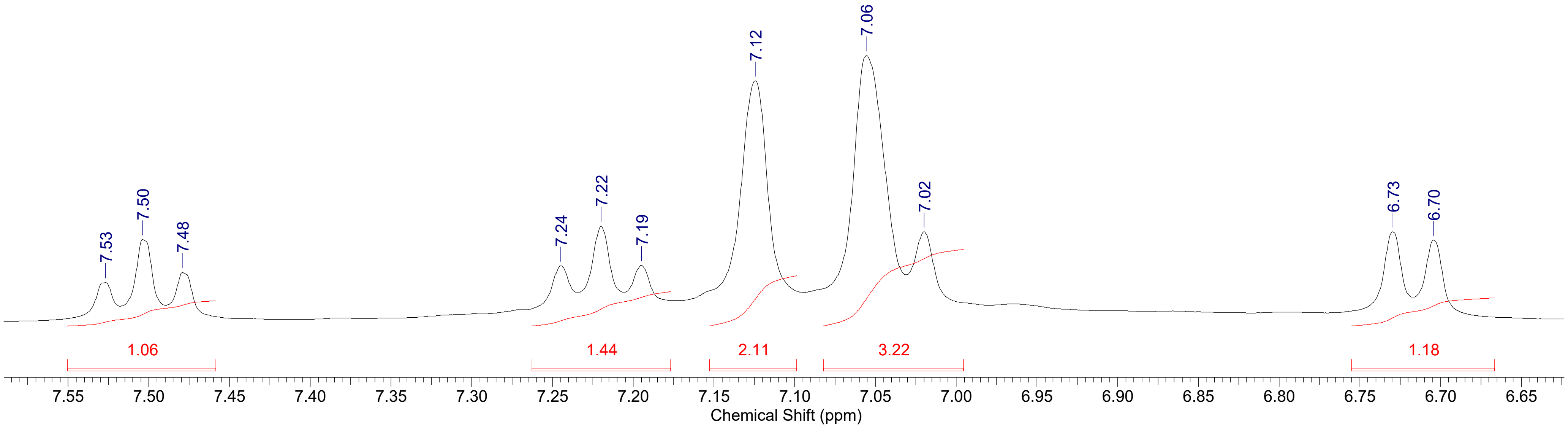

| | | | | | | | |
|---|---|---|---|---|---|---|---|
| **Acquisition Time (sec)** | 2.5690 | **Comment** | H1 | **Date** | 02 Oct 2018 18:46:24 | | |
| **Date Stamp** | 02 Oct 2018 18:46:24 | | | | | | |
| **File Name** | C:\Users\Fedor\Desktop\Наброски Статей\Кирилл Статья по Катализаторам Граббса\ЯМР Граббс\ЯМР Граббс от Ромы\FZ_Grubbs_1 NHMe_002001r | | | | | | |
| **Frequency (MHz)** | 300.13 | **Nucleus** | 1H | **Number of Transients** | 32 | **Origin** | spect |
| **Original Points Count** | 65536 | **Owner** | nmr | **Points Count** | 262144 | **Pulse Sequence** | zg |
| **Receiver Gain** | 72.88 | **SW(cyclical) (Hz)** | 25510.20 | **Solvent** | DICHLOROMETHANE-d2 | | |
| **Spectrum Offset (Hz)** | 1350.5919 | **Sweep Width (Hz)** | 25510.11 | **Temperature (degree C)** | 30.010 | | |

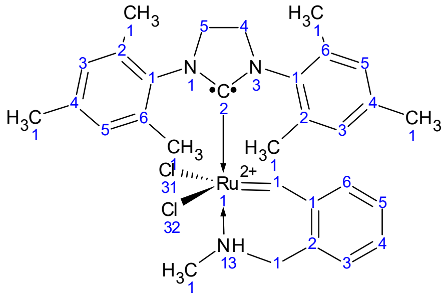

11c

FZ_Grubbs_1 NHMe_002001r

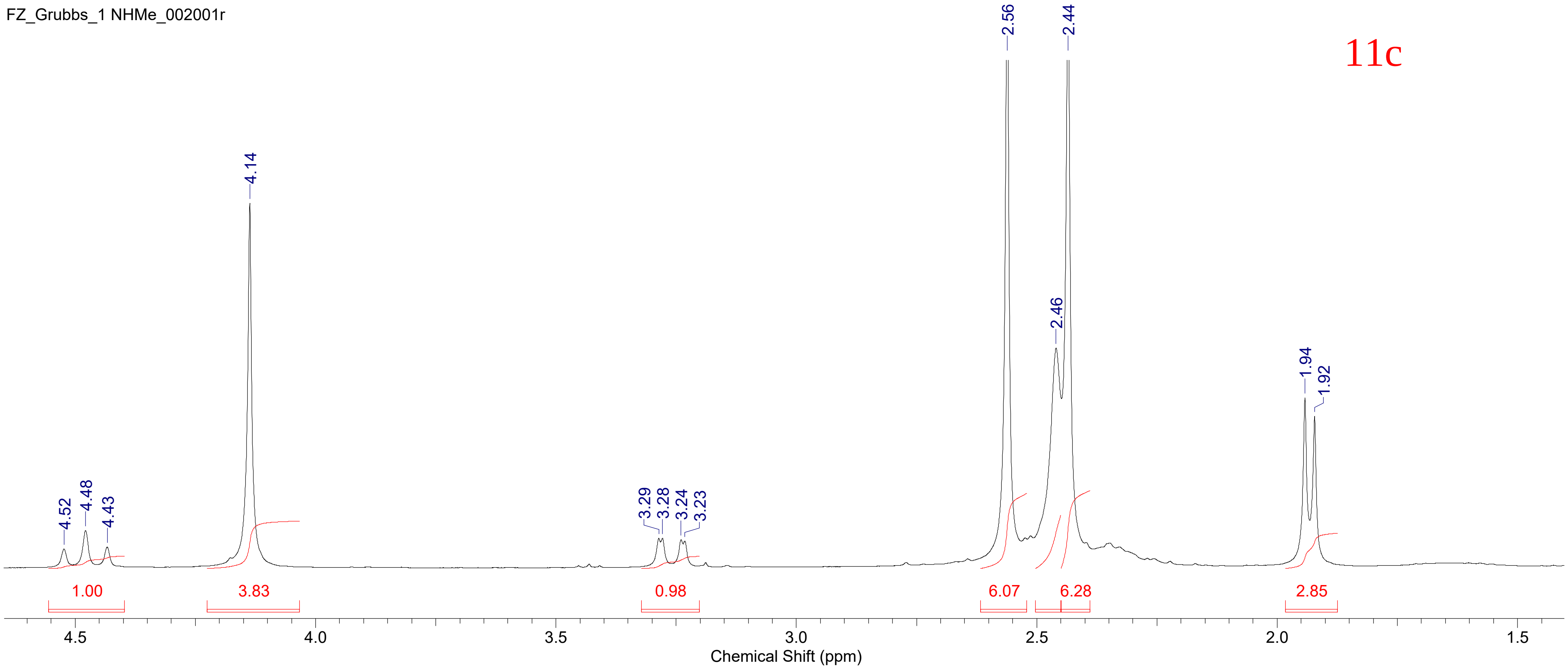

| | | | | | | | |
|---|---|---|---|---|---|---|---|
| ***Acquisition Time (sec)*** | 1.1010 | ***Comment*** | FZ_Grubbs_1-K4-C13dec [hp-dec] | | | ***Date*** | 02 Oct 2018 17:53:04 |
| ***Date Stamp*** | 02 Oct 2018 17:53:04 | | | | | | |
| ***File Name*** | C:\Users\Fedor\Desktop\Наброски Статей\Кирилл Статья по Катализаторам Граббса\ЯМР Граббс\ЯМР Граббс от Ромы\FZ_Grubbs_1 NHMe_013001r | | | | | | |
| ***Frequency (MHz)*** | 75.47 | ***Nucleus*** | 13C | ***Number of Transients*** | 584 | ***Origin*** | spect |
| ***Original Points Count*** | 65536 | ***Owner*** | nmr | ***Points Count*** | 262144 | ***Pulse Sequence*** | zgpg |
| ***Receiver Gain*** | 202.48 | ***SW(cyclical) (Hz)*** | 59523.81 | ***Solvent*** | DICHLOROMETHANE-d2 | | |
| ***Spectrum Offset (Hz)*** | 7546.7783 | ***Sweep Width (Hz)*** | 59523.58 | ***Temperature (degree C)*** | 30.011 | | |

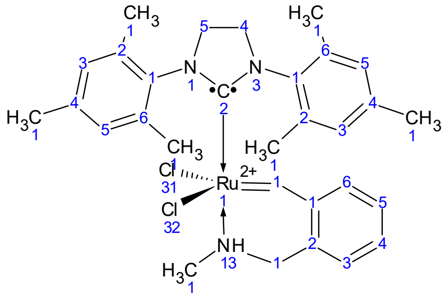

FZ_Grubbs_1 NHMe_013001r

11c

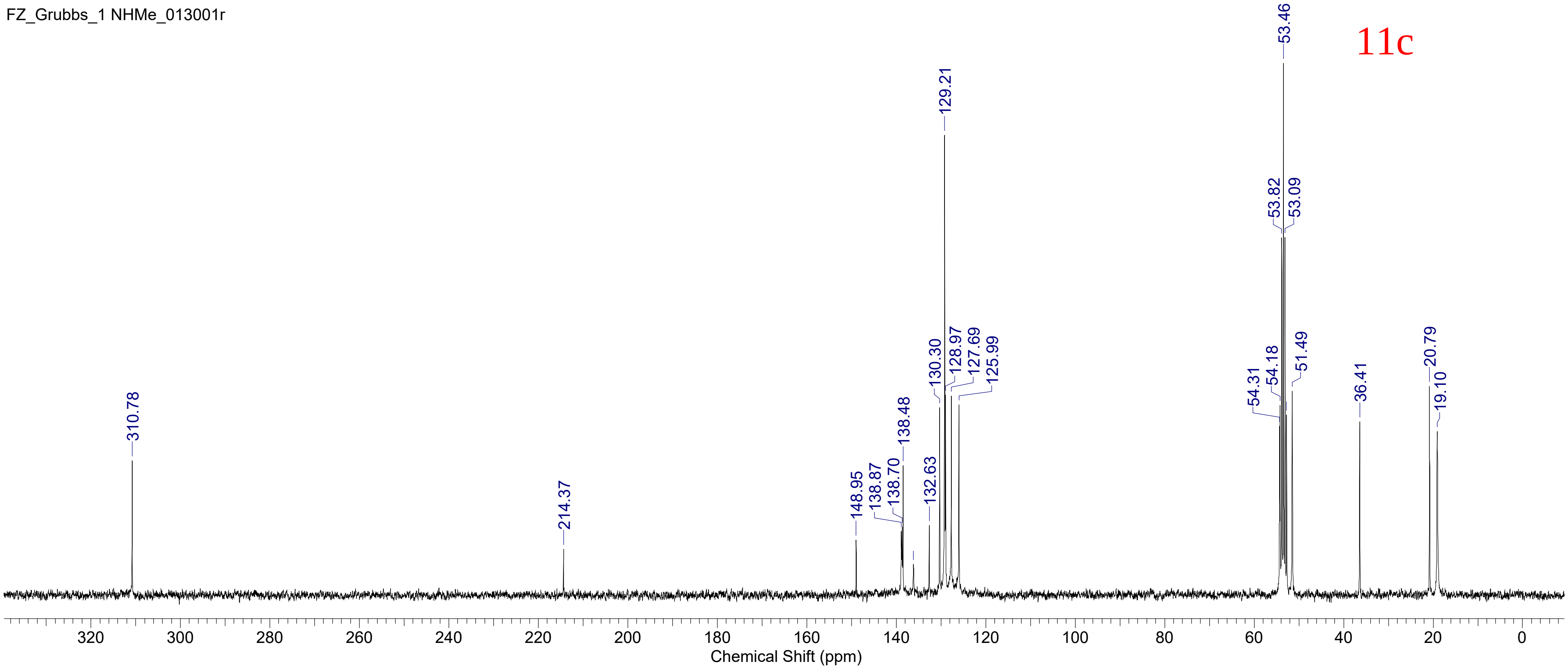

| | | | | | | | |
|---|---|---|---|---|---|---|---|
| ***Acquisition Time (sec)*** | 1.1010 | ***Comment*** | FZ_Grubbs_1-K4-C13dec [hp-dec] | | | ***Date*** | 02 Oct 2018 17:53:04 |
| ***Date Stamp*** | 02 Oct 2018 17:53:04 | | | | | | |
| ***File Name*** | C:\Users\Fedor\Desktop\Наброски Статей\Кирилл Статья по Катализаторам Граббса\ЯМР Граббс\ЯМР Граббс от Ромы\FZ_Grubbs_1 NHMe_013001r | | | | | | |
| ***Frequency (MHz)*** | 75.47 | ***Nucleus*** | 13C | ***Number of Transients*** | 584 | ***Origin*** | spect |
| ***Original Points Count*** | 65536 | ***Owner*** | nmr | ***Points Count*** | 262144 | ***Pulse Sequence*** | zgpg |
| ***Receiver Gain*** | 202.48 | ***SW(cyclical) (Hz)*** | 59523.81 | ***Solvent*** | DICHLOROMETHANE-d2 | | |
| ***Spectrum Offset (Hz)*** | 7546.7783 | ***Sweep Width (Hz)*** | 59523.58 | ***Temperature (degree C)*** | 30.011 | | |

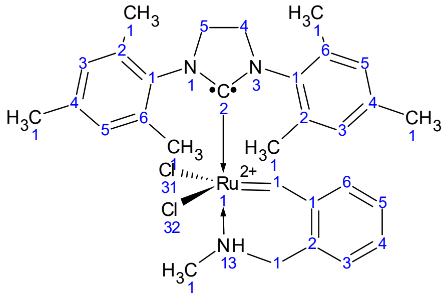

FZ_Grubbs_1 NHMe_013001r

11c

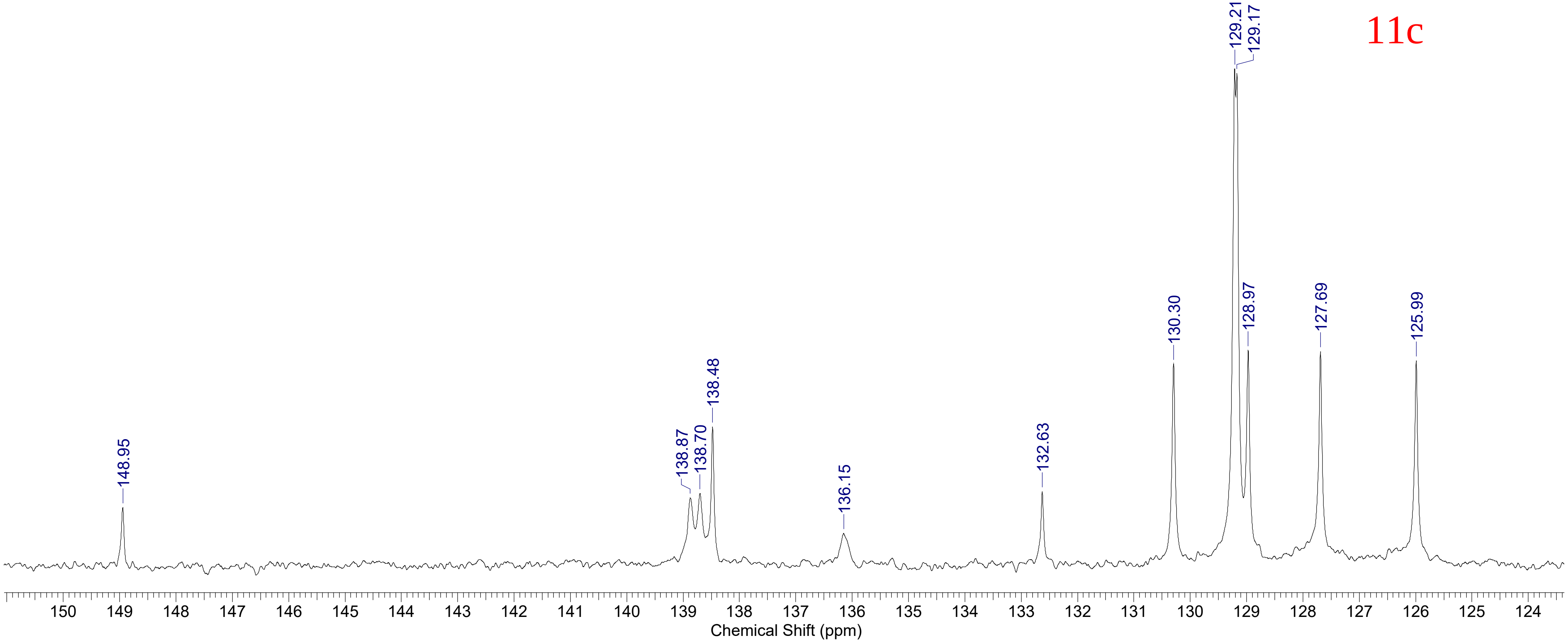

| | | | | | | | |
|---|---|---|---|---|---|---|---|
| ***Acquisition Time (sec)*** | 1.1010 | ***Comment*** | FZ_Grubbs_1-K4-C13dec [hp-dec] | | | ***Date*** | 02 Oct 2018 17:53:04 |
| ***Date Stamp*** | 02 Oct 2018 17:53:04 | | | | | | |
| ***File Name*** | C:\Users\Fedor\Desktop\Наброски Статей\Кирилл Статья по Катализаторам Граббса\ЯМР Граббс\ЯМР Граббс от Ромы\FZ_Grubbs_1 NHMe_013001r | | | | | | |
| ***Frequency (MHz)*** | 75.47 | ***Nucleus*** | 13C | ***Number of Transients*** | 584 | ***Origin*** | spect |
| ***Original Points Count*** | 65536 | ***Owner*** | nmr | ***Points Count*** | 262144 | ***Pulse Sequence*** | zgpg |
| ***Receiver Gain*** | 202.48 | ***SW(cyclical) (Hz)*** | 59523.81 | ***Solvent*** | DICHLOROMETHANE-d2 | | |
| ***Spectrum Offset (Hz)*** | 7546.7783 | ***Sweep Width (Hz)*** | 59523.58 | ***Temperature (degree C)*** | 30.011 | | |

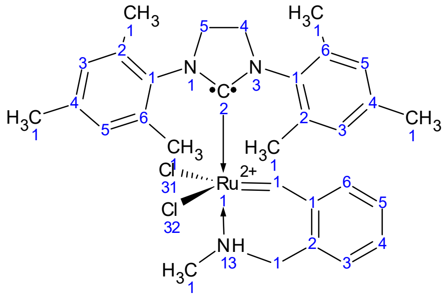

11c

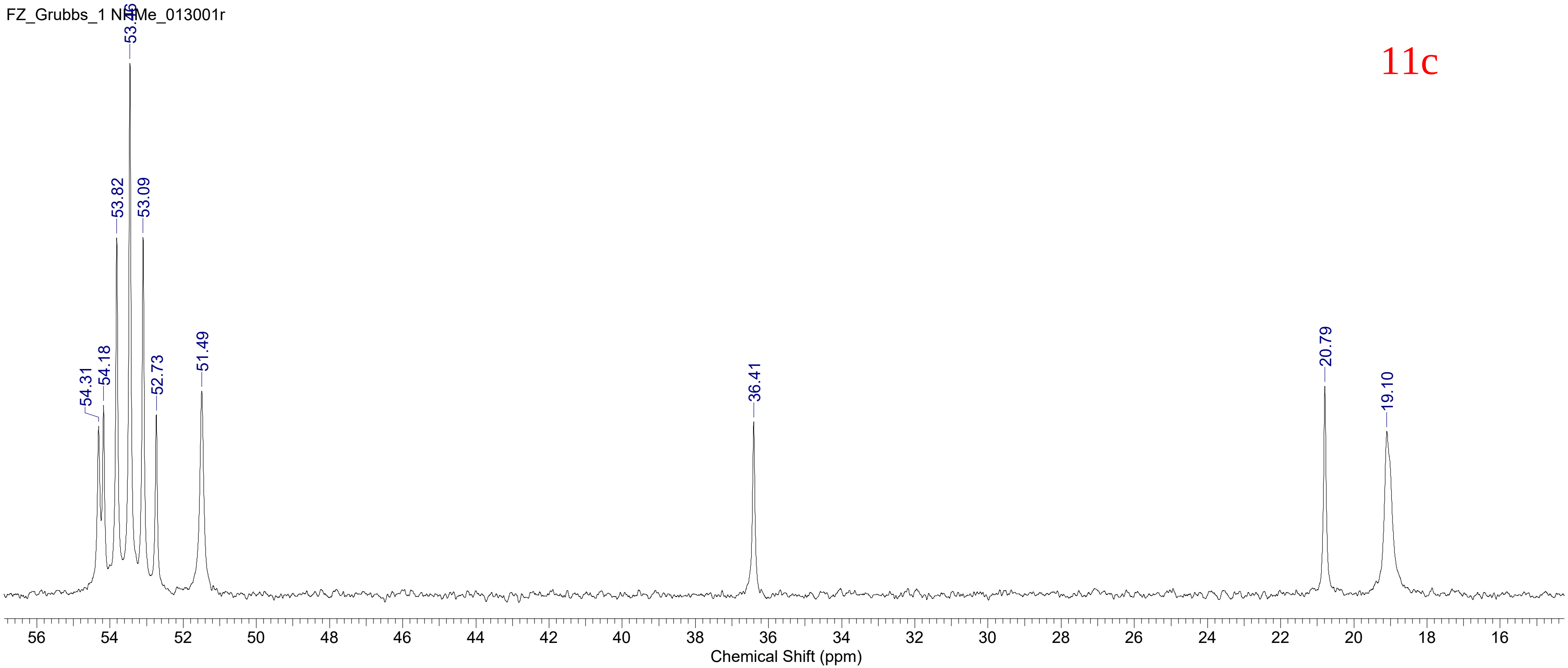

| | | | | | | | |
|---|---|---|---|---|---|---|---|
| ***Acquisition Time (sec)*** | 1.1010 | ***Comment*** | FZ_Grubbs_1-K4-dept135 [hp-dec] | | | ***Date*** | 02 Oct 2018 18:18:40 |
| ***Date Stamp*** | 02 Oct 2018 18:18:40 | | | | | | |
| ***File Name*** | C:\Users\Fedor\Desktop\Наброски Статей\Кирилл Статья по Катализаторам Граббса\ЯМР Граббс\ЯМР Граббс от Ромы\FZ_Grubbs_1 NHMe_015001r | | | | | | |
| ***Frequency (MHz)*** | 75.47 | ***Nucleus*** | 13C | ***Number of Transients*** | 184 | ***Origin*** | spect |
| ***Original Points Count*** | 65536 | ***Owner*** | nmr | ***Points Count*** | 262144 | ***Pulse Sequence*** | dept135 |
| ***Receiver Gain*** | 202.48 | ***SW(cyclical) (Hz)*** | 59523.81 | ***Solvent*** | DICHLOROMETHANE-d2 | | |
| ***Spectrum Offset (Hz)*** | 7546.7783 | ***Sweep Width (Hz)*** | 59523.58 | ***Temperature (degree C)*** | 30.012 | | |

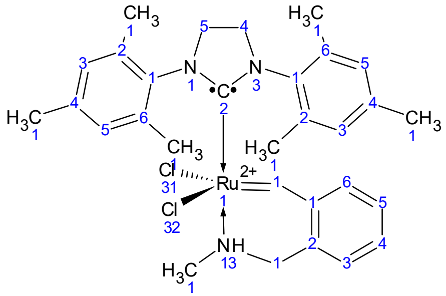

11c

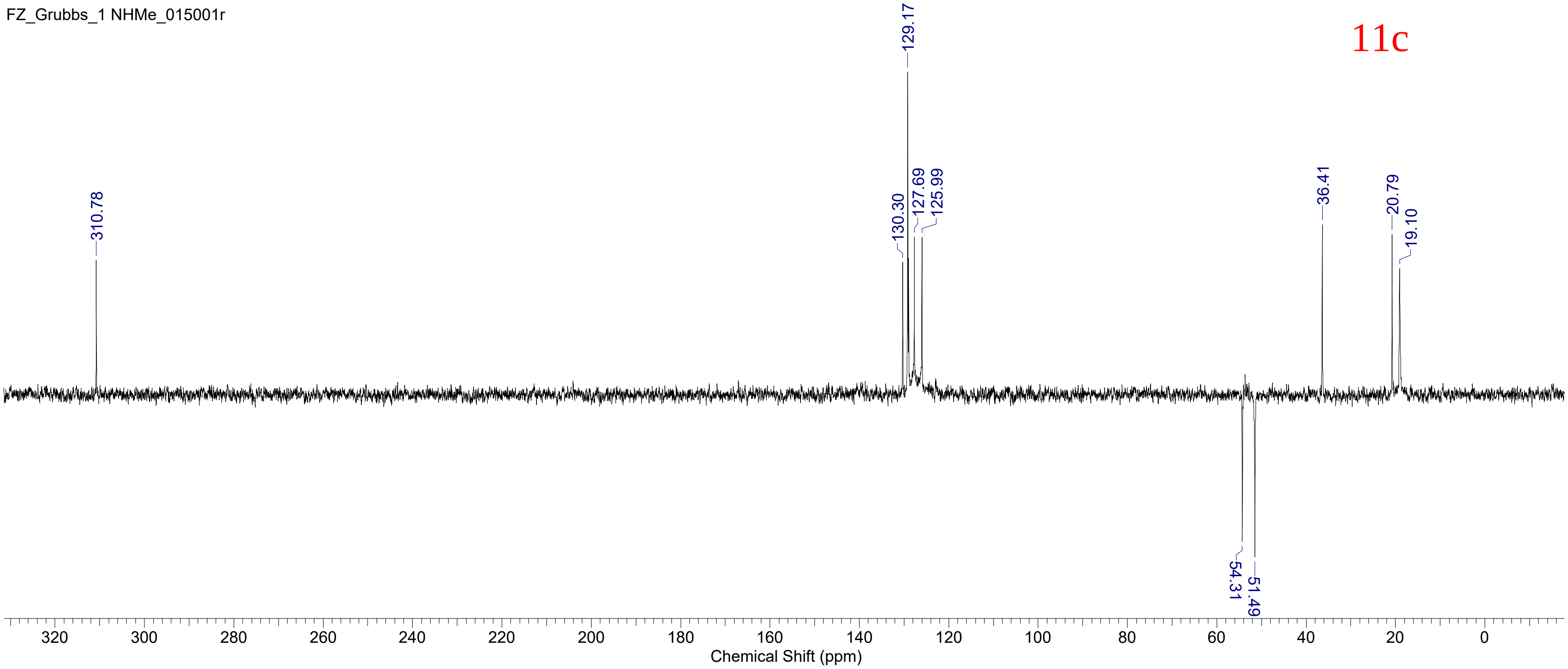

| Acquisition Time (sec) | 1.1010 | Comment | FZ_Grubbs_1-K4-dept135 [hp-dec] | | | Date | 02 Oct 2018 18:18:40 |
|---|---|---|---|---|---|---|---|
| Date Stamp | 02 Oct 2018 18:18:40 | | | | | | |
| File Name | C:\Users\Fedor\Desktop\Наброски Статей\Кирилл Статья по Катализаторам Граббса\ЯМР Граббс\ЯМР Граббс от Ромы\FZ_Grubbs_1 NHMe_015001r | | | | | | |
| Frequency (MHz) | 75.47 | Nucleus | 13C | Number of Transients | 184 | Origin | spect |
| Original Points Count | 65536 | Owner | nmr | Points Count | 262144 | Pulse Sequence | dept135 |
| Receiver Gain | 202.48 | SW(cyclical) (Hz) | 59523.81 | Solvent | DICHLOROMETHANE-d2 | | |
| Spectrum Offset (Hz) | 7546.7783 | Sweep Width (Hz) | 59523.58 | Temperature (degree C) | 30.012 | | |

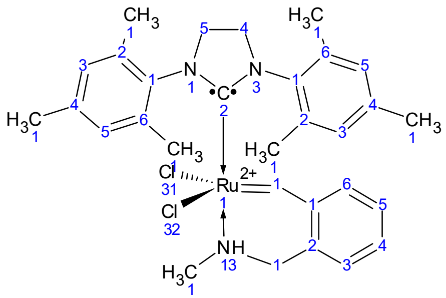

11c

FZ_Grubbs_1 NHMe_015001r

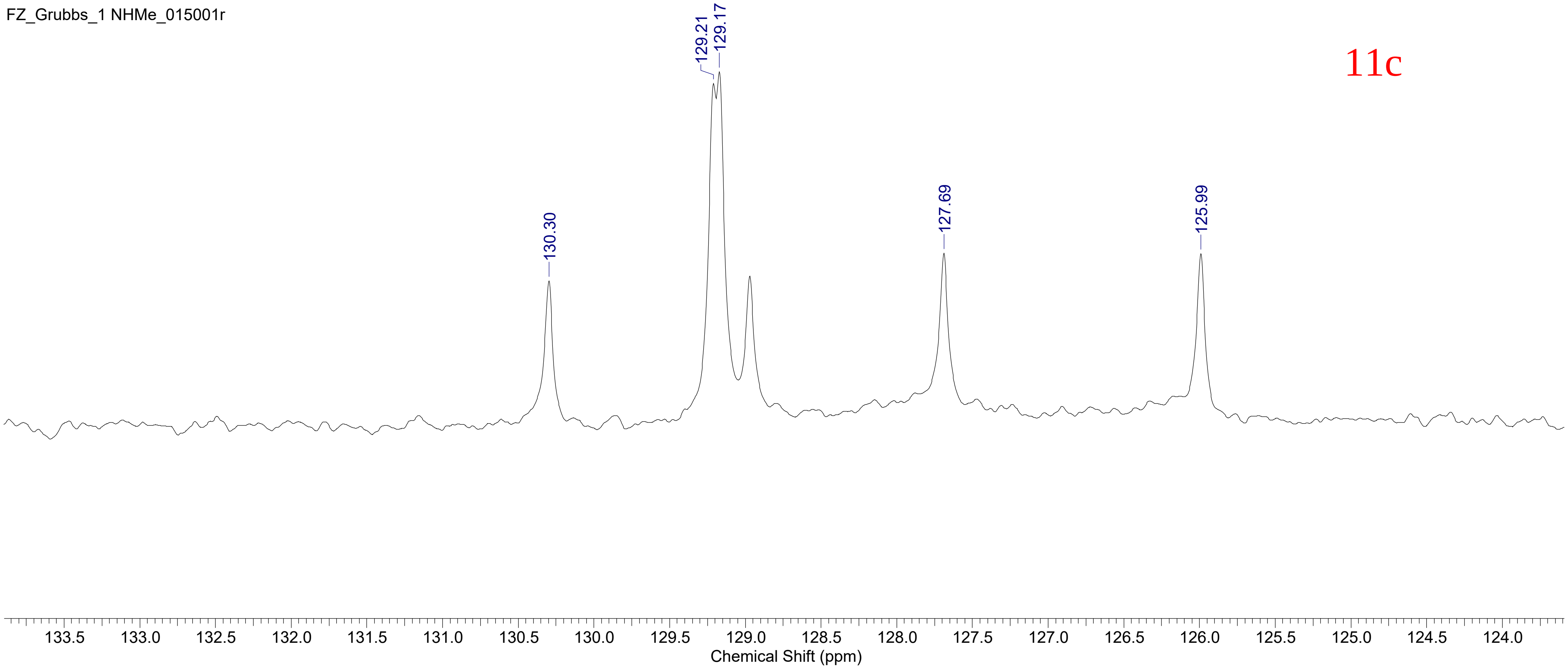

| | | | | | | | |
|---|---|---|---|---|---|---|---|
| ***Acquisition Time (sec)*** | 1.1010 | ***Comment*** | FZ_Grubbs_1-K4-dept135 [hp-dec] | | | ***Date*** | 02 Oct 2018 18:18:40 |
| ***Date Stamp*** | 02 Oct 2018 18:18:40 | | | | | | |
| ***File Name*** | C:\Users\Fedor\Desktop\Наброски Статей\Кирилл Статья по Катализаторам Граббса\ЯМР Граббс\ЯМР Граббс от Ромы\FZ_Grubbs_1 NHMe_015001r | | | | | | |
| ***Frequency (MHz)*** | 75.47 | ***Nucleus*** | 13C | ***Number of Transients*** | 184 | ***Origin*** | spect |
| ***Original Points Count*** | 65536 | ***Owner*** | nmr | ***Points Count*** | 262144 | ***Pulse Sequence*** | dept135 |
| ***Receiver Gain*** | 202.48 | ***SW(cyclical) (Hz)*** | 59523.81 | ***Solvent*** | DICHLOROMETHANE-d2 | | |
| ***Spectrum Offset (Hz)*** | 7546.7783 | ***Sweep Width (Hz)*** | 59523.58 | ***Temperature (degree C)*** | 30.012 | | |

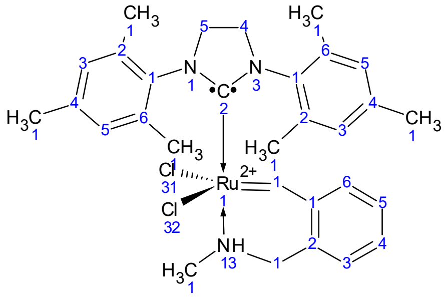

11c

FZ_Grubbs_1 NHMe_015001r

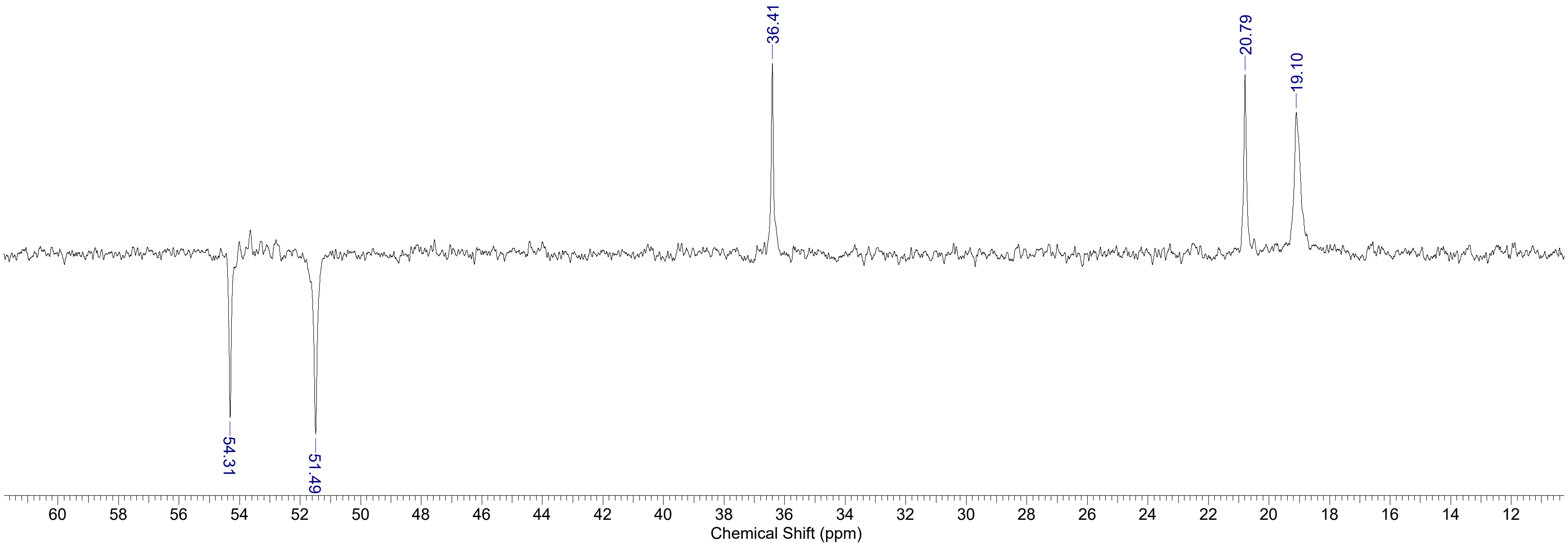

| Acquisition Time (sec) | (0.3408, 0.0069) | Comment | 5 mm PABBO BB-1H/D Z-GRD Z104275/0345 | | |
|---|---|---|---|---|---|
| Date | 02 Oct 2018 21:38:58 | | | | |
| File Name | C:\Users\Fedor\Desktop\Наброски Статей\Кирилл Статья по Катализаторам Граббса\ЯМР Граббс\ЯМР Граббс от Ромы\FZ_Grubbs_1 NHMe\113\pdata\1\2rr | | | | |
| Frequency (MHz) | (300.13, 75.48) | Nucleus | (1H, 13C) | Number of Transients | 2 |
| Origin | spect | Original Points Count | (3072, 233) | Owner | nmr |
| Points Count | (8192, 2048) | Pulse Sequence | hsqcetgp | Solvent | CD2Cl2 |
| Sweep Width (Hz) | (9014.42, 34013.61) | Temperature (degree C) | 29.985 | Title | FZ_Grubbs_1-K4-HSQC [hp-dec] |

| Formula | $C_{30}H_{37}Cl_2N_3Ru$ | FW | 611.6098 |
|---|---|---|---|

FZ_Grubbs_1 NHMe.113.001.2rr.esp

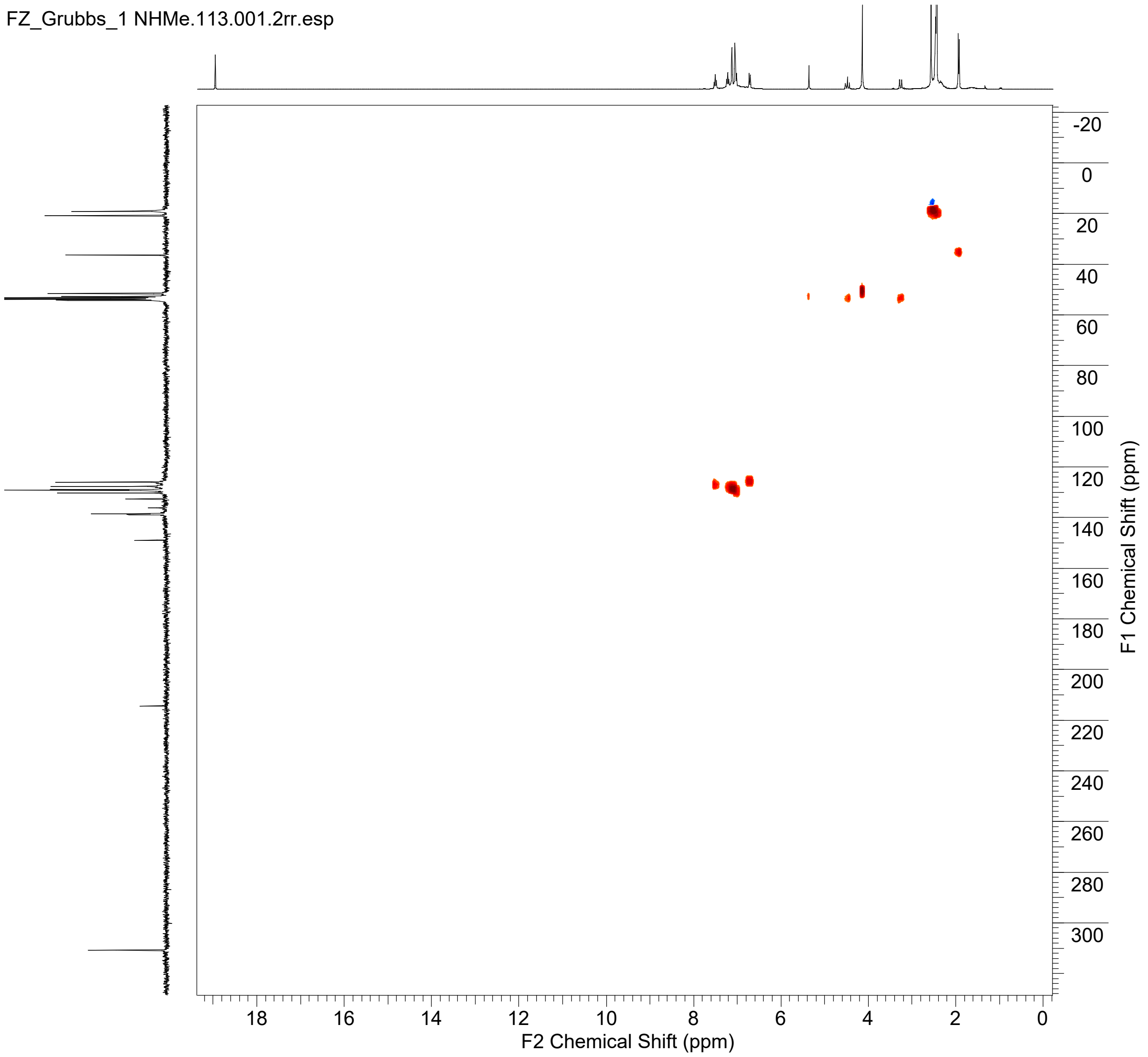


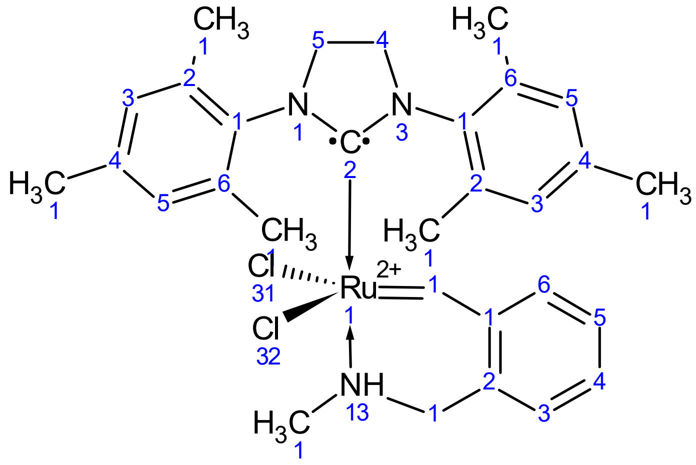

11c

| | | | | | |
|---|---|---|---|---|---|
| ***Acquisition Time (sec)*** | (0.3408, 0.0069) | ***Comment*** | 5 mm PABBO BB-1H/D Z-GRD Z104275/0345 | | |
| ***Date*** | 02 Oct 2018 21:38:58 | | | | |
| ***File Name*** | C:\Users\Fedor\Desktop\Наброски Статей\Кирилл Статья по Катализаторам Граббса\ЯМР Граббс\ЯМР Граббс от Ромы\FZ_Grubbs_1 NHMe\113\pdata\1\2rr | | | | |
| ***Frequency (MHz)*** | (300.13, 75.48) | ***Nucleus*** | (1H, 13C) | ***Number of Transients*** | 2 |
| ***Origin*** | spect | ***Original Points Count*** | (3072, 233) | ***Owner*** | nmr |
| ***Points Count*** | (8192, 2048) | ***Pulse Sequence*** | hsqcetgp | ***Solvent*** | CD2Cl2 |
| ***Sweep Width (Hz)*** | (9014.42, 34013.61) | ***Temperature (degree C)*** | 29.985 | ***Title*** | FZ_Grubbs_1-K4-HSQC [hp-dec] |

| | | | |
|---|---|---|---|
| ***Formula*** | $C_{30}H_{37}Cl_2N_3Ru$ | ***FW*** | 611.6098 |

FZ_Grubbs_1 NHMe.113.001.2rr.esp

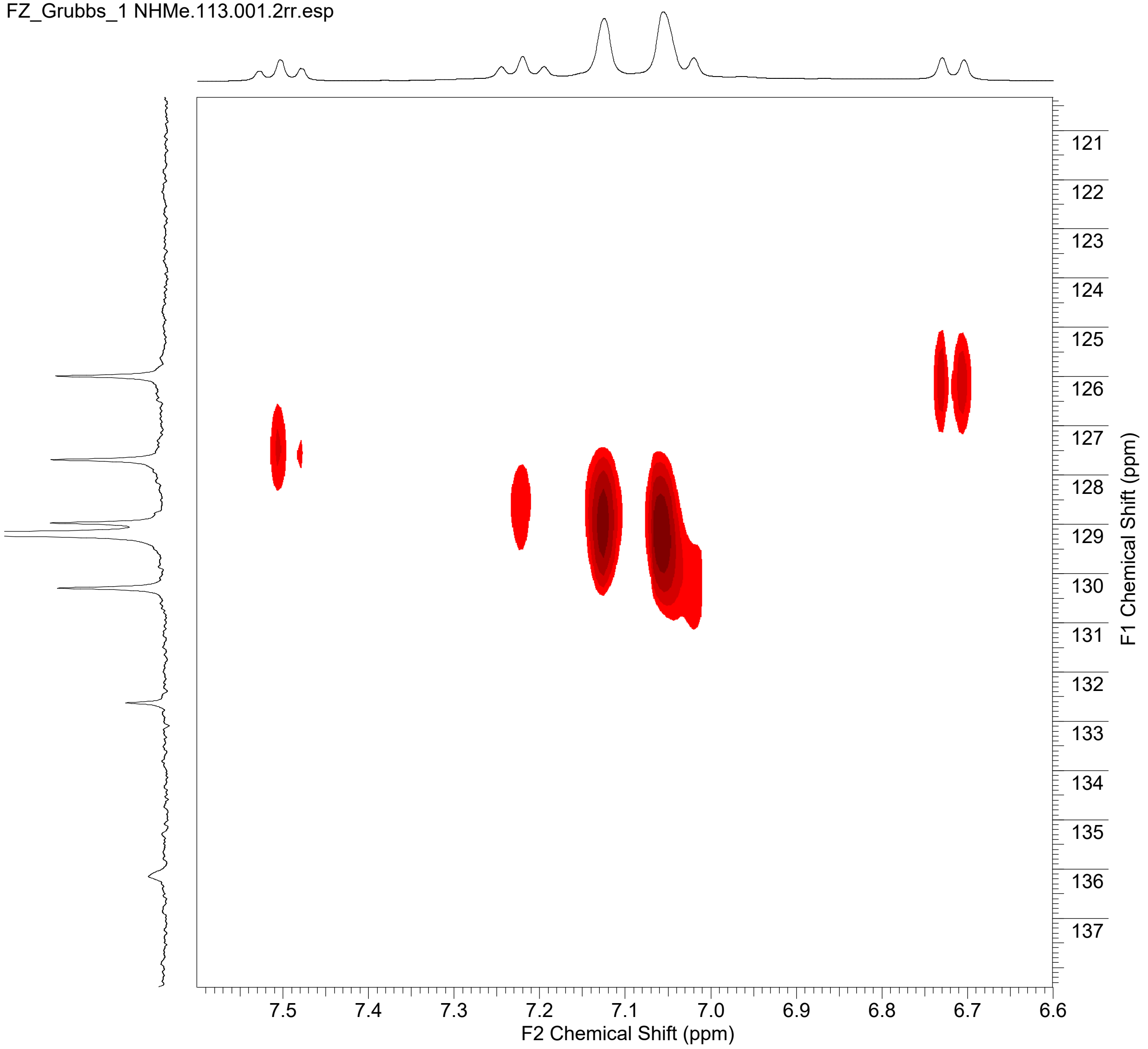


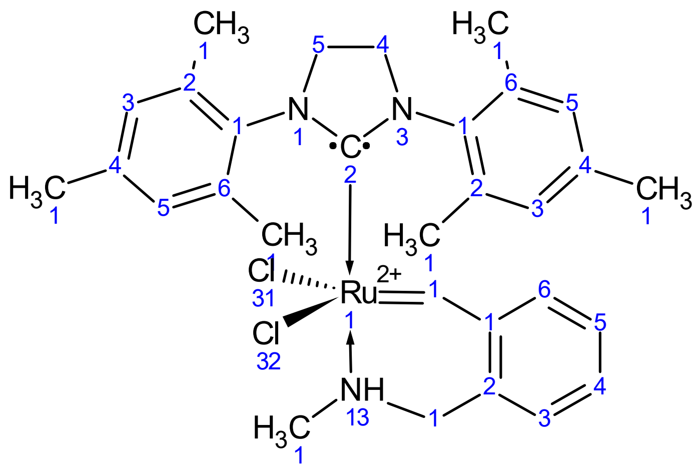

11c

| Acquisition Time (sec) | (0.3408, 0.0069) | Comment | 5 mm PABBO BB-1H/D Z-GRD Z104275/0345 | | |
|---|---|---|---|---|---|
| Date | 02 Oct 2018 21:38:58 | | | | |
| File Name | C:\Users\Fedor\Desktop\Наброски Статей\Кирилл Статья по Катализаторам Граббса\ЯМР Граббс\ЯМР Граббс от Ромы\FZ_Grubbs_1 NHMe\113\pdata\1\2rr | | | | |
| Frequency (MHz) | (300.13, 75.48) | Nucleus | (1H, 13C) | Number of Transients | 2 |
| Origin | spect | Original Points Count | (3072, 233) | Owner | nmr |
| Points Count | (8192, 2048) | Pulse Sequence | hsqcetgp | Solvent | CD2Cl2 |
| Sweep Width (Hz) | (9014.42, 34013.61) | Temperature (degree C) | 29.985 | Title | FZ_Grubbs_1-K4-HSQC [hp-dec] |

| Formula | FW |
|---|---|
| $C_{30}H_{37}Cl_2N_3Ru$ | 611.6098 |

FZ_Grubbs_1 NHMe.113.001.2rr.esp

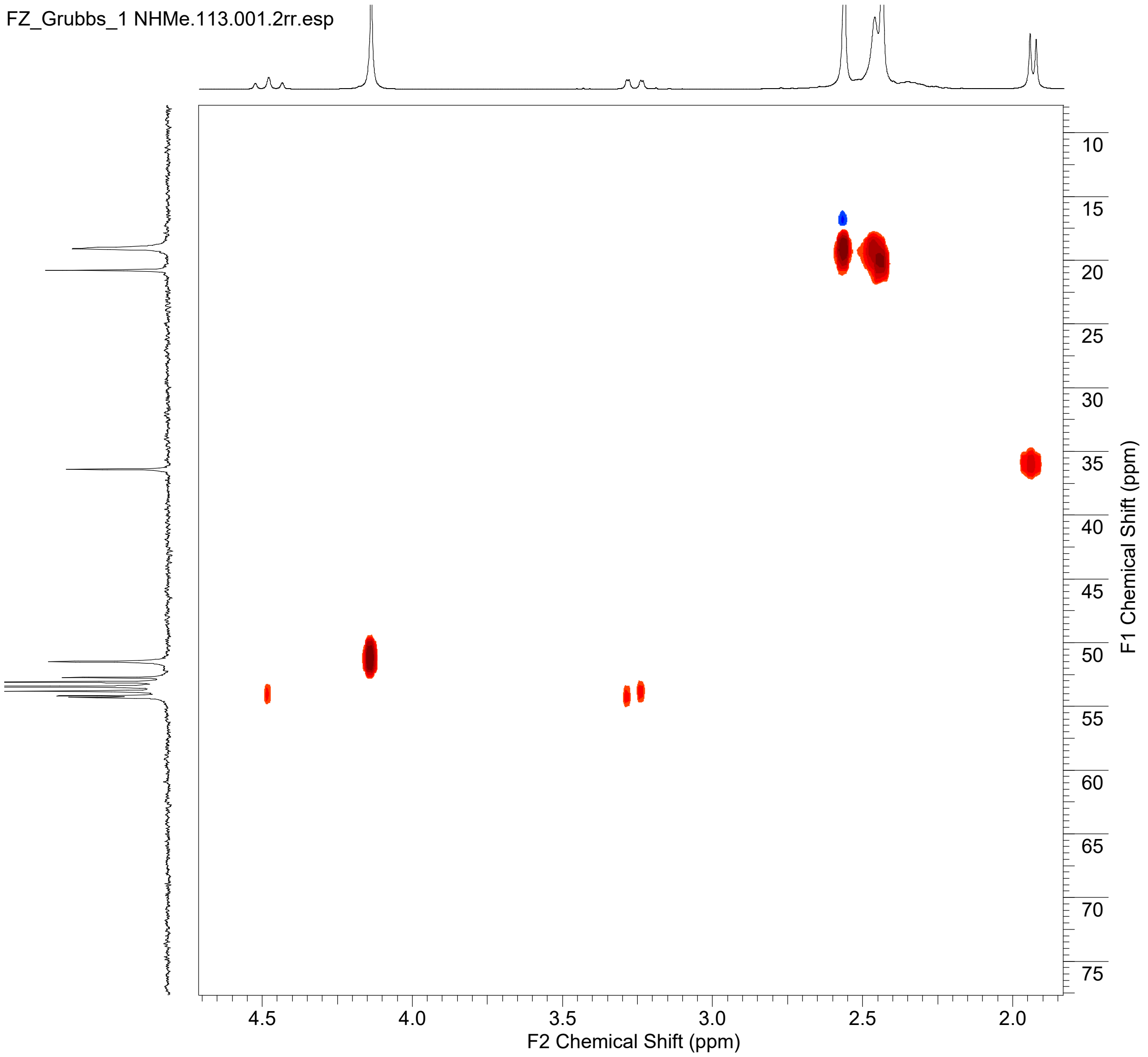


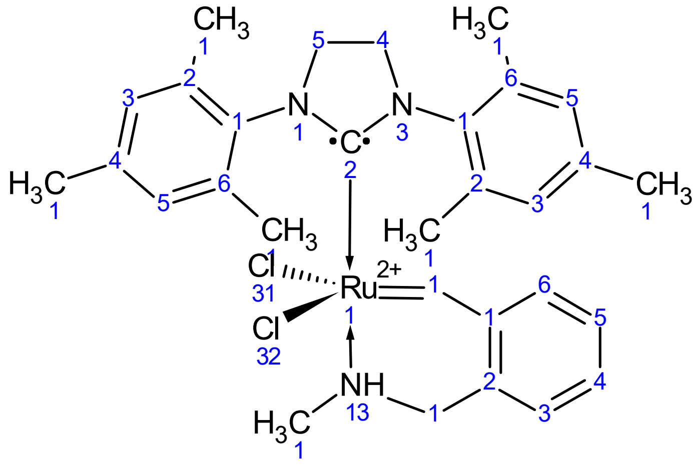


11c

| | | | | | | | |
|---|---|---|---|---|---|---|---|
| ***Acquisition Time (sec)*** | 2.5690 | ***Comment*** | H1 | ***Date*** | 02 Oct 2018 14:56:00 | | |
| ***Date Stamp*** | 02 Oct 2018 14:56:00 | | | | | | |
| ***File Name*** | C:\Users\Fedor\Desktop\Наброски Статей\Кирилл Статья по Катализаторам Граббса\ЯМР Граббс\ЯМР Граббс от Ромы\Grubbs_12-K10 (Морфолиновый)_003001r | | | | | | |
| ***Frequency (MHz)*** | 300.13 | ***Nucleus*** | 1H | ***Number of Transients*** | 32 | ***Origin*** | spect |
| ***Original Points Count*** | 65536 | ***Owner*** | nmr | ***Points Count*** | 262144 | ***Pulse Sequence*** | zg |
| ***Receiver Gain*** | 36.14 | ***SW(cyclical) (Hz)*** | 25510.20 | ***Solvent*** | DICHLOROMETHANE-d2 | | |
| ***Spectrum Offset (Hz)*** | 1350.5919 | ***Sweep Width (Hz)*** | 25510.11 | ***Temperature (degree C)*** | 30.008 | | |

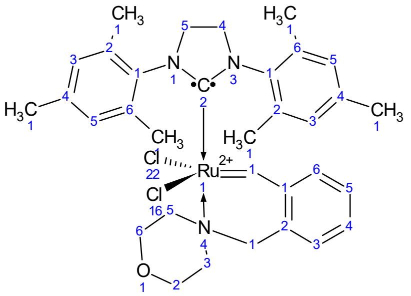

Grubbs_12-K10 (Морфолиновый)_003001r

11d

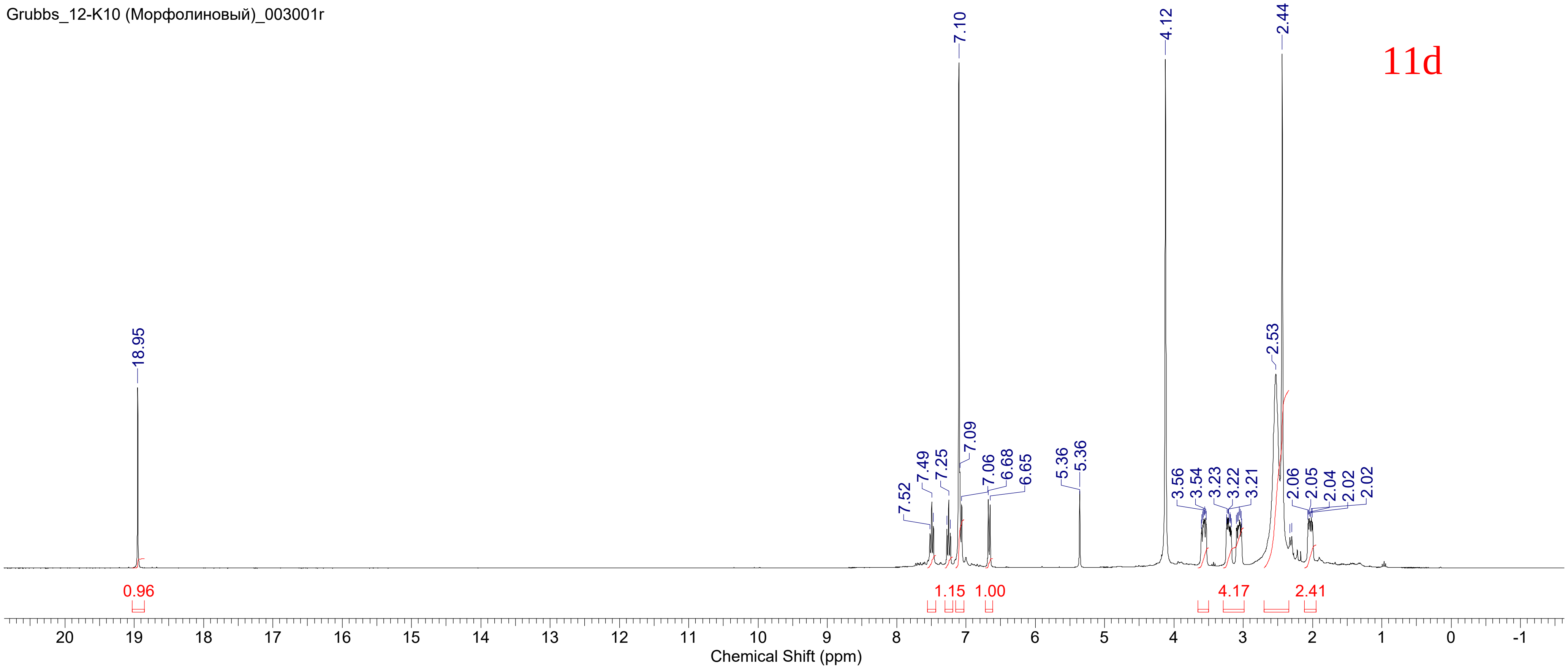

| | | | | | | | |
|---|---|---|---|---|---|---|---|
| ***Acquisition Time (sec)*** | 2.5690 | ***Comment*** | H1 | ***Date*** | 02 Oct 2018 14:56:00 | | |
| ***Date Stamp*** | 02 Oct 2018 14:56:00 | | | | | | |
| ***File Name*** | C:\Users\Fedor\Desktop\Наброски Статей\Кирилл Статья по Катализаторам Граббса\ЯМР Граббс\ЯМР Граббс от Ромы\Grubbs_12-K10 (Морфолиновый)_003001r | | | | | | |
| ***Frequency (MHz)*** | 300.13 | ***Nucleus*** | 1H | ***Number of Transients*** | 32 | ***Origin*** | spect |
| ***Original Points Count*** | 65536 | ***Owner*** | nmr | ***Points Count*** | 262144 | ***Pulse Sequence*** | zg |
| ***Receiver Gain*** | 36.14 | ***SW(cyclical) (Hz)*** | 25510.20 | ***Solvent*** | DICHLOROMETHANE-d2 | | |
| ***Spectrum Offset (Hz)*** | 1350.5919 | ***Sweep Width (Hz)*** | 25510.11 | ***Temperature (degree C)*** | 30.008 | | |

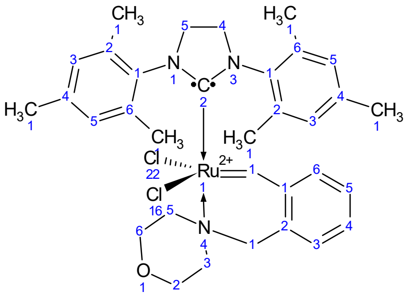

11d

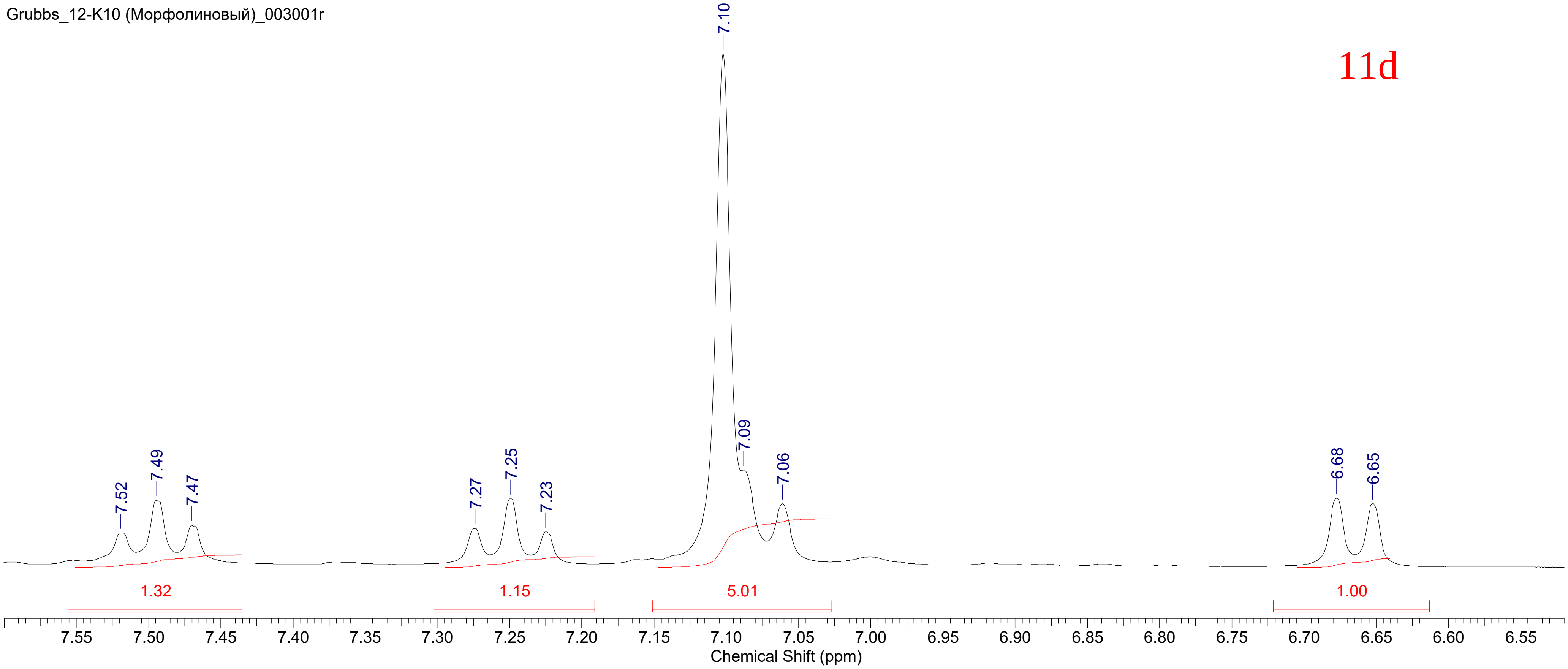

| | | | | | | | |
|---|---|---|---|---|---|---|---|
| ***Acquisition Time (sec)*** | 2.5690 | ***Comment*** | H1 | ***Date*** | 02 Oct 2018 14:56:00 | | |
| ***Date Stamp*** | 02 Oct 2018 14:56:00 | | | | | | |
| ***File Name*** | C:\Users\Fedor\Desktop\Наброски Статей\Кирилл Статья по Катализаторам Граббса\ЯМР Граббс\ЯМР Граббс от Ромы\Grubbs_12-K10 (Морфолиновый)_003001r | | | | | | |
| ***Frequency (MHz)*** | 300.13 | ***Nucleus*** | 1H | ***Number of Transients*** | 32 | ***Origin*** | spect |
| ***Original Points Count*** | 65536 | ***Owner*** | nmr | ***Points Count*** | 262144 | ***Pulse Sequence*** | zg |
| ***Receiver Gain*** | 36.14 | ***SW(cyclical) (Hz)*** | 25510.20 | ***Solvent*** | DICHLOROMETHANE-d2 | | |
| ***Spectrum Offset (Hz)*** | 1350.5919 | ***Sweep Width (Hz)*** | 25510.11 | ***Temperature (degree C)*** | 30.008 | | |

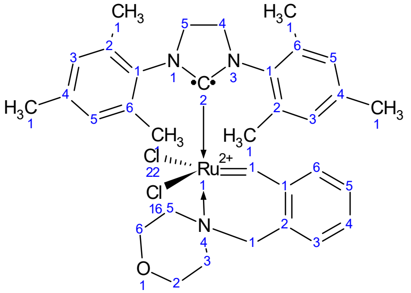

11d

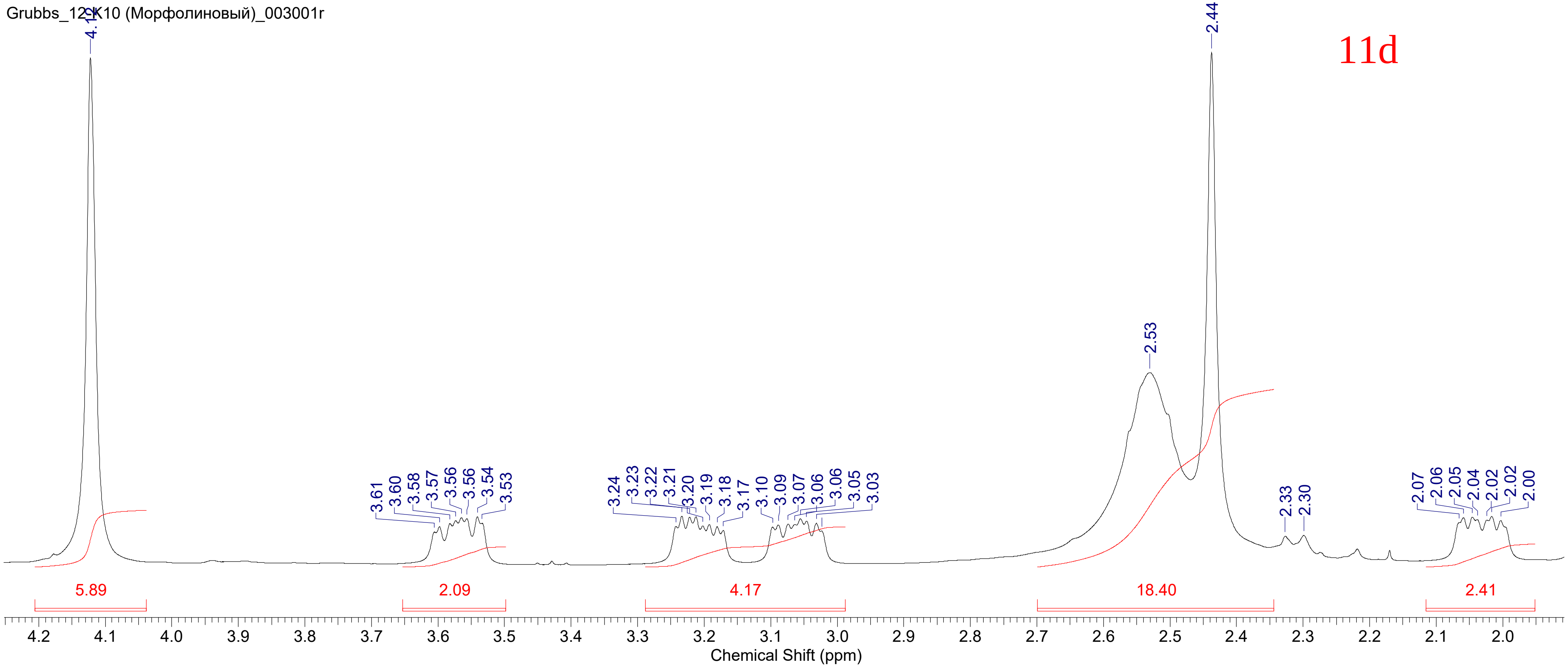

| | | | | | | | |
|---|---|---|---|---|---|---|---|
| ***Acquisition Time (sec)*** | 1.1010 | ***Comment*** | FZ_Grubbs_12-K10-C13dec [hp-dec] | | | ***Date*** | 02 Oct 2018 12:50:08 |
| ***Date Stamp*** | 02 Oct 2018 12:50:08 | | | | | | |
| ***File Name*** | C:\Users\Fedor\Desktop\Наброски Статей\Кирилл Статья по Катализаторам Граббса\ЯМР Граббс\ЯМР Граббс от Ромы\Grubbs_12-K10 (Морфолиновый)_013001r | | | | | | |
| ***Frequency (MHz)*** | 75.47 | ***Nucleus*** | 13C | ***Number of Transients*** | 800 | ***Origin*** | spect |
| ***Original Points Count*** | 65536 | ***Owner*** | nmr | ***Points Count*** | 262144 | ***Pulse Sequence*** | zgpg |
| ***Receiver Gain*** | 202.48 | ***SW(cyclical) (Hz)*** | 59523.81 | ***Solvent*** | DICHLOROMETHANE-d2 | | |
| ***Spectrum Offset (Hz)*** | 7546.7783 | ***Sweep Width (Hz)*** | 59523.58 | ***Temperature (degree C)*** | 30.005 | | |

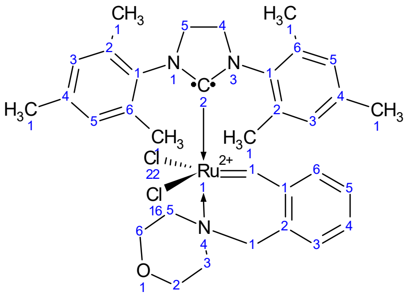

Grubbs_12-K10 (Морфолиновый)_013001r

11d

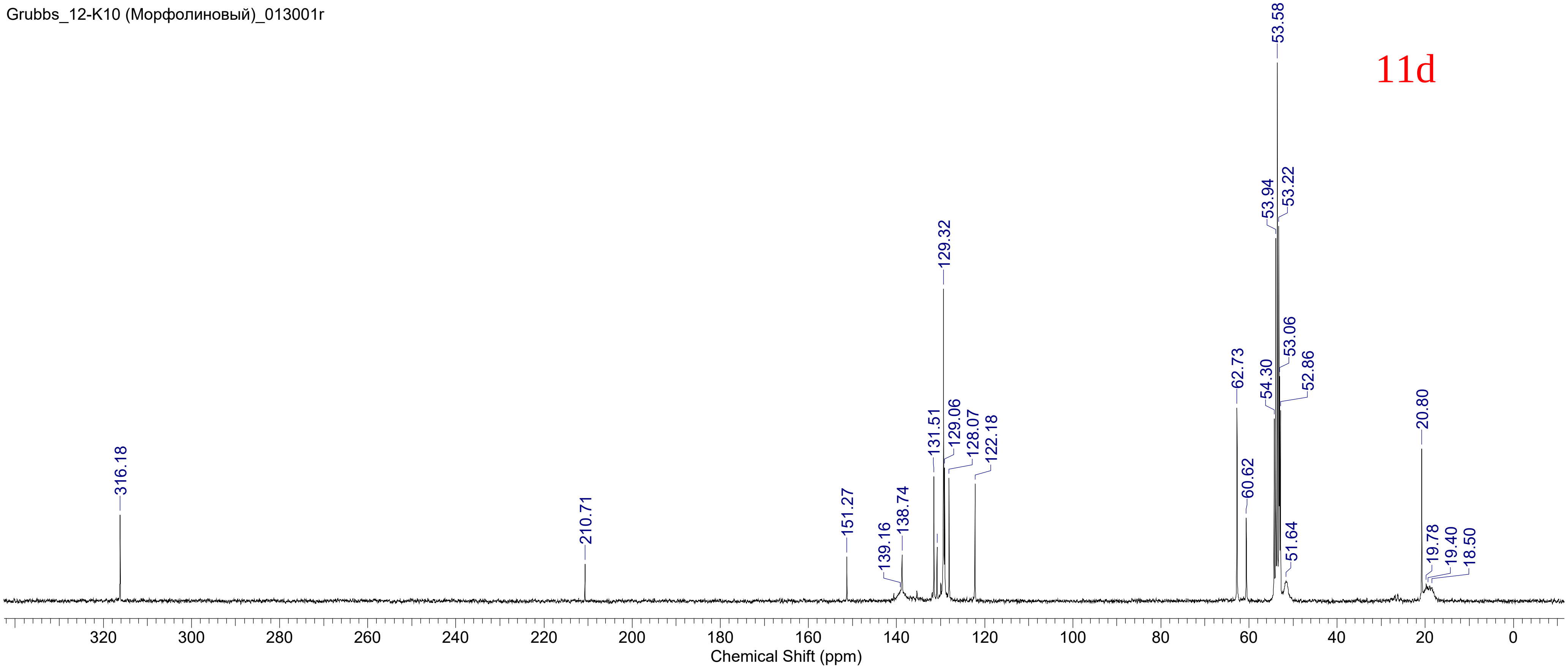

| | | | | | | | |
|---|---|---|---|---|---|---|---|
| ***Acquisition Time (sec)*** | 1.1010 | ***Comment*** | FZ_Grubbs_12-K10-C13dec [hp-dec] | | | ***Date*** | 02 Oct 2018 12:50:08 |
| ***Date Stamp*** | 02 Oct 2018 12:50:08 | | | | | | |
| ***File Name*** | C:\Users\Fedor\Desktop\Наброски Статей\Кирилл Статья по Катализаторам Граббса\ЯМР Граббс\ЯМР Граббс от Ромы\Grubbs_12-K10 (Морфолиновый)_013001r | | | | | | |
| ***Frequency (MHz)*** | 75.47 | ***Nucleus*** | 13C | ***Number of Transients*** | 800 | ***Origin*** | spect |
| ***Original Points Count*** | 65536 | ***Owner*** | nmr | ***Points Count*** | 262144 | ***Pulse Sequence*** | zgpg |
| ***Receiver Gain*** | 202.48 | ***SW(cyclical) (Hz)*** | 59523.81 | ***Solvent*** | DICHLOROMETHANE-d2 | | |
| ***Spectrum Offset (Hz)*** | 7546.7783 | ***Sweep Width (Hz)*** | 59523.58 | ***Temperature (degree C)*** | 30.005 | | |

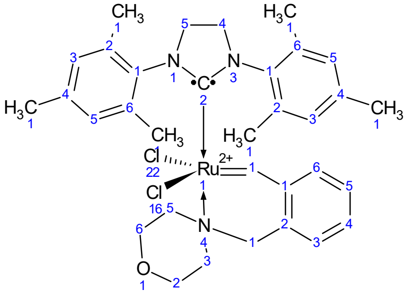

11d

Grubbs_12-K10 (Морфолиновый)_013001r

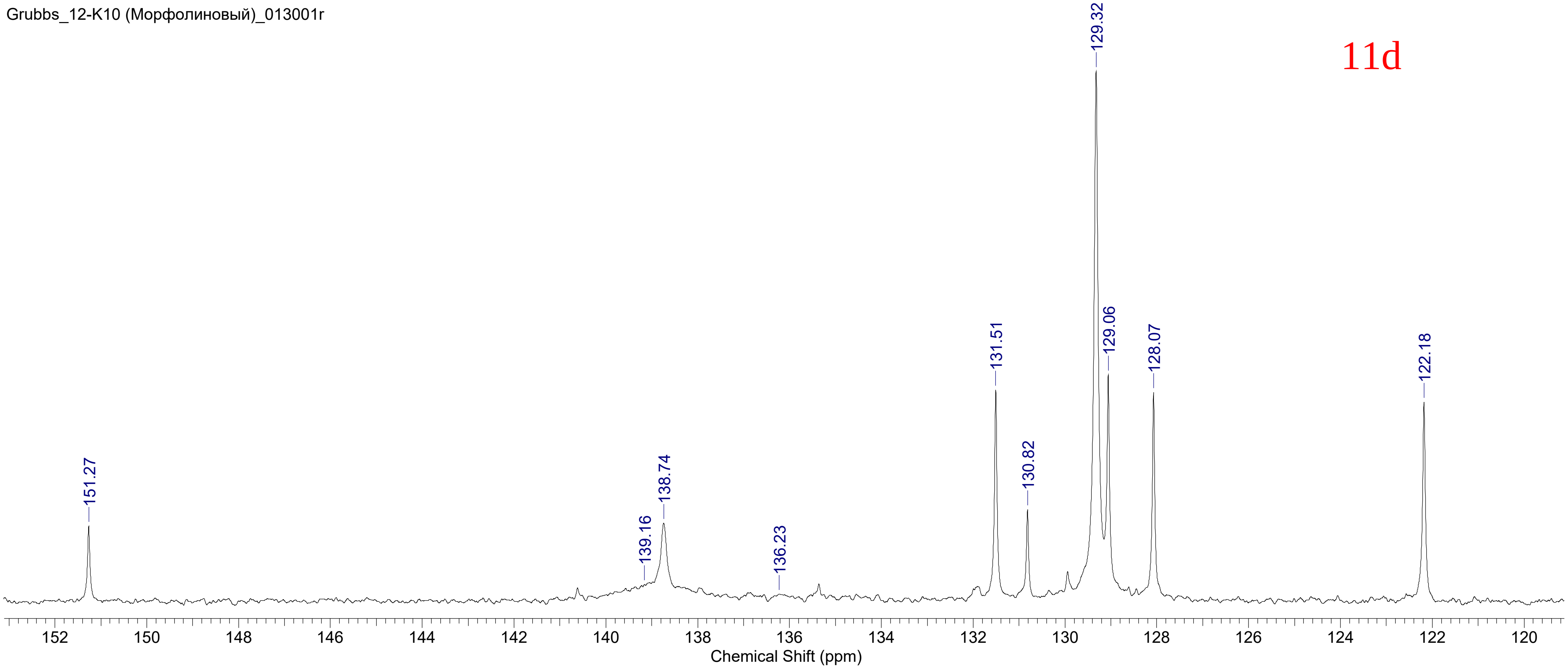

| | | | | | | | |
|---|---|---|---|---|---|---|---|
| ***Acquisition Time (sec)*** | 1.1010 | ***Comment*** | FZ_Grubbs_12-K10-C13dec [hp-dec] | | | ***Date*** | 02 Oct 2018 12:50:08 |
| ***Date Stamp*** | 02 Oct 2018 12:50:08 | | | | | | |
| ***File Name*** | C:\Users\Fedor\Desktop\Наброски Статей\Кирилл Статья по Катализаторам Граббса\ЯМР Граббс\ЯМР Граббс от Ромы\Grubbs_12-K10 (Морфолиновый)_013001r | | | | | | |
| ***Frequency (MHz)*** | 75.47 | ***Nucleus*** | 13C | ***Number of Transients*** | 800 | ***Origin*** | spect |
| ***Original Points Count*** | 65536 | ***Owner*** | nmr | ***Points Count*** | 262144 | ***Pulse Sequence*** | zgpg |
| ***Receiver Gain*** | 202.48 | ***SW(cyclical) (Hz)*** | 59523.81 | ***Solvent*** | DICHLOROMETHANE-d2 | | |
| ***Spectrum Offset (Hz)*** | 7546.7783 | ***Sweep Width (Hz)*** | 59523.58 | ***Temperature (degree C)*** | 30.005 | | |

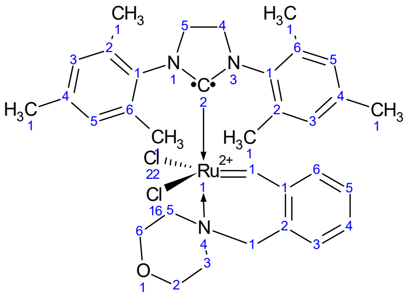

11d

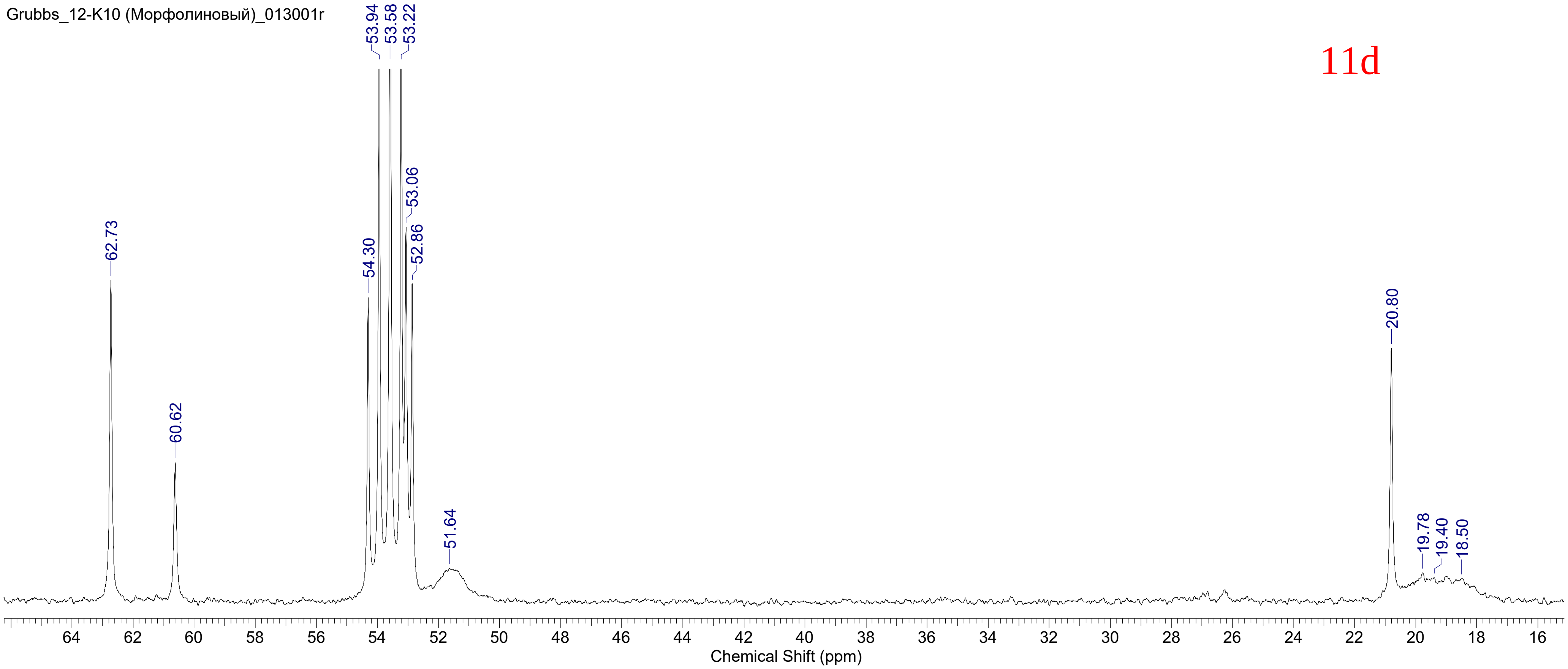

| | | | | | | | |
|---|---|---|---|---|---|---|---|
| ***Acquisition Time (sec)*** | 1.1010 | ***Comment*** | FZ_Grubbs_12-K10-dept135 [hp-dec] | | | ***Date*** | 02 Oct 2018 15:00:16 |
| ***Date Stamp*** | 02 Oct 2018 15:00:16 | | | | | | |
| ***File Name*** | C:\Users\Fedor\Desktop\Наброски Статей\Кирилл Статья по Катализаторам Граббса\ЯМР Граббс\ЯМР Граббс от Ромы\Grubbs_12-K10 (Морфолиновый)_015001r | | | | | | |
| ***Frequency (MHz)*** | 75.47 | ***Nucleus*** | 13C | ***Number of Transients*** | 360 | ***Origin*** | spect |
| ***Original Points Count*** | 65536 | ***Owner*** | nmr | ***Points Count*** | 262144 | ***Pulse Sequence*** | dept135 |
| ***Receiver Gain*** | 202.48 | ***SW(cyclical) (Hz)*** | 59523.81 | ***Solvent*** | DICHLOROMETHANE-d2 | | |
| ***Spectrum Offset (Hz)*** | 7546.7783 | ***Sweep Width (Hz)*** | 59523.58 | ***Temperature (degree C)*** | 29.989 | | |

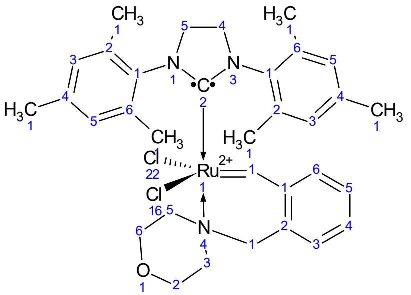

11d

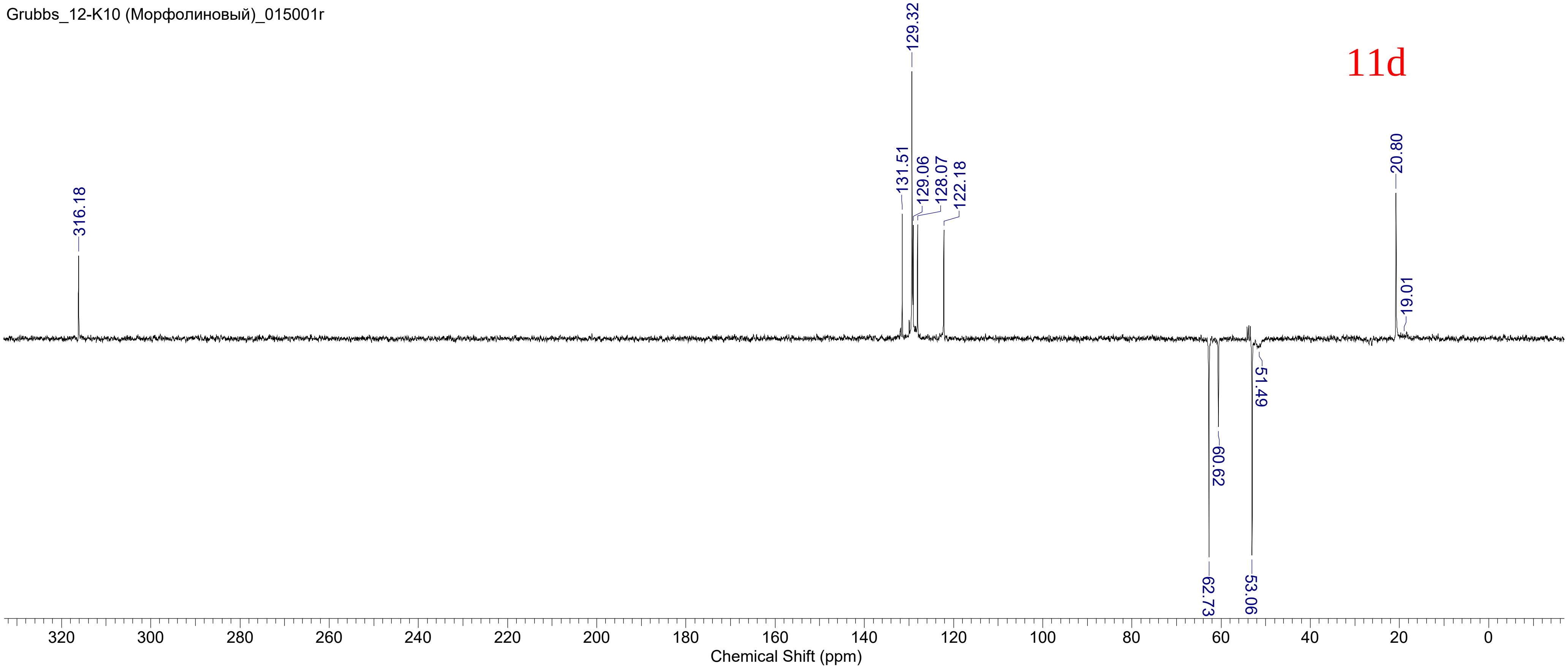

| | | | | | | | |
|---|---|---|---|---|---|---|---|
| ***Acquisition Time (sec)*** | 1.1010 | ***Comment*** | FZ_Grubbs_12-K10-dept135 [hp-dec] | | | ***Date*** | 02 Oct 2018 15:00:16 |
| ***Date Stamp*** | 02 Oct 2018 15:00:16 | | | | | | |
| ***File Name*** | C:\Users\Fedor\Desktop\Наброски Статей\Кирилл Статья по Катализаторам Граббса\ЯМР Граббс\ЯМР Граббс от Ромы\Grubbs_12-K10 (Морфолиновый)_015001r | | | | | | |
| ***Frequency (MHz)*** | 75.47 | ***Nucleus*** | 13C | ***Number of Transients*** | 360 | ***Origin*** | spect |
| ***Original Points Count*** | 65536 | ***Owner*** | nmr | ***Points Count*** | 262144 | ***Pulse Sequence*** | dept135 |
| ***Receiver Gain*** | 202.48 | ***SW(cyclical) (Hz)*** | 59523.81 | ***Solvent*** | DICHLOROMETHANE-d2 | | |
| ***Spectrum Offset (Hz)*** | 7546.7783 | ***Sweep Width (Hz)*** | 59523.58 | ***Temperature (degree C)*** | 29.989 | | |

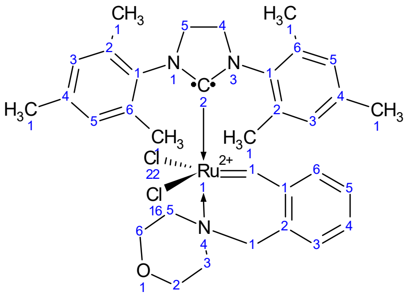

11d

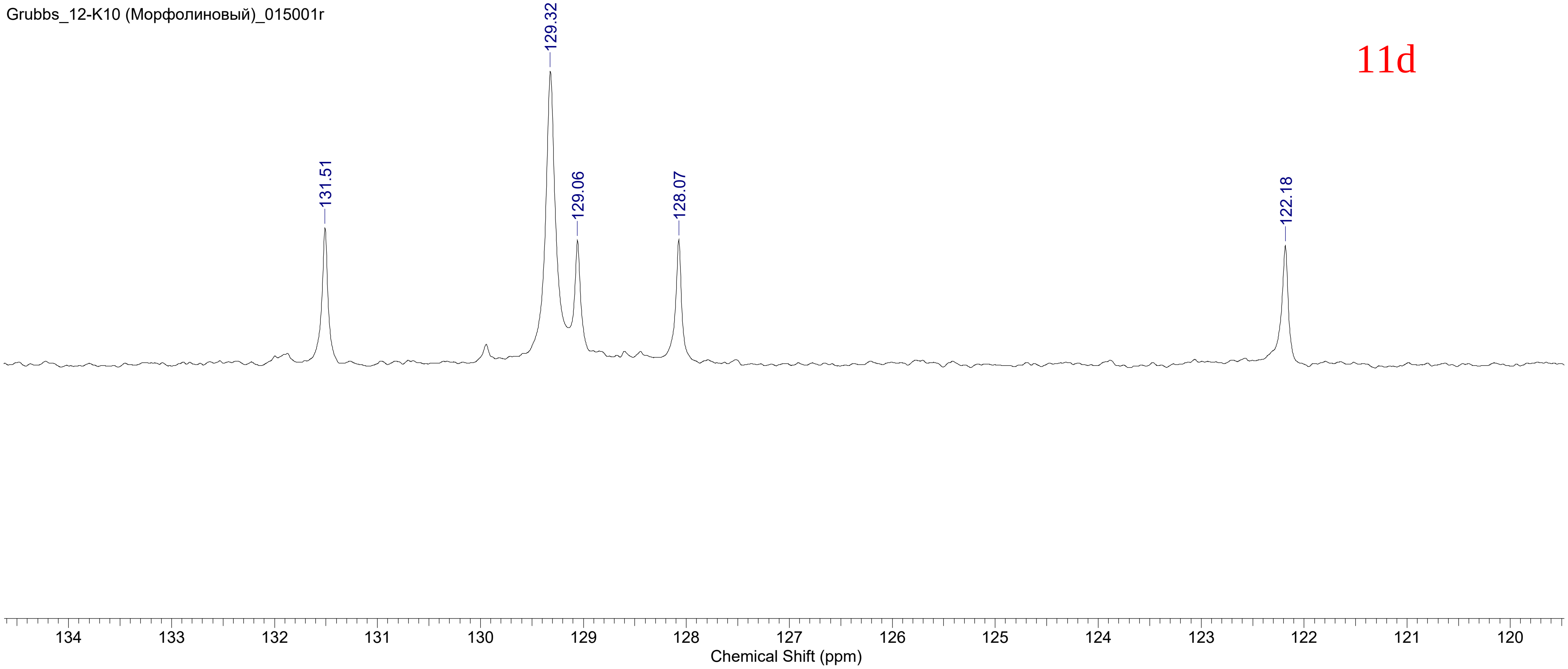

| | | | | | | | |
|---|---|---|---|---|---|---|---|
| ***Acquisition Time (sec)*** | 1.1010 | ***Comment*** | FZ_Grubbs_12-K10-dept135 [hp-dec] | | | ***Date*** | 02 Oct 2018 15:00:16 |
| ***Date Stamp*** | 02 Oct 2018 15:00:16 | | | | | | |
| ***File Name*** | C:\Users\Fedor\Desktop\Наброски Статей\Кирилл Статья по Катализаторам Граббса\ЯМР Граббс\ЯМР Граббс от Ромы\Grubbs_12-K10 (Морфолиновый)_015001r | | | | | | |
| ***Frequency (MHz)*** | 75.47 | ***Nucleus*** | 13C | ***Number of Transients*** | 360 | ***Origin*** | spect |
| ***Original Points Count*** | 65536 | ***Owner*** | nmr | ***Points Count*** | 262144 | ***Pulse Sequence*** | dept135 |
| ***Receiver Gain*** | 202.48 | ***SW(cyclical) (Hz)*** | 59523.81 | ***Solvent*** | DICHLOROMETHANE-d2 | | |
| ***Spectrum Offset (Hz)*** | 7546.7783 | ***Sweep Width (Hz)*** | 59523.58 | ***Temperature (degree C)*** | 29.989 | | |

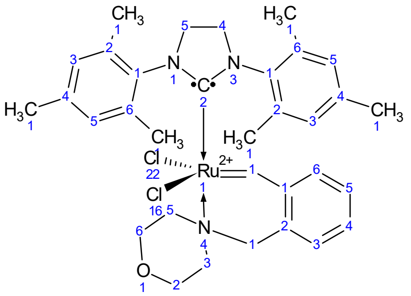

Grubbs_12-K10 (Морфолиновый)_015001r

11d

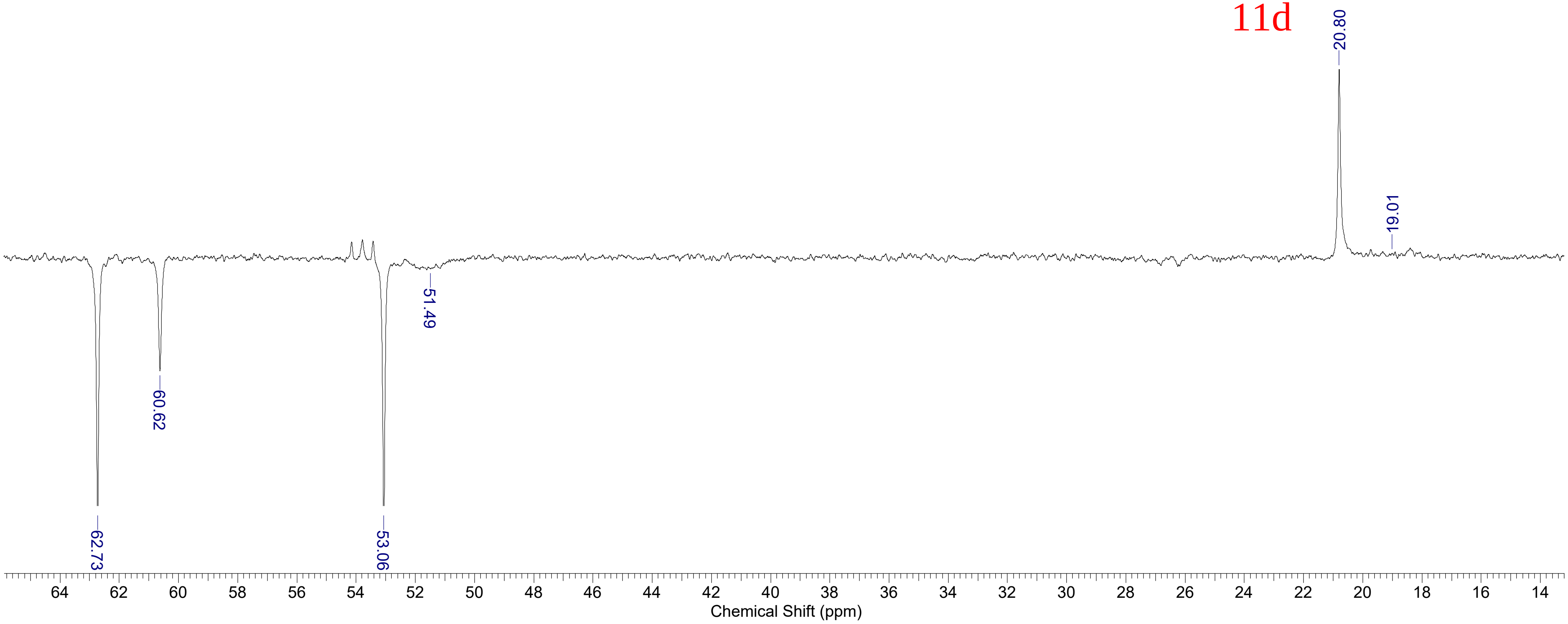

| Acquisition Time (sec) | 2.5690 | Comment | FZ_Grubbs_12-FZ6921 (cyclo){-Ru[SIMes]Cl2=CH-C6H4-CH2-N[-CH2-CH2-O-CH2-CH2-]-} | | | | |
|---|---|---|---|---|---|---|---|
| Date | 02 Nov 2018 19:01:20 | | | Date Stamp | 02 Nov 2018 19:01:20 | | |
| File Name | C:\Users\Fedor\Desktop\FZ_Grubbs_12-FZ6921_c13dec-night_001001r | | | | | Frequency (MHz) | 300.13 |
| Nucleus | 1H | Number of Transients | 26 | Origin | spect | Original Points Count | 65536 |
| Owner | nmr | Points Count | 262144 | Pulse Sequence | zg | Receiver Gain | 130.70 |
| SW(cyclical) (Hz) | 25510.20 | Solvent | CHLOROFORM-d | Spectrum Offset (Hz) | 1342.4913 | Sweep Width (Hz) | 25510.11 |
| Temperature (degree C) | 29.983 | | | | | | |

11d in CDCl3

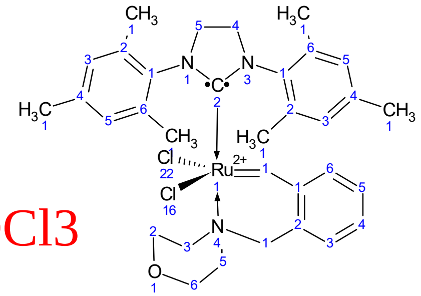

FZ_Grubbs_12-FZ6921_c13dec-night_001001r

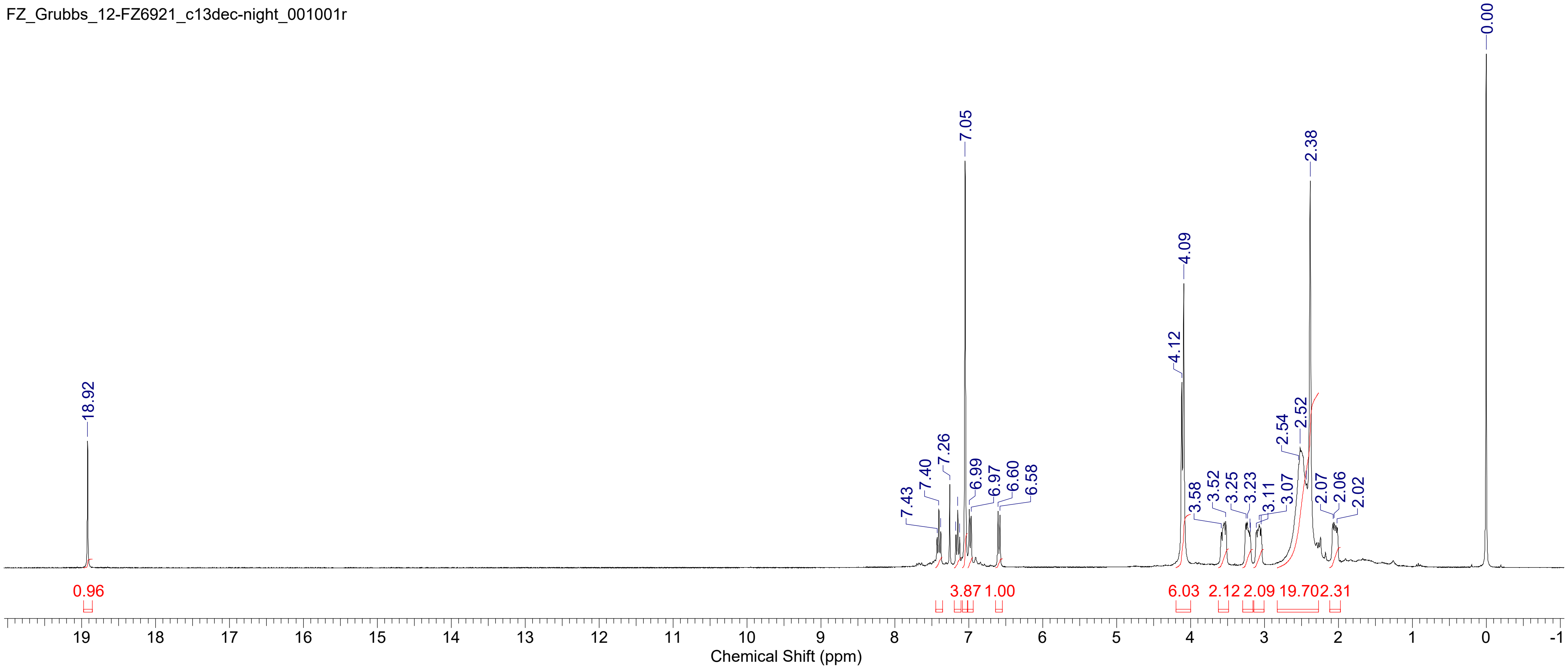

| | | | | | | | |
|---|---|---|---|---|---|---|---|
| ***Acquisition Time (sec)*** | 2.5690 | ***Comment*** | FZ_Grubbs_12-FZ6921 (cyclo){-Ru[SIMes]Cl2=CH-C6H4-CH2-N[-CH2-CH2-O-CH2-CH2-]-} | | | | |
| ***Date*** | 02 Nov 2018 19:01:20 | | | ***Date Stamp*** | 02 Nov 2018 19:01:20 | | |
| ***File Name*** | C:\Users\Fedor\Desktop\FZ_Grubbs_12-FZ6921_c13dec-night_001001r | | | | | ***Frequency (MHz)*** | 300.13 |
| ***Nucleus*** | 1H | ***Number of Transients*** | 26 | ***Origin*** | spect | ***Original Points Count*** | 65536 |
| ***Owner*** | nmr | ***Points Count*** | 262144 | ***Pulse Sequence*** | zg | ***Receiver Gain*** | 130.70 |
| ***SW(cyclical) (Hz)*** | 25510.20 | ***Solvent*** | CHLOROFORM-d | ***Spectrum Offset (Hz)*** | 1342.4913 | ***Sweep Width (Hz)*** | 25510.11 |
| ***Temperature (degree C)*** | 29.983 | | | | | | |

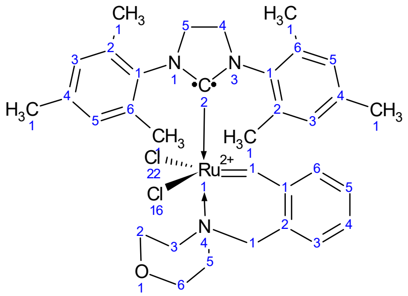

11d in CDCl3

FZ_Grubbs_12-FZ6921_c13dec-night_001001r

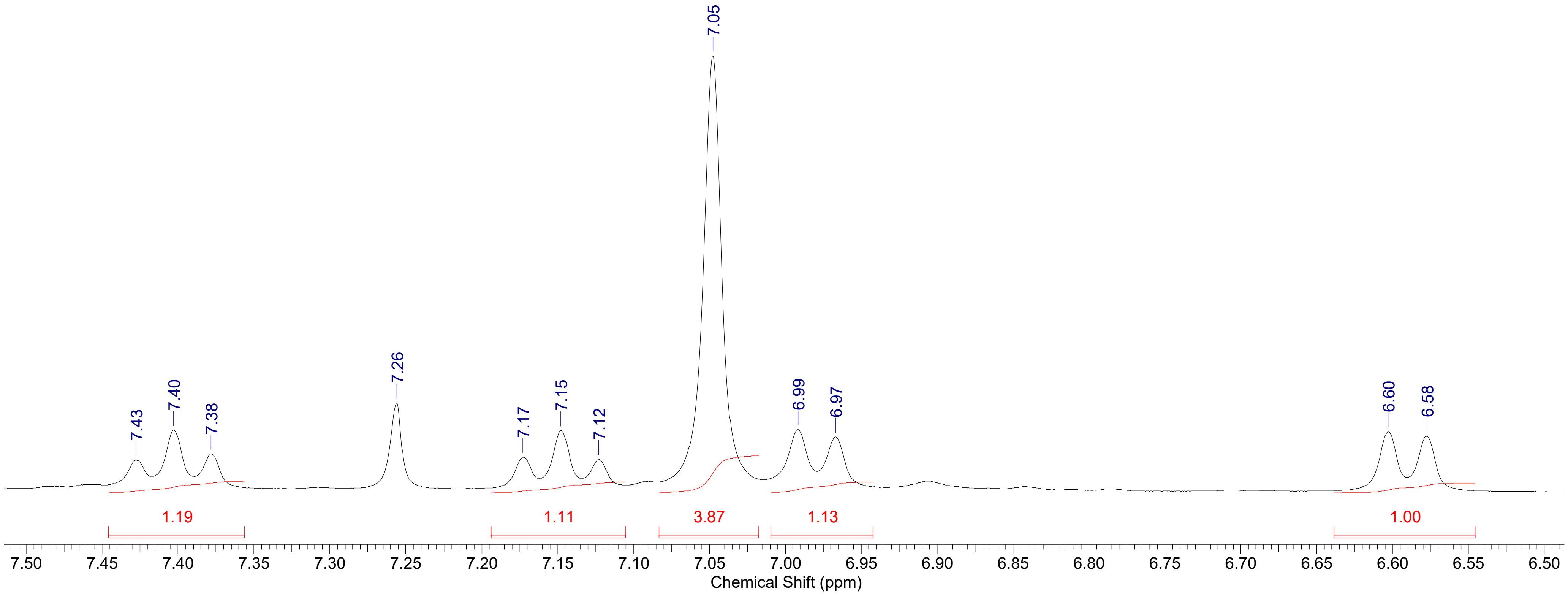

| | | | | | | | |
|---|---|---|---|---|---|---|---|
| *Acquisition Time (sec)* | 2.5690 | *Comment* | FZ_Grubbs_12-FZ6921 (cyclo){-Ru[SIMes]Cl2=CH-C6H4-CH2-N[-CH2-CH2-O-CH2-CH2-]-} | | | | |
| *Date* | 02 Nov 2018 19:01:20 | | | *Date Stamp* | 02 Nov 2018 19:01:20 | | |
| *File Name* | C:\Users\Fedor\Desktop\FZ_Grubbs_12-FZ6921_c13dec-night_001001r | | | | | *Frequency (MHz)* | 300.13 |
| *Nucleus* | 1H | *Number of Transients* | 26 | *Origin* | spect | *Original Points Count* | 65536 |
| *Owner* | nmr | *Points Count* | 262144 | *Pulse Sequence* | zg | *Receiver Gain* | 130.70 |
| *SW(cyclical) (Hz)* | 25510.20 | *Solvent* | CHLOROFORM-d | *Spectrum Offset (Hz)* | 1342.4913 | *Sweep Width (Hz)* | 25510.11 |
| *Temperature (degree C)* | 29.983 | | | | | | |

11d in CDCl3

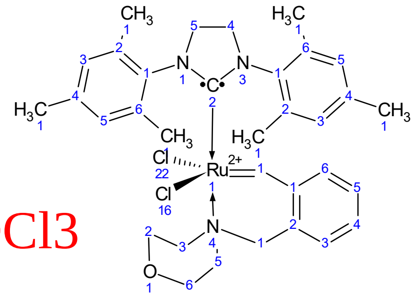

FZ_Grubbs_12-FZ6921_c13dec-night_001001r

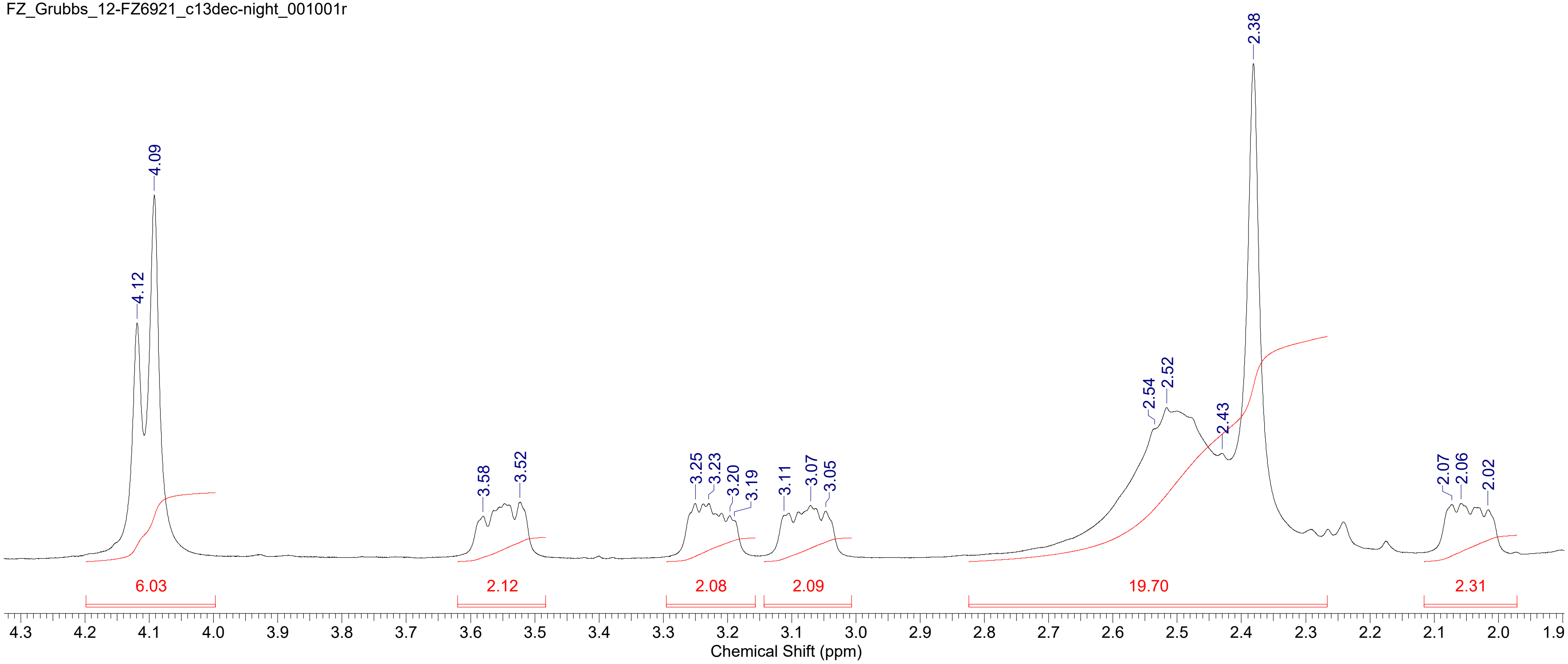

| Acquisition Time (sec) | 1.1010 | Comment | FZ_Grubbs_12-K10-C13dec [hp-dec] [night] | | | Date | 02 Nov 2018 19:07:44 |
|---|---|---|---|---|---|---|---|
| Date Stamp | 02 Nov 2018 19:07:44 | | | File Name | C:\Users\Fedor\Desktop\FZ_Grubbs_12-FZ6921_c13dec-night_013001r | | |
| Frequency (MHz) | 75.47 | Nucleus | 13C | Number of Transients | 24608 | Origin | spect |
| Original Points Count | 65536 | Owner | nmr | Points Count | 262144 | Pulse Sequence | zgpg |
| Receiver Gain | 202.48 | SW(cyclical) (Hz) | 59523.81 | Solvent | CHLOROFORM-d | | |
| Spectrum Offset (Hz) | 7547.7637 | Sweep Width (Hz) | 59523.58 | Temperature (degree C) | 30.040 | | |

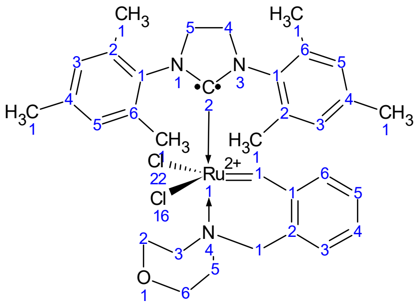

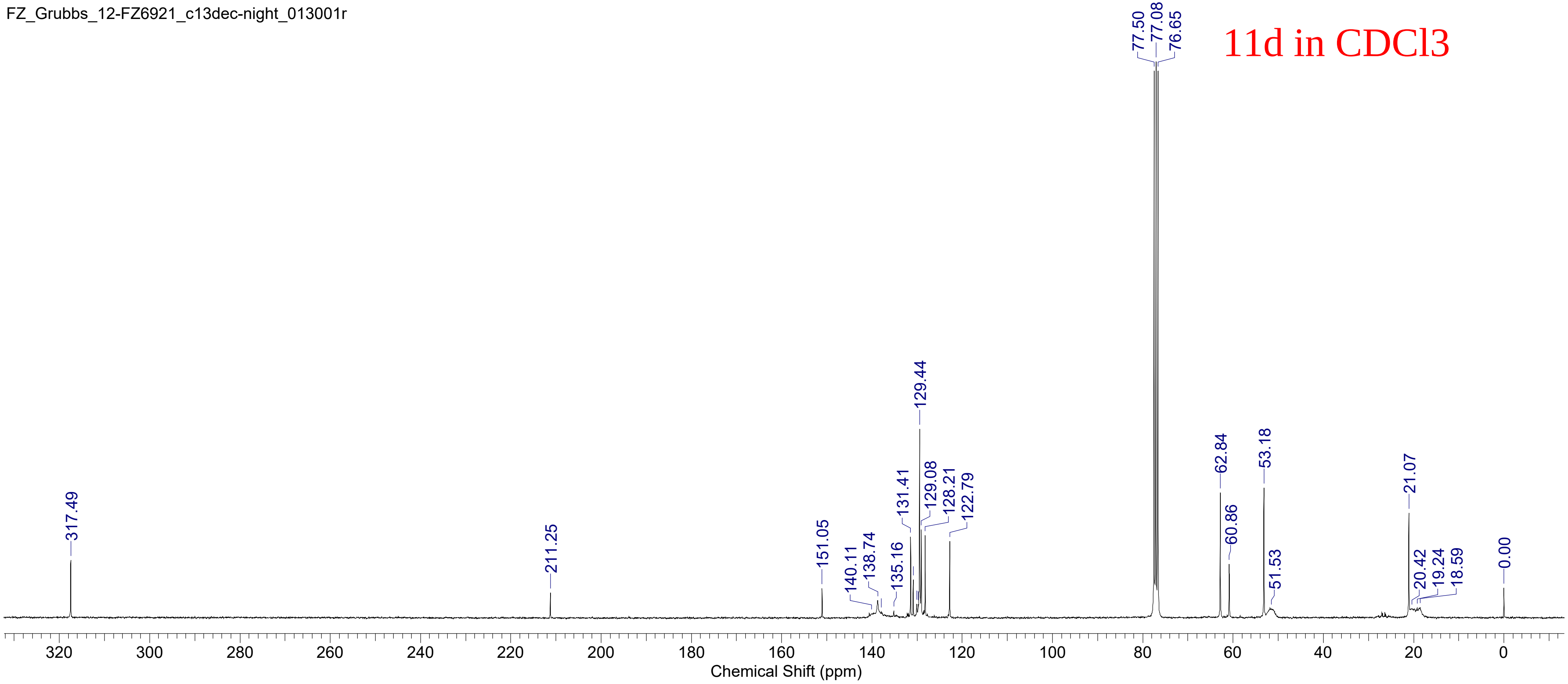

| | | | | | | | |
|---|---|---|---|---|---|---|---|
| ***Acquisition Time (sec)*** | 1.1010 | ***Comment*** | FZ_Grubbs_12-K10-C13dec [hp-dec] [night] | | | ***Date*** | 02 Nov 2018 19:07:44 |
| ***Date Stamp*** | 02 Nov 2018 19:07:44 | | | ***File Name*** | C:\Users\Fedor\Desktop\FZ_Grubbs_12-FZ6921_c13dec-night_013001r | | |
| ***Frequency (MHz)*** | 75.47 | ***Nucleus*** | 13C | ***Number of Transients*** | 24608 | ***Origin*** | spect |
| ***Original Points Count*** | 65536 | ***Owner*** | nmr | ***Points Count*** | 262144 | ***Pulse Sequence*** | zgpg |
| ***Receiver Gain*** | 202.48 | ***SW(cyclical) (Hz)*** | 59523.81 | ***Solvent*** | CHLOROFORM-d | | |
| ***Spectrum Offset (Hz)*** | 7547.7637 | ***Sweep Width (Hz)*** | 59523.58 | ***Temperature (degree C)*** | 30.040 | | |

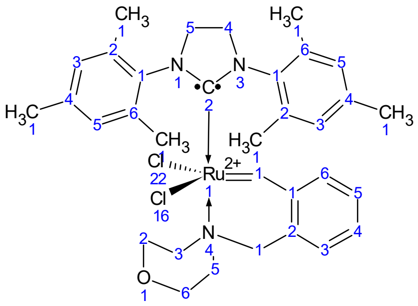

FZ_Grubbs_12-FZ6921_c13dec-night_013001r

11d in CDCl3

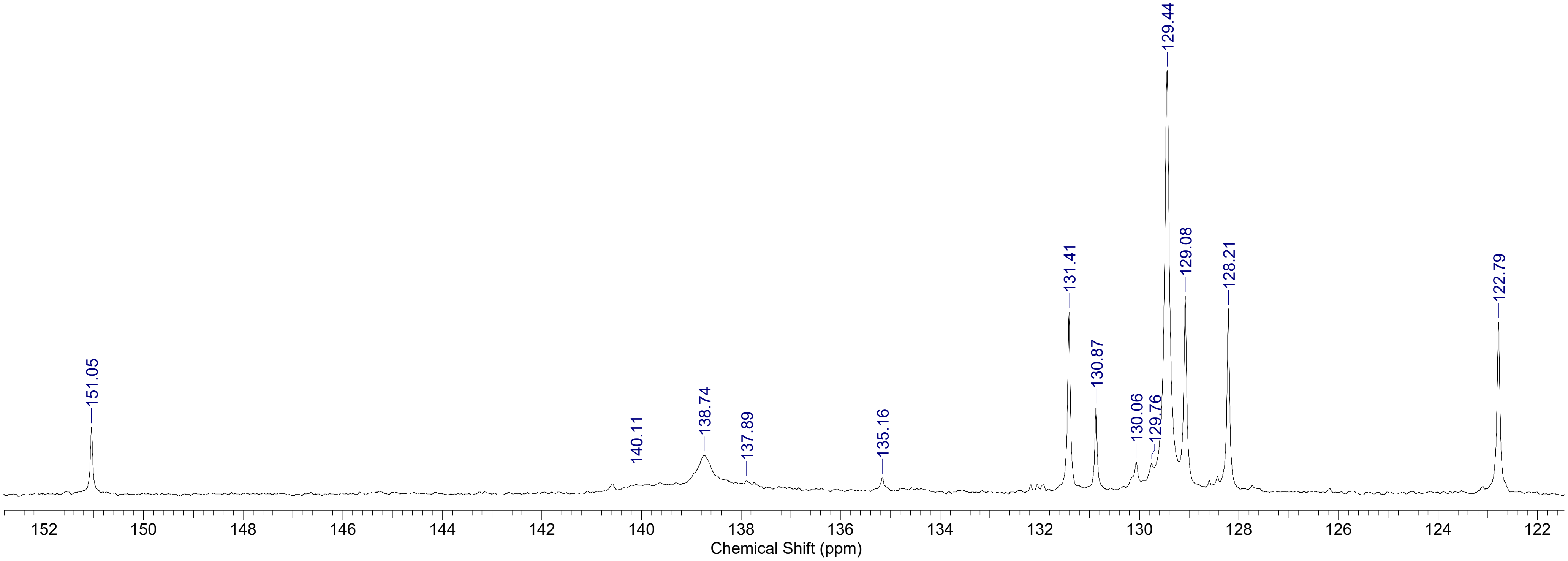

| | | | | | | | |
|---|---|---|---|---|---|---|---|
| ***Acquisition Time (sec)*** | 1.1010 | ***Comment*** | FZ_Grubbs_12-K10-C13dec [hp-dec] [night] | | | ***Date*** | 02 Nov 2018 19:07:44 |
| ***Date Stamp*** | 02 Nov 2018 19:07:44 | | | ***File Name*** | C:\Users\Fedor\Desktop\FZ_Grubbs_12-FZ6921_c13dec-night_013001r | | |
| ***Frequency (MHz)*** | 75.47 | ***Nucleus*** | 13C | ***Number of Transients*** | 24608 | ***Origin*** | spect |
| ***Original Points Count*** | 65536 | ***Owner*** | nmr | ***Points Count*** | 262144 | ***Pulse Sequence*** | zgpg |
| ***Receiver Gain*** | 202.48 | ***SW(cyclical) (Hz)*** | 59523.81 | ***Solvent*** | CHLOROFORM-d | | |
| ***Spectrum Offset (Hz)*** | 7547.7637 | ***Sweep Width (Hz)*** | 59523.58 | ***Temperature (degree C)*** | 30.040 | | |

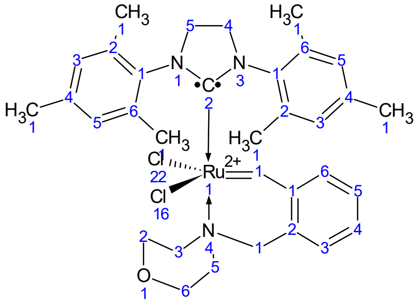

FZ_Grubbs_12-FZ6921_c13dec-night_013001r

11d in CDCl3

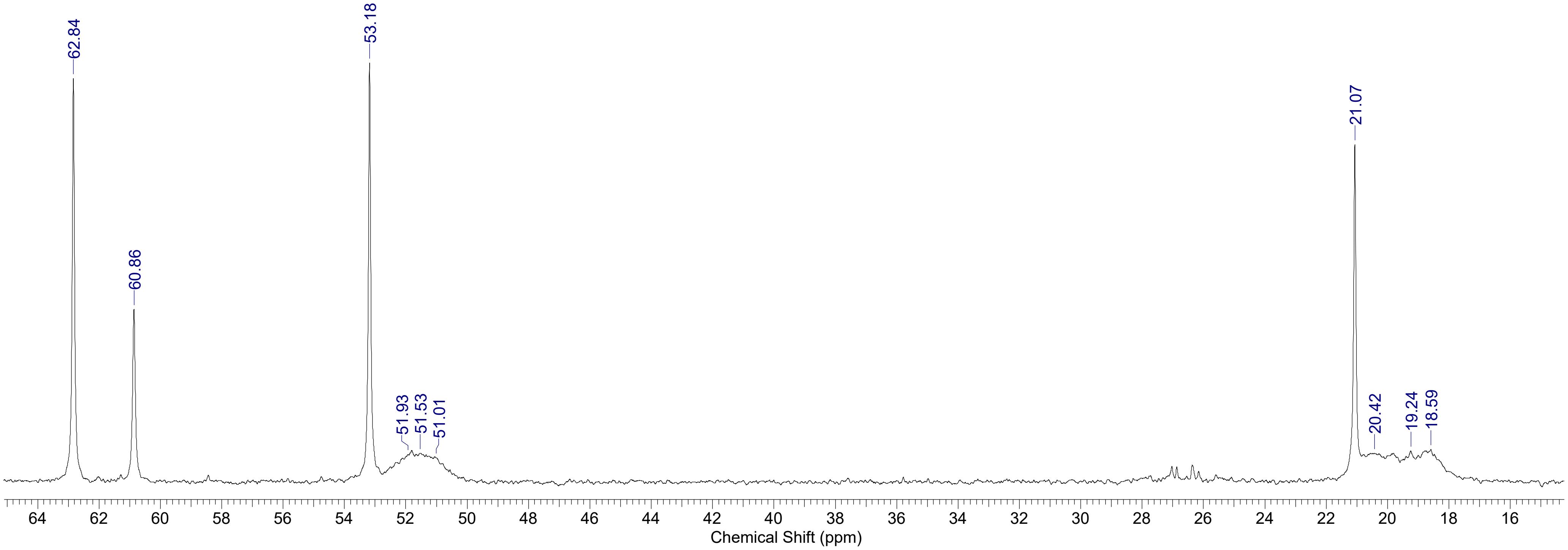

| Acquisition Time (sec) | (0.3408, 0.0074) | Comment | 5 mm PABBO BB-1H/D Z-GRD Z104275/0345 | | | Date | 02 Oct 2018 19:52:12 |
|---|---|---|---|---|---|---|---|
| File Name | C:\Users\Fedor\Desktop\ЯМР Граббс Морфолин\FZ_Grubbs_12-K10\114\pdata\1\2rr | | | | | Frequency (MHz) | (300.13, 75.48) |
| Nucleus | (1H, 13C) | Number of Transients | 2 | Origin | spect | Original Points Count | (3072, 252) |
| Owner | nmr | Points Count | (8192, 2048) | Pulse Sequence | hsqcedetgp | Solvent | CD2Cl2 |
| Sweep Width (Hz) | (9014.42, 34013.61) | Temperature (degree C) | 30.020 | Title | FZ_Grubbs_12-K10-edited-HSQC [hp-dec] | | |

| Formula | FW |
|---|---|
| $C_{33}H_{41}Cl_2N_3ORu$ | 667.6730 |

FZ_Grubbs_12-K10.114.001.2rr.esp

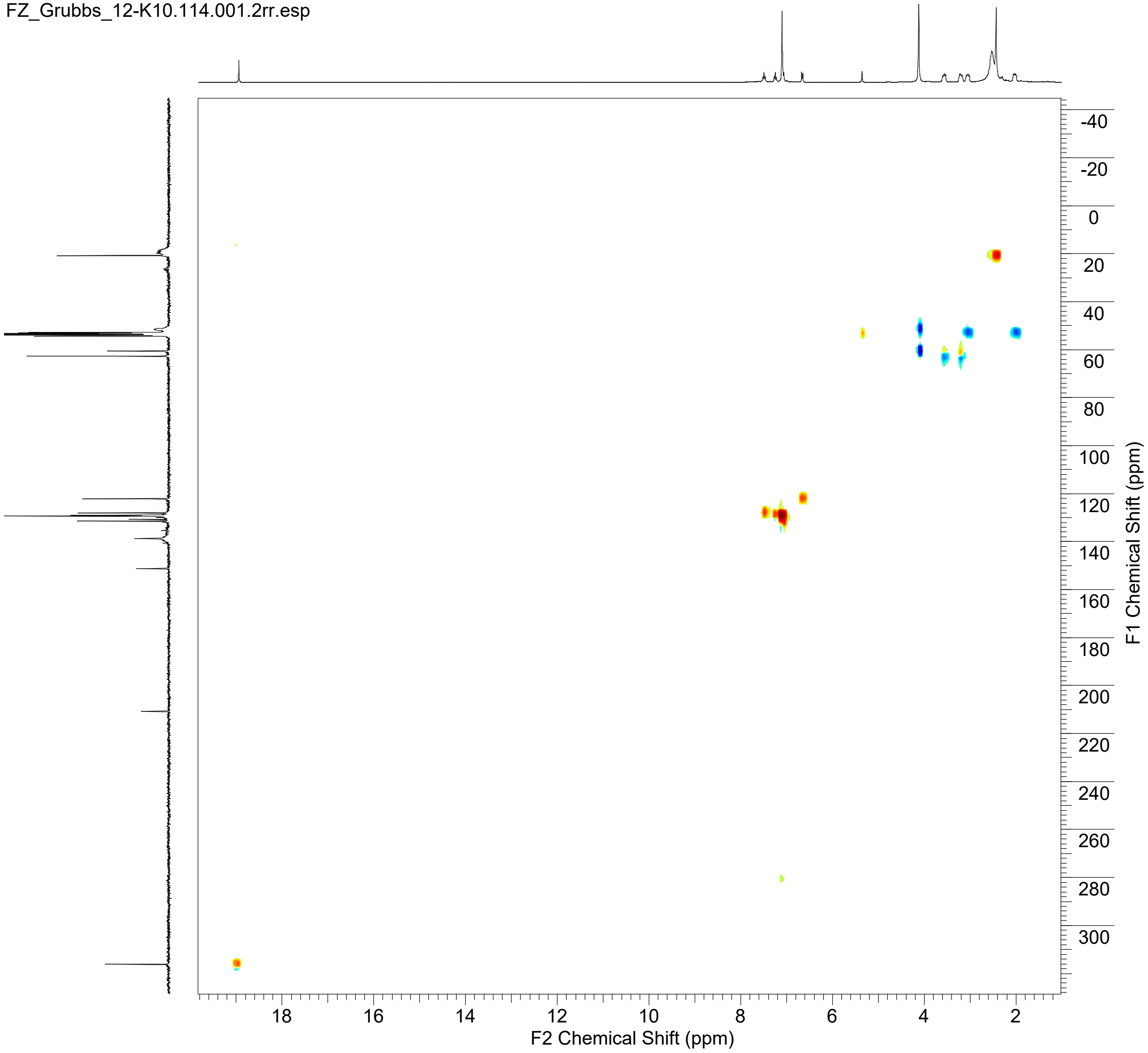


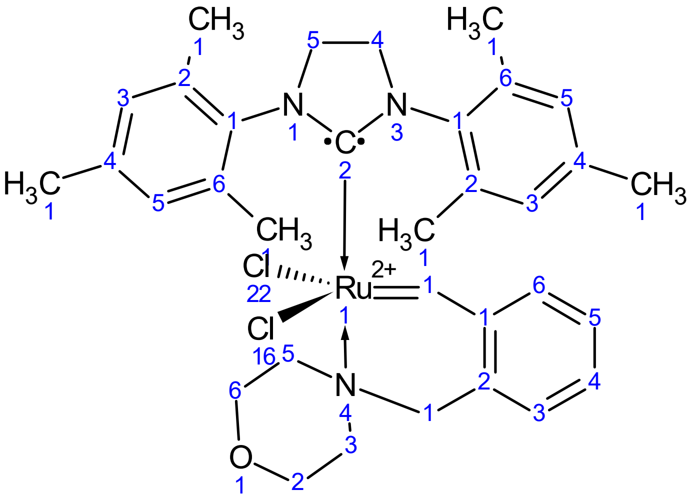

11d

| | | | | | | | |
|---|---|---|---|---|---|---|---|
| ***Acquisition Time (sec)*** | (0.3408, 0.0074) | ***Comment*** | 5 mm PABBO BB-1H/D Z-GRD Z104275/0345 | | | ***Date*** | 02 Oct 2018 19:52:12 |
| ***File Name*** | C:\Users\Fedor\Desktop\ЯМР Граббс Морфолин\FZ_Grubbs_12-K10\114\pdata\1\2rr | | | | | ***Frequency (MHz)*** | (300.13, 75.48) |
| ***Nucleus*** | (1H, 13C) | ***Number of Transients*** | 2 | ***Origin*** | spect | ***Original Points Count*** | (3072, 252) |
| ***Owner*** | nmr | ***Points Count*** | (8192, 2048) | ***Pulse Sequence*** | hsqcedetgp | ***Solvent*** | CD2Cl2 |
| ***Sweep Width (Hz)*** | (9014.42, 34013.61) | ***Temperature (degree C)*** | 30.020 | ***Title*** | FZ_Grubbs_12-K10-edited-HSQC [hp-dec] | | |

| | | | |
|---|---|---|---|
| ***Formula*** | $C_{33}H_{41}Cl_2N_3ORu$ | ***FW*** | 667.6730 |

FZ_Grubbs_12-K10.114.001.2rr.esp

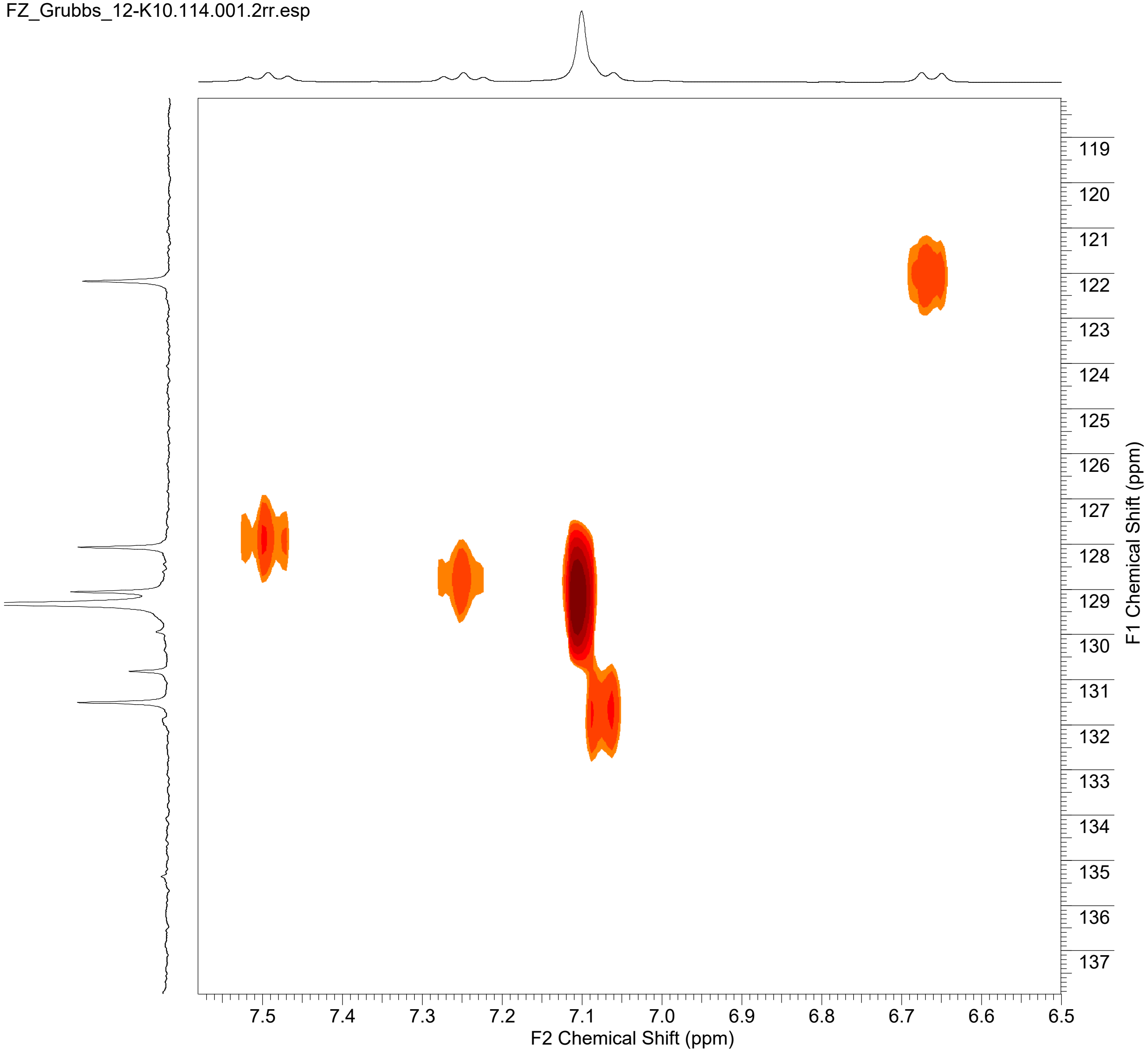


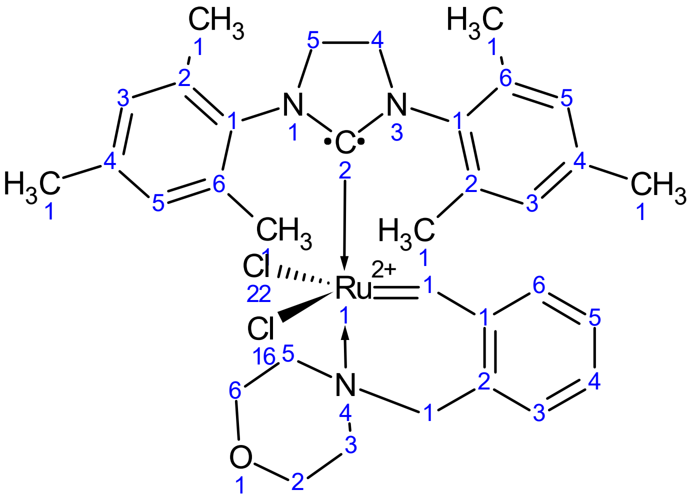

11d

| | | | | | | | |
|---|---|---|---|---|---|---|---|
| **Acquisition Time (sec)** | (0.3408, 0.0074) | **Comment** | 5 mm PABBO BB-1H/D Z-GRD Z104275/0345 | | | **Date** | 02 Oct 2018 19:52:12 |
| **File Name** | C:\Users\Fedor\Desktop\ЯМР Граббс Морфолин\FZ_Grubbs_12-K10\114\pdata\1\2rr | | | | | **Frequency (MHz)** | (300.13, 75.48) |
| **Nucleus** | (1H, 13C) | **Number of Transients** | 2 | **Origin** | spect | **Original Points Count** | (3072, 252) |
| **Owner** | nmr | **Points Count** | (8192, 2048) | **Pulse Sequence** | hsqcedetgp | **Solvent** | CD2Cl2 |
| **Sweep Width (Hz)** | (9014.42, 34013.61) | **Temperature (degree C)** | 30.020 | **Title** | FZ_Grubbs_12-K10-edited-HSQC [hp-dec] | | |

| | | | |
|---|---|---|---|
| **Formula** | $C_{33}H_{41}Cl_2N_3ORu$ | **FW** | 667.6730 |

FZ_Grubbs_12-K10.114.001.2rr.esp

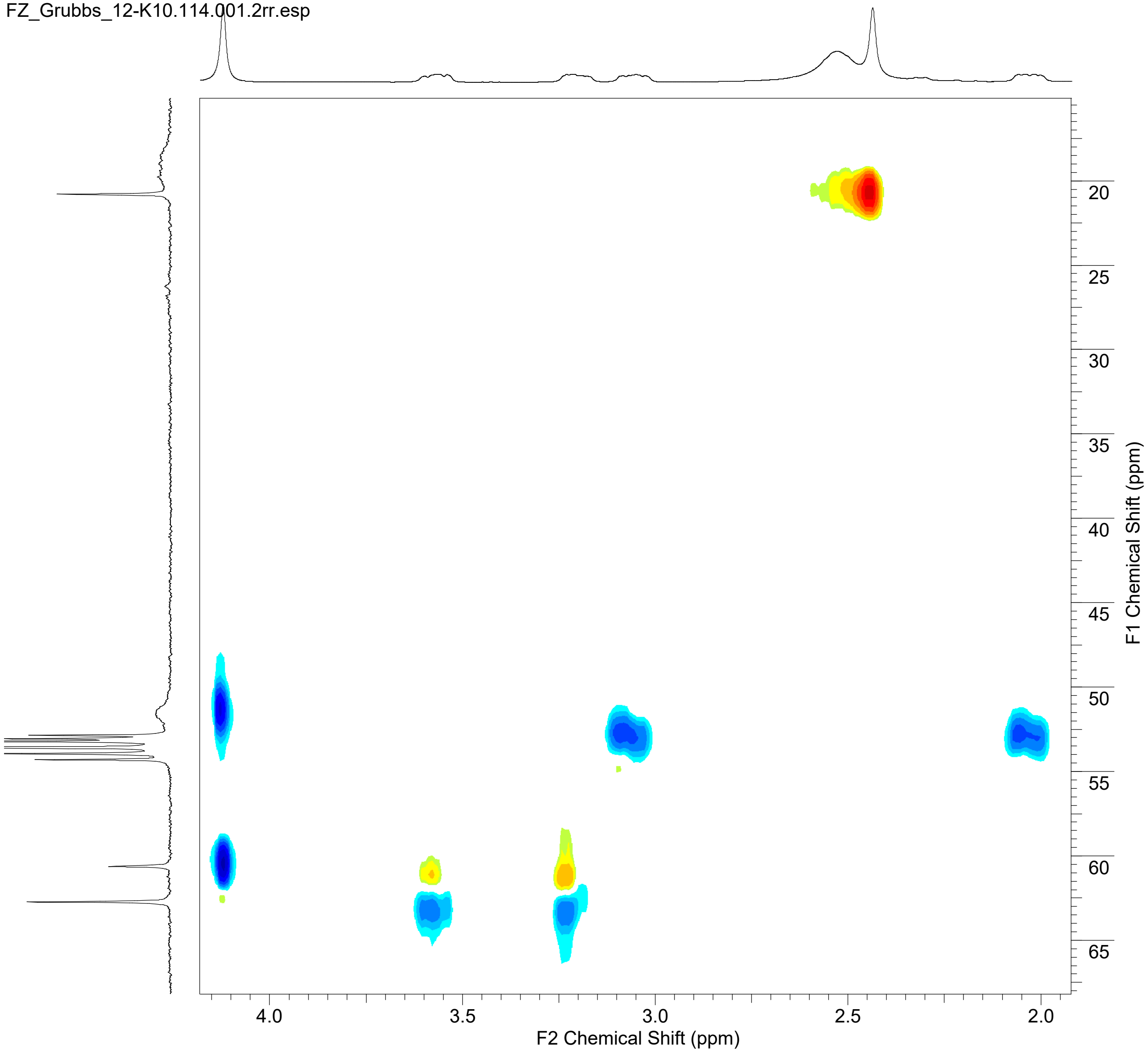


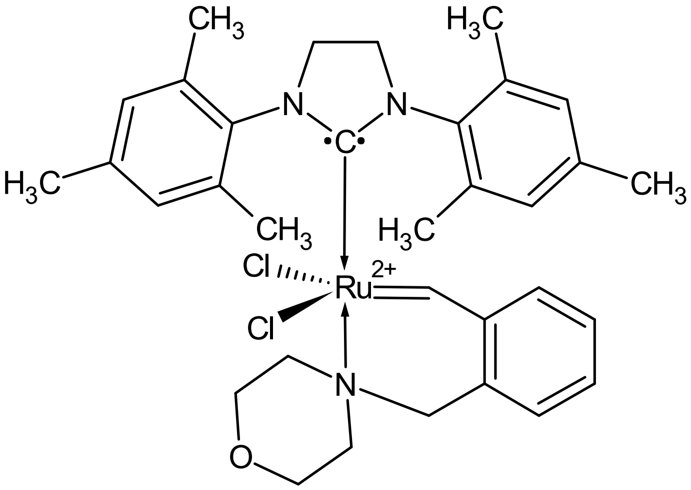


11d

| | | | | | | | |
|---|---|---|---|---|---|---|---|
| ***Acquisition Time (sec)*** | (0.3408, 0.0108) | ***Comment*** | 5 mm PABBO BB-1H/D Z-GRD Z104275/0345 | | | ***Date*** | 02 Oct 2018 20:28:34 |
| ***File Name*** | C:\Users\Fedor\Desktop\ЯМР Граббс Морфолин\FZ_Grubbs_12-K10\213\pdata\1\2rr | | | | | ***Frequency (MHz)*** | (300.13, 75.48) |
| ***Nucleus*** | (1H, 13C) | ***Number of Transients*** | 4 | ***Origin*** | spect | ***Original Points Count*** | (3072, 368) |
| ***Owner*** | nmr | ***Points Count*** | (8192, 2048) | ***Pulse Sequence*** | hmbcgplpndqf | ***Solvent*** | CD2Cl2 |
| ***Sweep Width (Hz)*** | (9014.42, 34013.61) | ***Temperature (degree C)*** | 29.873 | ***Title*** | FZ_Grubbs_12-K10-HMBC | | |

| | | | |
|---|---|---|---|
| ***Formula*** | $C_{33}H_{41}Cl_2N_3ORu$ | ***FW*** | 667.6730 |

FZ_Grubbs_12-K10.213.001.2rr.esp

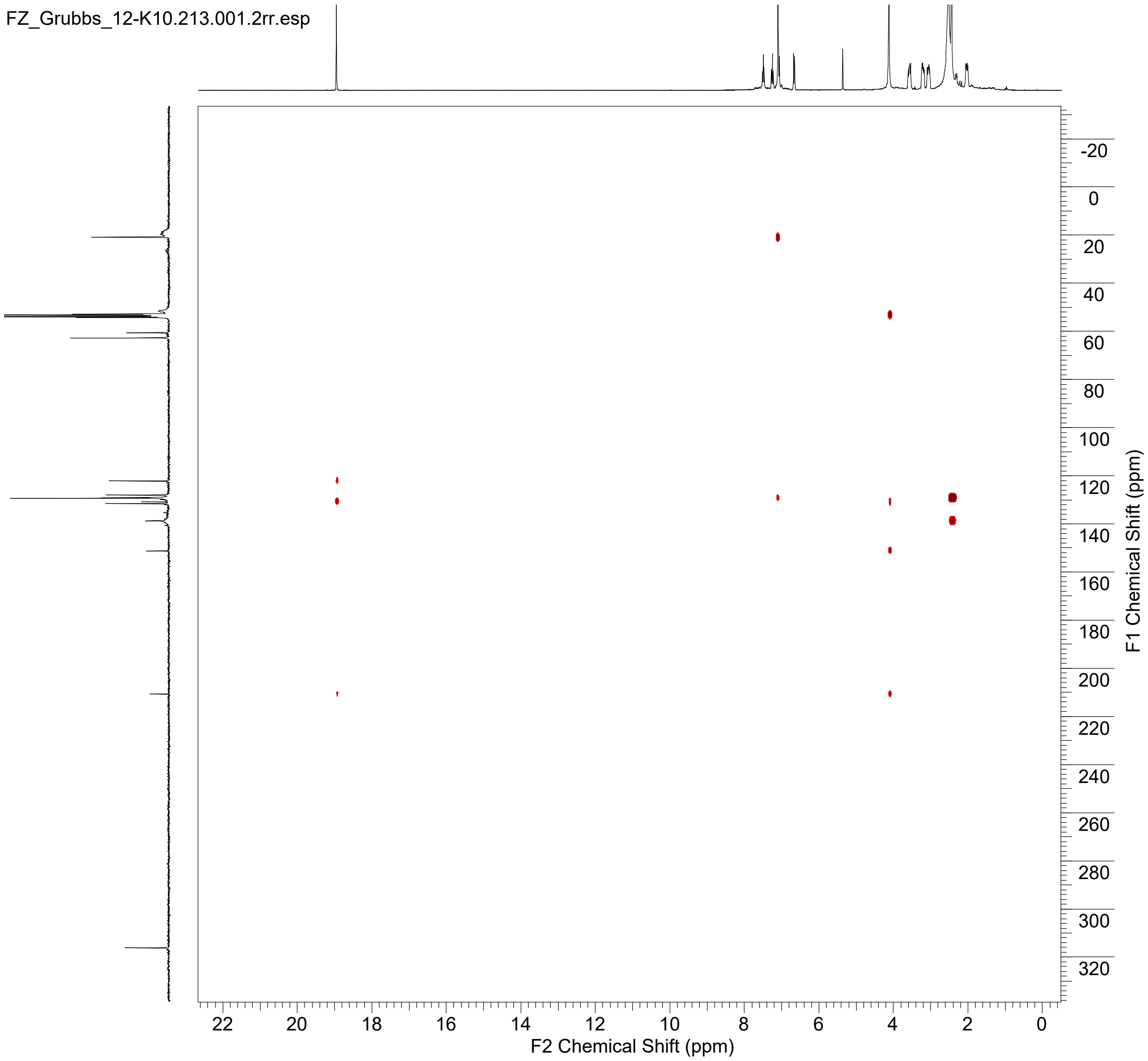


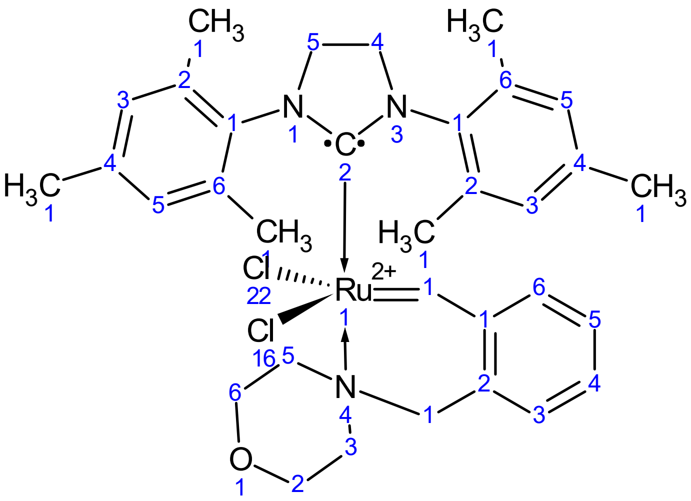

11d

| | | | | | | | |
|---|---|---|---|---|---|---|---|
| **Acquisition Time (sec)** | (0.3408, 0.0108) | **Comment** | 5 mm PABBO BB-1H/D Z-GRD Z104275/0345 | | | **Date** | 02 Oct 2018 20:28:34 |
| **File Name** | C:\Users\Fedor\Desktop\ЯМР Граббс Морфолин\FZ_Grubbs_12-K10\213\pdata\1\2rr | | | | | **Frequency (MHz)** | (300.13, 75.48) |
| **Nucleus** | (1H, 13C) | **Number of Transients** | 4 | **Origin** | spect | **Original Points Count** | (3072, 368) |
| **Owner** | nmr | **Points Count** | (8192, 2048) | **Pulse Sequence** | hmbcgplpndqf | **Solvent** | CD2Cl2 |
| **Sweep Width (Hz)** | (9014.42, 34013.61) | **Temperature (degree C)** | 29.873 | **Title** | FZ_Grubbs_12-K10-HMBC | | |

| | | | |
|---|---|---|---|
| **Formula** | $C_{33}H_{41}Cl_2N_3ORu$ | **FW** | 667.6730 |

FZ_Grubbs_12-K10.213.001.2rr.esp

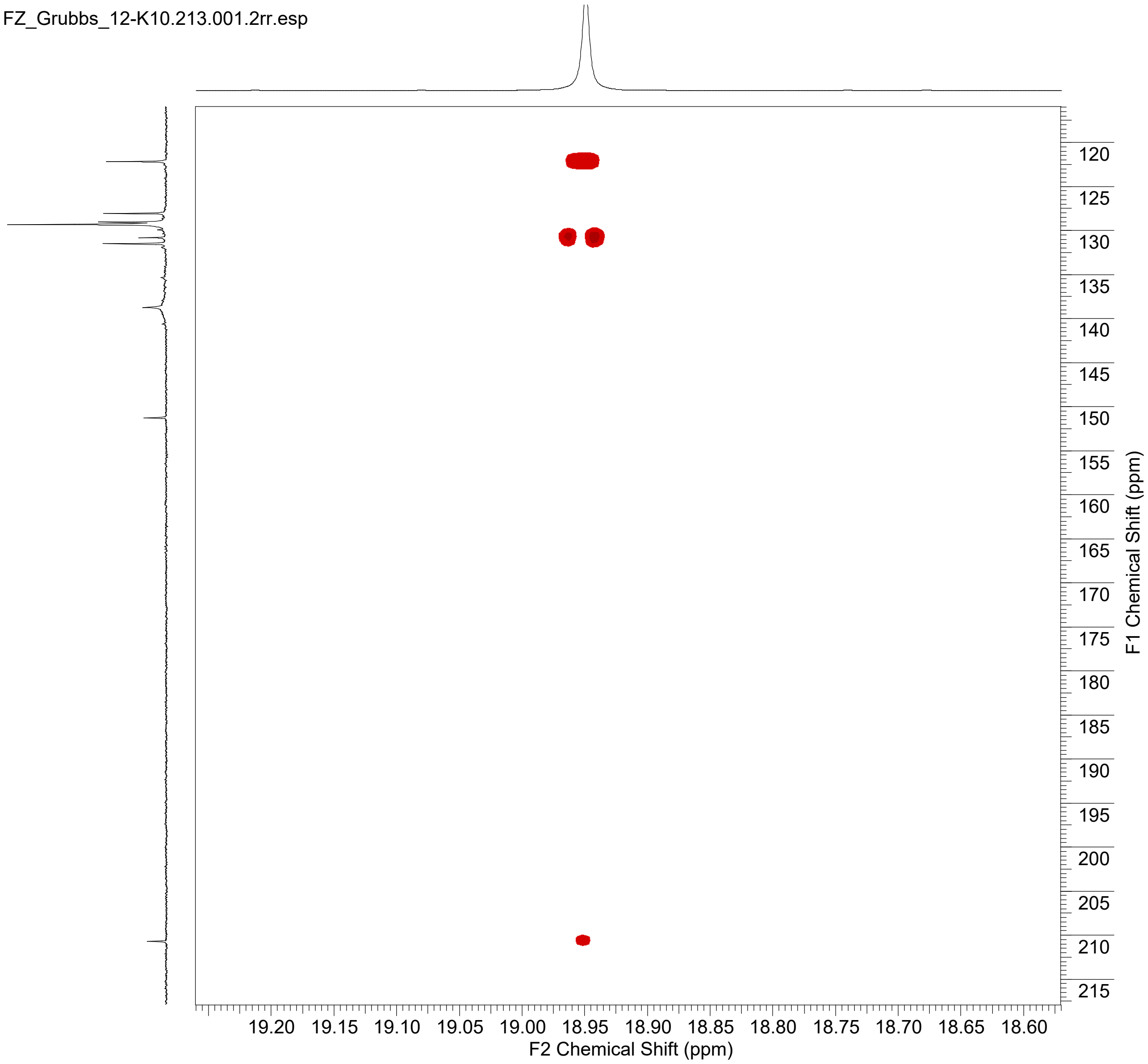


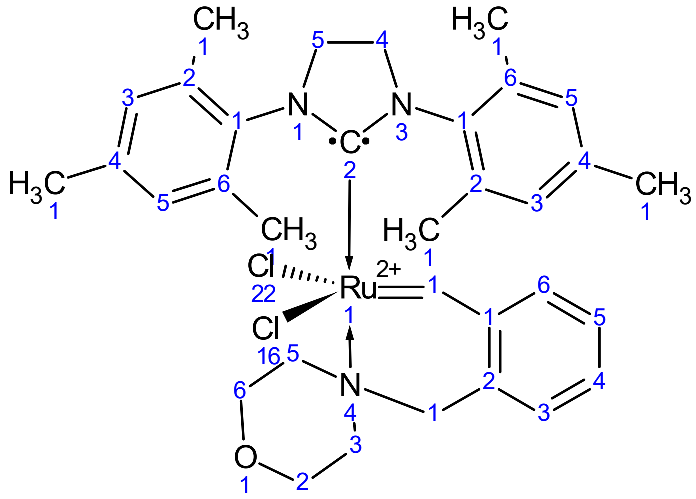

11d

| | | | | | | | |
|---|---|---|---|---|---|---|---|
| ***Acquisition Time (sec)*** | (0.3408, 0.0108) | ***Comment*** | 5 mm PABBO BB-1H/D Z-GRD Z104275/0345 | | | ***Date*** | 02 Oct 2018 20:28:34 |
| ***File Name*** | C:\Users\Fedor\Desktop\ЯМР Граббс Морфолин\FZ_Grubbs_12-K10\213\pdata\1\2rr | | | | | ***Frequency (MHz)*** | (300.13, 75.48) |
| ***Nucleus*** | (1H, 13C) | ***Number of Transients*** | 4 | ***Origin*** | spect | ***Original Points Count*** | (3072, 368) |
| ***Owner*** | nmr | ***Points Count*** | (8192, 2048) | ***Pulse Sequence*** | hmbcgplpndqf | ***Solvent*** | CD2Cl2 |
| ***Sweep Width (Hz)*** | (9014.42, 34013.61) | ***Temperature (degree C)*** | 29.873 | ***Title*** | FZ_Grubbs_12-K10-HMBC | | |

| | | | |
|---|---|---|---|
| ***Formula*** | $C_{33}H_{41}Cl_2N_3ORu$ | ***FW*** | 667.6730 |

FZ_Grubbs_12-K10.213.001.2rr.esp

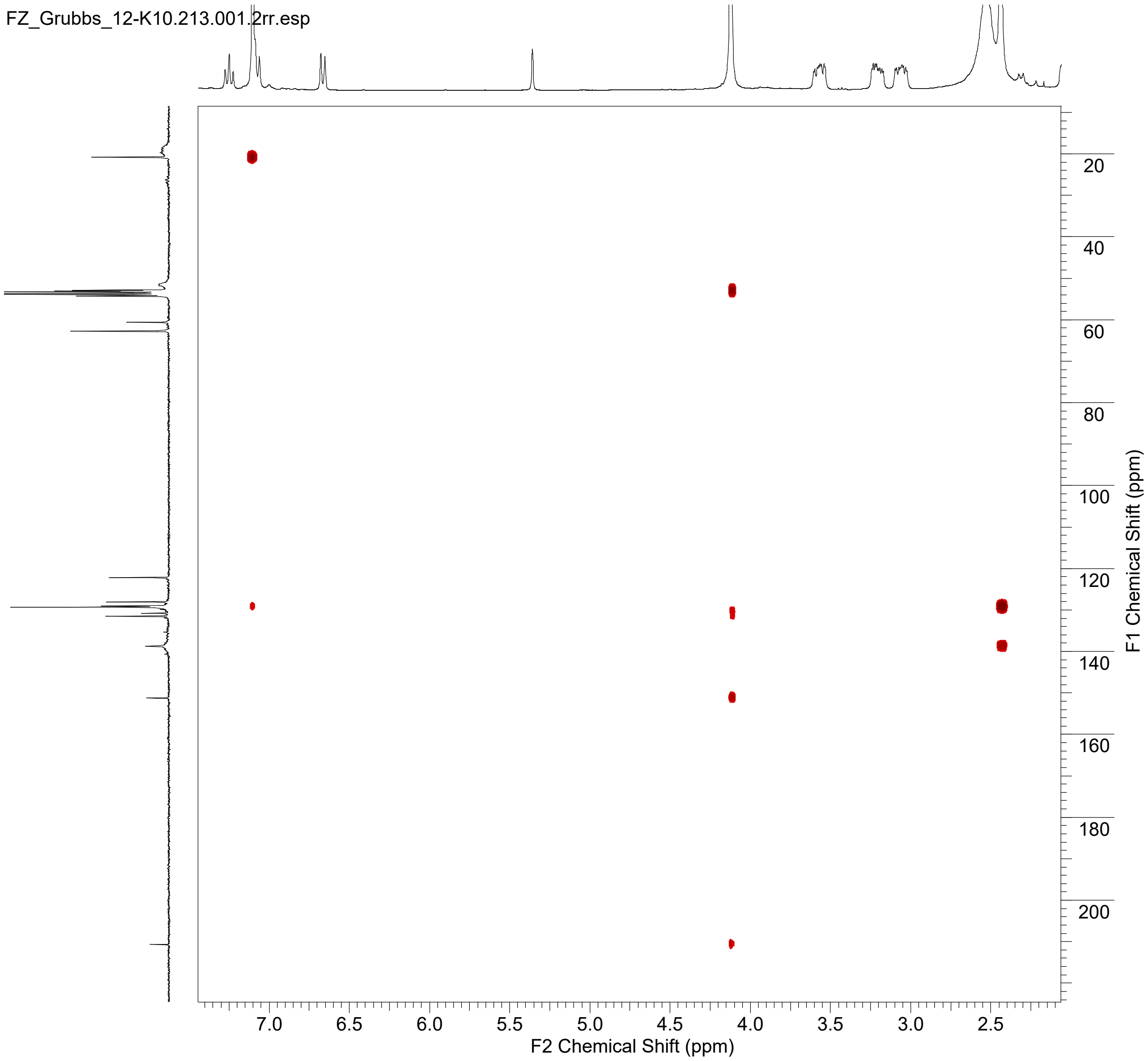


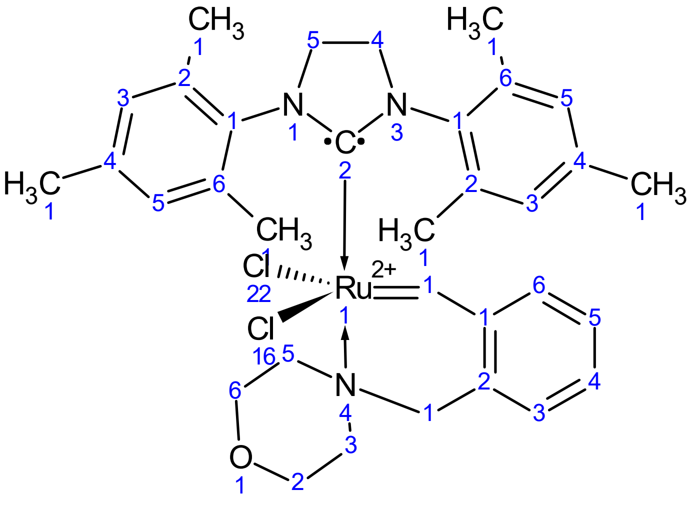

11d

| | | | | | |
|---|---|---|---|---|---|
| ***Acquisition Time (sec)*** | (0.3408, 0.0642) | ***Comment*** | 5 mm PABBO BB-1H/D Z-GRD Z104275/0345 | | |
| ***Date*** | 02 Oct 2018 18:30:04 | | | | |
| ***File Name*** | C:\Users\Fedor\Desktop\Наброски Статей\Кирилл Статья по Катализаторам Граббса\ЯМР Граббс\ЯМР Граббс от Ромы\Grubbs_12-K10 (Морфолиновый)\11\pdata\1\2rr | | | | |
| ***Frequency (MHz)*** | (300.13, 300.13) | ***Nucleus*** | (1H, 1H) | ***Number of Transients*** | 1 |
| ***Origin*** | spect | ***Original Points Count*** | (3072, 578) | ***Owner*** | nmr |
| ***Points Count*** | (8192, 2048) | ***Pulse Sequence*** | cosygpqf | ***Solvent*** | CD2Cl2 |
| ***Sweep Width (Hz)*** | (9014.42, 9009.01) | ***Temperature (degree C)*** | 29.656 | ***Title*** | FZ_Grubbs_12-K10-COSY |

| | |
|---|---|
| ***Formula*** $C_{33}H_{41}Cl_2N_3ORu$ | ***FW*** 667.6730 |

Grubbs_12-K10 (Морфолиновый).011.001.2rr.esp

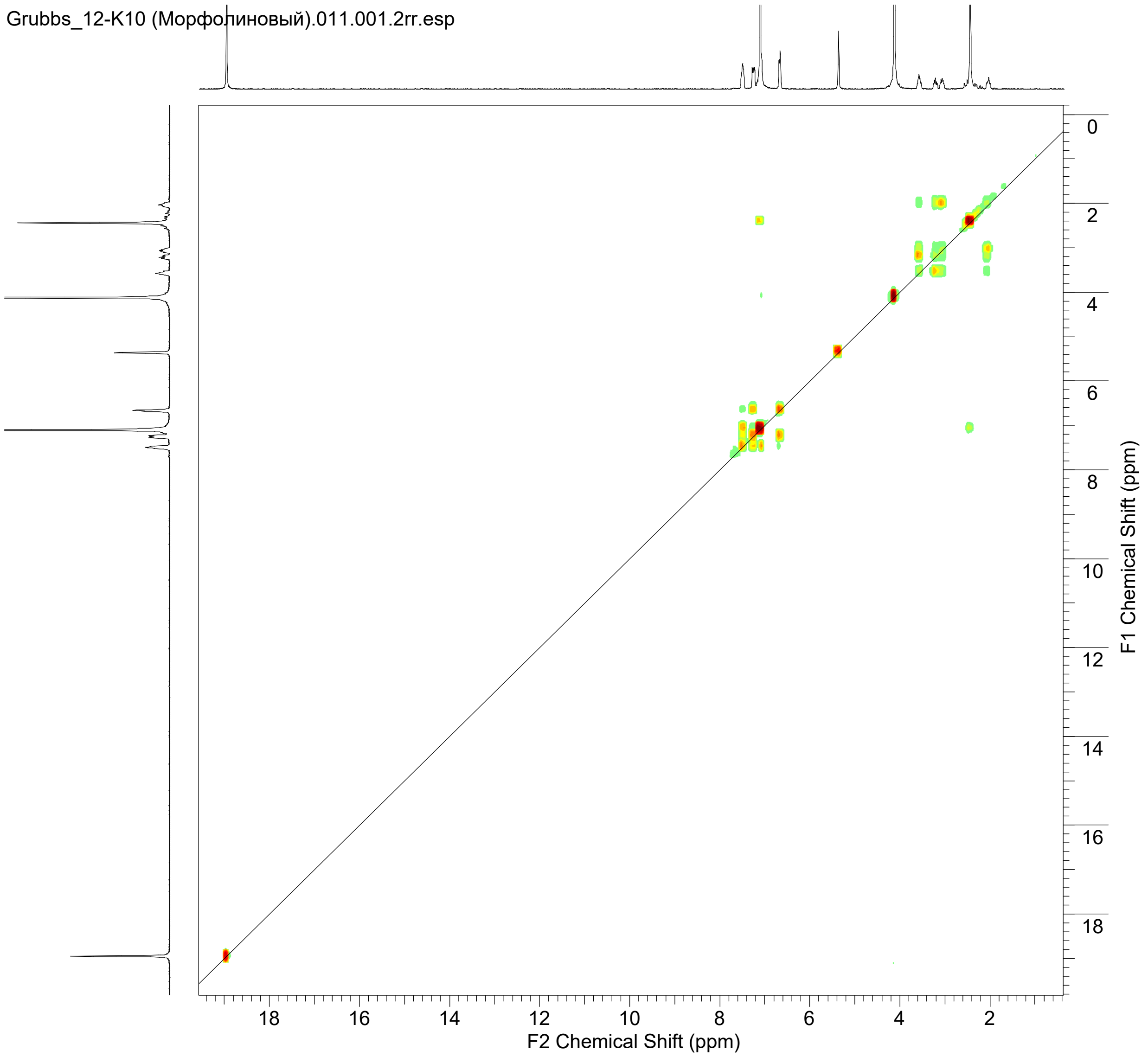


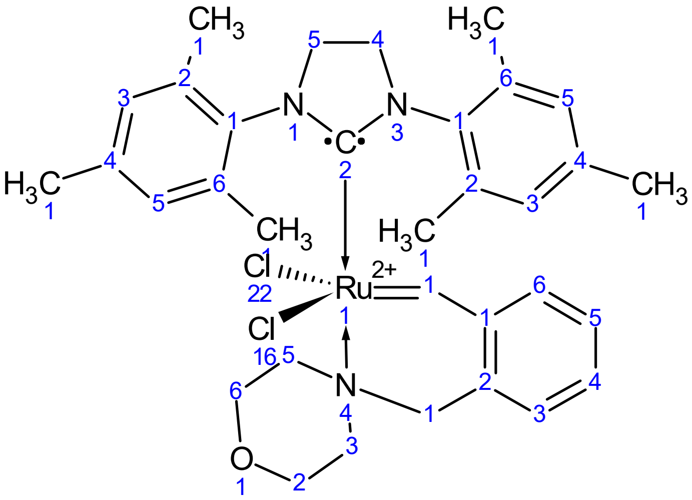

11d

| | | | | | |
|---|---|---|---|---|---|
| **Acquisition Time (sec)** | (0.3408, 0.0642) | **Comment** | 5 mm PABBO BB-1H/D Z-GRD Z104275/0345 | | |
| **Date** | 02 Oct 2018 18:30:04 | | | | |
| **File Name** | C:\Users\Fedor\Desktop\Наброски Статей\Кирилл Статья по Катализаторам Граббса\ЯМР Граббс\ЯМР Граббс от Ромы\Grubbs_12-K10 (Морфолиновый)\11\pdata\1\2rr | | | | |
| **Frequency (MHz)** | (300.13, 300.13) | **Nucleus** | (1H, 1H) | **Number of Transients** | 1 |
| **Origin** | spect | **Original Points Count** | (3072, 578) | **Owner** | nmr |
| **Points Count** | (8192, 2048) | **Pulse Sequence** | cosygpqf | **Solvent** | CD2Cl2 |
| **Sweep Width (Hz)** | (9014.42, 9009.01) | **Temperature (degree C)** | 29.656 | **Title** | FZ_Grubbs_12-K10-COSY |

| | | | |
|---|---|---|---|
| **Formula** | $C_{33}H_{41}Cl_2N_3ORu$ | **FW** | 667.6730 |

Grubbs_12-K10 (Морфолиновый).011.001.2rr.esp

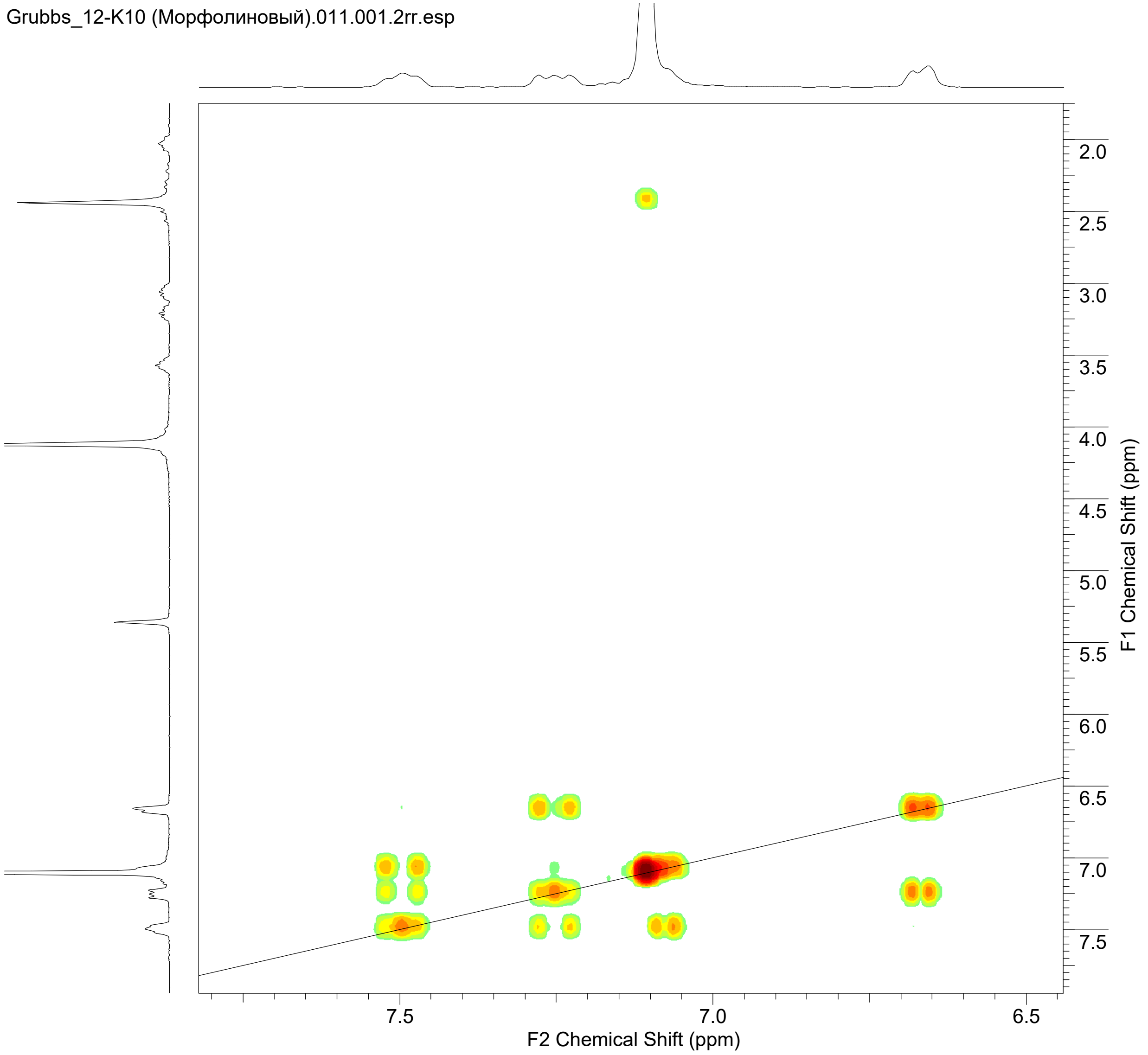


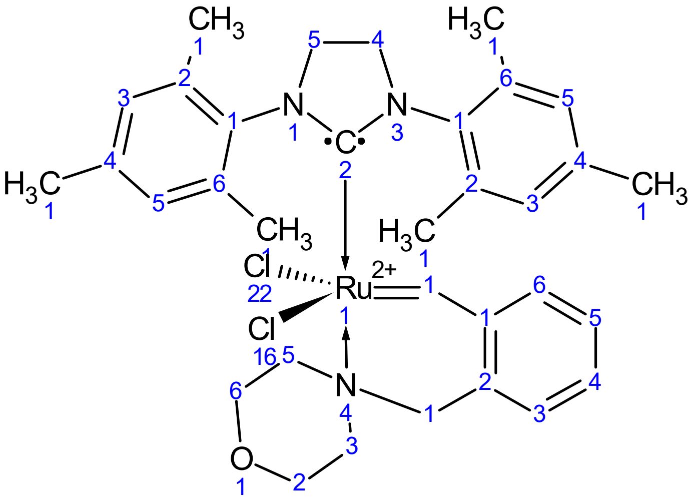

11d

| | | | | | |
|---|---|---|---|---|---|
| ***Acquisition Time (sec)*** | (0.3408, 0.0642) | ***Comment*** | 5 mm PABBO BB-1H/D Z-GRD Z104275/0345 | | |
| ***Date*** | 02 Oct 2018 18:30:04 | | | | |
| ***File Name*** | C:\Users\Fedor\Desktop\Наброски Статей\Кирилл Статья по Катализаторам Граббса\ЯМР Граббс\ЯМР Граббс от Ромы\Grubbs_12-K10 (Морфолиновый)\11\pdata\1\2rr | | | | |
| ***Frequency (MHz)*** | (300.13, 300.13) | ***Nucleus*** | (1H, 1H) | ***Number of Transients*** | 1 |
| ***Origin*** | spect | ***Original Points Count*** | (3072, 578) | ***Owner*** | nmr |
| ***Points Count*** | (8192, 2048) | ***Pulse Sequence*** | cosygpqf | ***Solvent*** | CD2Cl2 |
| ***Sweep Width (Hz)*** | (9014.42, 9009.01) | ***Temperature (degree C)*** | 29.656 | ***Title*** | FZ_Grubbs_12-K10-COSY |

| | |
|---|---|
| ***Formula*** $C_{33}H_{41}Cl_2N_3ORu$ | ***FW*** 667.6730 |

Grubbs_12-K10 (Морфолиновый).011.001.2rr.esp

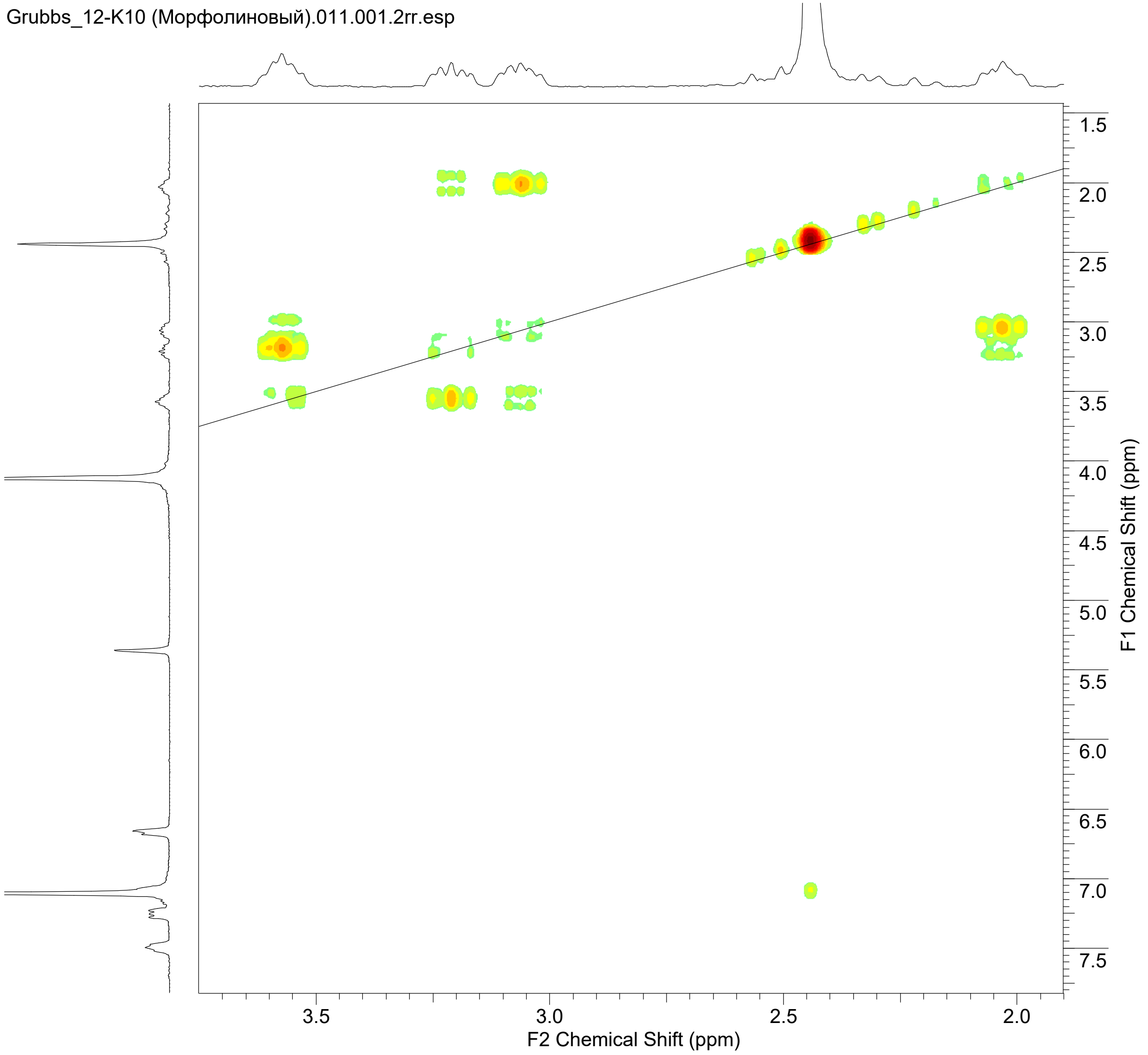


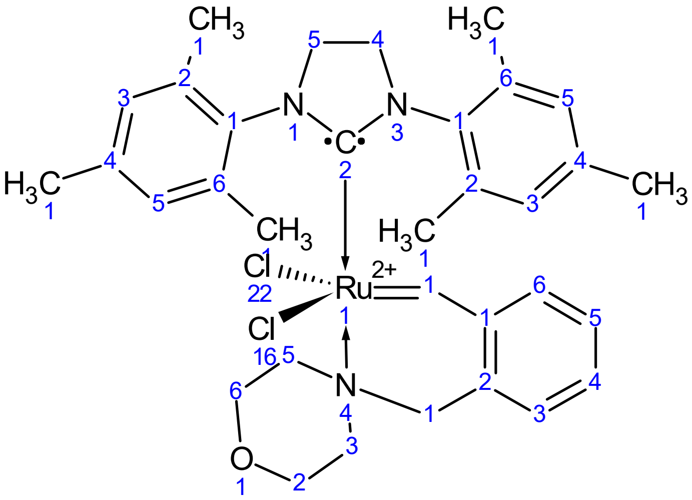

11d

| | | | | | | | |
|---|---|---|---|---|---|---|---|
| ***Acquisition Time (sec)*** | 1.9818 | ***Comment*** | single_pulse | ***Date*** | 10 Jan 1990 12:48:43 | ***Date Stamp*** | 13 Dec 2018 08:32:19 |
| ***File Name*** | C:\Users\Fedor\Desktop\11.12.18\FZ7080-1.jdf | | | ***Frequency (MHz)*** | 600.17 | ***Nucleus*** 1H | ***Number of Transients*** 8 |
| ***Origin*** | ECA 600 | ***Original Points Count*** | 32768 | ***Owner*** | delta | ***Points Count*** 32768 | ***Pulse Sequence*** single_pulse.ex2 |
| ***Receiver Gain*** | 38.00 | ***Solvent*** | CHLOROFORM-d | | | ***Spectrum Offset (Hz)*** 5401.5503 | ***Sweep Width (Hz)*** 16534.39 |

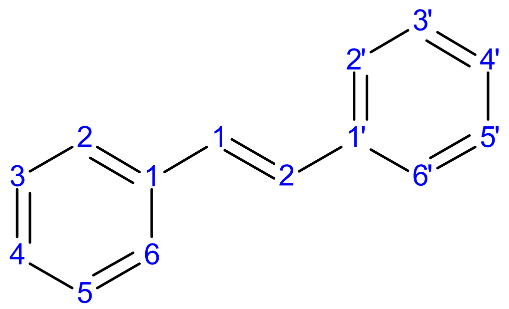


13

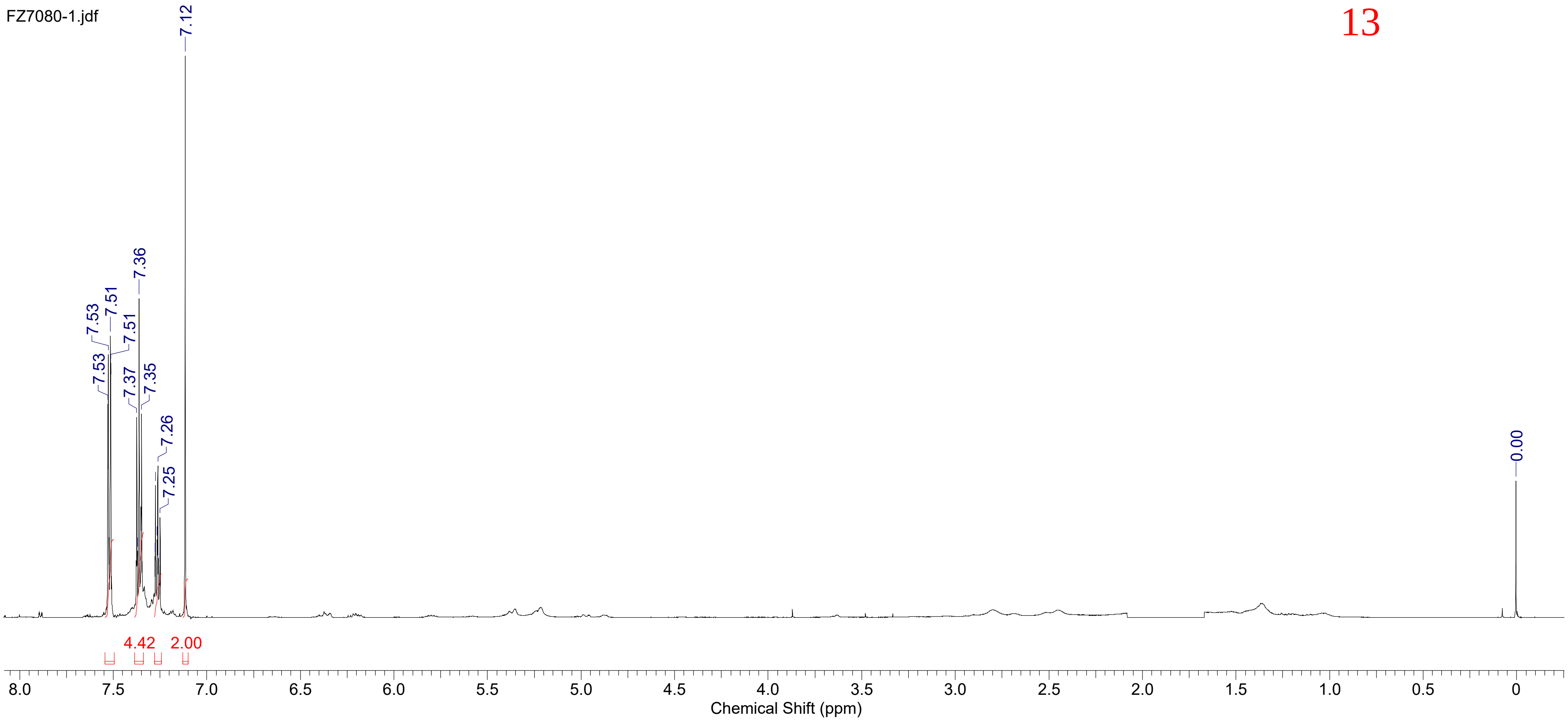

| | | | | | | | |
|---|---|---|---|---|---|---|---|
| ***Acquisition Time (sec)*** | 1.9818 | ***Comment*** | single_pulse | ***Date*** | 10 Jan 1990 12:48:43 | ***Date Stamp*** | 13 Dec 2018 08:32:19 |
| ***File Name*** | C:\Users\Fedor\Desktop\11.12.18\FZ7080-1.jdf | | | ***Frequency (MHz)*** | 600.17 | ***Nucleus*** | 1H |
| ***Number of Transients*** | 8 | ***Origin*** | ECA 600 | ***Original Points Count*** | 32768 | ***Owner*** | delta |
| ***Points Count*** | 32768 | ***Pulse Sequence*** | single_pulse.ex2 | ***Receiver Gain*** | 38.00 | ***Solvent*** | CHLOROFORM-d |
| ***Spectrum Offset (Hz)*** | 5401.5503 | ***Sweep Width (Hz)*** | 16534.39 | | | | |

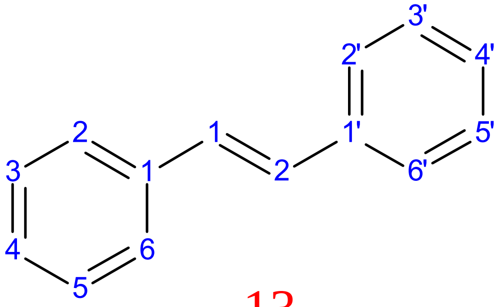


13

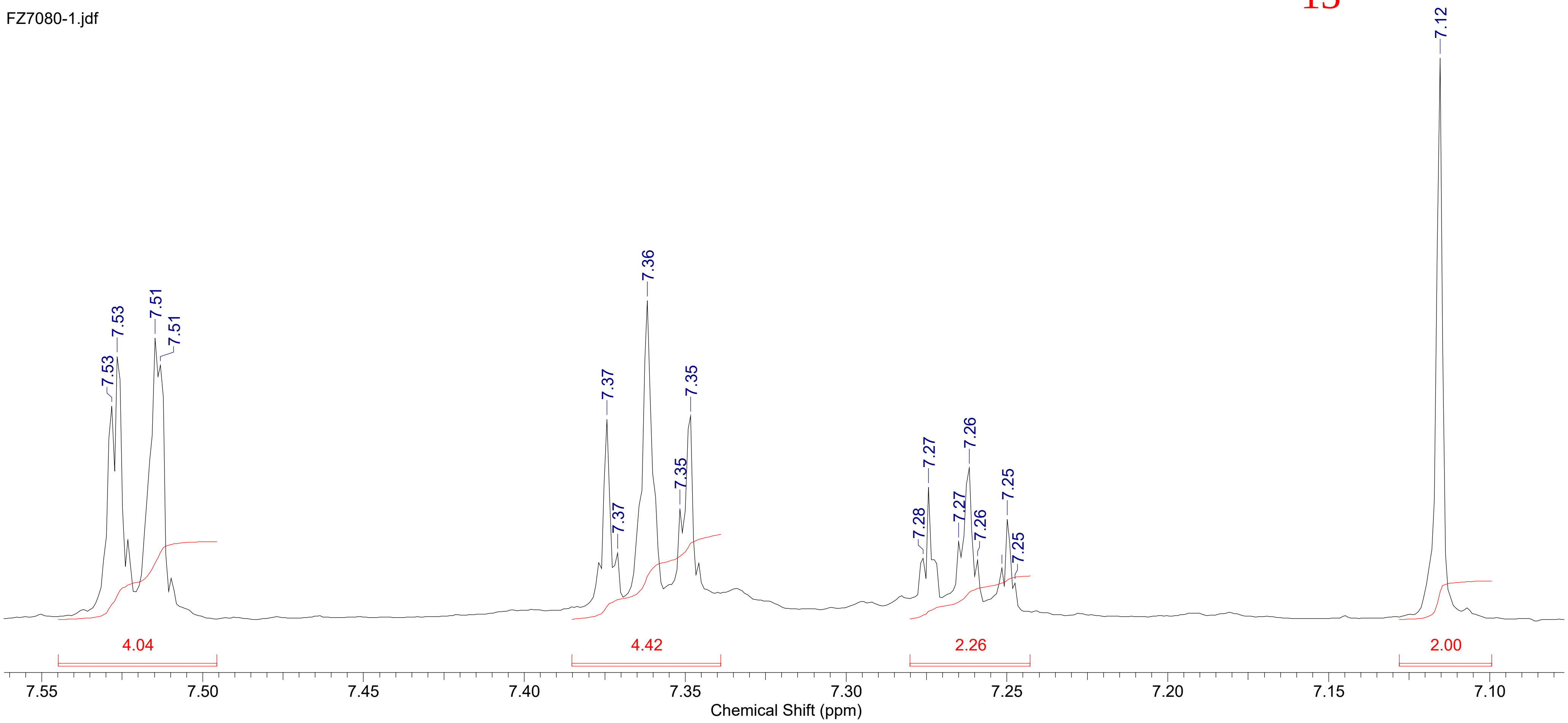

| | | | | | | | |
|---|---|---|---|---|---|---|---|
| ***Acquisition Time (sec)*** | 0.6921 | ***Comment*** | single pulse decoupled gated NOE | ***Date*** | 10 Jan 1990 12:57:45 | | |
| ***Date Stamp*** | 13 Dec 2018 08:41:21 | ***File Name*** | C:\Users\Fedor\Desktop\11.12.18\FZ7080-2.jdf | ***Frequency (MHz)*** | 150.91 | | |
| ***Nucleus*** | 13C | ***Number of Transients*** | 400 | ***Origin*** | ECA 600 | ***Original Points Count*** | 32768 |
| ***Owner*** | delta | ***Points Count*** | 32768 | ***Pulse Sequence*** | single_pulse_dec | ***Receiver Gain*** | 56.00 |
| ***Solvent*** | CHLOROFORM-d | ***Spectrum Offset (Hz)*** | 15091.3428 | ***Sweep Width (Hz)*** | 47348.49 | | |

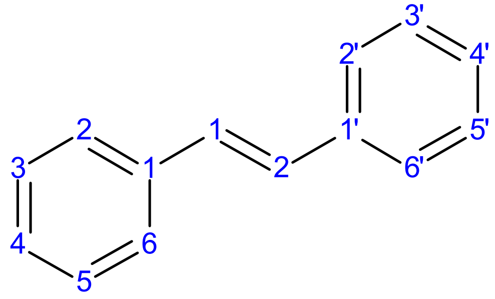


13

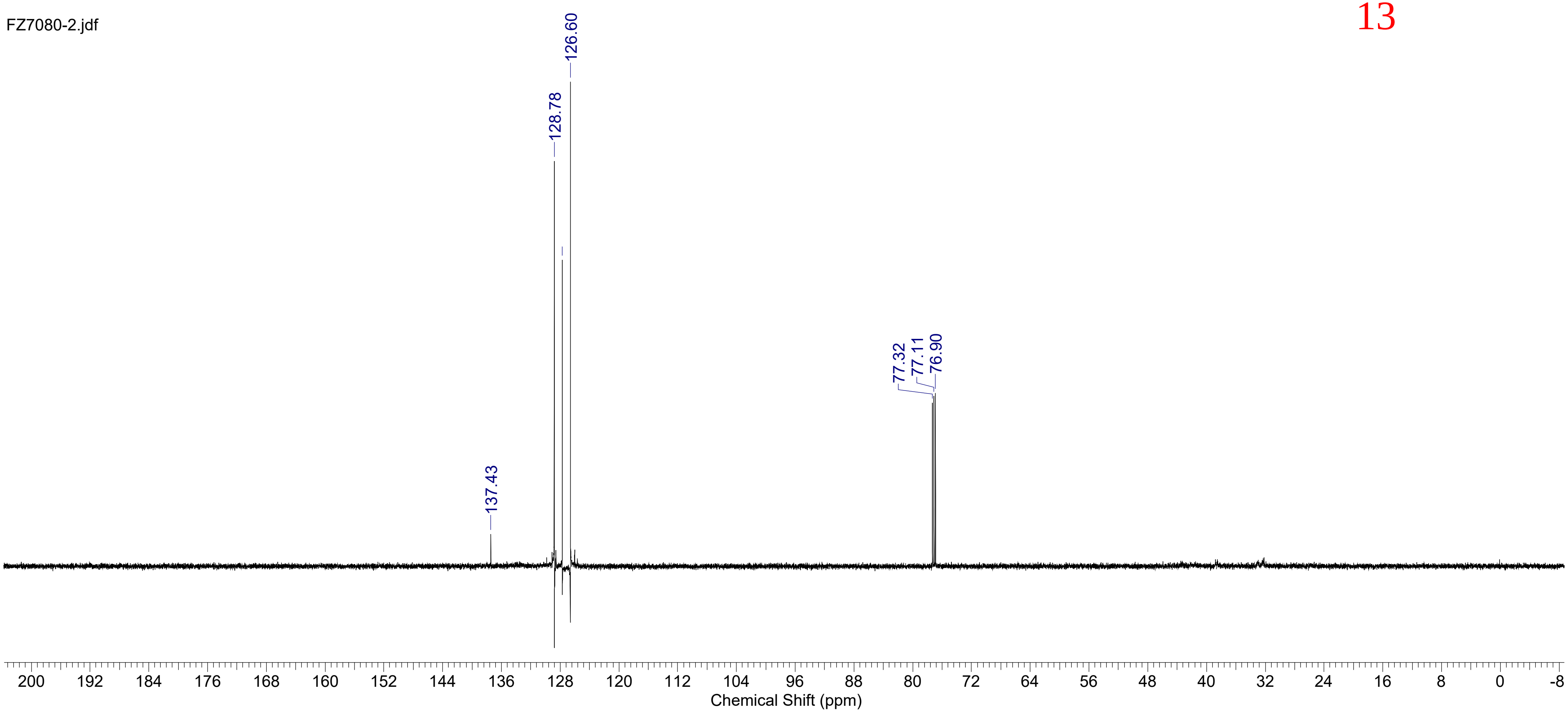

| | | | |
|---|---|---|---|
| **Acquisition Time (sec)** | 0.6921 | **Comment** | single pulse decoupled gated NOE |
| **Date** | 10 Jan 1990 12:57:45 | **Date Stamp** | 13 Dec 2018 08:41:21 |
| **File Name** | C:\Users\Fedor\Desktop\11.12.18\FZ7080-2.jdf | **Frequency (MHz)** | 150.91 |
| **Nucleus** | 13C | **Number of Transients** | 400 |
| **Origin** | ECA 600 | **Original Points Count** | 32768 |
| **Owner** | delta | **Points Count** | 32768 |
| **Pulse Sequence** | single_pulse_dec | **Receiver Gain** | 56.00 |
| **Solvent** | CHLOROFORM-d | **Spectrum Offset (Hz)** | 15091.3428 |
| **Sweep Width (Hz)** | 47348.49 | | |

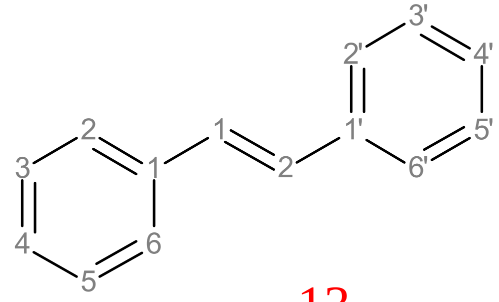


13

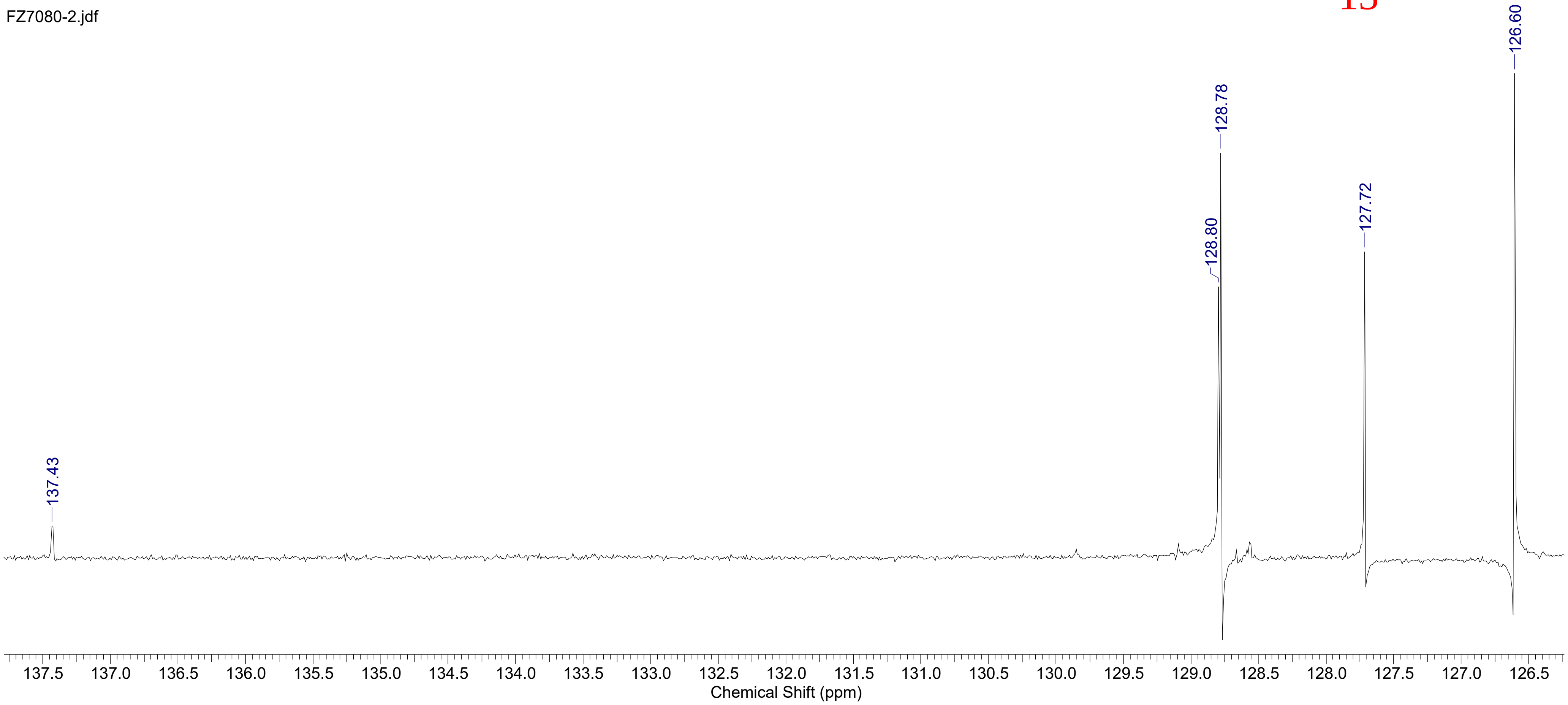

| Acquisition Time (sec) | 1.9818 | Comment | single_pulse | Date | 10 Jan 1990 12:19:53 | | | Date Stamp | 13 Dec 2018 08:03:29 |
|---|---|---|---|---|---|---|---|---|---|
| File Name | C:\Users\Fedor\Desktop\11.12.18\FZ7061-1.jdf | | | Frequency (MHz) | 600.17 | Nucleus | 1H | Number of Transients | 8 |
| Origin | ECA 600 | Original Points Count | 32768 | Owner | delta | Points Count | 32768 | Pulse Sequence | single_pulse.ex2 |
| Receiver Gain | 46.00 | Solvent | CHLOROFORM-d | | | Spectrum Offset (Hz) | 5401.5503 | Sweep Width (Hz) | 16534.39 |

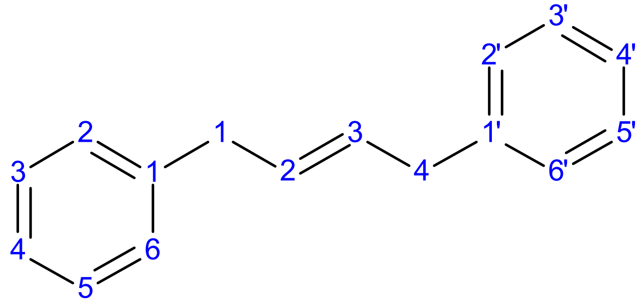


15

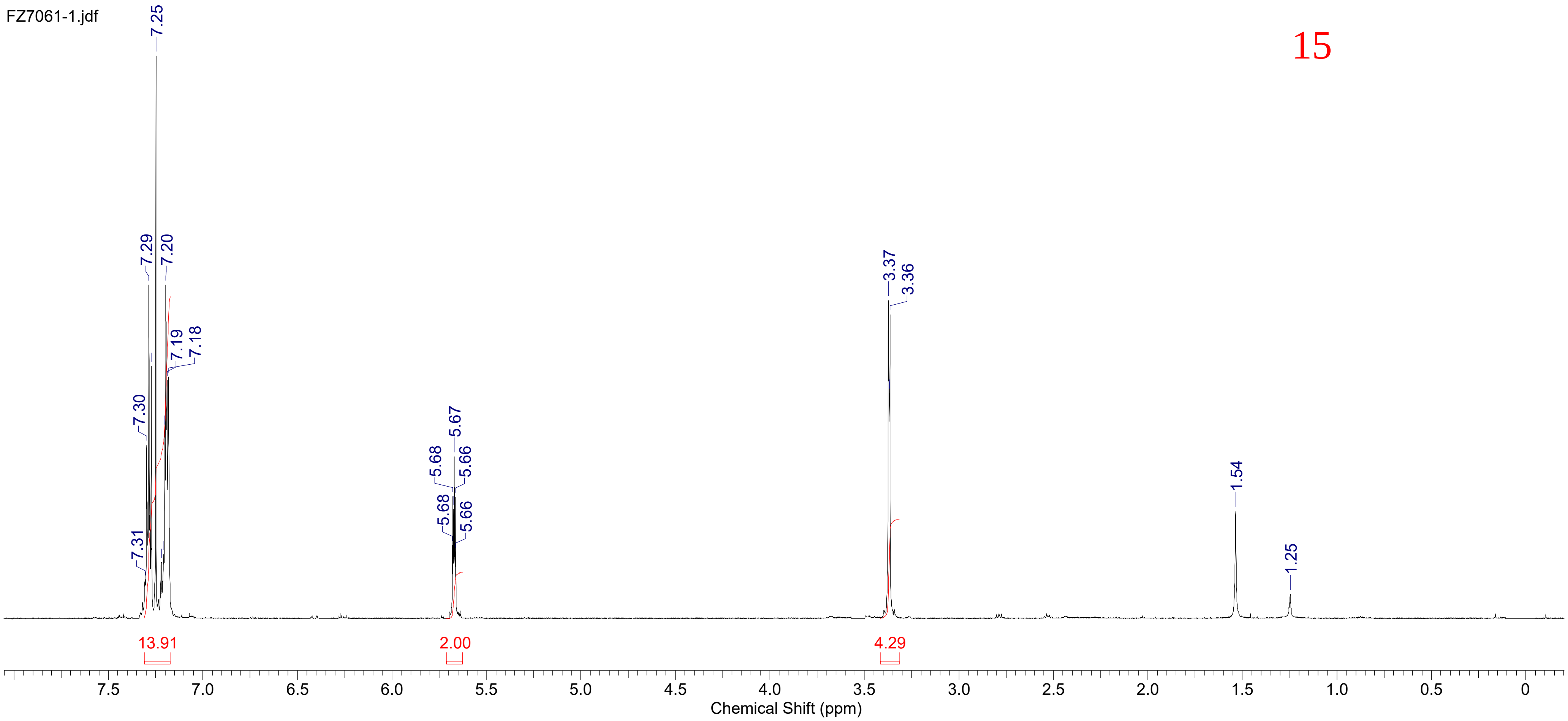

| Acquisition Time (sec) | 1.9818 | Comment | single_pulse | Date | 10 Jan 1990 12:19:53 | | | Date Stamp | 13 Dec 2018 08:03:29 |
|---|---|---|---|---|---|---|---|---|---|
| File Name | C:\Users\Fedor\Desktop\11.12.18\FZ7061-1.jdf | | | Frequency (MHz) | 600.17 | Nucleus | 1H | Number of Transients | 8 |
| Origin | ECA 600 | Original Points Count | 32768 | Owner | delta | Points Count | 32768 | Pulse Sequence | single_pulse.ex2 |
| Receiver Gain | 46.00 | Solvent | CHLOROFORM-d | | | Spectrum Offset (Hz) | 5401.5503 | Sweep Width (Hz) | 16534.39 |

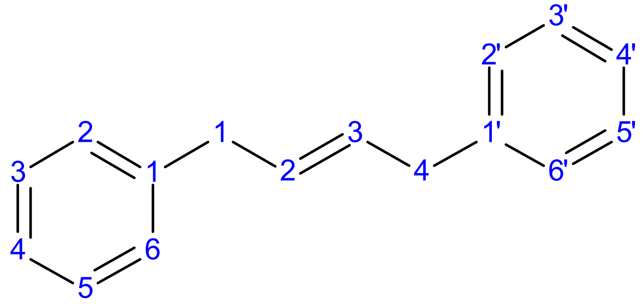


15

FZ7061-1.jdf

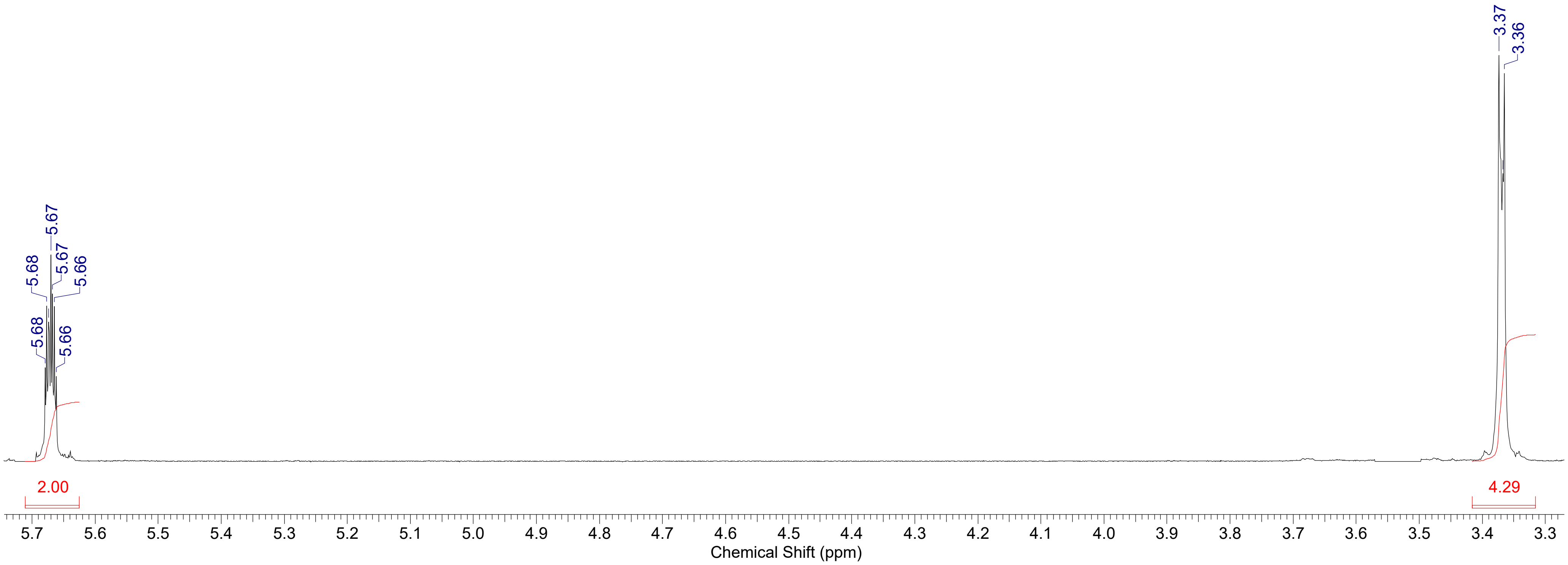

| | | | | | | | |
|---|---|---|---|---|---|---|---|
| ***Acquisition Time (sec)*** | 0.6921 | ***Comment*** | single pulse decoupled gated NOE | | ***Date*** | 10 Jan 1990 12:28:52 | |
| ***Date Stamp*** | 13 Dec 2018 08:12:28 | | ***File Name*** | C:\Users\Fedor\Desktop\11.12.18\FZ7061-2.jdf | | ***Frequency (MHz)*** | 150.91 |
| ***Nucleus*** | 13C | ***Number of Transients*** 400 | ***Origin*** | ECA 600 | ***Original Points Count*** 32768 | ***Owner*** | delta |
| ***Points Count*** | 32768 | ***Pulse Sequence*** | single_pulse_dec | | ***Receiver Gain*** 56.00 | ***Solvent*** | CHLOROFORM-d |
| ***Spectrum Offset (Hz)*** | 15091.3428 | ***Sweep Width (Hz)*** | 47348.49 | | | | |

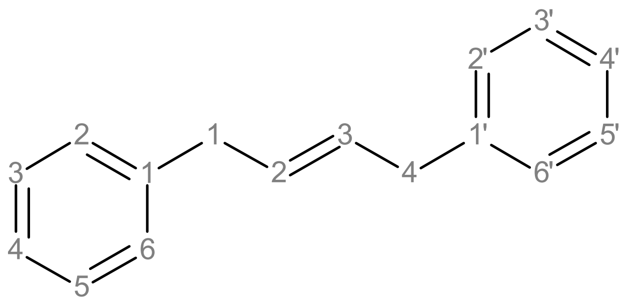


15

FZ7061-2.esp

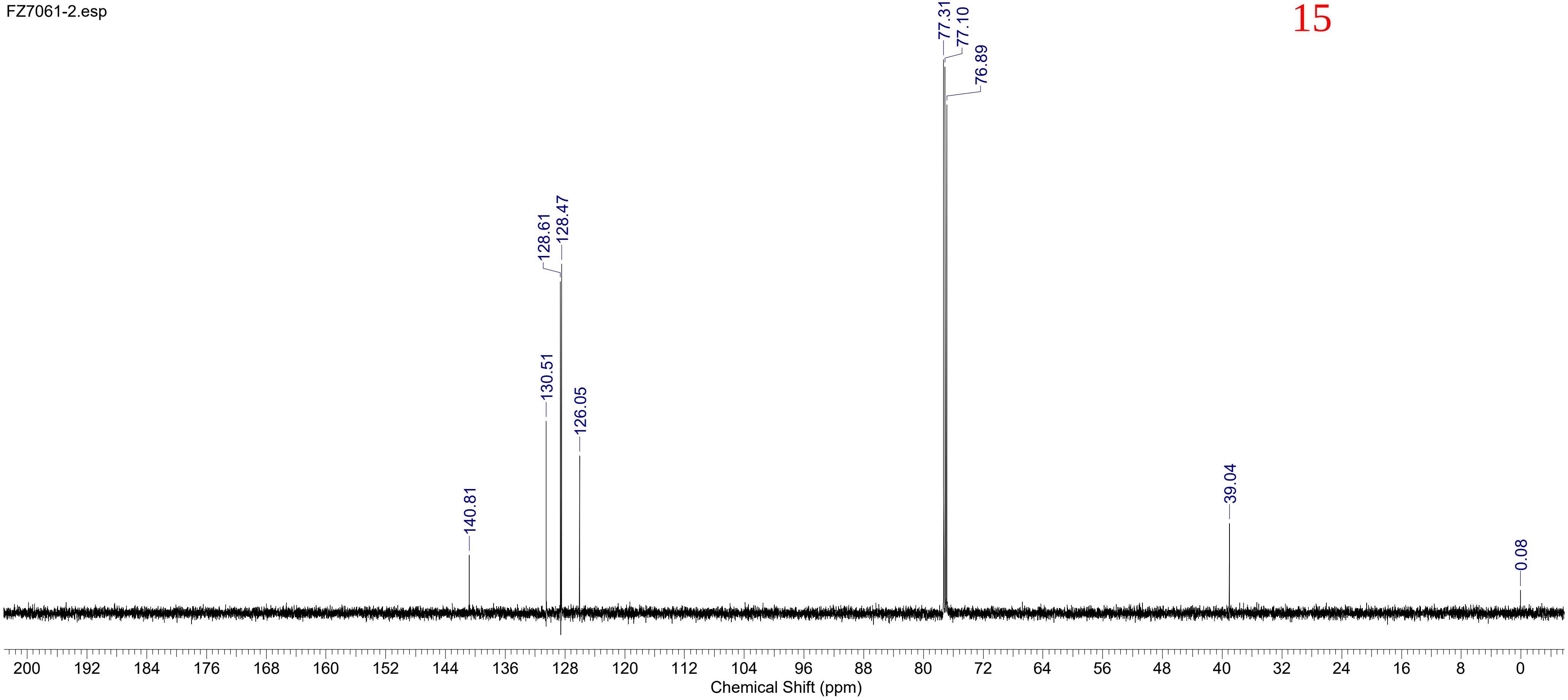

| | | | | | | | | | |
|---|---|---|---|---|---|---|---|---|---|
| ***Acquisition Time (sec)*** | 0.6921 | ***Comment*** | single pulse decoupled gated NOE | | | ***Date*** | 10 Jan 1990 12:28:52 | | |
| ***Date Stamp*** | 13 Dec 2018 08:12:28 | | | ***File Name*** | C:\Users\Fedor\Desktop\11.12.18\FZ7061-2.jdf | | | ***Frequency (MHz)*** | 150.91 |
| ***Nucleus*** | 13C | ***Number of Transients*** | 400 | ***Origin*** | ECA 600 | ***Original Points Count*** | 32768 | ***Owner*** | delta |
| ***Points Count*** | 32768 | ***Pulse Sequence*** | single_pulse_dec | | | ***Receiver Gain*** | 56.00 | ***Solvent*** | CHLOROFORM-d |
| ***Spectrum Offset (Hz)*** | 15091.3428 | ***Sweep Width (Hz)*** | 47348.49 | | | | | | |

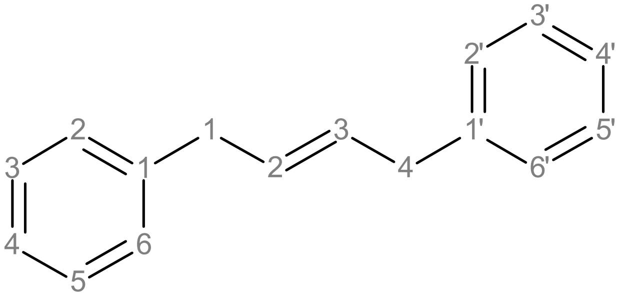


15

FZ7061-2.esp

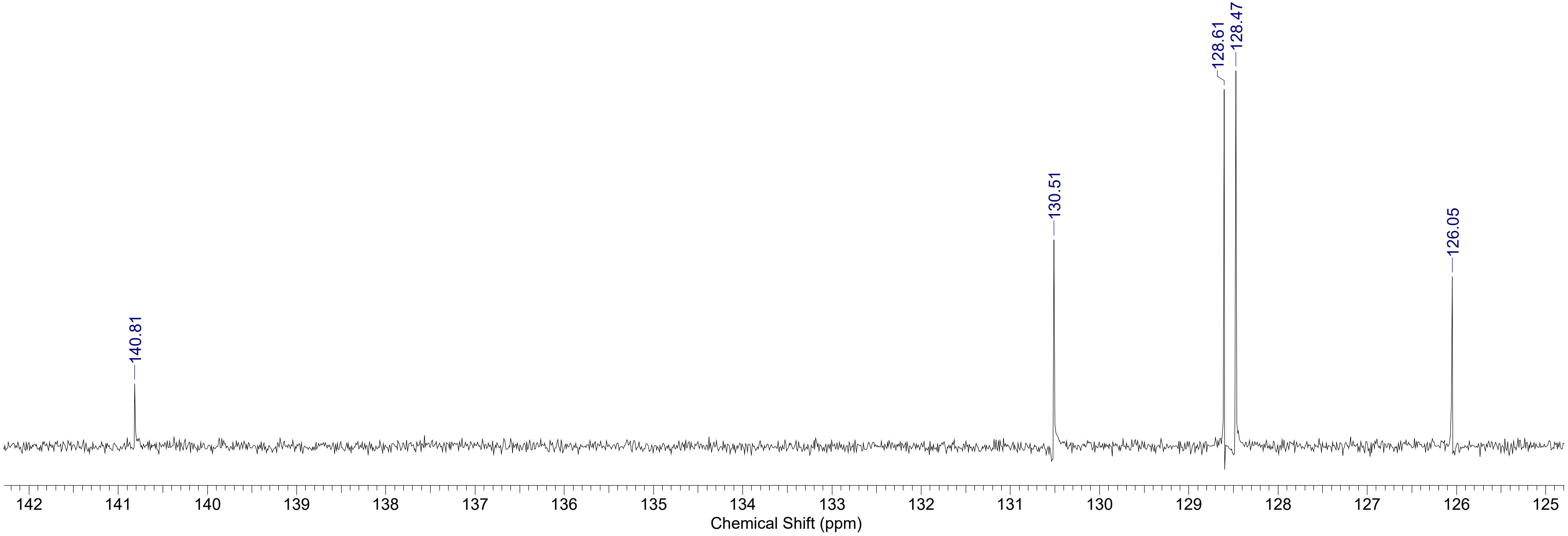

| | | | | | | | |
|---|---|---|---|---|---|---|---|
| ***Acquisition Time (sec)*** | 1.9818 | ***Comment*** | single_pulse | ***Date*** | 10 Jan 1990 11:52:50 | ***Date Stamp*** | 13 Dec 2018 07:36:26 |
| ***File Name*** | C:\Users\Fedor\Desktop\11.12.18\FZ7063-1.jdf | ***Frequency (MHz)*** | 600.17 | ***Nucleus*** | 1H | ***Number of Transients*** | 8 |
| ***Origin*** | ECA 600 | ***Original Points Count*** | 32768 | ***Owner*** | delta | ***Points Count*** | 32768 |
| ***Pulse Sequence*** | single_pulse.ex2 | ***Receiver Gain*** | 28.00 | ***Solvent*** | CHLOROFORM-d | ***Spectrum Offset (Hz)*** | 5401.5503 |
| ***Sweep Width (Hz)*** | 16534.39 | | | | | | |

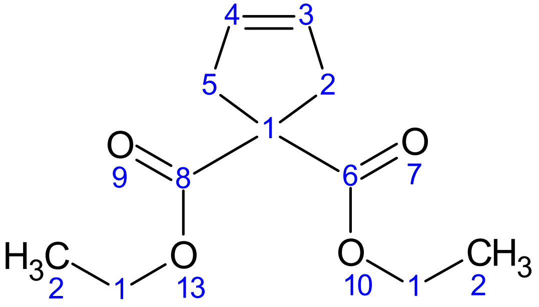

FZ7063-1.jdf

18

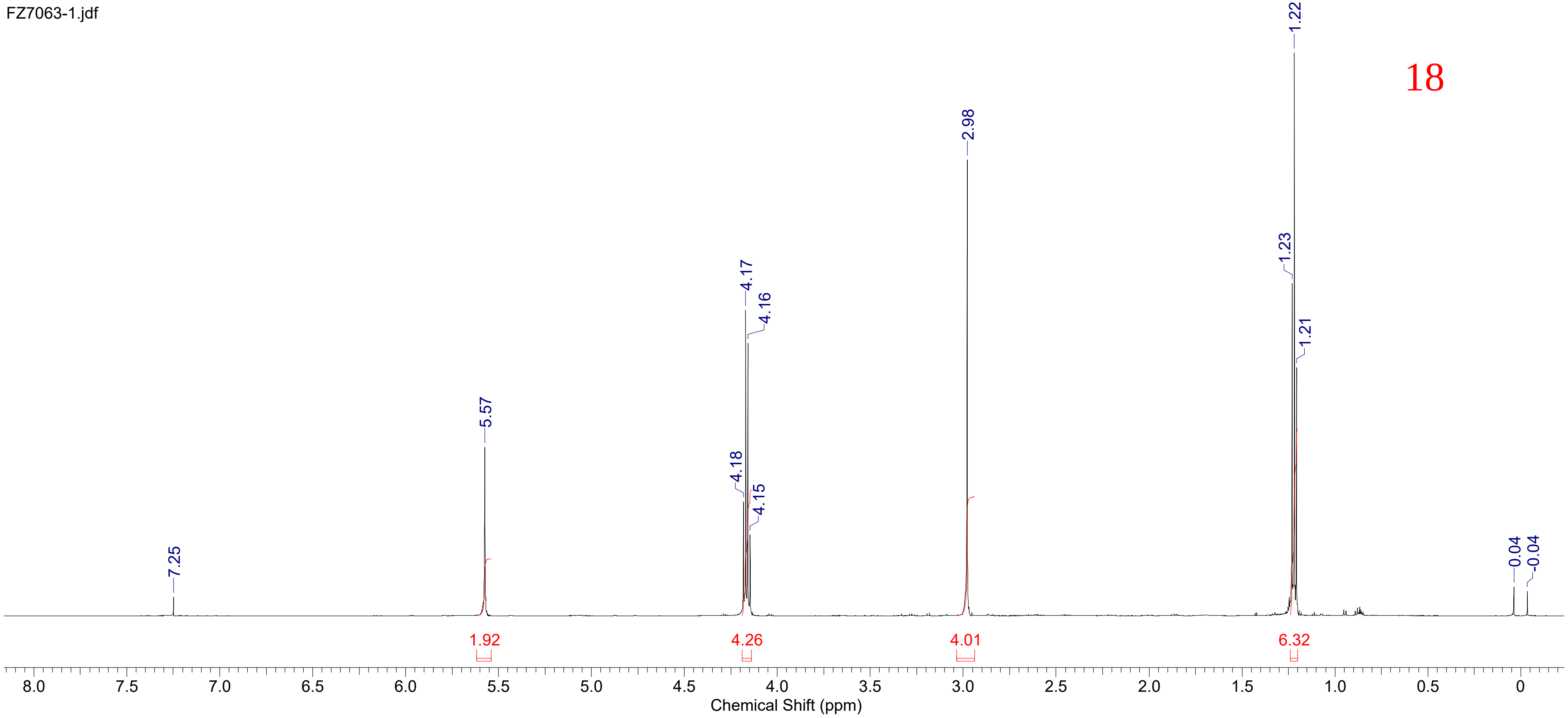

| | | | | | | | |
|---|---|---|---|---|---|---|---|
| ***Acquisition Time (sec)*** | 1.9818 | ***Comment*** | single_pulse | ***Date*** | 10 Jan 1990 11:52:50 | ***Date Stamp*** | 13 Dec 2018 07:36:26 |
| ***File Name*** | C:\Users\Fedor\Desktop\11.12.18\FZ7063-1.jdf | ***Frequency (MHz)*** | 600.17 | ***Nucleus*** | 1H | ***Number of Transients*** | 8 |
| ***Origin*** | ECA 600 | ***Original Points Count*** | 32768 | ***Owner*** | delta | ***Points Count*** | 32768 |
| ***Pulse Sequence*** | single_pulse.ex2 | ***Receiver Gain*** | 28.00 | ***Solvent*** | CHLOROFORM-d | ***Spectrum Offset (Hz)*** | 5401.5503 |
| ***Sweep Width (Hz)*** | 16534.39 | | | | | | |

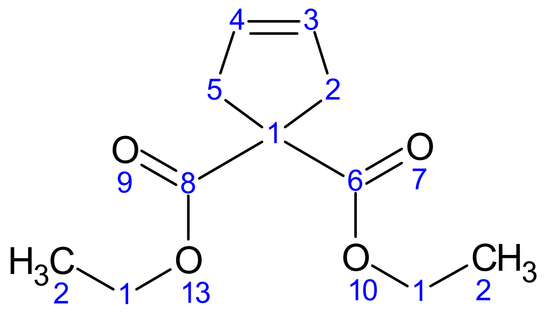


FZ7063-1.jdf

18

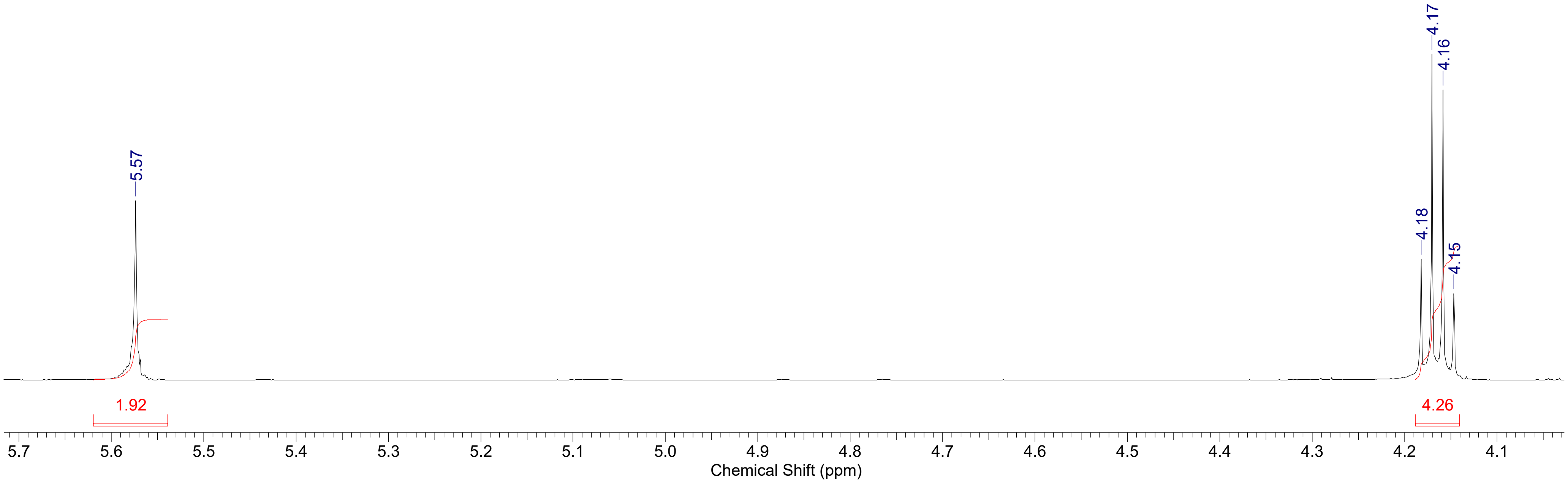

| | | | | | | | | | |
|---|---|---|---|---|---|---|---|---|---|
| ***Acquisition Time (sec)*** | 1.9818 | ***Comment*** | single_pulse | ***Date*** | 10 Jan 1990 11:52:50 | | | ***Date Stamp*** | 13 Dec 2018 07:36:26 |
| ***File Name*** | C:\Users\Fedor\Desktop\11.12.18\FZ7063-1.jdf | | | ***Frequency (MHz)*** | 600.17 | ***Nucleus*** | 1H | ***Number of Transients*** | 8 |
| ***Origin*** | ECA 600 | ***Original Points Count*** | 32768 | ***Owner*** | delta | ***Points Count*** | 32768 | ***Pulse Sequence*** | single_pulse.ex2 |
| ***Receiver Gain*** | 28.00 | ***Solvent*** | CHLOROFORM-d | | | ***Spectrum Offset (Hz)*** | 5401.5503 | ***Sweep Width (Hz)*** | 16534.39 |

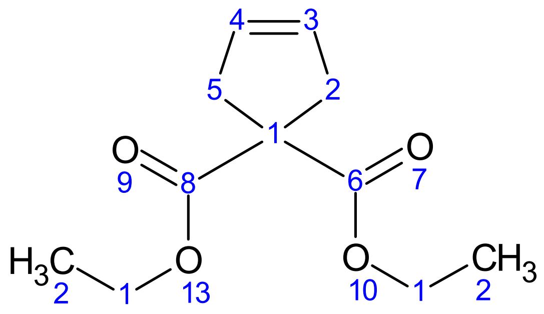

18

FZ7063-1.jdf

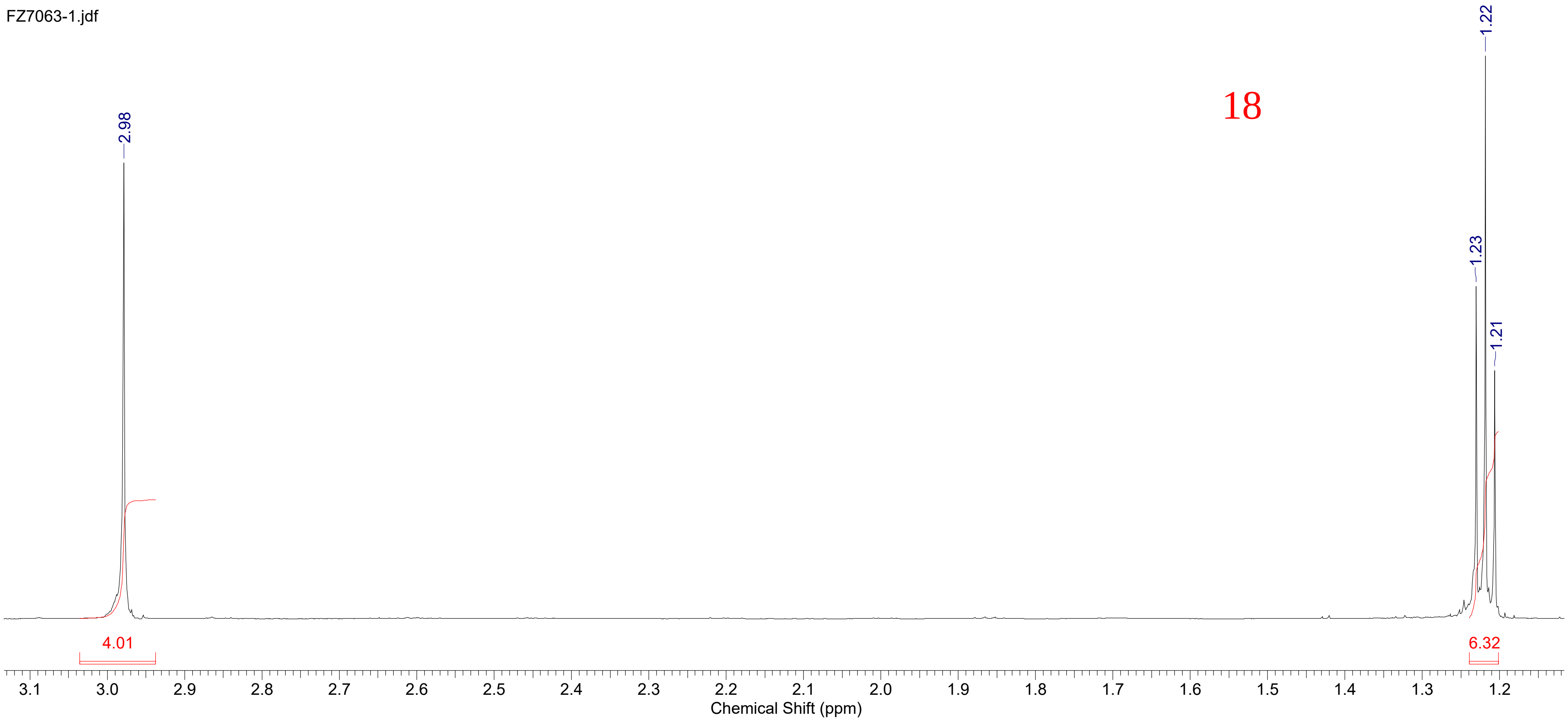

| | | | | | | | | | |
|---|---|---|---|---|---|---|---|---|---|
| ***Acquisition Time (sec)*** | 0.6921 | ***Comment*** | single pulse decoupled gated NOE | | | ***Date*** | 10 Jan 1990 11:59:28 | | |
| ***Date Stamp*** | 13 Dec 2018 07:43:04 | | | ***File Name*** | C:\Users\Fedor\Desktop\11.12.18\FZ7063-2.jdf | | | ***Frequency (MHz)*** | 150.91 |
| ***Nucleus*** | 13C | ***Number of Transients*** | 290 | ***Origin*** | ECA 600 | ***Original Points Count*** | 32768 | ***Owner*** | delta |
| ***Points Count*** | 32768 | ***Pulse Sequence*** | single_pulse_dec | | | ***Receiver Gain*** | 56.00 | ***Solvent*** | CHLOROFORM-d |
| ***Spectrum Offset (Hz)*** | 15091.3428 | ***Sweep Width (Hz)*** | 47348.49 | | | | | | |

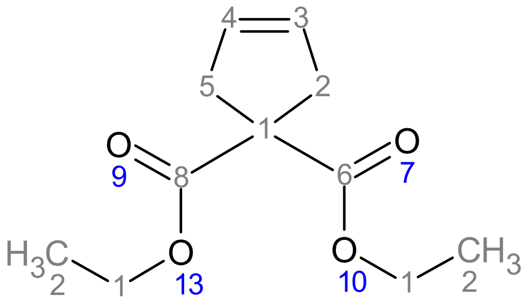

18

FZ7063-2.jdf

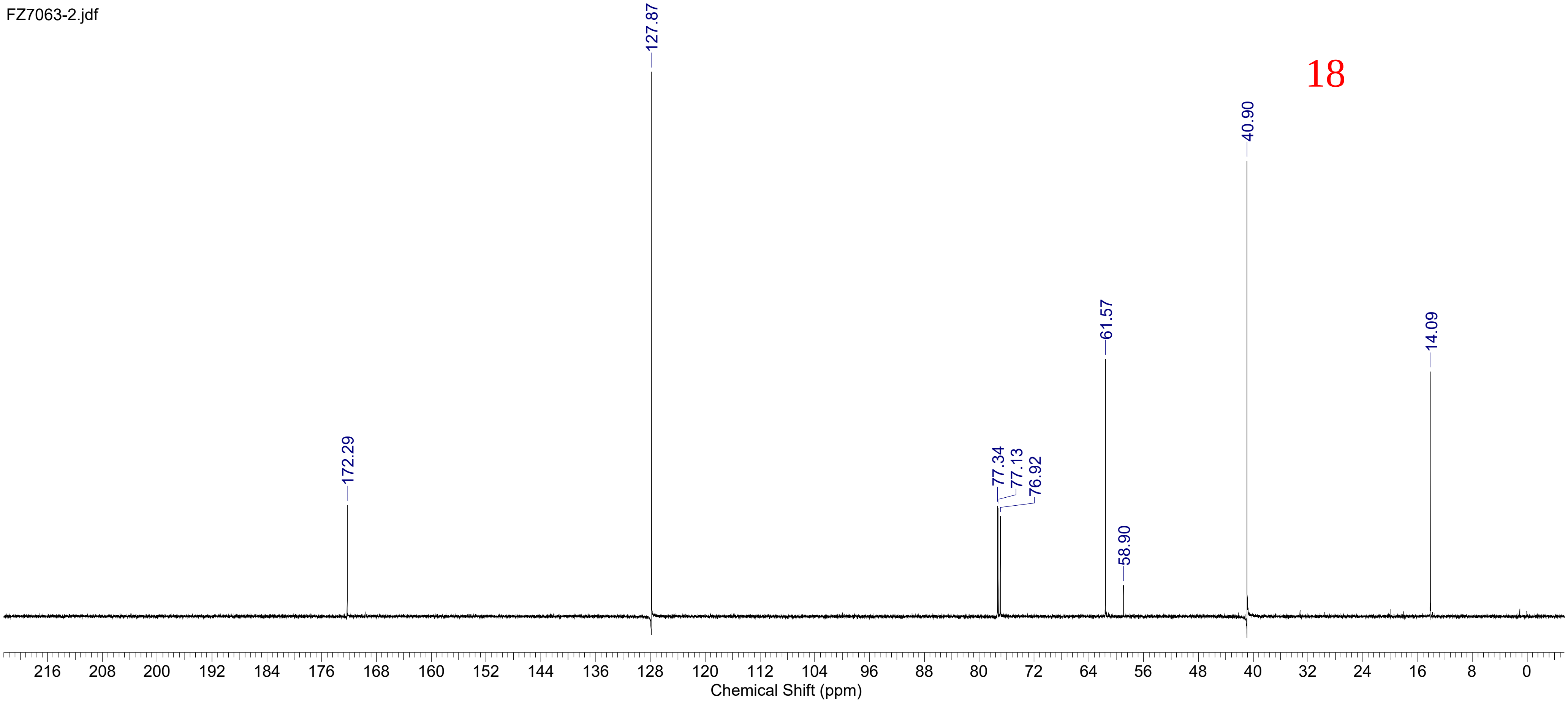

| | | | | | | | |
|---|---|---|---|---|---|---|---|
| ***Acquisition Time (sec)*** | 0.6921 | ***Comment*** | single pulse decoupled gated NOE | | | ***Date*** | 10 Jan 1990 11:59:28 |
| ***Date Stamp*** | 13 Dec 2018 07:43:04 | ***File Name*** | C:\Users\Fedor\Desktop\11.12.18\FZ7063-2.jdf | ***Frequency (MHz)*** | 150.91 | | |
| ***Nucleus*** | 13C | ***Number of Transients*** | 290 | ***Origin*** | ECA 600 | ***Original Points Count*** | 32768 |
| ***Owner*** | delta | ***Points Count*** | 32768 | ***Pulse Sequence*** | single_pulse_dec | ***Receiver Gain*** | 56.00 |
| ***Solvent*** | CHLOROFORM-d | ***Spectrum Offset (Hz)*** | 15091.3428 | ***Sweep Width (Hz)*** | 47348.49 | | |

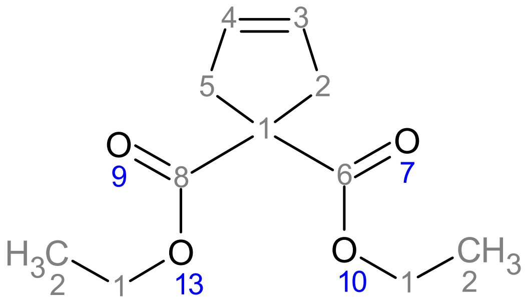

18

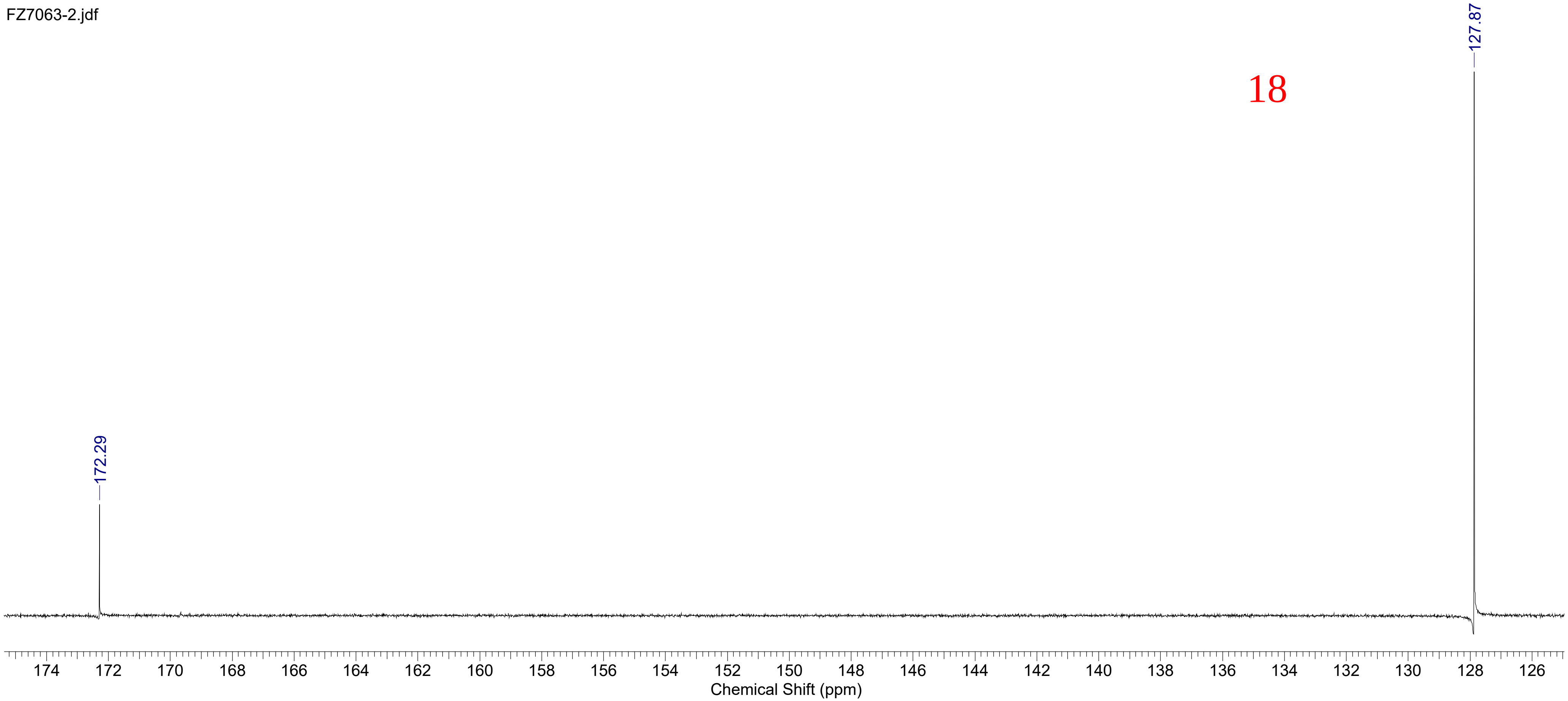

| | | | | | | | | | |
|---|---|---|---|---|---|---|---|---|---|
| ***Acquisition Time (sec)*** | 0.6921 | ***Comment*** | single pulse decoupled gated NOE | | | ***Date*** | 10 Jan 1990 11:59:28 | | |
| ***Date Stamp*** | 13 Dec 2018 07:43:04 | | | ***File Name*** | C:\Users\Fedor\Desktop\11.12.18\FZ7063-2.jdf | | | ***Frequency (MHz)*** | 150.91 |
| ***Nucleus*** | 13C | ***Number of Transients*** | 290 | ***Origin*** | ECA 600 | ***Original Points Count*** | 32768 | ***Owner*** | delta |
| ***Points Count*** | 32768 | ***Pulse Sequence*** | single_pulse_dec | | | ***Receiver Gain*** | 56.00 | ***Solvent*** | CHLOROFORM-d |
| ***Spectrum Offset (Hz)*** | 15091.3428 | ***Sweep Width (Hz)*** | 47348.49 | | | | | | |

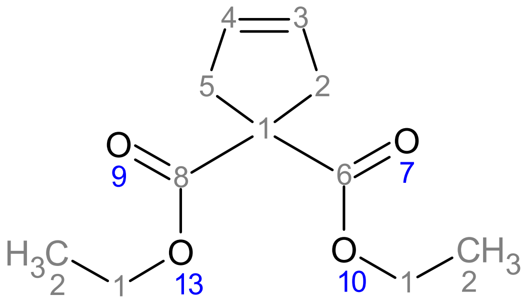


18

FZ7063-2.jdf

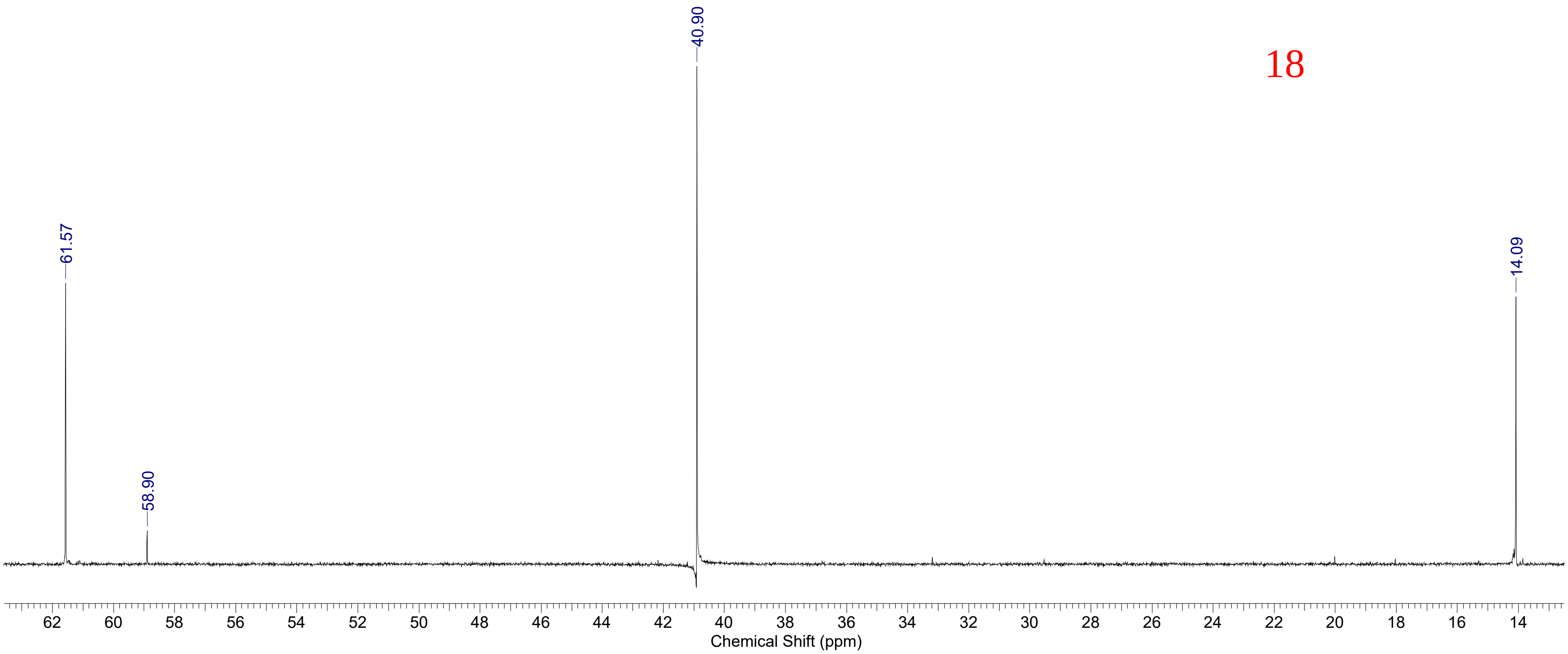

| | | | | | | | | | |
|---|---|---|---|---|---|---|---|---|---|
| ***Acquisition Time (sec)*** | 1.9818 | ***Comment*** | single_pulse | ***Date*** | 10 Jan 1990 12:13:38 | | | ***Date Stamp*** | 13 Dec 2018 07:57:14 |
| ***File Name*** | C:\Users\Fedor\Desktop\11.12.18\FZ7060-2.jdf | | | ***Frequency (MHz)*** | 600.17 | ***Nucleus*** | 1H | ***Number of Transients*** | 8 |
| ***Origin*** | ECA 600 | ***Original Points Count*** | 32768 | ***Owner*** | delta | ***Points Count*** | 32768 | ***Pulse Sequence*** | single_pulse.ex2 |
| ***Receiver Gain*** | 40.00 | ***Solvent*** | CHLOROFORM-d | | | ***Spectrum Offset (Hz)*** | 5412.1411 | ***Sweep Width (Hz)*** | 16534.39 |

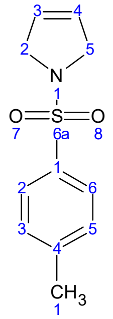

20

FZ7060-2.jdf

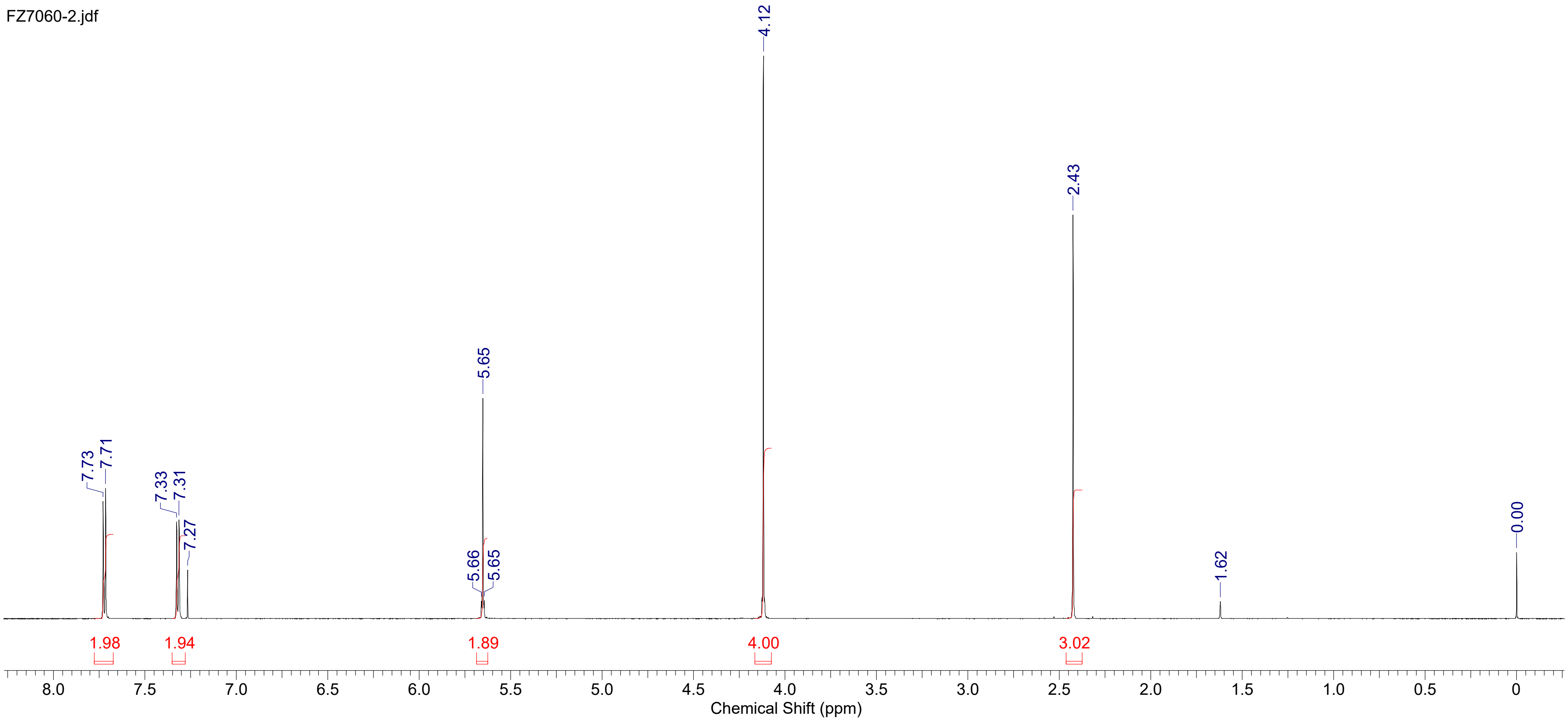

| | | | | | | | |
|---|---|---|---|---|---|---|---|
| ***Acquisition Time (sec)*** | 1.9818 | ***Comment*** | single_pulse | ***Date*** | 10 Jan 1990 12:13:38 | ***Date Stamp*** | 13 Dec 2018 07:57:14 |
| ***File Name*** | C:\Users\Fedor\Desktop\11.12.18\FZ7060-2.jdf | ***Frequency (MHz)*** | 600.17 | ***Nucleus*** | 1H | ***Number of Transients*** | 8 |
| ***Origin*** | ECA 600 | ***Original Points Count*** | 32768 | ***Owner*** | delta | ***Points Count*** | 32768 |
| ***Pulse Sequence*** | single_pulse.ex2 | ***Receiver Gain*** | 40.00 | ***Solvent*** | CHLOROFORM-d | ***Spectrum Offset (Hz)*** | 5412.1411 |
| ***Sweep Width (Hz)*** | 16534.39 | | | | | | |

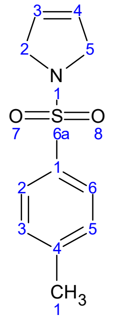


20

FZ7060-2.esp

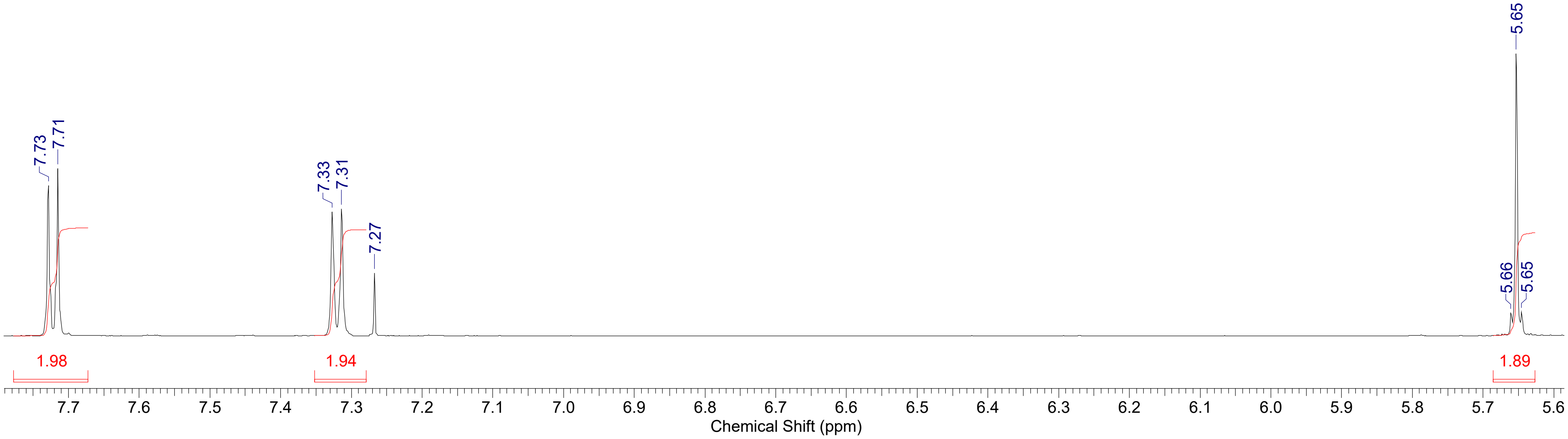

| | | | | | | | | | |
|---|---|---|---|---|---|---|---|---|---|
| ***Acquisition Time (sec)*** | 1.9818 | ***Comment*** | single_pulse | ***Date*** | 10 Jan 1990 12:13:38 | | | ***Date Stamp*** | 13 Dec 2018 07:57:14 |
| ***File Name*** | C:\Users\Fedor\Desktop\11.12.18\FZ7060-2.jdf | | | ***Frequency (MHz)*** | 600.17 | ***Nucleus*** | 1H | ***Number of Transients*** | 8 |
| ***Origin*** | ECA 600 | ***Original Points Count*** | 32768 | ***Owner*** | delta | ***Points Count*** | 32768 | ***Pulse Sequence*** | single_pulse.ex2 |
| ***Receiver Gain*** | 40.00 | ***Solvent*** | CHLOROFORM-d | | | ***Spectrum Offset (Hz)*** | 5412.1411 | ***Sweep Width (Hz)*** | 16534.39 |

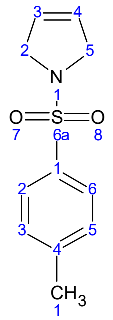

20

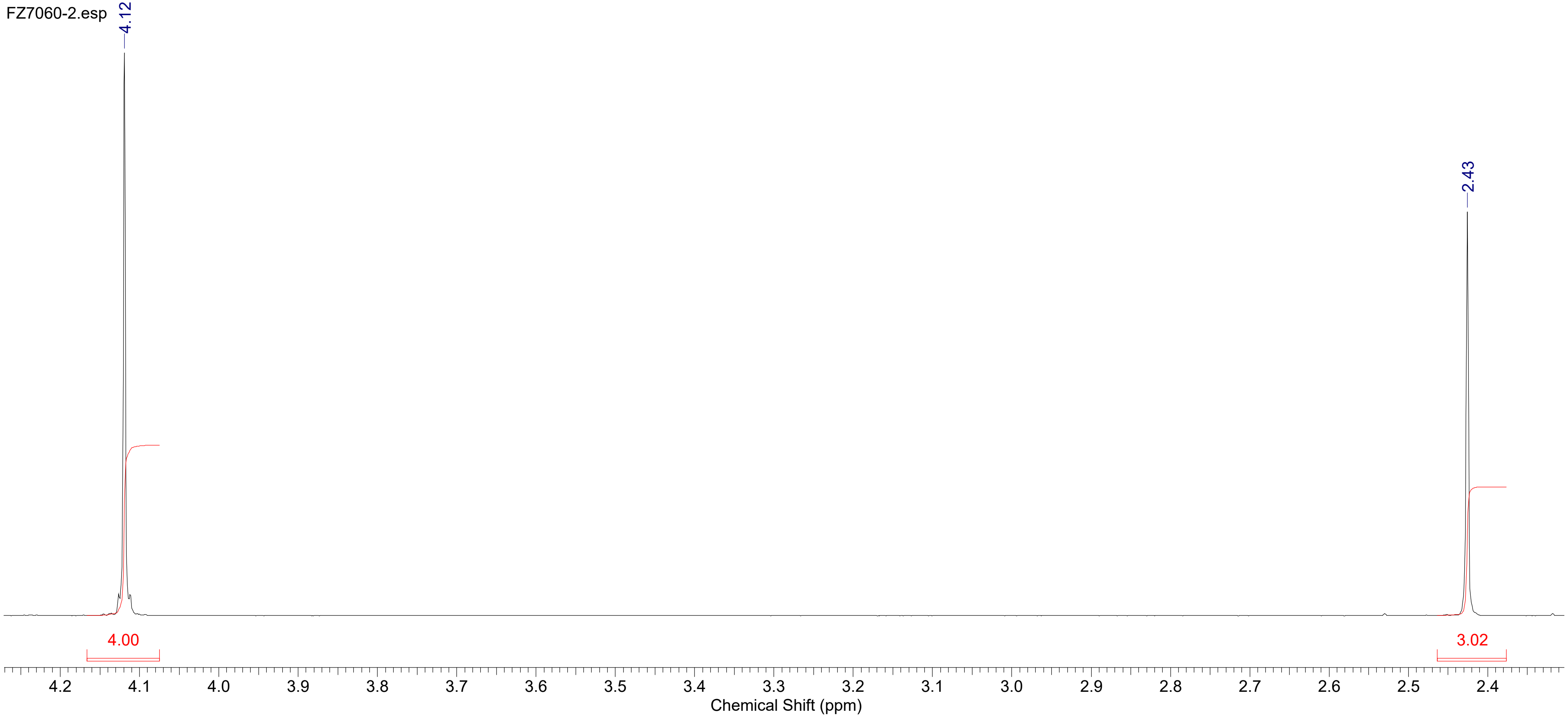

| | | | | | | | | | |
|---|---|---|---|---|---|---|---|---|---|
| ***Acquisition Time (sec)*** | 0.6921 | ***Comment*** | single pulse decoupled gated NOE | ***Date*** | 10 Jan 1990 12:12:28 | | | | |
| ***Date Stamp*** | 13 Dec 2018 07:56:04 | ***File Name*** | C:\Users\Fedor\Desktop\11.12.18\FZ7060-1.jdf | ***Frequency (MHz)*** | 150.91 | | | | |
| ***Nucleus*** | 13C | ***Number of Transients*** | 400 | ***Origin*** | ECA 600 | ***Original Points Count*** | 32768 | ***Owner*** | delta |
| ***Points Count*** | 32768 | ***Pulse Sequence*** | single_pulse_dec | ***Receiver Gain*** | 56.00 | ***Solvent*** | CHLOROFORM-d | | |
| ***Spectrum Offset (Hz)*** | 15079.3525 | ***Sweep Width (Hz)*** | 47348.49 | | | | | | |

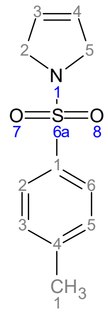


20

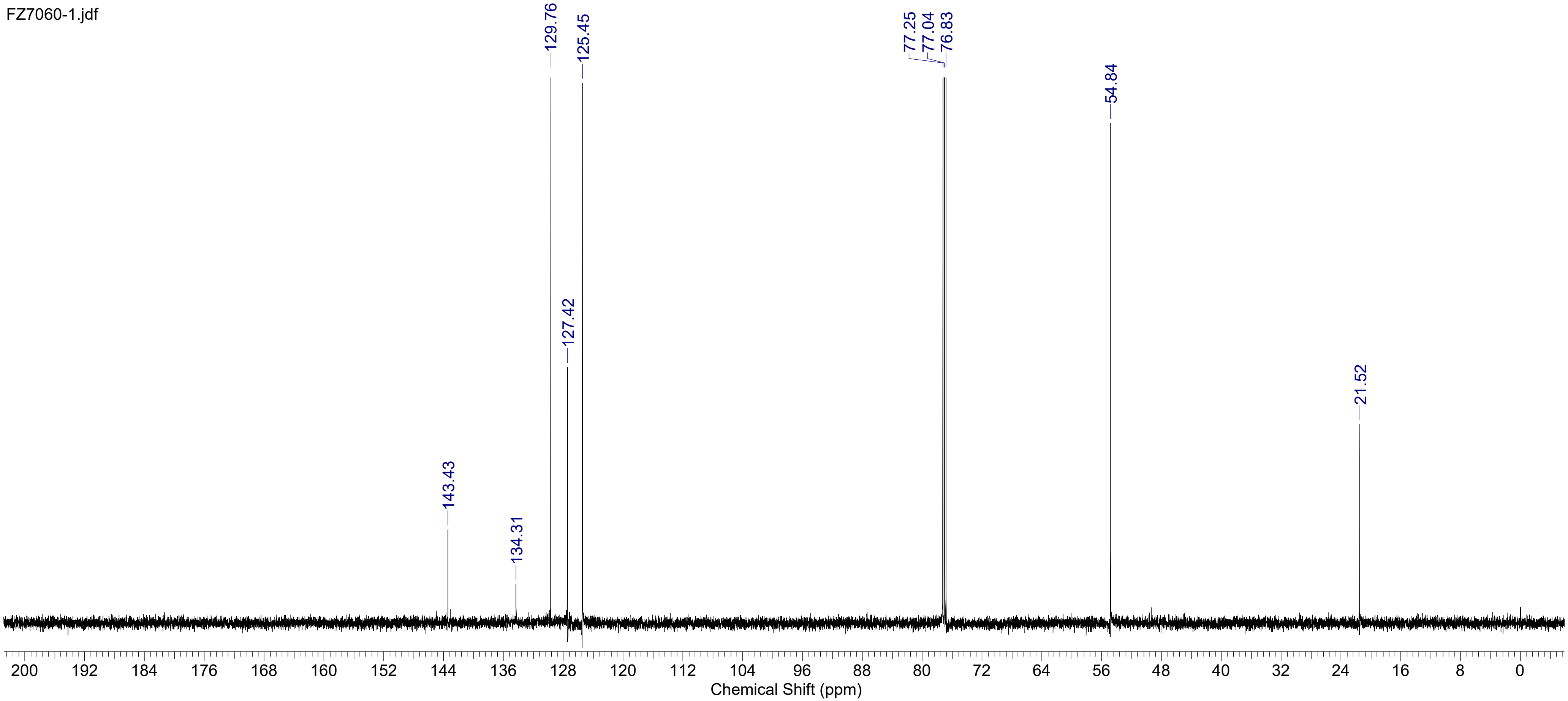

| | | | | | | | | | |
|---|---|---|---|---|---|---|---|---|---|
| ***Acquisition Time (sec)*** | 0.6921 | ***Comment*** | single pulse decoupled gated NOE | | | ***Date*** | 10 Jan 1990 12:12:28 | | |
| ***Date Stamp*** | 13 Dec 2018 07:56:04 | | | ***File Name*** | C:\Users\Fedor\Desktop\11.12.18\FZ7060-1.jdf | | | ***Frequency (MHz)*** | 150.91 |
| ***Nucleus*** | 13C | ***Number of Transients*** | 400 | ***Origin*** | ECA 600 | ***Original Points Count*** | 32768 | ***Owner*** | delta |
| ***Points Count*** | 32768 | ***Pulse Sequence*** | single_pulse_dec | | | ***Receiver Gain*** | 56.00 | ***Solvent*** | CHLOROFORM-d |
| ***Spectrum Offset (Hz)*** | 15079.3525 | ***Sweep Width (Hz)*** | 47348.49 | | | | | | |

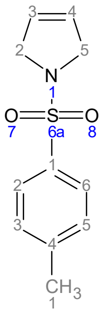

20

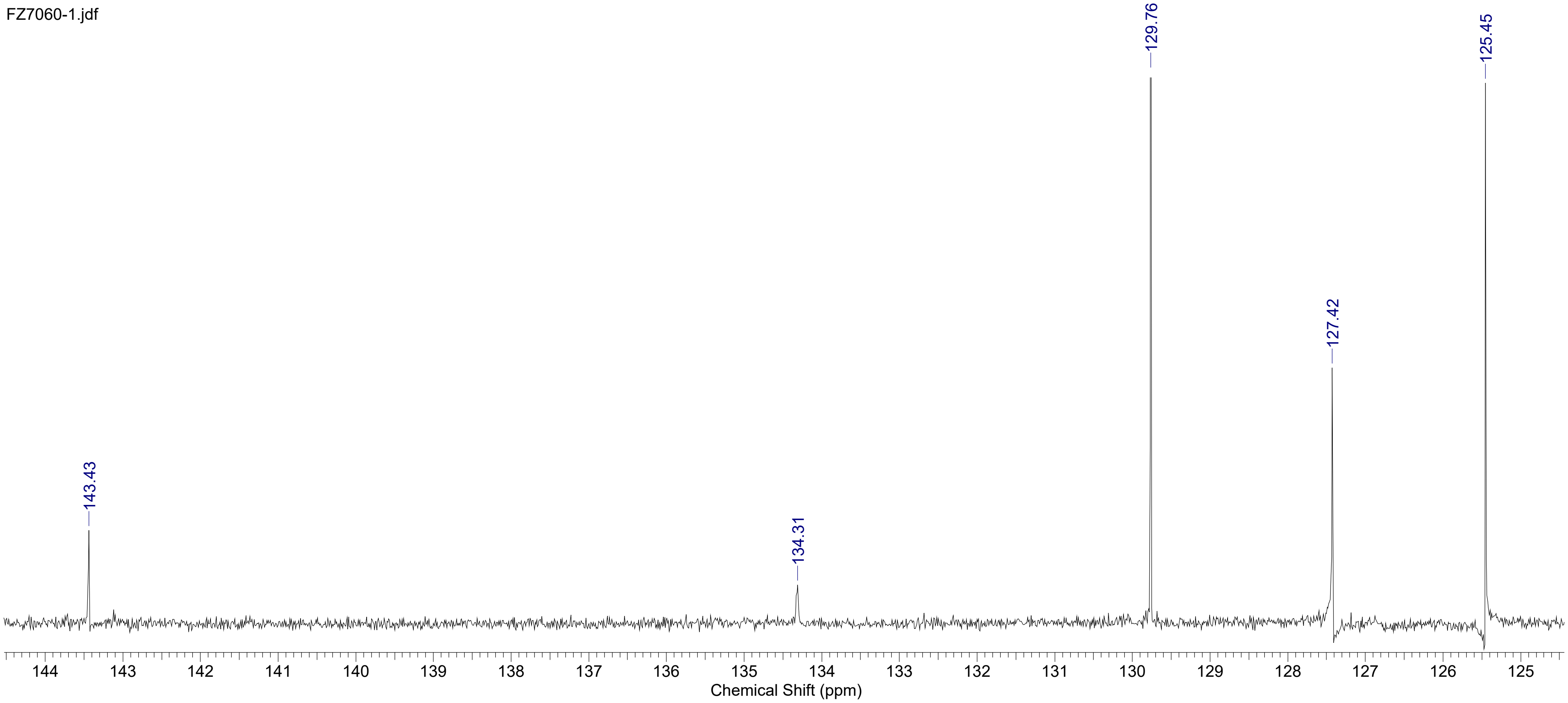

## 2. Selected GC-MS data for metathesis products (**13-27**)

**Table 3:** Reaction conditions and yields of the metathesis products

| Entry | Starting compound | Catalyst | Catalyst concentration (mol %) | Solvent[a] (conditions) | Yield (%), ratio[b] of the products |
|---|---|---|---|---|---|
| 1 | **12** | **11a** | 1 | PhMe (Ar) | **13** (traces)[c] |
| 2 | **12** | **11b** | 1 | PhMe (Ar) | **12**/**13** (43%), 51/49 |
| 3 | **12** | **11a** | 1 | PhH (Ar) | **13** (traces)[c] |
| 4 | **12** | **11b** | 1 | PhH (Ar) | **12**/**13** (52%), 40/60 |
| 5 | **12** | **11a** | 1 | MeCN (Ar) | no product[c] |
| 6 | **12** | **11a** | 1 | THF (Ar) | no product[c] |
| 7 | **12** | **11a** | 1 | $CH_2Cl_2$ (Ar) | **13** (17%)[d] |
| 8 | **12** | **11a** | 0.1 | $CH_2Cl_2$ (Ar) | **13** (traces)[c] |
| 9 | **12** | **11b** | 1 | $CH_2Cl_2$ (Ar) | **13** (49%)[d] |
| 10 | **12** | **11b** | 0.1 | $CH_2Cl_2$ (Ar) | **13** (79%)[d] |
| 11 | **12** | **11c** | 1 | $CH_2Cl_2$ (Ar) | **12**/**13** (51%), 44/56 |
| 12 | **12** | **11d** | 1 | $CH_2Cl_2$ (Ar) | **13** (91%)[d] |
| 13 | **12** | **11d** | 0.1 | $CH_2Cl_2$ (Ar) | **13** (97%)[d] |
| 14 | **12** | **11d** | 0.01 | $CH_2Cl_2$ (Ar) | **12**/**13** (93%), 69/31 |
| 15 | **12** | **11a** | 1 | $CHCl_3$ (Ar) | **13** (86%)[d] |
| 16 | **12** | **11a** | 0.1 | $CHCl_3$ (Ar) | **13** (95%)[d] |
| 17 | **12** | **11b** | 1 | $CHCl_3$ (Ar) | **13** (96%)[d] |
| 18 | **12** | **11b** | 0.1 | $CHCl_3$ (Ar) | **13** (97%)[d] |
| 19 | **12** | **11b** | 0.01 | $CHCl_3$ (Ar) | **13** (81%)[d] |
| 20 | **12** | **11b** | 0.1 | $CHCl_3$ (air) | **13** (89%)[d] |
| 21 | **12** | **11d** | 1 | $CHCl_3$ (Ar) | **13** (95%)[d] |
| 22 | **12** | **11d** | 0.1 | $CHCl_3$ (Ar) | **13** (99%)[d] |
| 23 | **12** | **11d** | 0.01 | $CHCl_3$ (Ar) | **12**/**13** (98%), 64/36 |
| 24 | **14** | **11b** | 0.1 | $CHCl_3$ (Ar) | **15**/**16** (93%), 64/36 |
| 25 | **17** | **11a** | 0.1 | $CHCl_3$ (air) | **17**/**18** (46%), 44/56 |
| 26 | **17** | **11b** | 0.1 | $CHCl_3$ (air) | **17**/**18** (58%), 42/58 |
| 27 | **17** | **11b** | 0.01 | $CHCl_3$ (Ar) | **18** (traces)[e] |
| 28 | **17** | **11b** | 0.1 | $CHCl_3$ (Ar) | **17**/**18** (98%), 2/98 |
| 29 | **17** | **11d** | 0.1 | $CHCl_3$ (air) | **17**/**18** (63%), 42/58 |
| 30 | **17** | **11d** | 0.1 | $CHCl_3$ (Ar) | **17**/**18** (96%), 4/96 |
| 31 | **17** | **11d** | 0.01 | $CHCl_3$ (air) | **18** (traces) |
| 32 | **19** | **11d** | 0.1 | $CHCl_3$ (Ar) | **19**/**20** (99%), 5/95 |
| 33 | **19** | **11d** | 0.01 | $CHCl_3$ (Ar) | **19**/**20** (75%), 23/77 |
| 34 | **21** + 2 eq **12** | **11a** | 0.1 | $CHCl_3$ (Ar) | **13**/**22**/**23** (71%), ~77/20/3[f] |
| 35 | **21** + 2 eq **12** | **11d** | 0.1 | $CHCl_3$ (Ar) | **13**/**22**/**23** (78%), ~81/18/1[f] |
| 36 | **21** + 2 eq **24** | **11a** | 0.1 | $CHCl_3$ (Ar) | **25**/**26**/**27** (40%), ~3/76/21[f] |
| 37 | **21** + 2 eq **24** | **11b** | 0.1 | $CHCl_3$ (Ar) | **25**/**26**/**27** (52%), ~2/72/26[f] |

**Entry 2.**

Starting compound – **12**. Catalyst – **11a** (1 mol %). The reaction mixture was heated at reflux in PhH for 4 h under an argon atmosphere.

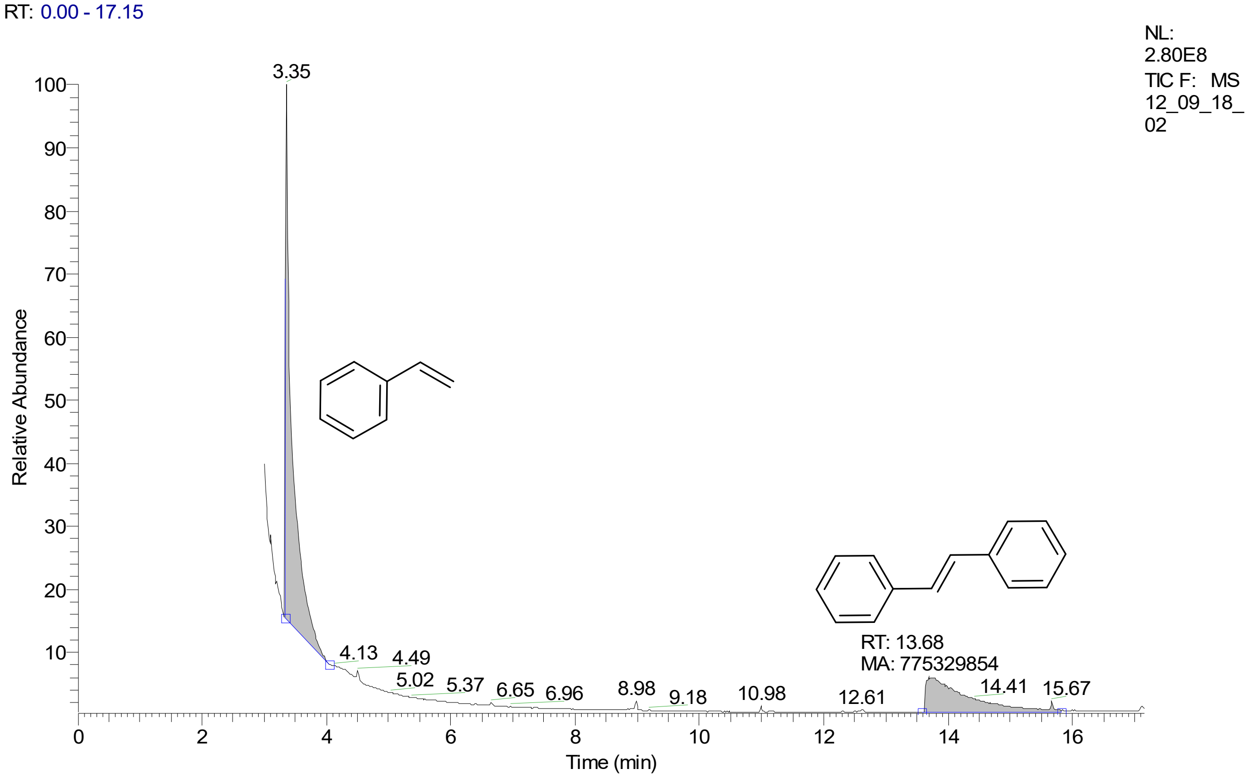


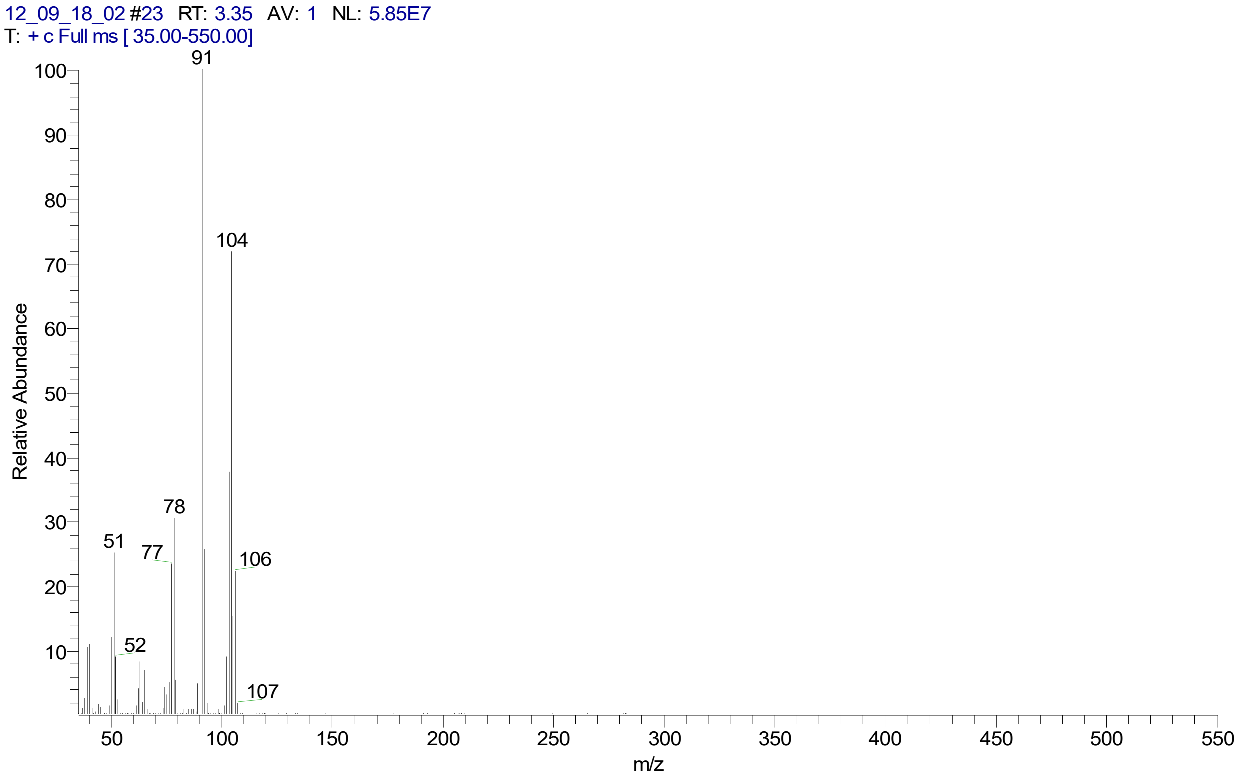

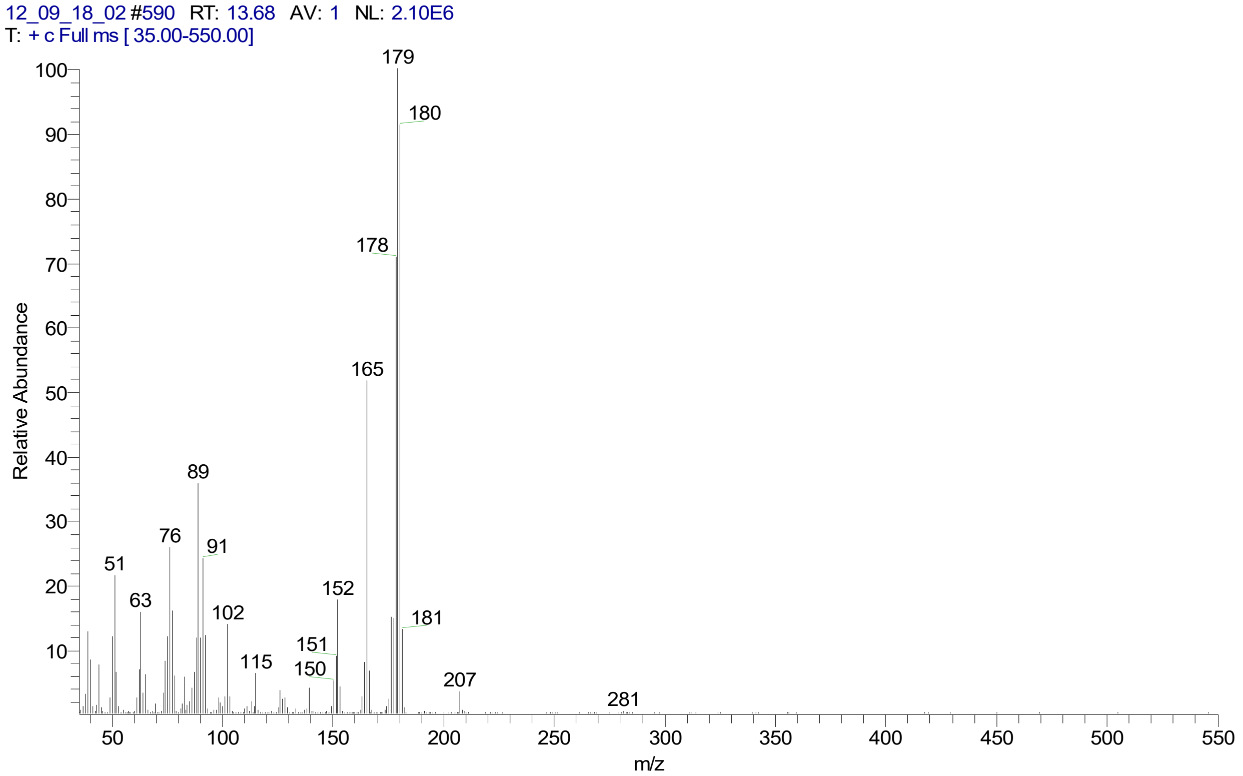


**Entry 3.**
Starting compound – **12**. Catalyst – **11a** (1 mol %). The reaction mixture was heated at reflux in PhH for 4 h under an argon atmosphere.

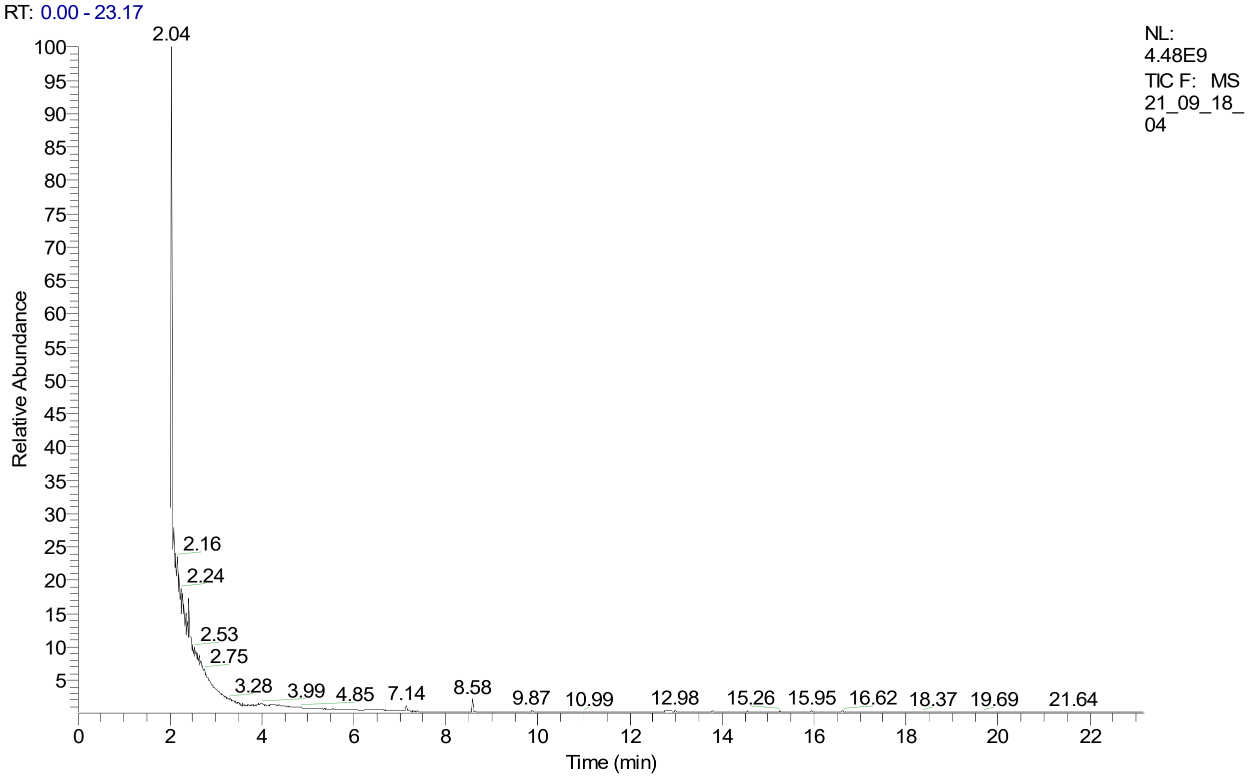

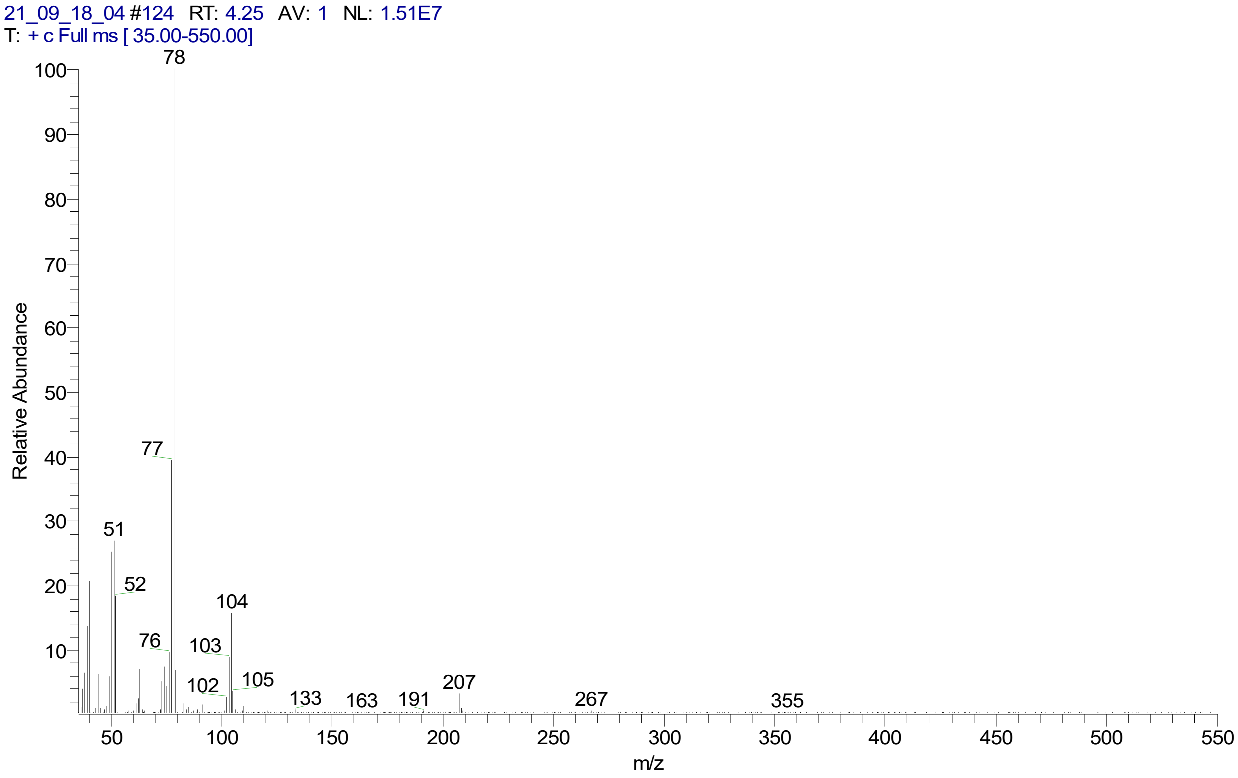


**Entry 4.**
Starting compound – **12**. Catalyst – **11d** (1 mol %). The reaction mixture was heated at reflux in PhMe for 4 h.

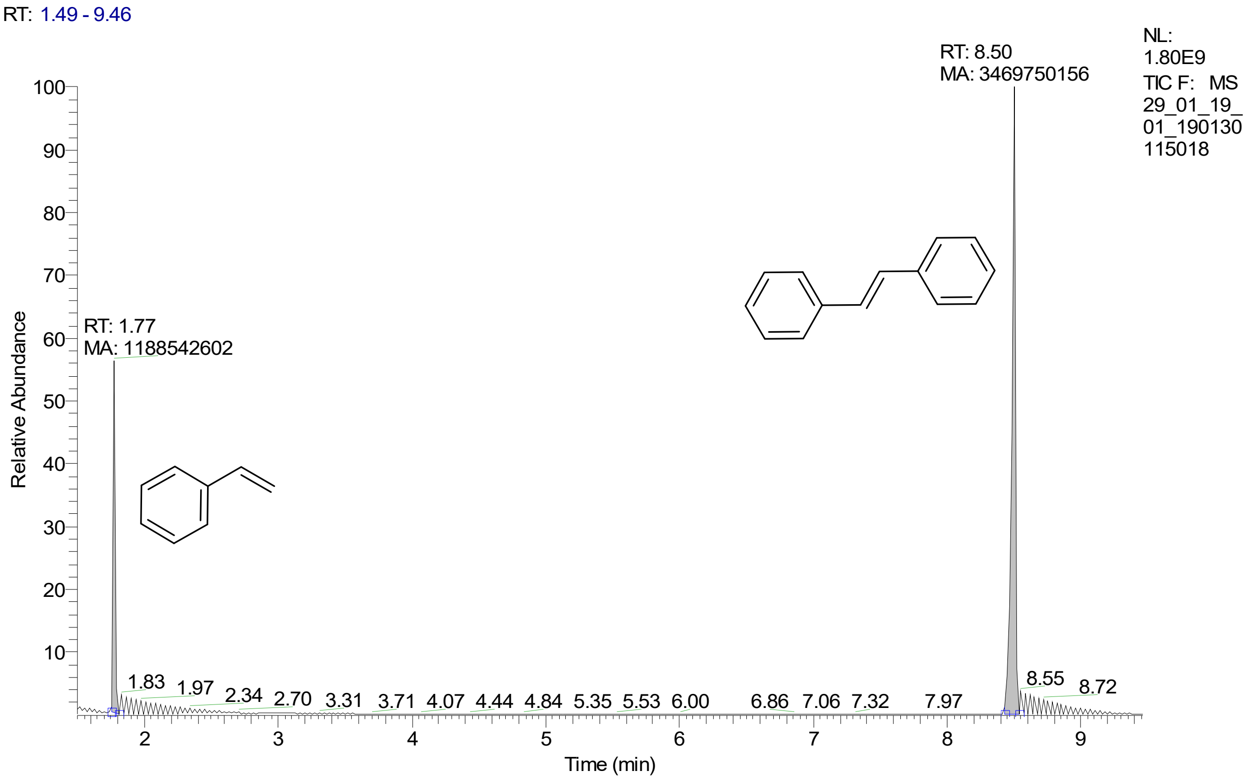

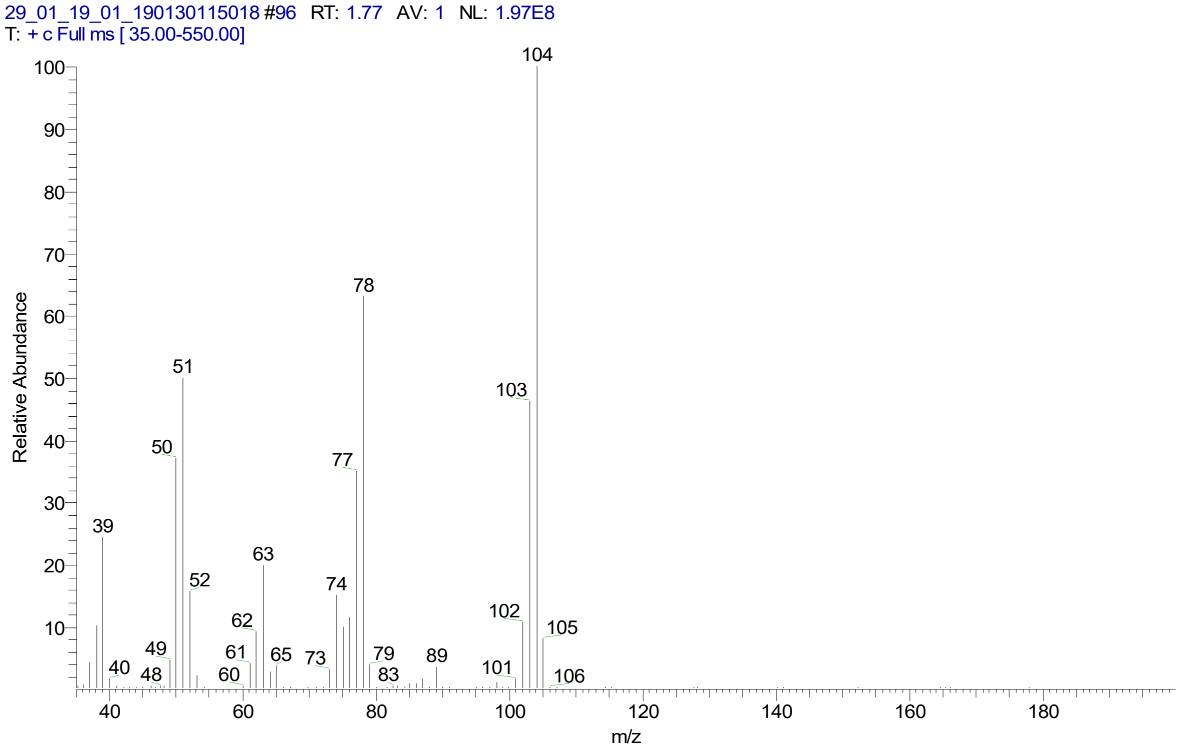
29_01_19_01_190130115018 #96 RT: 1.77 AV: 1 NL: 1.97E8
T: + c Full ms [ 35.00-550.00]
Relative Abundance
m/z
104
78
51
103
50
77
39
63
52
74
102
105
62
49
61
65
73
79
89
40
48
60
83
101
106

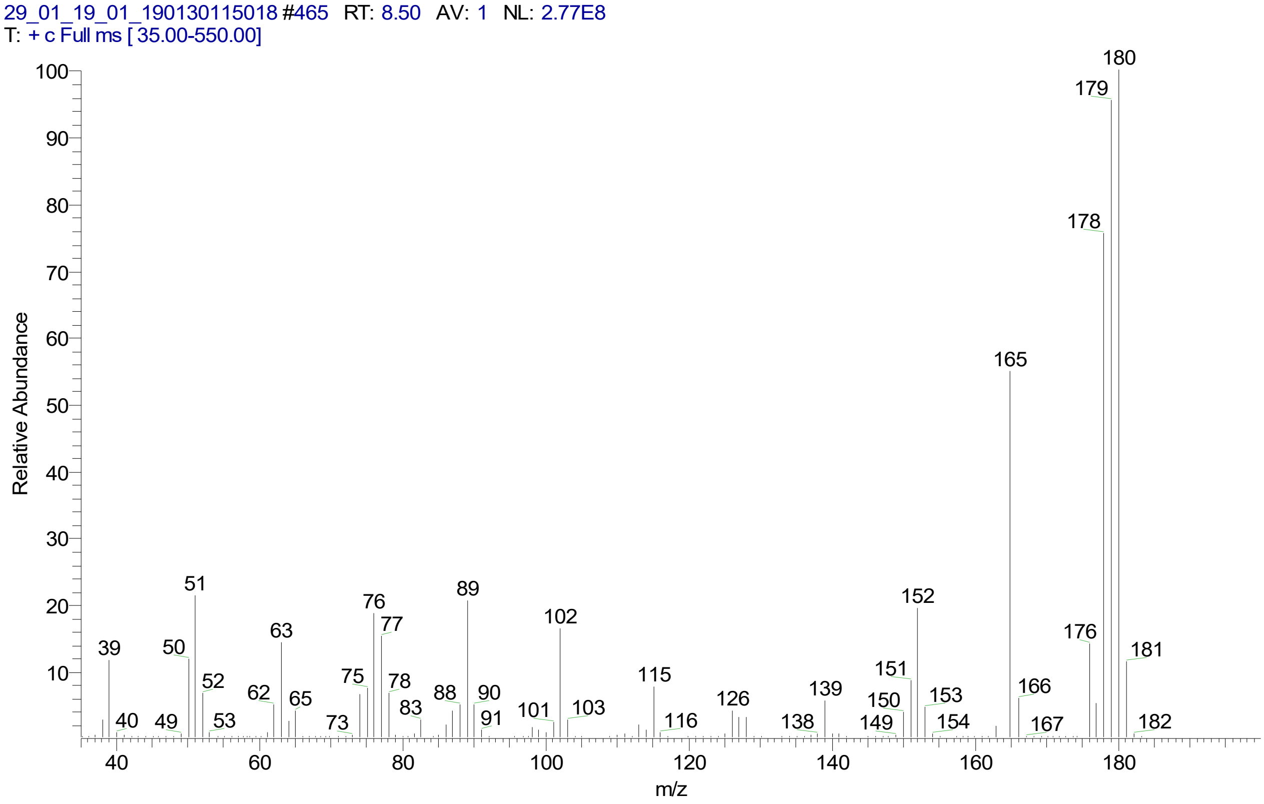
29_01_19_01_190130115018 #465 RT: 8.50 AV: 1 NL: 2.77E8
T: + c Full ms [ 35.00-550.00]
Relative Abundance
m/z
180
179
178
165
51
89
76
152
77
102
39
50
63
176
181
115
151
52
62
75
78
88
90
139
126
166
153
65
83
101
103
150
40
49
53
73
91
116
138
149
154
167
182

**Entry 5.**

Starting compound – **12**. Catalyst – **11a** (1 mol %). The reaction mixture was heated at reflux in MeCN for 4 h under an argon atmosphere.

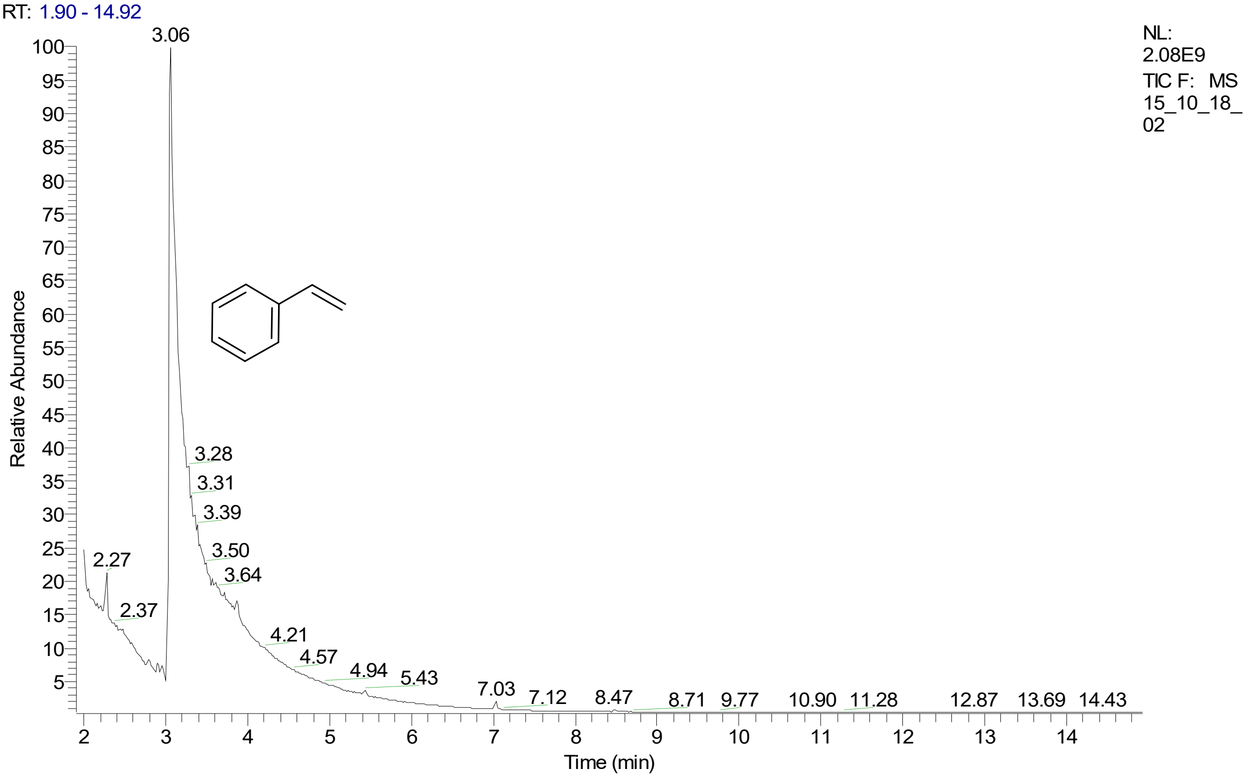


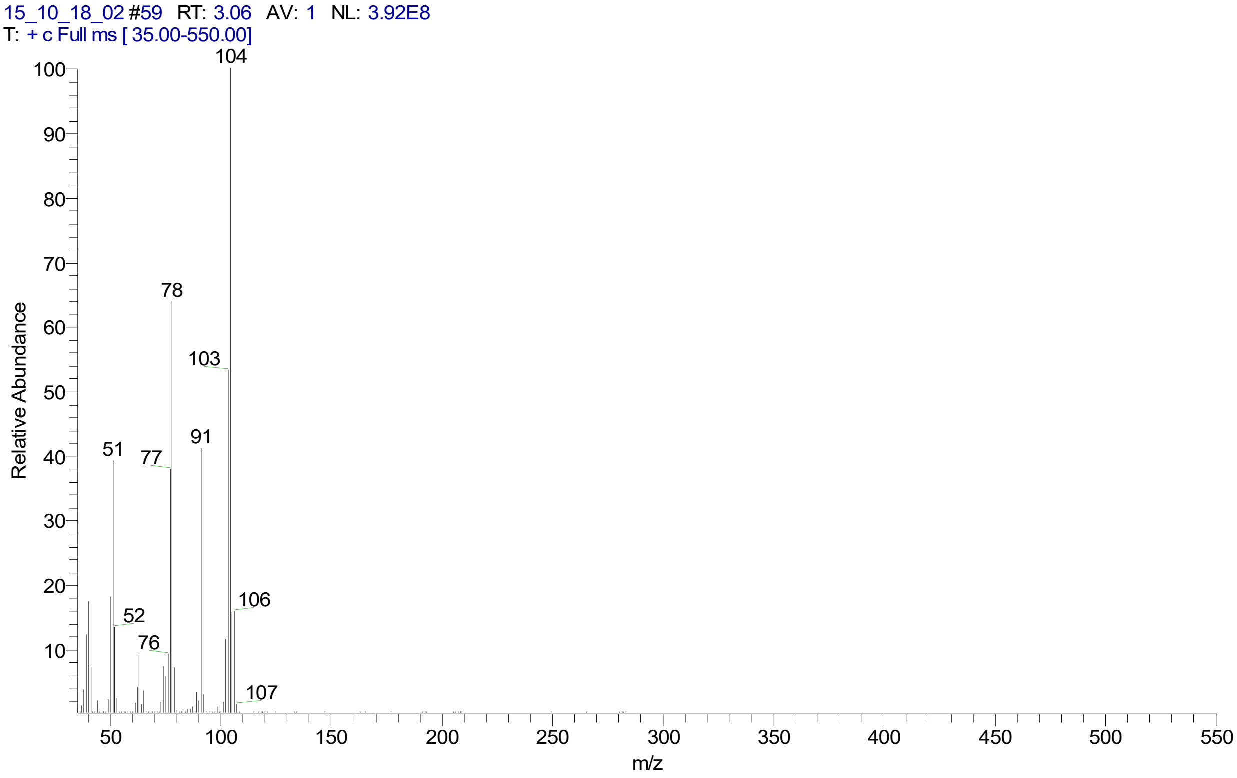

**Entry 9.**

Starting compound – **12**. Catalyst – **11b** (1 mol %). The reaction mixture was heated at reflux in $CH_2Cl_2$ for 4 h under an argon atmosphere.

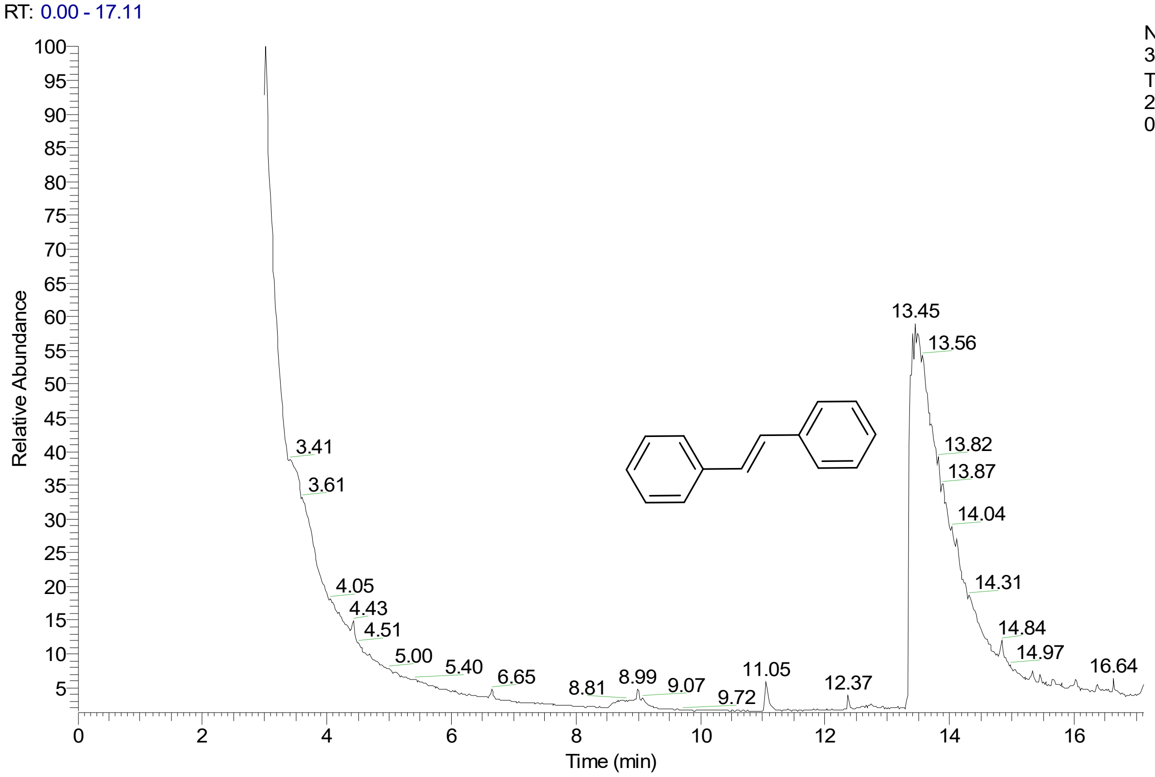


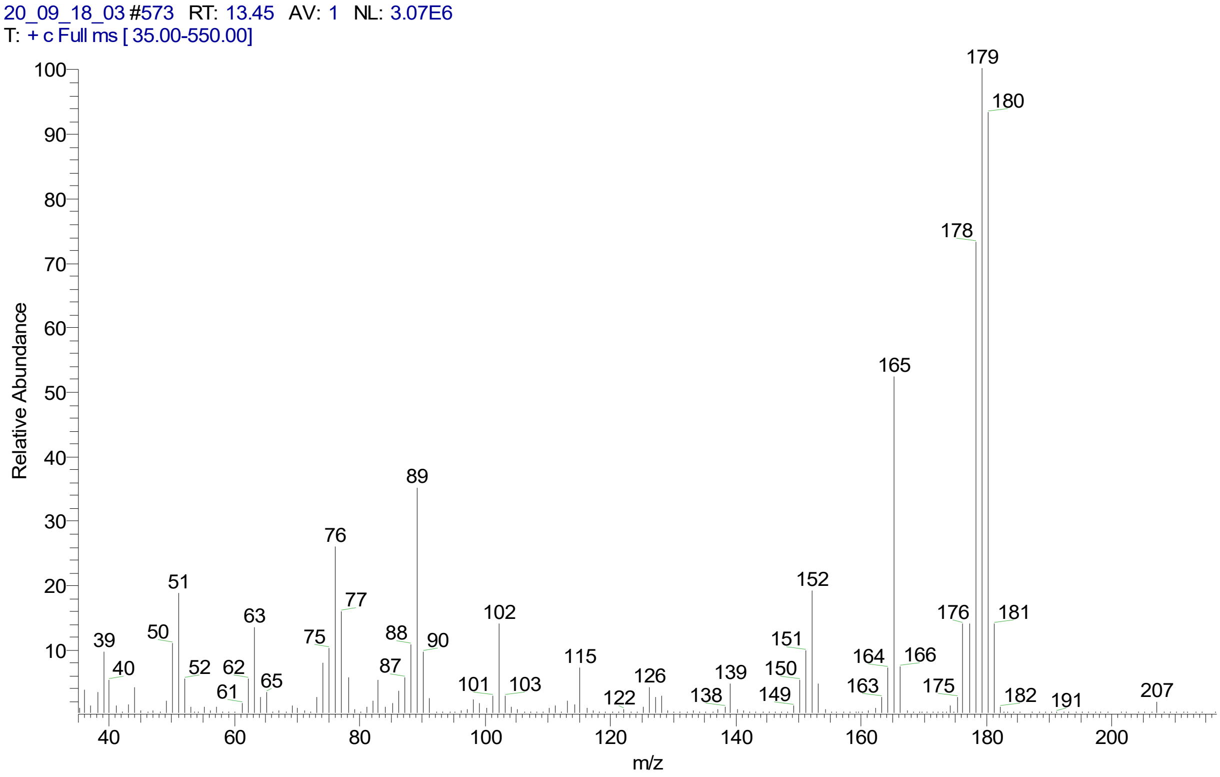

**Entry 11.**

Starting compound – **12**. Catalyst – **11c** (1 mol %). The reaction mixture was heated at reflux in $CH_2Cl_2$ for 4 h under an argon atmosphere.

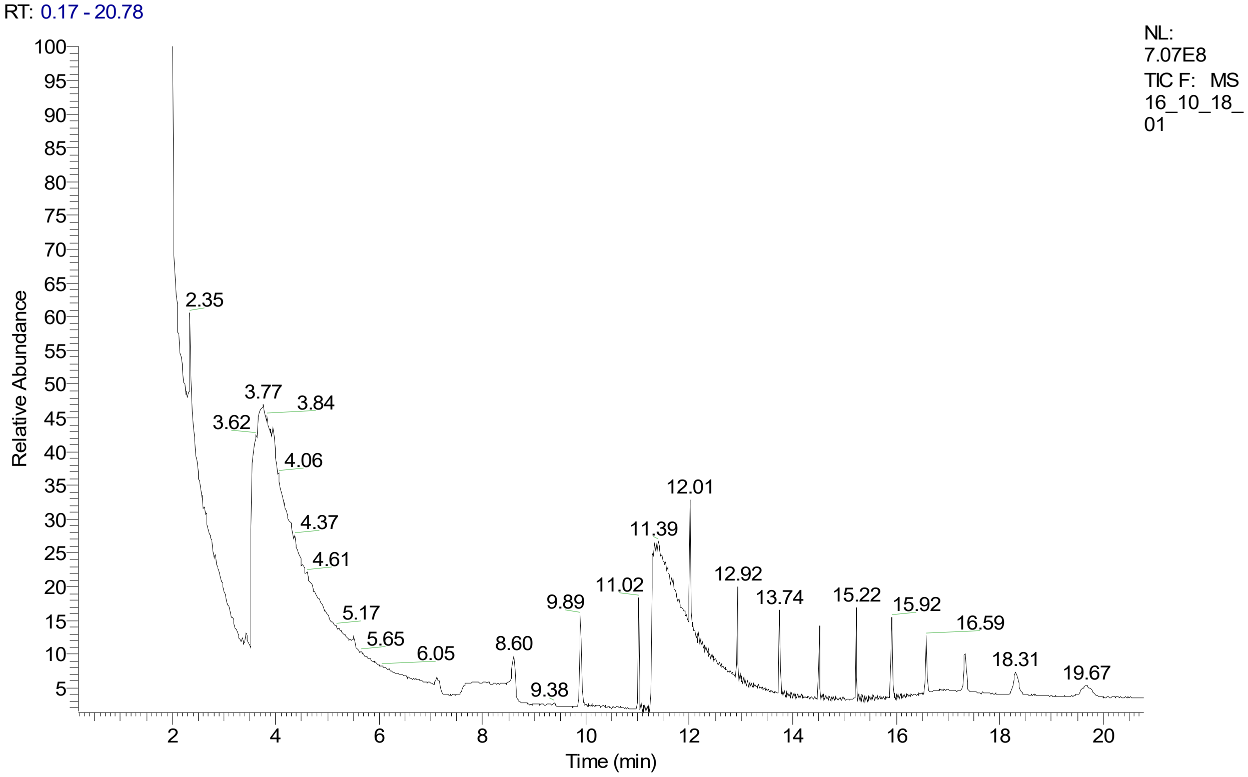


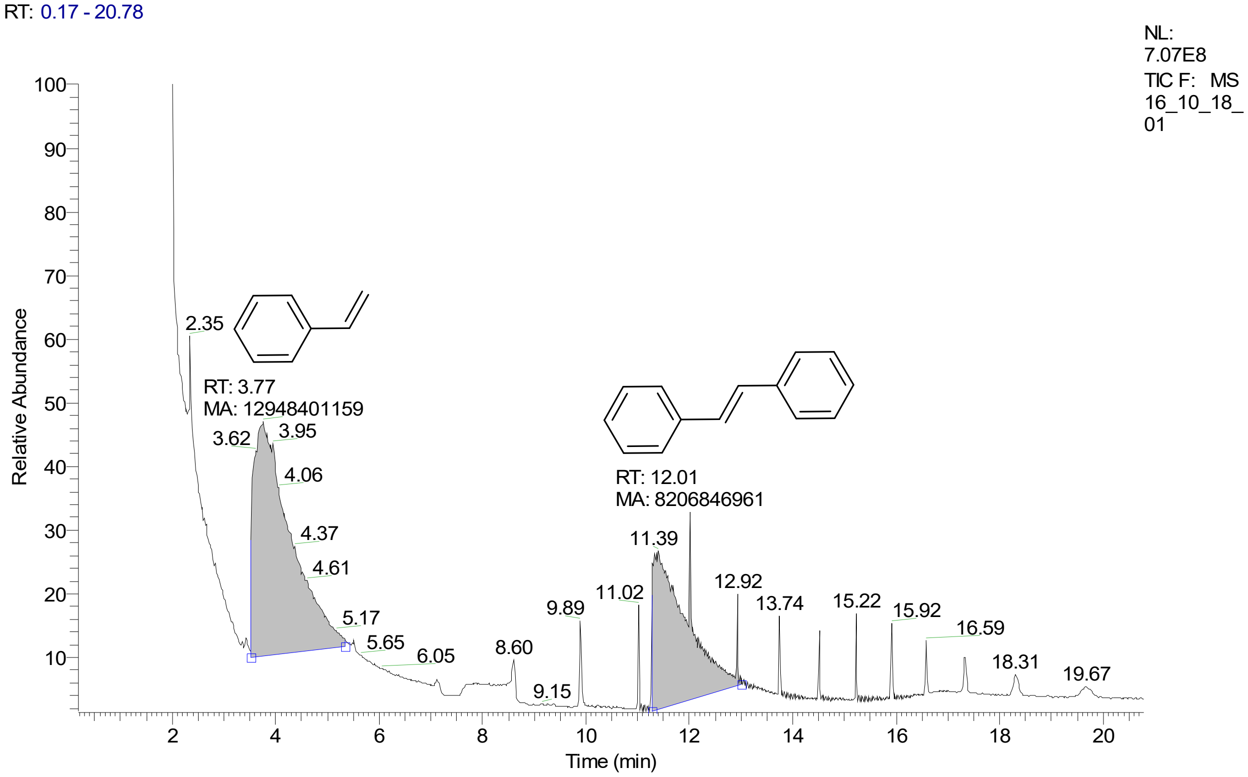

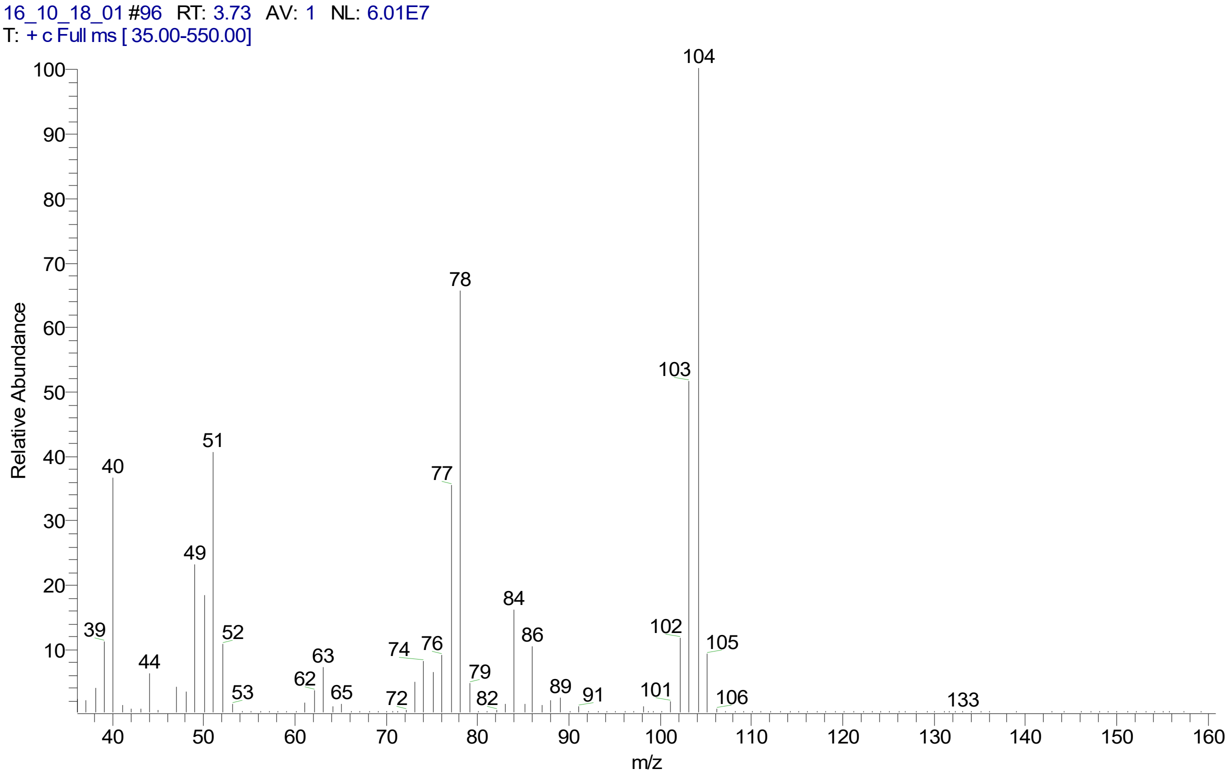
16_10_18_01 #96 RT: 3.73 AV: 1 NL: 6.01E7
T: + c Full ms [ 35.00-550.00]
Relative Abundance
m/z
104
103
78
77
51
40
49
84
86
102
105
39
52
44
63
74
76
79
62
53
65
72
82
89
91
101
106
133

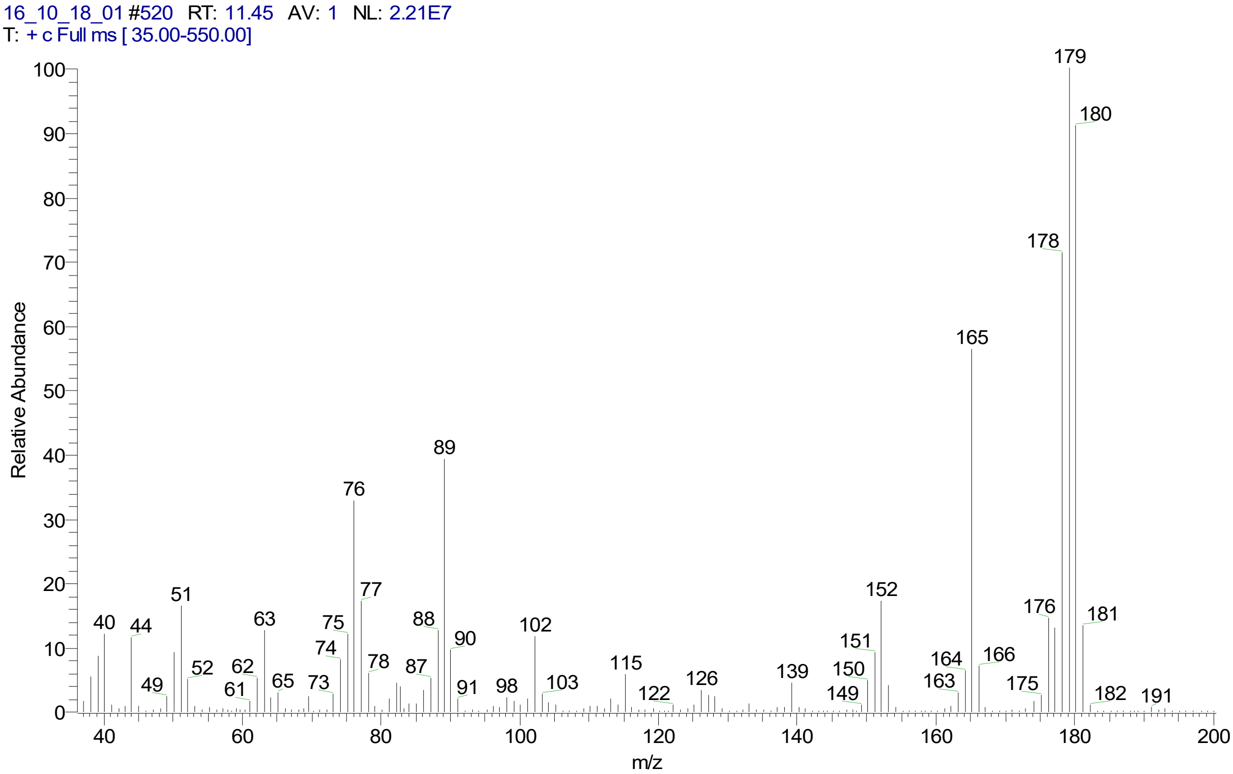
16_10_18_01 #520 RT: 11.45 AV: 1 NL: 2.21E7
T: + c Full ms [ 35.00-550.00]
Relative Abundance
m/z
179
180
178
165
89
76
152
176
181
51
77
40
44
63
75
88
102
90
74
151
164
166
62
52
78
87
115
150
126
139
49
61
65
73
91
98
103
122
149
163
175
182
191

**Entry 12.**

Starting compound – **12**. Catalyst – **11d** (1 mol %). The reaction mixture was heated at reflux in $CH_2Cl_2$ for 4 h under an argon atmosphere.

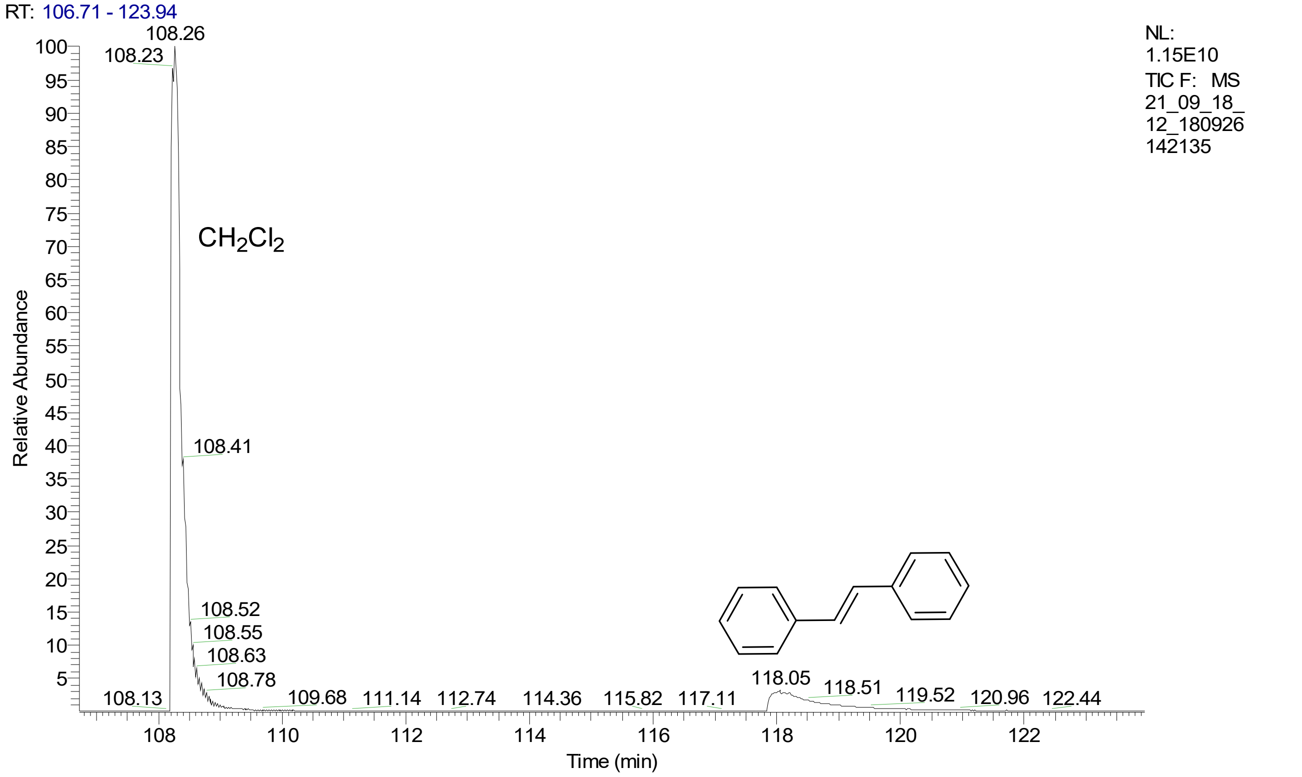


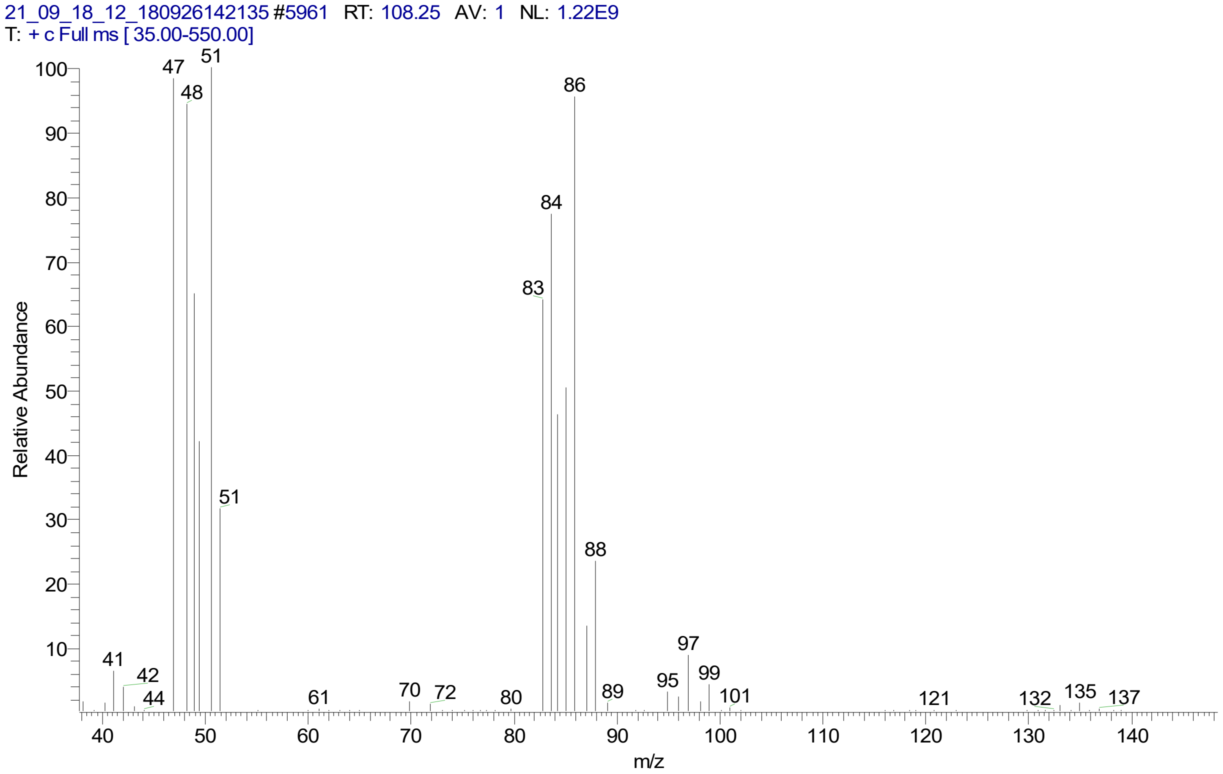

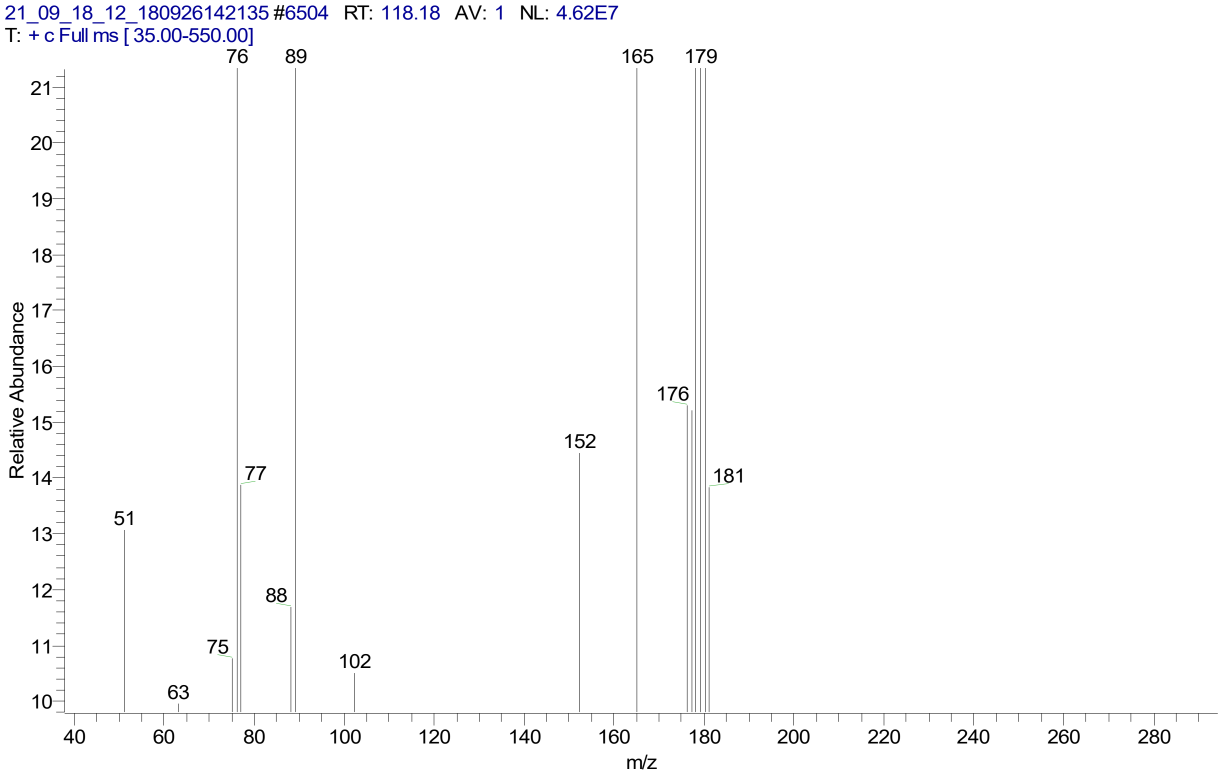


**Entry 13.**

Starting compound – **12**. Catalyst – **11d** (0.1 mol %). The reaction mixture was heated at reflux in $CH_2Cl_2$ for 4 h under an argon atmosphere.

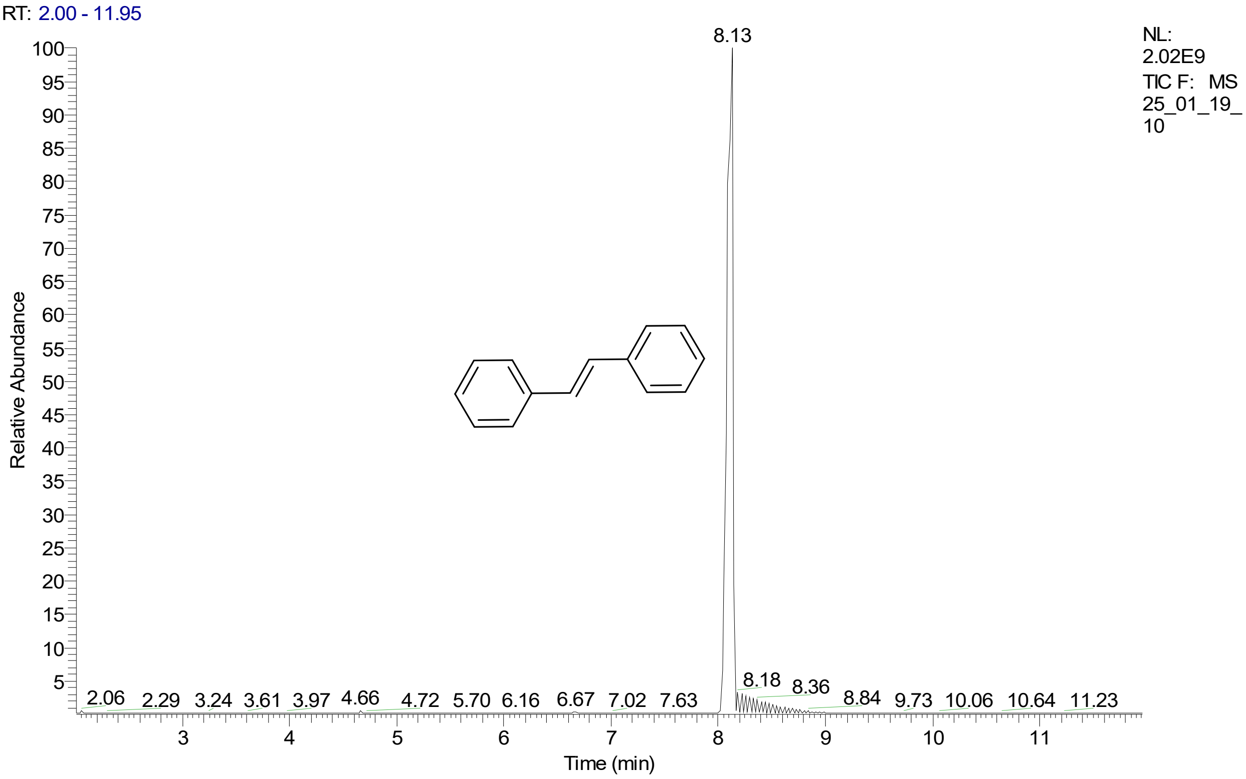

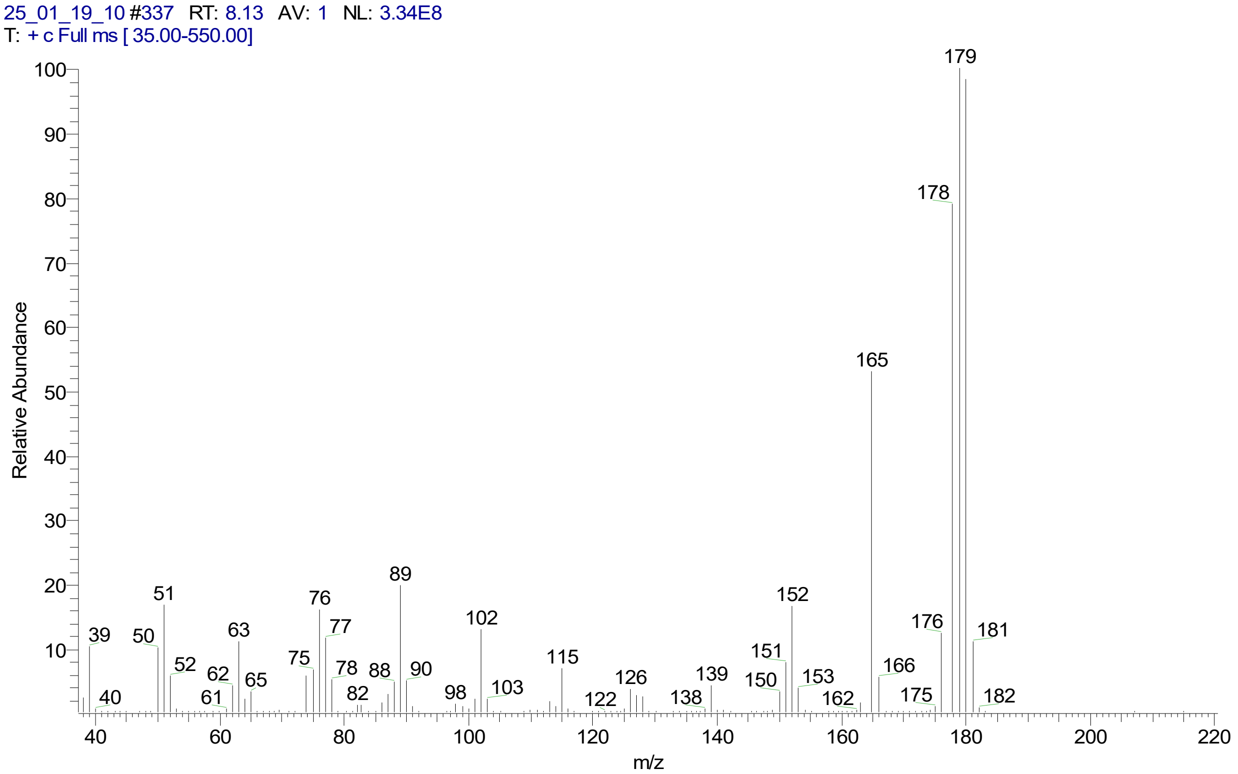


**Entry 17.**

Starting compound – **12**. Catalyst – **11b** (1 mol %). The reaction mixture was heated at reflux in $CHCl_3$ for 4 h under an argon atmosphere.

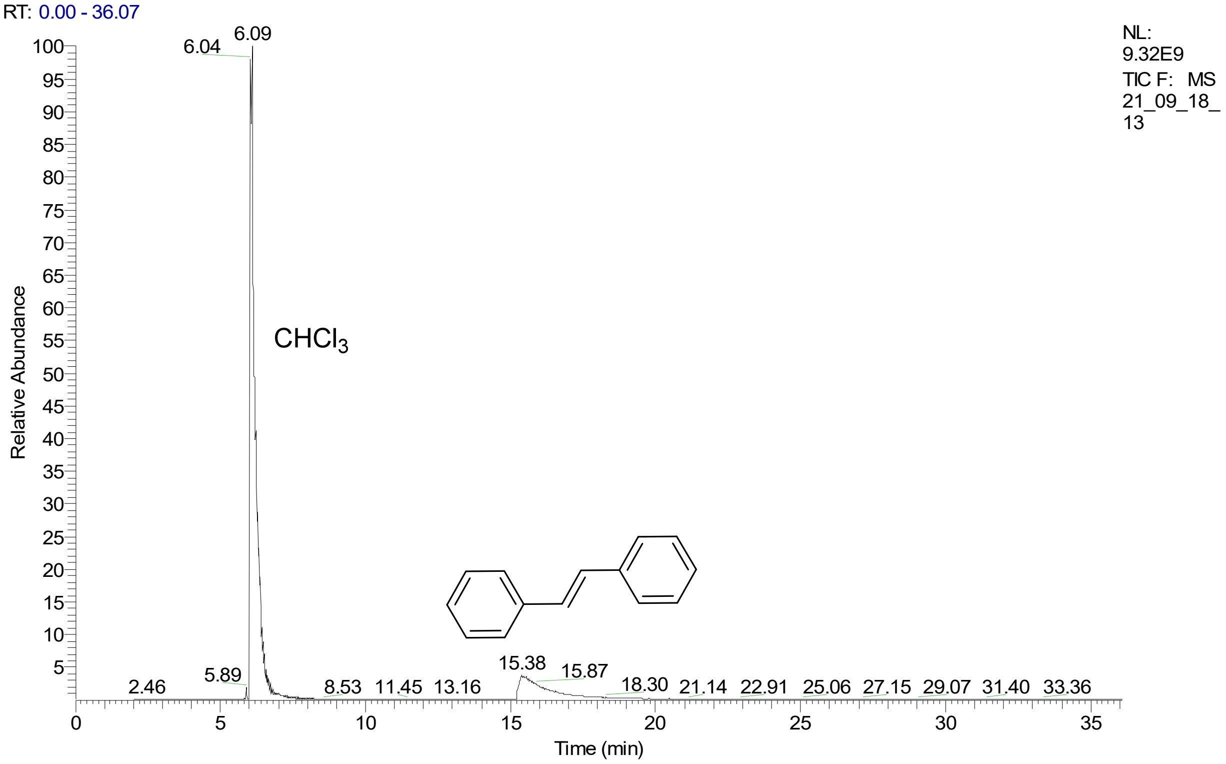

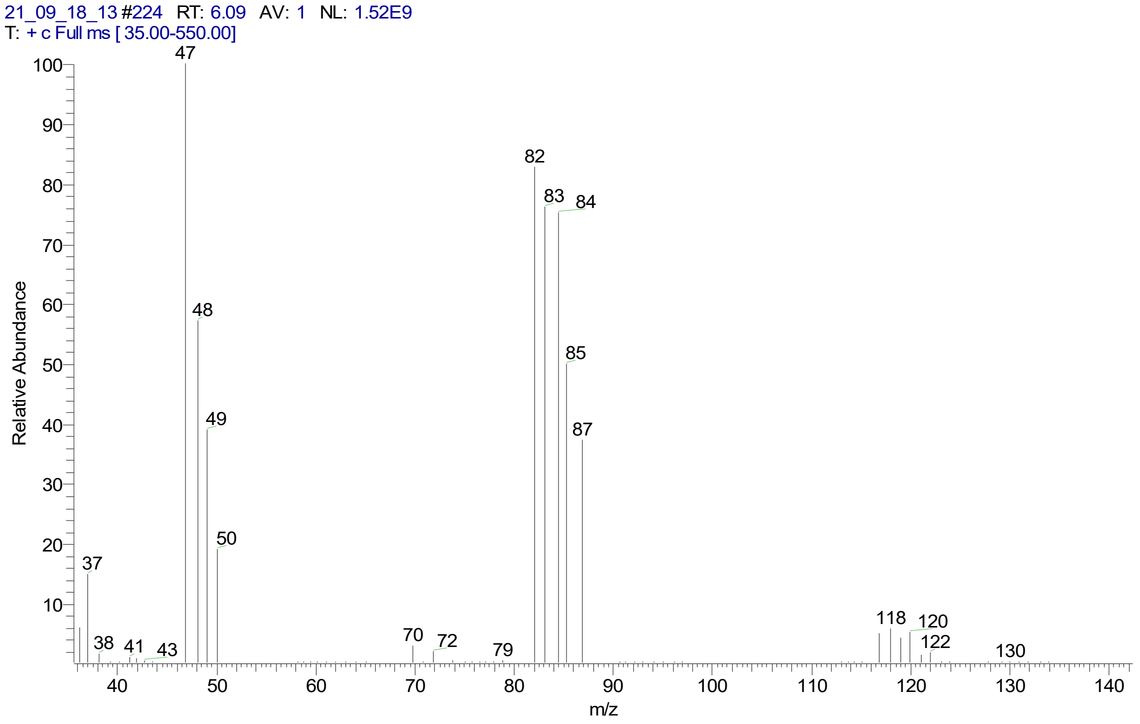
21_09_18_13 #224 RT: 6.09 AV: 1 NL: 1.52E9
T: + c Full ms [ 35.00-550.00]
Relative Abundance
m/z
47
82
83
84
48
85
49
87
50
37
38
41
43
70
72
79
118
120
122
130

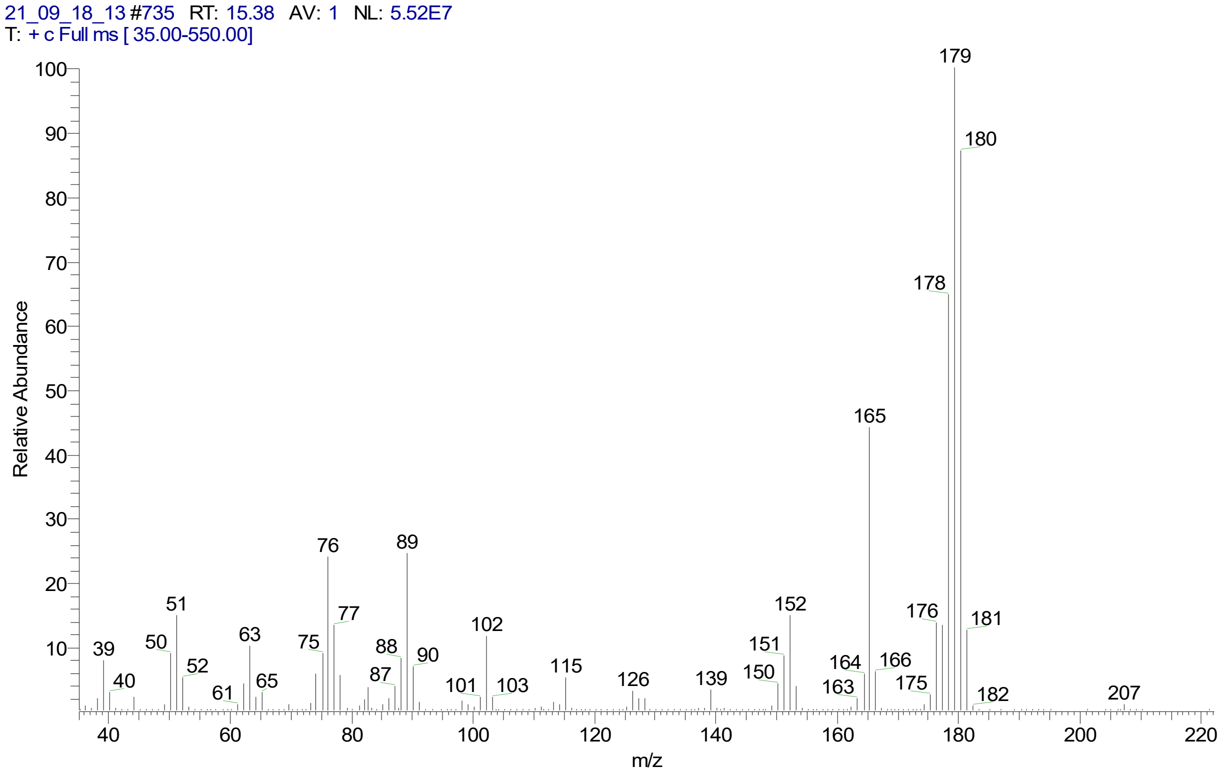
21_09_18_13 #735 RT: 15.38 AV: 1 NL: 5.52E7
T: + c Full ms [ 35.00-550.00]
Relative Abundance
m/z
179
180
178
165
76
89
152
51
77
102
176
181
39
50
63
75
88
151
90
52
40
65
87
115
126
139
150
164
166
61
101
103
163
175
182
207

**Entry 19.**

Starting compound – **12**. Catalyst – **11b** (0.01 mol %). The reaction mixture was heated at reflux in $CHCl_3$ for 4 h under an argon atmosphere.

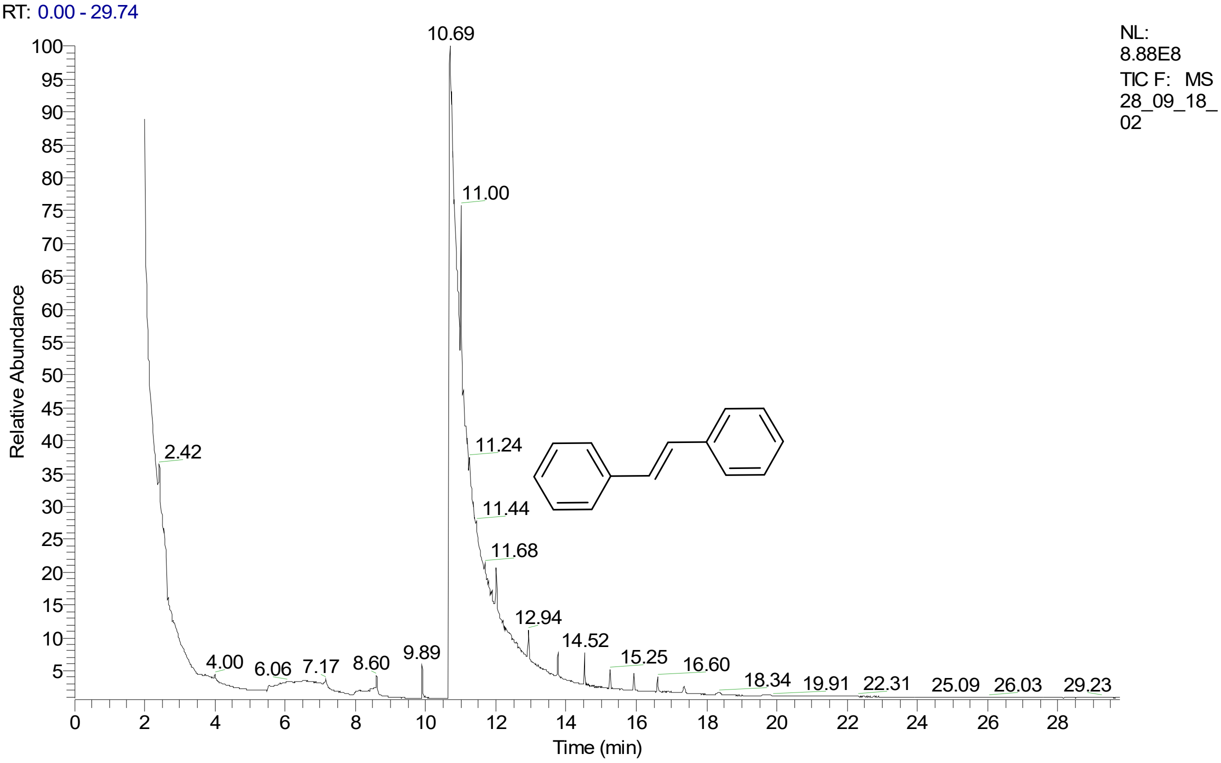


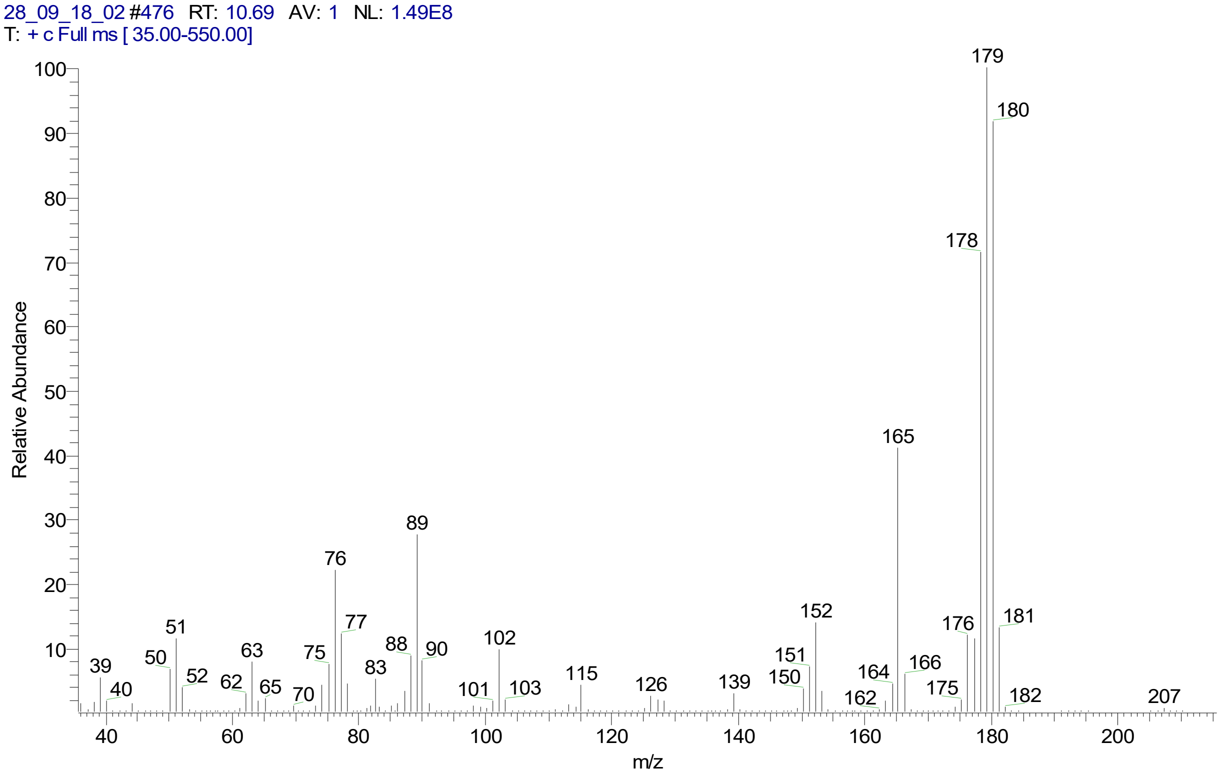

**Entry 20.**

Starting compound – **12**. Catalyst – **11b** (0.1 mol %). The reaction mixture was heated at reflux in $CHCl_3$ for 4 h.

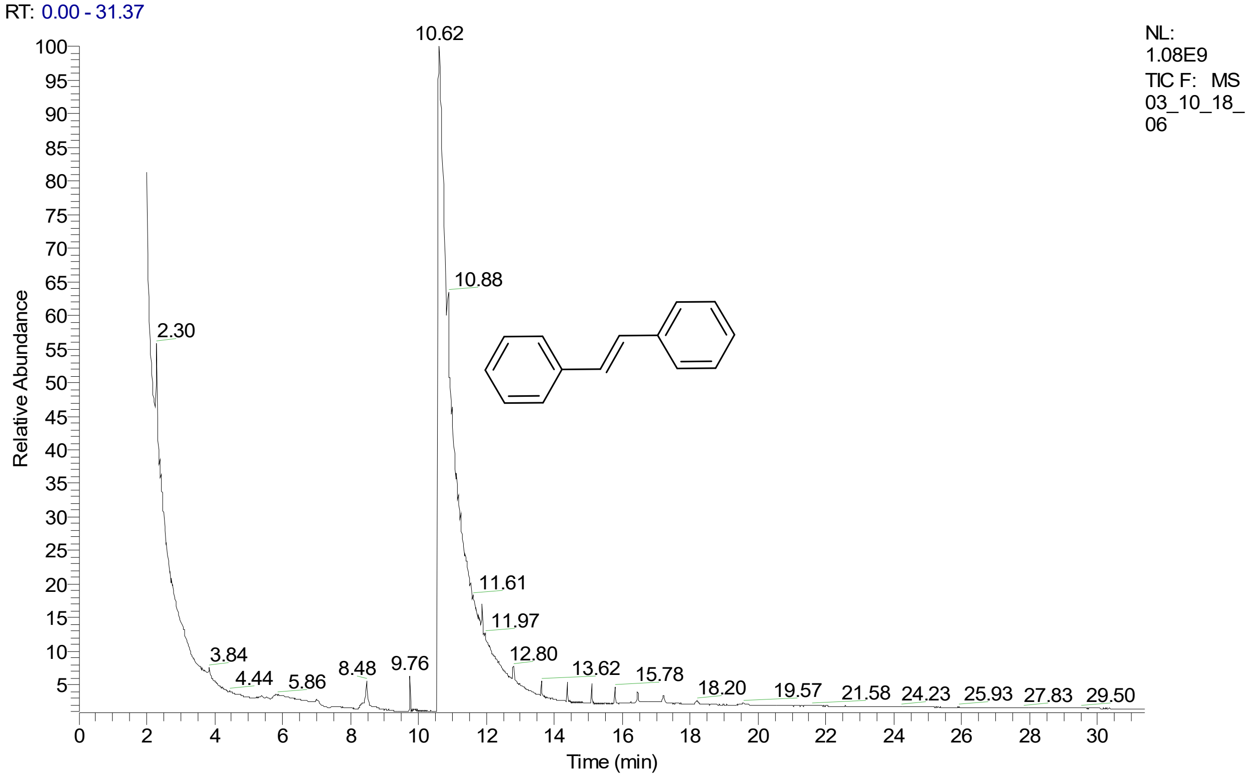


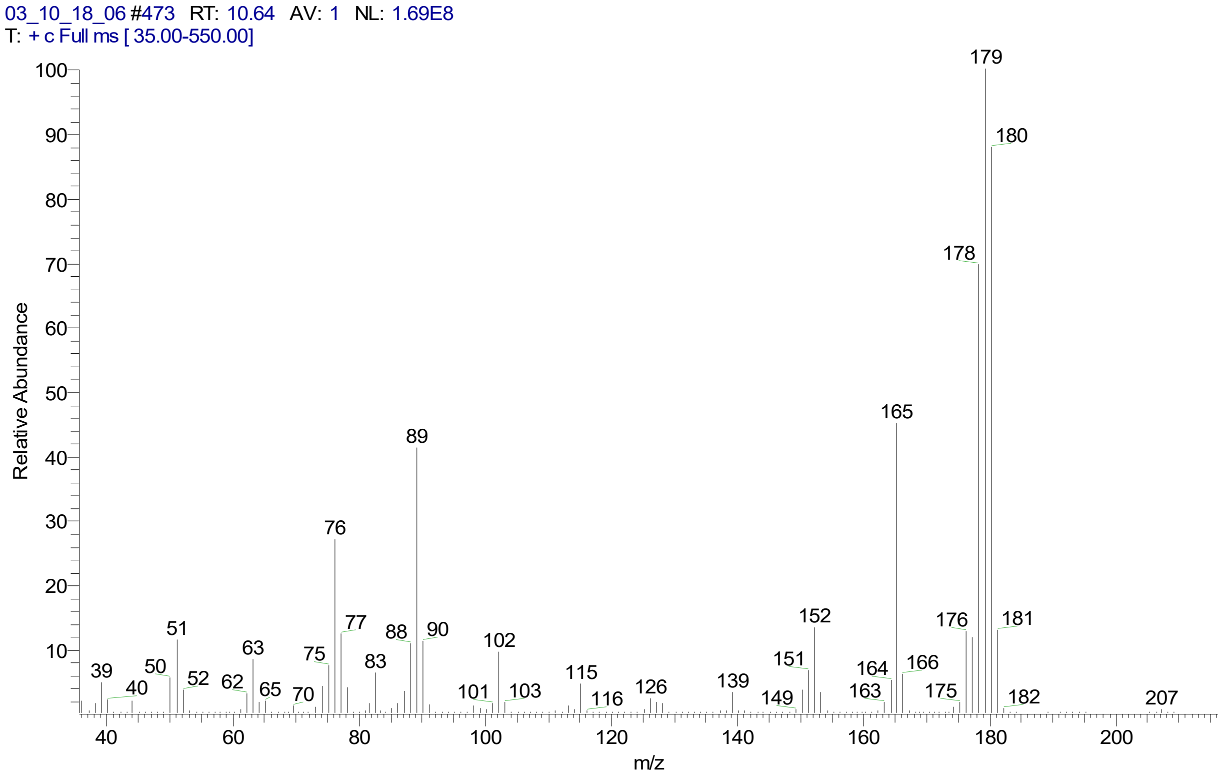

**Entry 24.**
Starting compound – **14**. Catalyst – **11b** (0.1 mol %). The reaction mixture was heated at reflux in $CHCl_3$ for 4 h under an argon atmosphere.

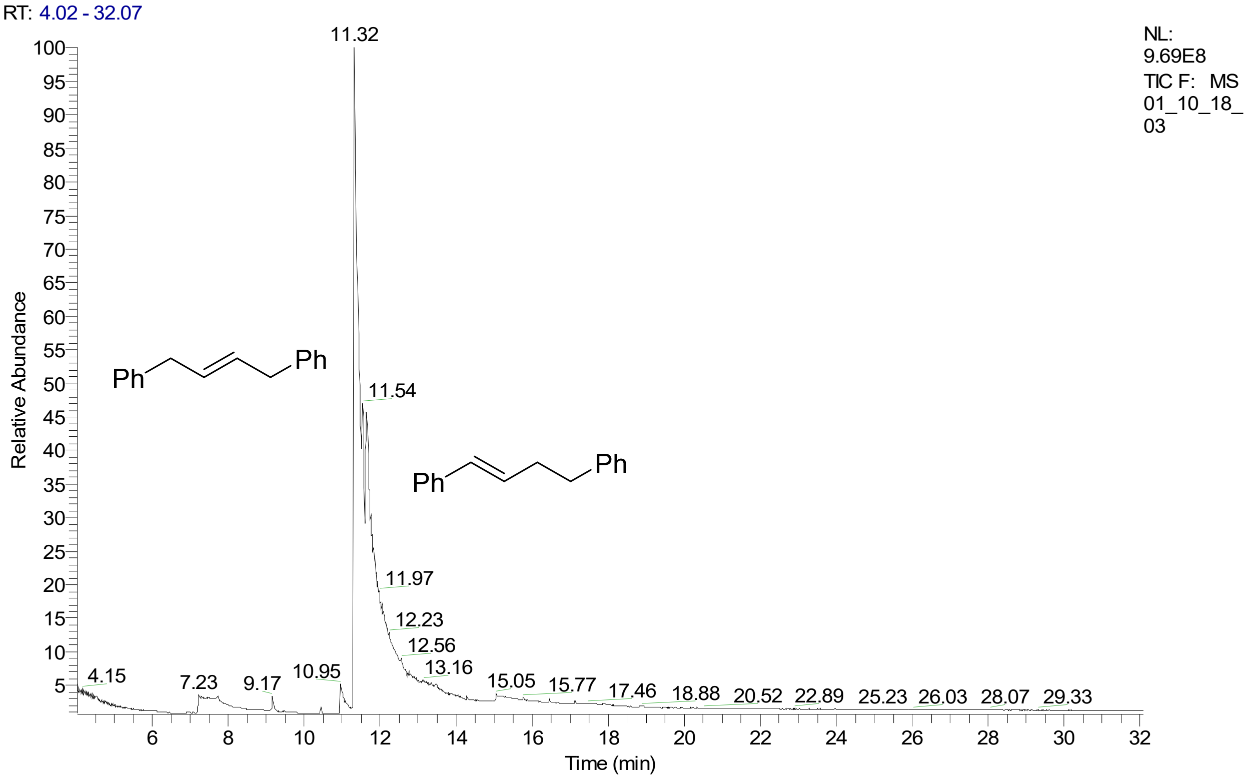


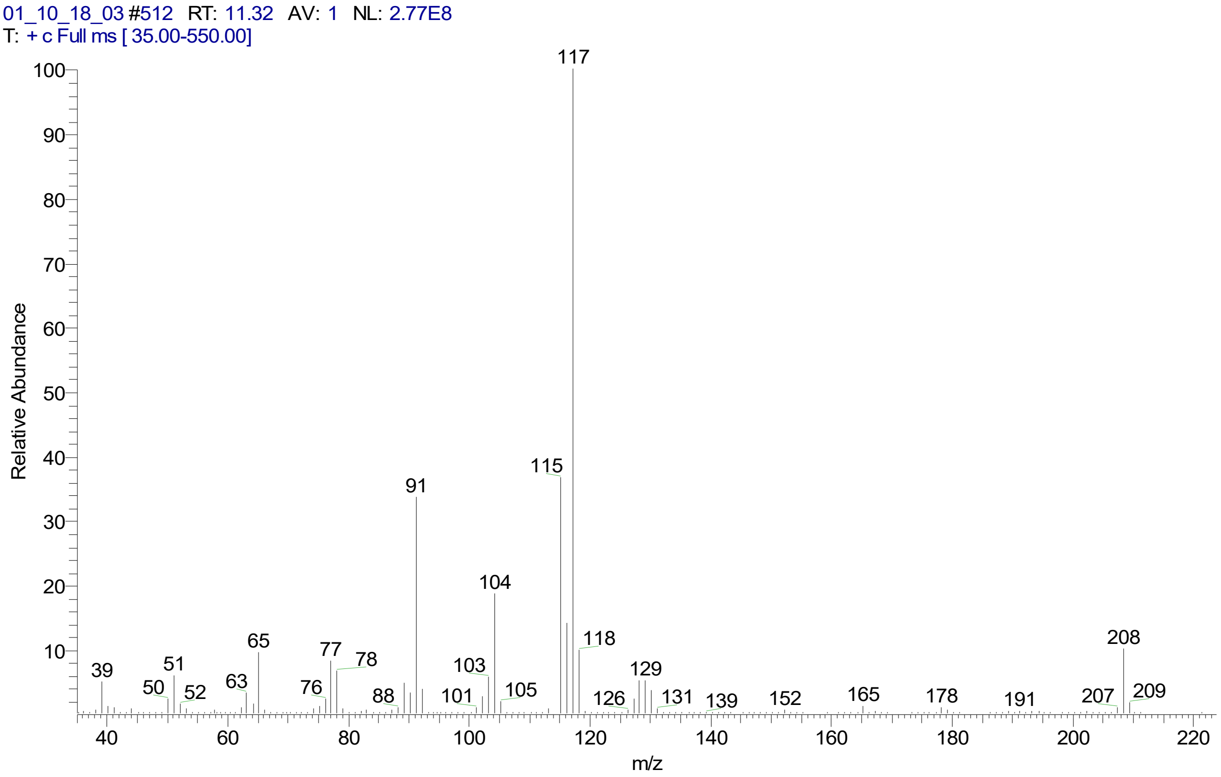

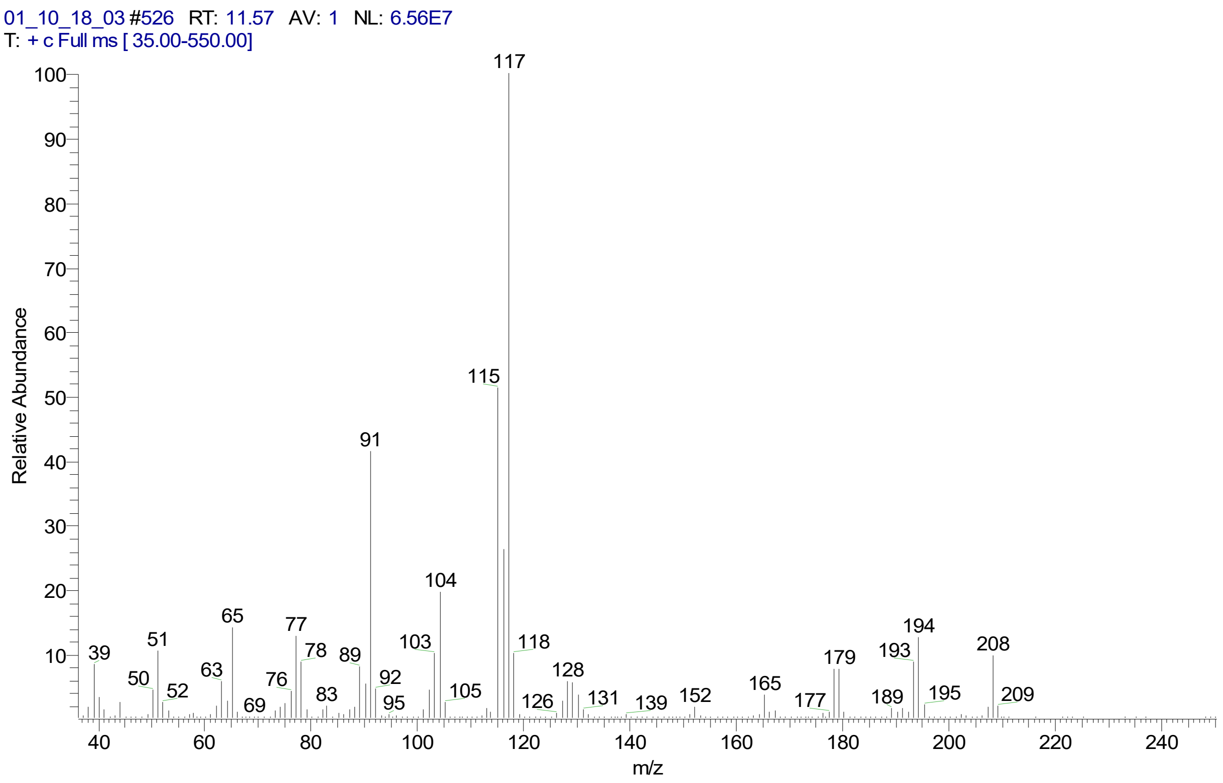


**Entry 25.**

Starting compound – **17**. Catalyst – **11a** (0.1 mol %). The reaction mixture was heated at reflux in $CHCl_3$ for 4 h.

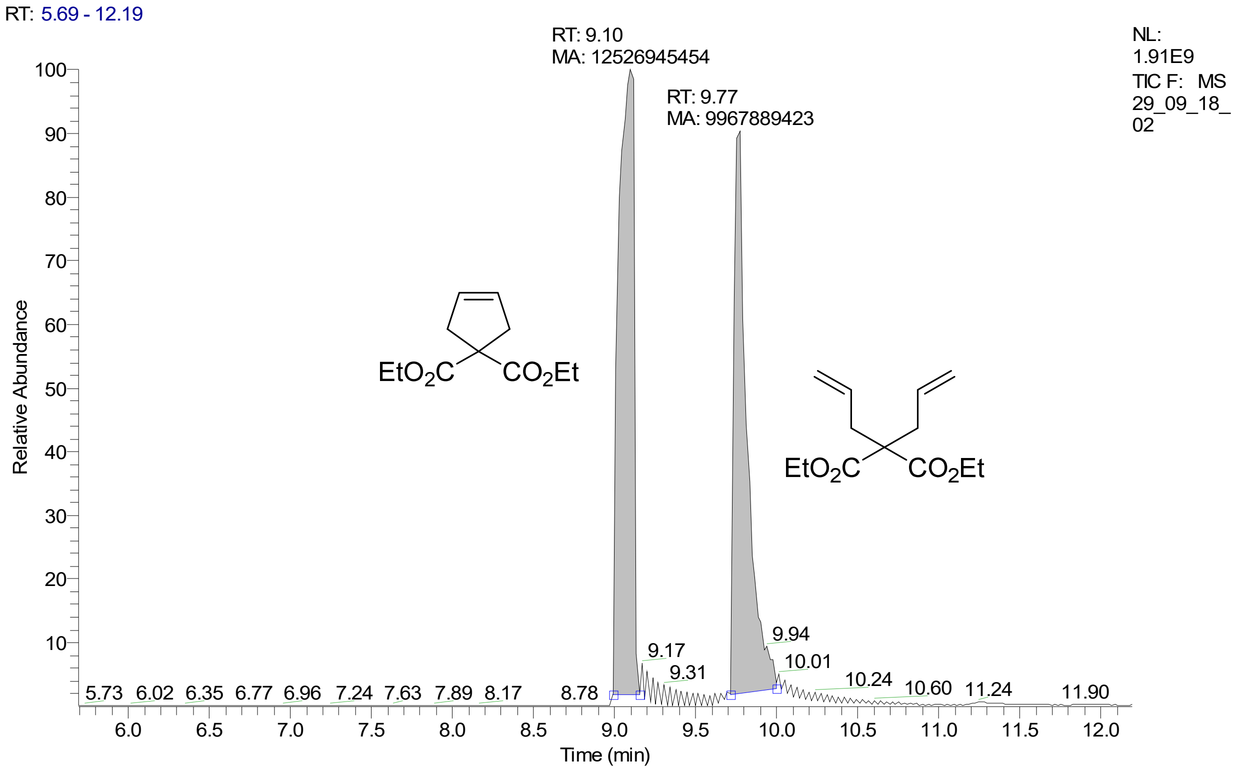

29_09_18_02 #328 RT: 9.10 AV: 1 NL: 2.34E8
T: + c Full ms [ 35.00-550.00]

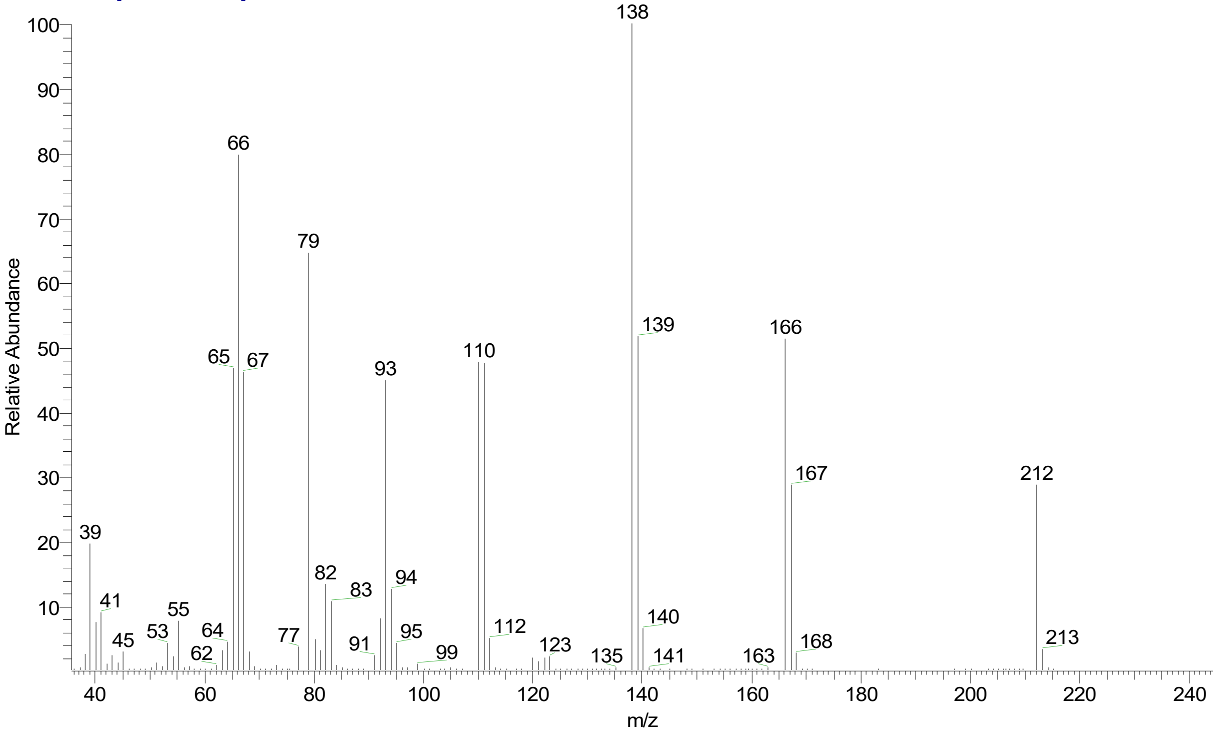


29_09_18_02 #366 RT: 9.77 AV: 1 NL: 1.42E8
T: + c Full ms [ 35.00-550.00]

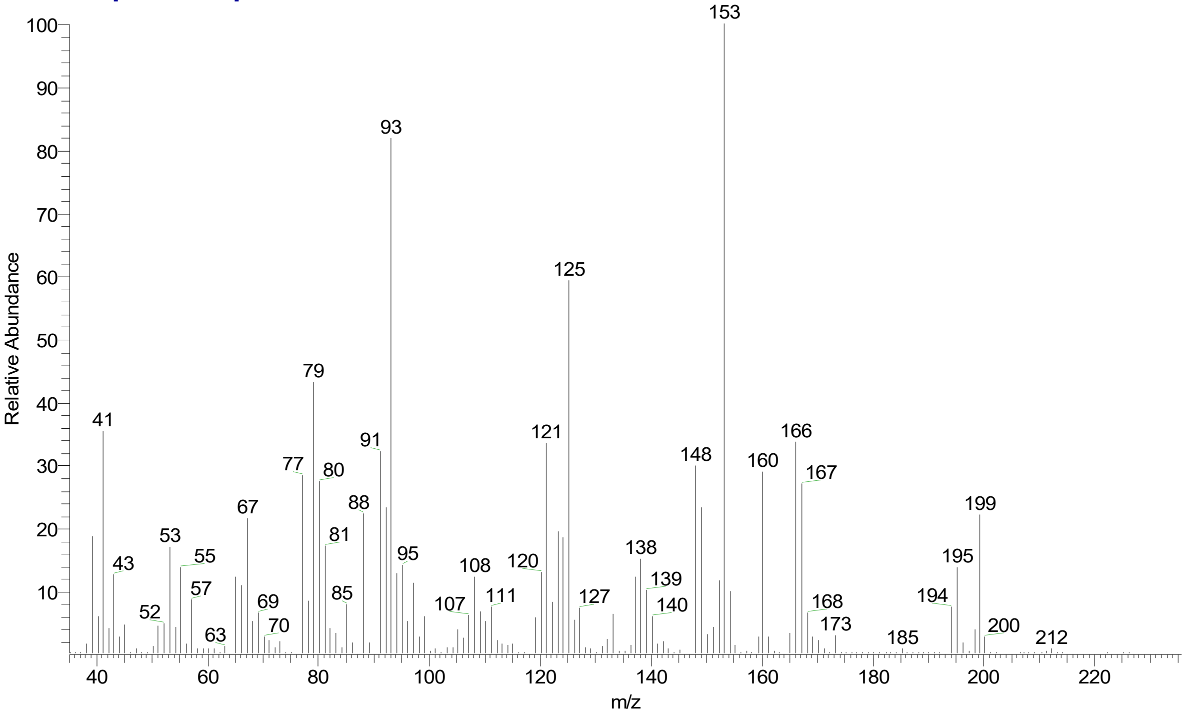

**Entry 26.**

Starting compound – **17**. Catalyst – **11b** (0.1 mol %). The reaction mixture was heated at reflux in $CHCl_3$ for 4 h.

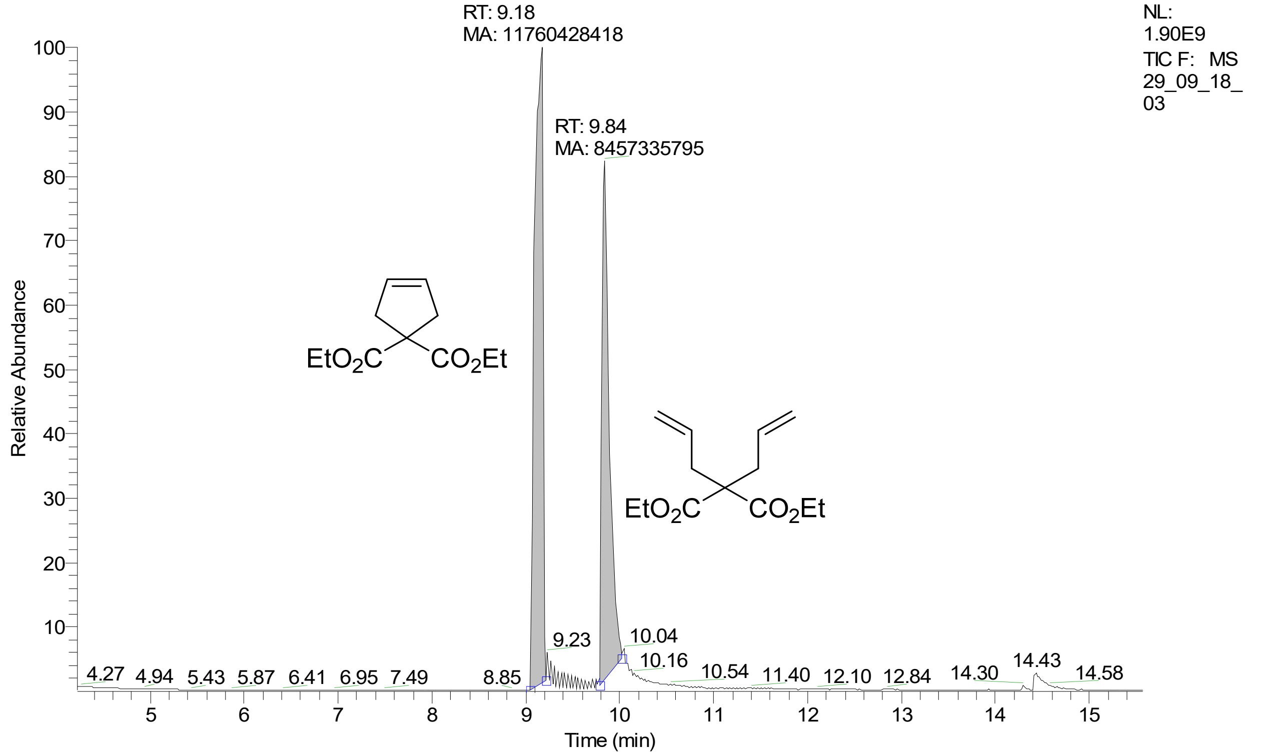


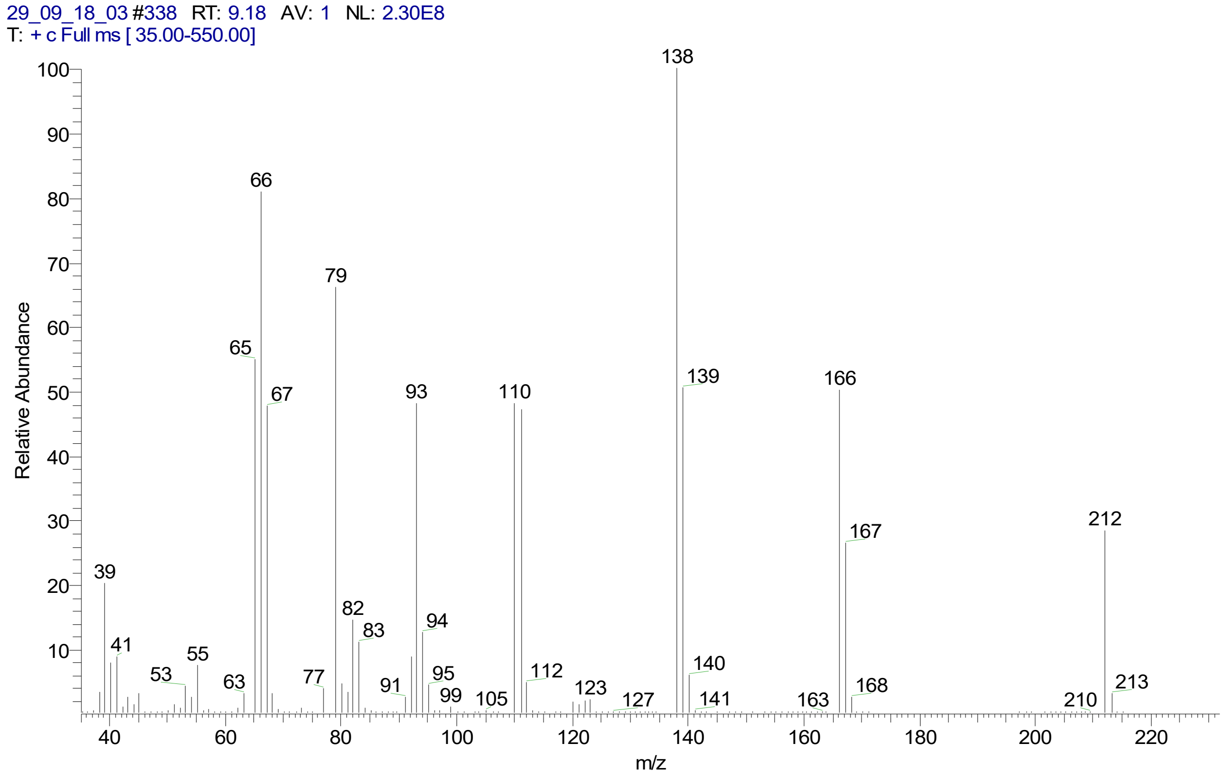

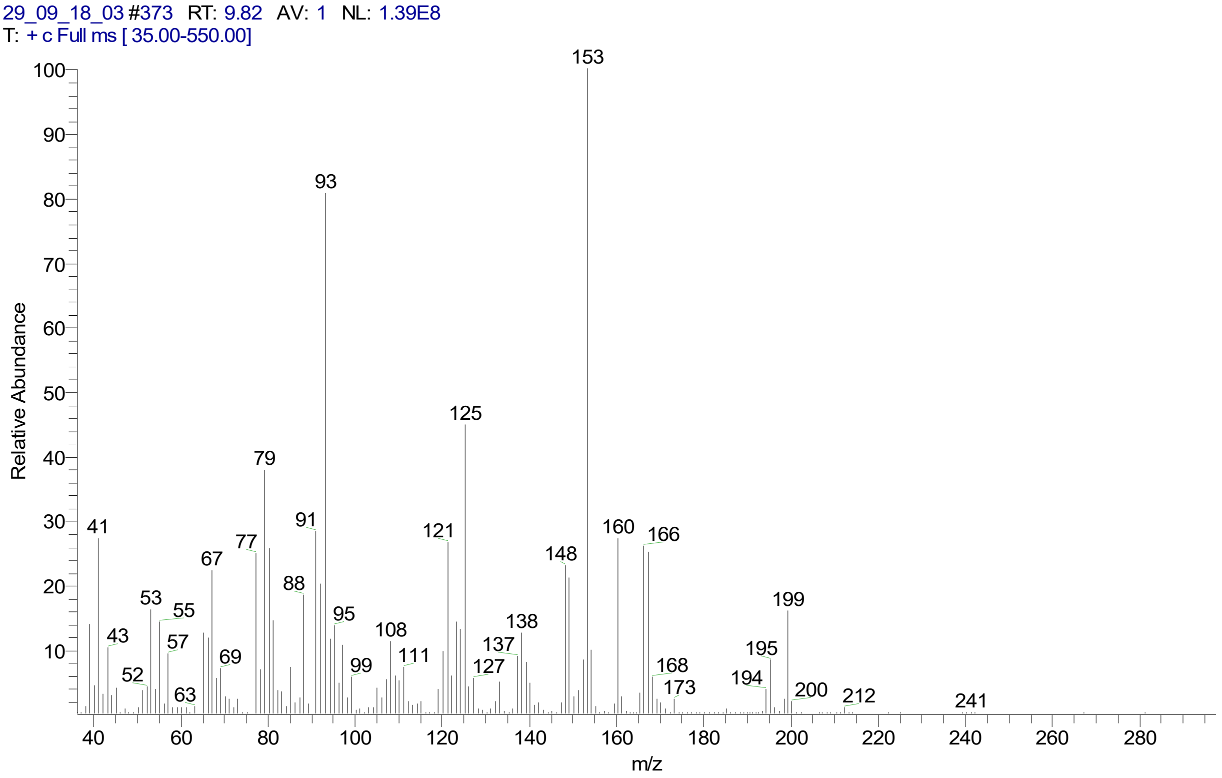


## Entry 27.

Starting compound – **17**. Catalyst – **11b** (0.1 mol %). The reaction mixture was heated at reflux in $CHCl_3$ for 4 h.

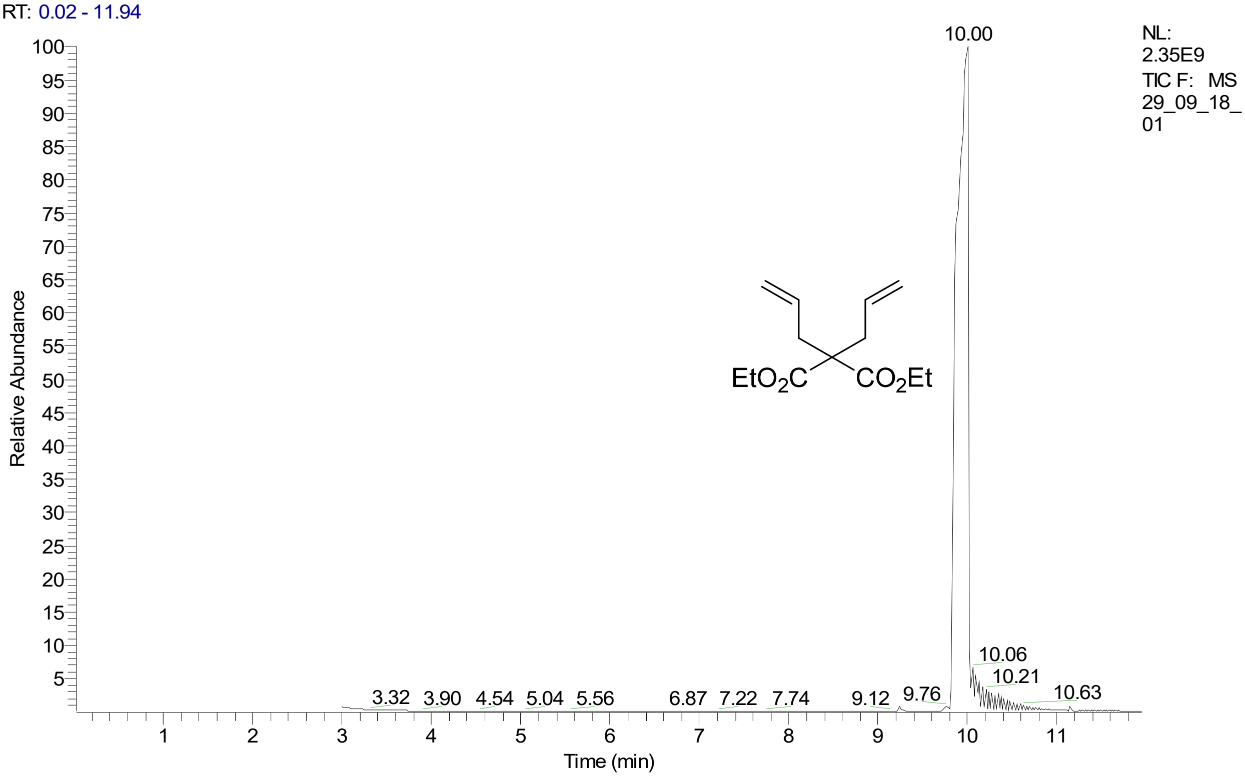

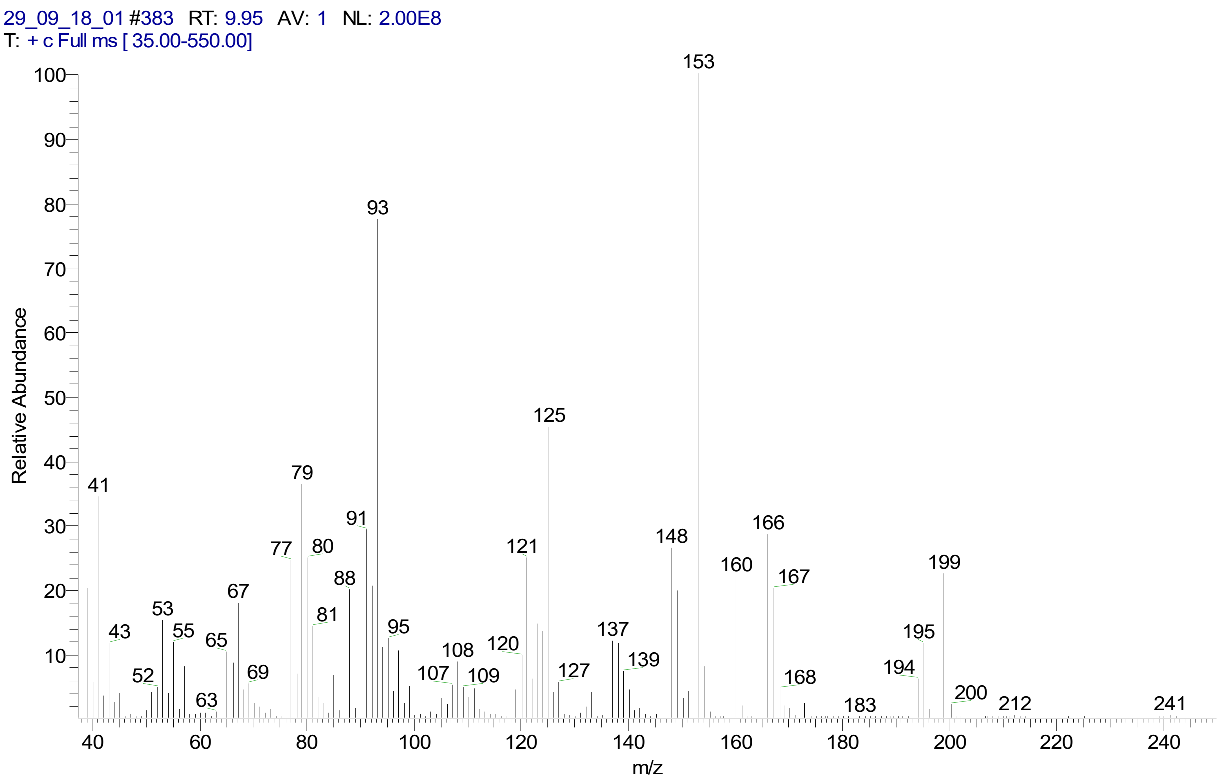


**Entry 28.**

Starting compound – **17**. Catalyst – **11b** (0.1 mol %). The reaction mixture was heated at reflux in $CHCl_3$ for 4 h.

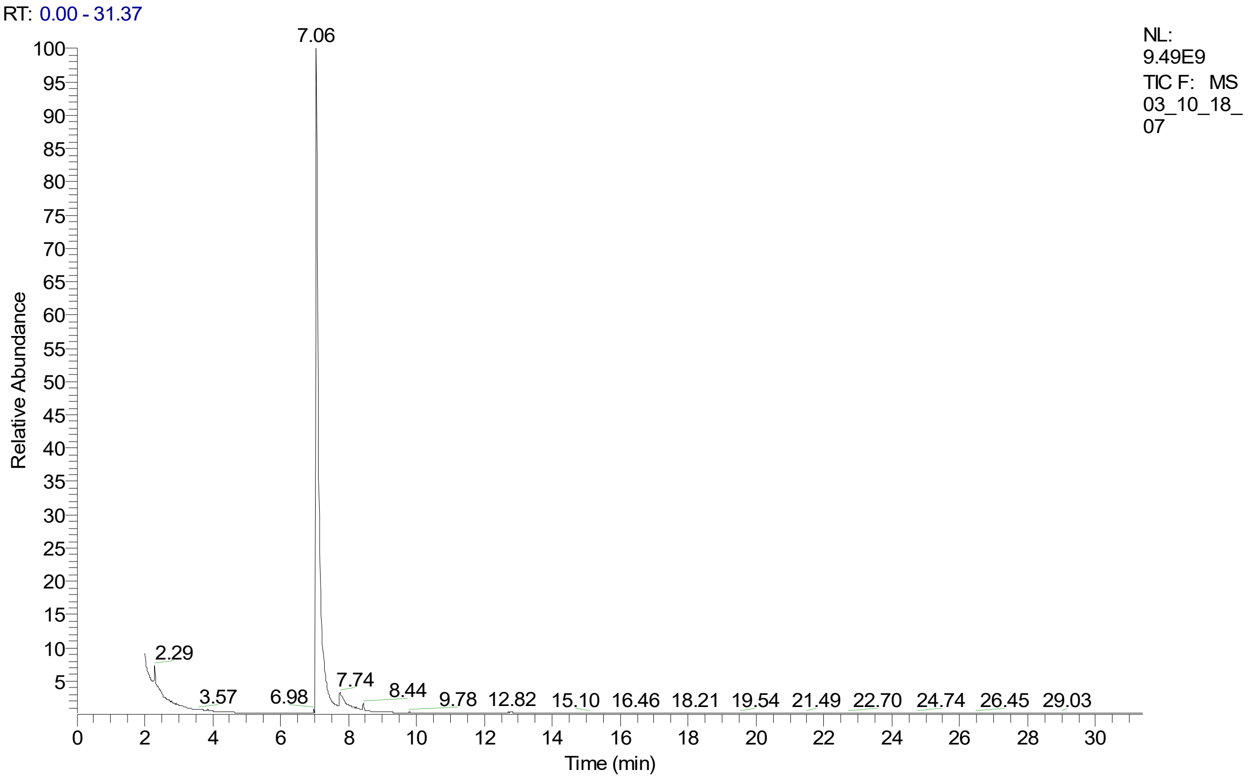

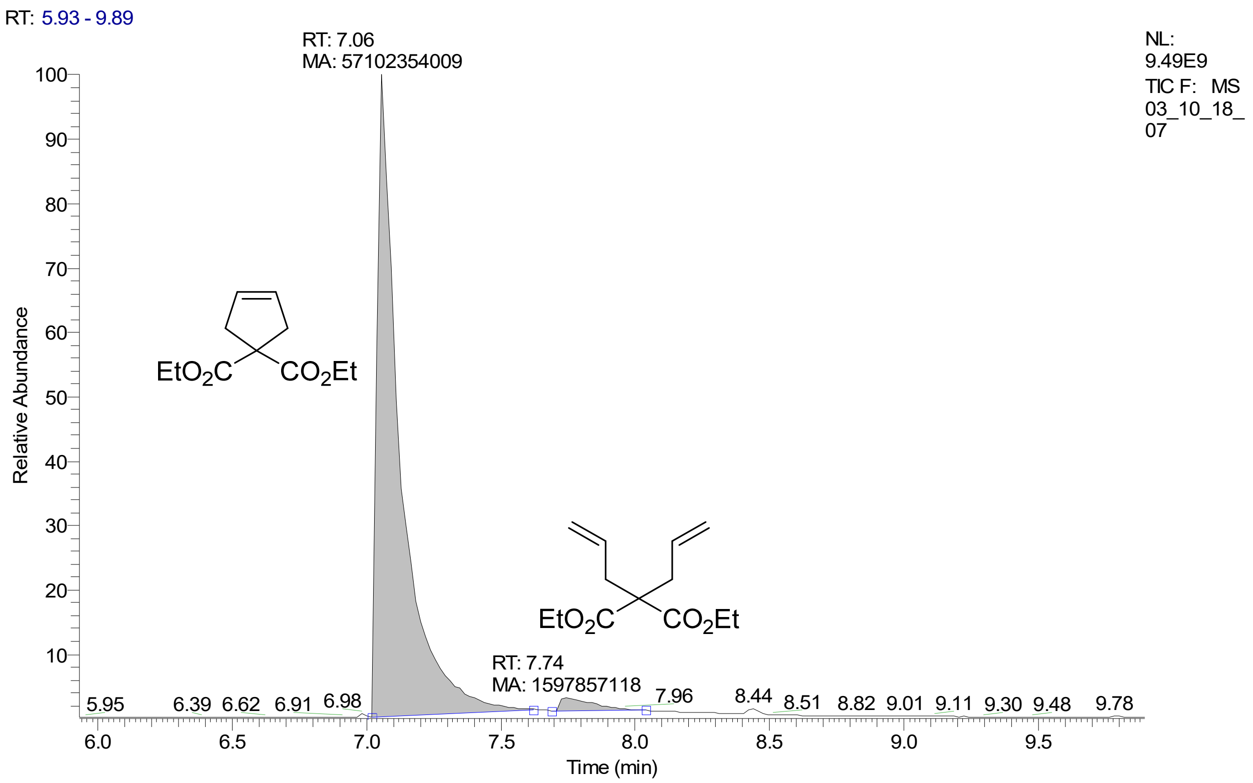
RT: 5.93 - 9.89
RT: 7.06
MA: 57102354009
NL:
9.49E9
TIC F: MS
03_10_18_
07
Relative Abundance
EtO2C
CO2Et
RT: 7.74
MA: 1597857118
Time (min)

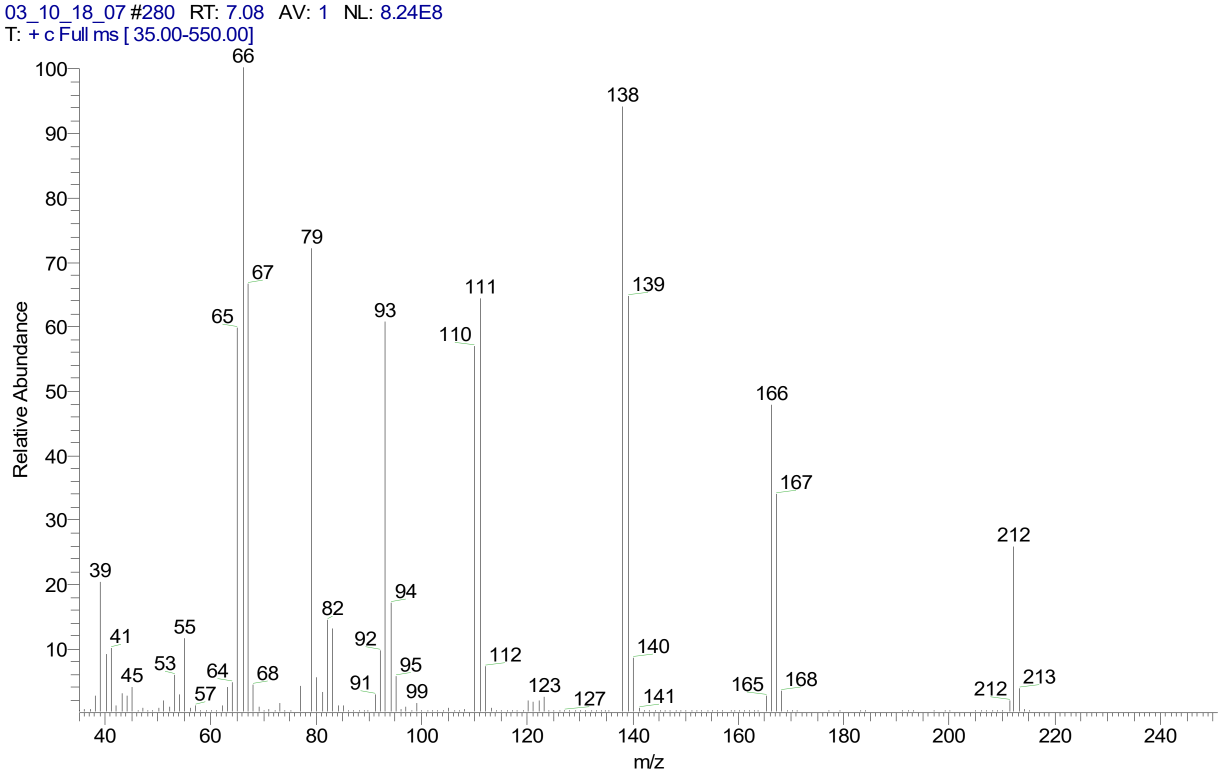
03_10_18_07 #280 RT: 7.08 AV: 1 NL: 8.24E8
T: + c Full ms [ 35.00-550.00]
Relative Abundance
m/z

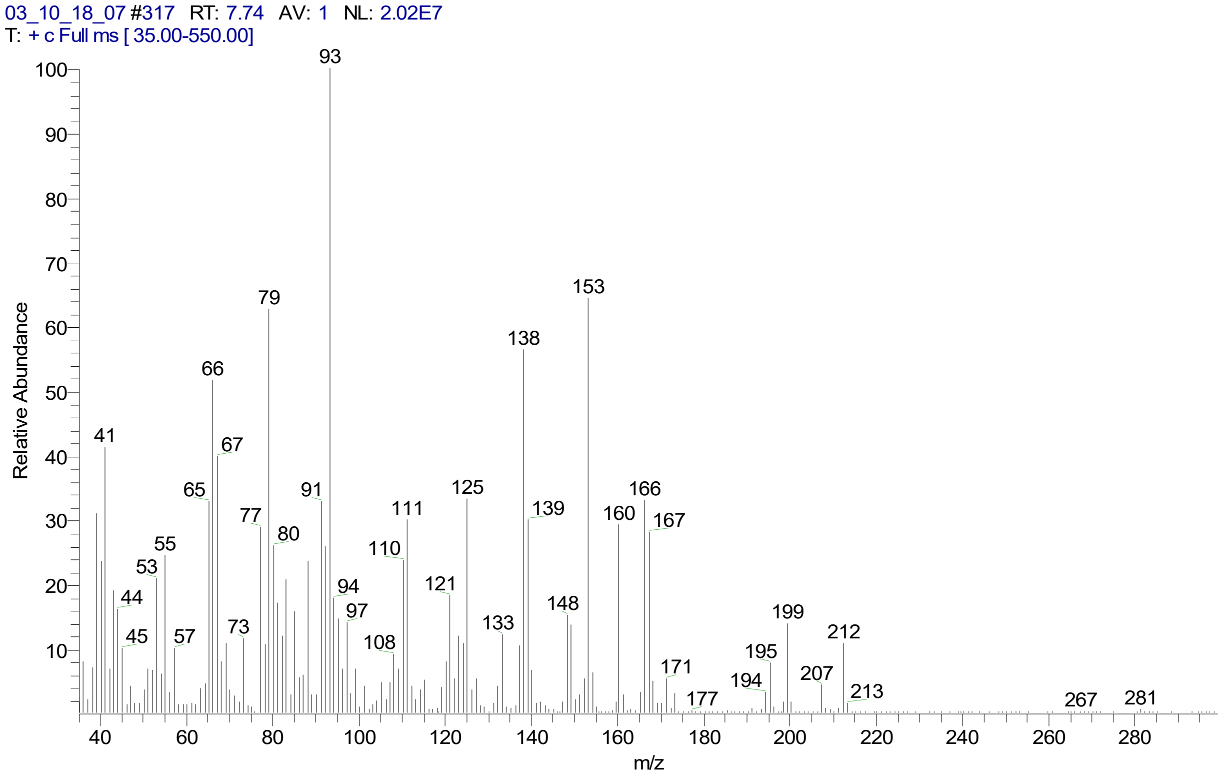


**Entry 29.**
Starting compound – **17**. Catalyst – **11d** (0.1 mol %). The reaction mixture was heated at reflux in $CHCl_3$ for 6 h under an argon atmosphere.

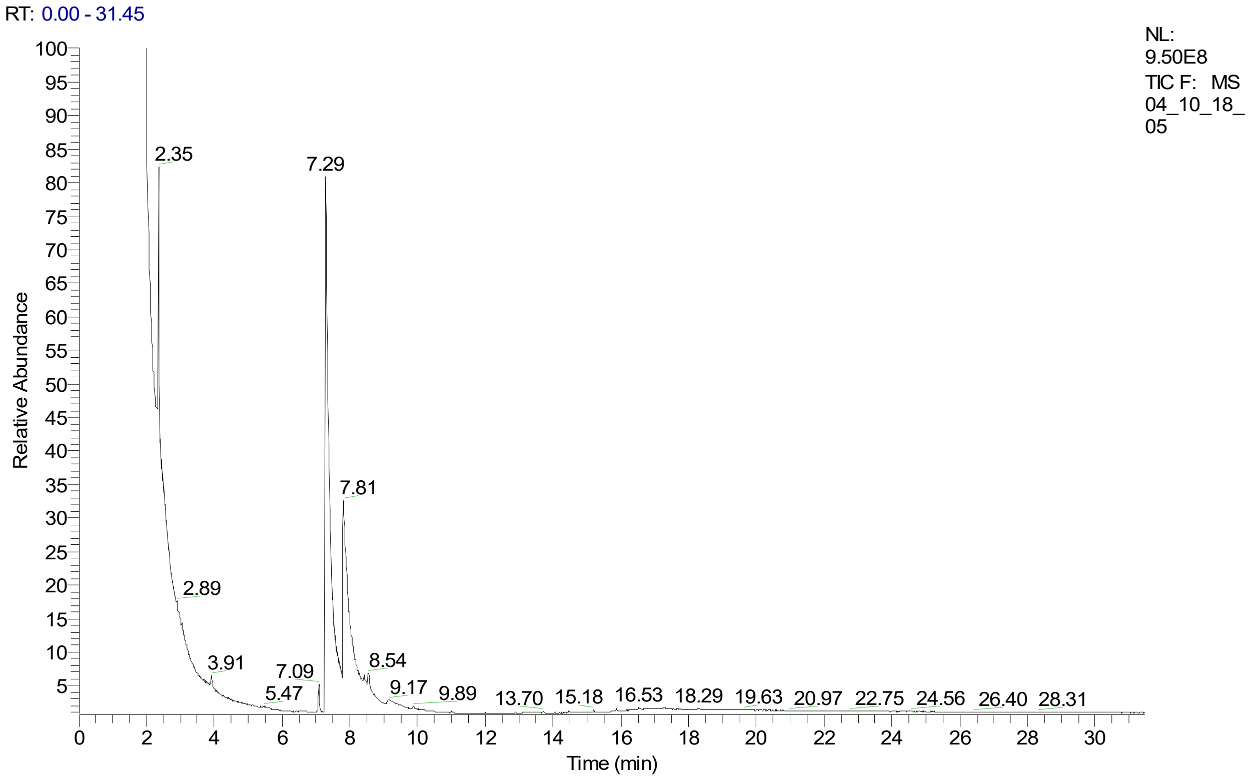

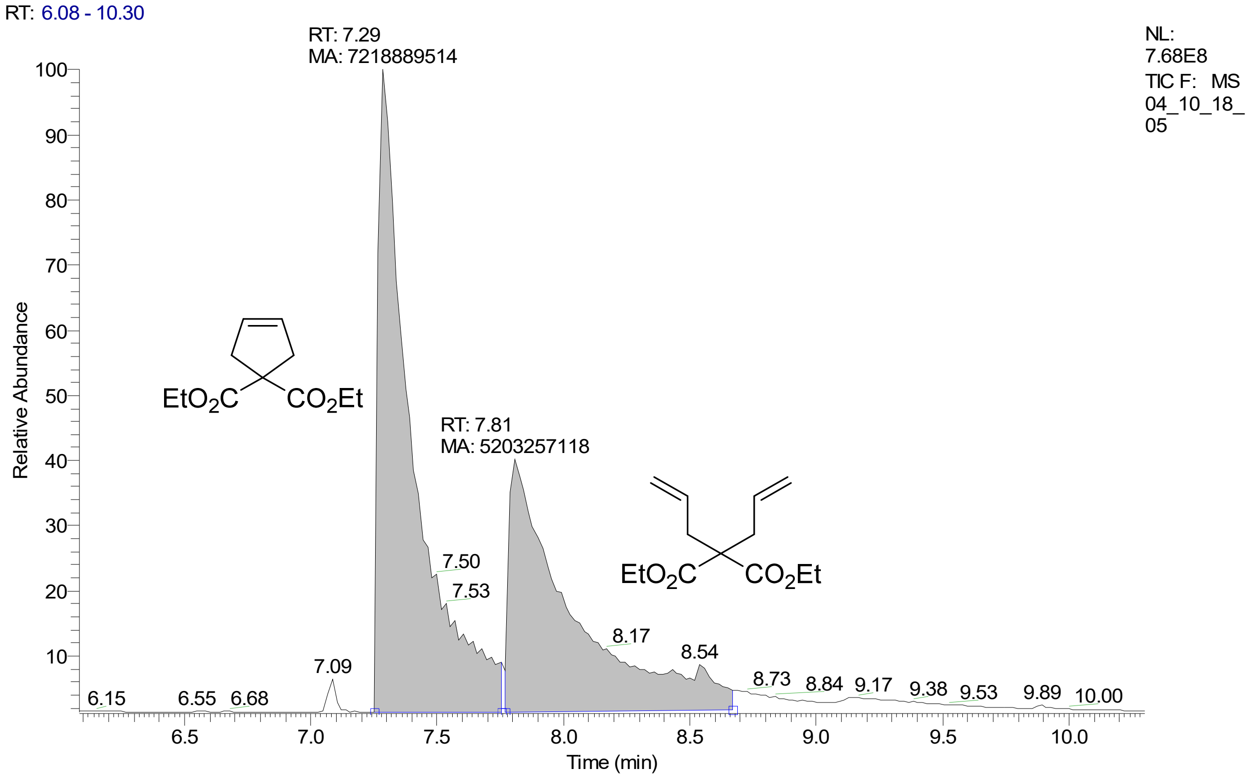
RT: 6.08 - 10.30
RT: 7.29
MA: 7218889514
NL:
7.68E8
TIC F: MS
04_10_18_
05
EtO2C
CO2Et
RT: 7.81
MA: 5203257118
EtO2C
CO2Et
Relative Abundance
Time (min)
7.09
7.50
7.53
8.17
8.54
8.73
8.84
9.17
9.38
9.53
9.89
10.00
6.15
6.55
6.68

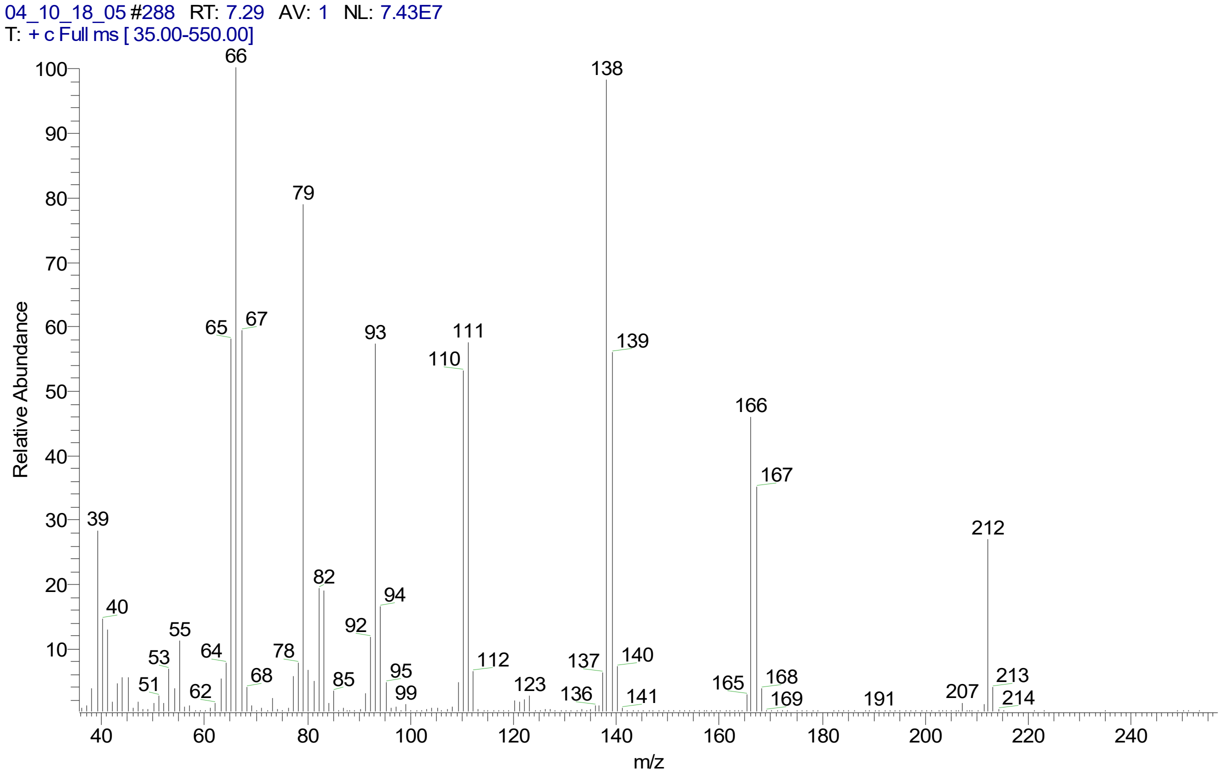
04_10_18_05 #288 RT: 7.29 AV: 1 NL: 7.43E7
T: + c Full ms [ 35.00-550.00]
Relative Abundance
m/z
66
138
79
65
67
93
111
110
139
166
167
212
39
82
94
40
92
55
53
64
78
112
137
140
51
62
68
85
95
99
123
136
141
165
168
169
191
207
213
214

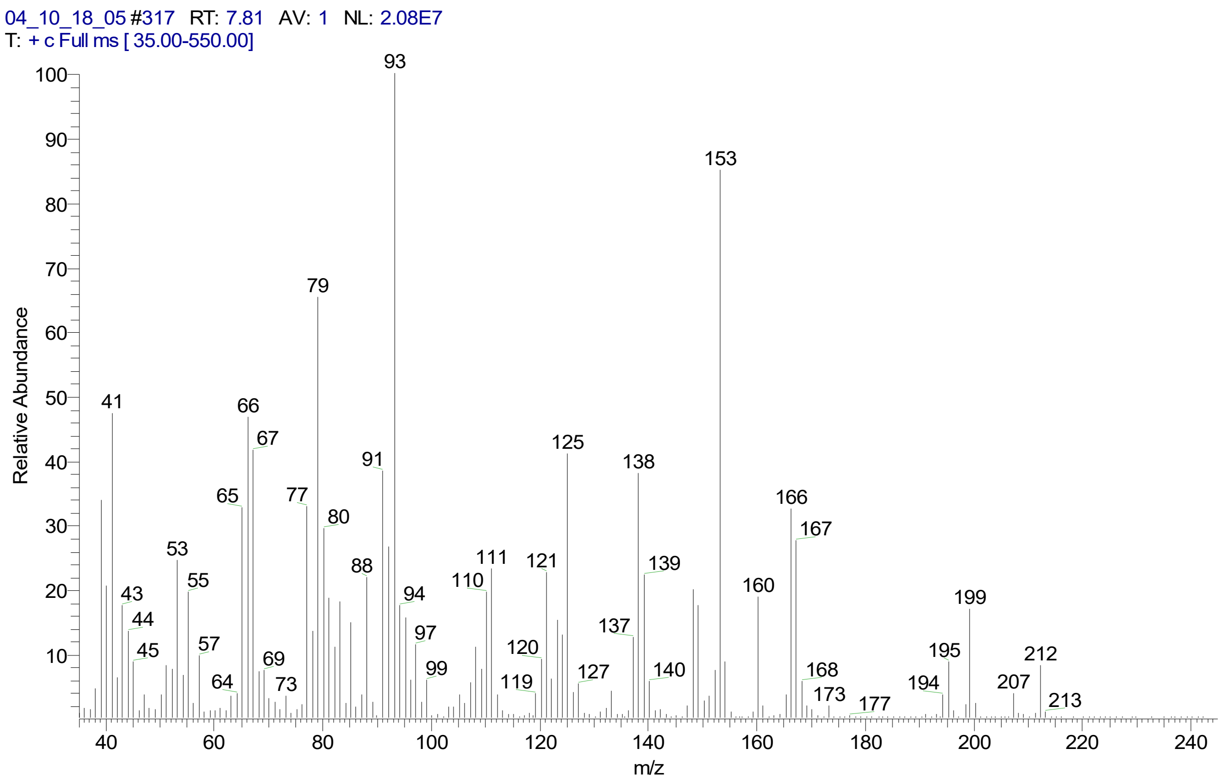


## Entry 30.

Starting compound – **17**. Catalyst – **11d** (0.1 mol %). The reaction mixture was heated at reflux in $CHCl_3$ for 4 h under an argon atmosphere.

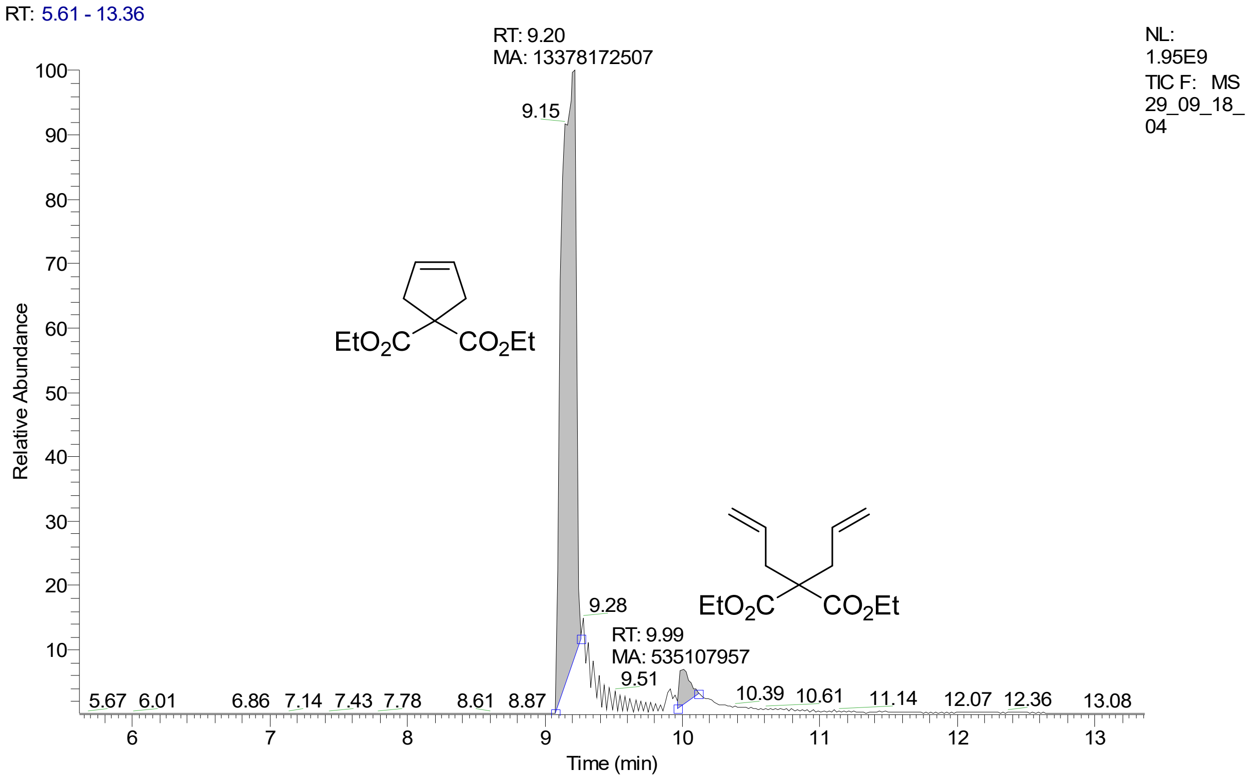

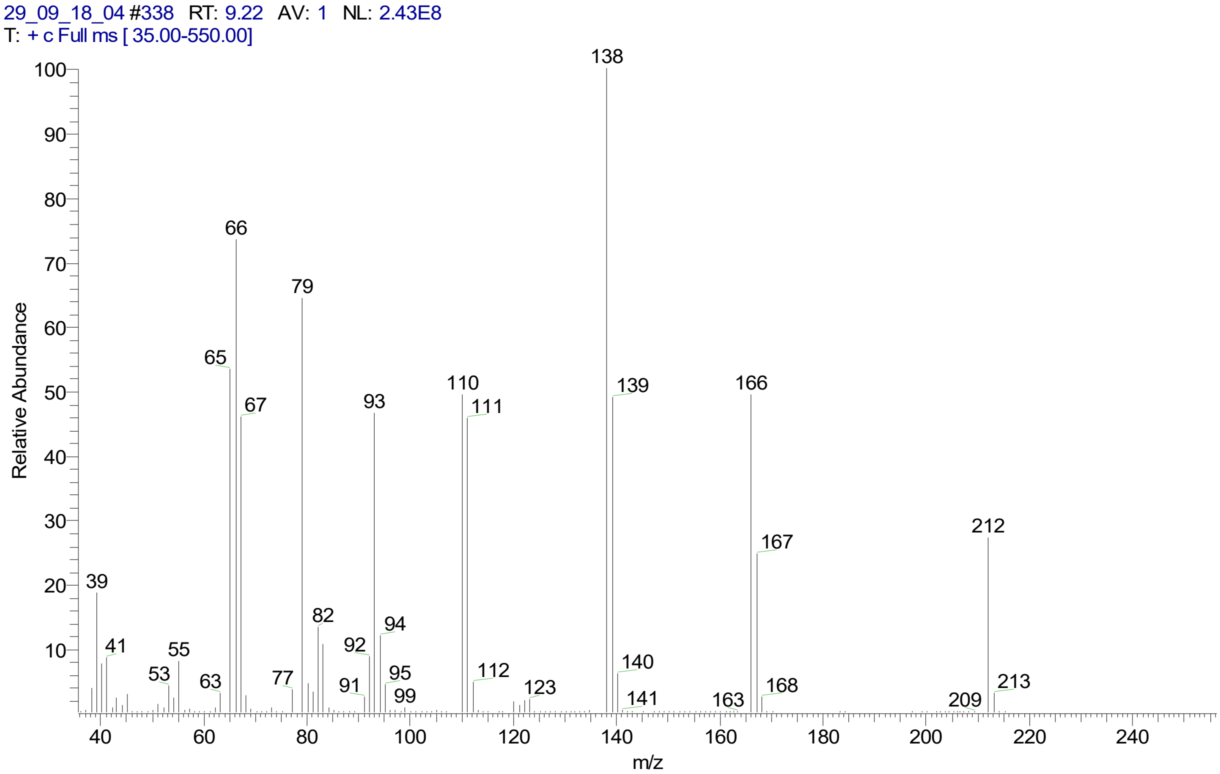
29_09_18_04 #338 RT: 9.22 AV: 1 NL: 2.43E8
T: + c Full ms [ 35.00-550.00]
Relative Abundance
m/z
138
66
79
65
67
110
139
166
93
111
212
167
39
82
94
41
55
92
53
63
77
95
91
99
112
123
140
141
163
168
209
213

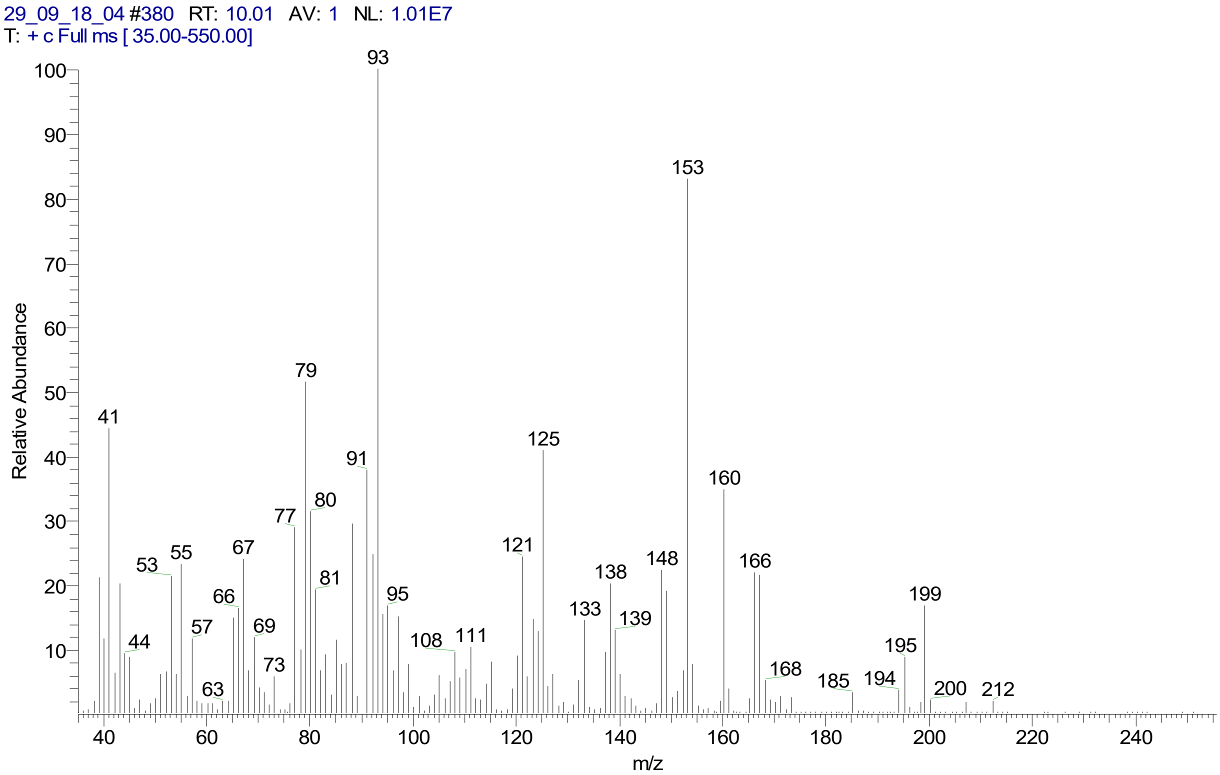
29_09_18_04 #380 RT: 10.01 AV: 1 NL: 1.01E7
T: + c Full ms [ 35.00-550.00]
Relative Abundance
m/z
93
153
79
41
125
91
160
77
80
53
55
67
121
138
148
166
81
66
95
199
133
139
57
69
44
108
111
195
73
63
168
185
194
200
212

**Entry 31.**

Starting compound – **17**. Catalyst – **11d** (0.01 mol %). The reaction mixture was heated at reflux in $CHCl_3$ for 4 h under an argon atmosphere.

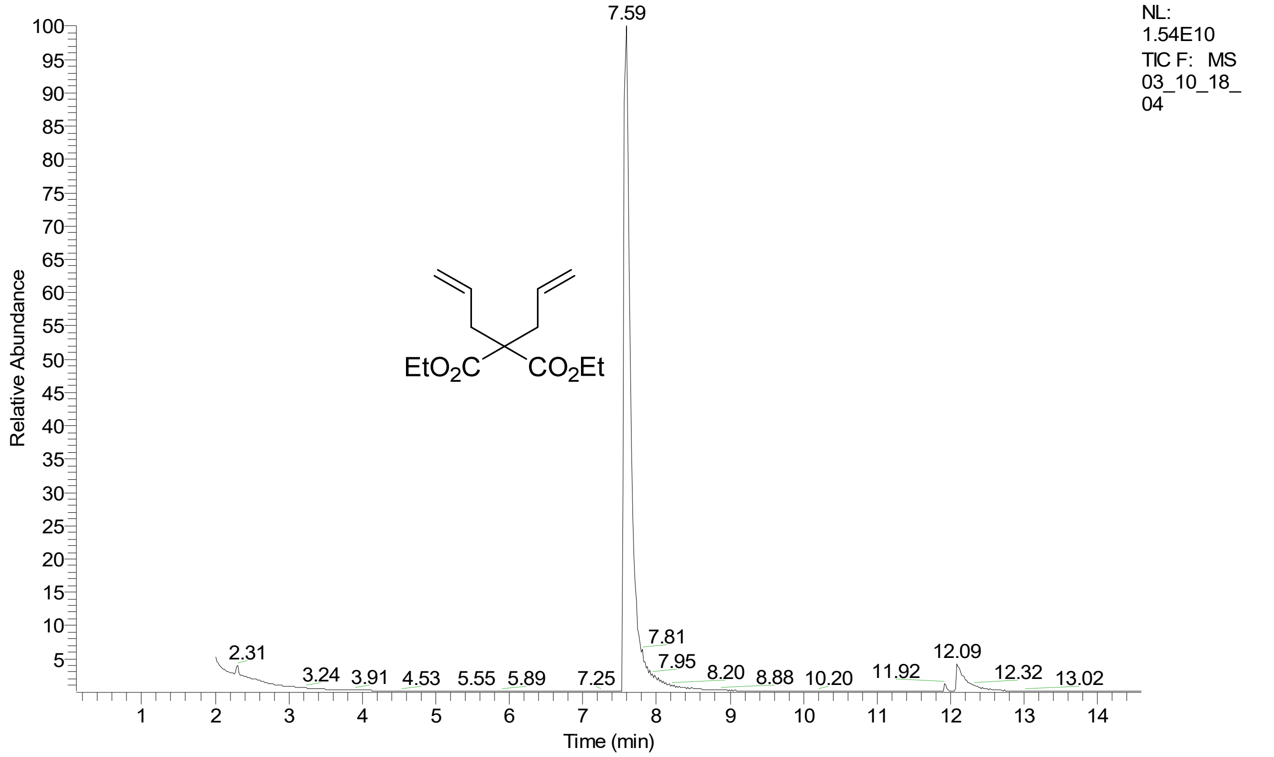


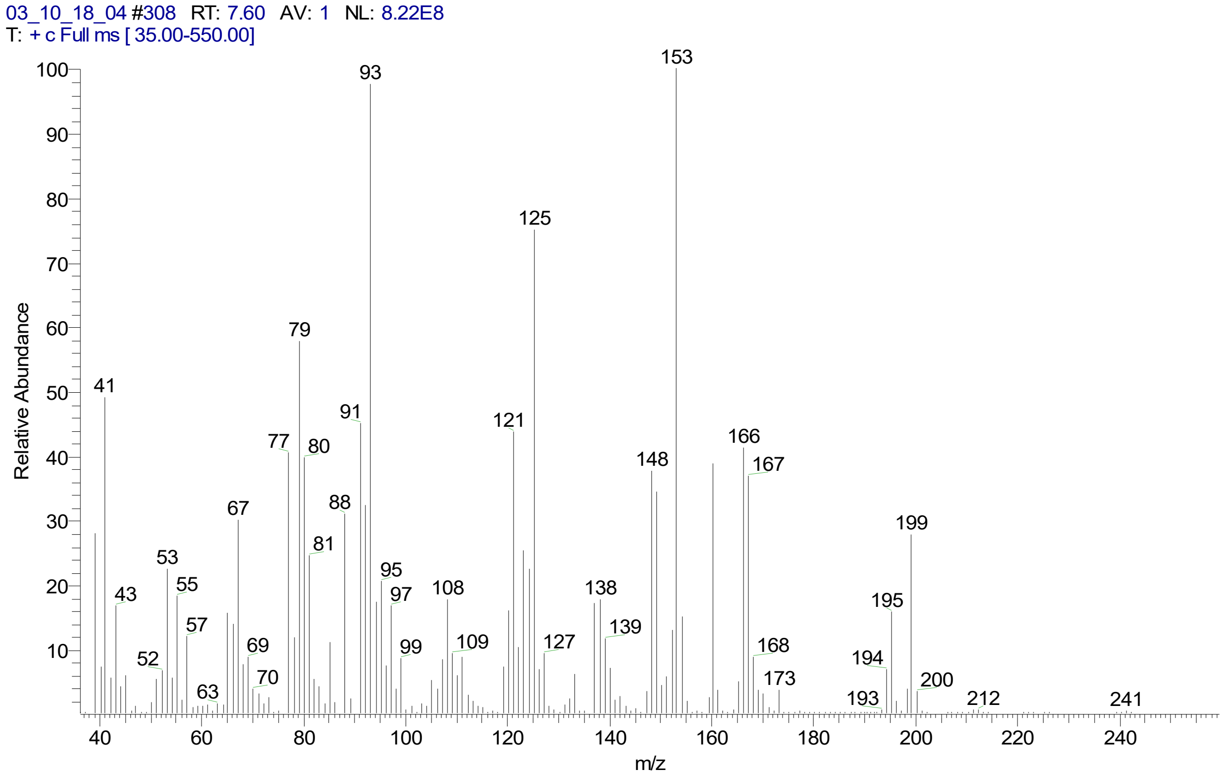

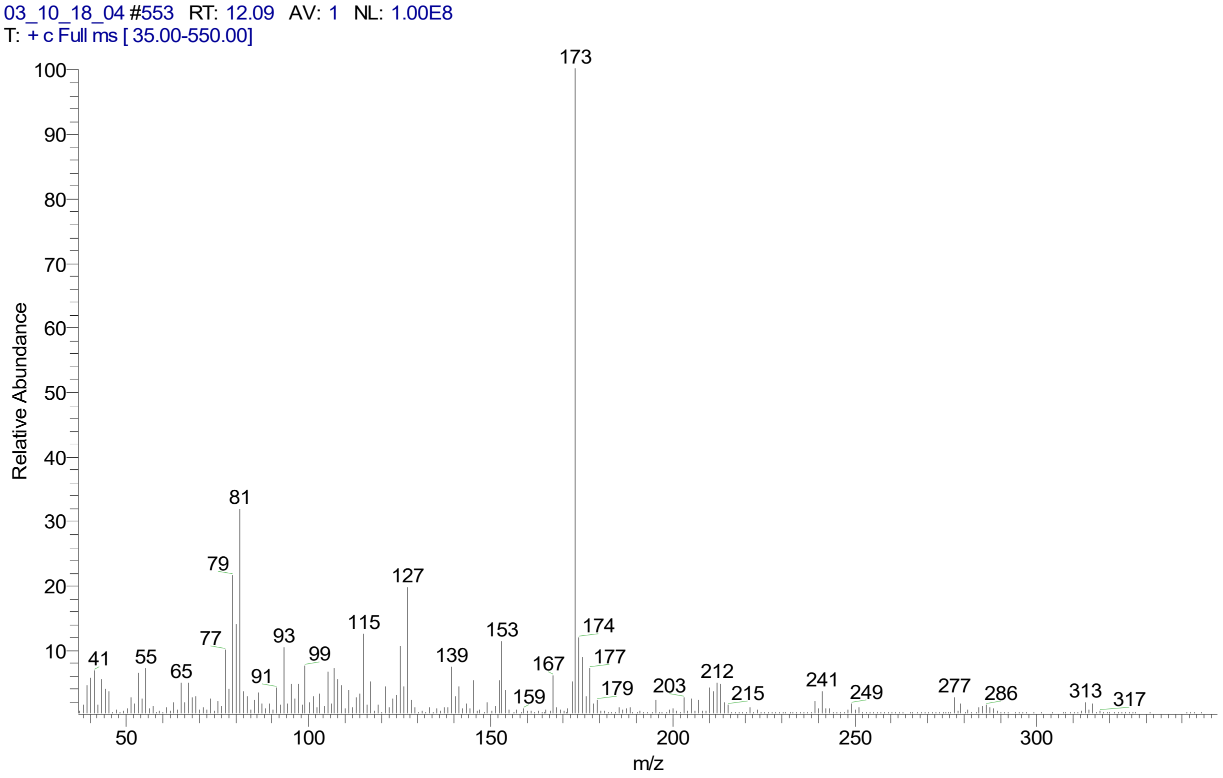


**Entry 32.**

Starting compound – **19**. Catalyst – **11d** (0.1 mol %). The reaction mixture was heated at reflux in $CHCl_3$ for 4 h under an argon atmosphere.

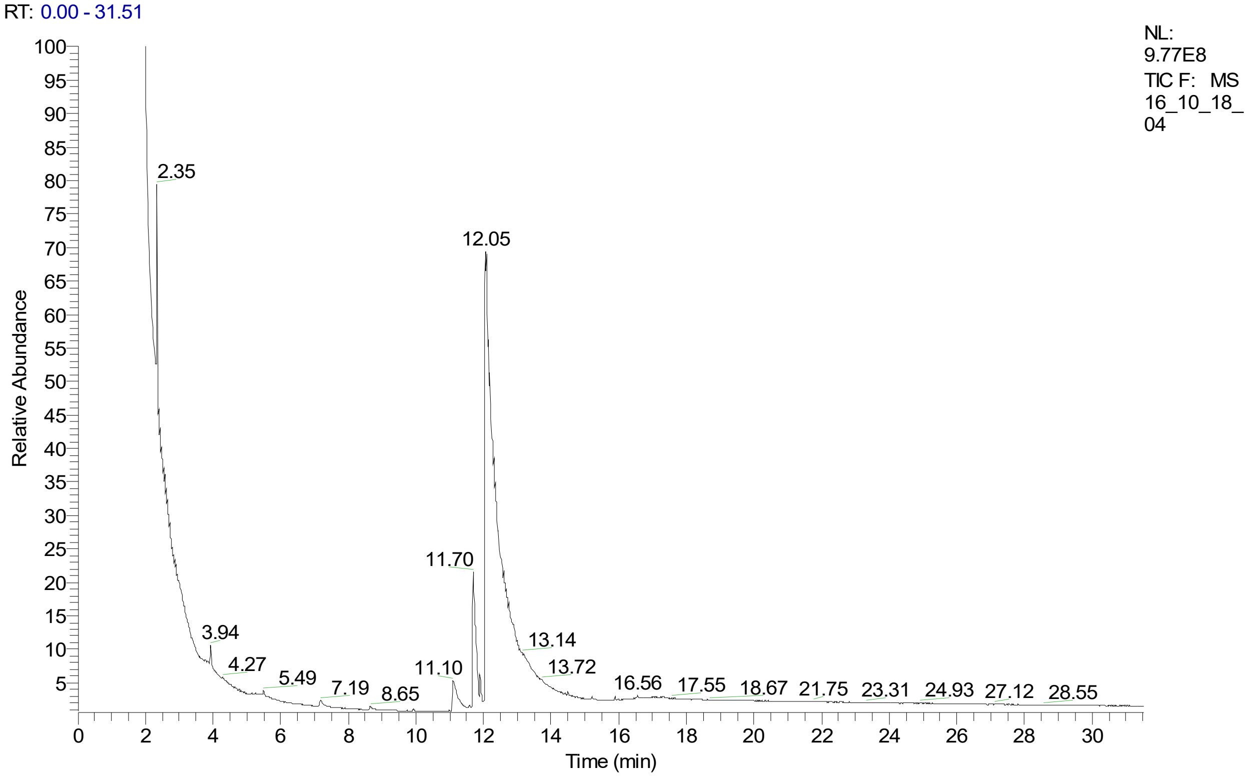

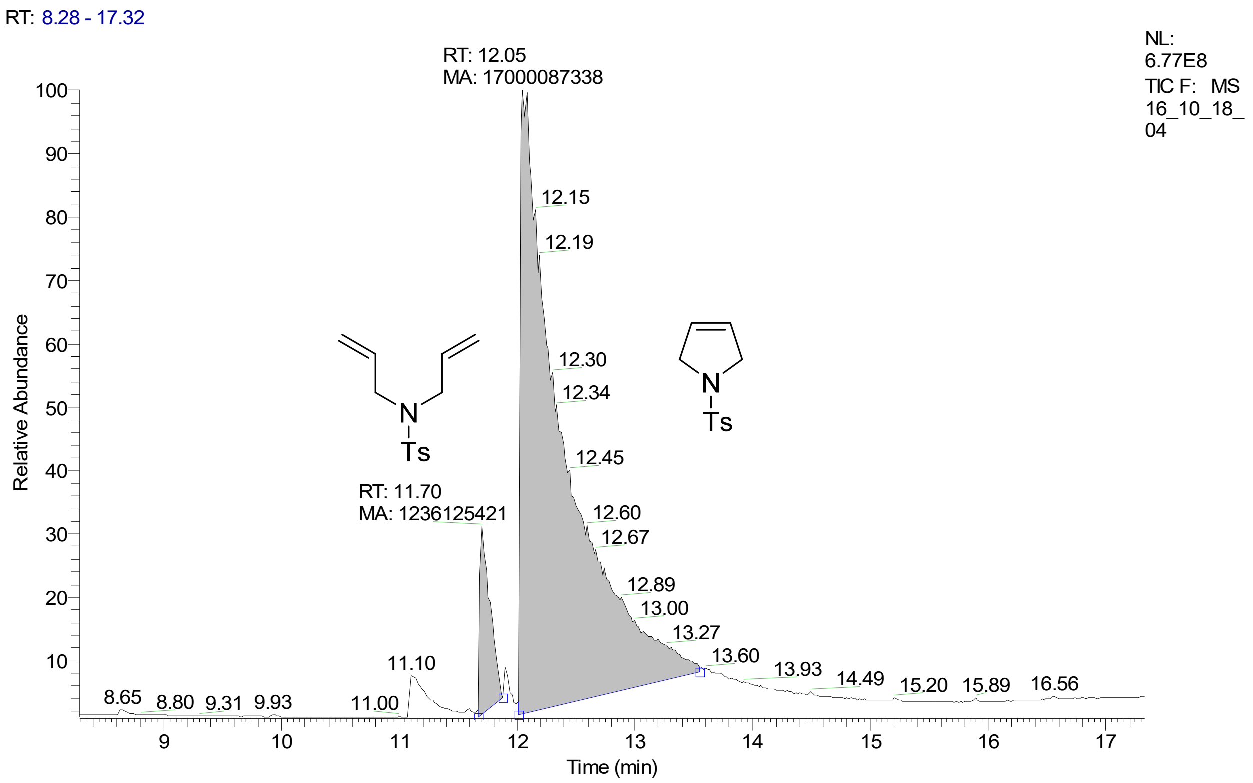
RT: 8.28 - 17.32
NL: 6.77E8
TIC F: MS 16_10_18_04
RT: 12.05
MA: 17000087338
RT: 11.70
MA: 1236125421
N
Ts
Relative Abundance
Time (min)

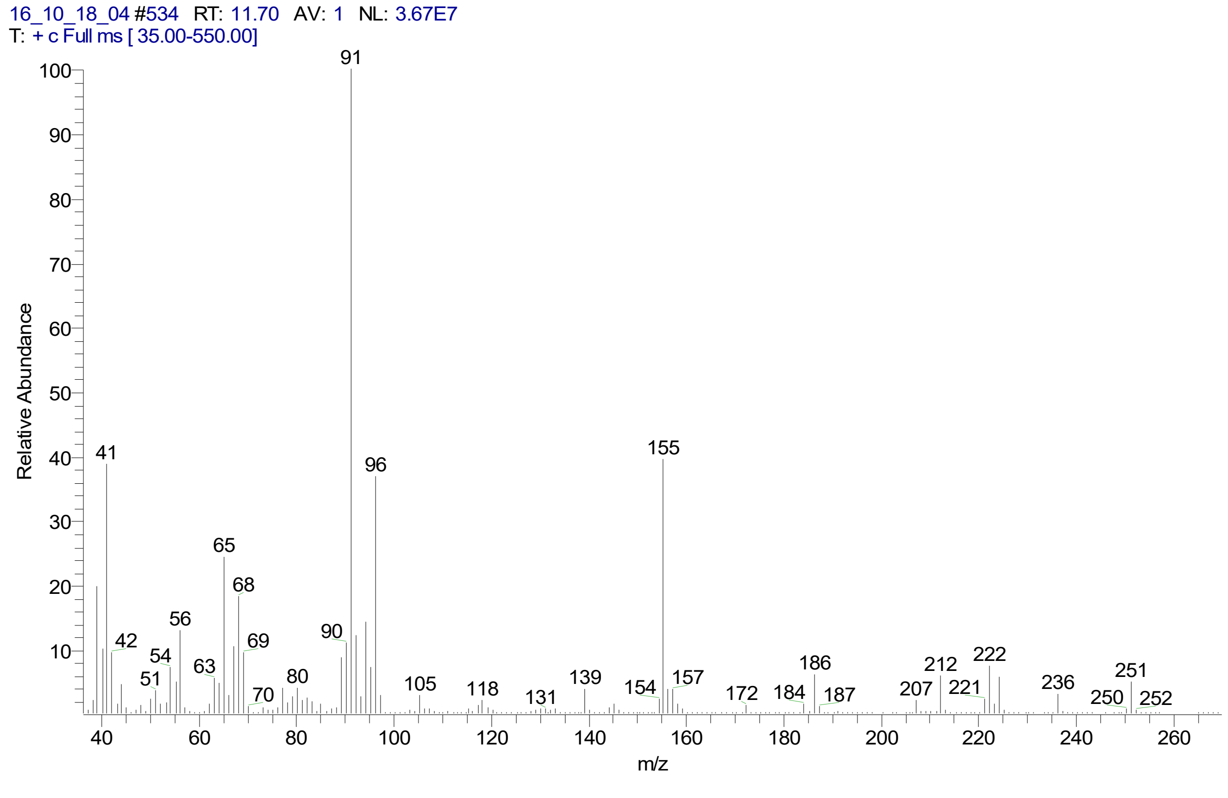
16_10_18_04 #534 RT: 11.70 AV: 1 NL: 3.67E7
T: + c Full ms [ 35.00-550.00]
Relative Abundance
m/z

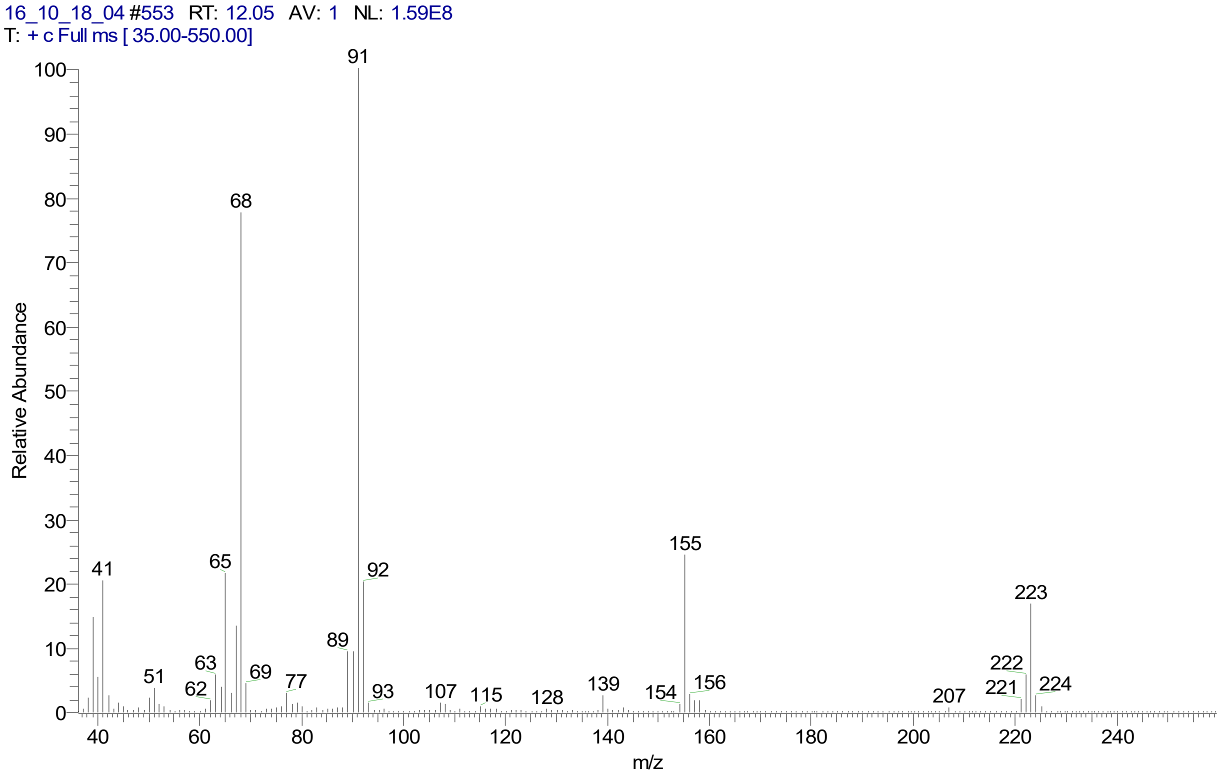


## Entry 33.

Starting compound – **19**. Catalyst – **11d** (0.01 mol %). The reaction mixture was heated at reflux in $CHCl_3$ for 4 h under an argon atmosphere.

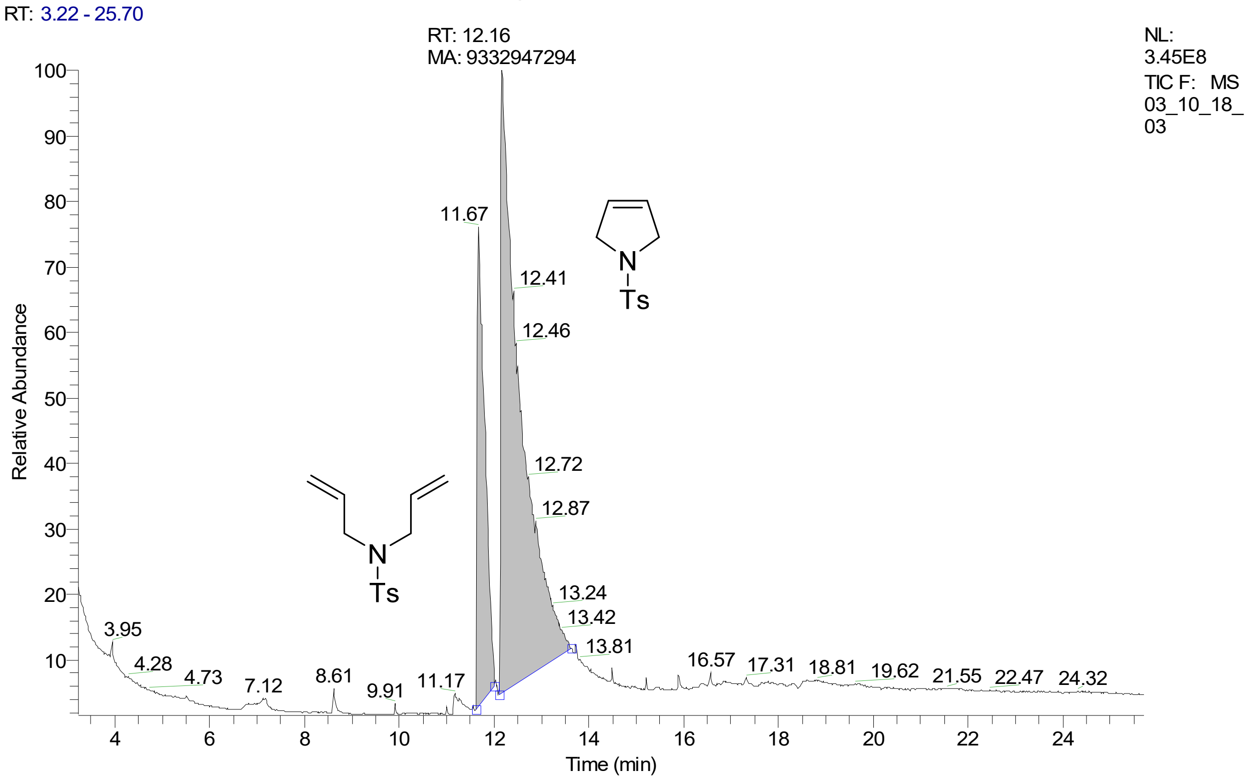

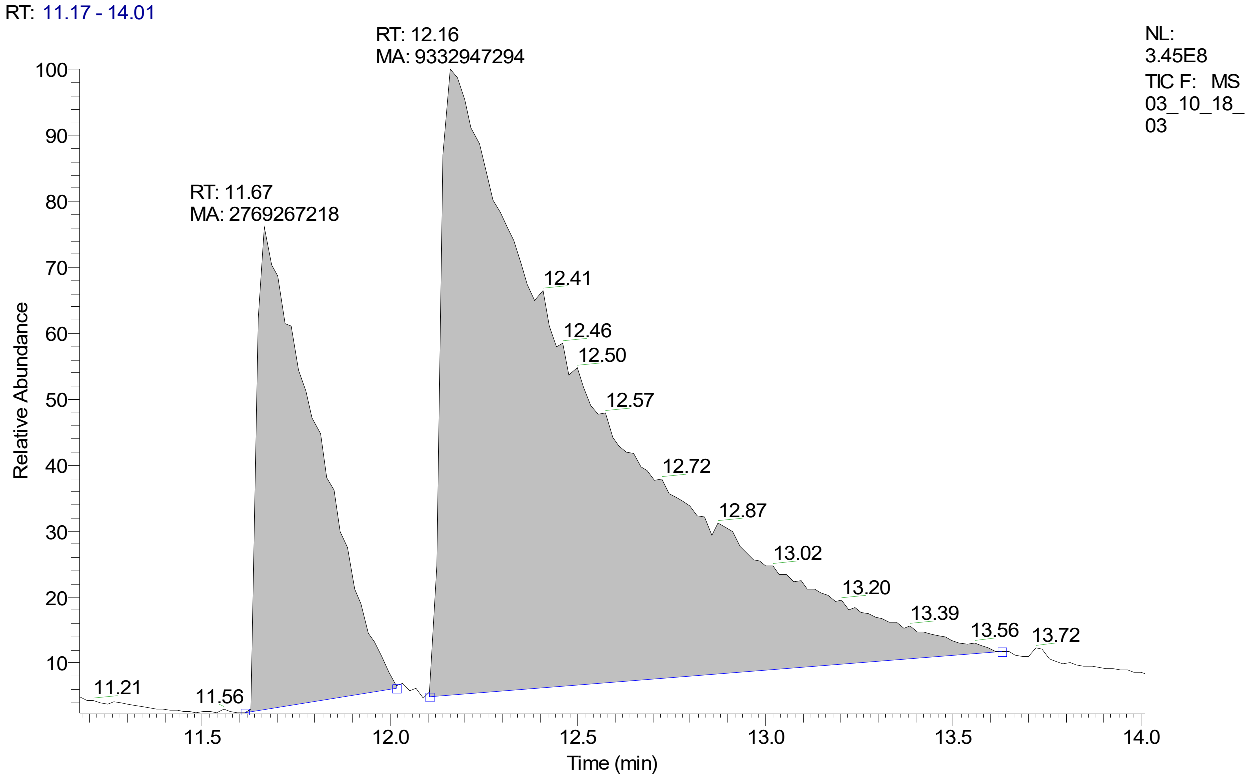

RT: 11.17 - 14.01
RT: 12.16
MA: 9332947294
RT: 11.67
MA: 2769267218
NL:
3.45E8
TIC F: MS
03_10_18_
03
Relative Abundance
Time (min)
11.21
11.56
12.41
12.46
12.50
12.57
12.72
12.87
13.02
13.20
13.39
13.56
13.72


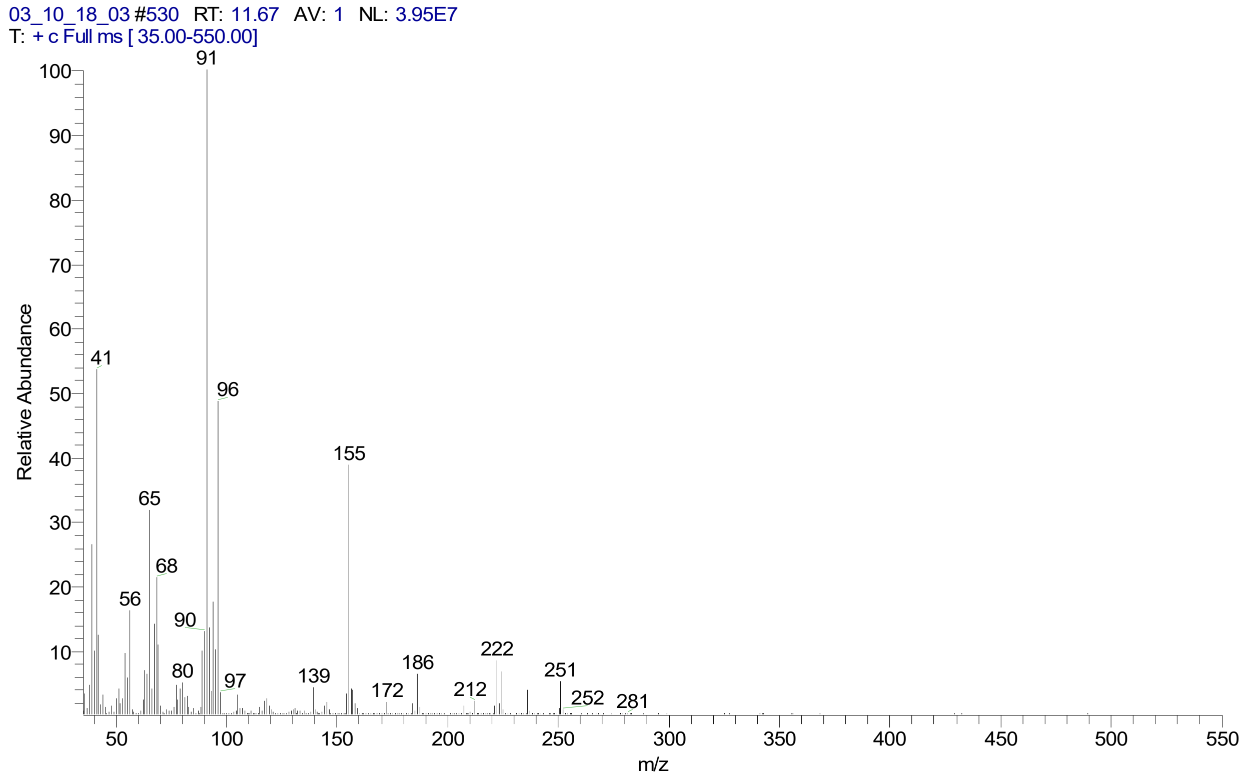

03_10_18_03 #530 RT: 11.67 AV: 1 NL: 3.95E7
T: + c Full ms [ 35.00-550.00]
Relative Abundance
m/z
91
41
96
155
65
68
56
90
80
97
139
172
186
212
222
251
252
281

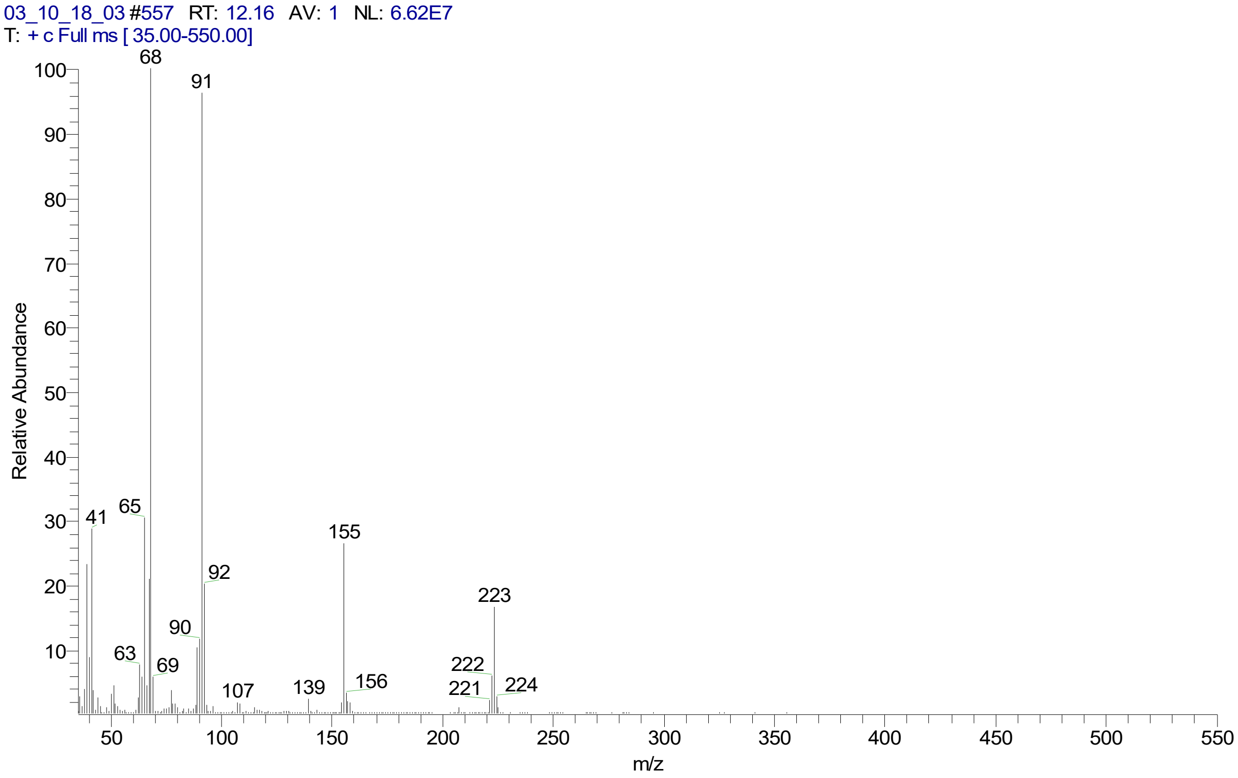


**Entry 34.**
Starting compounds are **21** + 2 eq. **12**. Catalyst is **11a** (0.1 mol %). The reaction mixture was heated at reflux in $CHCl_3$ for 4 h under an argon atmosphere.

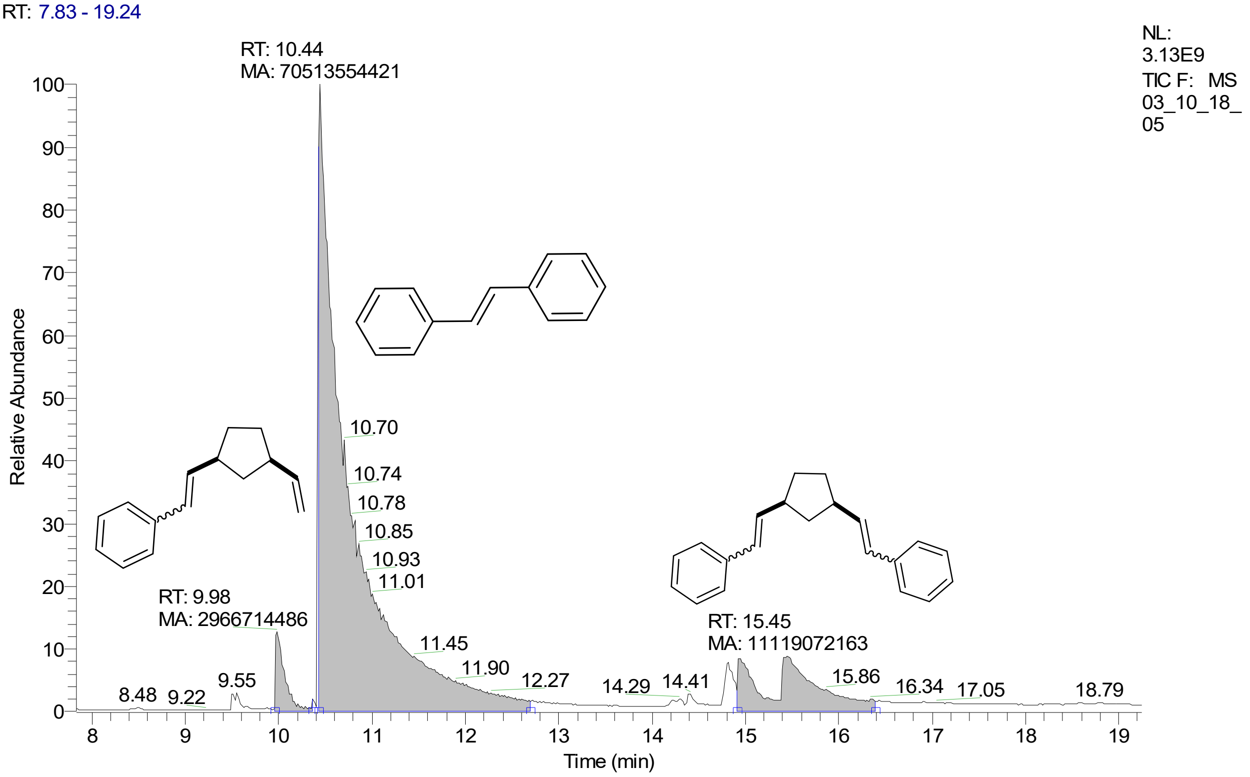

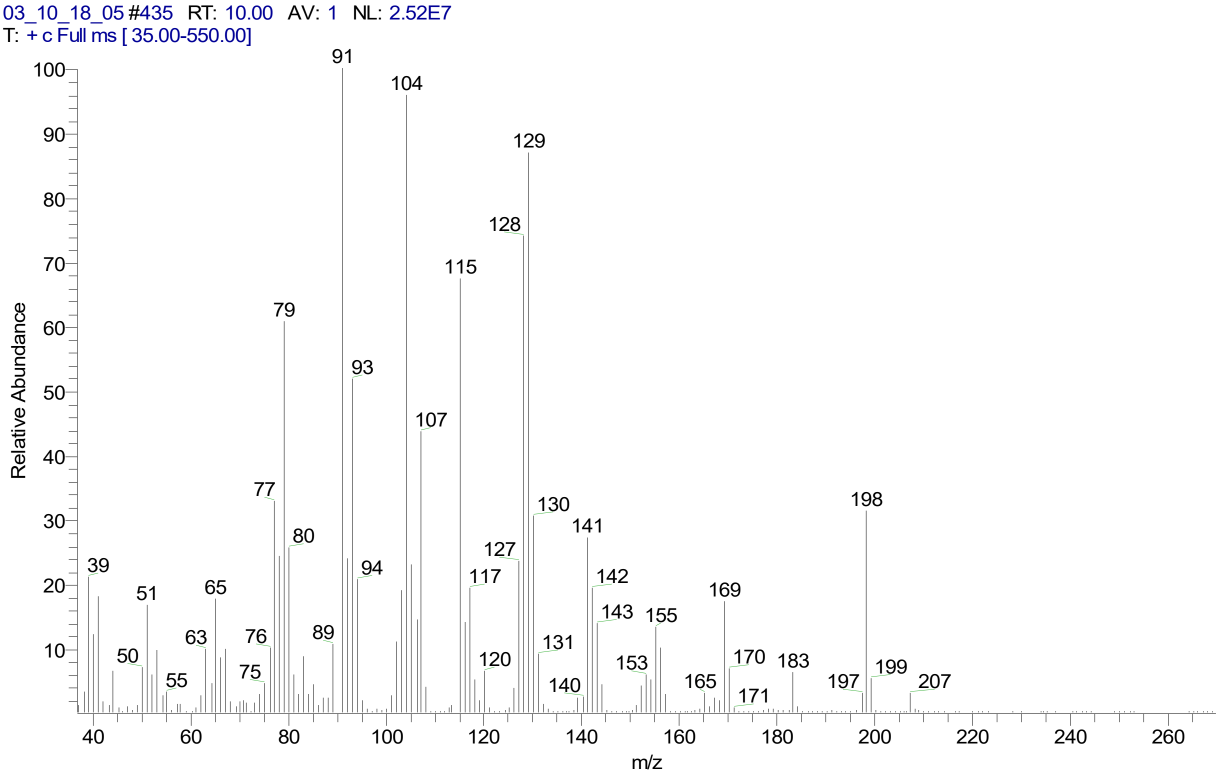
03_10_18_05 #435 RT: 10.00 AV: 1 NL: 2.52E7
T: + c Full ms [ 35.00-550.00]
Relative Abundance
m/z

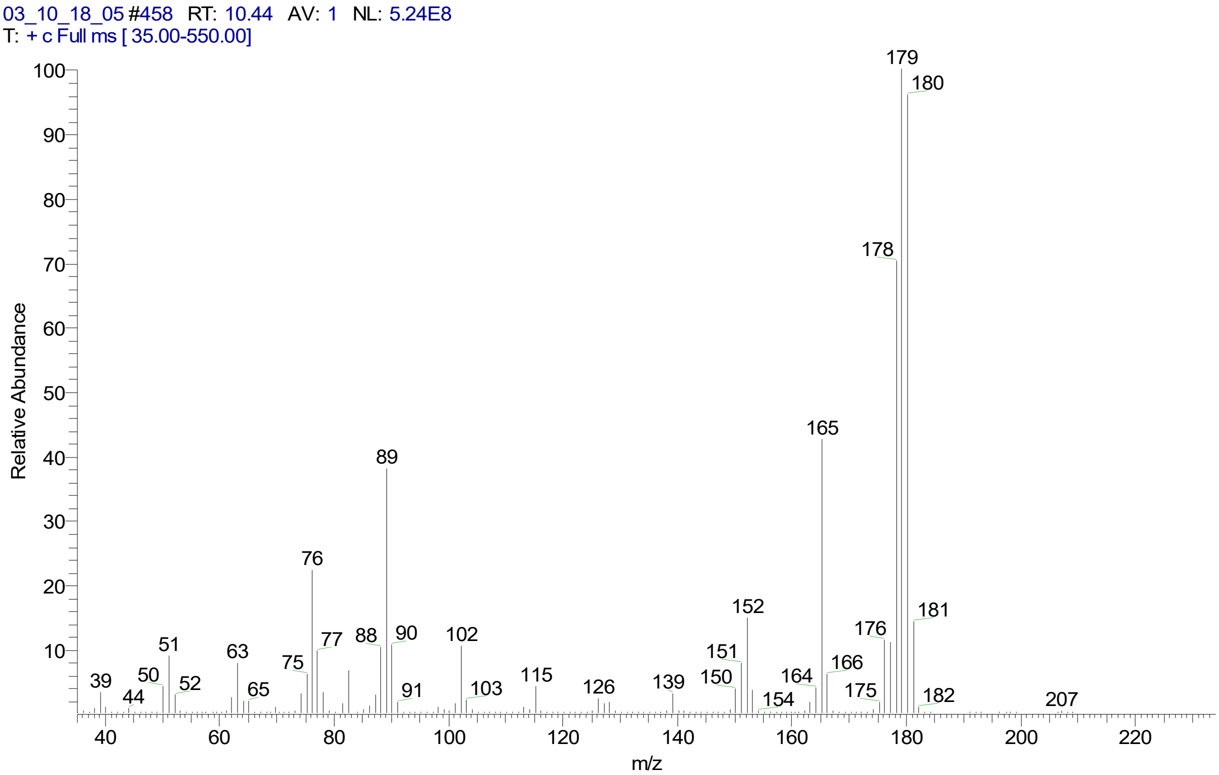
03_10_18_05 #458 RT: 10.44 AV: 1 NL: 5.24E8
T: + c Full ms [ 35.00-550.00]
Relative Abundance
m/z

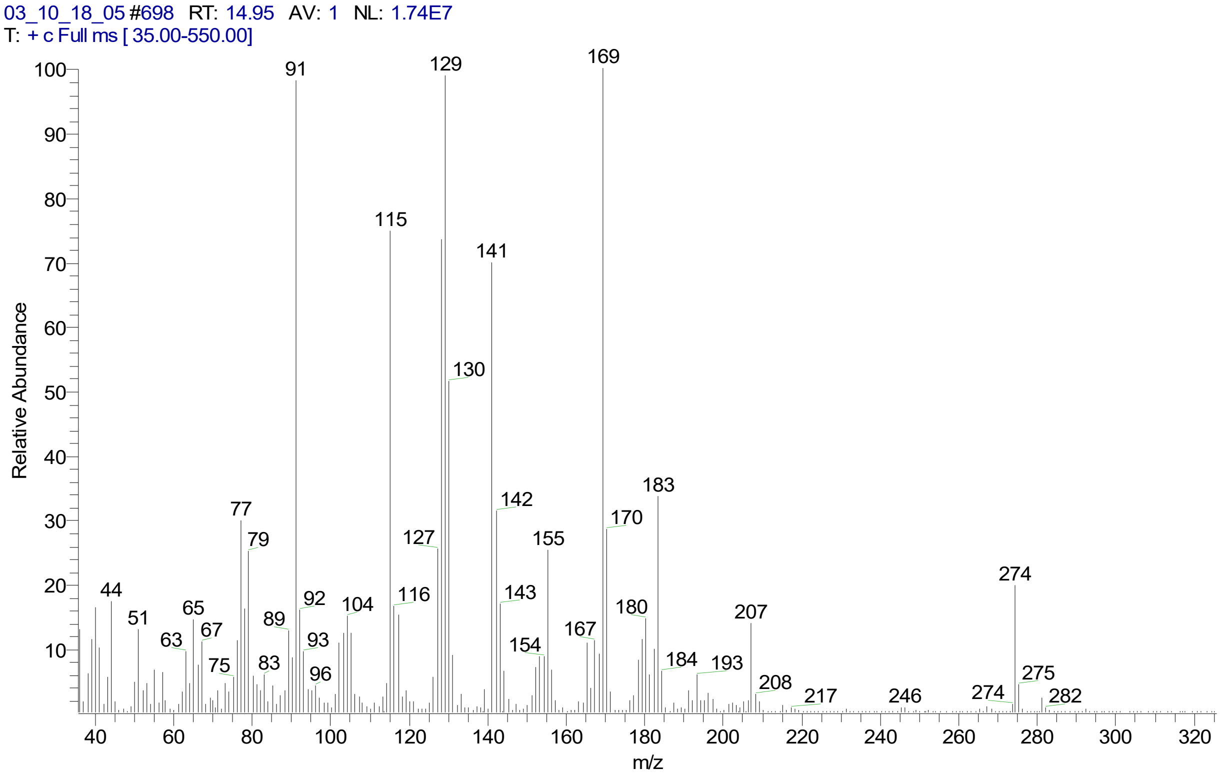
03_10_18_05 #698 RT: 14.95 AV: 1 NL: 1.74E7
T: + c Full ms [ 35.00-550.00]
Relative Abundance
m/z
91
129
169
115
141
130
183
142
170
77
79
127
155
274
44
51
65
63
67
75
83
89
92
93
96
104
116
143
154
167
180
184
193
207
208
217
246
274
275
282

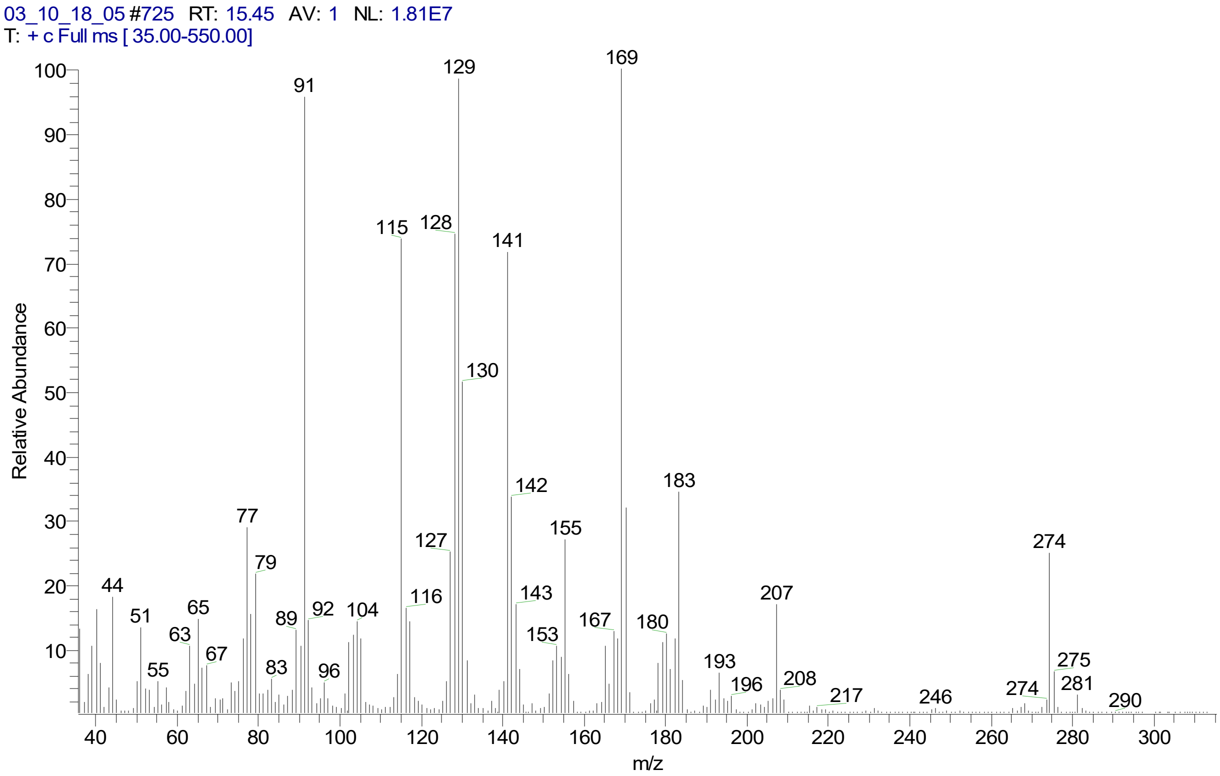
03_10_18_05 #725 RT: 15.45 AV: 1 NL: 1.81E7
T: + c Full ms [ 35.00-550.00]
Relative Abundance
m/z
169
129
91
115
128
141
130
142
183
77
155
274
79
127
44
51
65
63
55
67
83
89
92
96
104
116
143
153
167
180
193
196
207
208
217
246
274
275
281
290

**Entry 35.**

Starting compounds are **21** + 2 eq. **12**. Catalyst is **11d** (0.1 mol %). The reaction mixture was heated at reflux in $CHCl_3$ for 4 h under an argon atmosphere.

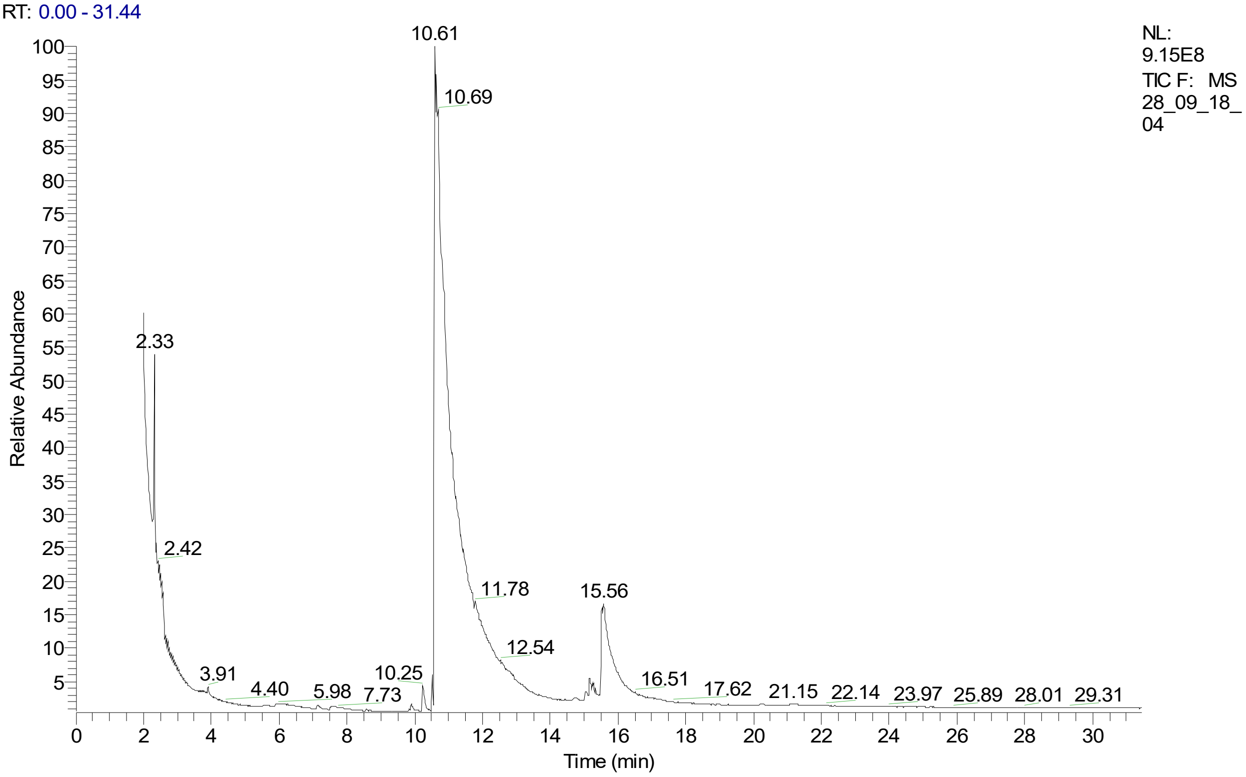


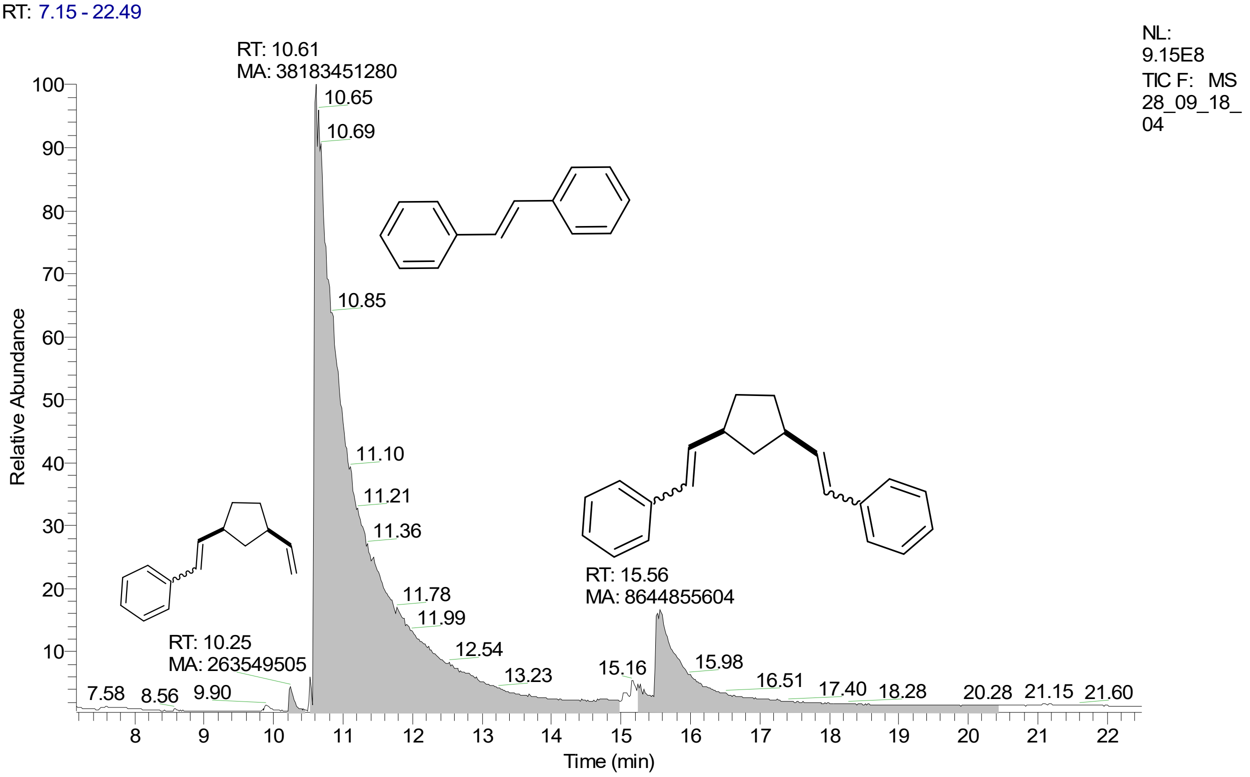

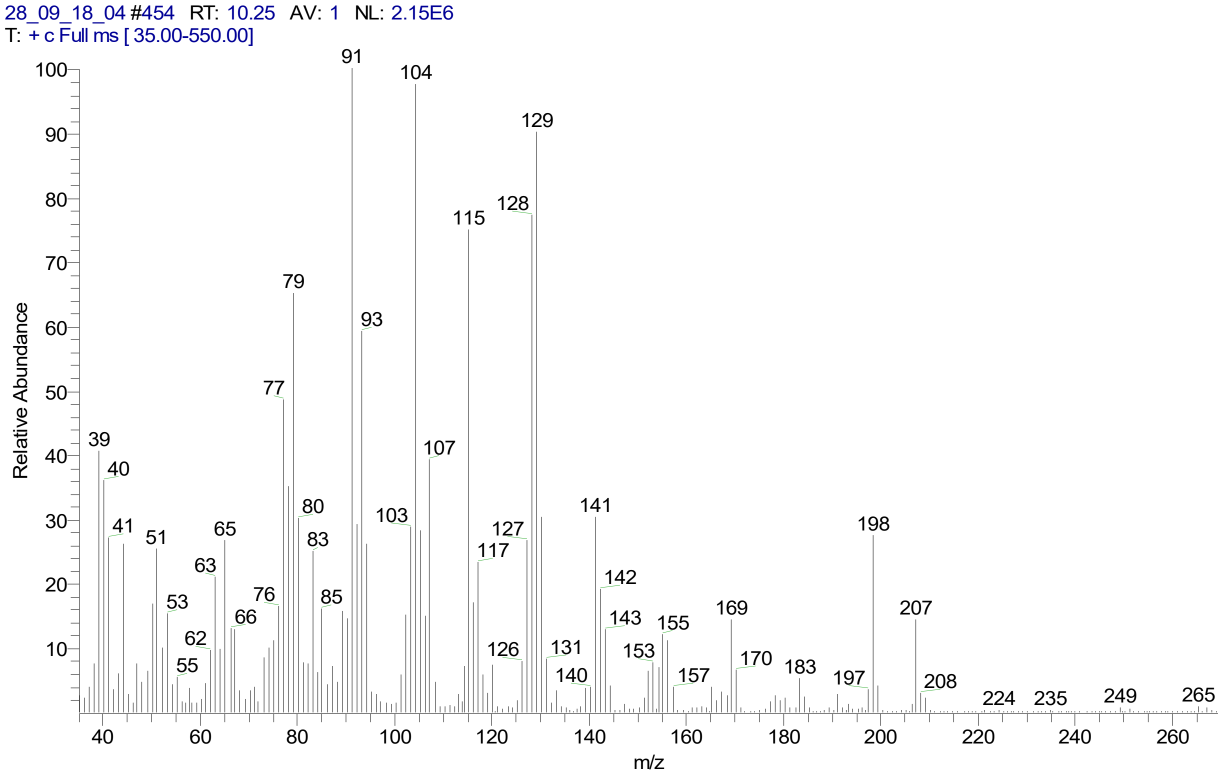
28_09_18_04 #454 RT: 10.25 AV: 1 NL: 2.15E6
T: + c Full ms [ 35.00-550.00]
Relative Abundance
m/z

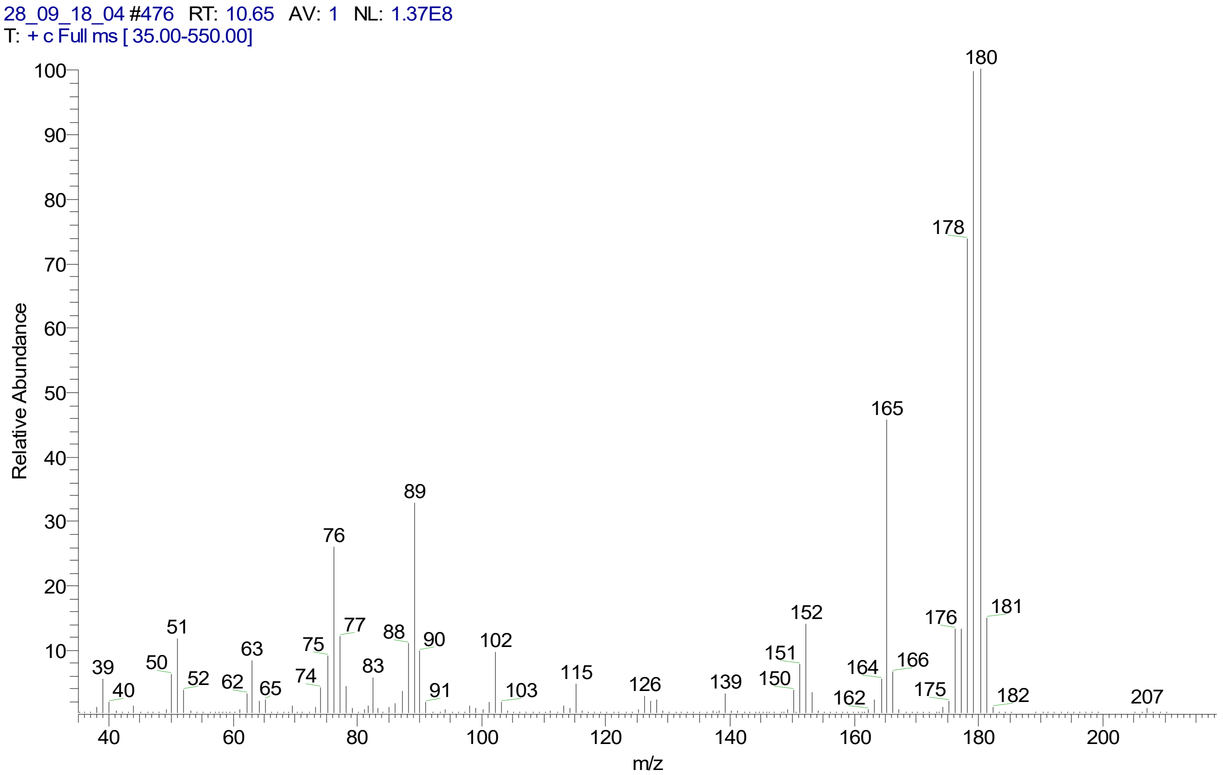
28_09_18_04 #476 RT: 10.65 AV: 1 NL: 1.37E8
T: + c Full ms [ 35.00-550.00]
Relative Abundance
m/z

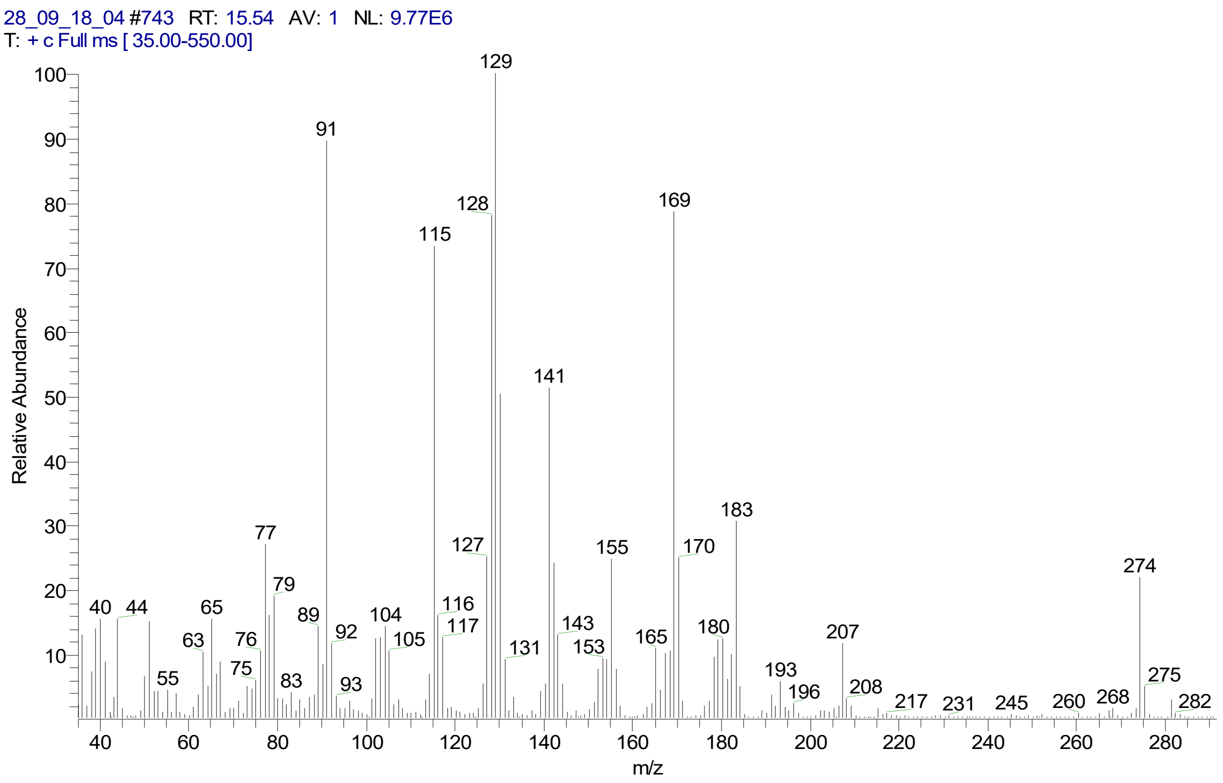


## Entry 36.

Starting compounds are **21** + 2 eq. **24**. Catalyst is **11a** (0.1 mol %). The reaction mixture was heated at reflux in $CHCl_3$ for 4 h under an argon atmosphere.

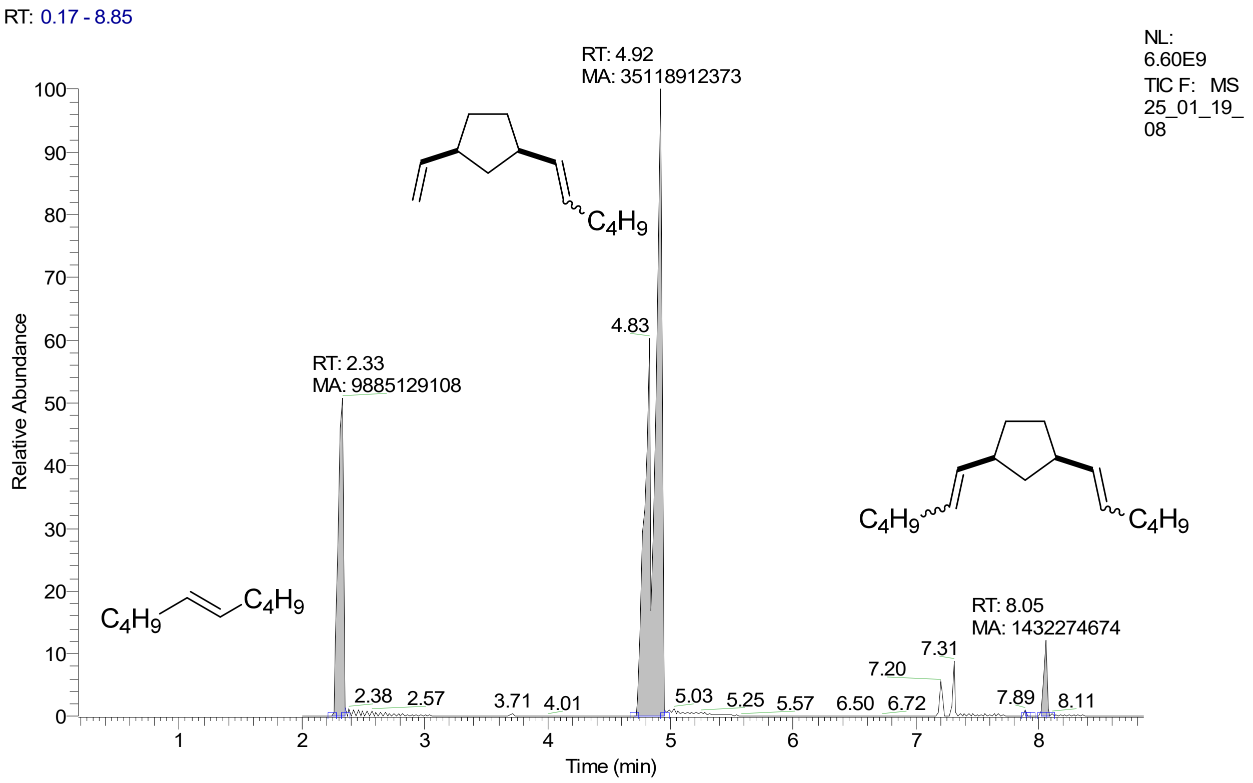

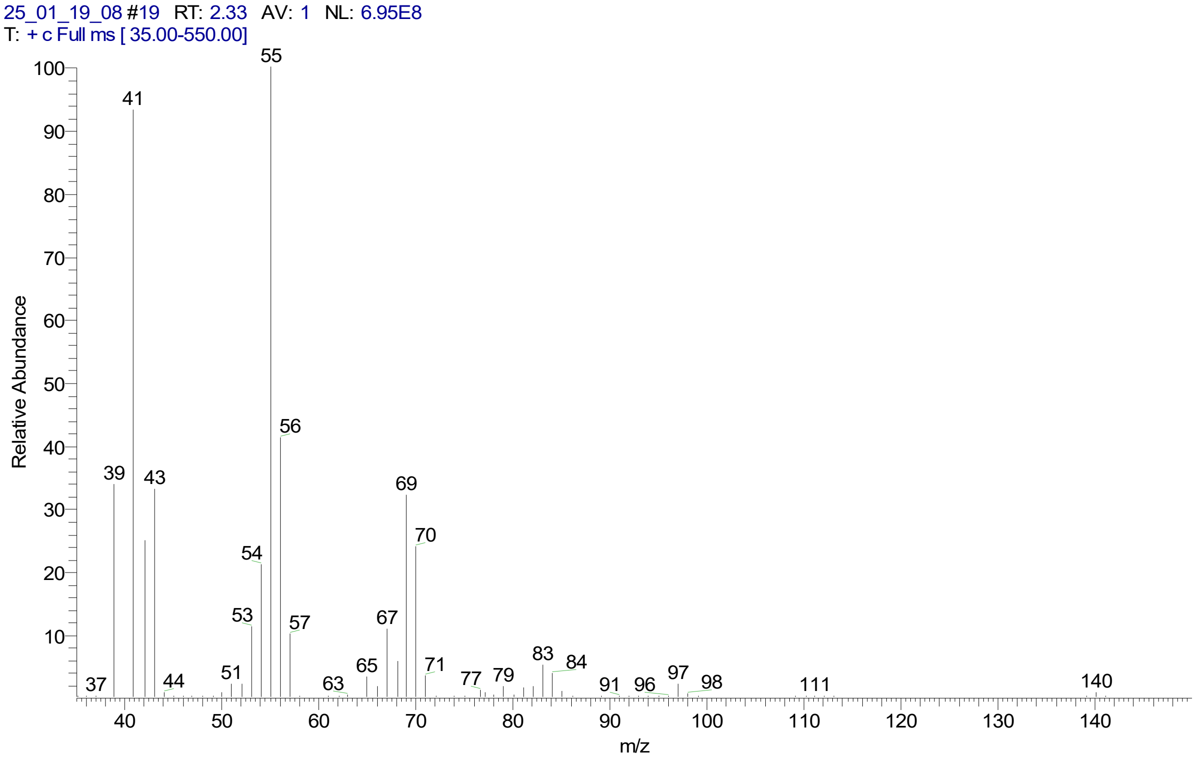
25_01_19_08 #19 RT: 2.33 AV: 1 NL: 6.95E8
T: + c Full ms [ 35.00-550.00]
Relative Abundance
m/z
55
41
56
39
43
69
70
54
53
57
67
83
84
71
65
97
77
79
98
51
44
63
37
91
96
111
140

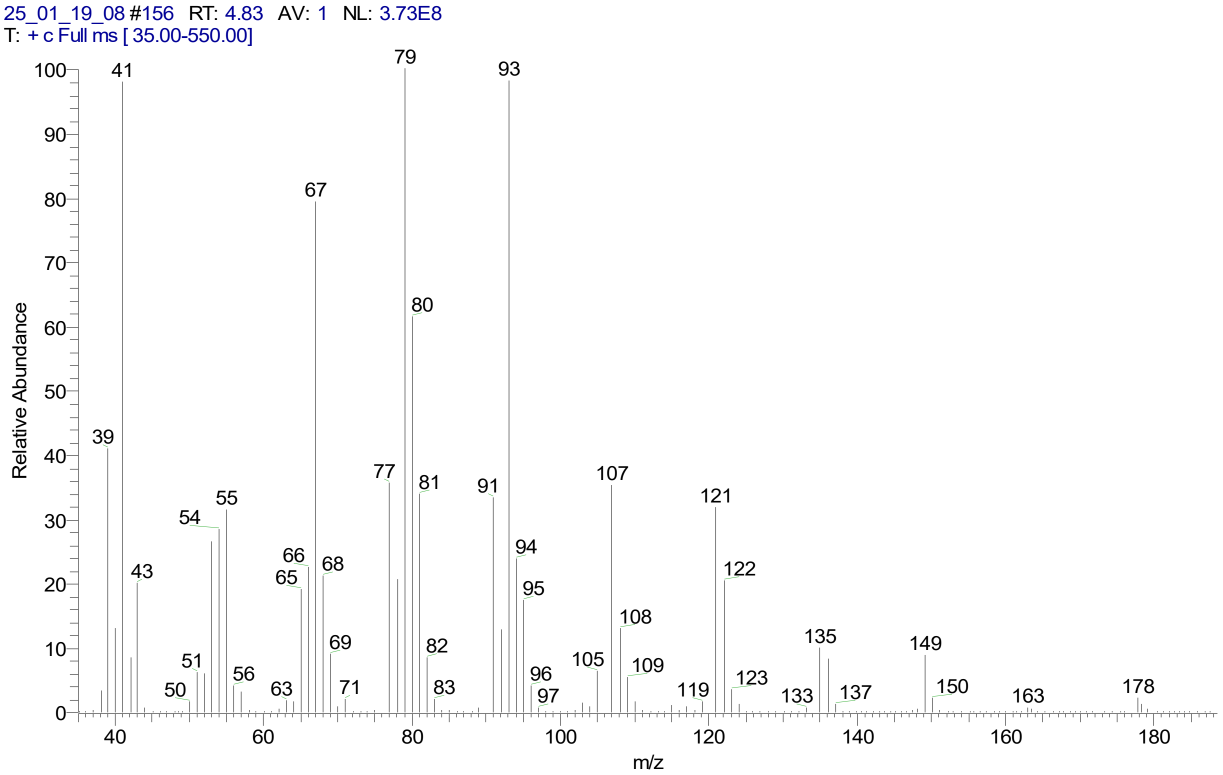
25_01_19_08 #156 RT: 4.83 AV: 1 NL: 3.73E8
T: + c Full ms [ 35.00-550.00]
Relative Abundance
m/z
79
93
41
67
80
39
77
107
81
91
121
55
54
94
66
68
43
65
122
95
108
135
149
69
82
105
51
109
56
96
123
63
71
83
150
178
50
119
97
133
137
163

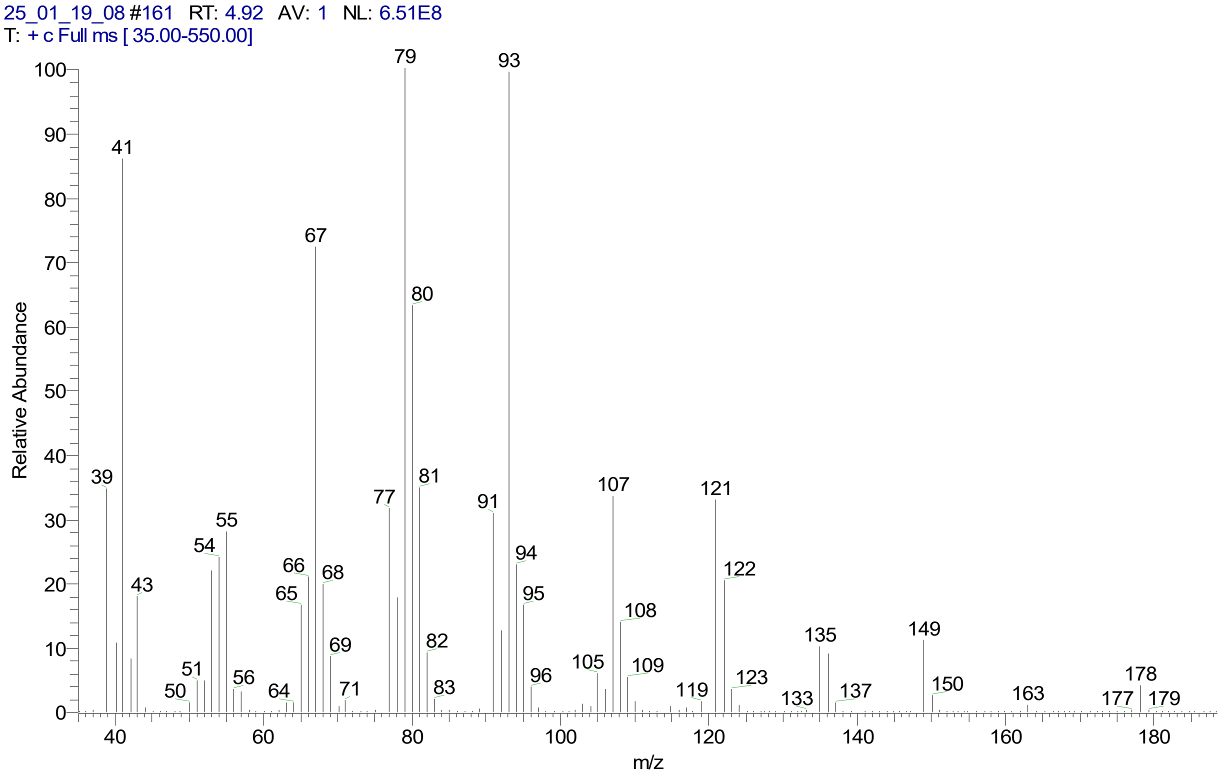
25_01_19_08 #161 RT: 4.92 AV: 1 NL: 6.51E8
T: + c Full ms [ 35.00-550.00]
Relative Abundance
m/z

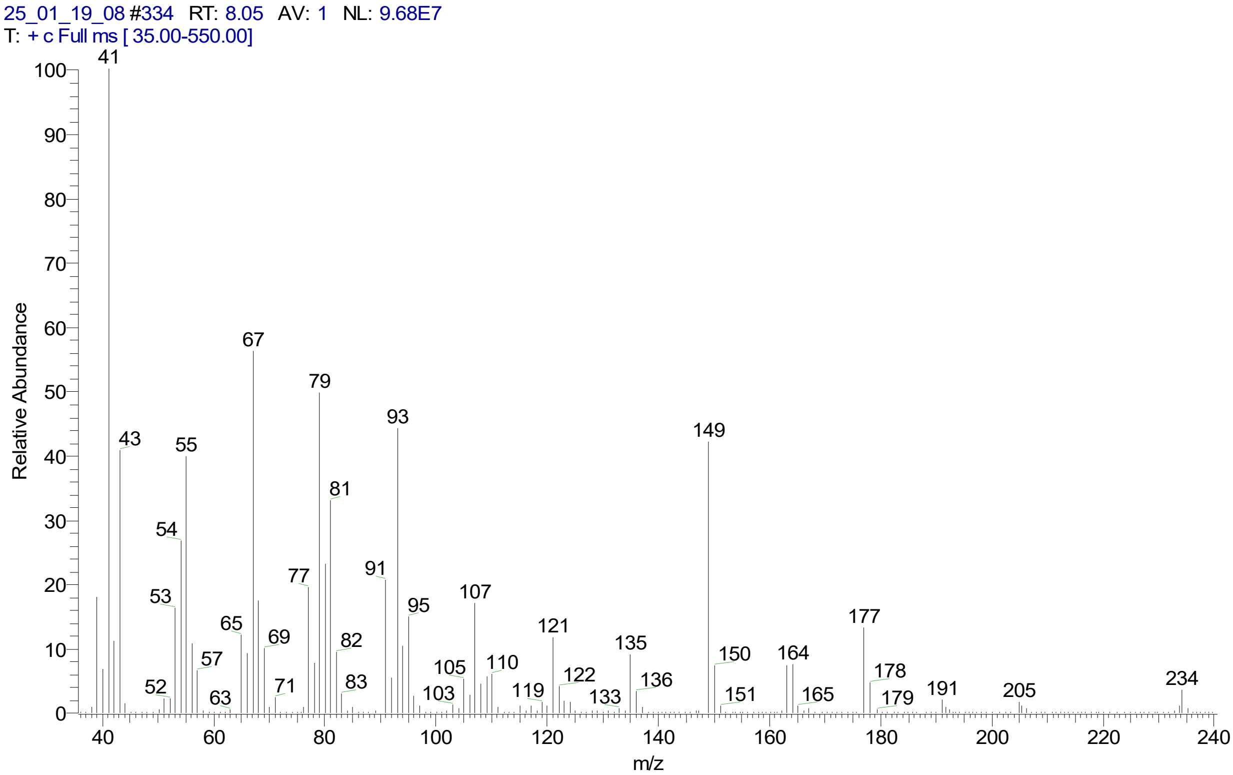
25_01_19_08 #334 RT: 8.05 AV: 1 NL: 9.68E7
T: + c Full ms [ 35.00-550.00]
Relative Abundance
m/z

**Entry 37.**
Starting compounds are **21** + 2 eq. **24**. Catalyst is **11b** (0.1 mol %). The reaction mixture was heated at reflux in $CHCl_3$ for 4 h under an argon atmosphere.

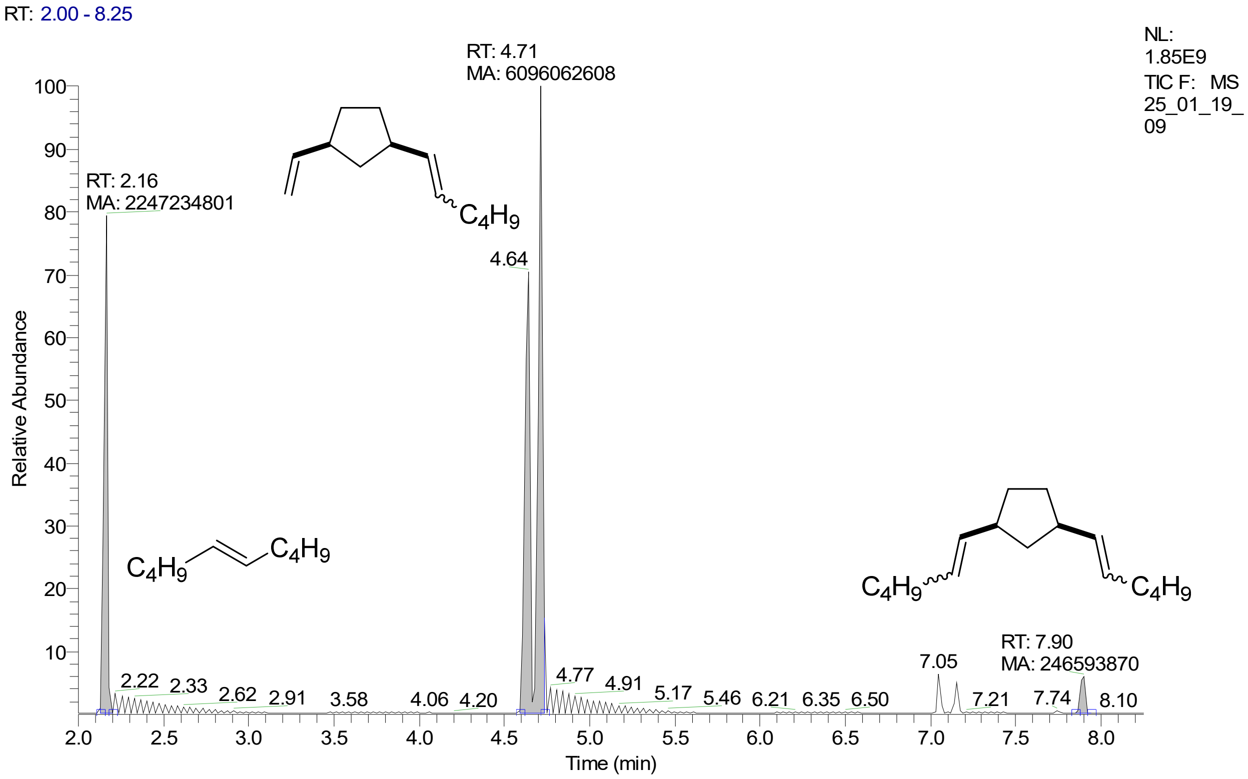


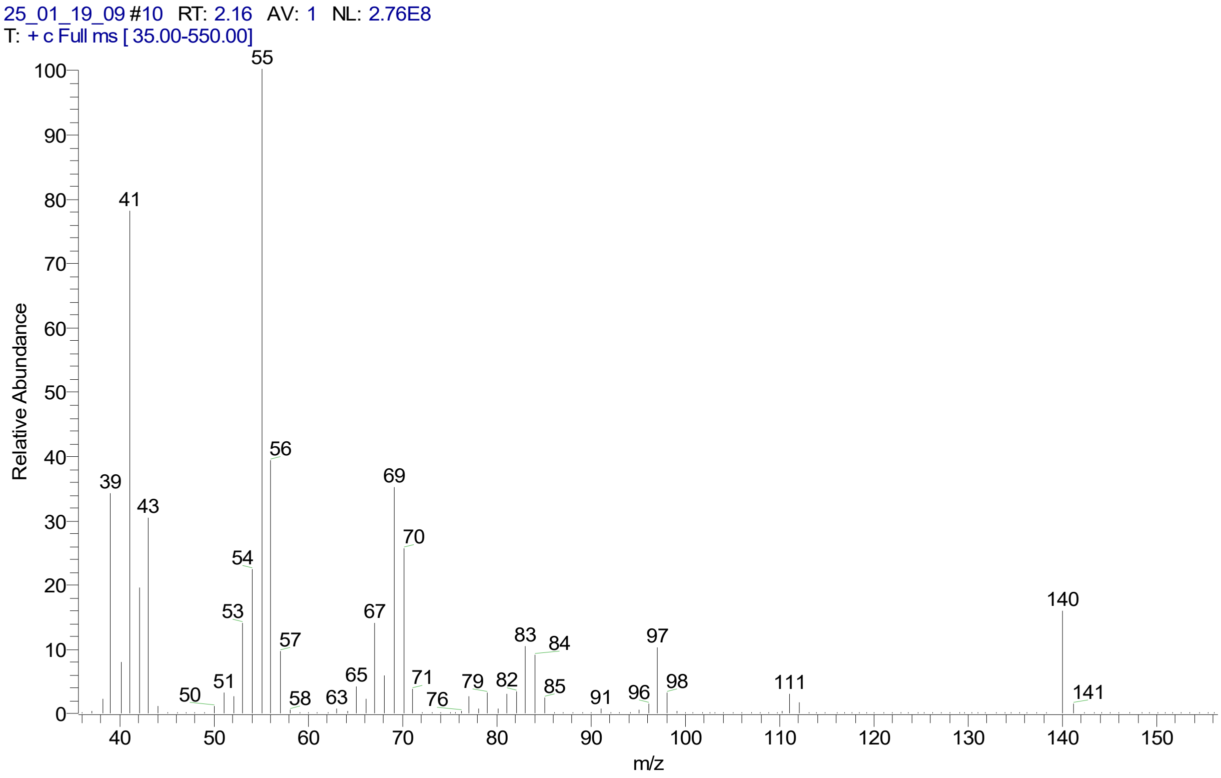

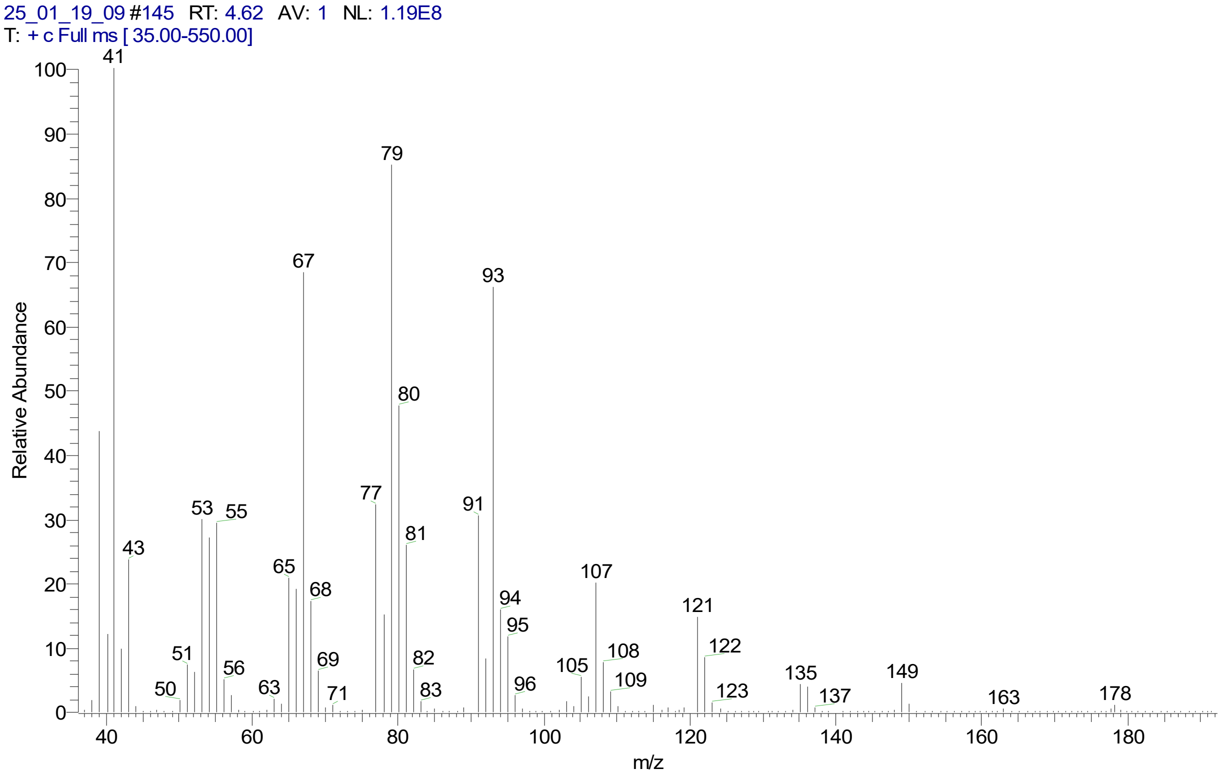
25_01_19_09 #145 RT: 4.62 AV: 1 NL: 1.19E8
T: + c Full ms [ 35.00-550.00]
Relative Abundance
m/z

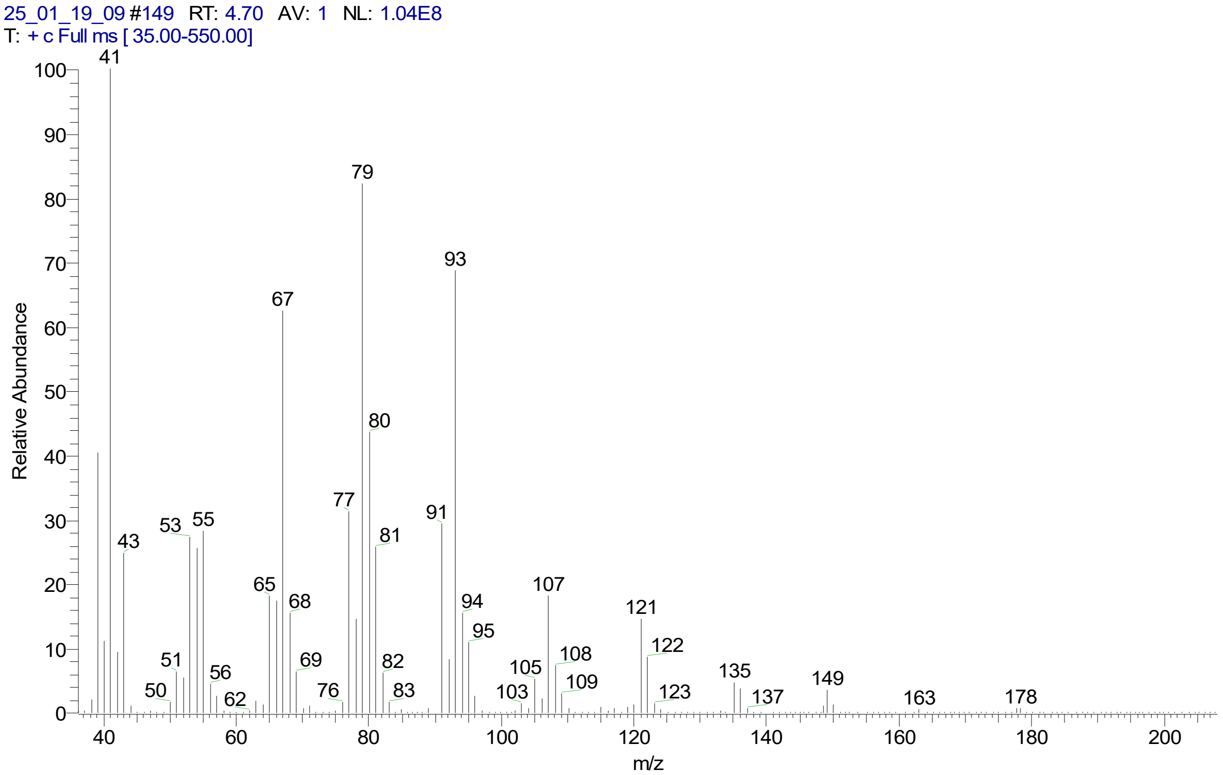
25_01_19_09 #149 RT: 4.70 AV: 1 NL: 1.04E8
T: + c Full ms [ 35.00-550.00]
Relative Abundance
m/z

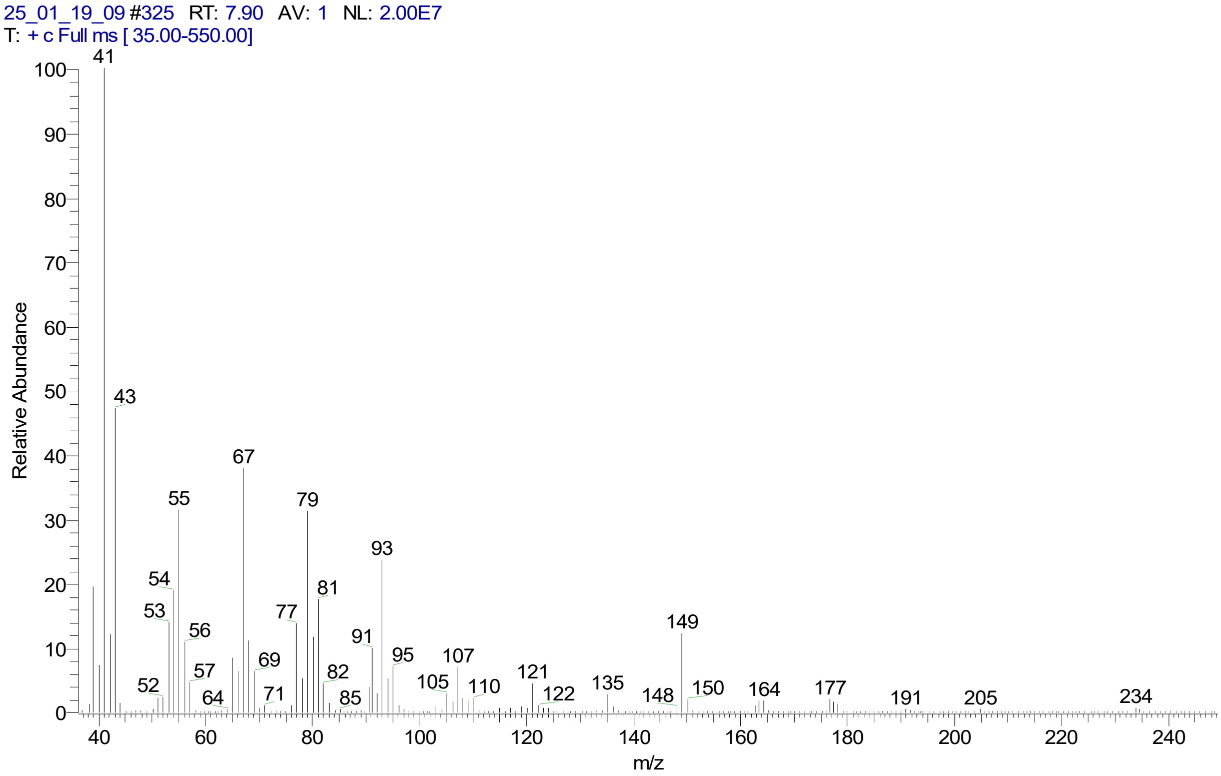

25_01_19_09 #325 RT: 7.90 AV: 1 NL: 2.00E7
T: + c Full ms [ 35.00-550.00]
Relative Abundance
m/z
41
43
55
67
79
93
54
53
81
77
56
91
95
107
69
82
57
52
105
110
121
64
71
85
122
135
149
148
150
164
177
191
205
234

3. Comparison of $^{1}H$ NMR and GC/MS methods for quantitative analysis of mixtures *diallylmalonate (**17**)/ diethyl cyclopent-3-ene-1,1-dicarboxylate (**18**), and diallyltosylamide (**19**)/ 1-tosyl-2,5-dihydro-1H-pyrrole (**20**)* obtained in the course of metathesis reactions.

GC-MS analysis of a mixture of *diethyl diallylmalonate* (**17**)/ *diethyl cyclopent-3-ene-1,1-dicarboxylate* (**18**), the observed ratio is 44/56.

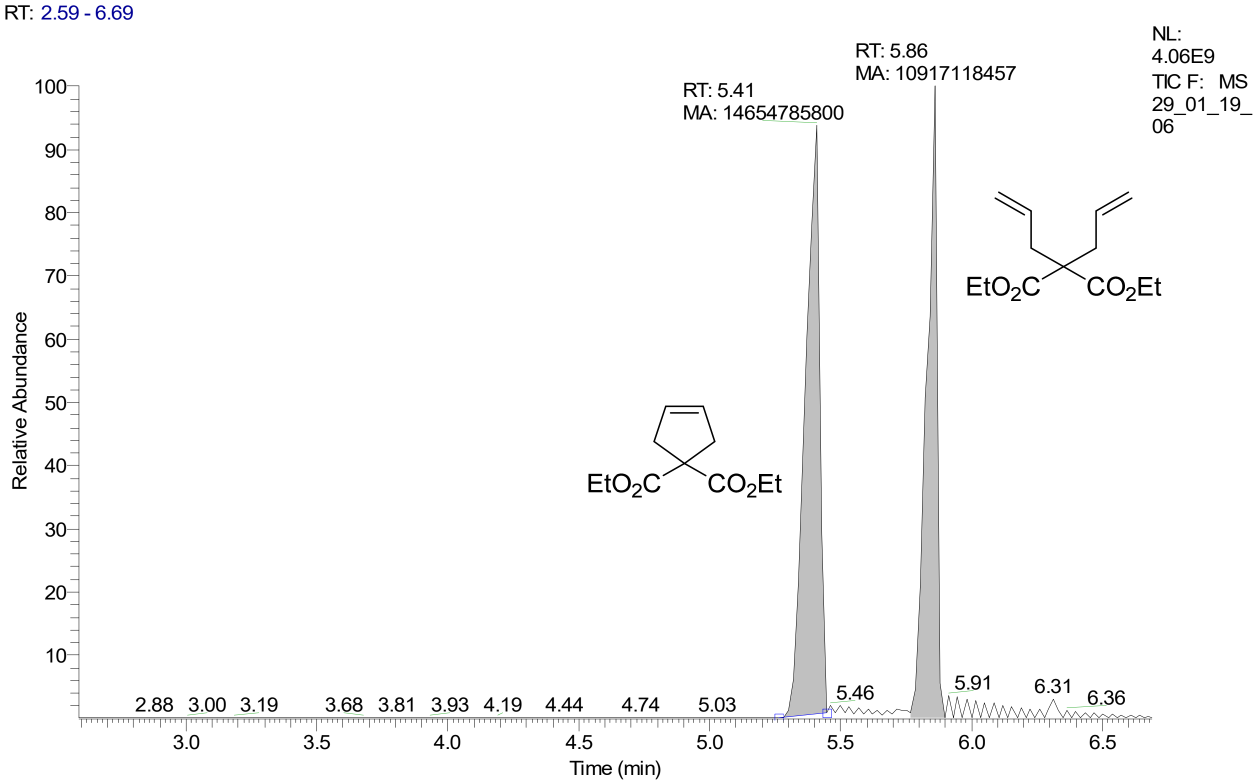


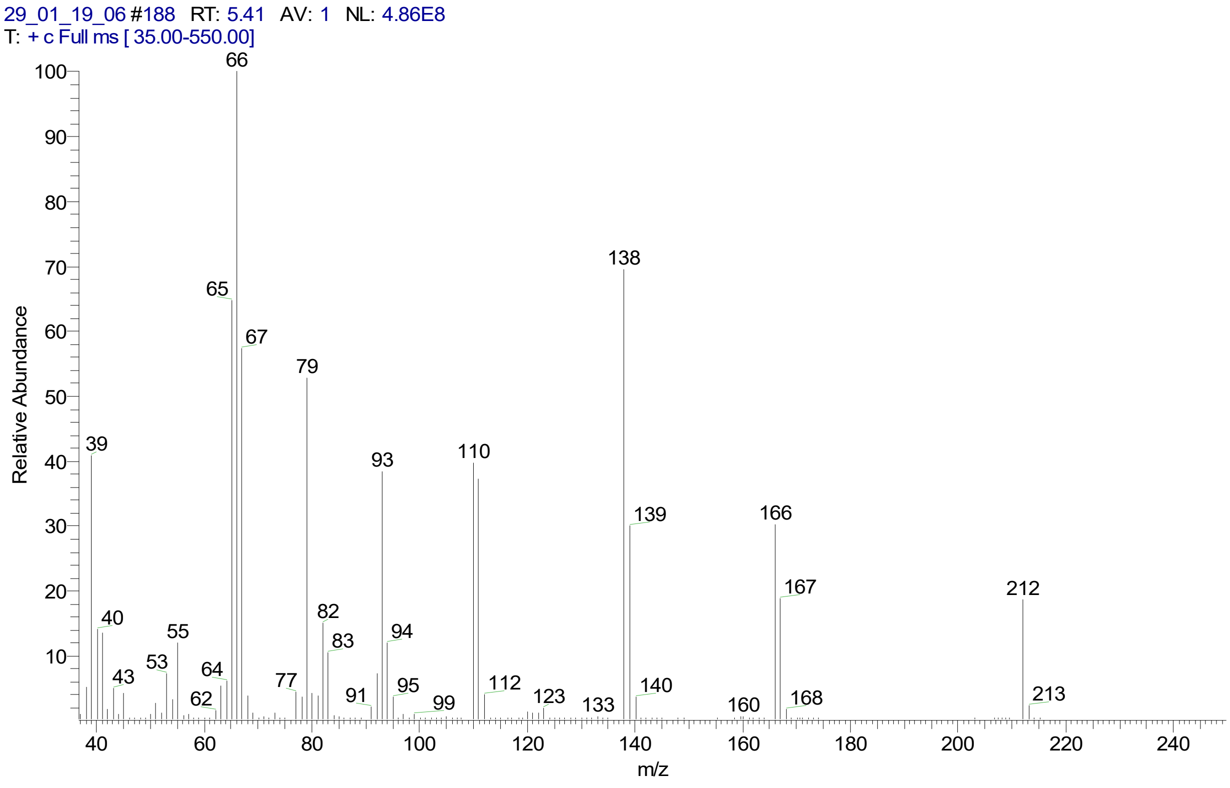

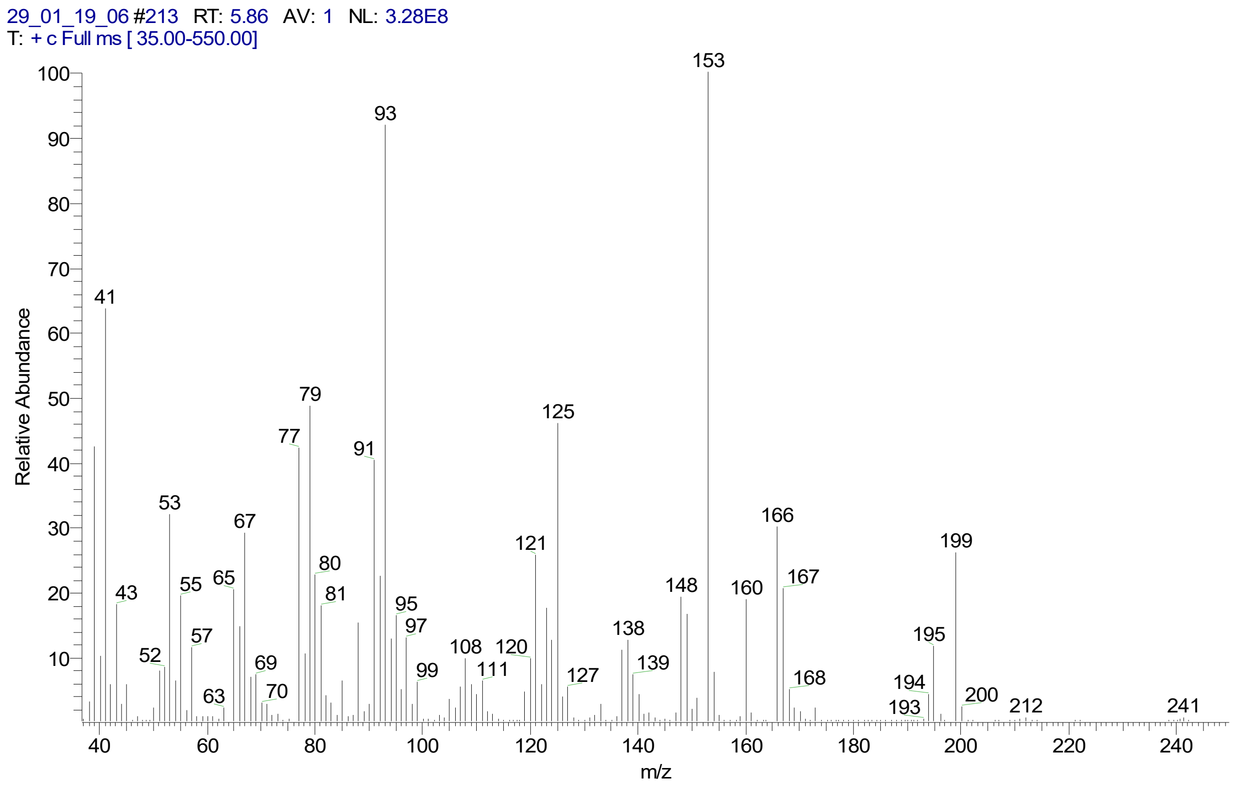


$^{1}$H NMR analysis of the same mixture of diethyl diallylmalonate (**17**)/ diethyl cyclopent-3-ene-1,1-dicarboxylate (**18**), the observed ratio is 47/53.

| | | | | | | | |
|---|---|---|---|---|---|---|---|
| **Acquisition Time (sec)** | 1.9818 | **Comment** | single_pulse | **Date** | 26 Feb 1990 12:08:37 | | |
| **Date Stamp** | 29 Jan 2019 07:51:37 | | | **File Name** | C:\Users\Fedor\Desktop\28.01.19\FZ7125-1.jdf | **Frequency (MHz)** | 600.17 |
| **Nucleus** | 1H | **Number of Transients** | 8 | **Origin** | ECA 600 | **Original Points Count** | 32768 |
| **Owner** | delta | | | | | | |
| **Points Count** | 32768 | **Pulse Sequence** | single_pulse.ex2 | **Receiver Gain** | 26.00 | **Solvent** | CHLOROFORM-d |
| **Spectrum Offset (Hz)** | 5429.8022 | **Sweep Width (Hz)** | 16534.39 | **Temperature (degree C)** | 32.300 | | |

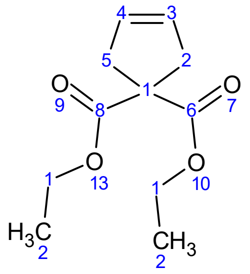

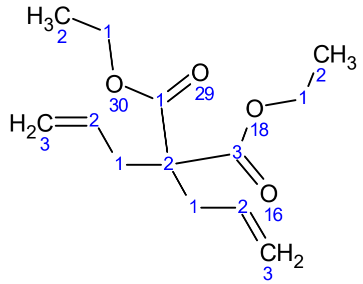

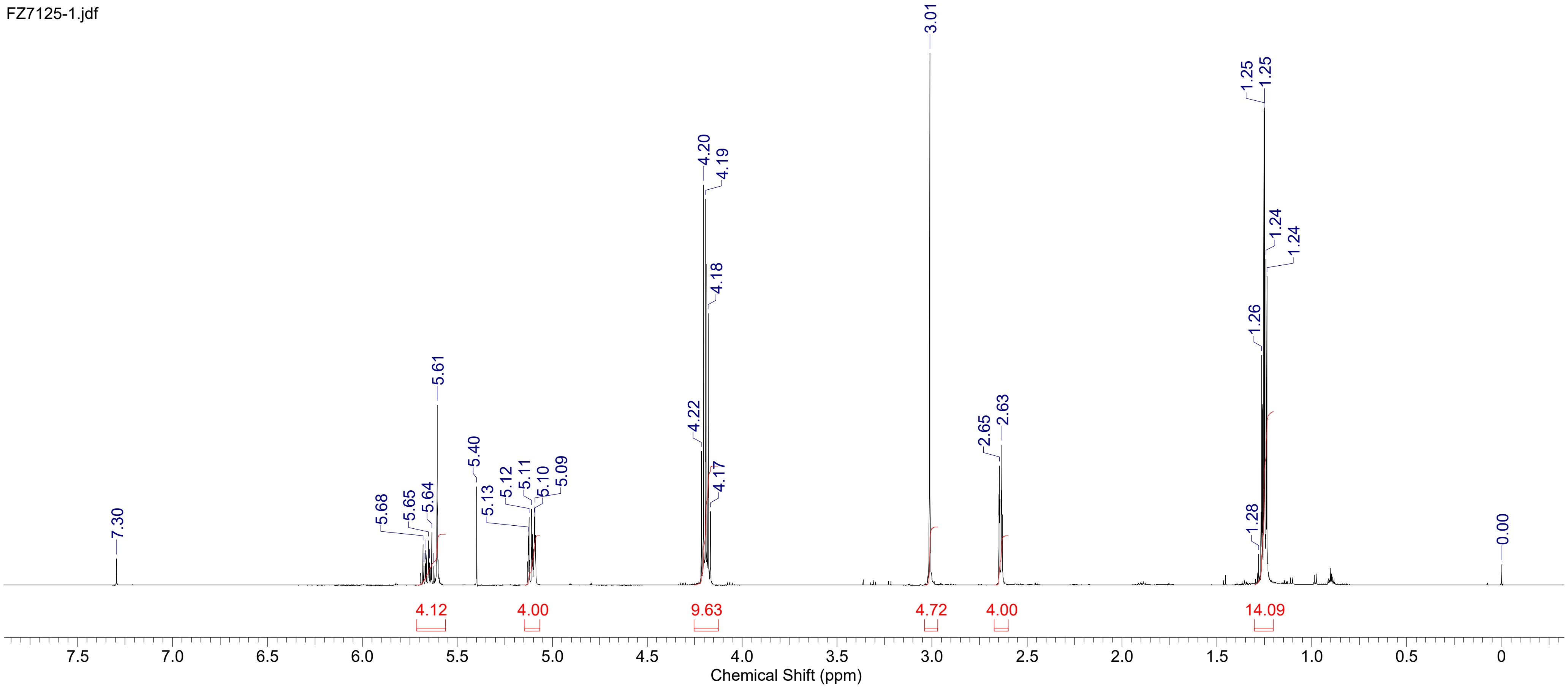

| | | | | | | | |
|---|---|---|---|---|---|---|---|
| **Acquisition Time (sec)** | 1.9818 | **Comment** | single_pulse | **Date** | 26 Feb 1990 12:08:37 | | |
| **Date Stamp** | 29 Jan 2019 07:51:37 | | | **File Name** | C:\Users\Fedor\Desktop\28.01.19\FZ7125-1.jdf | **Frequency (MHz)** | 600.17 |
| **Nucleus** | 1H | **Number of Transients** | 8 | **Origin** | ECA 600 | **Original Points Count** 32768 | **Owner** delta |
| **Points Count** | 32768 | **Pulse Sequence** | single_pulse.ex2 | | | **Receiver Gain** 26.00 | **Solvent** CHLOROFORM-d |
| **Spectrum Offset (Hz)** | 5429.8022 | **Sweep Width (Hz)** | 16534.39 | **Temperature (degree C)** | 32.300 | | |

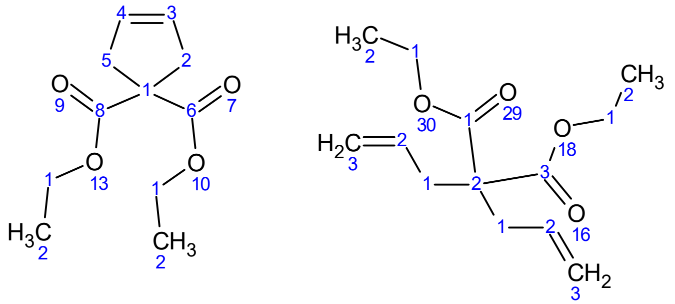

FZ7125-1.jdf

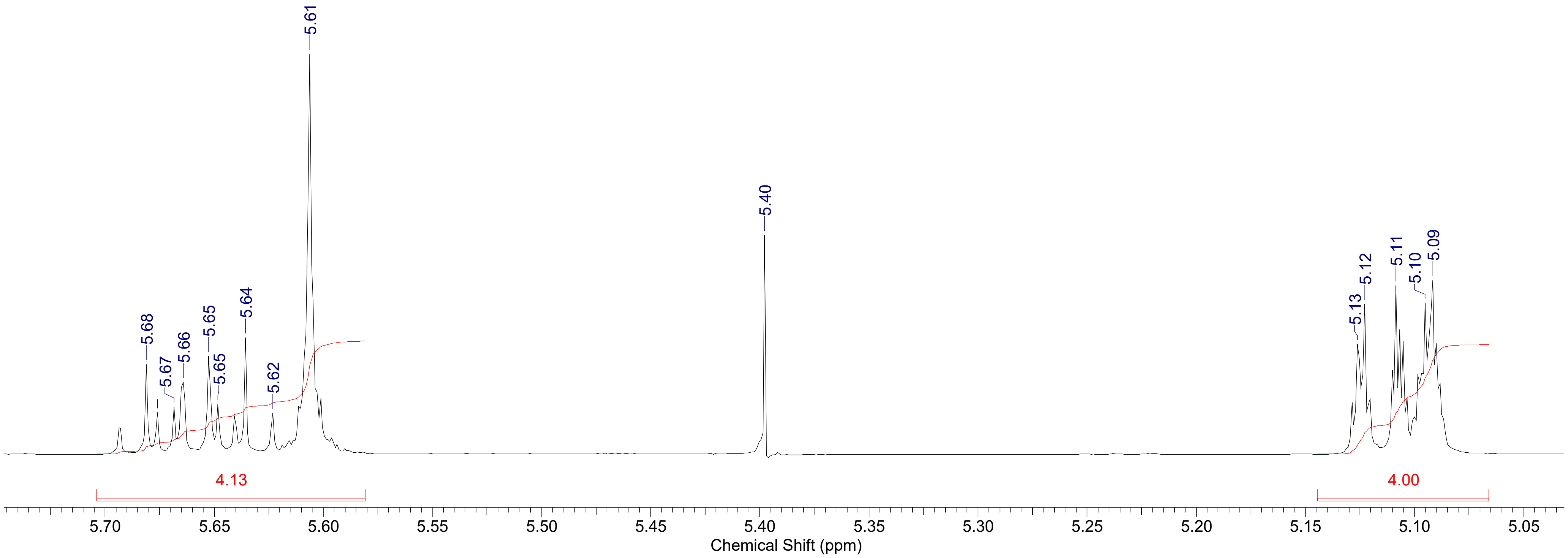

GC-MS analysis of a mixture of *diallyltosylamide (**19**)/ 1-tosyl-2,5-dihydro-1H-pyrrole (**20**)*, the established ratio is 49/51.

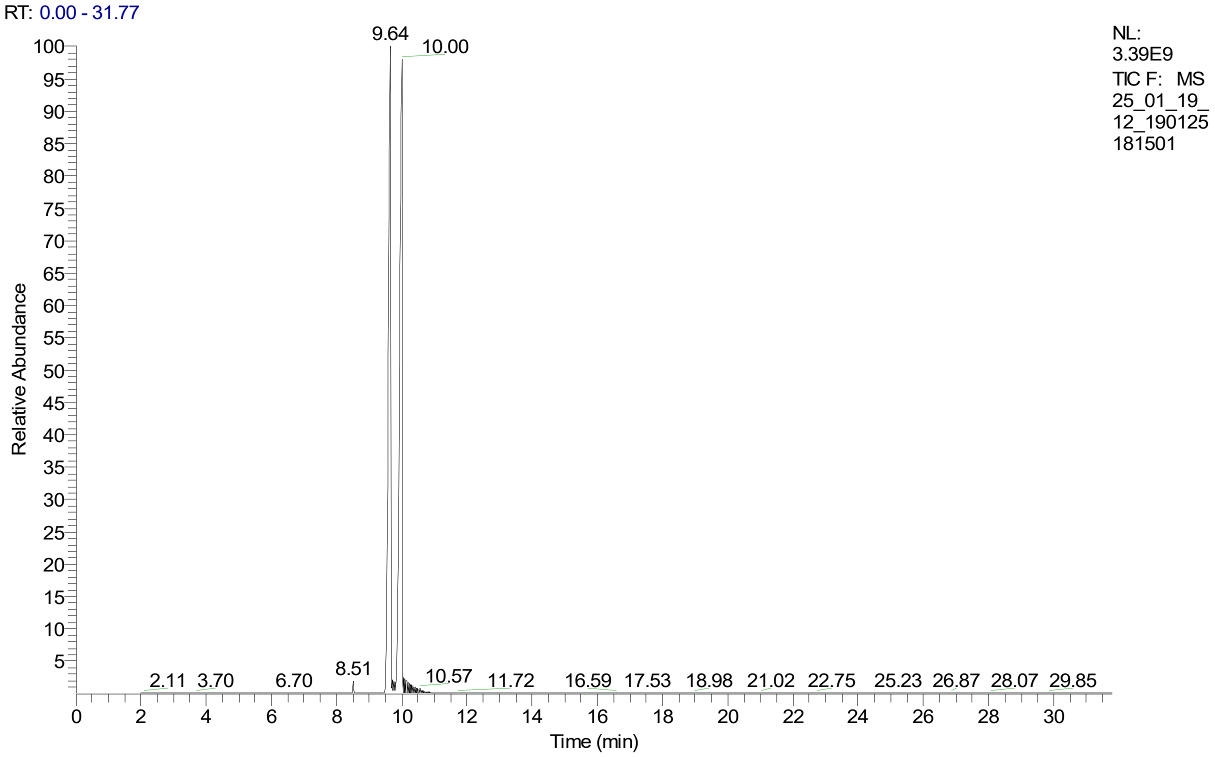

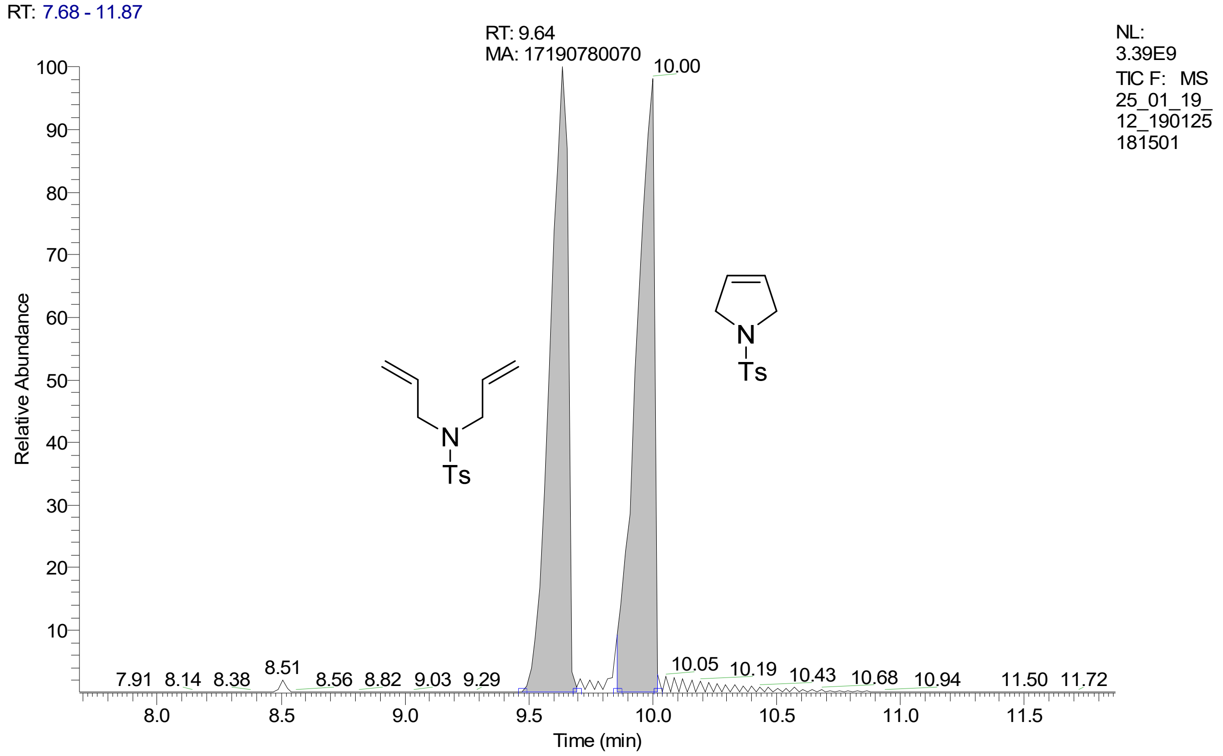
RT: 7.68 - 11.87
RT: 9.64
MA: 17190780070
10.00
NL:
3.39E9
TIC F: MS
25_01_19_
12_190125
181501
Relative Abundance
N
Ts
N
Ts
7.91 8.14 8.38 8.51 8.56 8.82 9.03 9.29
10.05 10.19 10.43 10.68 10.94 11.50 11.72
Time (min)

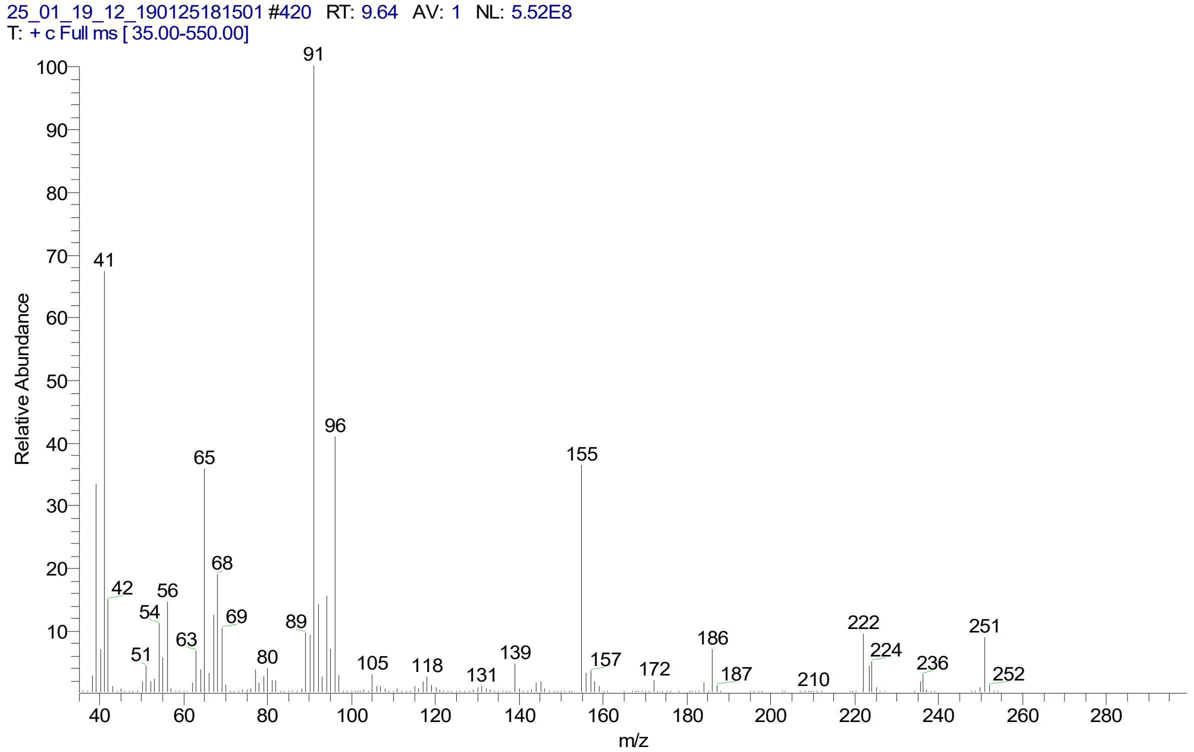
25_01_19_12_190125181501 #420 RT: 9.64 AV: 1 NL: 5.52E8
T: + c Full ms [ 35.00-550.00]
Relative Abundance
91
41
96
65
155
68
42
56
54
69
89
51
63
80
105
118
131
139
157
172
186
187
210
222
224
236
251
252
m/z

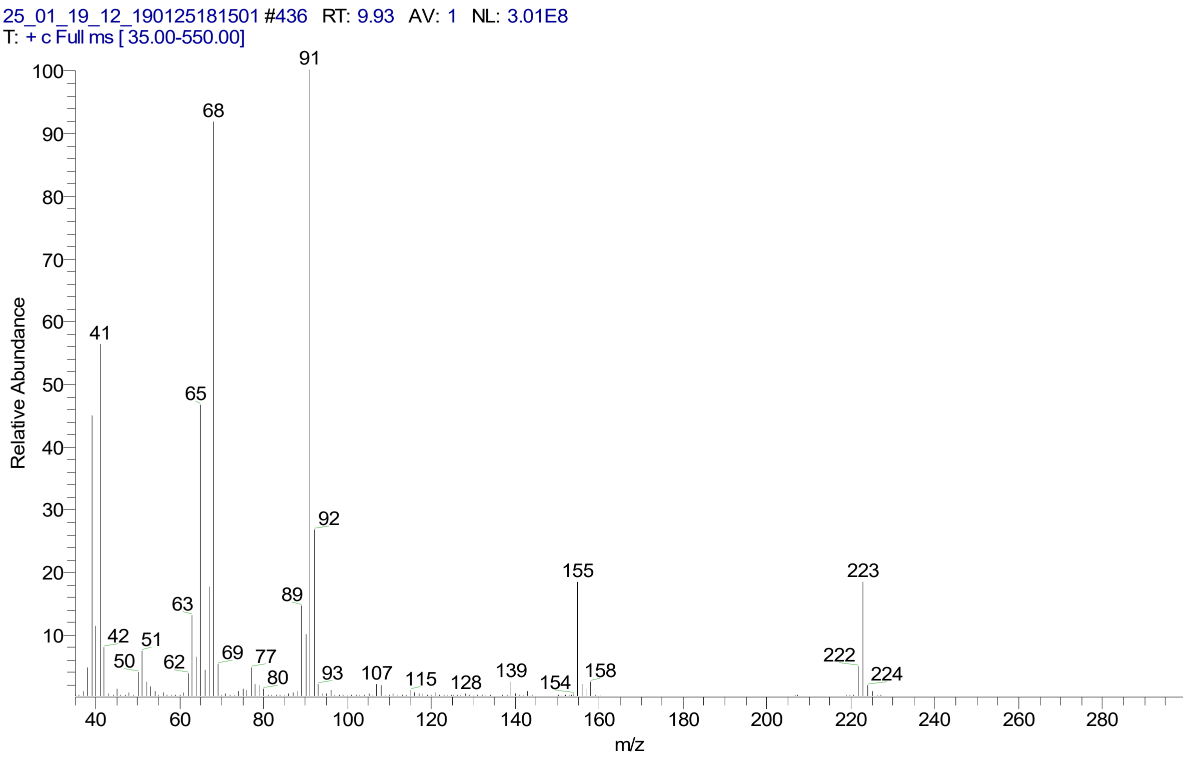


$^{1}$H NMR analysis of a mixture of of *diallyltosylamide* ***(19)****/ 1-tosyl-2,5-dihydro-1H-pyrrole* ***(20)***, the established ratio is 47/53.

| | | | | | | | |
|---|---|---|---|---|---|---|---|
| ***Acquisition Time (sec)*** | 1.9818 | ***Comment*** | single_pulse | ***Date*** | 22 Feb 1990 16:28:00 | ***Date Stamp*** | 25 Jan 2019 12:10:57 |
| ***File Name*** | C:\Users\Fedor\Desktop\25.01.19\FZ7122-1.jdf | | | ***Frequency (MHz)*** | 600.17 | ***Nucleus*** 1H | ***Number of Transients*** 8 |
| ***Origin*** | ECA 600 | ***Original Points Count*** | 32768 | ***Owner*** | delta | ***Points Count*** 32768 | ***Pulse Sequence*** single_pulse.ex2 |
| ***Receiver Gain*** | 34.00 | ***Solvent*** | CHLOROFORM-d | | | ***Spectrum Offset (Hz)*** 5414.6641 | ***Sweep Width (Hz)*** 16534.39 |

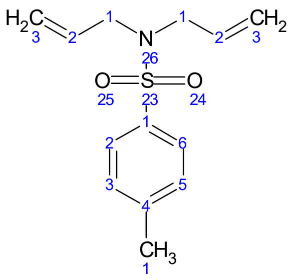

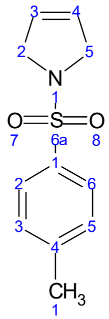

FZ7122-1.jdf

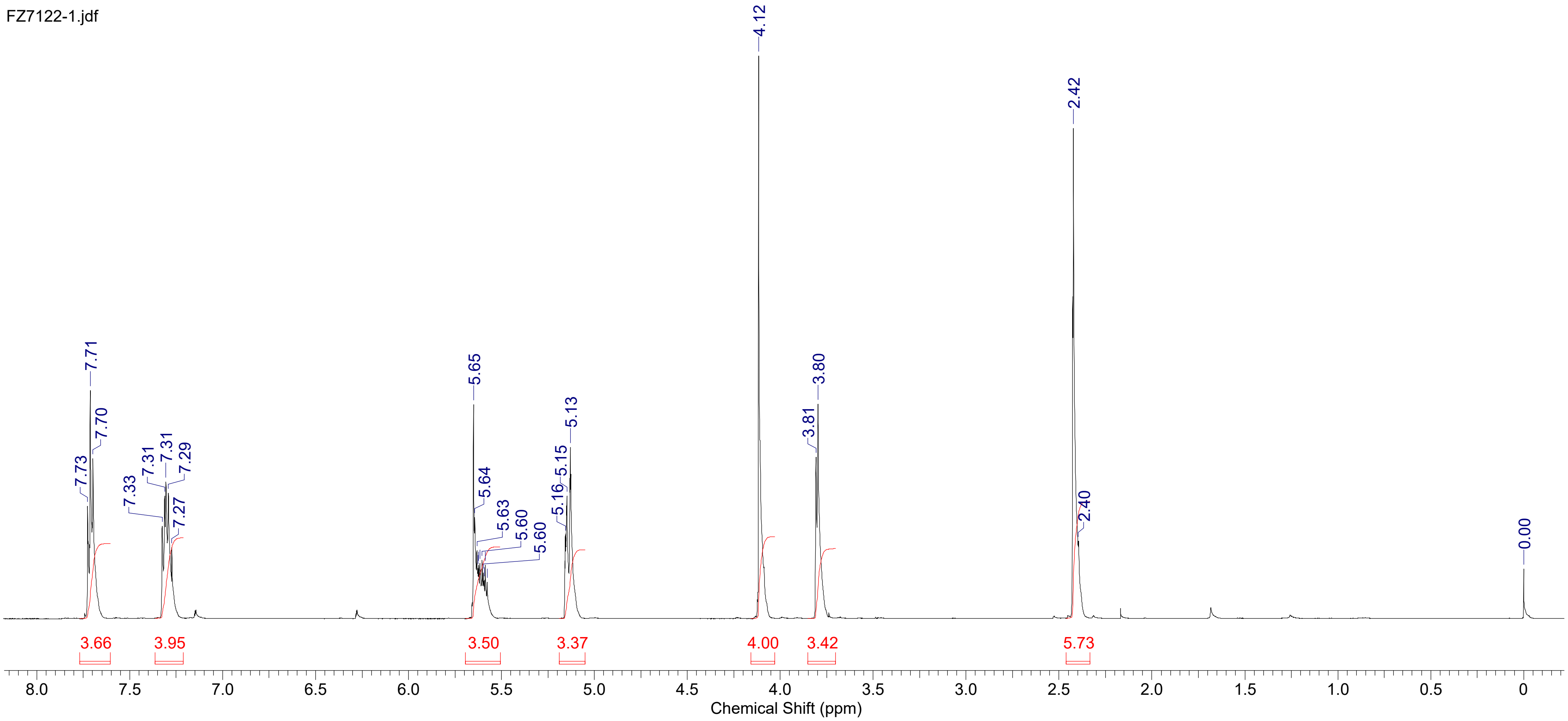

| | | | | | | | |
|---|---|---|---|---|---|---|---|
| ***Acquisition Time (sec)*** | 1.9818 | ***Comment*** | single_pulse | ***Date*** | 22 Feb 1990 16:28:00 | ***Date Stamp*** | 25 Jan 2019 12:10:57 |
| ***File Name*** | C:\Users\Fedor\Desktop\25.01.19\FZ7122-1.jdf | | | ***Frequency (MHz)*** | 600.17 | ***Nucleus*** | 1H |
| ***Number of Transients*** | 8 | ***Origin*** | ECA 600 | ***Original Points Count*** | 32768 | ***Owner*** | delta |
| ***Points Count*** | 32768 | ***Pulse Sequence*** | single_pulse.ex2 | ***Receiver Gain*** | 34.00 | ***Solvent*** | CHLOROFORM-d |
| ***Spectrum Offset (Hz)*** | 5414.6641 | ***Sweep Width (Hz)*** | 16534.39 | | | | |

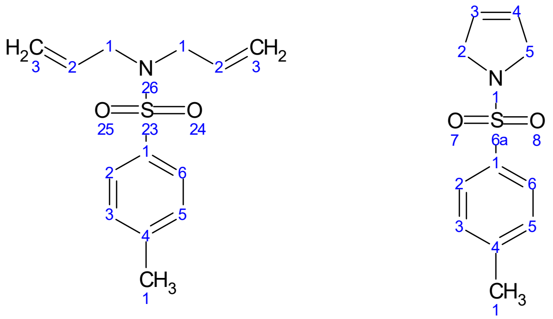

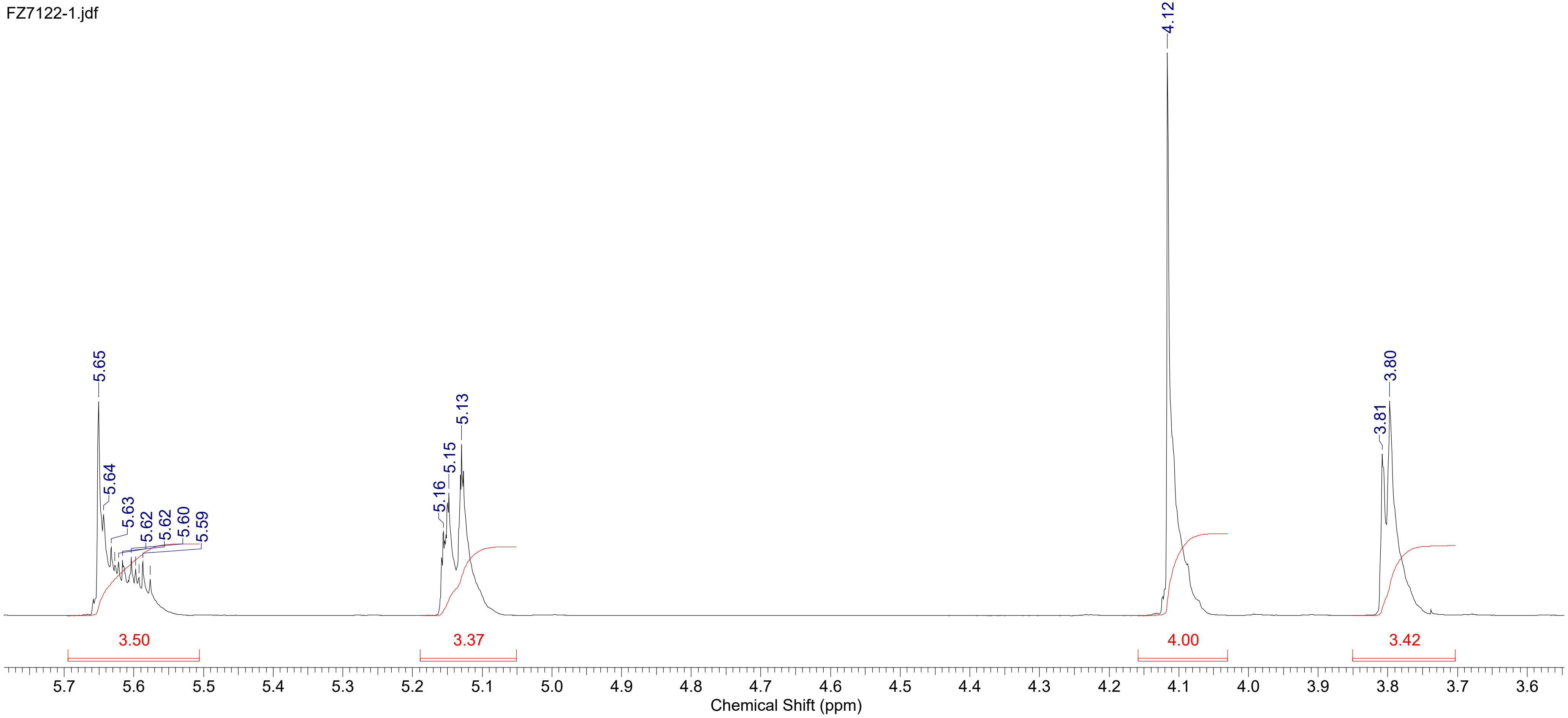

# 4. Experimental details and GPC analysis of polymer materials obtained in the metathesis reaction between norbornene **21** and styrene **12** (see Entry 35, Table 3).

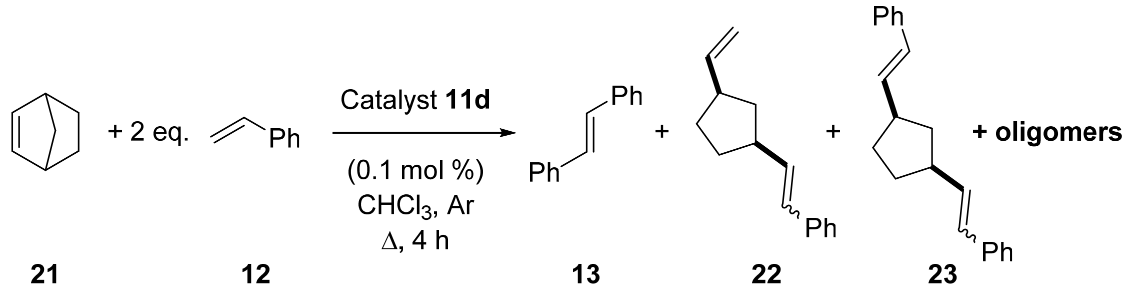

A mixture of norbornene **21** (0.19 g, 2.0 mmol) and styrene **12** (0.46 mL, 4.0 mmol) was added to a solution of catalyst (**11d**) 0.1 mol % (1.34 mg, 0.002 mmol) in dry chloroform (10 mL) in a Schlenk flask purged with argon. The resulting mixture was heated at reflux for 4 h under continuous stirring and in an argon atmosphere (after this time the green color of the reaction mixture changes to yellow). Inorganic products were separated by filtration through a layer of $Al_2O_3$ (2×2 cm, neutral, using $CHCl_3$ as eluent). The solvent was evaporated under reduced pressure. High-molecular products were separated by addition of MeOH (20 mL) and following filtration of a precipitate. The solution was concentrated under reduced pressure yield the mixture of non-polymeric compounds **13**, **22**, **23** as colourless oil (0.51 g, 78%). The composition of the mixture was determined by GC-MS analysis as **13**/**22**/**23**, ~ 81/18/1. Molecular weight of the obtained solid (0.06 g) was evaluated by gel permeation chromatography (GPC) relative to a polystyrene standard reference material. GPC analysis was performed on a Dionex Ultimate 3000 device with a refractometric detector for 1% solution of the polymer in PhMe. The column Agilent PLgel 5 μm MIXED-C, 7.5 × 300 mm and PhMe as a solvent have been used for separation of the components in GPC analysis. Total peak area (%), weight-average molecular weight (Da), polydispersity: peak 1, a norbornene oligomer (7.9 %, 923 Da, 1.55), peak 2, an impurity or a noise (0.2 %, 109 Da, 1.02), peak 3, a solvent (91.9 %, 33 Da, 1.04) (see Pictures below).

# Sample Report

| | | | |
|---|---|---|---|
| *Sample Name* | PK-1210 | *Quantif. Method* | GPC |
| *Sequence* | GPC_test-04.2018 | *Start Time* | 2018-12-11 14:48 |
| *Directory* | ROSNEFT\2_Data | *Injection Volume* | 50,0 |
| *Sample Type* | unknown | *Channel* | RI_1 |
| *Control Program* | GPC | *Number of Peak Slices* | 25 |

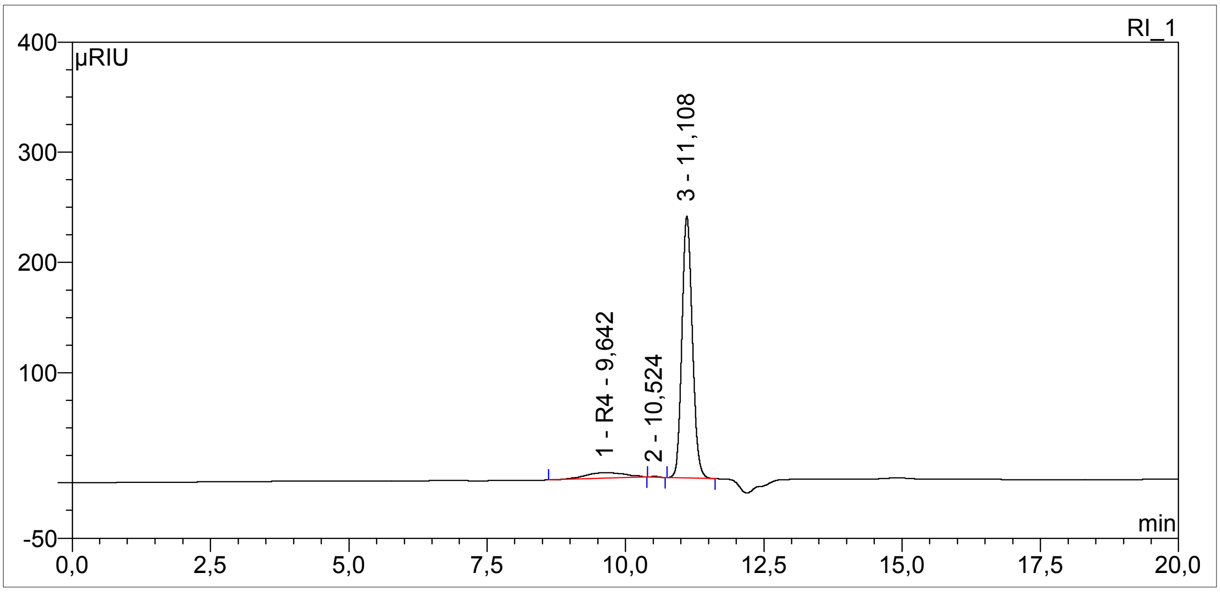


| **Peaks** | | | |
|---|---|---|---|
| *Peak number* | 1 | 2 | 3 |
| *Peak name* | R4 | n.a. | n.a. |
| **Peak Parameters** | | | |
| *Ret. Time Peak Start [min]* | 8,61 | 10,39 | 10,75 |
| *Mw (Peak Start) [Da]* | 6450 | 146 | 68 |
| *Ret. Time Peak Maximum [min]* | 9,64 | 10,52 | 11,11 |
| *Mw (Peak Maximum) [Da]* | 724 | 111 | 32 |
| *Ret. Time Peak End [min]* | 10,39 | 10,72 | 11,62 |
| *Mw (Peak End) [Da]* | 147 | 73 | 11 |
| *Peak Area [rel. units]* | 4,42 | 0,12 | 51,43 |
| *% Total Peak Area* | 7,90 | 0,22 | 91,88 |
| **Averages** | | | |
| *Number Average Mn* | 595 | 107 | 30 |
| *Weight Average Mw* | 923 | 109 | 32 |
| *Z Average Mz* | 1390 | 110 | 33 |
| *(Z+1) Average Mz+1* | 1930 | 112 | 34 |
| **Polydispersity** | | | |
| *Polydispersity PD* | 1,55 | 1,02 | 1,04 |

# Molecular Weight Distribution Report: R4

**Sample**

| | |
|---|---|
| *Sample Name* | PK-1210 |
| *Sequence* | GPC_test-04.2018 |
| *Directory* | ROSNEFT\2_Data |
| *Sample Type* | unknown |
| *Control Program* | GPC |
| *Quantif. Method* | GPC |
| *Start Time* | 2018-12-11 14:48 |
| *Injection Volume* | 50,0 |
| *Channel* | RI_1 |
| *Number of Peak Slices* | 25 |

**Peak Parameters**

| | |
|---|---|
| *Peak number* | 1 |
| *Peak name* | R4 |
| *Ret. Time Peak Start [min]* | 8,61 |
| *Mw (Peak Start) [Da]* | 6450 |
| *Ret. Time Peak Maximum [min]* | 9,64 |
| *Mw (Peak Maximum) [Da]* | 724 |
| *Ret. Time Peak End [min]* | 10,39 |
| *Mw (Peak End) [Da]* | 147 |
| *Peak Area [rel. units]* | 4,42 |
| *% Total Peak Area* | 7,90 |

**Averages and Polydispersity**

| | |
|---|---|
| *Number Average Mn* | 595 |
| *Weight Average Mw* | 923 |
| *Z Average Mz* | 1390 |
| *(Z+1) Average Mz+1* | 1930 |
| *Polydispersity PD* | 1,55 |

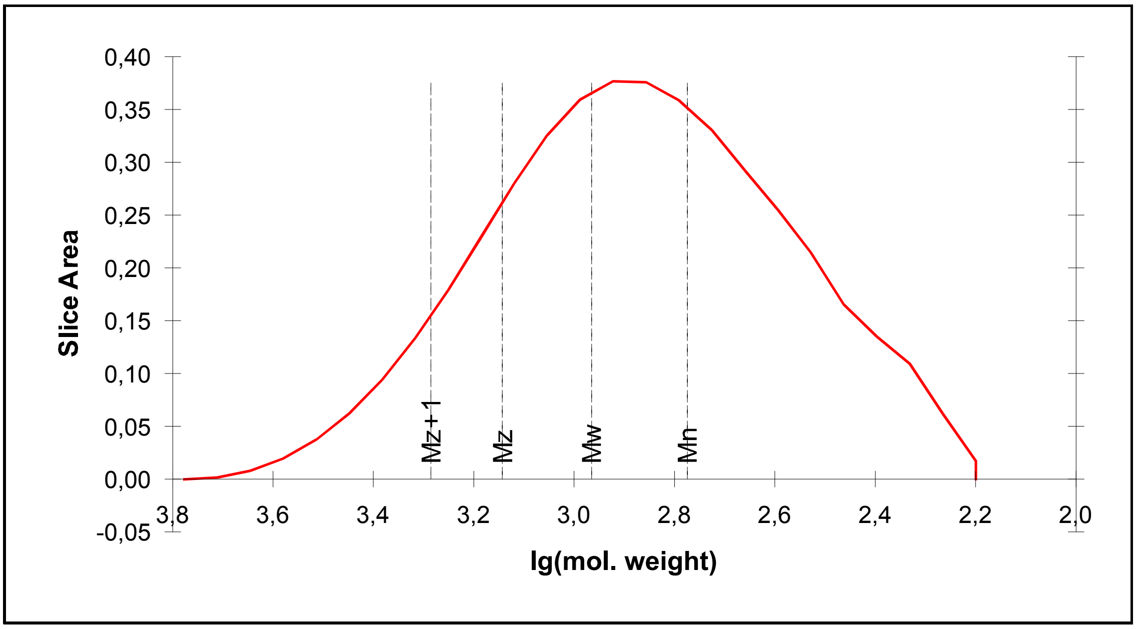


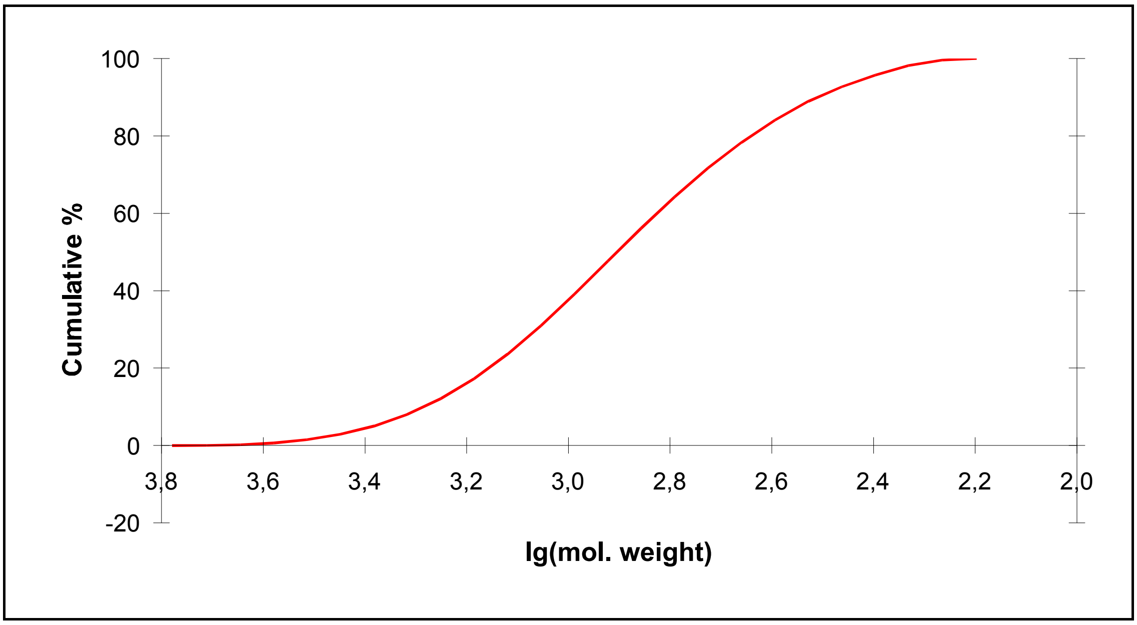


GPC/Distribution_Curve_1
Chromeleon 6.80 SR15b Build 4981 (268185)

Date Printed: 11-Dec-2018 15:55:30

# Slice Detail Report: R4

**Sample**

| | |
|---|---|
| *Sample Name* | PK-1210 |
| *Sequence* | GPC_test-04.2018 |
| *Directory* | ROSNEFT\2_Data |
| *Sample Type* | unknown |
| *Control Program* | GPC |
| *Quantif. Method* | GPC |
| *Start Time* | 2018-12-11 14:48 |
| *Injection Volume* | 50,0 |
| *Channel* | RI_1 |
| *Number of Peak Slices* | 25 |

**Peak Parameters**

| | |
|---|---|
| *Peak number* | 1 |
| *Peak name* | R4 |
| *Ret. Time Peak Start [min]* | 8,61 |
| *Mw (Peak Start) [Da]* | 6450 |
| *Ret. Time Peak Maximum [min]* | 9,64 |
| *Mw (Peak Maximum) [Da]* | 724 |
| *Ret. Time Peak End [min]* | 10,39 |
| *Mw (Peak End) [Da]* | 147 |
| *Peak Area [rel. units]* | 4,4 |
| *% Total Peak Area* | 7,90 |

**Averages and Polydispersity**

| | |
|---|---|
| *Number Average Mn* | 595 |
| *Weight Average Mw* | 923 |
| *Z Average Mz* | 1390 |
| *(Z+1) Average Mz+1* | 1930 |
| *Polydispersity PD* | 1,55 |

| Slice No. | Slice Start [min] | Slice End [min] | lg(Mw) | Mw [Da] | Slice Area [rel. units] | Percentage [%] | Cumulative [%] |
|---|---|---|---|---|---|---|---|
| 1 | 8,614 | 8,685 | 3,777 | 5980 | -0,00016 | -0,004 | -0,004 |
| 2 | 8,685 | 8,756 | 3,711 | 5140 | 0,00160 | 0,036 | 0,033 |
| 3 | 8,756 | 8,827 | 3,645 | 4418 | 0,00790 | 0,179 | 0,211 |
| 4 | 8,827 | 8,898 | 3,580 | 3798 | 0,01953 | 0,442 | 0,653 |
| 5 | 8,898 | 8,969 | 3,514 | 3265 | 0,03732 | 0,844 | 1,497 |
| 6 | 8,969 | 9,040 | 3,448 | 2806 | 0,06205 | 1,403 | 2,901 |
| 7 | 9,040 | 9,111 | 3,382 | 2412 | 0,09414 | 2,129 | 5,030 |
| 8 | 9,111 | 9,182 | 3,317 | 2073 | 0,13349 | 3,019 | 8,049 |
| 9 | 9,182 | 9,254 | 3,251 | 1782 | 0,17922 | 4,054 | 12,103 |
| 10 | 9,254 | 9,325 | 3,185 | 1532 | 0,22928 | 5,186 | 17,289 |
| 11 | 9,325 | 9,396 | 3,120 | 1317 | 0,27999 | 6,333 | 23,622 |
| 12 | 9,396 | 9,467 | 3,054 | 1132 | 0,32555 | 7,363 | 30,985 |
| 13 | 9,467 | 9,538 | 2,988 | 973 | 0,35950 | 8,131 | 39,116 |
| 14 | 9,538 | 9,609 | 2,922 | 836 | 0,37679 | 8,522 | 47,639 |
| 15 | 9,609 | 9,680 | 2,857 | 719 | 0,37588 | 8,502 | 56,140 |
| 16 | 9,680 | 9,751 | 2,791 | 618 | 0,35864 | 8,112 | 64,252 |
| 17 | 9,751 | 9,822 | 2,725 | 531 | 0,33040 | 7,473 | 71,725 |
| 18 | 9,822 | 9,894 | 2,660 | 457 | 0,29215 | 6,608 | 78,333 |
| 19 | 9,894 | 9,965 | 2,594 | 393 | 0,25492 | 5,766 | 84,099 |
| 20 | 9,965 | 10,036 | 2,528 | 337 | 0,21458 | 4,853 | 88,953 |
| 21 | 10,036 | 10,107 | 2,462 | 290 | 0,16512 | 3,735 | 92,687 |
| 22 | 10,107 | 10,178 | 2,397 | 249 | 0,13491 | 3,051 | 95,739 |
| 23 | 10,178 | 10,249 | 2,331 | 214 | 0,10910 | 2,468 | 98,206 |
| 24 | 10,249 | 10,320 | 2,265 | 184 | 0,06203 | 1,403 | 99,609 |
| 25 | 10,320 | 10,391 | 2,200 | 158 | 0,01727 | 0,391 | 100,000 |

# Molecular Weight Distribution Report: Peak No. 2

**Sample**

| | |
|---|---|
| *Sample Name* | PK-1210 |
| *Sequence* | GPC_test-04.2018 |
| *Directory* | ROSNEFT\2_Data |
| *Sample Type* | unknown |
| *Control Program* | GPC |
| *Quantif. Method* | GPC |
| *Start Time* | 2018-12-11 14:48 |
| *Injection Volume* | 50,0 |
| *Channel* | RI_1 |
| *Number of Peak Slices* | 25 |

**Peak Parameters**

| | |
|---|---|
| *Peak number* | 2 |
| *Peak name* | n.a. |
| *Ret. Time Peak Start [min]* | 10,39 |
| *Mw (Peak Start) [Da]* | 146 |
| *Ret. Time Peak Maximum [min]* | 10,52 |
| *Mw (Peak Maximum) [Da]* | 111 |
| *Ret. Time Peak End [min]* | 10,72 |
| *Mw (Peak End) [Da]* | 73 |
| *Peak Area [rel. units]* | 0,12 |
| *% Total Peak Area* | 0,22 |

**Averages and Polydispersity**

| | |
|---|---|
| *Number Average Mn* | 107 |
| *Weight Average Mw* | 109 |
| *Z Average Mz* | 110 |
| *(Z+1) Average Mz+1* | 112 |
| *Polydispersity PD* | 1,02 |

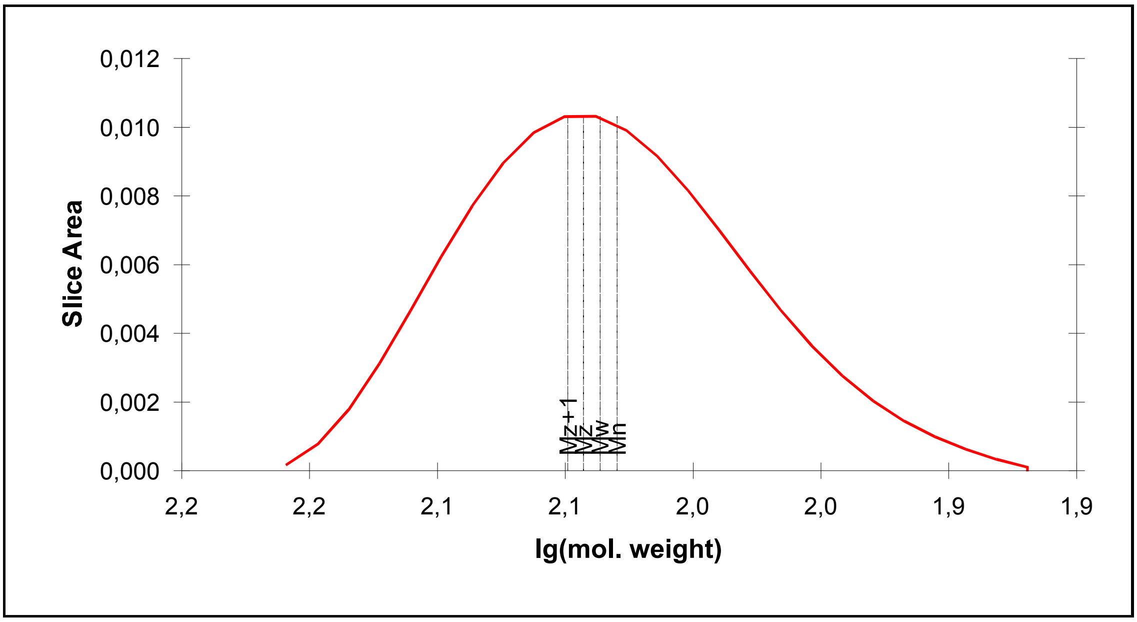


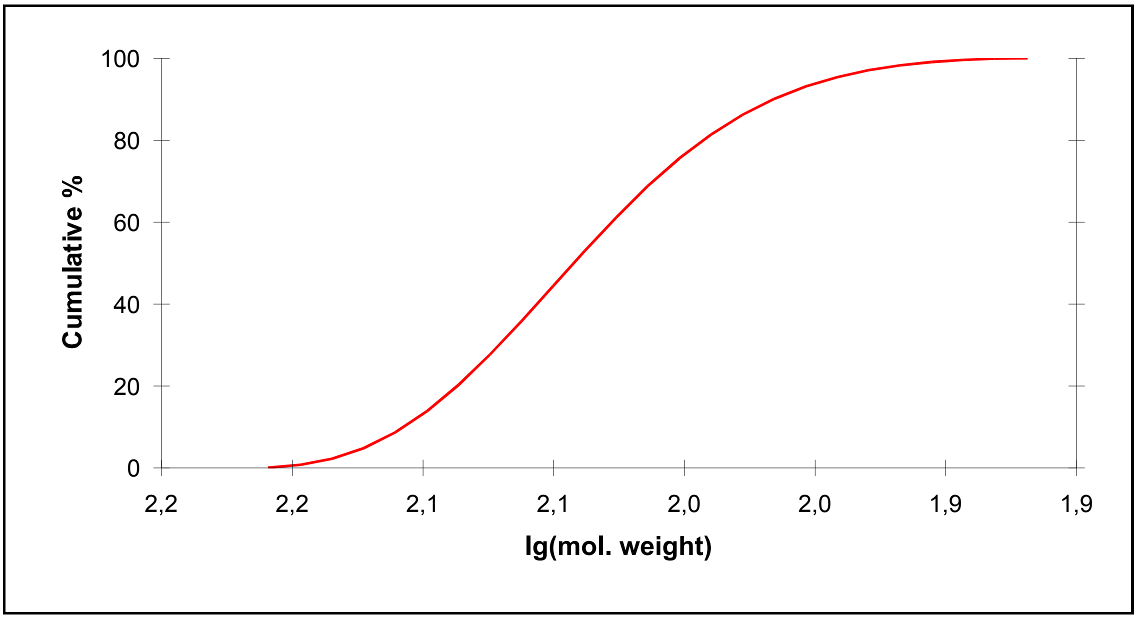

# Slice Detail Report: Peak No. 2

**Sample**

| | |
|---|---|
| *Sample Name* | PK-1210 |
| *Sequence* | GPC_test-04.2018 |
| *Directory* | ROSNEFT\2_Data |
| *Sample Type* | unknown |
| *Control Program* | GPC |
| *Quantif. Method* | GPC |
| *Start Time* | 2018-12-11 14:48 |
| *Injection Volume* | 50 |
| *Channel* | RI_1 |
| *Number of Peak Slices* | 25 |

**Peak Parameters**

| | |
|---|---|
| *Peak number* | 2 |
| *Peak name* | n.a. |
| *Ret. Time Peak Start [min]* | 10,39 |
| *Mw (Peak Start) [Da]* | 146 |
| *Ret. Time Peak Maximum [min]* | 10,52 |
| *Mw (Peak Maximum) [Da]* | 111 |
| *Ret. Time Peak End [min]* | 10,72 |
| *Mw (Peak End) [Da]* | 73 |
| *Peak Area [rel. units]* | 0,1 |
| *% Total Peak Area* | 0,22 |

**Averages and Polydispersity**

| | |
|---|---|
| *Number Average Mn* | 107 |
| *Weight Average Mw* | 109 |
| *Z Average Mz* | 110 |
| *(Z+1) Average Mz+1* | 112 |
| *Polydispersity PD* | 1,02 |

| Slice No. | Slice Start [min] | Slice End [min] | lg(Mw) | Mw [Da] | Slice Area [rel. units] | Percentage [%] | Cumulative [%] |
|---|---|---|---|---|---|---|---|
| 1 | 10,393 | 10,406 | 2,159 | 144 | 0,00019 | 0,156 | 0,156 |
| 2 | 10,406 | 10,419 | 2,147 | 140 | 0,00078 | 0,646 | 0,802 |
| 3 | 10,419 | 10,433 | 2,135 | 136 | 0,00178 | 1,476 | 2,278 |
| 4 | 10,433 | 10,446 | 2,123 | 133 | 0,00312 | 2,588 | 4,866 |
| 5 | 10,446 | 10,459 | 2,111 | 129 | 0,00466 | 3,864 | 8,731 |
| 6 | 10,459 | 10,472 | 2,099 | 125 | 0,00623 | 5,172 | 13,903 |
| 7 | 10,472 | 10,485 | 2,086 | 122 | 0,00771 | 6,394 | 20,297 |
| 8 | 10,485 | 10,498 | 2,074 | 119 | 0,00895 | 7,425 | 27,722 |
| 9 | 10,498 | 10,511 | 2,062 | 115 | 0,00984 | 8,166 | 35,888 |
| 10 | 10,511 | 10,524 | 2,050 | 112 | 0,01031 | 8,552 | 44,441 |
| 11 | 10,524 | 10,537 | 2,038 | 109 | 0,01032 | 8,559 | 53,000 |
| 12 | 10,537 | 10,550 | 2,026 | 106 | 0,00991 | 8,223 | 61,223 |
| 13 | 10,550 | 10,563 | 2,014 | 103 | 0,00916 | 7,600 | 68,823 |
| 14 | 10,563 | 10,576 | 2,002 | 100 | 0,00815 | 6,763 | 75,586 |
| 15 | 10,576 | 10,589 | 1,990 | 98 | 0,00701 | 5,813 | 81,400 |
| 16 | 10,589 | 10,602 | 1,978 | 95 | 0,00582 | 4,829 | 86,228 |
| 17 | 10,602 | 10,615 | 1,966 | 92 | 0,00467 | 3,878 | 90,106 |
| 18 | 10,615 | 10,628 | 1,954 | 90 | 0,00365 | 3,024 | 93,131 |
| 19 | 10,628 | 10,641 | 1,942 | 87 | 0,00276 | 2,291 | 95,422 |
| 20 | 10,641 | 10,655 | 1,930 | 85 | 0,00203 | 1,683 | 97,106 |
| 21 | 10,655 | 10,668 | 1,917 | 83 | 0,00144 | 1,198 | 98,304 |
| 22 | 10,668 | 10,681 | 1,905 | 80 | 0,00099 | 0,819 | 99,123 |
| 23 | 10,681 | 10,694 | 1,893 | 78 | 0,00062 | 0,518 | 99,641 |
| 24 | 10,694 | 10,707 | 1,881 | 76 | 0,00033 | 0,274 | 99,915 |
| 25 | 10,707 | 10,720 | 1,869 | 74 | 0,00010 | 0,085 | 100,000 |

# Molecular Weight Distribution Report: Peak No. 3

**Sample**

| | |
|---|---|
| *Sample Name* | PK-1210 |
| *Sequence* | GPC_test-04.2018 |
| *Directory* | ROSNEFT\2_Data |
| *Sample Type* | unknown |
| *Control Program* | GPC |
| *Quantif. Method* | GPC |
| *Start Time* | 2018-12-11 14:48 |
| *Injection Volume* | 50,0 |
| *Channel* | RI_1 |
| *Number of Peak Slices* | 25 |

**Peak Parameters**

| | |
|---|---|
| *Peak number* | 3 |
| *Peak name* | n.a. |
| *Ret. Time Peak Start [min]* | 10,75 |
| *Mw (Peak Start) [Da]* | 68 |
| *Ret. Time Peak Maximum [min]* | 11,11 |
| *Mw (Peak Maximum) [Da]* | 32 |
| *Ret. Time Peak End [min]* | 11,62 |
| *Mw (Peak End) [Da]* | 11 |
| *Peak Area [rel. units]* | 51,43 |
| *% Total Peak Area* | 91,88 |

**Averages and Polydispersity**

| | |
|---|---|
| *Number Average Mn* | 30 |
| *Weight Average Mw* | 32 |
| *Z Average Mz* | 33 |
| *(Z+1) Average Mz+1* | 34 |
| *Polydispersity PD* | 1,04 |

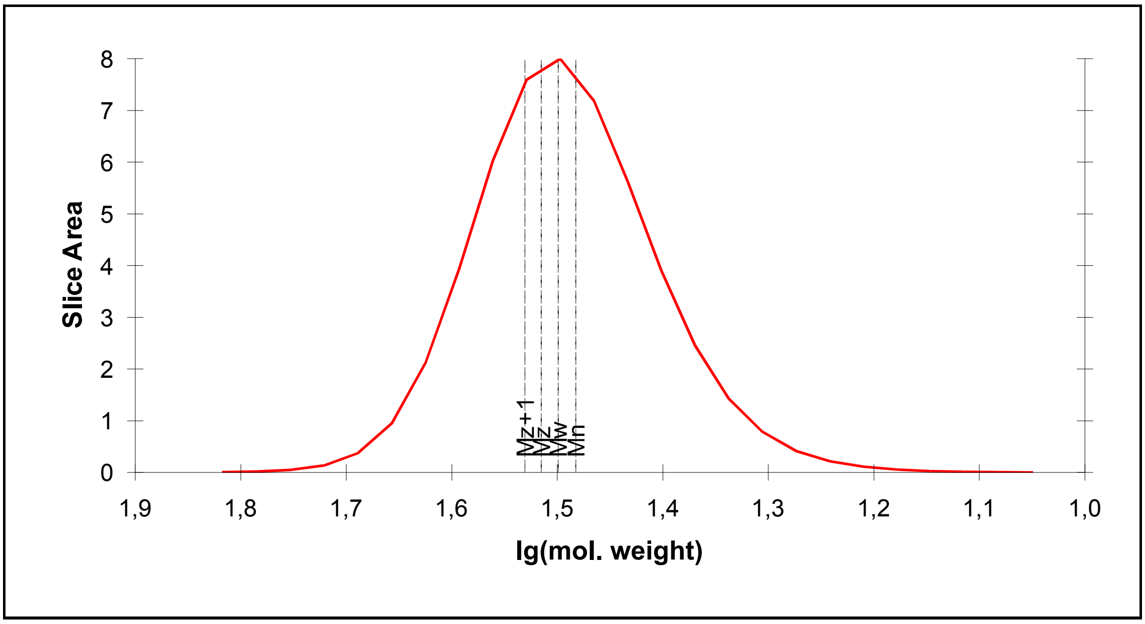


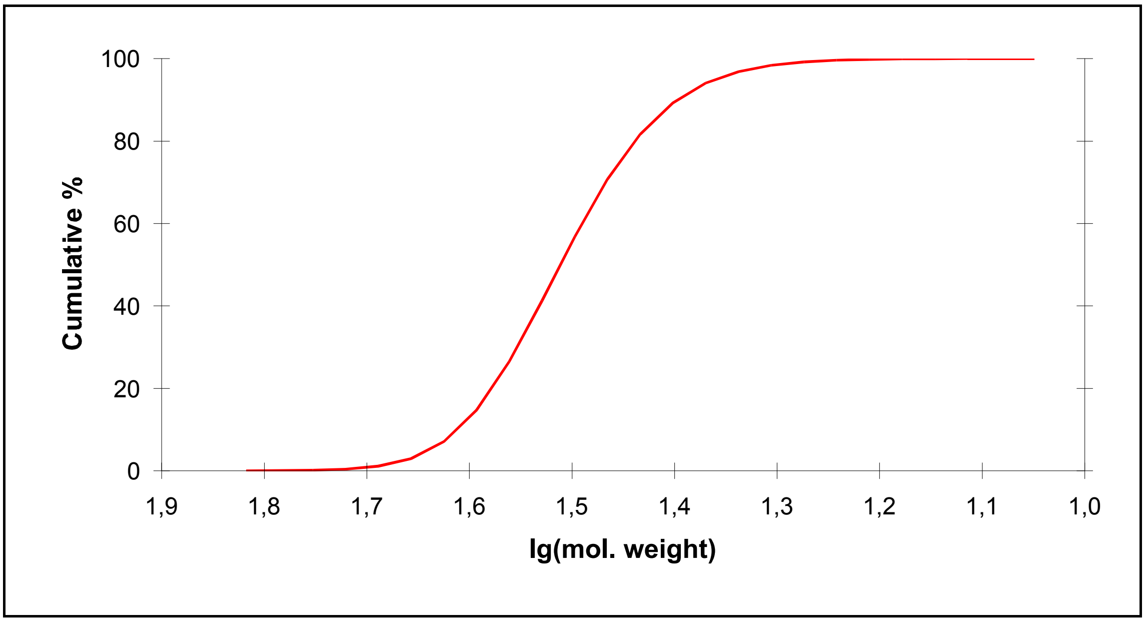


GPC/Distribution_Curve_3
Chromeleon 6.80 SR15b Build 4981 (268185)

Date Printed: 11-Dec-2018 15:57:27

# Slice Detail Report: Peak No. 3

**Sample**

| | |
|---|---|
| *Sample Name* | PK-1210 |
| *Sequence* | GPC_test-04.2018 |
| *Directory* | ROSNEFT\2_Data |
| *Sample Type* | unknown |
| *Control Program* | GPC |
| *Quantif. Method* | GPC |
| *Start Time* | 2018-12-11 14:48 |
| *Injection Volume* | 50 |
| *Channel* | RI_1 |
| *Number of Peak Slices* | 25 |

**Peak Parameters**

| | |
|---|---|
| Peak number | 3 |
| Peak name | n.a. |
| Ret. Time Peak Start [min] | 10,75 |
| Mw (Peak Start) [Da] | 68 |
| Ret. Time Peak Maximum [min] | 11,11 |
| Mw (Peak Maximum) [Da] | 32 |
| Ret. Time Peak End [min] | 11,62 |
| Mw (Peak End) [Da] | 11 |
| Peak Area [rel. units] | 51,4 |
| % Total Peak Area | 91,88 |

**Averages and Polydispersity**

| | |
|---|---|
| Number Average Mn | 30 |
| Weight Average Mw | 32 |
| Z Average Mz | 33 |
| (Z+1) Average Mz+1 | 34 |
| Polydispersity PD | 1,04 |

| Slice No. | Slice Start [min] | Slice End [min] | lg(Mw) | Mw [Da] | Slice Area [rel. units] | Percentage [%] | Cumulative [%] |
|---|---|---|---|---|---|---|---|
| 1 | 10,753 | 10,788 | 1,816 | 66 | 0,00306 | 0,006 | 0,006 |
| 2 | 10,788 | 10,822 | 1,784 | 61 | 0,01538 | 0,030 | 0,036 |
| 3 | 10,822 | 10,857 | 1,753 | 57 | 0,04908 | 0,095 | 0,131 |
| 4 | 10,857 | 10,891 | 1,721 | 53 | 0,13820 | 0,269 | 0,400 |
| 5 | 10,891 | 10,926 | 1,689 | 49 | 0,37508 | 0,729 | 1,129 |
| 6 | 10,926 | 10,960 | 1,657 | 45 | 0,95165 | 1,850 | 2,979 |
| 7 | 10,960 | 10,995 | 1,625 | 42 | 2,11819 | 4,118 | 7,097 |
| 8 | 10,995 | 11,029 | 1,593 | 39 | 3,94277 | 7,665 | 14,762 |
| 9 | 11,029 | 11,064 | 1,561 | 36 | 6,02650 | 11,716 | 26,477 |
| 10 | 11,064 | 11,099 | 1,529 | 34 | 7,59118 | 14,757 | 41,234 |
| 11 | 11,099 | 11,133 | 1,497 | 31 | 7,99992 | 15,552 | 56,786 |
| 12 | 11,133 | 11,168 | 1,465 | 29 | 7,18934 | 13,976 | 70,762 |
| 13 | 11,168 | 11,202 | 1,433 | 27 | 5,62312 | 10,931 | 81,694 |
| 14 | 11,202 | 11,237 | 1,401 | 25 | 3,90571 | 7,593 | 89,286 |
| 15 | 11,237 | 11,271 | 1,370 | 23 | 2,45754 | 4,777 | 94,064 |
| 16 | 11,271 | 11,306 | 1,338 | 22 | 1,43084 | 2,782 | 96,845 |
| 17 | 11,306 | 11,340 | 1,306 | 20 | 0,78610 | 1,528 | 98,373 |
| 18 | 11,340 | 11,375 | 1,274 | 19 | 0,41561 | 0,808 | 99,181 |
| 19 | 11,375 | 11,409 | 1,242 | 17 | 0,21479 | 0,418 | 99,599 |
| 20 | 11,409 | 11,444 | 1,210 | 16 | 0,10939 | 0,213 | 99,812 |
| 21 | 11,444 | 11,479 | 1,178 | 15 | 0,05463 | 0,106 | 99,918 |
| 22 | 11,479 | 11,513 | 1,146 | 14 | 0,02611 | 0,051 | 99,969 |
| 23 | 11,513 | 11,548 | 1,114 | 13 | 0,01136 | 0,022 | 99,991 |
| 24 | 11,548 | 11,582 | 1,082 | 12 | 0,00400 | 0,008 | 99,998 |
| 25 | 11,582 | 11,617 | 1,050 | 11 | 0,00080 | 0,002 | 100,000 |

5. Check-cif reports (CCDC) for compounds **11a**, **11b**, **11c**.

# checkCIF/PLATON report

You have not supplied any structure factors. As a result the full set of tests cannot be run.

THIS REPORT IS FOR GUIDANCE ONLY. IF USED AS PART OF A REVIEW PROCEDURE FOR PUBLICATION, IT SHOULD NOT REPLACE THE EXPERTISE OF AN EXPERIENCED CRYSTALLOGRAPHIC REFEREE.

No syntax errors found.  CIF dictionary  Interpreting this report

# Datablock: 11a

---

```
Bond precision:  C-C = 0.0046 A          Wavelength=0.71073

Cell:           a=10.208(6)       b=10.837(7)      c=14.745(6)
                alpha=76.76(2)    beta=85.41(2)    gamma=70.66(2)
Temperature:    295 K

                Calculated                 Reported
Volume          1498.2(15)                 1498.2(15)
Space group     P -1                       P -1
Hall group      -P 1                       -P 1
Moiety formula  C31 H39 Cl2 N3 Ru          C31 H39 Cl2 N3 Ru
Sum formula     C31 H39 Cl2 N3 Ru          C31 H39 Cl2 N3 Ru
Mr              625.62                     625.62
Dx,g cm-3       1.387                      1.389
Z               2                          2
Mu (mm-1)       0.726                      0.726
F000            648.0                      648.0
F000'           646.23
h,k,lmax        12,13,18                   12,13,18
Nref            5858                       5856
Tmin,Tmax       0.865,0.865                0.792,0.864
Tmin'           0.865

Correction method= # Reported T Limits: Tmin=0.792 Tmax=0.864
AbsCorr = PSI-SCAN

Data completeness= 1.000          Theta(max)= 25.970

R(reflections)= 0.0368( 4768)     wR2(reflections)= 0.0762( 5856)

S = 0.992                   Npar= 342
```

---

The following ALERTS were generated. Each ALERT has the format
**test-name_ALERT_alert-type_alert-level**.
Click on the hyperlinks for more details of the test.

**Alert level G**

```
PLAT005_ALERT_5_G No Embedded Refinement Details found  in the CIF     Please Do !
PLAT154_ALERT_1_G The s.u.'s on the Cell Angles are Equal ..(Note)       0.02 Degree
PLAT232_ALERT_2_G Hirshfeld Test Diff (M-X)  Ru     --  Cl2     ..        5.8 s.u.
PLAT899_ALERT_4_G SHELXL97   is Deprecated and Succeeded by SHELXL       2016 Note
```

---

```
   0 ALERT level A = Most likely a serious problem - resolve or explain
   0 ALERT level B = A potentially serious problem, consider carefully
   0 ALERT level C = Check. Ensure it is not caused by an omission or oversight
   4 ALERT level G = General information/check it is not something unexpected

   1 ALERT type 1 CIF construction/syntax error, inconsistent or missing data
   1 ALERT type 2 Indicator that the structure model may be wrong or deficient
   0 ALERT type 3 Indicator that the structure quality may be low
   1 ALERT type 4 Improvement, methodology, query or suggestion
   1 ALERT type 5 Informative message, check
```

---

It is advisable to attempt to resolve as many as possible of the alerts in all categories. Often the minor alerts point to easily fixed oversights, errors and omissions in your CIF or refinement strategy, so attention to these fine details can be worthwhile. In order to resolve some of the more serious problems it may be necessary to carry out additional measurements or structure refinements. However, the purpose of your study may justify the reported deviations and the more serious of these should normally be commented upon in the discussion or experimental section of a paper or in the "special_details" fields of the CIF. checkCIF was carefully designed to identify outliers and unusual parameters, but every test has its limitations and alerts that are not important in a particular case may appear. Conversely, the absence of alerts does not guarantee there are no aspects of the results needing attention. It is up to the individual to critically assess their own results and, if necessary, seek expert advice.

**Publication of your CIF in IUCr journals**

A basic structural check has been run on your CIF. These basic checks will be run on all CIFs submitted for publication in IUCr journals (*Acta Crystallographica*, *Journal of Applied Crystallography*, *Journal of Synchrotron Radiation*); however, if you intend to submit to *Acta Crystallographica Section C* or *E* or *IUCrData*, you should make sure that full publication checks are run on the final version of your CIF prior to submission.

**Publication of your CIF in other journals**

Please refer to the *Notes for Authors* of the relevant journal for any special instructions relating to CIF submission.

---

**PLATON version of 13/08/2017; check.def file version of 27/07/2017**

**Datablock bab1213** - ellipsoid plot

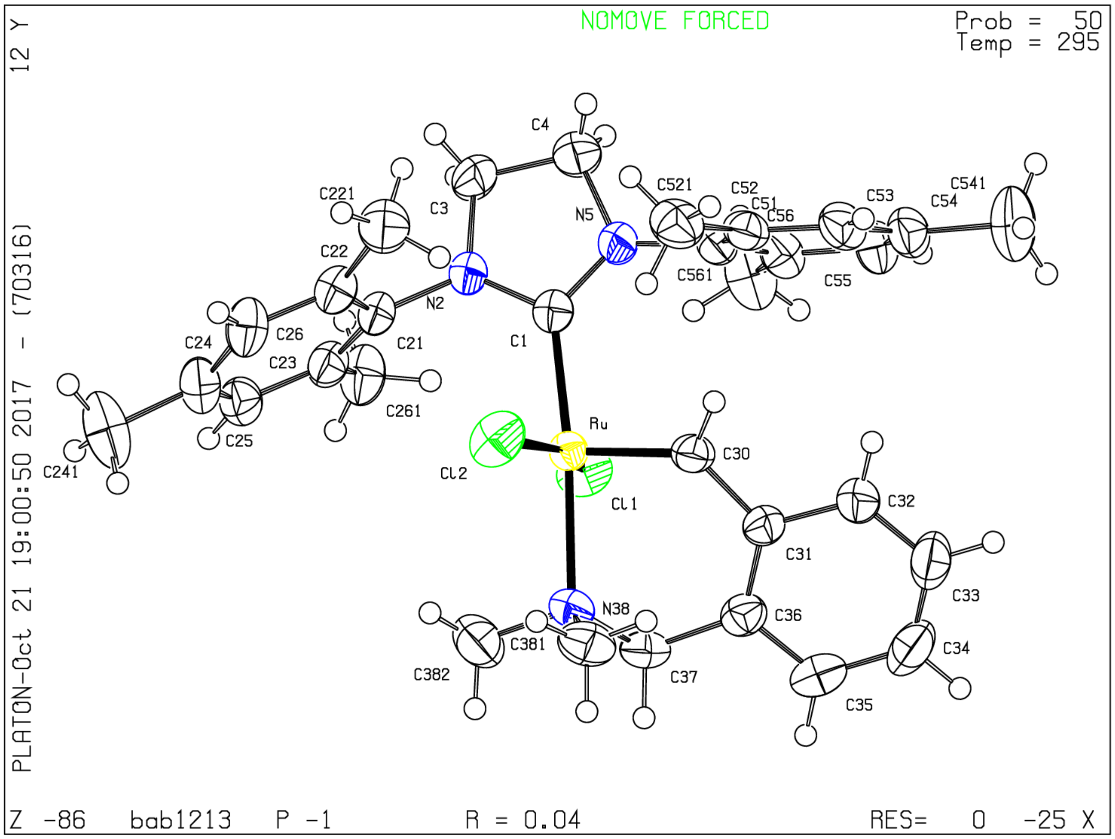

# checkCIF/PLATON report

Structure factors have been supplied for datablock(s) K1

THIS REPORT IS FOR GUIDANCE ONLY. IF USED AS PART OF A REVIEW PROCEDURE FOR PUBLICATION, IT SHOULD NOT REPLACE THE EXPERTISE OF AN EXPERIENCED CRYSTALLOGRAPHIC REFEREE.

No syntax errors found.  CIF dictionary  Interpreting this report

## Datablock: 11b

---

```
Bond precision:  C-C = 0.0074 A         Wavelength=0.71073

Cell:           a=21.218(3)    b=19.218(2)       c=15.3386(19)
                alpha=90       beta=95.830(2)    gamma=90
Temperature:    100 K

                Calculated                 Reported
Volume          6222.2(13)                 6222.4(13)
Space group     P 21/c                     P 21/c
Hall group      -P 2ybc                    -P 2ybc
Moiety formula  C33 H43 Cl2 N3 Ru          C33 H43 Cl2 N3 Ru
Sum formula     C33 H43 Cl2 N3 Ru          C33 H43 Cl2 N3 Ru
Mr              653.67                     653.67
Dx,g cm-3       1.396                      1.396
Z               8                          8
Mu (mm-1)       0.702                      0.702
F000            2720.0                     2720.0
F000'           2712.95
h,k,lmax        26,23,18                   26,23,18
Nref            12238                      12236
Tmin,Tmax       0.838,0.932                0.834,0.921
Tmin'           0.822

Correction method= # Reported T Limits: Tmin=0.834 Tmax=0.921
AbsCorr = MULTI-SCAN

Data completeness= 1.000          Theta(max)= 25.999

R(reflections)= 0.0612( 9638)     wR2(reflections)= 0.1547( 12236)

S = 1.203                  Npar= 719
```

---

The following ALERTS were generated. Each ALERT has the format
**test-name_ALERT_alert-type_alert-level**.
Click on the hyperlinks for more details of the test.

### Alert level B

```
PLAT971_ALERT_2_B Check Calcd Resid. Dens.  0.94A   From Ru1          2.83 eA-3
PLAT971_ALERT_2_B Check Calcd Resid. Dens.  0.97A   From Ru1          2.66 eA-3
```

### Alert level C

```
PLAT094_ALERT_2_C Ratio of Maximum / Minimum Residual Density ....       2.02 Report
PLAT220_ALERT_2_C Non-Solvent  Resd 2  C   Ueq(max)/Ueq(min) Range        3.9 Ratio
PLAT222_ALERT_3_C Non-Solv.  Resd 2  H   Uiso(max)/Uiso(min) Range        4.9 Ratio
PLAT906_ALERT_3_C Large K Value in the Analysis of Variance ......      6.479 Check
PLAT911_ALERT_3_C Missing FCF Refl Between Thmin & STh/L=    0.600          2 Report
PLAT971_ALERT_2_C Check Calcd Resid. Dens.  0.90A   From Ru2             2.50 eA-3
PLAT971_ALERT_2_C Check Calcd Resid. Dens.  0.98A   From Ru2             2.22 eA-3
```

### Alert level G

```
PLAT720_ALERT_4_G Number of Unusual/Non-Standard Labels ..........          4 Note
PLAT910_ALERT_3_G Missing # of FCF Reflection(s) Below Theta(Min).          2 Note
PLAT933_ALERT_2_G Number of OMIT Records in Embedded .res File ...          3 Note
PLAT978_ALERT_2_G Number C-C Bonds with Positive Residual Density.          1 Info
```

0 **ALERT level A** = Most likely a serious problem - resolve or explain
2 **ALERT level B** = A potentially serious problem, consider carefully
7 **ALERT level C** = Check. Ensure it is not caused by an omission or oversight
4 **ALERT level G** = General information/check it is not something unexpected

0 ALERT type 1 CIF construction/syntax error, inconsistent or missing data
8 ALERT type 2 Indicator that the structure model may be wrong or deficient
4 ALERT type 3 Indicator that the structure quality may be low
1 ALERT type 4 Improvement, methodology, query or suggestion
0 ALERT type 5 Informative message, check

It is advisable to attempt to resolve as many as possible of the alerts in all categories. Often the minor alerts point to easily fixed oversights, errors and omissions in your CIF or refinement strategy, so attention to these fine details can be worthwhile. In order to resolve some of the more serious problems it may be necessary to carry out additional measurements or structure refinements. However, the purpose of your study may justify the reported deviations and the more serious of these should normally be commented upon in the discussion or experimental section of a paper or in the "special_details" fields of the CIF. checkCIF was carefully designed to identify outliers and unusual parameters, but every test has its limitations and alerts that are not important in a particular case may appear. Conversely, the absence of alerts does not guarantee there are no aspects of the results needing attention. It is up to the individual to critically assess their own results and, if necessary, seek expert advice.

**Publication of your CIF in IUCr journals**

A basic structural check has been run on your CIF. These basic checks will be run on all CIFs submitted for publication in IUCr journals (*Acta Crystallographica*, *Journal of Applied Crystallography*, *Journal of Synchrotron Radiation*); however, if you intend to submit to *Acta Crystallographica Section C* or *E* or *IUCrData*, you should make sure that full publication checks are run on the final version of your CIF prior to submission.

**Publication of your CIF in other journals**

Please refer to the *Notes for Authors* of the relevant journal for any special instructions relating to CIF submission.

---

**PLATON version of 23/04/2018; check.def file version of 23/04/2018**

**Datablock K1** - ellipsoid plot

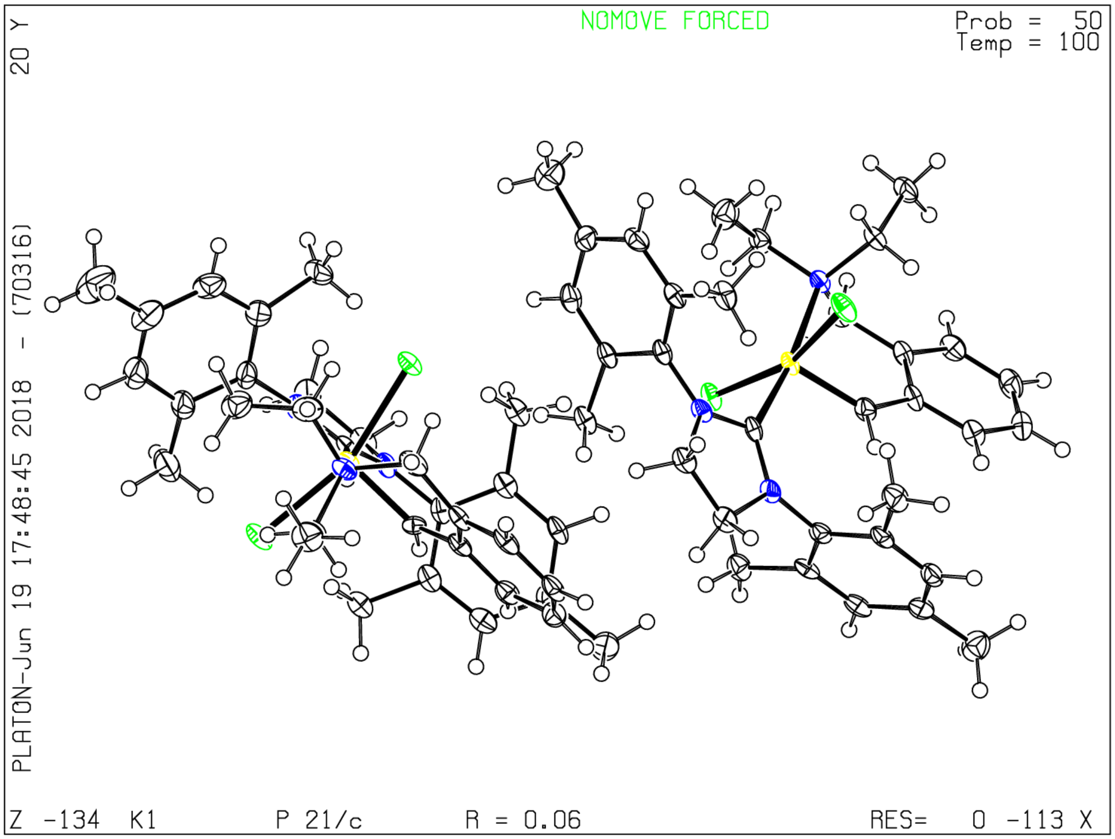

# checkCIF/PLATON report

Structure factors have been supplied for datablock(s) FZ1

THIS REPORT IS FOR GUIDANCE ONLY. IF USED AS PART OF A REVIEW PROCEDURE FOR PUBLICATION, IT SHOULD NOT REPLACE THE EXPERTISE OF AN EXPERIENCED CRYSTALLOGRAPHIC REFEREE.

No syntax errors found.    CIF dictionary    Interpreting this report

# Datablock: 11c

---

```
Bond precision:  C-C = 0.0090 A          Wavelength=0.71073

Cell:           a=12.9054(12)     b=10.8240(11)      c=21.684(2)
                alpha=90          beta=104.828(6)    gamma=90
Temperature:    150 K

                Calculated                 Reported
Volume          2928.1(5)                  2928.1(5)
Space group     P 21/n                     P 21/n
Hall group      -P 2yn                     -P 2yn
Moiety formula  C30 H37 Cl2 N3 Ru          C30 H37 Cl2 N3 Ru
Sum formula     C30 H37 Cl2 N3 Ru          C30 H37 Cl2 N3 Ru
Mr              611.60                     611.59
Dx,g cm-3       1.387                      1.387
Z               4                          4
Mu (mm-1)       0.741                      0.741
F000            1264.0                     1264.0
F000'           1260.45
h,k,lmax        15,13,26                   15,13,26
Nref            5796                       5778
Tmin,Tmax       0.899,0.949                0.885,0.941
Tmin'           0.875

Correction method= # Reported T Limits: Tmin=0.885 Tmax=0.941
AbsCorr = MULTI-SCAN

Data completeness= 0.997          Theta(max)= 26.074

R(reflections)= 0.0581( 3076)     wR2(reflections)= 0.1173( 5778)

S = 0.985                  Npar= 332
```

---

The following ALERTS were generated. Each ALERT has the format
**test-name_ALERT_alert-type_alert-level**.
Click on the hyperlinks for more details of the test.

## Alert level C

```
PLAT234_ALERT_4_C Large Hirshfeld Difference N3'      --C4'             0.17 Ang.
PLAT342_ALERT_3_C Low Bond Precision on  C-C Bonds ...............   0.00904 Ang.
PLAT906_ALERT_3_C Large K Value in the Analysis of Variance ......     2.077 Check
PLAT910_ALERT_3_C Missing # of FCF Reflection(s) Below Theta(Min).         6 Note
PLAT911_ALERT_3_C Missing FCF Refl Between Thmin & STh/L=    0.600         2 Report
PLAT978_ALERT_2_C Number C-C Bonds with Positive Residual Density.         0 Info
```

## Alert level G

```
PLAT007_ALERT_5_G Number of Unrefined Donor-H Atoms .............          1 Report
PLAT720_ALERT_4_G Number of Unusual/Non-Standard Labels ..........         3 Note
PLAT793_ALERT_4_G Model has Chirality at N3'         (Centro SPGR)         R Verify
PLAT912_ALERT_4_G Missing # of FCF Reflections Above STh/L=  0.600        10 Note
PLAT933_ALERT_2_G Number of OMIT Records in Embedded .res File ...         6 Note
```

```
   0 ALERT level A = Most likely a serious problem - resolve or explain
   0 ALERT level B = A potentially serious problem, consider carefully
   6 ALERT level C = Check. Ensure it is not caused by an omission or oversight
   5 ALERT level G = General information/check it is not something unexpected

   0 ALERT type 1 CIF construction/syntax error, inconsistent or missing data
   2 ALERT type 2 Indicator that the structure model may be wrong or deficient
   4 ALERT type 3 Indicator that the structure quality may be low
   4 ALERT type 4 Improvement, methodology, query or suggestion
   1 ALERT type 5 Informative message, check
```

It is advisable to attempt to resolve as many as possible of the alerts in all categories. Often the minor alerts point to easily fixed oversights, errors and omissions in your CIF or refinement strategy, so attention to these fine details can be worthwhile. In order to resolve some of the more serious problems it may be necessary to carry out additional measurements or structure refinements. However, the purpose of your study may justify the reported deviations and the more serious of these should normally be commented upon in the discussion or experimental section of a paper or in the "special_details" fields of the CIF. checkCIF was carefully designed to identify outliers and unusual parameters, but every test has its limitations and alerts that are not important in a particular case may appear. Conversely, the absence of alerts does not guarantee there are no aspects of the results needing attention. It is up to the individual to critically assess their own results and, if necessary, seek expert advice.

**Publication of your CIF in IUCr journals**

A basic structural check has been run on your CIF. These basic checks will be run on all CIFs submitted for publication in IUCr journals (*Acta Crystallographica*, *Journal of Applied Crystallography*, *Journal of Synchrotron Radiation*); however, if you intend to submit to *Acta Crystallographica Section C* or *E* or *IUCrData*, you should make sure that full publication checks are run on the final version of your CIF prior to submission.

**Publication of your CIF in other journals**

Please refer to the *Notes for Authors* of the relevant journal for any special instructions relating to CIF submission.

# PLATON version of 20/08/2018; check.def file version of 20/08/2018

**Datablock FZ1** - ellipsoid plot

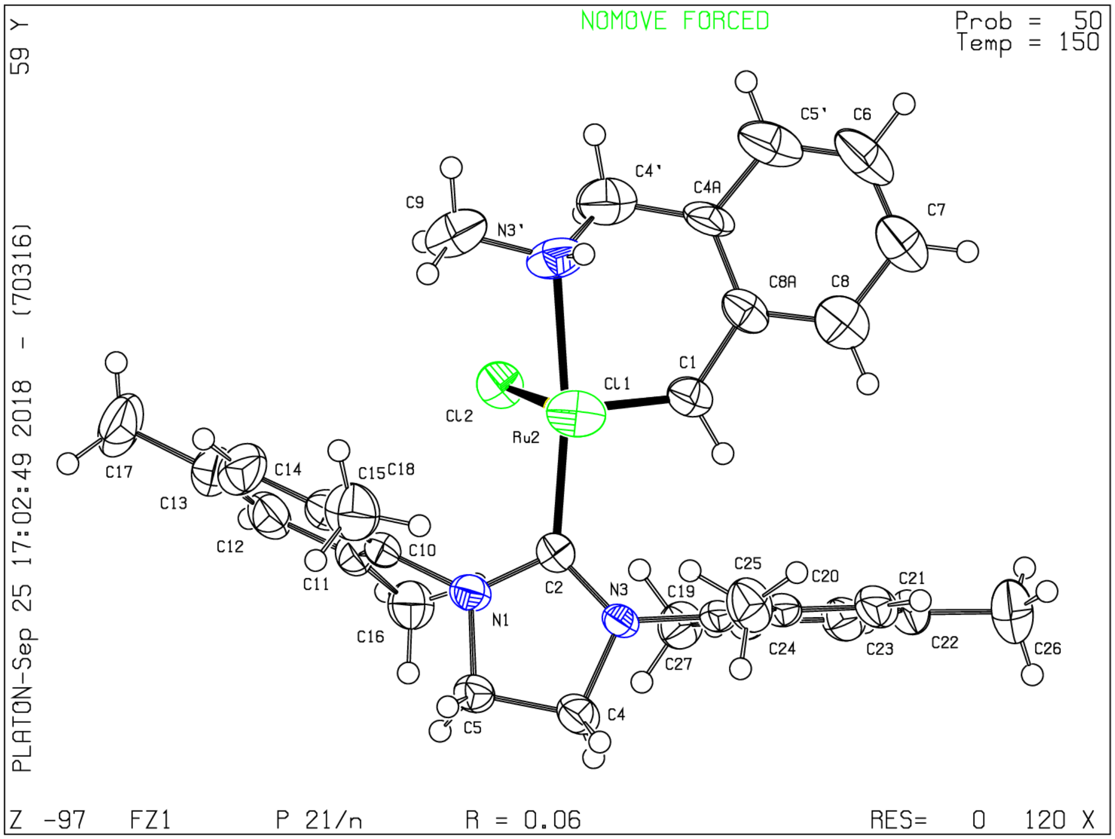

Supplement: File 2 — Copies of NMR spectra of synthesised compounds and selected GC–MS data of the metathesis products. Check-cif reports for compounds 11a–c. [file Beilstein_J_Org_Chem-15-769-s002.pdf]
